# Intramolecular substitutions of secondary and tertiary alcohols with chirality transfer by an iron(III) catalyst

**DOI:** 10.1038/s41467-019-11838-x

**Published:** 2019-08-23

**Authors:** Rahul A. Watile, Anon Bunrit, Jèssica Margalef, Sunisa Akkarasamiyo, Rabia Ayub, Emi Lagerspets, Srijit Biswas, Timo Repo, Joseph S. M. Samec

**Affiliations:** 1 Department of Organic Chemistry, Stockholm University, 106 91 Stockholm, Sweden; 2 Department of Chemistry, University of Helsinki, A. I. Virtasen aukio 1, P.O. Box 55, 00014 Helsinki, Finland; 3 Department of Chemistry, University College of Science, University of Calcutta, 700 009 Kolkata, West Bengal India

**Keywords:** Homogeneous catalysis, Stereochemistry, Synthetic chemistry methodology

## Abstract

Optically pure alcohols are abundant in nature and attractive as feedstock for organic synthesis but challenging for further transformation using atom efficient and sustainable methodologies, particularly when there is a desire to conserve the chirality. Usually, substitution of the OH group of stereogenic alcohols with conservation of chirality requires derivatization as part of a complex, stoichiometric procedure. We herein demonstrate that a simple, inexpensive, and environmentally benign iron(III) catalyst promotes the direct intramolecular substitution of enantiomerically enriched secondary and tertiary alcohols with *O*-, *N*-, and *S*-centered nucleophiles to generate valuable 5-membered, 6-membered and aryl-fused 6-membered heterocyclic compounds with chirality transfer and water as the only byproduct. The power of the methodology is demonstrated in the total synthesis of (+)-lentiginosine from D-glucose where iron-catalysis is used in a key step. Adoption of this methodology will contribute towards the transition to sustainable and bio-based processes in the pharmaceutical and agrochemical industries.

## Abstract (web summary)

The direct substitution of the OH group of stereogenic alcohols are reported rarely in literature. Here, the authors demonstrate direct substitution of both secondary and tertiary alcohols with chirality transfer leading to enantioenriched 5-membered, 6-membered and aryl-fused 6-membered heterocyclic compounds.

## Introduction

Finding efficient and atom-economical strategies for directly substituting the OH group in stereogenic alcohols is a major challenge in synthetic organic chemistry^1–4^. Traditional methods are usually multistep and rely on the use of hazardous reagents in stoichiometric amounts, often leading to tedious purifications^5,6^. An example of the state-of-the-art transformations is the Mitsunobu reaction^7^, which is based on the stoichiometric use of an azodicarboxylate and triphenylphosphine. Alternative methodologies are, therefore, searched for and particularly the direct substitution of non-derivatized alcohols has long been viewed as one of the most desired chemical transformations for which pharmaceutical companies want greener alternatives^8–10^. As potential applications for direct substitution of alcohols are found in both pharmaceutical and agricultural products, where only one of the enantiomers has the desired biological effect, a special emphasis on stereoselective and stereospecific substitutions has been given^10^. The main benefits with a direct substitution are atom economy, one-step procedure, less reagents, and non-derivatized intermediates.

**Fig. 1:**
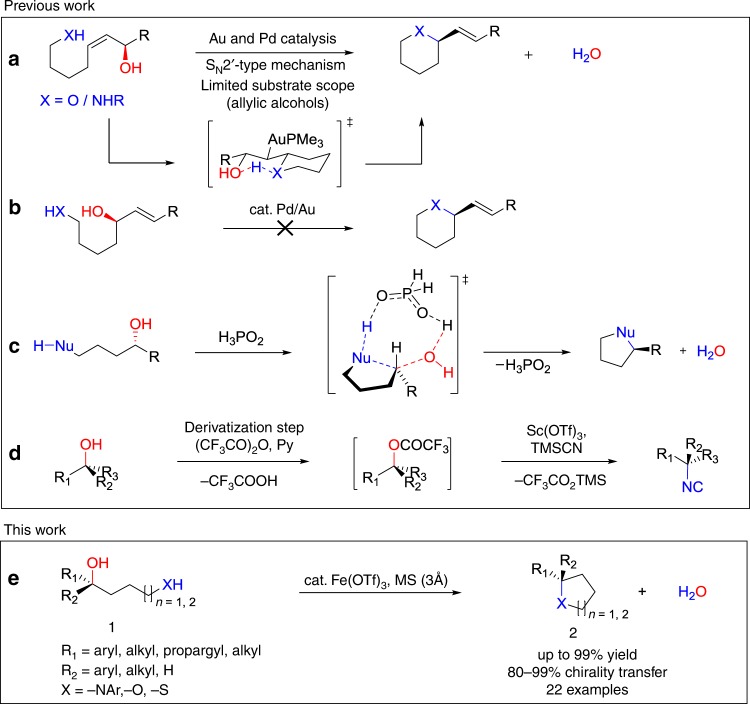
Substitution of the OH groups of enantioenriched alcohols with chirality transfer. **a**, **b**, Gold-catalyzed and palladium-catalyzed S_N_2′-type allylic amination and etherification reactions. **c** Phosphinic acid-catalyzed S_N_2-type intramolecular substitution of the OH groups of secondary alcohols. **d** Stereoinversion of tertiary alcohols by tertiary-alkyl isonitriles. **e** Iron-catalyzed intramolecular substitution with chirality transfer of the OH groups of enantioenriched secondary and tertiary alcohols

Whereas direct substitutions of the OH group in alcohols has been achieved through an S_N_1-type mechanism and these reactions have been thoroughly explored in the past decades^11^, reports on plausible highly stereoselective S_N_2-like reactions are relatively rare^11–13^. Only recently have S_N_2′-type reactions been reported for gold-catalyzed and palladium-catalyzed intramolecular amination and etherification reactions of stereogenic allylic alcohols (Fig. 1a)^14–21^. However, these reactions are substrate dependent and if the double bond and OH group was juxtapositioned, the reactivity collapsed (Fig. 1b)^22,23^. In this context, we recently achieved direct intramolecular S_N_2-like reaction of underivatized stereogenic alcohols to generate heterocycles by using a phosphinic acid catalyst, that reaction was limited to secondary alcohols, strong nucleophiles, and substrates giving kinetically and thermodynamically favored five-membered products (Fig. 1c)^1,24^.

The substitution of the OH group in enantioenriched tertiary alcohols with chirality transfer is even more challenging, nevertheless very important as many biologically active compounds have tertiary structures^25,26^. As state of the art, a substitution with transfer of chirality was only achieved via in situ derivatization of alcohols to trifluoro acetate derivatives (Fig. 1d) (Terminology of such transformation is not settled by the community and both stereospecific and chirality transfer are used. In this article, chirality transfer will be used.) Interestingly, it was found that the protocol was chemoselective for tertiary alcohols over secondary and primary alcohols^27^.

Despite these recent advances emphasized above, there are no reported methods on either the synthesis of non-allylic six-membered heterocyclic compounds through intramolecular substitution of the OH group or the direct intramolecular substitution of tertiary alcohols with chirality transfers, or the use of substrates containing weak nucleophiles (such as phenolic OH)^28^. In order to offer a synthetic route to overcome these limitations, we continued our efforts on acid-catalyzed transformations and report herein a Fe(OTf)_3_-catalyzed intramolecular substitution of enantioenriched secondary and tertiary alcohols by *N-*, *O-* and *S-*centered nucleophiles with high degree of chirality transfer. Accordingly, this approach gives a high yield route to enantioenriched pyrrolidine, tetrahydrofuran, tetrahydrothiophene, 1,2,3,4-tetra-hydroquinoline, and chromane derivatives that are core structures found in several biologically active compounds important for pharmaceutical and agricultural industries (Fig. 1e).

## Results

### Screening of Lewis acid catalysts and reaction conditions

**Fig. 2:**
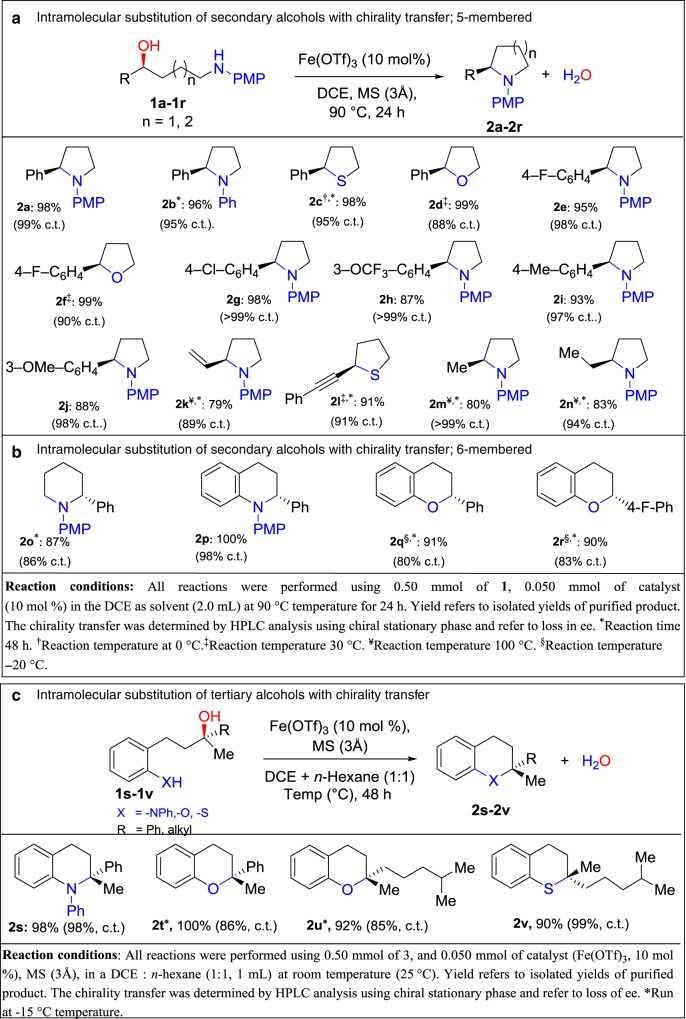
Intramolecular substitution of the OH group of alcohols with chirality transfer. **a**, **b** General reaction scheme, products obtained, and reaction conditions for the intramolecular substitution of secondary alcohols with chirality transfer to five-membered and six-membered rings, respectively. **c** General reaction scheme, products obtained, and reaction conditions employed in the intramolecular substitution of tertiary alcohols with chirality transfer

For the initial screening of chirality transfer, we selected 4-((4-methoxyphenyl)amino)-1-phenylbutan-1-ol (**1a**) (Table 1) as a model substrate for the intramolecular substitution reaction to yield 1-(4-methoxyphenyl)-2-phenylpyrrolidine (**2a**). The *p-*methoxyphenyl (PMP) group was introduced on the nitrogen to enhance its nucleophilicity and thus facilitate the substitution reaction. In addition, the PMP has further synthetic advantages because it can be removed after the reaction if desired (Fig. 2)^29,30^.

**Table 1:** Selected optimization of the reaction conditions for benzylic alcohols


Entry	Catalysts	*T* (°C)	Yield (%)^a^	c.t. (%)^b^
1	FeF_3_(III)	90	15	0
2	FeCl_2_(II)	90	20	92
3	Fe(NO_3_)_3_·(H_2_O) (III)	90	NR	0
4	Fe(acac)_3_(III)	90	NR	0
5	Fe_4_[Fe(CN)_6_]_3_	90	NR	0
6	Fe(ClO_4_)_2_·4H_2_O	90	10	91
7	Fe(EDTA) sodium salt	90	NR	0
8	Fe_2_O_3_	90	10	93
9	FeCl_3_(III)	90	35	92
10	Fe(OTf)_2_(II)	90	29	90
11	Iron (III) tartrate	90	NR	0
12	Fe(OTf)_3_(III)	90	62	96
13	Fe(OTf)_3_	110	65	80
**14**	**Fe(OTf)**_**3**_^**+**^ **MS (3** **Å)**	**90**	>**95**	**99**
15	MS (3 Å)	90	NR	0

*Reaction conditions*: All reactions were performed using 0.5 mmol of **1a**, 0.050 mmol of catalyst (10 mol%), and 300 mg of MS (3 Å) in DCE as the solvent (2.0 mL) at 90 °C for 24 h under an argon atmosphere. NR indicates no reaction

^a^NMR yield using 1,3,5-trimethoxybenzene as an internal standard

^b^Chirality transfer (c.t.) was determined by HPLC analysis with a chiral stationary phase and refer to loss of ee

Bold entry indicates the optimized conditions used

Initially, the catalytic reactivity and chirality transfer of different Lewis acids were screened with model compound **1a**, from those oxophilic iron-based catalysts gave higher yields and chirality transfers than other Lewis acids in this series (Supplementary Table 2). (Chirality transfer is determined as percentage of conserved ee.) In further studies, the most Lewis acidic complex, Fe(OTf)_3_, was found to be the most efficient catalyst for the transformation, and it gave desired pyrrolidine **2a** in 62% yield (Table 1, entry 12). Accordingly, high Lewis acidity is crucial for activating the C–O bond towards nucleophilic attack and the counter ion had a profound effect on both the yield and the chirality transfer (Table 1, entries 1–12). To further improve the efficiency of the reaction, different additives were studied. As a result, the addition of molecular sieves (MS) (3 Å) improved both the yield and chirality transfer of the transformation (Table 1, entry 14). A control reaction showed that the MS did not promote the reaction in the absence of Fe(OTf)_3_ (Table 1, entry 15). The role of the MS is to suppress hydrolysis of Fe(OTf)_3_ species by generated water and thus prevent catalyst deactivation.

The influences of solvent and reaction temperature were also studied. The optimal solvent was found to be 1,2-dichloroethane (DCE) in terms of both reactivity and chirality transfer (Supplementary Table 3). We observed a correlation between conversion and the reaction temperature up to 90 °C, but further increases in the temperature decreased the chirality transfer (Table 1, entry 13). Thus, using 10 mol% Fe(OTf)_3_ in DCE at 90 °C generated **2a** in a quantitative yield with 99% chirality transfer (Table 1, entry 14). Based on the results above, the Fe(OTf)_3_ catalyst have a well-tuned reactivity where the C–O bond is activated for nucleophilic attack. Notably, under optimized conditions the C–O bond is cleaved in the presence of an intramolecular nucleophile leading to chirality transfer.

### Synthesis of (+)-lentiginosine

**Fig. 3:**
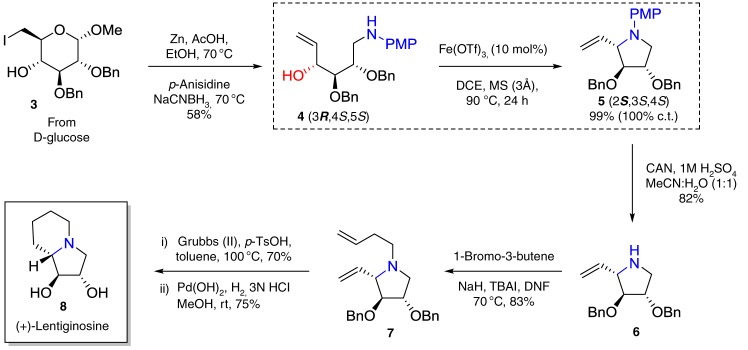
Total synthesis of (+)-lentiginosine. 100% enantiospecificity in the key step of the synthesis of (+)-lentiginosine, a five-membered heterocyclic ring

After determining the optimized reaction conditions, we investigated the substrate scope. *N-*, *O-*, and *S-*centered nucleophiles were utilized in the intramolecular substitution of various enantioenriched benzylic alcohols with chirality transfer to generate five-membered heterocyclic derivatives (Fig. 3). Substrates with *N-, O-*, and *S-*centered nucleophiles generated products in excellent yields and with high chirality transfers (Fig. 3a, entries **2a**–**2j**). We extended our studies to non-benzylic alcohols. As mentioned in the “Introduction” section, allylic alcohols, where the nucleophile cannot protonate the leaving OH group, were not tolerated by previous protocols (Fig. 1b)^24,25^. Overcoming the restrictions of previous strategies, using the iron catalyst we found allylic alcohol **1k** furnished **2k** in 79% yield and 89% chirality transfer with the iron catalyst (Fig. 3a, entry **2k**). Interestingly, the present method was appropriate for less reactive propargyl alcohol **1l**, generating enantioenriched 2-(phenylethynyl)tetrahydrothiophene (**2l**) in a 98% yield and 91% chirality transfer (Fig. 3a, entry **2l**).

Next, we examined non-activated alkyl alcohols. For the reaction to proceed with less reactive aliphatic alcohols, the reaction temperature was increased to 100 °C. The *N*-centered nucleophiles **1m** and **1n** generated enantioenriched 2-methyl-1-phenylpyrrolidine (**2m**) and 2-ethyl-1-phenylpyrrolidine (**2n**), respectively, in good yields and with high degrees of chirality transfer (Fig. 3a, entries **2m** and **2n**). This result is notable as to date, only a few methods are available for direct nucleophilic substitution of non-activated aliphatic alcohols, and those reactions proceed through S_N_1 pathways to give racemic products^31–37^. One challenge with aliphatic alcohols compared to π-activated alcohols is that competing elimination processes can occur.

To demonstrate the utility of the intramolecular substitution reaction with chirality transfer, we completed the total synthesis of (+)-lentiginosine^38^ (**8**, Fig. 2) from optically pure d-glucose. The key step in the synthesis was the intramolecular substitution of **4** in the presence of Fe(OTf)_3_ to furnish **5** (Fig. 2) in 99% yield with complete inversion of configuration (100% chirality transfer). The removal of PMP from optically pure **5** using CAN gave **6** in 82% yield without erosion of the enantiomeric excess. Subsequent *N*-allylation afforded **7**, which smoothly underwent ring closing metathesis. The metathesis product was hydrogenated to afford (+)-lentiginosine (**8**). Thereby, we have prepared (+)-lentiginosine in only 10 steps from the most available natural building block d-glucose and thus we have shortened the synthetic route considerably from previously reported (18 steps)^39^.

### Formation of six-membered heterocycles

Although with the substrates above, there was only gradual improvement in reactivity and selectivity in comparison to our previous phosphinic acid studies, with Fe(OTf)_3_ catalyst we were able to extend the substrate scope markedly. In the present study, six-membered and aryl-fused six-membered heterocyclic compounds can be generated in excellent yields with good chirality transfers. This class of heterocyclic compounds are prevalent in pharmaceuticals and agrochemicals and their synthesis through direct catalytic substitution with chirality transfer is not reported in literature. In these cases, longer reaction times were required to obtain good yields and chirality transfers (Fig. 3b, entries **2o**–**2p**). Furthermore, substrates containing poor nucleophiles (phenolic-*O* nucleophiles) showed full conversion and good chirality transfers (Fig. 3b, entries **2q**–**2r**). Notably, this is the first time a non-activated phenol has been used as a nucleophile in the substitution of an OH group with transfer of chirality.

### Substitution reactions of underivatized tertiary alcohols

Prompted by the good results achieved with secondary alcohols, the more challenging tertiary alcohols were next examined. Substitution reactions of tertiary alcohols with chirality transfer have all, to date, required a stoichiometric-activating agent to promote the substitution^5,6^, and furthermore, the substitution reactions have been restricted with respect to nucleophiles. We report here a range of six-membered heterocyclic compounds produced from underivatized tertiary alcohols in excellent yield and with high chirality transfer (see Fig. 3c).

We initially performed the intramolecular substitution of *N-*centered enantioenriched tertiary alcohol **1s** using the same reaction conditions as were used for secondary alcohols. While the yield of **2s** was quantitative, the chirality transfer was low (Supplementary Table 3, entry 1, c.t. 9%). The key to achieving high chirality transfer turned out to be decreasing the polarity of the solvent system. By using a mixture of n-hexane and DCE, as well as optimizing the temperature and time (Supplementary Table 3) the chirality transfer was increased to 98% (Fig. 3c, entry 4a). This is the first example of a substitution reaction of the OH group of a tertiary alcohol with chirality transfer without prior derivatization.

We were also able to apply this protocol to tertiary alcohols containing weak nucleophiles. Substrate **1t**, having a phenolic *O-*nucleophile, provided full conversion, albeit initially only moderate chirality transfer. Lowering the temperature of the reaction to −15 °C increased the chirality transfer of the reaction while still providing full conversion (Fig. 3c, entry **2t**). Finally, we investigated sterically hindered and easily dehydrated aliphatic tertiary alcohols in the intramolecular nucleophilic substitution reaction (Fig. 3c, entries **2u** and **2v**). These are very challenging substrates known to undergo elimination reactions and chosen to demonstrate any limitations of the reaction. Aliphatic tertiary alcohols **1u** and **1v** generated 2-methyl-2-(4-methylpentyl)chromane (**2u**) in 92% yield and 85% chirality transfer and (S)-2-methyl-2-(4-methylpentyl)thiochromane (**2v**) in 90% yield and 99% chirality transfer, respectively (Fig. 3c, entries **2u** and **2v**). This is noteworthy where for substrate **2u**, the combination of a tertiary alcohol prone to undergo elimination with an extremely poor nucleophilic phenol generates products in high chirality transfers. The product resembles the core of Vitamin E, and thus has biological significance. As shown above, the inherent property of Fe(OTf)_3_ to activate underivatized and tertiary alcohols opens a truly attractive and general approach to synthesize five-membered, six-membered, and aryl-fused six-membered heterocyclic compounds in excellent yield and with high chirality transfer.

### Mechanistic studies

The intramolecular substitution reactions reported here could, theoretically, proceed by a spectra of S_N_1- or S_N_2-like reaction mechanisms, but to distinguish them is challenging. The electrophile, or the nucleofuge in this work, can directly be substituted in an S_N_2-like pathway or can be ionized in a rate-limiting step without nucleophilic assistance proceeding through an S_N_1 pathway. In addition, a substitution of nucleofuges can occur with different pathways, including nucleophile-assisted ionization, contact ion pair, solvent-separated ion pair, and free carbocation^40^. As a result, mechanistic spectra can be very complex.^41–46^ In general, rate order and chirality transfer are used to distinguish between S_N_1 and S_N_2 reaction pathways^1,47^, but the intramolecular substitution reactions set inherent limitations. Firstly, the rate order is not possible to measure because the electrophile and nucleophile are present in the same molecule, and therefore, it is impossible to vary the concentration of the individual species. To meet this challenge, we synthesized substrate analogs with two equivalent nucleophiles in the molecule, as detailed below. Secondly, using the chirality transfer to draw conclusions about the reaction mechanism is not straightforward, as an S_N_1 reaction that proceeds through a tight ion pair can give a substituted product with perfect transfer of chirality.

### Kinetic studies

To elucidate the reaction mechanism of the present transformations, we studied the rate order of the Fe(OTf)_3_ catalyst. The rate-order was determined by varying the concentration of catalyst in the transformation of **1a**–**2a**, and a first-order dependence on catalyst concentration was found (Supplementary Fig. 1).

**Fig. 4:**
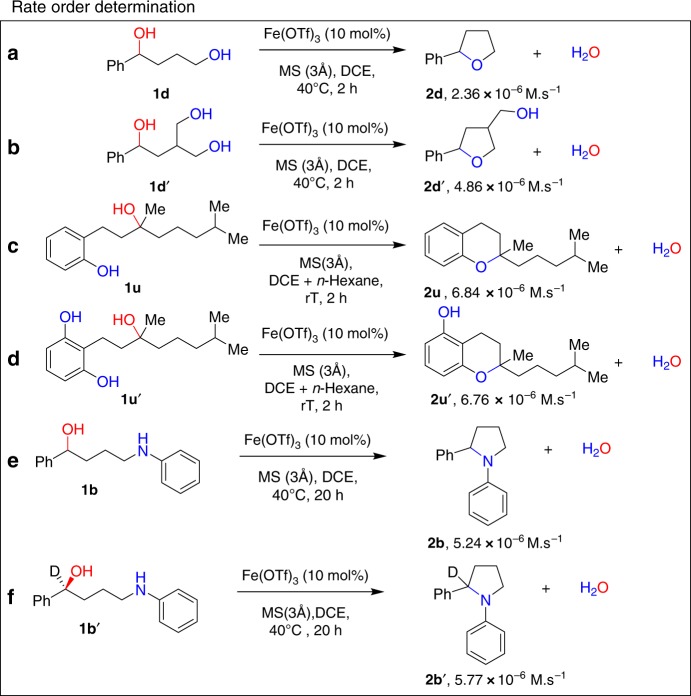
Control experiments. **a**–**d** Comparative studies for the rate-order determination of the reaction with respect to nucleophiles. *Reaction conditions*: The alcohol (0.5 mmol), DCE (2 mL), and catalyst (10 mol%) were heated in an oil bath at the desired temperature. Initial rates were determined at conversions below 20% (up to 2 h) by ^1^H NMR spectroscopy. Each value is the mean of two runs. **e** and **f** show rate constant and deuterium kinetic isotope effect for **1b** and **1b**′

The overall rate in an S_N_1 reaction is only dependent on the concentration of the electrophile due to the rate-limiting generation of the carbenium ion, whereas an S_N_2 reaction is dependent on both the electrophile and nucleophile. As we could not vary the concentration of the electrophile and nucleophile, we prepared substrates with two nucleophiles present instead of only one (**1d**′ and **1u**′, Fig. 4). Even though this does not show the rate-order, it might give useful information whether the kinetics are governed by the nucleophile. It should be noted that this experimental set-up does not follow first principles and the results should be interpreted with care. As a result, we found that the reaction rate was doubled when two nucleophiles were present on the molecule (**1d**′) instead of only one (**1d**) (Fig. 4a, b). Similarly, the reaction rate with a substrate with two electrophilic moieties was twice that of the standard substrate (Supplementary Fig. 2). Moreover, rate constant and deuterium kinetic isotope effect (KIE) was also measured for substrates **1b** and **1b**′ (Fig. 4e, f and Supplementary Fig. 4). A deuterium KIE (KIE = *k*_H_/*k*_D_ = 0.91) was observed when the hydrogen atom on **1b** is replaced by deuterium and the reactions were performed under similar conditions. This inverse KIE also support an S_N_2-like pathway. Even though the full rate-order is not elucidated, these observations support an S_N_2-like reaction mechanism for secondary alcohols where one equivalent of iron is responsible for the catalysis^47^.

To continue our mechanistic investigations, we introduced tertiary alcohols **1u** and its derivative **1u**′ with two nucleophiles for comparison (Fig. 4c, d). Now the reaction rate did not show a rate increase for **1u**′ and indicates that the reaction mechanism for the tertiary alcohol is different than for secondary alcohols, likely following an S_N_1 pathway.

In our previous report on phosphinic acid-catalyzed intramolecular substitution of alcohols^24^, it was found that, in the absence of an internal nucleophile, iron(III) promotes an S_N_1-type reaction, yielding a racemic mixture of the corresponding product. In other words, iron promotes C−O bond cleavage; however, in the absence of an internal nucleophile, a solvent-separated carbenium ion is generated before being attacked by another molecule of the substrate via an S_N_1-type reaction. In the present case, the tethering of the nucleophile to the molecule enables the reaction to proceed through an S_N_2-like mechanism, inducing the high chirality transfer.

### Clarification of the mechanism by theoretical calculations

On the basis of the experimental results, an S_N_2-like mechanism seems to be feasible for secondary benzylic alcohols. To get further insights to the reaction mechanisms we decided to study the reaction involving substrates **1a**, **c**, and **d** representing the three nucleophiles and secondary benzyl alcohol used in this study by DFT calculations (B3LYP/6-31+G**/LANL2DZ). Since first-row transition metals have partially occupied *d*-orbitals, several spin states might be energetically accessible, depending on the oxidation state of the metal and its coordination sphere. Furthermore, the most favorable spin state can change during the course of the reaction^48,49^. Hence, we studied the transformation of **1d** to **2d** by initially considering all three possible spin states for iron (III) (doublet, quartet, and sextet). FeCl_3_ was chosen as a model to mimic the Fe(OTf)_3_ used in the experiments as it also was found to promote the substitution (Table 1, entry 9) and FeCl_3_ gave more coherent results as compared to Fe(OTf)_3_.

**Fig. 5:**
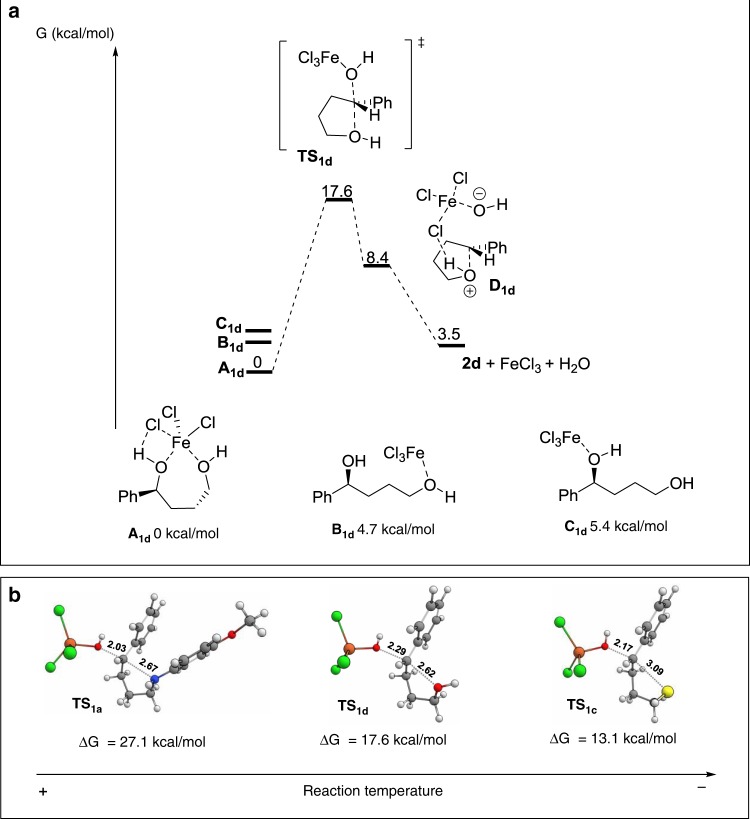
DFT studies. **a** Structures for intermediate iron complexes **A, B** and **C** generated from interactions between FeCl_3_ and **1d** and their corresponding energy profiles for the intramolecular S_N_2-type nucleophilic substitution reaction. **b** Structures and energies for substrates **1a**, **c**, and **d** in the transition states (TS)

The sextet spin state was found to be preferred over the quartet and doublet spin states by 20 and 40 kcal/mol, respectively, for all computed structures (Supplementary Fig. 5). The energy profile for the intramolecular nucleophilic substitution of **1d** is depicted in Fig. 5a. First, Lewis acidic FeCl_3_ can coordinate to the hydroxyl group attached the benzylic position giving Fe species **C**_**1d**_ or to the primary alcohol group giving Fe species **B**_**1d**_. They are both in similar energy but only **C**_**1d**_ can facilitate the nucleophilic attack in an S_N_2 fashion that leads to transition state **TS**_**1d**_ with an activation free energy of 17.6 kcal/mol. Finally, product **2d** and water are released, regenerating the iron (III) catalyst. A third possible Fe species **A**_**1d**_, where both alcohol groups coordinate to FeCl_3_ is 5 kcal/mol more stable than **B**_**1d**_ and **C**_**1d**_ and can be considered rather as a resting state of the catalysis.

We next studied the transformation of substrates **1a** and **1c** (Fig. 5, Supplementary Fig. 6). The energy profiles for both substrates are similar to those obtained for **1d** (Supplementary Fig. 6). However, in the resting state, the *p*-methoxyamino group prefers the non-coordinating mode, probably due to steric hindrance (Supplementary Fig. 6, intermediate **C**_**1a**_). The transition states found for each substrate are shown in Fig. 5b; substrate **1a** shows a substantially higher energy barrier than either **1c** or **1d**, which is consistent with the much higher reaction temperature required for **1a** (90 °C) than for **1c**, and **d** (30 and 0 °C, respectively).

Both the experimental data and DFT calculations support an S_N_2-type mechanism for secondary alcohols. However, an S_N_1 pathway through the formation of an ion pair still cannot be completely ruled out. Due to charge separation in the transition state of an S_N_1 reaction mechanism, calculations cannot be used for comparison. Accordingly, attempts to find transition states comprising tight-ion pairs failed. In the case of tertiary alcohols, the experimental results suggest that the coordination of iron to the nucleofuge would lead to C–O bond cleavage to generate a tight ion-pair intermediate. In nonpolar solvent mixtures, the ion pair remains tight, and nucleophilic attack generates the substitution products with high chirality transfer. However, in a polar solvent, the ion pairing is loosened, and nucleophilic attack can occur from either side, leading to a product with lower chirality transfer. This is in accordance with our experimental results and we propose that the reaction of secondary alcohols with poor nucleophiles that generate six-membered ethers and tertiary alcohols proceeds through an S_N_1-like mechanism in which the preservation of chirality from the substrate to the product is governed by the solvent polarity.

Therefore, the mechanistic picture of the present transformation is complex and substrate dependent, and for substrates that have less drive to promote an S_N_2-like mechanism, the reaction media can be altered to govern both the yield and enantiomeric purity of the product. Due to the favorable energetics of internal ring closures, the system is tunable for a wide range of nucleophiles and secondary and tertiary alcohols that can provide intermediates and products of pharmaceutical and agrochemical relevance.

## Discussion

We report herein a substitution of both secondary and tertiary alcohols leading to enantioenriched five-membered, six-membered, and aryl-fused six-membered heterocyclic compounds. Notably, this direct substitution method works for enantioenriched tertiary alcohols, where the chirality is preserved to the product and water is generated as the only by-product. The transformation is catalyzed by an abundant, inexpensive, non-toxic iron catalyst, with substrates comprising non-activated alkyl alcohols and poor uncharged nucleophiles can successfully be used. These advancements have enabled a green transformation of readily available alcohols as raw material in the synthesis of important motifs found in biologically active compounds important in pharmaceutical and agrochemical industries. We demonstrate the power of the substitution in 22 substrates with chirality transfer and as a key step in the total synthesis of (+)-lentiginosine, where all the three stereocenters in the natural product are derived from glucose. Mechanistic studies support that the reaction with secondary alcohols to generate five-membered heterocycles proceed through an S_N_2-like mechanism, while with tertiary alcohols, an S_N_1-type reaction mechanism comprising a tight ion pair would occur in less polar solvent mixtures. Overall, our approach provides an efficient and atom-economic strategy for substitution of non-derivatized stereogenic alcohols with transfer of chirality, thereby filling a major gap in the methodology of organic chemistry.

## Methods

### General procedure

To an oven-dried 5-ml vial equipped with a magnetic stir bar, the substrate amino alcohol **1a** (135.5 mg, 0.5 mmol), MS (3 Å; 300 mg), and Fe(OTf)_3_ (25.05 mg, 0.05 mmol) were added. The tube was sealed with a Teflon-lined cap, connected to a vacuum and backfilled with argon three times by piercing with a needle attached to a Schlenk line. Then, DCE (2.0 ml; anhydrous) was added by syringe, and the mixture was stirred at 90 °C for 24 h. After this, the reaction was cooled to room temperature, and the crude material was concentrated under vacuum. The crude residue was purified by column chromatography with ethyl acetate and hexanes (1:20) to obtain pure product **2a** (99%, 133 mg). Full experimental details and characterization of compounds are given in the Supplementary Information.

## Supplementary information

Peer Review

Supplementary Information

## Data Availability

The data that support the plots within this paper and other findings of this study, such as ^1^H NMR, ^13^C NMR, and HPLC spectra, as well as experimental procedures and quantum chemical calculations are available in the Supplementary Information.
