# Supplementary material for: Intramolecular substitutions of secondary and tertiary alcohols with chirality transfer by an iron(III) catalyst
Source: Nat Commun. 2019 Aug 23;10:3826. doi: 10.1038/s41467-019-11838-x (PMC6707304; doi:10.1038/s41467-019-11838-x)
Supplement: Supplementary file 2 — Supplementary Information [file 41467_2019_11838_MOESM2_ESM.pdf]

Supplementary Information

Iron(III)-catalyzed intramolecular substitutions of enantioenriched secondary and tertiary alcohols with chirality transfer

Watile, R. A. *et al*.

Supplementary Table 1. Checklist of characterization data of all compounds

a. *Checklist of characterization data of starting alcohols*

Code	Compound	New/ Known	^{1}H-NMR	^{13}C-NMR	IR	HRMS
**1a**	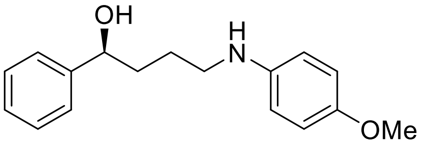	New	/	/	/	/
**1b**	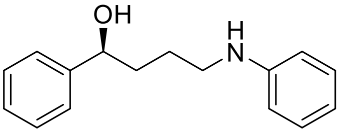	Known	/	/	-	-
**1b′**	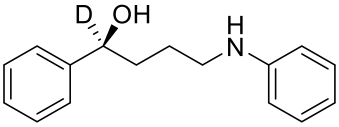	New	/	-	-	-
**1c**	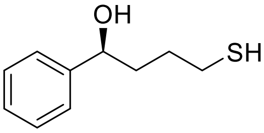	Known	-	-	-	-
**1d**	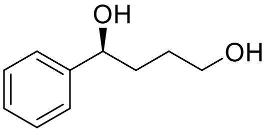	Known	/	/	-	-
**1e**	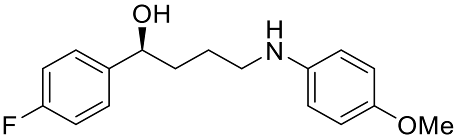	New	/	/	/	/
**1f**	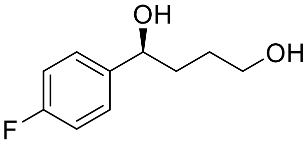	Known	/	/	-	-
**1g**	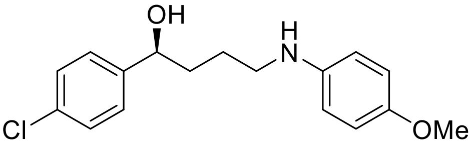	New	/	/	/	/
**1h**	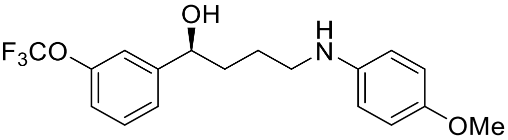	New	/	/	/	/
**1i**	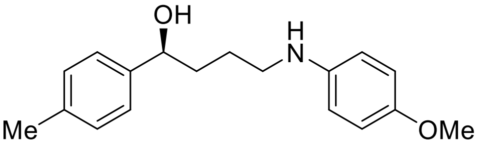	New	/	/	/	/
**1j**	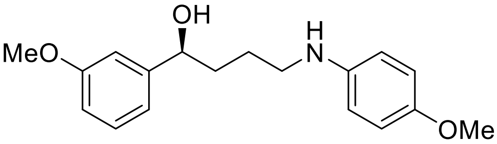	New	/	/	/	/

**1k**	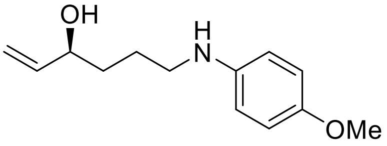	New	/	/	/	/
**1l**	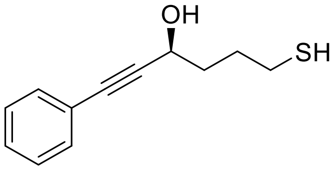	Known	/	/	-	-
**1m**	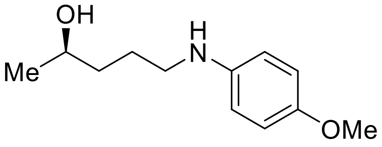	Known	/	/	-	-
**1n**	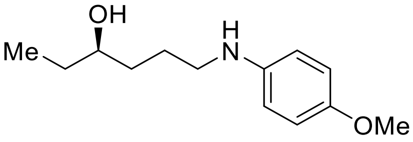	New	/	/	/	/
**1o**	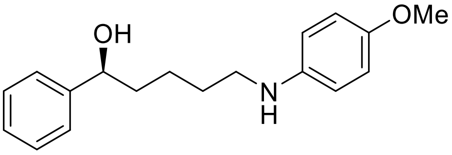	New	/	/	/	/
**1p**	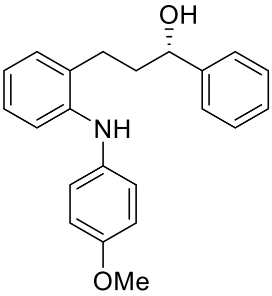	Known	/	/	/	/
**1q**	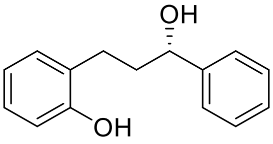	Known	/	/	-	-
**1r**	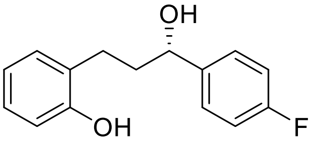	New	/	/	/	/
**1d′**	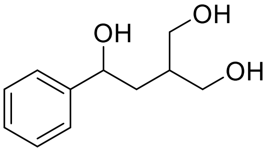	Known	/	/	/	/
**1d″**	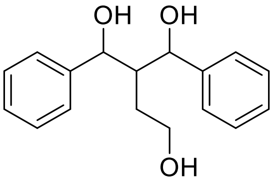	New	/	/	/	/

**1s**	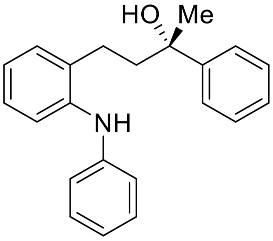	New	/	/	/	/
**1t**	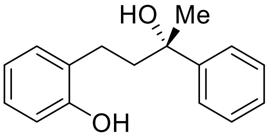	New	/	/	/	/
**1u**	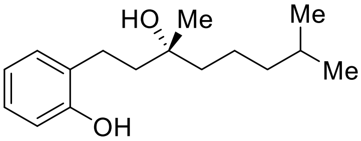	New	/	/	/	/
**1v**	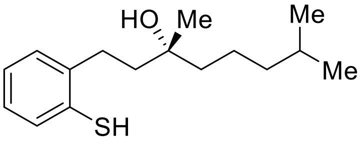	New	/	/	/	/
**1u′**	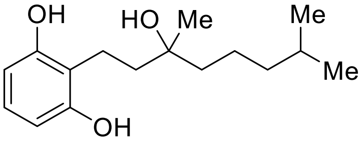	New	/	/	/	/

b. *Checklist of characterization data of products*

Code	Compound	New/ Known	^{1}H-NMR	^{13}C-NMR	IR	HRMS
**2a**	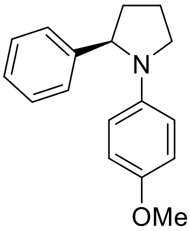	Known	/	/	/	/
**2b**	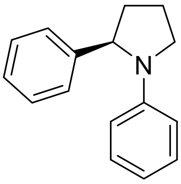	Known	/	/	/	/
**2c**	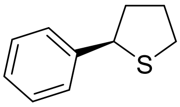	Known	/	/	-	-
**2d**	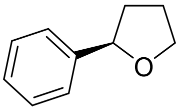	Known	/	/	-	-

**2e**	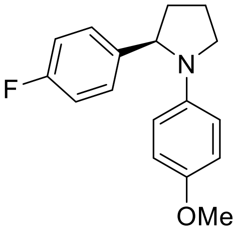	New	/	/	/	/
**2f**	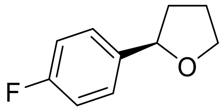	Known	/	/	-	-
**2g**	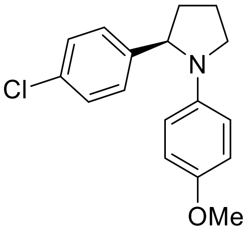	New	/	/	/	/
**2h**	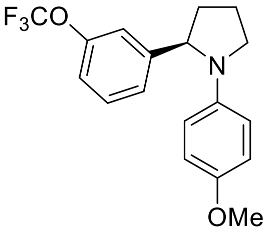	New	/	/	/	/
**2i**	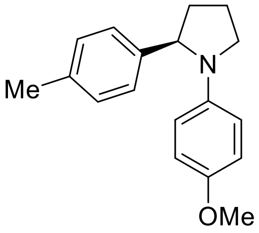	New	/	/	/	/
**2j**	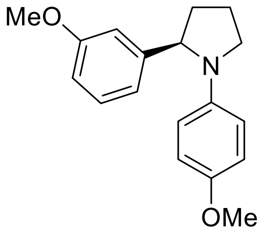	New	/	/	/	/
**2k**	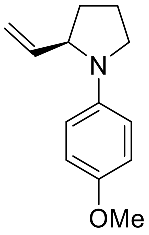	New	/	/	/	/
**2l**	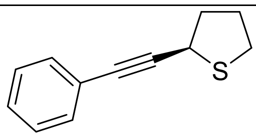	Known	/	/	-	-

**2m**	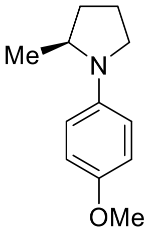	Known	/	/	-	-
**2n**	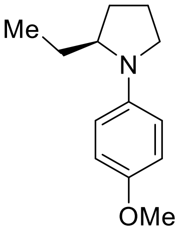	New	/	/	/	/
**2o**	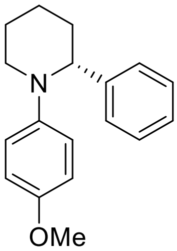	New	/	/	/	/
**2p**	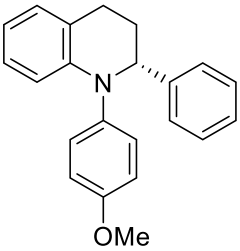	Known	/	/	/	/
**2q**	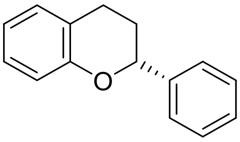	Known	/	/	-	-
**2r**	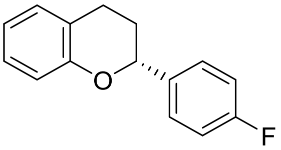	Known	/	/	-	-
**2d′**	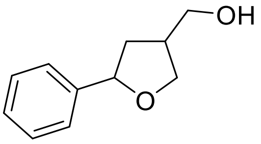	Known	/	/	/	/
**2d″**	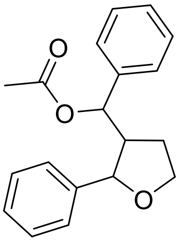	New	/	/	/	/

**2s**	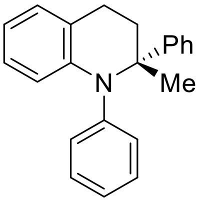	New	/	/	/	/
**2t**	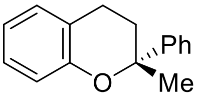	New	/	/	/	/
**2u**	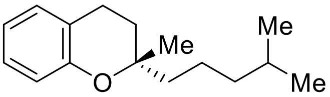	New	/	/	/	/
**2v**	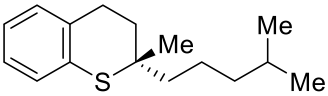	New	/	/	/	/
**2u′**	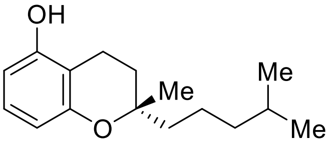	New	/	/	/	/

c. *Checklist of characterization data of synthesized intermediates*

Code	Compound	New/ Known	^{1}H-NMR	^{13}C-NMR	IR	HRMS
**A1**	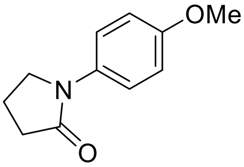	Known	/	/	-	-
**K1**	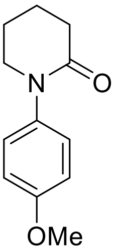	Known	/	/	-	-
**K2**	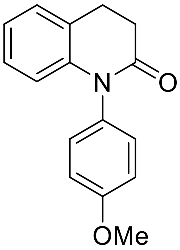	Known	/	/	-	-
**M1**	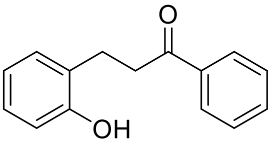	Known	/	/	-	-

**M2**	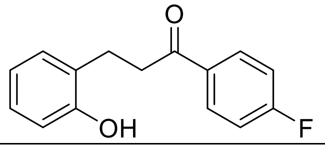	Known	/	/	-	-
**O**	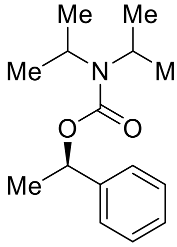	Known	/	/	-	-
**P**	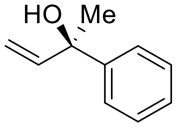	Known	/	/	-	-
**Q**	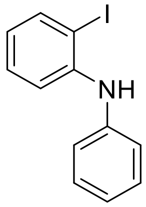	Known	/	/	-	-
**CC**	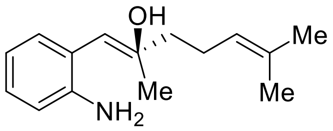	Known	/	/	-	-
**DD**	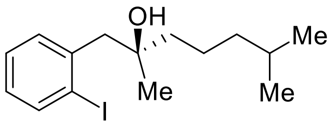	New	/	/	/	/
**EE**	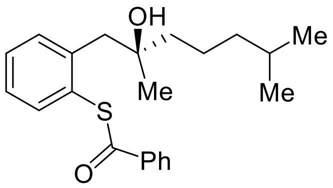	New	/	/	/	/
**FF**	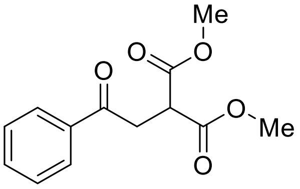	Known	/	/	-	-
**GG**	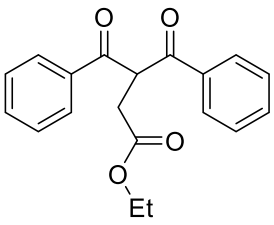	Known	/	/	-	-
**3**	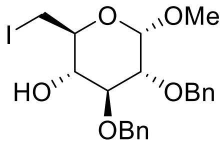	Known	/	/	/	/

**4**	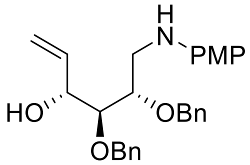	Unknown	/	/	/	/
**5**	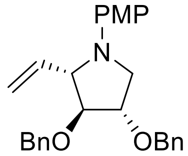	Unknown	/	/	/	/
**6**	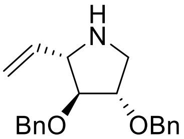	Known	/	/	/	/
**7**	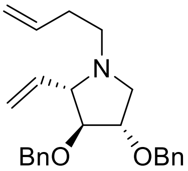	Known	/	/	/	/
**7a**	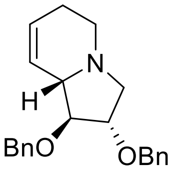	Known	/	/	/	/
**8** (+)-Lentiginosine	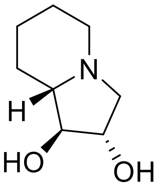	Known	/	/	/	/

Supplementary Table 2: Optimization of reaction conditions for secondary benzylic alcohols*

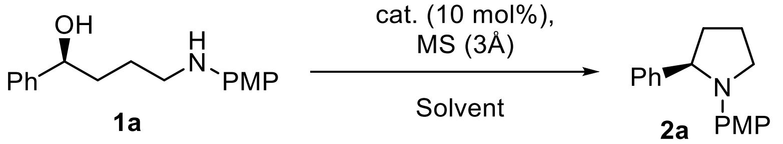

Entry	Catalysts	Solvent	Temp (°C)	Yield (%)†	e.s. (%)‡
1	FeF_3(III)	DCE	90	15	0
2	$FeCl_2$(II)	DCE	90	20	92
3	$Fe(NO_3)_3\cdot(H_2O)$	DCE	90	NR	0
4	$Fe(acac)_3$	DCE	90	NR	0
5	$Fe_4[Fe(CN)_6]_3$	DCE	90	NR	0
6	$Fe(ClO_4)_2\cdot 4H_2O$	DCE	90	10	91.1
7	Fe(EDTA)sodium salt	DCE	90	NR	0
8	Fe_2O_3	DCE	90	10	93
9	$FeCl_3$	DCE	90	35	92
10	Ferric citrate	DCE	90	NR	0
11	Iron(III) tartrate	DCE	90	NR	0
12	$Fe(OTf)_3$	DCE	90	62	96
13	$Fe(OTf)_3$	DCE	110	85	80
14	$Fe(OTf)_3$	ACN	90	13	0
15	$Fe(OTf)_3$	$MeNO_2$	90	10	92
16	$Fe(OTf)_3$	1, 2 dibro-momethane	90	05	0
17	$Fe(OTf)_3$	$CDCl_3$	90	14	95
18	$Fe(OTf)_3$	Toluene	90	32	0
19	$Fe(OTf)_3$	1,4 dioxane	90	23	90
20	$Fe(OTf)_3$	Hexane	90	21	99
21	$Fe(OTf)_3$ + $AgBF_4$(10 mol %)	DCE	90	NR	0
22	$Fe(OTf)_3$ + $AgSbF_6$(10 mol %)	DCE	90	NR	0
23	$Fe(OTf)_3$ + $AgPF_6$ (10 mol %)	DCE	90	NR	0
24	$Fe(OTf)_3$§ + MS (3Å)	DCE	90	98	99
25	MS (3Å)	DCE	90	NR	0
26	$Fe(OTf)_3$ + CF_3SO_3H (5 mol%)	DCE	90	31	88
27	CF_3SO_3H (10 mol%)	DCE	90	NR	0
28	Without catalyst	DCE	90	NR	0
29	$Cu(OTf)_2$	DCE	90	<10	0
30	$Ni(OTf)_2$	DCE	90	<10	0
31	$Mn(OTf)_2$	DCE	90	<10	0
32	$Co(OTf)_2$	DCE	90	<10	0

Reaction condition: *All reactions were performed using 0.5 mmol of **1a**, 0.050 mmol of catalyst (10 mol %) in DCE as solvent (2.0 mL), MS (3Å) = 300 mg, at 90 °C temperature for time 24 h under argon atmosphere. †NMR yield. ‡Enantiospecificity was determined by chiral stationary phase HPLC analysis.

§The purity of catalyst has been determined by Inductively Coupled Plasma-Mass Spectroscopy (ICP-MS) analysis. NR, no reaction.

Supplementary Table 3: Optimization of reaction conditions for tertiary alcohols*

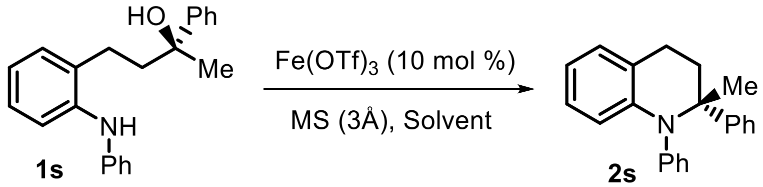

Entry	Solvent (mL)	Temp (°C)	Time (h)	Yield (%)†	e.s. (%)‡
1	DCE	90	24	100	09
2	*n*-Hexane	90	24	100	26.8
3	DCE + *n*-Hexane (0.5+0.5)	90	24	100	40
4	DCE + *n*-Hexane (0.4+0.6)	90	24	100	32.2
5	DCE + *n*-Hexane (0.3+0.7)	90	24	100	32.2
6	DCE + *n*-Hexane (0.2+0.8)	90	24	100	29
7	DCE + *n*-Hexane (0.25+0.25)	90	24	100	36
8	DCE + *n*-Hexane (01+01)	90	24	100	35
9	DCE + *n*-Hexane (0.5+0.5)	80	24	100	50.53
10	DCE + *n*-Hexane (0.5+0.5)	60	24	100	64
11§	DCE + *n*-Hexane (0.5+0.5)	rt	48	98	96

Reaction condition: *All reactions were performed using 0.2 mmol of **1s**, MS (3Å) = 100 mg, and 0.020 mmol of catalyst (10 mol %) in the indicated solvent (01 mL) under argon atmosphere. †NMR yield. ‡Enantiomeric excess was determined by chiral stationary phase HPLC analysis. §The purity of catalyst has been determined by Inductively Coupled Plasma-Mass Spectroscopy (ICP-MS) analysis.

Inductively Coupled Plasma Mass Spectrometry analysis of $Fe(OTf)_3$ catalyst: Inductively Coupled Plasma Mass Spectrometry (ICP-MS) was used for detecting trace elemental impurities in the $Fe(OTf)_3$ catalyst (purity 90.00%, Supplementary Table 2). The major metal impurities were individually screened as catalysts for the transformation (Supplementary Table 2, entry 29-32). However, none of the trace metals outperformed $Fe(OTf)_3$ as catalyst in the intramolecular substitution reaction.

Supplementary Figure 1: Rate order determination

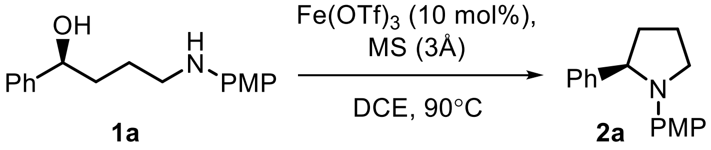

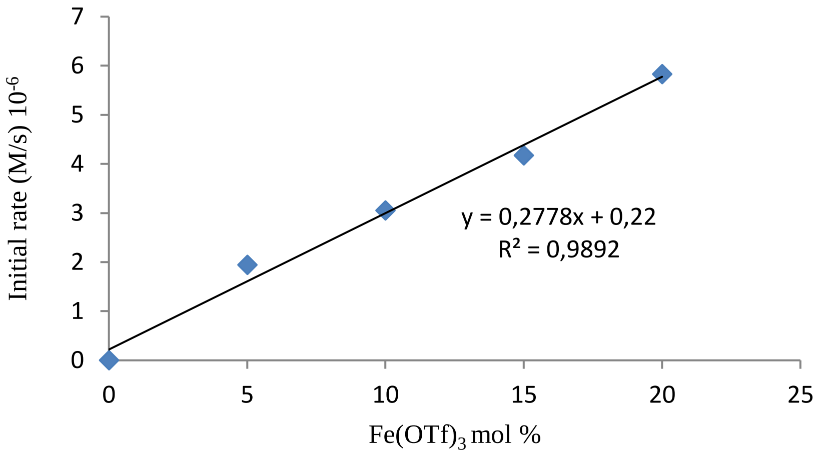

Supplementary Figure 1: 1a (0.2 mmol), DCE (1 mL), MS (3Å) (200 mg), and catalyst (0, 5, 10, 15, and 20 mol%) were heated in an oil bath at 90° C. Initial rates of the reaction were determined below 20% conversion (up to 2h) by ^{1}H NMR spectroscopy. The values are the mean value of two reactions.

The reaction of **1a** to **2a** was performed using five different concentrations of catalyst (0, 5, 10, 15, and 20 mol%). The reactions were monitored by using ^{1}H NMR spectroscopy and the initial rates were determined below 20% conversion. Duplicates of the reactions were made and the data is the mean value of these duplicates (Suplementary Figure I).

Supplementary Figure 2: Comparative studies of kinetic dependance of reaction with respect to nucleofuge and nucleophile in secondary alcohols

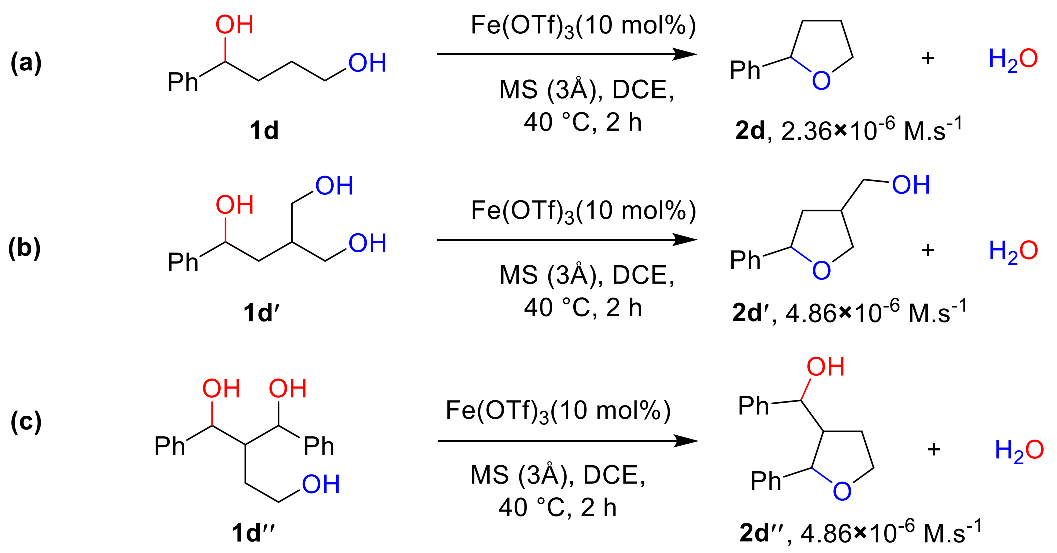

Supplementary Figure 2: Comparative rate-order determination of reaction with respect to nucleophile and nucleofuge.

To an oven-dried 5 ml vial equipped with a magnetic stir bar was added substrate **1d**, **1d’** or **1d’’** (0.5 mmol), MS (3Å) (300 mg), and $Fe(OTf)_3$ (25.05 mg, 0.05 mmol). The tube was sealed with a teflon-lined cap, connected to a vacuum and backfilled with argon three times by piercing with a needle attached to a Schlenk line. Then 2.0 ml of anhydrous DCE was added by syringe and the mixture was stirred at 40 °C. Initial rates were determined below 20% conversion (up to 2 h) by ^{1}H NMR spectroscopy. The values are the mean value of two runs.

Supplementary Figure 3: Comparative studies of kinetic dependance of reaction with respect to nucleofuge and nucleophile in tertiary alcohols:

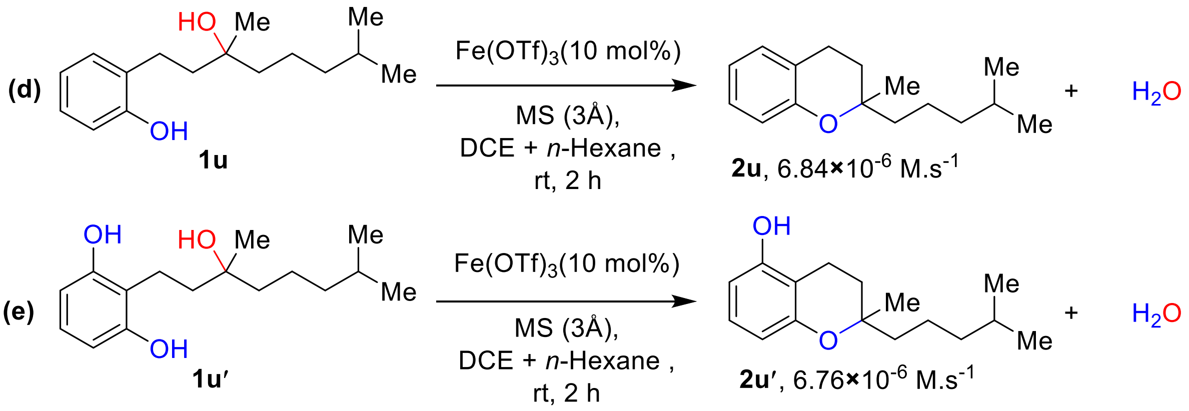

Supplementary Figure 3: Comparative rate-order determination of reaction with respect to nucleophile.

To an oven-dried 5 ml vial equipped with a magnetic stir bar was added substrate **1u** or **1u'** (0.5 mmol), MS (3Å) (300 mg), and $Fe(OTf)_3$ (25.05 mg, 0.05 mmol). The tube was sealed with a teflon-lined cap, connected to a vacuum and backfilled with argon three times by piercing with a needle attached to a Schlenk line. Then 2.0 ml of anhydrous DCE was added by syringe and the mixture was stirred at room temperature. Initial rates were determined below 20% conversion (up to 2 h) by ^{1}H NMR spectroscopy. The values are the mean value of two runs.

Supplementary Figure 4: Deutrium kinetic isotope effect study

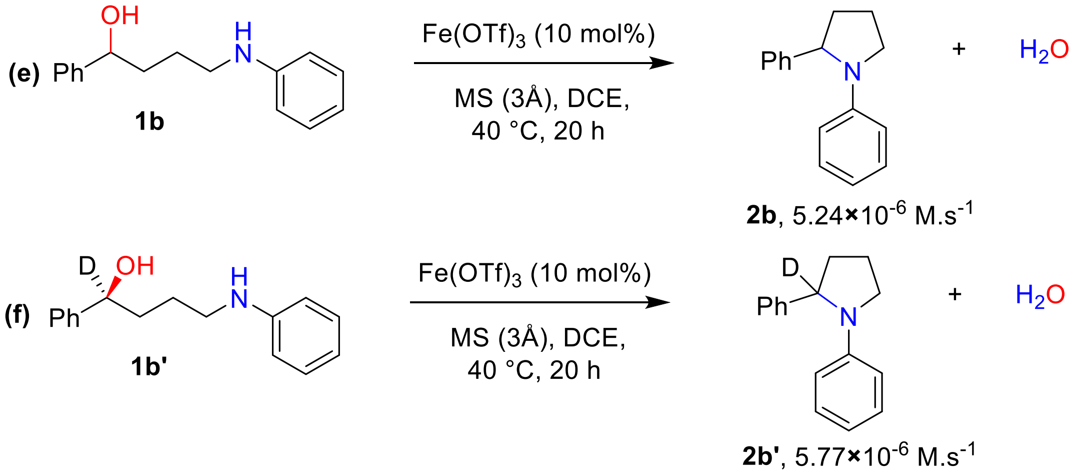

Supplementary Figure 4: Kinetic isotope effect study of compounds **1b** and **1b'**.

For kinetic isotope effect (KIE) study, deutrieted analogue of **1b** was prepared by $NaBH_4$ reduction and rate constants for **1b** and **1b'** were measured under similar conditions. The deuterium incorporation of 85% was found on **1b'**. Thereafter, formation of product was calculated by comparing the integration of signals at 3.45, 3.56, and 2.94 ppm. A KIE (kH/kD) of 0.91 was found when rates of formation of **2b** and **2b'** were compared.

Supplementary Note 1

Experimental details: Unless otherwise noted, all reactions were carried out in oven-dried 5 ml vial. All the reagents and solvents were bought from commercial sources and were used without further purification. All reactions were executed with oven-dried glassware under inert condition using argon for protection. 1, 2-dichloroethane (DCE) was distilled using CaH_2. Dry THF, diethyl ether and toluene were obtained from a VAC solvent purifier. NMR spectra were recorded with a 400 MHz (^{1}H) and 100 MHz (^{13}C) spectrometer as solutions in $CDCl_3$. Chemical shifts (δ) are reported in parts per million (ppm) and are referenced to $CDCl_3$ (δ = 7.26 ppm) as an internal standard. All coupling constants (*J*) are expressed in Hz. The description of the signals include: s = singlet, d = doublet, t = triplet, m = multiplet and dd = doublet of doublets, at = apparent triplet. IR spectra were recorded by a Perkin Elmer FT-IR Spectrometer. High-Resolution Mass Spectra (HRMS) were performed with a micrOTOF (Bruker) spectrometer by Na-formate. The molecular fragments are quoted as the relation between mass and charge (*m/z*). The enantiospecificity (e.s.) of products were determined by chiral HPLC using the corresponding racemic compounds as references. The routine monitoring of reactions was performed by crude ^{1}H NMR.

Abbreviations

Cb	*N,N'*-diisopripylcarbamoyl
Me	Methyl
n-Hex	*n*-hexyl
PE	Petroleum ether 40/60 fraction
Ph	Phenyl
*s*Bu	*sec*-butyl
THF	Tetrahydrofuran
DMF	N,N-Dimethylformamide
DCE	Dichloroethane
DPPF	1,1′-Ferrocenediyl-bis(diphenylphosphine)

Supplementary methods

Experimental procedures

Synthesis of 1a, 1b, 1e, 1g, 1h, 1i, and 1j:

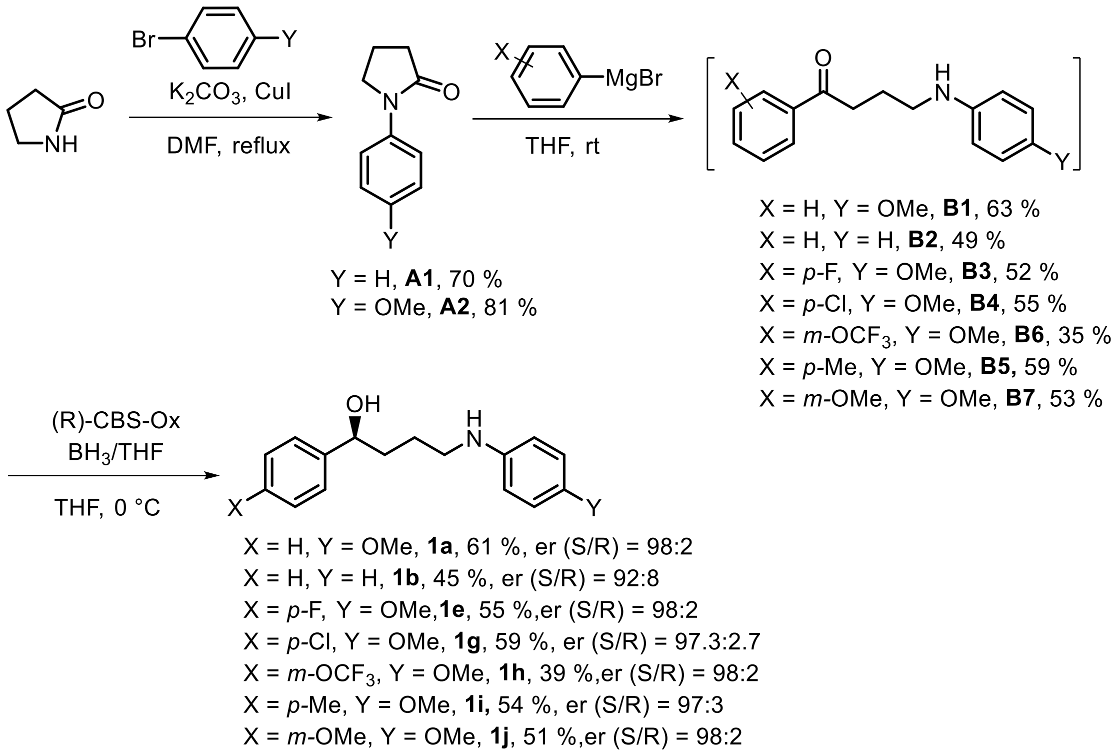

To a solution of 2-pyrrolidinone (30 mmol) in 30 mL dry DMF was added CuI (10 mol%), anhydrous K_2CO_3 (1.1 equiv.) and aryl bromide (2 equiv.). The reaction mixture was refluxed for 48 h. After completion of the reaction, the reaction mixture was allowed to attain room temperature. Aqueous saturated NH_4Cl (50 ml) was then added and the aqueous layer was separated and extracted with EtOAc (4 × 50 ml). The combined organic phase were washed with brine (1×50 mL), dried over anhydrous $MgSO_4$ and concentrated under reduced pressure to give the crude product. Purification is carried out by silica gel column chromatography to afford *N*-aryl-2-pyrrolidinones **A**.

An oven-dried round-bottomed flask equipped with a magnetic stir bar was charged with dry THF (20 mL) and *N*-aryl-2-pyrrolidinones **A** (10 mmol) under argon atmosphere. The solution was cooled to 0 °C and aryl magnesium bromide (1.1 equiv, in 4 mL THF) was added dropwise.

The reaction was allowed to attain room temperature and was run at the same temperature for 3 h. The reaction was quenched with saturated NH_4Cl solution (30 mL) extracted into diethyl ether (3×50 mL). The combined organics were dried over sodium sulfate, filtered, and concentrated under reduced pressure. The crude residues (ketones, **B1**-**B7**) were directly used for the next step (*i.e.* CBS-reduction) without further purification.

Ketones (**B1**-**B7**) were reduced to the alcohols enantioselectively by Corey-Bakshi-Shibata (CBS) reduction method. An oven-dried round-bottomed flask equipped with a magnetic stir bar was charged with BH_3/THF complex (1.2 equiv.) and chiral oxazaborolidine catalyst (R-CBS-Ox, 10 mol%) under argon. The solution was cooled to 0 °C and stirred for 15 min. Ketones **B1–B7** (5 mmol) dissolved in dry THF (10 mL) were added dropwise and the reaction was continued for 2h at same temperature. After completion of reaction (TLC), the reaction was quenched with saturated NH_4Cl solution (30 mL), extracted into ethyl acetate (3×50 mL). The combined organics were dried over sodium sulfate, filtered, and concentrated in vacuo. The residue was purified by silica gel column chromatography to afford the alcohols **1a**-**1b**, **1e**, and **1g**-**1j**.[1]

Synthesis of 1d and 1f:

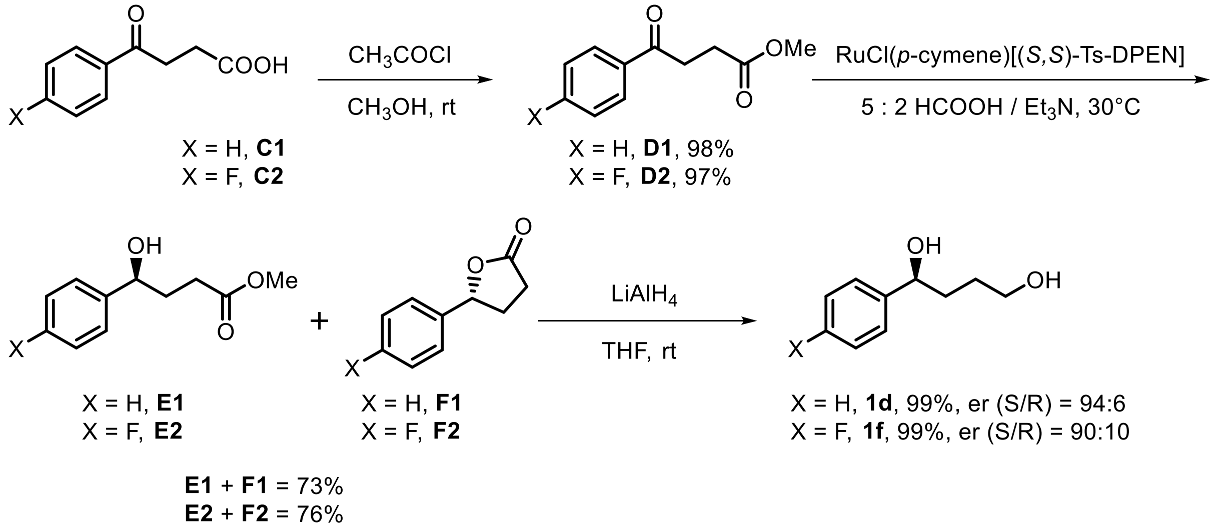

4-Oxo-4-arylbutyric acid **C** (10 mmol) was dissolved in methanol (10 mL). Acetyl chloride (1.2 equiv.) was added dropwise and the reaction mixture was stirred at room temperature for overnight. After completion of the reaction (TLC), the reaction mixture was extracted into DCM (3 × 50 mL). The combined organic parts were washed with water (2×50 mL) and brine (1×50 mL); dried over anhydrous Na_2SO_4 and concentrated under reduced pressure to obtain the corresponding methyl esters **D**. Esters **D** were used in the next step without further purification.

Ester **D** (5 mmol) dissolved in dry THF (10 mL) were added dropwise to a solution of RuCl(*p*-cymene)[(*S*,*S*)-Ts-DPEN (5 mol%) in 5 : 2 formic acid / triethylamine (10 mL) under argon and stirred for 48 h at 30 °C oil bath. After completion of the reaction, the reaction was quenched with saturated $NaHCO_3$ solution (30 mL) and extracted into DCM (3×50 mL). The combined organic layers were washed with water (2×50 mL) and brine (1×50 mL); dried on anhydrous Na_2SO_4 and concentrated under reduced pressure. The residue was purified by column chromatography to obtain a non-separable mixture of alcohol (**E**) and lactone (**F**).

A mixture of **E** and **F** (approx. 5 mmol) were reduced by using $LiAlH_4$ (0.5 equiv.) in dry THF (20 mL) at room temperature to obtain the products **1d** and **1f** in quantitative yields.[2,3]

Synthesis of 1c:

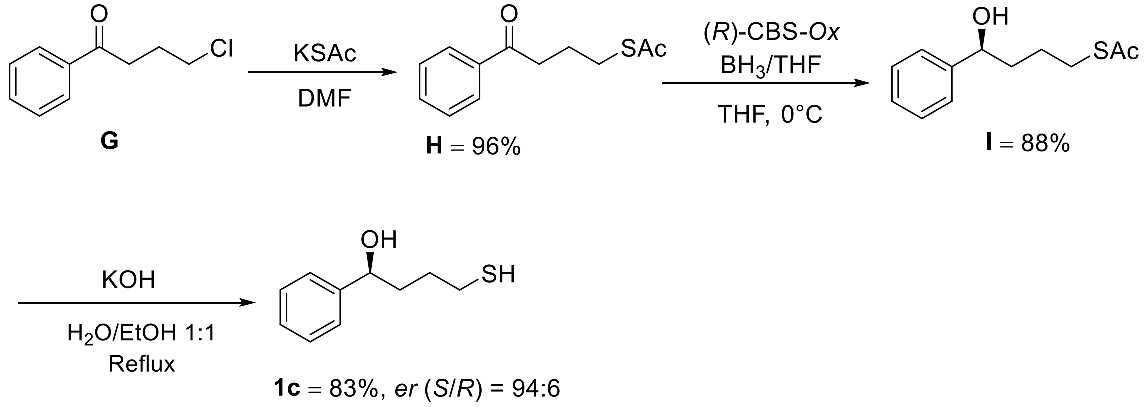

To a solution of KSAc (1.5 equiv.) in DMF (25 ml) was added 4-chloro-1-arylbutan-1-one **G** (10 mmol) dropwise at 0 °C. The reaction was run at rt for 10 h. After completion of the reaction (TLC), the solvent was evaporated; the residue was extracted with EtOAc (3×50 mL) and washed with water (2×50 mL) and brine (1×50 mL). The combined organic phase was dried over $MgSO_4$ and the solvent was removed in reduced pressure to obtain pure **H** in analytically pure forms which were used directly for the next synthetic steps without further purifications.

To a 1 M solution of BH_3 in THF (0.5 equiv.) at 0°C was added 1 M solution of (*R*)-(+)-2-methyl-CBS-oxazaborolidine catalyst in toluene (10 mol%) drop wise. Compound **H** (8 mmol) was dissolved in THF (12 mL) and added dropwise. The reaction was completed after 3 h stirring at the same temperature (TLC). A saturated solution of NH_4Cl (25 mL) was added and solution was stirred for 15 min at rt. The reaction mixture was extracted with EtOAc (3×50 mL), washed with brine, dried over $MgSO_4$. The combined organic layer was concentrated under reduced pressure and purified by silica gel (100-200 mess) column chromatography to obtain **I**.

A solution of KOH (5 equiv.) in 1:1 EtOH/H_2O (15 mL) was added to **I** (4 mmol) and the reaction mixture was refluxed for 2 h. After completion of the reaction (TLC), the reaction mixture was allowed to attain room temperature; saturated NH_4Cl solution (20 mL) was added to it and extracted three times with EtOAc (3×50 mL). The combined organic phases were dried

over $MgSO_4$ and the solvent was removed by rotary evaporation. The crude product was purified by silica gel (100-200 mess) column chromatography to obtain **1c**.[4]

Synthesis of 1l:

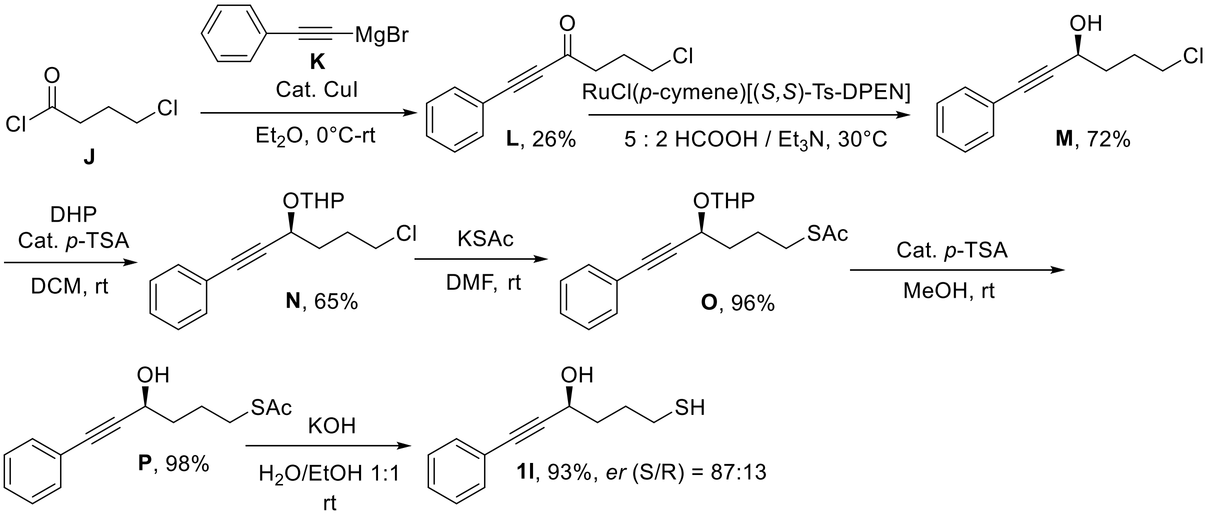

To a stirred solution of CuI (10 mol%) in 30 mL dry Et_2O, was added 4-chlorobutyryl chloride **J** (30 mmol) at 0°C and stirred for 15 mint. Phenylethynylmagnesium bromide solution **K** (1 equiv.) was added very slowly over the period of 1 h and the reaction mixture was stirred for another 1 h and then allowed to attain room temperature. Saturated NH_4Cl solution (50 mL) was added and extracted by Et_2O (3×50 mL). The combined organic layers were washed with water (1×50 mL) and Brine (1×50 mL) and concentrated under reduced pressure. The residue was purified by silica gel column chromatography to obtain **L** in 26% yield.

Ketone **L** (4.8 mmol) was added to a solution of RuCl(*p*-cymene)[(*S*,*S*)-Ts-DPEN (1 mol%) in 5 : 2 formic acid / triethylamine (10 mL) under argon and stirred for 12 h at rt. After completion of the reaction, saturated $NaHCO_3$ solution (20 mL) was added and the reaction mixture was extracted by DCM (3×50 mL). The combined organic layers were washed with water (2×50 mL) and Brine (1×50 mL); dried on anhydrous Na_2SO_4 and concentrated under reduced pressure. The residue was purified by column chromatography to obtain alcohol **M** in 72% yield.

To a solution of alcohol **M** (2.4 mmol.) in DCM (10 ml) was added *p*-TSA (10 mol%) and dihydropyran (1.2 equiv.) dropwise at rt. The reaction was run at rt for 12 h. After completion of the reaction (TLC), the reaction mixture was worked-up with water (50 mL) and extracted

by DCM (3×50 mL). The combined organic layers were washed with Brine (1×50 mL); dried on anhydrous Na_2SO_4 and concentrated under reduced pressure. The residue was purified by column chromatography to obtain ether **N** in 65% yield.

To a solution of ether **N** (4.5 mmol) in DMF (10 ml) was added KSAc (1.2 equiv.) at 0 °C. The reaction was warmed up and run at rt for 10 h. After completion of the reaction (TLC), the reaction mixture was worked up with water (50 mL) and extracted by DCM (3×50 mL). The combined organic layers were washed with water (2x50 mL) and Brine (1×50 mL); dried on anhydrous Na_2SO_4 and concentrated under reduced pressure. The residue was purified by column chromatography to obtain a thioacetate **O** in 96% yield.

To a solution of thioacetate **O** (1.8 mmol) in MeOH (5 ml) was added *p*-TSA (10 mol%) at rt. The reaction was run at rt for 4 h. After completion of the reaction (TLC), the reaction mixture was worked up with water (50 mL) and extracted by DCM (3×50 mL). The combined organic layers were washed with Brine (1×50 mL); dried on anhydrous Na_2SO_4 and concentrated under reduced pressure to obtain pure **P** (98% yield) in analytically pure forms which were used directly for the next synthetic steps without further purifications.

A solution of KOH (5 equiv.) in 1:1 EtOH/H_2O (5 mL) was added to **P** (2.2 mmol) and the reaction mixture was stirred at rt for 4 h. After completion of the reaction (TLC), the reaction mixture was worked up with water (50 mL) and extracted by DCM (3×50 mL). The combined organic layers were washed with Brine (1×50 mL); dried on anhydrous Na_2SO_4 and concentrated under reduced pressure. The residue was purified by column chromatography to obtain a thioacetate **1l** in 93% yield.[5]

Synthesis of 1k, 1m, and 1n:

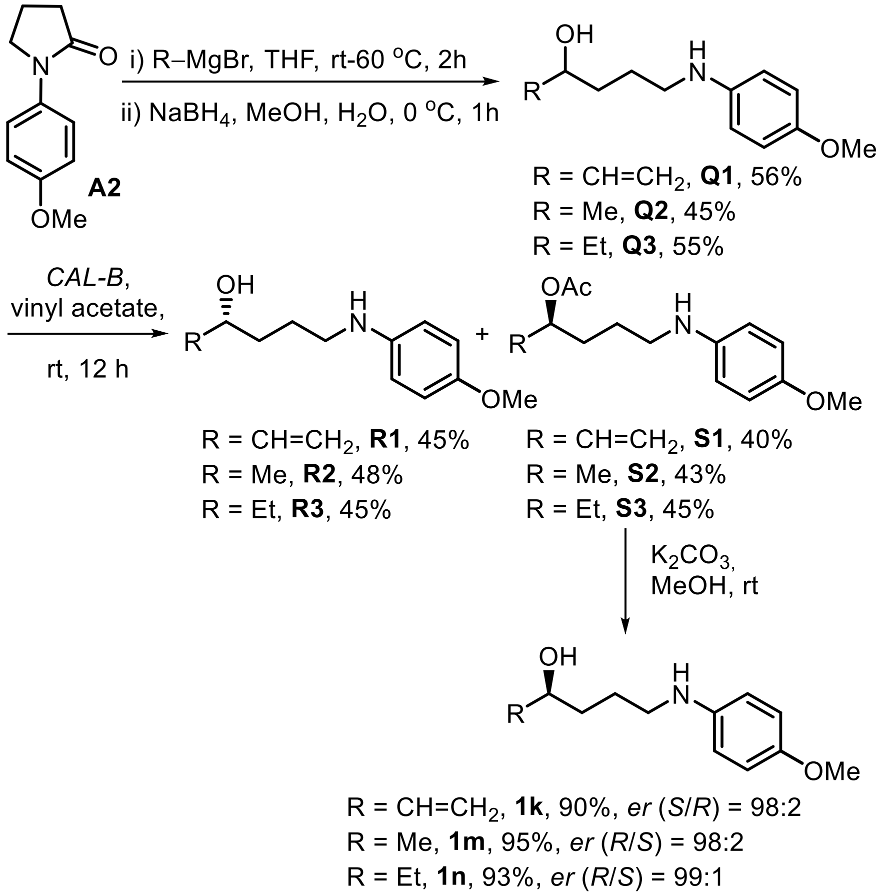

Alcohols **1k, 1m** and **1n** were prepared by ring opening of lactam **A2** with Grignard reagent. After completion by TLC, the following ketones were *in situ* reduced by $NaBH_4$ for 1 hour to obtain racemate alcohols. Crude reaction mixtures were purified by silica gel column chromatography to obtain **Q1**, **Q2**, and **Q3** in 56%, 45% and 55% yields.

Alcohols **Q1**, **Q2**, and **Q3** were used to perform kinetic resolution with *Candida Antarctica lipase-B* (*CAL-B*) in the excess amount of vinyl acetate for 12 hours. After completion of the reaction, crude mixtures were purified by silica gel column chromatography to alcohols **R1**, **R2**, **R3** in 45%, 48% and 45% yields and acetylated products **S1**, **S2**, **S3** in 40%, 43%, 45% yields, respectively.

Acetylated compounds **S1**, **S2**, **S3** were used to perform deprotection in the present of K_2CO_3 in MeOH for 2 hours. After completion by TLC, crude mixtures were purified by silica gel column chromatography to alcohols **1k**, **1m**, and **1n** in 90%, 95% and 93% yields, respectively.

Synthesis of 1o and 1p:

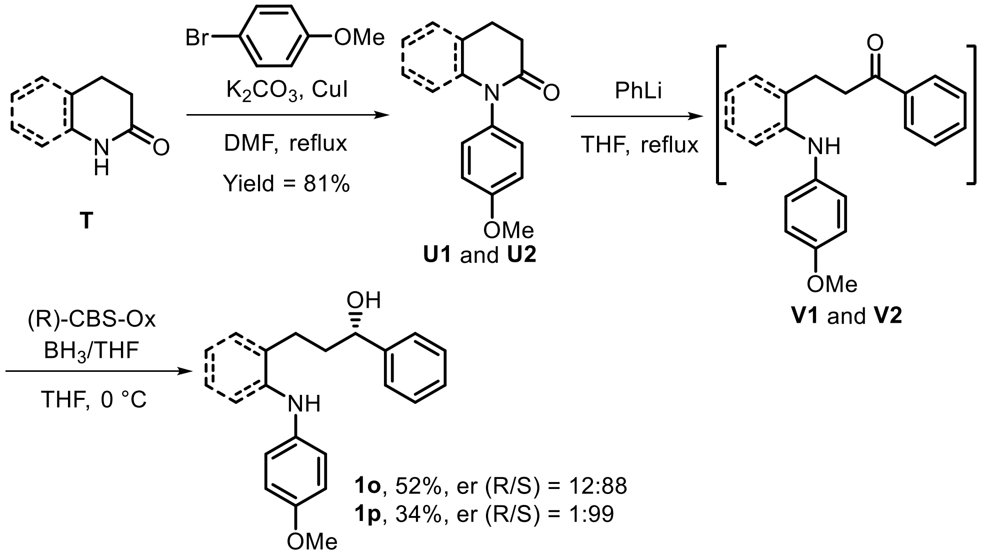

N-aryl lactam **U1** and **U2** were prepared following a similar procedure as described in general method **5**. To a solution of γ-lactam **T** (30 mmol) in 30 mL dry DMF was added CuI (10 mol%), anhydrous K_2CO_3 (1.1 equiv.), and aryl bromide (2 equiv.). The reaction mixture was refluxed for 48 h. After completion of the reaction, the reaction mixture was allowed to attain room temperature. Aqueous saturated NH_4Cl (50 ml) was then added and the aqueous layer was separated and extracted with ethyl acetate (4 × 50 ml). The combined organic phase were washed with brine (1×50 mL), dried over anhydrous Na_2SO_4 and concentrated under reduced pressure to give the crude product. Purification is carried out by usual silica gel column chromatography to afford pure **U1** and **U2**.

A warm solution of N-aryl lactam **U** (10 mmol) in dry benzene (30 ml) was added slowly to a well stir solution of phenyl lithium (10 mmol) under argon atmosphere. The reaction mixture was stirred at reflux for 2 h under argon atmosphere. Benzene and ice-water were added at ice temperature. The combined organic phase was separated, washed with water, dried over sodium sulfate and concentrated under reduced pressure. The crude residues (ketones, **V1**–**V2**) were directly used without further purification for the CBS-reduction, after which the crude reaction mixtures were purified by silica gel column chromatography to obtain pure alcohols **1o** and **1p**.[6,7]

Synthesis of 1q and 1r:

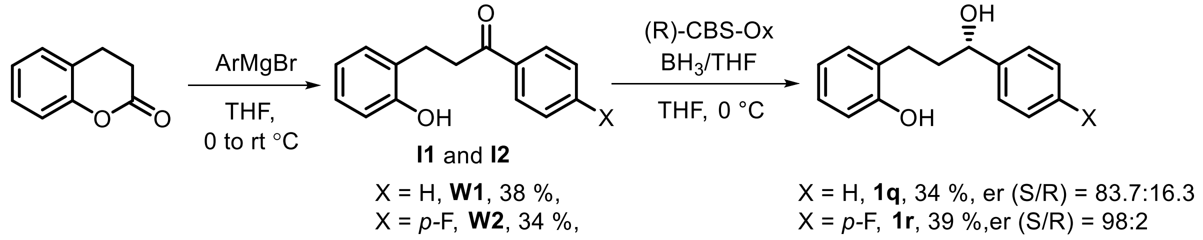

An oven-dried round-bottomed flask equipped with a magnetic stir bar was charged with dry THF (20 mL) and chroman-2-one (10 mmol) under argon atmosphere. The solution was cooled to 0 °C and aryl magnesium bromide (1.1 equiv, in 4 mL THF) was added dropwise. The reaction was allowed to attain room temperature and was run at the same temperature for 3 h. The reaction was quenched with saturated NH_4Cl solution (30 mL) extracted into diethyl ether (3×50 mL). The combined organics were dried over sodium sulfate, filtered, and concentrated under reduced pressure. The crude (ketones, **W1** and **W2**) was directly used without further purification for the CBS-reduction after which the crude reaction mixtures were purified by silica gel column chromatography to obtain pure **1q** and **1r** in 34% and 39% overall yields respectively.

Synthesis of enantiomerically enriched tertiary alcohols 1s, 1t, 1u and 1u':

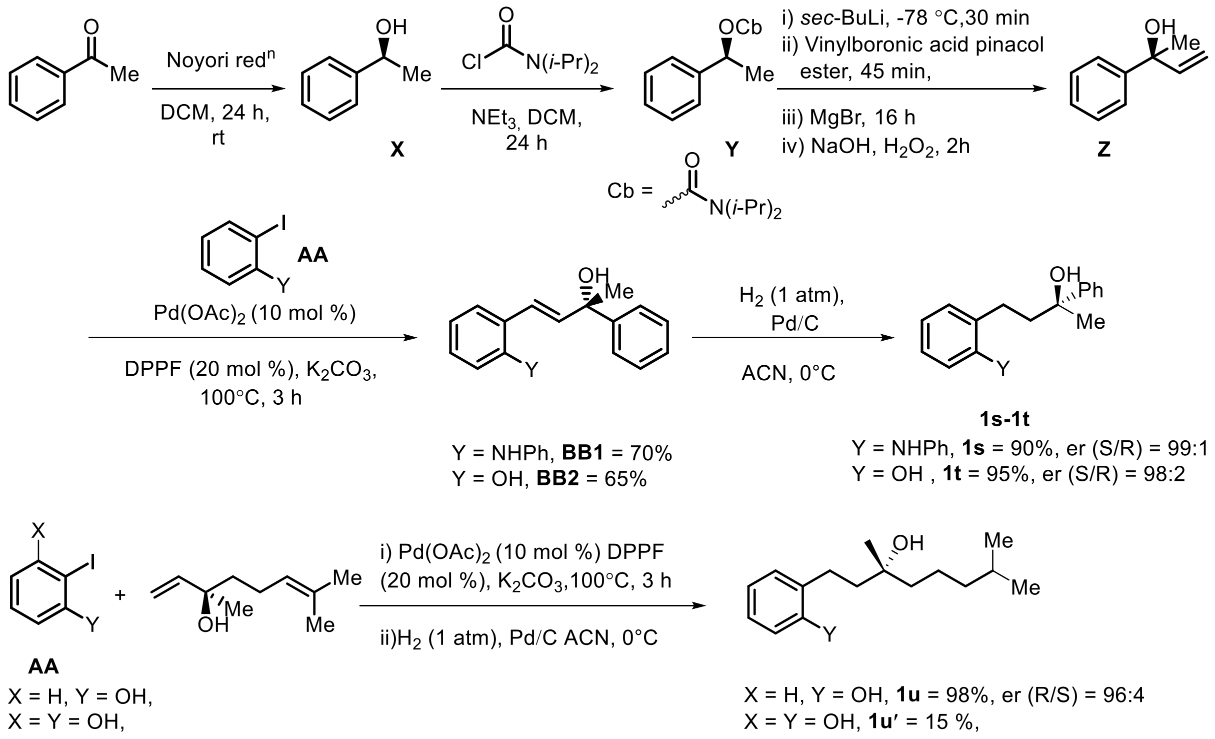

Preparation of enantiomerically enriched secondary benzylic alcohols **X** *via Noyori's asymmetric reduction:* Acetophenone (1.0 g, 8.33 mmol) was added to a solution of RuCl(*p*-cymene)[(*S*,*S*)-Ts-DPEN (52.9 mg, 0.083 mmol, 1.0 mol %) in 5:2 formic acid / triethylamine (15 mL) under argon and stirred at 28 °C for 24 h. After completion of the reaction, saturated $NaHCO_3$ solution (50 mL) was added and stirred for another 15 min. The reaction mixture was extracted into DCM (3×50 mL). The combined organic layers were washed with water (2×50 mL) and brine (1×50 mL); dried over anhydrous $MgSO_4$ and concentrated under reduced pressure. Purification of the crude residue by column chromatography afforded pure alcohol **X** in 90 % yield.

The following procedure is representative of the preparation of secondary carbamates **Y** *from chiral secondary benzylic alcohol*: A solution of alcohol **X** (5 mmol), diisopropylcarbamoyl chloride (1.1 equiv.), and triethyl amine (1.1 equiv.) in anhydrous DCM (30 mL) was refluxed for 24 h. After completion of the reaction (TLC), the reaction mixture was poured in water (50 mL). The mixture was extracted with diethyl ether (3 × 50 mL). The combined organic parts

were washed with water (2×50 mL) and brine (1×50 mL); dried over anhydrous Na_2SO_4 and concentrated under reduced pressure. The crude product was purified by usual silica gel column chromatography to afford pure carbamate **Y**.

Lithiation/borylation of chiral secondary carbamates to tertiary allylic alcohol **Z:**

To a stirred solution of (*S*)-1-Phenylethyl diisopropylcarbamate **Y** (1 g, 4.01 mmol) in 20 mL anhydrous diethyl ether at –78 °C was added *s*-BuLi (3.4 mL of 1.4 M solution, 4.8 mmol, 1.2 equiv.) drop wise under an atmosphere of argon. The resulting light yellow homogeneous solution was stirred at –78 °C for 30 min and neat vinylboronic acid pinacol ester (1 mL, 6 mmol, 1.5 equiv.) was added drop wise with vigorous stirring. The reaction mixture was then stirred for 45 minutes at –78 °C. A methanol solution of magnesium bromide (6.0 mL, 6.0 mmol; 1M) was added dropwise under argon. The reaction mixture was stirred at –78 °C for an additional 15 min. and then allowed to attain room temperature and was run at the same temperature for 16 h. The reaction was quenched with the addition of an ice cold solution of 3 M aqueous sodium hydroxide (14.8 mL) and 30% aqueous H_2O_2 (8.5 mL) and stirred at room temperature for an additional 2 hours. The reaction mixture was extracted by Et_2O (3×50 mL). The combined organic layers were washed with water (1×50 mL) and brine (1×50 mL) and concentrated under reduced pressure. The crude product was purified by column chromatography to obtain tertiary allylic alcohol **Z** (474.7 mg, 80%) as a colorless oil.[8,9]

Palladium-catalyzed vinylations of iodoanilines **AA:**

A mixture of **AA** (1.0 mmol), 2-phenylbut-3-en-2-ol **Z** (5.0 mmol), $Pd(OAc)_2$ (0.10 mmol), and DPPF as ligand (0.20 mmol) in the presence of K_2CO_3 (1.5 equiv.) in toluene : H_2O (1:1, 2.0 mL) were heated with stirring in a sealed tube at the temperatures 100°C for 3 h. After completion of the reaction (TLC), saturated K_2CO_3 solution (30 mL) was added and the reaction mixture was extracted into ethyl acetate three times. The combined organic layers were washed

with saturated NaCl (1×50 mL); dried over anhydrous Na_2SO_4 and concentrated under reduced pressure. The residue was purified by column chromatography to obtain a pure alcohol **BB**.[10]

Hydrogenation of a tertiary alcohol **BB:**

Tertiary alcohol **BB** (1 mmol) was dissolved in ACN (10 mL) at room temperature. Pd/C (10 % wt) was added under argon atmosphere, and the reaction vessel was cooled to 0°C. Then, the atmosphere was substituted with H_2 (1 atm) and the reaction mixture was stirred at the same temperature for 1 h. After the completion of reaction (TLC), the mixture was filtered through a tight packed pad of Celite®. The filtrate was concentrated and purified *via* silica gel (100-200 mess) column chromatography to obtain pure **1s**, **1t**, **1u** and **1u'**.

Synthesis of 1v:

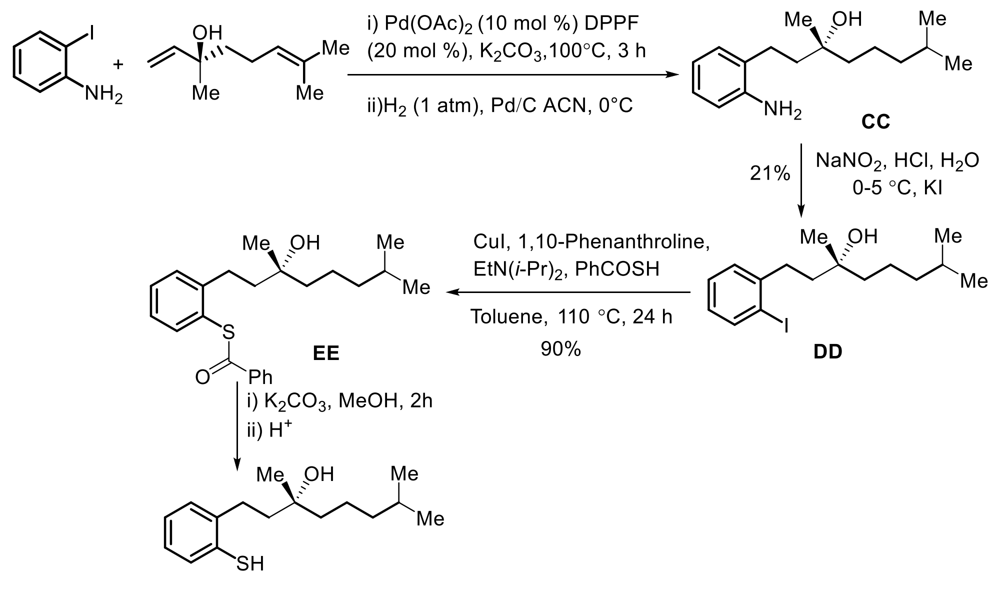

1v = 95%, er (S/R) = 96:4

Palladium-catalyzed vinylations of iodoanilines:

A mixture of *iodoanilines* (1.0 mmol), 2-phenylbut-3-en-2-ol **Z** (5.0 mmol), Pd(OAc)$_2$ (0.10 mmol), and DPPF as ligand (0.20 mmol) in the presence of K_2CO_3 (1.5 equiv.) in toluene : H_2O (1:1, 2.0 mL) were heated with stirring in a sealed tube at the temperatures 100°C for 3 h. After completion of the reaction (TLC), saturated K_2CO_3 solution (30 mL) was added and the reaction mixture was extracted into ethyl acetate three times. The combined organic layers were washed with saturated NaCl (1×50 mL); dried over anhydrous Na_2SO_4 and concentrated under reduced pressure. The residue was purified by column chromatography to obtain a pure tertiary alcohol.[10]

Hydrogenation of a tertiary alcohol:

Tertiary alcohol (1 mmol) was dissolved in ACN (10 mL) at room temperature. Pd/C (10 % wt) was added under argon atmosphere, and the reaction vessel was cooled to 0°C. Then, the atmosphere was substituted with H_2 (1 atm) and the reaction mixture was stirred at the same temperature for 1 h. After the completion of reaction (TLC), the mixture was filtered through a tight packed pad of Celite®. The filtrate was concentrated and purified *via* silica gel (100-200 mess) column chromatography to obtain pure **CC**.

Iodination through diazotization:

A mixture of tertiary alcohol **CC** (1 g, 4.2 mmol), aqueous HCl (37%, 0.9 mL) and water (3.2 mL) was cooled to 0°C. A solution of $NaNO_2$ (297 mg, 4.3 mmol) in water (3.2 mL) was added dropwise and stirred for 10 min. The resulting diazonium salt was treated with a solution of KI (714 mg, 4.3 mmol) in water (3.2 mL). The resulting brown foamy mixture was stirred for 30 min at room temperature and heated at reflux for 30 min. After cooling to room temperature, the reaction was diluted with water (10 mL) and neutralized by slow addition of aqueous $Na_2S_2O_3$. The mixture was extracted with dichloromethane (10 mL x 2). The combined organic layer was dried over $MgSO_4$, filtered and evaporated *in vacuo*. The residue was purified by silica gel column chromatographyto afford **DD** (351 mg, 24%).[11]

Copper-catalyzed coupling of aryl iodides and thiobenzoic acid:

To a solution of aryl iodide **DD** (1 mmol), thiobenzoic acid (1 mmol), 1,10 phenanthroline (20 mol %), and iPr_2NEt (2 mmol) in toluene (5 mL) was added CuI (10 mol %). The resulting mixture was degassed and then stirred under N_2 at 110 °C for 24 h. The reaction mixture was then cooled to rt and diluted to 50 mL with CH_3CN. The resulting mixture was purified by silica gel column chromatography to afford **EE** (90 %). The resulting pure S-phenyl thiobenzoate **EE** on further deprotection with K_2CO_3 in methanol as solvent furnished a desire product **(*S*)-1v**.[12]

Synthesis of 2-iodo-N-phenylaniline (AA)

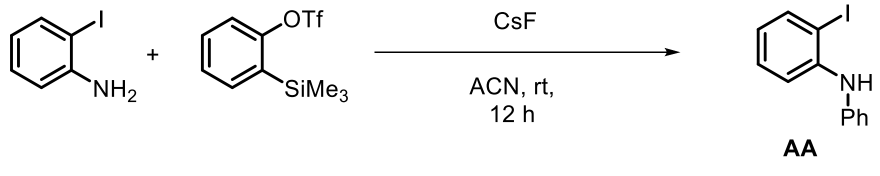

To a solution of 2-iodoaniline (2.0 mmol, 438.04 mg) and 2-(Trimethylsilyl)phenyl trifluoromethanesulfonate (2.2 mmol, 656.37 mg) in acetonitrile (20 mL) was added CsF (4.0 mmol, 607.6 mg). The reaction was allowed to stir at room temperature for 12 h. After completion of the reaction (TLC), H_2O (10 mL) was added carefully and stirred for 15 min.

The mixture was extracted with DCM (3×50 mL) and the combined organic layers were washed with water (1×50 mL) and brine (1×50 mL)); dried over anhydrous $MgSO_4$ and concentrated under reduced pressure. The residue was purified by column chromatography to obtain 2-iodo-N-phenylaniline (**AA**, 92.0%).[10]

Synthesis of dioxygen-centered nucleophiles 1d':

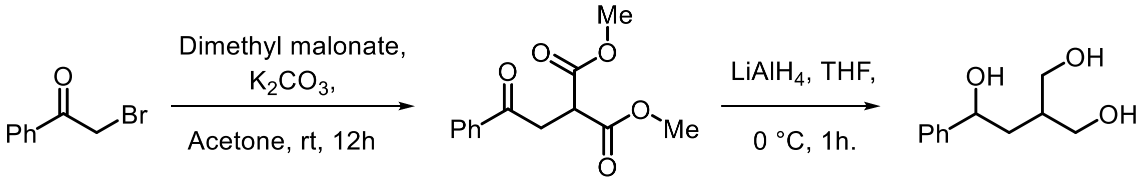

A mixture of 2-bromoacetophenone (10 mmol, 199 mg), anhydrous K_2CO_3 (1 equiv. 138mg, 10 mmol), and methyl ethyl ketone (2 equiv. 144mg) in acetone (20 mL) was stirred at room temperature for 12 h. After completion of the reaction it was diluted with water, extracted in DCM, washed with water, brine and dried over the anhydrous sodium sulphate. The solvent was removed under vacuum. The crude product was recrystallized from 2-propanol gave pure compound dimethyl 2-(2-oxo-2-phenylethyl)malonate **FF** (90 %, 225 mg). The compound **FF** (approx. 8 mmol) was reduced by using $LiAlH_4$ (0.5 equiv.) in dry THF (20 mL) at room temperature to obtain the products (3-(hydroxymethyl)-1-phenylbutane-1,4-diol) **1d'** in quantitative yields.

Synthesis of dinucleofuges 1d'':

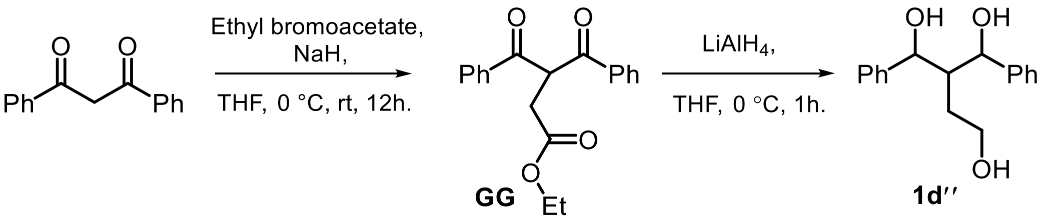

Similar to the synthesis of di-O-centered nucleophiles **1d''**, a substitution reaction of ethyl bromoacetate with dibenzoylmethane generated ethyl 3-benzoyl-4-oxo-4-phenylbutanoate **GG**, followed by then $LiAlH_4$ reduction to give 2-(hydroxy(phenyl)methyl)-1-phenylbutane-1,4-diol **1d''**.

Characterization data of all starting alcohols:

All characterization data for alcohols **1a, 1e, 1g, 1h, 1i, 1j, 1k, 1m, 1n, 1o, 1p, 1q, 1r, 1s, 1t, 1u,** and **1b** which are not reported previously, are supplemented below. Alcohols **1b**, **1c**, **1d**, **1f,** and **1l** were previously reported and the obtained NMR data (see copies of NMR attached below) matched with the reported values.

(*S*)-4-((4-methoxyphenyl)amino)-1-phenylbutan-1-ol (1a)[1]**:**

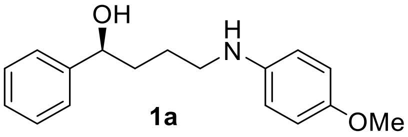

1a

IR (neat) 3360.64, 3028, 2932.25, 2831, 1617, 1512.40, 1455, 1296, 1235.54, 1178, 1119, 1119.18, 1034.64, 913, 819, 749, 701 cm^{-1}. **^{1}H NMR** (400 MHz, Chloroform-d) δ = 7.37 – 7.33 (m, 4H), 7.30 – 7.27 (m, 1H), 6.78 (d, *J* = 9.0 Hz, 2H), 6.61 (d, *J* = 8.9 Hz, 2H), 4.72 (dd, *J* = 7.5, 5.3 Hz, 1H), 3.75 (s, 3H), 3.11 (t, *J* = 6.8 Hz, 2H), 1.89 (dddd, *J* = 10.2, 8.4, 6.7, 5.7 Hz, 1H), 1.81 – 1.71 (m, 1H), 1.70 – 1.60 (m, 2H) ppm. **^{13}C NMR** (100 MHz, $CDCl_3$) δ = 152.5, 144.6, 142.0, 128.5, 127.6, 125.8, 114.9, 114.7, 74.3, 55.8, 45.4, 36.8, 26.0 ppm. **HRMS (ESI)** calcd. for $C_{17}H_{22}NO_2$ [M+H] *m/z* 272.1572 found *m/z* 272.1645. The enantiomeric ratio of **1a** was determined by HPLC analysis using Daicel Chiralcel OD-H column: *n-Hexane*: isopropanol = 90:10, flow rate 1.0 mL/min, λ = 254 nm (channel 1), 232

nm (channel 2): t_1 (major) = 25.45 min, t_2 (minor) = 30.12 min. $[\alpha]_D^{28}$ -21.0, (*c* 0.1, $CHCl_3$)

(*S*)-1-phenyl-4-(phenylamino)butan-1-ol (1b)[1]**:**

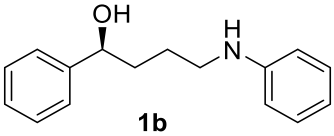

1b

IR (neat) 3354.64, 3029, 2930.25, 2835, 1615, 1510.40, 1465, 1316, 1145.54, 1178, 1119.18, 1034.64, 911, 819, 750, 711 cm^{-1}. **^{1}H NMR** (400 MHz, Chloroform-*d*) δ 7.36 (d, *J* = 4.3 Hz, 4H), 7.32 – 7.25 (m, 1H), 7.21 – 7.12 (m, 2H), 6.70 (tt, *J* = 7.3, 1.1 Hz, 1H), 6.63 – 6.54 (m,

2H), 4.73 (dd, *J* = 7.5, 5.4 Hz, 1H), 3.14 (t, *J* = 6.9 Hz, 2H), 1.97 – 1.82 (m, 2H), 1.81 – 1.72 (m, 1H), 1.69 – 1.60 (m, 1H). **^{13}C NMR** (101 MHz, $CDCl_3$) δ = 148.3, 144.5, 129.2, 128.5, 127.7, 125.8, 117.4, 112.9, 74.3, 44.0, 36.6, 25.9 ppm. **HRMS (ESI)** calcd. for $C_{16}H_{20}NO$ [M+H] *m/z* 242.1548 found *m/z* 242.1545. The enantiomeric ratio of **1b** was determined by HPLC analysis using Daicel Chiralcel OD-H column: *n-Hexane* : *iso*propanol = 90:10, flow rate 1.0 mL/min, λ = 254 nm (channel 1), 232 nm (channel 2): t_1 (major) = 22.1 min, t_2 (minor) = 32.8 min.

(S)-4-mercapto-1-phenylbutan-1-ol (1c)[2]

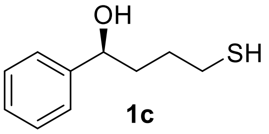

1c

The enantiomeric ratio of **1c** was determined by HPLC analysis using Daicel Chiralcel OD-H column: *n*-Hexane : isopropanol = 95:5, flow rate 0.5 mL/min, λ = 254 nm (channel 1), 232 nm (channel 2): t1 (major) = 24.5 min, t2 (minor) = 26.4 min.

(*S*)-1-phenylbutane-1,4-diol (1d)[3]

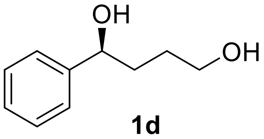

1d

^{1}H NMR (400 MHz, Chloroform-*d*) δ = 7.39 – 7.32 (m, 4H), 7.28 (d, *J* = 5.7 Hz, 1H), 4.74 (t, *J* = 6.3 Hz, 1H), 3.72 – 3.66 (m, 2H), 1.87 (td, *J* = 7.0, 5.9 Hz, 2H), 1.74 – 1.63 (m, 2H) ppm. **^{13}C NMR** (100 MHz, $CDCl_3$) δ = 144.7, 128.4, 127.5, 125.8, 74.4, 62.8, 36.2, 29.2. **HRMS** (**ESI**) calcd. for $C_{10}H_{15}O_2$ [M+H] *m/z* 167.1070 found *m/z* 167.1076. The enantiomeric ratio of **1d** was determined by HPLC analysis using Daicel Chiralcel OD-H column: *n*-Hexane : isopropanol = 95:5, flow rate 0.5 mL/min, λ = 254 nm: t1 (minor) = 44.3 min, t2 (major) = 48.4 min. $[\alpha]_D^{28}$ -55.0, (*c* 0.1, $CHCl_3$)

(*S*)-1-(4-fluorophenyl)-4-((4-methoxyphenyl)amino)butan-1-ol (1e):

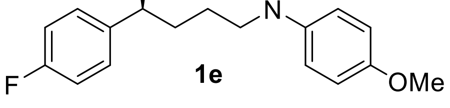

1e

IR (neat) 3367.48, 2994, 2935.19, 2834.38, 1603, 1511.78, 1464, 1386, 1235.4, 1179, 1092.8, 1035.34, 821.49, 755, 718, 574 cm^{-1}. **^{1}H NMR** (400 MHz, Chloroform-*d*) δ 7.36 – 7.31 (m, 4H), 7.26 (d, 1H), 4.89 (t, *J* = 7.2 Hz, 1H), 4.14 – 4.06 (m, 1H), 3.98 – 3.89 (m, 1H), 2.38 – 2.27 (m, 1H), 2.05 – 1.96 (m, 1H), 1.86 – 1.76 (m, 1H) ppm. **^{13}C NMR** (100 MHz, $CDCl_3$) δ = 152.8, 141.6, 140.3, 127.4 (d, $^4J_{C-F}$ = 8.1 Hz), 115.3 (d, $^2J_{C-F}$ = 21.3 Hz), 115.1, 114.9, 73.6, 55.8, 45.6, 37.0, 26.0 ppm. **HRMS** (**ESI**) calcd. for $C_{17}H_{21}FNO_2$ [M+H] *m/z* 290.1569 found *m/z* 290.1551. The enantiomeric ratio of **1e** was determined by HPLC analysis using Daicel Chiralcel OD-H column: *n-Hexane*: isopropanol = 90:10, flow rate 1.0 mL/min, λ = 254 nm (channel 1), 232 nm (channel 2): t_1 (major) = 22.81 min, t_2 (minor) = 28.03 min.

(*S*)-1-(4-fluorophenyl)butane-1,4-diol (**1f**)[4]**:**

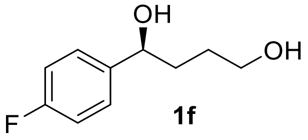

1f

^{1}H NMR (400 MHz, Chloroform-*d*) δ = 7.28 (ddd, *J* = 8.0, 5.1, 2.3 Hz, 2H), 7.07 – 6.94 (m, 2H), 4.66 (t, *J* = 6.3 Hz, 1H), 3.70 – 3.55 (m, 2H), 3.08 (s, 2H), 1.80 (q, *J* = 7.1 Hz, 2H), 1.67 – 1.59 (m, 2H). **^{13}C NMR** (101 MHz, $CDCl_3$) δ 162.0 (d, $^1J_{C-F}$ = 246.0 Hz), 140.4 (d, $^4J_{C-F}$ = 3.2 Hz), 127.3 (d, $^3J_{C-F}$ = 8.1 Hz), 115.1 (d, $^2J_{C-F}$ = 21.5 Hz), 73.6, 62.7, 36.5, 29.0 ppm. **HRMS** (ESI) calcd. for $C_{10}H_{13}FNaO2$ [M+Na] *m/z* 207.0797 found *m/z* 207.0797. The enantiomeric ratio of **1f** was determined by HPLC analysis using Daicel Chiralcel AD column: *n-Hexane*: isopropanol = 95:5, flow rate 1.0 mL/min, λ = 254 nm (channel 1), 232 nm (channel 2): t_1 (major) = 111.0 min, t_2 (minor) = 118.4 min. $[\alpha]_D^{28}$ -53.0, (*c* 0.1, $CHCl_3$)

(*S*)-1-(4-chlorophenyl)-4-((4-methoxyphenyl)amino)butan-1-ol (1g):

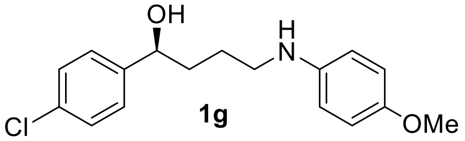

1g

IR (neat) 3370.58, 2935, 2830.34, 1616, 1512.28, 1463, 1365, 1295, 1237.31, 1179, 1088.9, 1036.44, 818.9, 770, 702, 475 cm^{-1}. **^{1}H NMR** (400 MHz, Chloroform-*d*) δ = 7.29 (d, *J* = 5.5 Hz, 4H), 6.81 – 6.75 (m, 2H), 6.66 – 6.56 (m, 2H), 4.70 (dd, *J* = 7.4, 5.3 Hz, 1H), 3.75 (s, 3H),

3.10 (s, 2H), 1.89 – 1.79 (m, 2H), 1.77 – 1.62 (m, 2H) ppm. **^{13}C NMR** (100 MHz, $CDCl_3$) δ = 152.8, 141.6, 127.5, 127.4, 115.4, 115.2, 115.1, 114.9, 73.6, 55.8, 45.6, 37.0, 26.0 ppm. **HRMS** (**ESI**) calcd. for $C_{17}H_{21}ClNO_2$ [M+H] *m/z* 306.1267 found *m/z* 306.1255. The enantiomeric ratio of **1g** was determined by HPLC analysis using Daicel Chiralcel OD-H column: *n-Hexane*: isopropanol = 90:10, flow rate 1.0 mL/min, λ = 254 nm (channel 1), 232 nm (channel 2): t_1 (major) = 45.3 min, t_2 (minor) = 53.7 min. $[\alpha]_D^{28}$ -25.0, (*c* 0.1, $CHCl_3$)

(*S*)-4-((4-methoxyphenyl)amino)-1-(3-(trifluoromethoxy)phenyl)butan-1-ol (1h):

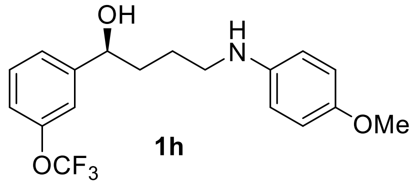

IR (neat) 3371.68, 2934, 2831.44, 1606, 1513.81, 1465, 1360, 1190, 1240.71, 1201, 1080.1, 811.9, 765, 701, 470 cm^{-1}. **^{1}H NMR** (400 MHz, Chloroform-*d*) δ = 7.36 (t, *J* = 7.9 Hz, 1H), 7.26 (s, 1H), 7.23 (s, 1H), 7.15 – 7.10 (m, 1H), 6.81 – 6.74 (m, 2H), 6.65 – 6.56 (m, 2H), 4.76 (t, *J* = 6.3 Hz, 1H), 3.75 (s, 3H), 3.12 (td, *J* = 6.7, 1.7 Hz, 2H), 1.87 (dd, *J* = 7.3, 6.2 Hz, 2H), 1.78 – 1.67 (m, 1H) ppm. **^{13}C NMR** (101 MHz, $CDCl_3$) δ = 152.5, 149.4, 147.2, 142.2, 129.8, 124.1, 121.7, 120.5 (appearant to quartet, $^1J_{C-F}$ = 257.8 Hz), 119.7, 118.3, 114.9, 114.8, 73.5, 55.8, 45.2, 37.1, 26.0 ppm. **HRMS (ESI)** calcd. for $C_{18}H_{21}F_3NO_3$ [M+H] *m/z* 356.1395 found *m/z* 356.1385. The enantiomeric ratio of **1h** was determined by HPLC analysis using Daicel Chiralcel OD-H column: *n-Hexane*: isopropanol = 90:10, flow rate 1.0 mL/min, λ = 254 nm (channel 1), 232 nm (channel 2): t_1 (major) = 16.01 min, t_2 (minor) = 21.1 min. $[\alpha]_D^{28}$ -16.0, (*c* 0.1, $CHCl_3$)

(*S*)-4-((4-methoxyphenyl)amino)-1-(*p*-tolyl)butan-1-ol (1i):

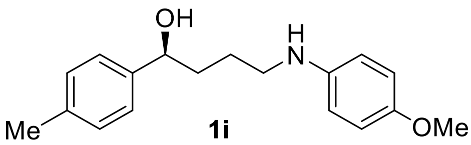

IR (neat) 3361.8, 2937, 2830.86, 1614, 1511.98, 1462, 1293, 1235, 1179, 1119.8, 1035.67, 818.6, 518.77 cm^{-1}. **^{1}H NMR** (400 MHz, Chloroform-*d*) δ = 7.24 (d, *J* = 8.1 Hz, 2H), 7.17 –

7.13 (m, 2H), 6.80 – 6.75 (m, 2H), 6.67 – 6.60 (m, 2H), 4.68 (dd, *J* = 7.5, 5.2 Hz, 1H), 3.75 (s, 3H), 3.14 – 3.07 (m, 3H), 2.34 (s, 3H), 1.94 – 1.81 (m, 2H), 1.78 – 1.71 (m, 1H), 1.65 (dt, *J* = 9.1, 6.2 Hz, 1H) ppm. **^{13}C NMR** (101 MHz, $CDCl_3$) δ = 152.7, 141.6, 137.3, 129.2, 125.8, 115.1, 114.9, 100.0, 74.1, 55.8, 45.7, 36.7, 25.9, 21.1 ppm. **HRMS** (**ESI**)calcd. for $C_{18}H_{23}NO_2Na$ [M+Na] *m/z* 308.1618 found *m/z* 308.1621. The enantiomeric ratio of **1i** was determined by HPLC analysis using Daicel Chiralcel AD column: *n-Hexane* : isopropanol = 90:10, flow rate 1.0 mL/min, λ = 254 nm (channel 1), 232 nm (channel 2): t_1 (major) = 27.1 min, t_2 (minor) = 29.6 min. $[\alpha]^{28}_{D}$ -37.0, (*c* 0.51, $CHCl_3$)

(*S*)-1-(3-methoxyphenyl)-4-((4-methoxyphenyl)amino)butan-1-ol (1j):

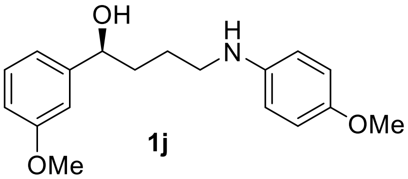

IR (neat) 3370, 3030, 2831, 1616, 1505, 1468, 1440, 1417, 1311, 1267, 1170, 1118, 1094, 1030. 817, 755 cm^{-1}. **^{1}H NMR** (400 MHz, Chloroform-*d*) δ 7.30 – 7.22 (m, 1H), 6.92 (dd, *J* = 4.2, 1.8 Hz, 2H), 6.81 (ddd, *J* = 8.3, 2.6, 1.1 Hz, 1H), 6.79 – 6.75 (m, 2H), 6.59 (d, *J* = 8.9 Hz, 2H), 4.68 (dd, *J* = 7.2, 5.5 Hz, 1H), 3.81 (s, 3H), 3.74 (s, 3H), 3.09 (t, *J* = 6.8 Hz, 2H), 1.94 – 1.81 (m, 2H), 1.78 – 1.70 (m, 1H), 1.69 – 1.61 (m, 1H) ppm. **^{13}C NMR** (100 MHz, $CDCl_3$) δ 159.7, 152.4, 146.4, 129.5, 118.1, 114.9, 114.8, 114.6, 112.9, 111.3, 74.1, 55.8, 55.2, 45.2, 36.8, 26.0 ppm. **HRMS** (**ESI**) calcd. for $C_{18}H_{24}NO_3$[M+H] *m/z* 302.1752 found *m/z* 302.1751. The enantiomeric ratio of **1j** was determined by HPLC analysis using Daicel Chiralcel AD column: *n-Hexane*: isopropanol = 90:10, flow rate 1.0 mL/min, λ = 254 nm (channel 1), 232 nm (channel 2): t_1 (major) = 42.5 min, t_2 (minor) = 48.9 min.

3-(hydroxymethyl)-1-phenylbutane-1,4-diol (1d'):

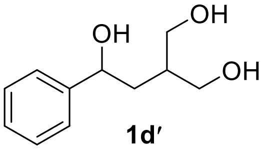

IR (neat) 3337.11, 3063, 3031, 2931.01, 1603, 1493, 1453, 1348, 1205, 1218, 1156, 1028, 913.78, 849, 757.33, 700.74, 553.70 cm^{-1}. **^{1}H NMR** (400 MHz, Chloroform-*d*) δ = 7.38 – 7.30 (m, 4H), 7.29 – 7.22 (m, 1H), 4.80 (dd, *J* = 8.7, 4.0 Hz, 1H), 3.80 – 3.58 (m, 4H), 2.75 (s, 3H), 1.93 (q, *J* = 5.8 Hz, 1H), 1.86 – 1.69 (m, 2H) ppm. **^{13}C NMR** (101 MHz, $CDCl_3$) δ = 144.8, 128.5, 127.6, 125.6, 72.8, 65.4, 65.3, 40.5, 38.6 ppm. **HRMS** (**ESI**) calcd. for $C_{11}H_{16}NaO_3$ [M+Na] *m/z* 219.0994 found *m/z* 219.0992.

2-(hydroxy(phenyl)methyl)-1-phenylbutane-1,4-diol (**1d''**):

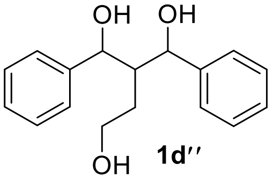

1d''

IR (neat) 3314.70, 3027, 2924.74, 1603, 1493, 1450, 1342, 1202, 1217, 1089, 1047.97, 1028.09, 913.28, 744.08, 701.12, 655 cm^{-1}. **^{1}H NMR** (400 MHz, Chloroform-*d*) δ = 7.45 – 7.35 (m, 4H), 7.34 – 7.24 (m, 3H), 7.23 – 7.11 (m, 3H), 5.05 (d, *J* = 4.4 Hz, 1H), 4.96 (d, *J* = 2.2 Hz, 1H), 3.55 (t, *J* = 6.1 Hz, 2H), 2.15 (dtd, *J* = 7.2, 4.6, 2.2 Hz, 1H), 1.80 (ddt, *J* = 14.2, 8.0, 6.1 Hz, 1H), 1.60 – 1.46 (m, 1H) ppm. **^{13}C NMR** (101 MHz, $CDCl_3$) δ = 143.1, 142.7, 128.5, 128.1, 127.4, 126.8, 125.8, 125.5, 75.4, 72.4, 60.8, 48.4, 27.3 ppm. **HRMS** (**ESI**) calcd. for $C_{17}H_{20}NaO_3$ [M+Na] *m/z* 295.1305 found *m/z* 295.1298.

(*R*)-6-((4-methoxyphenyl)amino)hexan-3-ol (**1k**)[4]**:**

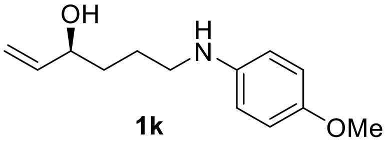

1k

IR (neat) cm^{-1} 3382, 3030, 2931, 2870, 1661, 1614, 1510, 1451, 1354, 1311, 1217, 1170, 1118, 1040, 1036. 917, 754 cm^{-1}. **^{1}H NMR** (400 MHz, Chloroform-*d*) δ = 6.85 – 6.74 (m, 2H), 6.69 – 6.56 (m, 2H), 5.88 (ddd, *J* = 17.2, 10.4, 6.1 Hz, 1H), 5.24 (dt, *J* = 17.2, 1.5 Hz, 1H), 5.12 (dt, *J* = 10.4, 1.4 Hz, 1H), 4.13 (dtd, *J* = 5.9, 4.7, 1.3 Hz, 1H), 3.76 (s, 3H), 3.16 – 3.02 (m, 4H), 1.81 – 1.57 (m, 4H) ppm. **^{13}C NMR** (100 MHz, $CDCl_3$) δ = 152.1, 142.4, 141.0, 114.8, 114.4, 114.4, 72.5, 55.7, 45.0, 34.6, 25.4 ppm. **HRMS (ESI)** calcd. for $C_{13}H_{20}NO_2$ [M+H] 222.1489 *m/z* found 222.1498 *m/z*. The enantiomeric ratio of **1k** was determined by HPLC analysis using

Daicel Chiralcel OJ-H column: *n-Hexane*: isopropanol = 90:10, flow rate 1.0 mL/min, λ = 254 nm (channel 1), 232 nm (channel 2): t_1 (major) = 46.3 min, t_2 (minor) =49.7 min.

(S)-6-mercapto-1-phenylhex-1-yn-3-ol (1l):

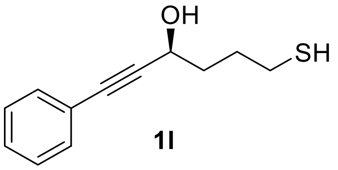

1l

IR (neat) 3351, 2925, 1705, 1598, 1489, 1442, 1412, 1264, 1143, 1050, 914, 755, 734 cm-1. **^{1}H NMR** (400 MHz, Chloroform-*d*) δ = 7.45 – 7.40 (m, 2H), 7.31 (dd, *J* = 5.1, 1.9 Hz, 3H), 4.63 (dd, *J* = 6.7, 5.3 Hz, 1H), 2.79 (t, *J* = 6.8 Hz, 2H), 1.99 – 1.87 (m, 4H) ppm. **^{13}C NMR** (101 MHz, $CDCl_3$) δ 131.7, 128.5, 128.3, 122.5, 89.6, 85.3, 62.5, 38.7, 36.4, 24.8 ppm. **HRMS (ESI)** calcd. for C12H14SO [M]. *m/z* 206.0765 found *m/z* 206.0770.

The enantiomeric ratio of **1l** was determined by HPLC analysis using Daicel Chiralcel OJ-H column: nHexane : isopropanol = 60:40, flow rate 1.0 mL/min, λ = 254 nm (channel 1), 232 nm (channel 2): t1 (minor) = 21.6 min, t2 (major) = 23.4 min. $[\alpha]_D^{28}$ -15.0, (*c* 0.2, $CHCl_3$)

(*R*)-5-((4-methoxyphenyl)amino)pentan-2-ol (**1m**)[4]**:**

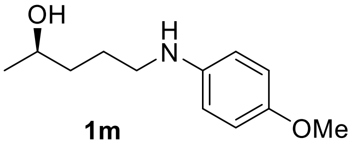

1m

^{1}H NMR (400 MHz, Chloroform-*d*) δ = 6.83 – 6.76 (m, 2H), 6.65 – 6.59 (m, 2H), 3.90 – 3.81 (m, 1H), 3.77 (s, 3H), 3.11 (td, *J* = 6.8, 2.0 Hz, 2H), 1.82 – 1.64 (m, 2H), 1.57 (tdd, *J* = 8.3, 5.9, 3.8 Hz, 2H), 1.22 (d, *J* = 6.2 Hz, 3H) ppm. **^{13}C NMR** (100 MHz, $CDCl_3$) δ = 152.2, 142.5, 114.8, 114.4, 67.7, 55.7, 45.2, 36.9, 26.0, 23.6 ppm. The enantiomeric ratio of **1m** was determined by HPLC analysis using Daicel Chiralcel OJ-H column: *n-Hexane* : isopropanol = 85:15, flow rate 0.5 mL/min, λ = 254 nm (channel 1), 232 nm (channel 2): t_1 (major) = 58.9 min, t_2 (minor) = 63.2 min.

(*S*)-6-((4-methoxyphenyl)amino)hex-1-en-3-ol (**1n**)[4]**:**

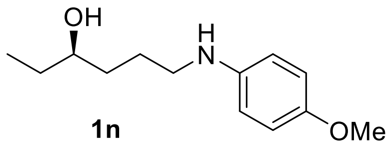

1n

IR (neat) cm^{-1} 3382, 3029, 2935, 2874, 2833, 1660, 1615, 1513.49, 1456.9, 1385, 1238.21, 1179, 1111, 1036.69, 969, 819.74, 753 cm^{-1}. **^{1}H NMR** (400 MHz, Chloroform-*d*) δ = 6.84 – 6.74 (m, 2H), 6.69 – 6.57 (m, 2H), 3.77 (s, 3H), 3.56 (dddd, *J* = 8.5, 7.5, 4.9, 3.7 Hz, 1H), 3.19 – 3.02 (m, 2H), 2.86 (s, 2H), 1.81 – 1.39 (m, 6H), 0.96 (t, *J* = 7.5 Hz, 3H) ppm. **^{13}C NMR** (100 MHz, $CDCl_3$) δ = 152.0, 142.3, 114.6, 114.4, 72.9, 55.6, 45.2, 34.6, 30.2, 25.9, 10.0 ppm. **HRMS** (**ESI**) calcd. For $C_{13}H_{22}NO_2$ [M+H] 224.1645 *m/z* found 224.2652 *m/z*. The enantiomeric ratio of **1n** was determined by HPLC analysis using Daicel Chiralcel OJ-H column: *n-Hexane*: isopropanol = 80:20, flow rate 1.0 mL/min, λ = 254 nm (channel 1), 232 nm (channel 2): t_1 (major) = 14.8 min, t_2 (minor) = 15.4 min. $[\alpha]_D^{28}$ +2.0, (*c* 0.1, $CHCl_3$)

(*S*)-5-((4-methoxyphenyl)amino)-1-phenylpentan-1-ol (1o)[2]:

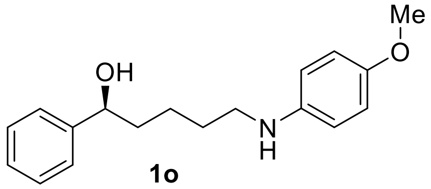

1o

IR (neat) 3362.14, 3029, 2935.27, 2833, 1611, 1522.80, 1450, 1206, 1230.44, 1186, 1121, 1044.51, 911, 820, 789 cm^{-1}. **^{1}H NMR** (400 MHz, Chloroform-*d*) δ = 7.39 – 7.31 (m, 4H), 7.29 (dd, *J* = 6.3, 2.3 Hz, 1H), 6.81 – 6.73 (m, 2H), 6.59 – 6.52 (m, 2H), 4.72 – 4.61 (m, 1H), 3.74 (s, 3H), 3.05 (t, *J* = 7.0 Hz, 2H), 1.91 – 1.80 (m, 1H), 1.80 – 1.70 (m, 1H), 1.68 – 1.58 (m, 2H), 1.57 – 1.48 (m, 1H), 1.40 (ddd, *J* = 10.3, 7.9, 5.5 Hz, 1H) ppm. **^{13}C NMR** (100 MHz, $CDCl_3$) δ = 152.0, 144.7, 142.6, 128.5, 127.6, 125.8, 114.9, 114.1, 74.5, 55.8, 44.8, 38.8, 29.5, 23.4 ppm. **HRMS** (**ESI**) calcd. for $C_{18}H_{24}NO_2$ [M+H] *m/z* 286.1811 found *m/z* 286.1807. The enantiomeric ratio of **1o** was determined by HPLC analysis using Daicel Chiralcel OD-H column: *n-Hexane*: isopropanol = 90:10, flow rate 1.0 mL/min, λ = 254 nm (channel 1), 232 nm (channel 2): t_1 (minor) = 29.23 min, t_2 (major) = 31.38 min. $[\alpha]_D^{28}$ +6.0, (*c* 0.1, $CHCl_3$)

(*S*)-3-(2-((4-methoxyphenyl)amino)phenyl)-1-phenylpropan-1-ol (1p)[2]:

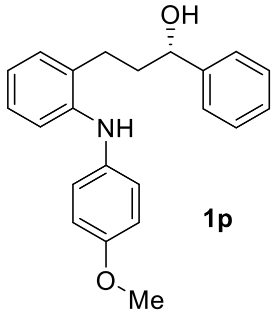

1p

IR (neat) 3366.44, 3031, 2945.55, 2823, 1615, 1520.79, 1441, 1216, 1202.40, 1184, 1116, 1040.50, 916, 821, 780, 711, 498 cm^{-1}. **^{1}H NMR** (400 MHz, Chloroform-*d*) δ = 7.34 (m, 4H), 7.31 – 7.26 (m, 1H), 7.18 – 7.14 (m, 1H), 7.13 – 7.05 (m, 2H), 7.01 – 6.94 (m, 2H), 6.89 – 6.82 (m, 3H), 4.68 (dd, *J* = 8.7, 4.4 Hz, 0H), 3.80 (s, 1H), 2.82 – 2.68 (m, 1H), 2.16 – 1.99 (m, 0H) pmp. **^{13}C NMR** (101 MHz, $CDCl_3$) δ = 154.8, 144.4, 142.9, 137.0, 130.0, 129.7, 128.5, 127.7, 126.9, 125.8, 121.3, 120.5, 116.8, 114.7, 73.4, 55.6, 39.0, 27.2 ppm. **HRMS** (**ESI**) calcd. for $C_{22}H_{23}NO_2Na$ [M+Na] *m/z* 356.1621 found *m/z* 235.1631.

The enantiomeric ratio of **1p** was determined by HPLC analysis using Daicel Chiralcel OD-H column: *n*-Hexane: isopropanol = 90:10, flow rate 1.0 mL/min, λ = 254 nm (channel 1), 232 nm (channel 2): t_1 (major) = 26.2 min, t_2 (minor) = 46.6 min.

(*R*)-2-(3-hydroxy-3-phenylpropyl)phenol (1q):

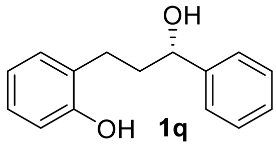

1q

^{1}H NMR (400 MHz, Chloroform-*d*) δ = 7.41 – 7.27 (m, 6H), 7.21 – 7.12 (m, 2H), 6.91 (td, *J* = 7.5, 1.4 Hz, 2H), 4.66 (dd, *J* = 10.3, 3.6 Hz, 1H), 2.99 (ddd, *J* = 14.2, 10.6, 6.1 Hz, 1H), 2.77 (ddd, *J* = 14.3, 6.5, 4.1 Hz, 1H), 2.51 (d, *J* = 35.1 Hz, 1H), 2.14 (dddd, *J* = 14.3, 10.3, 6.1, 4.1 Hz, 1H), 2.05 – 1.90 (m, 1H) ppm. **^{13}C NMR** (100 MHz, $CDCl_3$) δ = 154.6, 143.9, 130.5, 128.6, 127.9, 127.7, 127.1, 125.8, 120.8, 116.2, 73.1, 39.3, 25.9 ppm.

The enantiomeric ratio of **1q** was determined by HPLC analysis using Daicel Chiralcel OJ-H column: *n-Hexane*: isopropanol = 90:10, flow rate 0.5 mL/min, λ = 254 nm (channel 1), 232 nm (channel 2): t_1 (major) = 43.8 min, t_2 (minor) = 46.4 min. $[\alpha]_D^{28}$ -27.0, (*c* 0.1, $CHCl_3$)

(*R*)-2-(3-(4-fluorophenyl)-3-hydroxypropyl)phenol (1r):

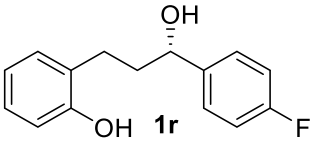

IR (neat) 3357, 2934.80, 2836.60, 1622, 1555, 1513, 1473, 1464, 1237, 1179, 1116, 1035, 992, 922, 821 cm^{-1}. **^{1}H NMR** (400 MHz, Chloroform-*d*) δ = 7.34 – 7.27 (m, 2H), 7.15 (ddd, *J* = 8.5, 7.0, 1.6 Hz, 2H), 7.08 – 6.98 (m, 2H), 6.95 – 6.83 (m, 2H), 4.63 (dd, *J* = 10.2, 3.6 Hz, 1H), 2.95 (ddd, *J* = 14.1, 10.3, 6.3 Hz, 1H), 2.75 (ddd, *J* = 14.3, 6.7, 4.2 Hz, 2H), 2.10 (dddd, *J* = 14.3, 10.4, 6.2, 4.2 Hz, 1H), 1.95 (dddd, *J* = 14.0, 10.3, 6.7, 3.6 Hz, 1H) ppm. **^{13}C NMR** (100 MHz, $CDCl_3$) δ = 162.3 (d, $^1J_{C\text{-}F}$ = 245.9 Hz), 154.4, 139.7 (d, $^4J_{C\text{-}F}$ = 3.2 Hz), 130.6, 127.7, 127.5 (d, $^3J_{C\text{-}F}$ = 8.1 Hz), 127.1, 120.8, 116.1, 115.4 (d, $^2J_{C\text{-}F}$ = 21.5 Hz), 72.4, 39.4, 25.9 ppm. **HRMS** (**ESI**) calcd. for $C_{15}H_{15}FO_2Na$ [M+Na] 269.0948 *m/z* found 269.0973 *m/z*. The enantiomeric ratio of **1r** was determined by HPLC analysis using Daicel Chiralcel AD column: *n-Hexane* : isopropanol = 90:10, flow rate 1.0 mL/min, λ = 254 nm (channel 1), 232 nm (channel 2): t_1 (minor) = 12.3 min, t_2 (major) = 16.2 min. $[\alpha]_D^{28}$ -64.0, (*c* 0.15, $CHCl_3$)

(*S*)-2-phenyl-4-(2-(phenylamino)phenyl)butan-2-ol (1s)[5]:

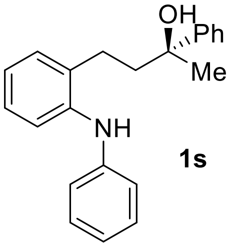

IR (neat) 3355, 3141, 2901.89, 2853.68, 1609.54, 1515.43, 1389.12, 1360, 1256, 1223.34, 1189.64, 1155, 1015, 816, 711, 459 cm^{-1}. **^{1}H NMR** (400 MHz, Chloroform-*d*) δ = 7.47 – 7.42 (m, 2H), 7.39 – 7.33 (m, 2H), 7.29 – 7.26 (m, 1H), 7.25 – 7.21 (m, 2H), 7.15 – 7.08 (m, 2H), 6.96 – 6.86 (m, 5H), 2.66 – 2.57 (m, 1H), 2.40 (ddd, *J* = 14.0, 11.0, 5.1 Hz, 1H), 2.18 – 2.00 (m, 1H), 1.59 (s, 3H) ppm. **^{13}C NMR** (100 MHz, $CDCl_3$) δ = 147.3, 144.2, 140.6, 132.3, 130.0, 129.2, 128.3, 126.8, 126.7, 124.7, 121.8, 120.2, 119.2, 117.2, 74.8, 44.0, 30.6, 26.2. **HRMS** (ESI) calcd. for $C_{22}H_{23}NONa$ [M+Na] *m/z* 340.1671 found *m/z* 340.1672. The enantiomeric ratio of **1s** was determined by HPLC analysis using Daicel Chiralcel OD-H column: *n-Hexane*:

isopropanol = 90:10, flow rate 1.0 mL/min, λ = 254 nm (channel 1), 232 nm (channel 2): t_1 (minor) = 8.7 min, t_2 (major) = 10.1 min. $[\alpha]_D^{28}$ +24.0, (*c* 0.1, $CHCl_3$)

(*S*)-2-(3-hydroxy-3-phenylbutyl)phenol (1t)[5]**:**

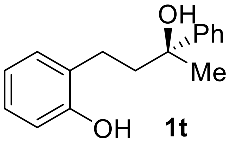

1t

IR (neat) 3361.83, 3058,, 3028, 2975, 2929, 1582.51, 1489, 1455.94, 1374, 1243, 1218, 1119, 1065, 1029, 944, 890, 753.71, 699, 548 cm^{-1}. **^{1}H NMR** (400 MHz, Chloroform-*d*) δ = 7.54 – 7.44 (m, 2H), 7.37 (dd, *J* = 8.5, 6.9 Hz, 2H), 7.30 – 7.23 (m, 1H), 7.11 – 7.05 (m, 1H), 7.00 (dd, *J* = 7.4, 1.8 Hz, 1H), 6.87 – 6.75 (m, 2H), 6.57 (s, 1H), 2.65 (td, *J* = 9.4, 4.8 Hz, 1H), 2.46 (ddt, *J* = 15.8, 9.4, 4.7 Hz, 1H), 2.21 (ddd, *J* = 14.3, 9.4, 6.2 Hz, 1H), 2.07 (ddd, *J* = 14.5, 9.4, 5.5 Hz, 1H), 1.63 (s, 3H). **^{13}C NMR** (101 MHz, $CDCl_3$) δ = 153.8, 147.1, 130.0, 128.3, 127.5, 126.8, 125.4, 124.7, 120.4, 115.9, 75.5, 43.9, 30.5, 24.8 ppm. **HRMS** (**ESI**) calcd. for $C_{16}H_{18}O_2Na$ [M+Na] *m/z* 265.1211 found *m/z* 265.1199.

The enantiomeric ratio of **1t** was determined by HPLC analysis using Daicel Chiralcel OD-H column: *n-Hexane*: isopropanol = 90:10, flow rate 1.0 mL/min, λ = 254 nm (channel 1), 232 nm (channel 2): t_1 (minor) = 11.1 min, t_2 (major) =12.8 min. $[\alpha]_D^{28}$ +8.0, (*c* 0.04, $CHCl_3$)

(*R*)-2-(3-hydroxy-3,7-dimethyloctyl)phenol (**1u**)[5]**:**

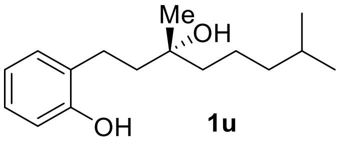

1u

IR (neat) 3338.03, 3071, 3036, 2953, 2868, 1593.65, 1490, 1457.54, 1366, 1243, 1175, 1089, 1041, 912, 847, 751.42 cm^{-1}. **^{1}H NMR** (400 MHz, Chloroform-*d*) δ = 7.09 – 6.97 (m, 2H), 6.82 – 6.72 (m, 2H), 2.71 – 2.56 (m, 2H), 1.74 – 1.66 (m, 2H), 1.53 – 1.38 (m, 3H), 1.31 – 1.22 (m, 2H), 1.18 (s, 3H), 1.10 (dd, *J* = 7.7, 1.1 Hz, 2H), 0.80 (d, *J* = 6.6 Hz, 6H) ppm. **^{13}C NMR** (101 MHz, $CDCl_3$) δ = 154.0, 130.0, 128.8, 127.4, 120.2, 116.2, 73.9, 42.4, 41.0, 39.2, 27.9, 26.7, 24.3, 22.6, 21.9 ppm. **HRMS** (ESI) calcd. for $C_{16}H_{26}O_2Na$ [M+Na] *m/z* 273.1837 found *m/z* 273.1825. The enantiomeric ratio of **1u** was determined by HPLC analysis using Daicel

Chiralcel OJ-H column: *n-Hexane*: isopropanol = 90:10, flow rate 0.5 mL/min, λ = 254 nm (channel 1), 232 nm (channel 2): t_1 (major) = 15.8 min, t_2 (minor) = 21.2 min. $[\alpha]_D^{28}$ +17.0, (*c* 0.5, $CHCl_3$)

(S)-2-(3-hydroxy-3,7-dimethyloctyl)benzene-1,3-diol (1u′):

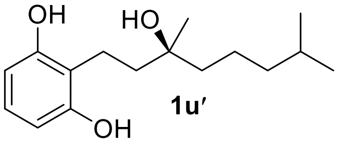

^{1}H NMR (400 MHz, Chloroform-*d*) δ = 6.94 (t, *J* = 8.1 Hz, 1H), 6.41 (d, *J* = 8.1 Hz, 2H), 5.99 (s, 2H), 2.74 (td, *J* = 7.0, 2.9 Hz, 2H), 1.77 (t, *J* = 7.0 Hz, 2H), 1.55 – 1.44 (m, 3H), 1.32 (dd, *J* = 8.0, 2.0 Hz, 2H), 1.23 (s, 3H), 1.17 (dd, *J* = 8.0, 6.7 Hz, 2H), 0.87 (d, *J* = 6.6 Hz, 6H) ppm. **^{13}C NMR** (101 MHz, $CDCl_3$) δ = 154.8, 127.0, 116.4, 108.2, 74.2, 39.7, 39.4, 22.6, 22.6, 21.9 ppm. $[\alpha]_D^{28}$ -3.0, (*c* 0.1, $CHCl_3$)

(S)-1-(2-mercaptophenyl)-3,7-dimethyloctan-3-ol (1v)[6,7]:

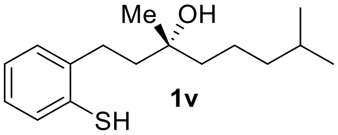

^{1}H NMR (400 MHz, Chloroform-*d*) δ 7.28 (d, *J* = 1.5 Hz, 1H), 7.21 – 7.01 (m, 3H), 3.36 (s, 1H), 2.80 – 2.68 (m, 2H), 1.80 – 1.68 (m, 2H), 1.60 – 1.48 (m, 3H), 1.43 – 1.32 (m, 2H), 1.27 (s, 3H), 1.24 – 1.14 (m, 2H), 0.89 (d, *J* = 6.6 Hz, 6H) ppm. **^{13}C NMR** (101 MHz, $CDCl_3$) δ 140.6, 130.8, 130.2, 129.6, 126.6, 126.2, 72.8, 42.3, 41.8, 39.5, 29.2, 28.0, 26.8, 22.6, 21.8 ppm. **HRMS** (ESI) calcd. for $C_{16}H_{26}NaOS$ [M+Na] *m/z* 289,1602 found *m/z* 289,1595. The enantiomeric ratio of **1v** was determined by HPLC analysis using Daicel Chiralcel OJ-H column: *n-Hexane*: isopropanol = 99.5:0.5, flow rate 0.5 mL/min, λ = 254 nm (channel 1), 232 nm (channel 2): t_1 (major) = 76.3 min, t_2 (minor) = 92.3. min. $[\alpha]_D^{28}$ -3.0, (*c* 0.16, $CHCl_3$)

Characterization data of all final products:

(*R*)-1-(4-methoxyphenyl)-2-phenylpyrrolidine (2a)[9,10]

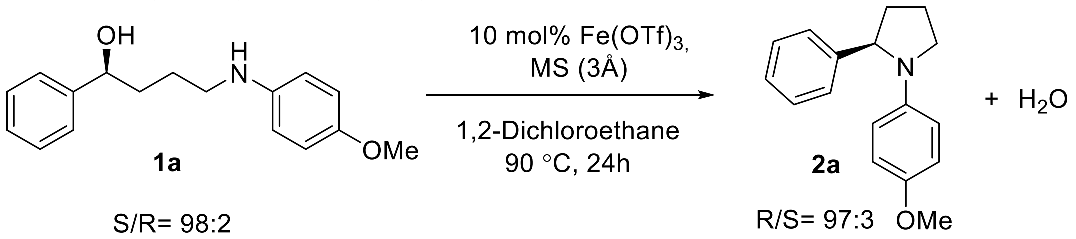

To an oven-dried 5 ml vial equipped with a magnetic stir bar was added substrate amino-alcohol **1a** (135.5 mg, 0.5 mmol), MS (3Å) (300 mg), and $Fe(OTf)_3$ (25.05 mg, 0.05 mmol). The tube was sealed with a teflon-lined cap, connected to a vacuum and backfilled with argon three times by piercing with a needle attached to a Schlenk line. Then 2.0 ml of anhydrous DCE was added by syringe and the mixture was stirred at 90 °C for 24 hours. After this, the reaction was cooled to room temperature and the crude was concentrated under vacuum. The crude residue was purified by column chromatography with ethyl acetate and hexanes (1:20) as solvent to obtain the pure product **2a** (98%, 133 mg) as colorless oil. **IR** (neat) 3059, 3044, 2966, 2901, 2829, 1618, 1513, 1490, 1450, 1363, 1262, 1240, 1179, 1174, 1042, 966, 811.93, 770.69, 747.12, 589.98, 519.20 cm^{-1}. **^{1}H NMR** (400 MHz, Chloroform-*d*) δ = 7.37 – 7.32 (m, 4H), 7.28 (d, *J* = 4.2 Hz, 1H), 6.82 – 6.74 (m, 2H), 6.66 (d, *J* = 8.9 Hz, 2H), 4.73 (dd, *J* = 7.5, 5.2 Hz, 1H), 3.75 (s, 3H), 3.12 (td, *J* = 6.8, 1.9 Hz, 2H), 1.94 – 1.84 (m, 2H), 1.81 – 1.73 (m, 1H), 1.72 – 1.64 (m, 1H) ppm. **^{13}C NMR** (101 MHz, $CDCl_3$) δ = 131.7, 128.5, 127.6, 125.8, 115.3, 115.2, 114.9, 100.0, 74.3, 55.8, 45.8, 36.8, 25.9 ppm. **HRMS** (**ESI**) calcd. for $C_{17}H_{20}NONa$ [M+Na] *m/z* 254.1546 found *m/z* 254.1539.

The enantiomeric ratio of **2a** was determined by HPLC analysis using Daicel Chiralcel OJ-H column: *n*-Hexane : isopropanol = 95:05, flow rate 0.5 mL/min, λ = 254 nm (channel 1): t_1 (minor) = 12.7 min, t_2 (major) = 14.0 min. $[\alpha]_D^{28}$ +1.0, (*c* 0.04, $CHCl_3$)

(R)-1,2-diphenylpyrrolidine (2b)

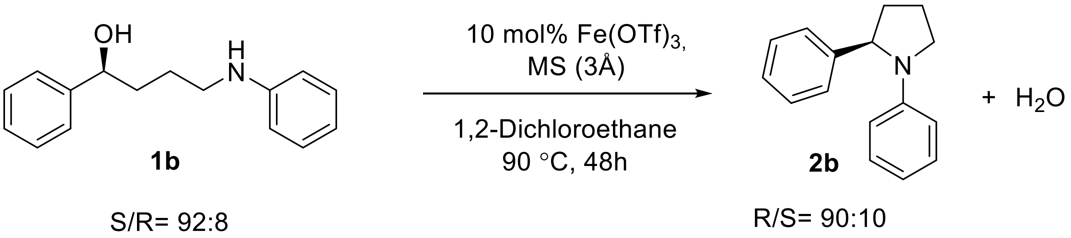

Alcohol **1b** (120.5 mg, 0.5 mmol), MS (3Å) (300 mg), and the catalyst $Fe(OTf)_3$ (25.05 mg, 0.05 mmol) were treated as described for **1a** for 48 h. After completion of reaction (TLC), the crude was concentrated under vacuum and purified by a fast column chromatographic using silica gel (mess 100-200) and dichloromethane eluent obtain pure **2b** (115.6 mg, 0.96 mmol, 96% yield) as colorless oil. **IR** (neat) 3060, 3034, 2965, 2911, 2834, 1601, 1512, 1455, 1362, 1264, 1239, 1180, 1101, 1034, 965, 812.73, 771.90, 740.13, 584 cm^{-1}. **^{1}H NMR** (400 MHz, Chloroform-*d*) δ = 7.36 – 7.19 (m, 5H), 7.15 (t, *J* = 7.8 Hz, 2H), 6.64 (t, *J* = 7.3 Hz, 1H), 6.50 (d, *J* = 8.0 Hz, 2H), 4.73 (d, *J* = 8.3 Hz, 1H), 3.70 (d, *J* = 8.6 Hz, 1H), 3.42 (q, *J* = 8.4 Hz, 1H), 2.38 (tt, *J* = 11.0, 7.9, 5.9 Hz, 1H), 2.00 (m, Hz, 3H) ppm. **^{13}C NMR** (100 MHz, $CDCl_3$) *δ* = 147.1, 144.6, 128.9, 128.4, 126.6, 125.9, 115.7, 112.3, 62.9, 49.1, 36.0, 23.0 ppm. **HRMS** (**ESI**) calcd. for $C_{16}H_{17}NNa$ [M+Na] *m/z* 246.1261 found *m/z* 246.1251.

The enantiomeric ratio of **2b** was determined by HPLC analysis using Daicel Chiralcel OJ-H column: *n*-Hexane: isopropanol = 95:5, flow rate 0.5 mL/min, λ = 254 nm (channel 1), 232 nm (channel 2): t_1 (major) = 8.3 min, t_2 (minor) = 9.9 min.

(R)-2-phenyltetrahydrothiophene (2c)

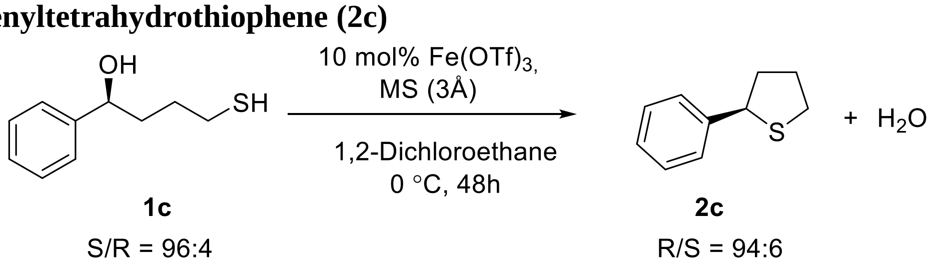

Alcohol **1c** (91 mg, 0.5 mmol), MS (3Å) (400 mg), and the catalyst $Fe(OTf)_3$ (25.05 mg, 0.05 mmol) were treated as described for **1a** for 48 h at 0 °C and purified as described for **1a** to obtain **2c** (80.30 mg, 0.487 mmol, 98% yield) as a colorless oil. **^{1}H NMR** (400 MHz, Chloroform-*d*) δ = 7.42 (ddd, *J* = 8.2, 1.3, 0.5 Hz, 2H), 7.34 – 7.27 (m, 2H), 7.25 – 7.19 (m,

1H), 4.52 (dd, *J* = 8.5, 6.1 Hz, 1H), 3.21 – 3.11 (m, 1H), 3.06 – 2.97 (m, 1H), 2.43 – 2.35 (m, 1H), 2.32 – 2.23 (m, 1H), 2.04 – 1.89 (m, 2H) ppm. **^{13}C NMR** (101 MHz, $CDCl_3$) δ 143.0, 128.4, 127.6, 127.0, 52.7, 40.5, 33.5, 31.0 ppm.

The enantiomeric ratio of **2c** was determined by HPLC analysis using Daicel Chiralcel OD-H column: *n*Hexane : isopropanol = 95:5, flow rate 0.5 mL/min, λ = 254 nm (channel 1), 232 nm (channel 2): t1 (major) = 9.2 min, t2 (minor) = 11.0 min.

(R)-2-phenyltetrahydrofuran (2d)[9]

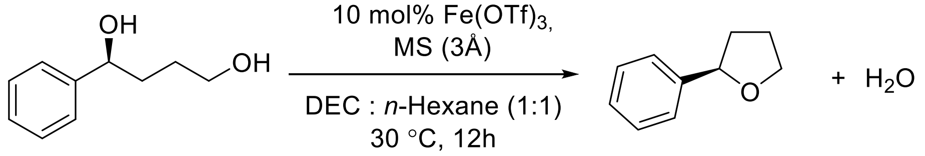

1d **2d**

S/R = 07:93 S/R = 88:12

Alcohol **1d** (83 mg, 0.5 mmol), MS (3Å) (300 mg), and the catalyst $Fe(OTf)_3$ (25.05 mg, 0.05 mmol) were treated as described for **1a** for 12 h. After completion of reaction (TLC), the crude was concentrated under vacuum and purified by a fast column chromatographic using silica gel (mess 100-200) and dichloromethane eluent obtain pure **2d** (82 mg, 0.98.8 mmol, 99% yield) as colorless oil. **^{1}H NMR** (400 MHz, Chloroform-*d*) δ = 7.37 – 7.30 (m, 4H), 7.28 – 7.22 (m, 1H), 4.90 (t, *J* = 7.2 Hz, 1H), 4.10 (dt, *J* = 8.3, 6.8 Hz, 1H), 3.94 (td, *J* = 7.8, 6.4 Hz, 1H), 2.38 – 2.26 (m, 1H), 2.07–1.95 (m, 2H), 1.87 – 1.76 (m, 1H) ppm. **^{13}C NMR** (101 MHz, $CDCl_3$) δ = 143.5, 128.3, 127.1, 125.6, 80.7, 68.6, 34.6, 26.0 ppm. The enantiomeric ratio of **2d** was determined by HPLC analysis using Daicel Chiralcel OJ-H column: *n*-Hexane: isopropanol = 95:05, flow rate 0.5 mL/min, λ = 254 nm (channel 1): t_1 (minor) = 9.2 min, t_2 (major) = 10.1 min. $[\alpha]_D^{28}$ +2.0, (*c* 0.06, $CHCl_3$)

(*R*)-2-(4-fluorophenyl)-1-(4-methoxyphenyl)pyrrolidine (2e)

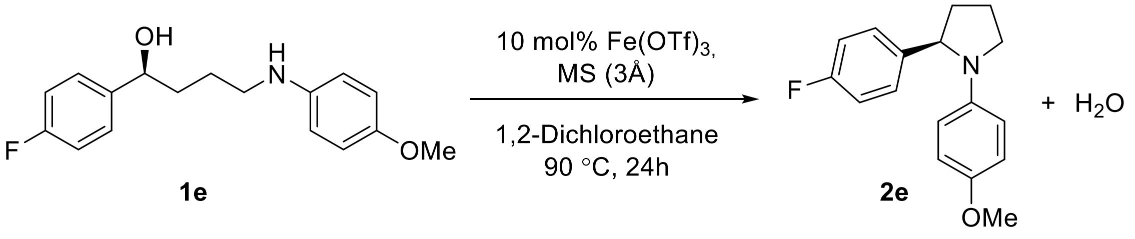

1e **2e**

S/R= 98:2 R/S= 95:5

Alcohol **1e** (144.5 mg, 0.5 mmol), MS (3Å) (300 mg), and the catalyst Fe(OTf)$_3$ (25.05 mg, 0.05 mmol) were treated as described for **1a** for 24 h and purified as described for **1a** to obtain **2e** (128.7 mg, 0.474 mmol, 95% yield) as a yellowish oil. **IR** (neat) 3061, 3045, 2963, 2911, 2815, 1611, 1525, 1493, 1451, 1362, 1281, 1229, 1178, 1177, 1044, 965, 812.23, 771.19, 737.12, 701.38, 523.21 cm^{-1}. **^{1}H NMR** (400 MHz, Chloroform-*d*) δ = 7.20 (s, 2H), 7.03 – 6.89 (m, 2H), 6.81 – 6.70 (m, 2H), 6.42 (d, *J* = 8.4 Hz, 2H), 4.60 (d, *J* = 8.5 Hz, 1H), 3.71 (s, 3H), 3.64 – 3.70 (m1H), 3.34 (q, *J* = 8.3 Hz, 1H), 2.38 (t, *J* = 10.3 Hz, 1H), 1.98 (s, 2H), 1.88 (s, 1H) ppm. **^{13}C NMR** (101 MHz, CDCl$_3$) δ 161.6 (d, $^1J_{C-F}$ = 244.9 Hz), 151.0, 142.0, 140.7, 127.4 (d, $^3J_{C-F}$ = 8.1 Hz), 115.2 (d, $^2J_{C-F}$ = 21.4 Hz), 114.9, 113.1, 62.8, 55.9, 49.7, 36.3, 23.3. **HRMS** (**ESI**) calcd. for $C_{17}H_{19}NFO$ [M+H] *m/z* 272.1447 found *m/z* 272.1445. The enantiomeric ratio of **2e** was determined by HPLC analysis using Daicel Chiralcel OJ-H column: *n*-Hexane : isopropanol = 95:5, flow rate 0.5 mL/min, λ = 254 nm (channel 1), 232 nm (channel 2): t_1 (minor) = 10.4 min, t_2 (major) = 18.1 min. $[\alpha]_D^{28}$ +2.0, (*c* 0.1, CHCl$_3$)

(*R*)-2-(4-fluorophenyl)tetrahydrofuran (2f)[10]

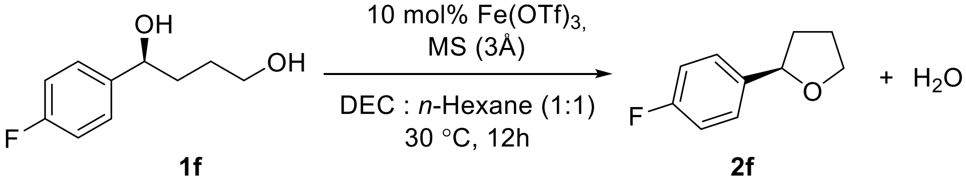

1f **2f**

S/R = 90:10 R/S = 88:12

Alcohol **1f** (83 mg, 0.5 mmol), MS (3Å) (300 mg), and the catalyst Fe(OTf)$_3$ (25.05 mg, 0.05 mmol) were treated as described for **1a** for 12 h. After completion of reaction (TLC), the crude was concentrated under vacuum and purified by a fast column chromatographic using silica gel (mess 100-200) and dichloromethane eluent obtain pure **2f** (82 mg, 0.98.8 mmol, 99% yield) as colorless oil. **^{1}H NMR** (400 MHz, CDCl$_3$) δ = 7.31-7.26 (m, 2H), 7.03-6.98 (m, 2H), 4.85 (t, *J* = 6.8 Hz), 4.08 (q, *J* = 8.0 Hz, 1H), 3.94 (q, *J* = 7.6 Hz, 1H), 2.34-2.26 (m, 1H), 2.04-1.96 (m,

1H), 1.80-1.71 (m, 1H) ppm. **^{13}C NMR** (100 MHz, $CDCl_3$) 162 .0 (d, $^1J_{C\text{-}F}$ = 245.6 Hz), 139.1 (d, $^4J_{C\text{-}F}$ = 3.2 Hz), 127.2 (d, $^3J_{C\text{-}F}$ = 8.0), 115.0 (d, $^2J_{C\text{-}F}$ = 21.3), 80.1, 68.6, 34.6, 25.9 ppm. The enantiomeric ratio of **2f** was determined by HPLC analysis using Daicel Chiralcel OJ-H column: *n*-Hexane : isopropanol = 80:20, flow rate 0.5 mL/min, λ = 254 nm (channel 1): t_1 (major) = 12.3 min, t_2 (minor) = 13.9 min. $[\alpha]_D^{28}$ +3.0, (*c* 0.2, $CHCl_3$)

(*R*)-2-(4-chlorophenyl)-1-(4-methoxyphenyl)pyrrolidine (**2g**)

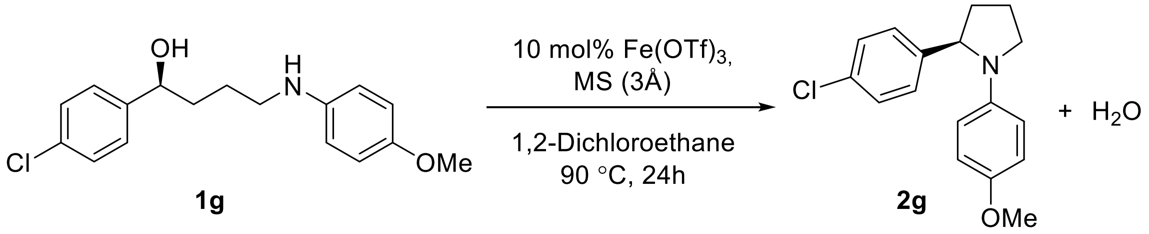

1g **2g**

S/R= 97:3 R/S= 97:3

Alcohol **1g** (152.5 mg, 0.5 mmol), MS (3Å) (300 mg), and the catalyst $Fe(OTf)_3$ (25.05 mg, 0.05 mmol) were treated as described for **1a** for 24 h and purified as described for **1a** to obtain **2g** (140.6 mg, 0.489 mmol, 98% yield) as a yellowish oil. **IR** (neat) 3060, 3055, 2910, 2825, 1615, 1531, 1490, 1450, 1356, 1280, 1232.45, 1188, 1167.35, 1063.54, 960, 811.33, 770.29, 717.11, 701.41, 520.83 cm^{-1}. **^{1}H NMR** (400 MHz, Chloroform-*d*) δ = 7.33 – 7.23 (m, 2H), 7.19 (s, 2H), 6.82 – 6.70 (m, 2H), 6.41 (d, *J* = 8.4 Hz, 2H), 4.59 (dd, *J* = 8.6, 2.7 Hz, 1H), 3.71 (s, 3H), 3.70 (s, 1H), 3.34 (q, *J* = 8.3 Hz, 1H), 2.44 – 2.28 (m, 1H), 1.98 (s, 2H), 1.87 (s, 1H). **^{13}C NMR** (101 MHz, $CDCl_3$) δ = 151.1, 143.7, 141.9, 132.2, 128.6, 127.4, 114.9, 113.1, 62.9, 55.9, 49.7, 36.2, 23.3 ppm. **HRMS** (**ESI**) calcd. for $C_{17}H_{19}ClNO$ [M+H] *m/z* 288.1158 found *m/z* 288.11150. The enantiomeric ratio of **2g** was determined by HPLC analysis using Daicel Chiralcel OJ-H column: *n*-Hexane : isopropanol = 95:5, flow rate 0.5 mL/min, λ = 254 nm (channel 1), 232 nm (channel 2): t_1 (minor) = 24.0 min, t_2 (major) = 33.4 min. $[\alpha]_D^{28}$ +41.0, (*c* 0.5, $CHCl_3$)

(*R*)-1-(4-methoxyphenyl)-2-(3-(trifluoromethoxy)phenyl)pyrrolidine (**2h**)

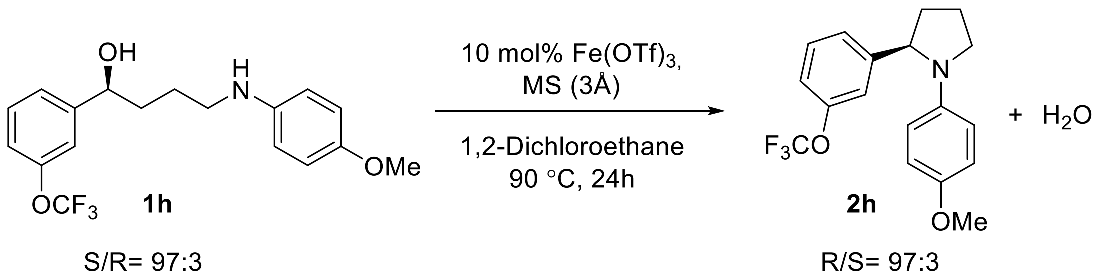

Alcohol **1h** (177.5 mg, 0.5 mmol), MS (3Å) (300 mg), and the catalyst $Fe(OTf)_3$ (25.05 mg, 0.05 mmol) were treated as described for **1a** for 24 h and purified as described for **1a** to obtain **2h** (165.5 mg, 0.485 mmol, 91% yield) as a yellowish oil. **IR** (neat) 3061, 3053, 2921, 2822, 1611, 1521, 1493, 1451, 1352, 1271, 1222.35, 1178, 1060.57, 963, 801.34, 771, 701.41 cm^{-1}. **^{1}H NMR** (400 MHz, Chloroform-*d*) δ = 7.31 (t, *J* = 7.8 Hz, 1H), 7.18 (dd, *J* = 7.7, 1.3 Hz, 1H), 7.13 – 7.04 (m, 2H), 6.81 – 6.71 (m, 2H), 6.47 – 6.38 (m, 2H), 4.62 (dd, *J* = 8.5, 2.5 Hz, 1H), 3.72 (s, 3H), 3.71 – 3.63 (m, 1H), 3.35 (td, *J* = 8.7, 7.1 Hz, 1H), 2.40 (m, 1H), 2.00 (m, 2H), 1.91 (m, 1H). **^{13}C NMR** (101 MHz, $CDCl_3$) δ 151.1, 149.6, 147.9, 141.9, 129.8, 124.2, 120.5 (q, $^1J_{C-F}$ = 257.8 Hz), 118.8, 118.5, 114.8, 113.1, 63.1, 55.9, 49.7, 36.1, 23.3. **HRMS (ESI)** calcd. for $C_{18}H_{18}F_3NO_2$ [M+H] *m/z* 338.1323 found *m/z* 338.1320. The enantiomeric ratio of **2h** was determined by HPLC analysis using Daicel Chiralcel OJ-H column: *n*-Hexane : isopropanol = 90:10, flow rate 1.0 mL/min, λ = 254 nm (channel 1), 232 nm (channel 2): t_1 (major) = 7.18 min, t_2 (minor) = 9.5 min. $[\alpha]_D^{28}$ +13.0, (*c* 0.1, $CHCl_3$)

(*R*)-1-(4-methoxyphenyl)-2-(p-tolyl)pyrrolidine (2i)

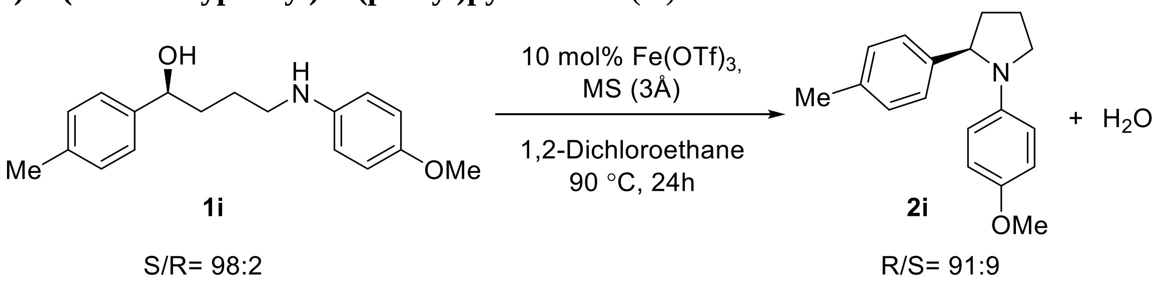

Alcohol **1i** (143.5 mg, 0.5 mmol), MS (3Å) (300 mg), and the catalyst $Fe(OTf)_3$ (25.05 mg, 0.05 mmol) were treated as described for **1a** for 24 h and purified as described for **1a** to obtain **2i** (124.1 mg, 0.464 mmol, 93% yield) as a colorless oil. **IR** (neat) 3068, 3050, 2911, 2821,

1609, 1530, 1491, 1453, 1372, 1273, 1231.42, 1137, 1166.32, 1064.43, 961, 816.43, 771.49, 707.13, 521.93 cm^{-1}. **^{1}H NMR** (400 MHz, Chloroform-*d*) δ = 7.27 – 7.17 (m, 4H), 6.91 – 6.81 (m, 2H), 6.60 – 6.51 (m, 2H), 4.72 (dd, *J* = 8.3, 2.5 Hz, 1H), 3.81 (s, 3H), 3.80 – 3.75 (m, 1H), 3.45 (td, *J* = 8.9, 6.6 Hz, 1H), 2.53 – 2.45 (m, 1H), 2.44 (s, 3H), 2.21 – 2.09 (m, 1H), 2.08 – 1.96 (m, 2H) ppm. **^{13}C NMR** (100 MHz, $CDCl_3$) δ = 150.7, 142.2, 142.1, 136.0, 129.1, 125.8, 114.8, 112.9, 63.1, 55.9, 49.6, 43.4, 36.3, 23.3, 21.0 ppm. **HRMS** (**ESI**) calcd. for $C_{18}H_{22}NO$ [M+H] *m/z* 268.1705 found *m/z* 268.1696. The enantiomeric ratio of **2i** was determined by HPLC analysis using Daicel Chiralcel OJ-H column: *n*-Hexane : isopropanol = 95:5, flow rate 1.0 mL/min, λ = 254 nm (channel 1), 232 nm (channel 2): t_1 (major) = 12.9 min, t_2 (major) = 14.5 min. $[\alpha]_D^{28}$ +21.0, (*c* 0.1, $CHCl_3$)

(*R*)-2-(3-methoxyphenyl)-1-(4-methoxyphenyl)pyrrolidine (**2j**)

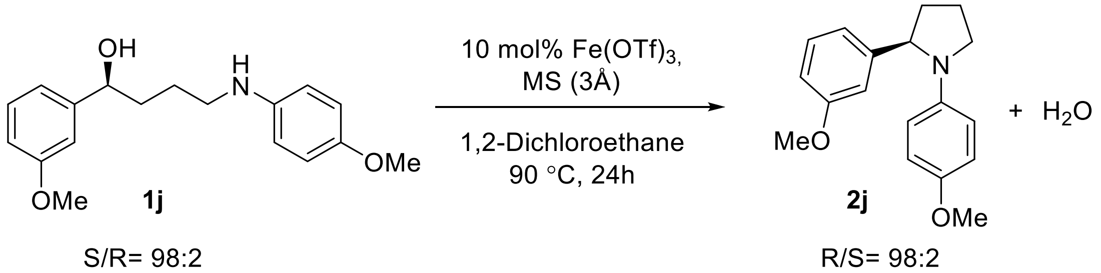

Alcohol **1j** (150.5 mg, 0.5 mmol), MS (3Å) (300 mg), and the catalyst $Fe(OTf)_3$ (25.05 mg, 0.05 mmol) were treated as described for **1a** for 24 h and purified as described for **1a** to obtain **2j** (119.4 mg, 0.421 mmol, 88% yield) as a colorless oil. **IR** (neat) 3068, 3050, 2911, 2821, 1609, 1530, 1491, 1453, 1372, 1273, 1231.42, 1137, 1166.32, 1064.43, 961, 816.43, 771.49, 707.13, 521.93 cm^{-1}. **^{1}H NMR** (400 MHz, Chloroform-*d*) δ = 7.22 (t, *J* = 7.8 Hz, 1H), 6.94 – 6.64 (m, 5H), 6.44 (d, *J* = 8.5 Hz, 2H), 4.65 – 4.50 (m, 1H), 3.77 (s, 3H), 3.71 (s, 3H), 3.67 (d, *J* = 10.1 Hz, 1H), 3.34 (q, *J* = 8.3 Hz, 1H), 2.37 (tt, *J* = 11.5, 7.8 Hz, 1H), 2.12 – 1.80 (m, 3H). **^{13}C NMR** (101 MHz, $CDCl_3$) δ = 159.9, 150.9, 147.2, 142.2, 129.5, 118.3, 114.8, 113.0, 111.9, 111.6, 63.5, 55.9, 55.1, 49.7, 36.2, 23.4 ppm. **HRMS** (**ESI**) calcd. for $C_{18}H_{22}NO_2$ [M+H] *m/z* 284.1649 found *m/z* 284.1645. The enantiomeric ratio of **2j** was determined by HPLC analysis

using Daicel Chiralcel OJ-H column: *n*-Hexane : isopropanol = 95:05, flow rate 0.5 mL/min, λ = 254 nm (channel 1), 232 nm (channel 2): t_1 (minor) = 24.8 min, t_2 (major) = 27.4 min.

(*S*)-1-(4-methoxyphenyl)-2-vinylpyrrolidine (2k)[11]

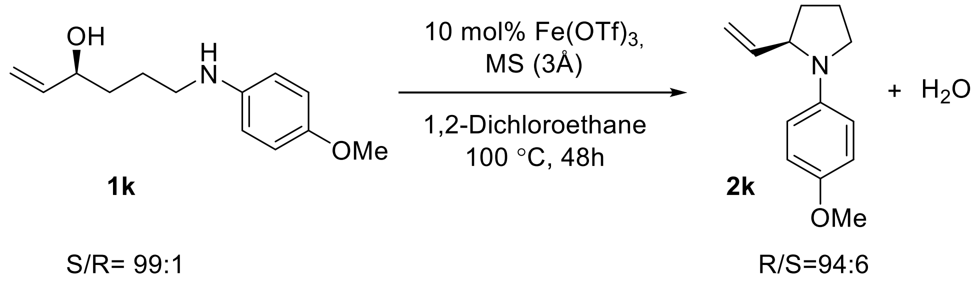

Alcohol **1k** (110.5 mg, 0.5 mmol), MS (3Å) (300 mg), and the catalyst $Fe(OTf)_3$ (25.05 mg, 0.05 mmol) were treated as described for **1a** at 100 °C for 48 h. After completion of reaction (TLC), the crude was concentrated under vacuum and purified by the column chromatographic using silica gel (mess 100-200) to obtain pure **2k** (89 mg, 0.438 mmol, 88% yield) as colorless oil. **IR** (neat) cm^{-1} 3040, 2955.9, 2890, 2875, 2821, 1616.5, 1575.6, 1513, 1460, 1365, 1274, 1241.5, 1181, 1166, 1045, 970, 820, 591 cm^{-1}. **^{1}H NMR** (400 MHz, Chloroform-*d*) δ = 6.87 – 6.81 (m, 2H), 6.61 – 6.55 (m, 2H), 5.84 (ddd, *J* = 17.1, 10.2, 5.4 Hz, 1H), 5.19 – 5.08 (m, 2H), 4.10 (dddd, *J* = 6.7, 5.4, 2.6, 1.3 Hz, 1H), 3.77 (s, 3H), 3.49 (ddd, *J* = 8.4, 7.0, 2.8 Hz, 1H), 3.22 (td, *J* = 8.7, 6.9 Hz, 1H), 2.21 – 1.91 (m, 4H), 1.83 (ddt, *J* = 8.7, 6.1, 3.0 Hz, 1H) ppm. **^{13}C NMR** (100 MHz, $CDCl_3$) δ = 150.8, 142.5, 139.9, 114.8, 114.3, 112.9, 61.4, 55.9, 49.2, 32.7, 23.3 ppm. **HRMS** (**ESI**) calcd. for $C_{13}H_{18}NO$ [M+H] 204.1383 *m/z* found 204.1386 *m/z*. The enantiomeric ratio of **2k** was determined by HPLC analysis using Daicel Chiralcel OJ-H column: *n*-Hexane: isopropanol = 99.5:0.5, flow rate 0.5 mL/min, λ = 254 nm (channel 1): t_1 (major) = 60.5 min, t_2 (minor) = 63.1 min.

(*R*)-2-(phenylethynyl)tetrahydrothiophene (2l)

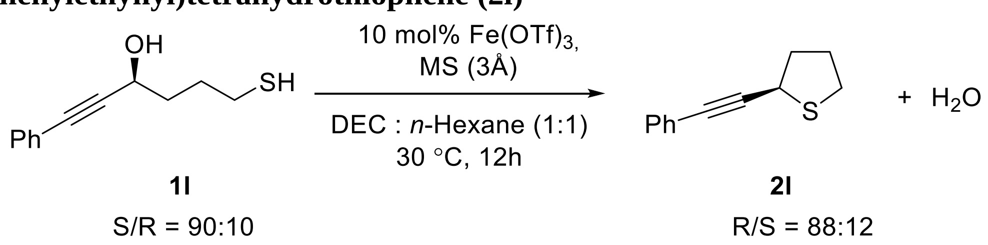

Alcohol **1l** (103 mg, 0.5 mmol), MS (3Å) (300 mg), and the catalyst $Fe(OTf)_3$ (25.05 mg, 0.05 mmol) were treated as described for **1a** for 24 h at room temperature and purified as described for **1a** to obtain **2l** (85.5 mg, 0.454 mmol, 91% yield) as a yellow color oil. **^{1}H NMR** (400 MHz, Chloroform-*d*) δ = 7.43 – 7.37 (m, 2H), 7.30 – 7.25 (m, 3H), 4.27 (t, *J* = 5.8 Hz, 1H), 3.17 – 3.07 (m, 1H), 2.94 (dt, *J* = 10.2, 6.6 Hz, 1H), 2.30 – 2.14 (m, 3H), 2.08 (dd, *J* = 6.6, 6.1 Hz, 1H) ppm. **^{13}C NMR** (101 MHz, $CDCl_3$) δ = 131.6, 128.1, 128.0, 123.2, 90.6, 82.8, 38.9, 36.9, 32.9, 30.5 ppm. The enantiomeric ratio of **2l** was determined by HPLC analysis using Daicel Chiralcel OD-H column: *n*-Hexane: isopropanol = 99.5:0.5, flow rate 0.5 mL/min, λ = 254 nm (channel 1): t_1 (major) = 16.2 min, t_2 (minor) = 22.3 min. $[\alpha]_D^{28}$ +6.0, (*c* 0.3, $CHCl_3$)

(*S*)-1-(4-methoxyphenyl)-2-methylpyrrolidine (2m)

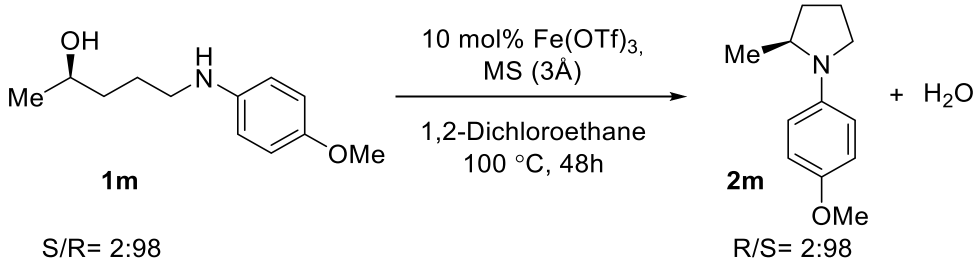

Alcohol **1m** (104.5 mg, 0.5 mmol), MS (3Å) (300 mg), and the catalyst $Fe(OTf)_3$ (25.05 mg, 0.05 mmol) were treated as described for **1a** at 100 °C for 48 h. After completion of reaction (TLC), the crude was concentrated under vacuum and purified by the column chromatographic using silica gel (mess 100-200) to obtain pure **2m** (76.4 mg, 0.4 mmol, 80% yield) as colorless oil. **IR** (neat) cm^{-1} 3045, 2961, 2930, 2874, 2824, 1616, 1575, 1464, 1329, 1275, 1245, 1181, 1164, 1041, 970, 811.6, 591 cm^{-1}. **^{1}H NMR** (400 MHz, Chloroform-*d*) δ = 6.97 – 6.87 (m, 2H), 6.68 – 6.56 (m, 2H), 3.85 (td, *J* = 6.5, 2.2 Hz, 1H), 3.82 (s, 3H), 3.52 – 3.43 (m, 1H), 3.17 (td, *J* = 8.8, 7.0 Hz, 1H), 2.21 – 1.91 (m, 3H), 1.75 (dp, *J* = 5.3, 2.6, 2.2 Hz, 1H), 1.23 (d, *J* = 6.2 Hz, 3H) ppm. **^{13}C NMR** (100 MHz, $CDCl_3$) δ = 150.6, 142.2, 114.9, 112.7, 55.9, 54.0, 48.9, 33.1, 23.3, 19.5 ppm. The enantiomeric ratio of **2m** was determined by HPLC analysis using Daicel Chiralcel OJ-H column: *n*-Hexane : isopropanol = 99.5:0.5, flow rate 0.5 mL/min, λ = 254 nm (channel 1): t_1 (minor) = 30.0 min, t_2 (major) = 60.6 min.

(*R*)-2-ethyl-1-(4-methoxyphenyl)pyrrolidine (2n)

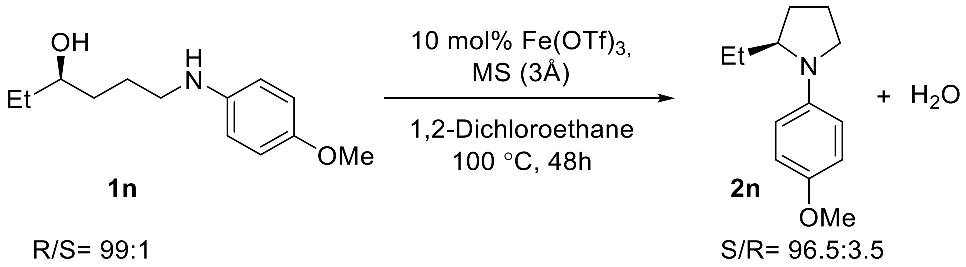

Alcohol **1n** (111.5 mg, 0.5 mmol), MS (3Å) (300 mg), and the catalyst Fe(OTf)3 (25.05 mg, 0.05 mmol) were treated as described for **1a** at 100 °C for 48 h. After completion of reaction (TLC), the crude was concentrated under vacuum and purified by the column chromatographic using silica gel (mess 100-200) to obtain pure **2n** (85.07 mg, 0.419 mmol, 83% yield) as colorless oil. **IR** (neat) cm^{-1} 3044, 2960.59, 2931, 2873, 2829, 1619.75, 1574.68, 1512.9, 1464, 1363, 1327, 1274, 1240.55, 1180, 1163, 1044, 969, 810.9, 590, 525 cm^{-1}. **^{1}H NMR** (400 MHz, Chloroform-*d*) δ = 7.01 – 6.93 (m, 2H), 6.69 – 6.60 (m, 2H), 3.87 (s, 3H), 3.62 (tt, *J* = 7.2, 2.6 Hz, 1H), 3.57 – 3.48 (m, 1H), 3.25 – 3.14 (m, 1H), 2.20 – 1.99 (m, 3H), 1.99 – 1.81 (m, 2H), 1.50 – 1.33 (m, 1H), 1.06 (t, *J* = 7.5 Hz, 3H) ppm. **^{13}C NMR** (100 MHz, $CDCl_3$) δ = 150.5, 142.4, 114.9, 112.5, 60.4, 55.7, 49.0, 29.8, 26.0, 23.5, 10.5 ppm. **HRMS** (**ESI**) calcd. For $C_{13}H_{20}NO$ [M+H] 206.1539 *m/z* found 206.1546 *m/z*. The enantiomeric ratio of **2n** was determined by HPLC analysis using Daicel Chiralcel OJ-H column: *n*-Hexane : isopropanol = 99.5:0.5, flow rate 0.5 mL/min, λ = 254 nm (channel 1): t_1 (minor) = 25.8 min, t_2 (minor) = 63.8 min. $[\alpha]_D^{28}$ -3.0, (*c* 0.1, $CHCl_3$)

(*R*)-1-(4-methoxyphenyl)-2-phenylpiperidine (2o)

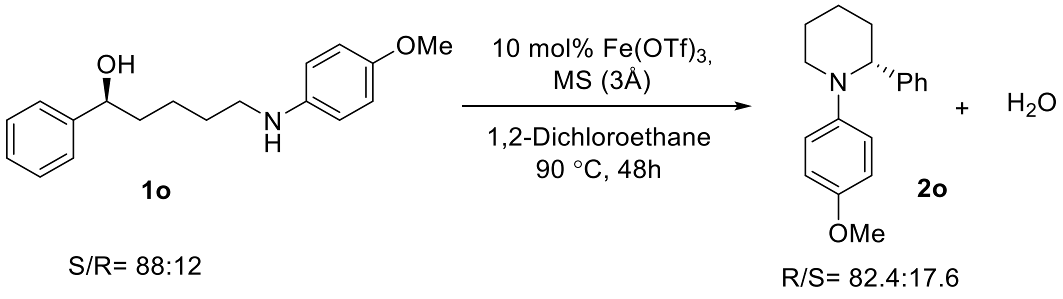

Alcohol **1o** (142.58 mg, 0.5 mmol), MS (3Å) (300 mg), and the catalyst $Fe(OTf)_3$ (25.05 mg, 0.05 mmol) were treated as described for **1a** for 24 h and purified as described for **1a** to obtain **2o** (116.1 mg, 0.434 mmol, 87% yield) as a colorless oil. **IR** (neat) 3060, 3044, 2956, 2915, 2830, 1611, 1512, 1491, 1451, 1360, 1261, 1245, 1180, 1171, 961.56, 811.75, 771.69, 748.15, 590.1, 521.25 cm^{-1}. **^{1}H NMR** (400 MHz, Chloroform-*d*) δ = 7.19 – 7.14 (m, 2H), 7.13 – 7.06 (m, 2H), 7.03 – 6.96 (m, 1H), 6.87 – 6.76 (m, 2H), 6.63 – 6.52 (m, 2H), 3.96 (dd, *J* = 9.5, 3.3 Hz, 1H), 3.61 (s, 3H), 3.35 – 3.25 (m, 1H), 2.81 (ddd, *J* = 12.0, 10.1, 3.5 Hz, 1H), 1.90 – 1.81 (m, 1H), 1.72 (dddd, *J* = 15.0, 13.4, 10.5, 6.4 Hz, 4H), 1.47 – 1.38 (m, 1H) ppm. **^{13}C NMR** (101 MHz, $CDCl_3$) δ = 144.7, 128.0, 127.4, 126.1, 123.8, 113.8, 100.0, 92.9, 77.3, 77.0, 76.7, 64.5, 56.4, 55.3, 36.1, 26.5, 24.2 ppm. **HRMS** (**ESI**) calcd. for $C_{18}H_{22}NO$ [M+H] *m/z* 268.1702 found *m/z* 268.1696. The enantiomeric ratio of **2o** was determined by HPLC analysis using Daicel Chiralcel OJ-H column: *n*-Hexane : isopropanol = 95:05, flow rate 0.5 mL/min, λ = 254 nm (channel 1), 232 nm (channel 2): t_1 (minor) = 6.6 min, t_2 (major) = 9.5 min. $[\alpha]_D^{28}$ -6.0, (*c* 0.1, $CHCl_3$)

(*R*)-1-(4-methoxyphenyl)-2-phenyl-1,2,3,4-tetrahydroquinoline (**2p**)

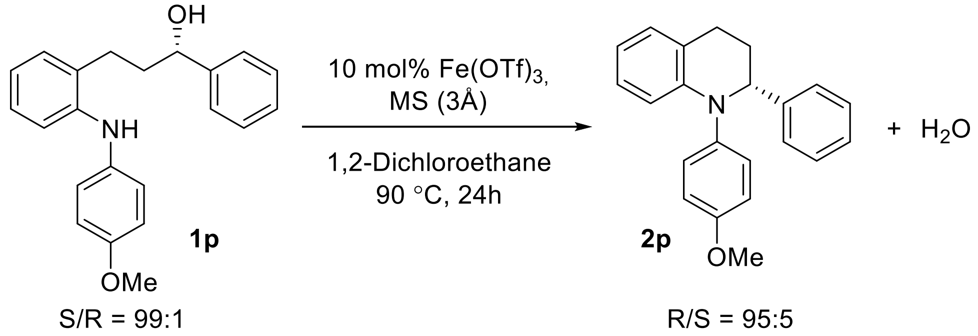

Alcohol **1p** (166.58 mg, 0.5 mmol), MS (3Å) (300 mg), and the catalyst $Fe(OTf)_3$ (25.05 mg, 0.05 mmol) were treated as described for **1a** for 24 h and purified as described for **1a** to obtain **2p** (157.5 mg, 0.5 mmol, 100% yield) as a colorless oil. **IR** (neat) 3013, 2931, 2852, 1602, 1508, 1451, 1361, 1289.97, 1238.08, 1211, 1179, 1101, 933.6, 827.75, 756.69, 700.08, 552.2 cm^{-1}. **^{1}H NMR** (400 MHz, Chloroform-*d*) δ = 7.21 (d, *J* = 4.4 Hz, 4H), 7.15 (dd, *J* = 4.8, 3.7

Hz, 1H), 7.07 – 7.01 (m, 2H), 6.99 – 6.94 (m, 1H), 6.93 – 6.85 (m, 1H), 6.78 – 6.70 (m, 2H), 6.60 (td, *J* = 7.3, 1.2 Hz, 1H), 6.52 (dd, *J* = 8.3, 1.1 Hz, 1H), 4.78 (d, *J* = 4.3 Hz, 1H), 3.71 (s, 3H), 2.71 – 2.52 (m, 2H), 2.25 (ddt, *J* = 12.9, 11.4, 4.9 Hz, 1H), 2.14 – 2.02 (m, 1H) ppm. **^{13}C NMR** (101 MHz, $CDCl_3$) δ = 156.8, 145.2, 144.2, 140.5, 129.2, 128.3, 128.2, 126.7, 126.7, 126.7, 122.4, 116.9, 114.7, 114.1, 63.7, 55.4, 28.9, 23.7 ppm. **HRMS** (**ESI**) calcd. for $C_{22}H_{21}NNaO$ [M+Na] *m/z* 338.1508 found *m/z* 338.1515. The enantiomeric ratio of **2p** was determined by HPLC analysis using Daicel Chiralcel OJ-H column: *n*-Hexane : isopropanol = 96:4, flow rate 1.0 mL/min, λ = 254 nm (channel 1), 232 nm (channel 2): t_1 (minor) = 10. min, t_2 (major) = 11 min.

(*S*)-2-phenylchromane (**2q**)

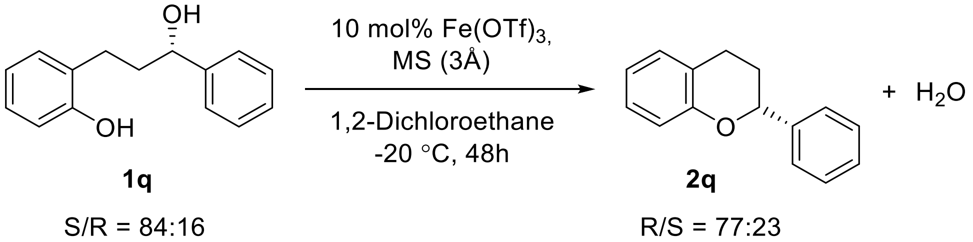

Alcohol **1q** (114 mg, 0.5 mmol), MS (3Å) (300 mg), and the catalyst $Fe(OTf)_3$ (25.05 mg, 0.05 mmol) were treated as described for **1a** at -20 °C for 48 h. After completion of reaction (TLC), the crude was concentrated under vacuum and purified by the column chromatographic using silica gel (mess 100-200) to obtain pure **2q** (95 mg, 0.454 mmol, 91% yield) as colorless oil. **^{1}H NMR** (400 MHz, Chloroform-*d*) δ = 7.50 – 7.38 (m, 4H), 7.38 – 7.31 (m, 1H), 7.21 – 7.08 (m, 2H), 6.99–6.85 (m, 2H), 5.10 (dd, *J* = 10.1, 2.5 Hz, 1H), 3.03 (dddd, *J* = 17.3, 11.2, 6.0, 1.1 Hz, 1H), 2.83 (ddd, *J* = 16.5, 5.3, 3.4 Hz, 1H), 2.25 (dddd, *J* = 13.7, 5.9, 3.3, 2.5 Hz, 1H), 2.13 (dddd, *J* = 13.7, 11.3, 10.1, 5.3 Hz, 1H) ppm. **^{13}C NMR** (100 MHz, $CDCl_3$) δ = 155.1, 141.7, 129.5, 128.5, 127.8, 127.3, 125.9, 121.8, 120.3, 116.9, 76.7, 29.9, 25.1 ppm. The enantiomeric ratio of **2q** was determined by HPLC analysis using Daicel Chiralcel JM column: *n*-Hexane: isopropanol = 98:2, flow rate 0.5 mL/min, λ = 254 nm (channel 1): t_1 (major) = 8.9 min, t_2 (minor) = 14.2 min. $[\alpha]_D^{28}$ +6.0, (*c* 0.1, $CHCl_3$)

(*S*)-2-(4-fluorophenyl)chromane (2r)

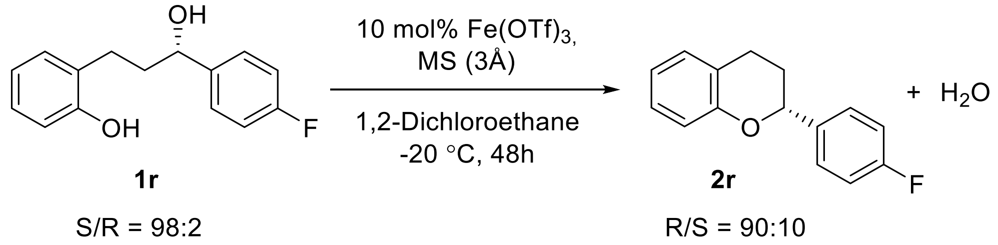

Alcohol **1r** (123 mg, 0.5 mmol), MS (3Å) (300 mg), and the catalyst $Fe(OTf)_3$ (25.05 mg, 0.05 mmol) were treated as described for **1a** at -20 °C for 48 h. After completion of reaction (TLC), the crude was concentrated under vacuum and purified by the column chromatographic using silica gel (mess 100-200) to obtain pure **2r** (92 mg, 0.419 mmol, 81% yield) as colorless oil. **^{1}H NMR** (400 MHz, Chloroform-*d*) δ = 7.47 – 7.39 (m, 2H), 7.21 – 7.06 (m, 4H), 6.97 – 6.87 (m, 2H), 5.08 (dd, *J* = 10.2, 2.5 Hz, 1H), 3.15 – 2.94 (m, 1H), 2.84 (ddd, *J* = 16.5, 5.3, 3.2 Hz, 1H), 2.23 (dddd, *J* = 13.7, 5.8, 3.2, 2.4 Hz, 1H), 2.10 (dddd, *J* = 13.7, 11.3, 10.2, 5.3 Hz, 1H) ppm. **^{13}C NMR** (100 MHz, $CDCl_3$) δ = 162.4 (d, $^1J_{C\text{-}F}$ = 245.8 Hz), 154.9, 137.5 (d, $^4J_{C\text{-}F}$ = 3.3 Hz), 129.5, 127.7 (d, $^3J_{C\text{-}F}$ = 8.1 Hz), 127.4, 121.7, 120.4, 116.9, 115.4 (d, $^1J_{C\text{-}F}$ = 21.4 Hz), 77.1, 30.0, 25.0 ppm. The enantiomeric ratio of **2r** was determined by HPLC analysis using Daicel Chiralcel JM-column: *n*-Hexane : isopropanol = 95:5, flow rate 1 mL/min, λ = 254 nm (channel 1): t_1 (major) = 6.5 min, t_2 (minor) = 9.7 min. $[\alpha]_D^{28}$ +4.0, (*c* 0.2, $CHCl_3$)

(*R*)-2-methyl-1,2-diphenyl-1,2,3,4-tetrahydroquinoline (2s)

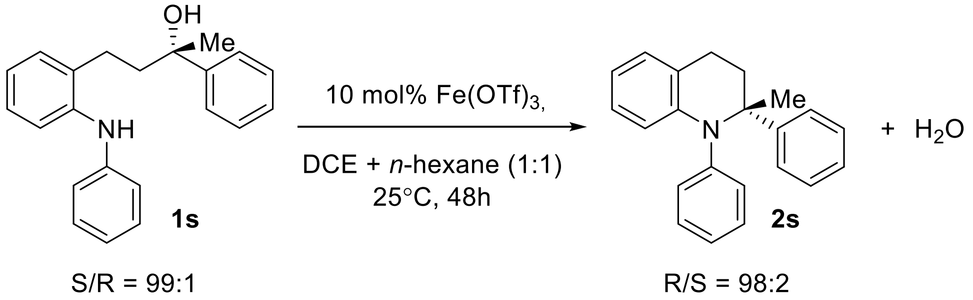

Alcohol **1s** (158.5 mg, 0.5 mmol) and the catalyst $Fe(OTf)_3$ (25.05 mg, 0.05 mmol) in DCE + *n*-Hexane (1:1) were treated as described for **1a** at room temperature for 48 h and purified as described for **1s** to obtain **2s** (146.51 mg, 0.5 mmol, 98% yield) as a white color solid. **IR** (neat) 3028, 3057.11, 29.79.30, 2935.16, 2845, 1602, 1591, 1575.59, 1492, 1455.32, 1378, 1319,

1236.83, 1156, 1132, 1072, 1026, 1003, 933.49, 841, 763, 746, 699.48 cm^{-1}. **^{1}H NMR** (400 MHz, Chloroform-*d*) δ = 7.47 – 7.36 (m, 2H), 7.29 (tt, *J* = 4.0, 3.3 Hz, 4H), 7.24 – 7.18 (m, 2H), 7.18 – 7.13 (m, 2H), 6.98 – 6.84 (m, 2H), 6.61 (td, *J* = 7.3, 1.1 Hz, 1H), 6.33 (dd, *J* = 8.3, 1.2 Hz, 1H), 2.71 (dt, *J* = 16.3, 4.2 Hz, 1H), 2.40 (ddd, *J* = 16.7, 12.3, 5.4 Hz, 1H), 2.25 (ddd, *J* = 12.8, 5.3, 3.6 Hz, 1H), 2.15 (td, *J* = 12.6, 4.8 Hz, 1H), 1.44 (s, 3H) ppm. **^{13}C NMR** (101 MHz, $CDCl_3$) δ = 147.6, 146.3, 144.3, 131.0, 129.2, 129.1, 128.2, 126.5, 126.5, 126.3, 126.0, 121.7, 116.4, 114.7, 61.3, 37.8, 29.4, 24.8 ppm. **HRMS** (**ESI**) calcd. for $C_{22}H_{21}NNa$ [M+Na] *m/z* 322.1570 found *m/z* 322.1574. The enantiomeric ratio of **2s** was determined by HPLC analysis using Daicel Chiralcel OD-H column: *n*-Hexane : isopropanol = 99:1, flow rate 1.0 mL/min, λ = 254 nm (channel 1), 232 nm (channel 2): t_1 (major) = 4.7 min, t_2 (minor) = 5.6 min. $[\alpha]_D^{28}$ -20.0, (*c* 0.2, $CHCl_3$)

(*R*)-2-methyl-2-phenylchromane (2t)

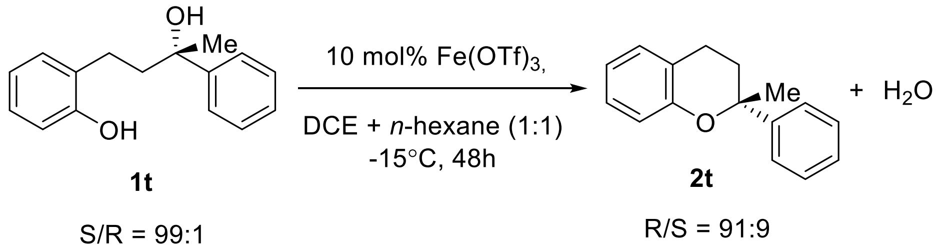

Alcohol **2t** (121.5 mg, 0.5 mmol) and the catalyst $Fe(OTf)_3$ (25.05 mg, 0.05 mmol) in DCE + *n*-Hexane (1:1) were treated as described for **1a** at -15 °C temperature for 48 h and purified as described for **1a** to obtain **2t** (122 mg, 0.5 mmol, 100% yield) as a white color solid. **IR** (neat) 3060, 3024.55, 2977.79, 2929, 2849, 16010.53, 1582, 1522.56, 1488, 1456, 1446, 1373, 1340.54, 1305.83, 1243.57, 1167, 1119, 1069, 1029, 971, 945.49, 826, 753, 699.68, 548 cm^{-1}. **^{1}H NMR** (400 MHz, Chloroform-*d*) δ = 7.38 (dd, *J* = 8.4, 1.3 Hz, 2H), 7.30 (ddd, *J* = 7.8, 6.8, 1.2 Hz, 2H), 7.24 – 7.18 (m, 1H), 7.17 – 7.10 (m, 1H), 7.04 – 6.91 (m, 2H), 6.81 (td, *J* = 7.4, 1.3 Hz, 1H), 2.67 (dt, *J* = 16.1, 4.9 Hz, 1H), 2.51 – 2.34 (m, 2H), 2.09 (ddd, *J* = 13.7, 10.5, 5.4 Hz, 1H), 1.66 (s, 3H) ppm. **^{13}C NMR** (101 MHz, $CDCl_3$) δ = 154.1, 145.6, 129.4, 128.3, 127.3, 126.7, 124.9, 121.6, 119.9, 116.9, 78.3, 33.0, 30.1, 22.6 ppm. **HRMS** (**ESI**) calcd. for

$C_{16}H_{16}NNa$ [M+Na] *m/z* 247.1231 found *m/z* 322.1225. The enantiomeric ratio of **2t** was determined by HPLC analysis using Daicel Chiralcel OD-H column: *n*-Hexane : isopropanol = 99:1, flow rate 0.5 mL/min, λ = 254 nm (channel 1), 232 nm (channel 2): t_1 (major) = 20.1 min, t_2 (minor) = 22.2 min. $[\alpha]_D^{28}$ -5.0, (*c* 0.18, $CHCl_3$)

(*S*)-2-methyl-2-(4-methylpentyl)chromane (**2u**)

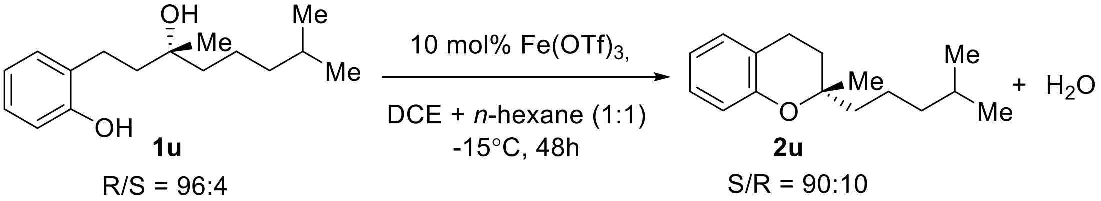

Alcohol **1u** (125.15 mg, 0.5 mmol) and the catalyst $Fe(OTf)_3$ (25.05 mg, 0.05 mmol) in DCE + *n*-Hexane (1:1) were treated as described for **1a** at -15 °C temperature for 48 h and purified as described for **1a** to obtain **2u** (106 mg, 0.456 mmol, 92% yield) as a colorless oil. IR (neat) 3374, 3041, 2928, 2865, 1603, 1515, 1430, 1319, 1254, 1214, 1170, 1155, 1063, 1023, 824, 693 cm^{-1}. **^{1}H NMR** (400 MHz, Chloroform-*d*) δ 7.12 – 7.02 (m, 2H), 6.84 – 6.75 (m, 2H), 2.75 (t, *J* = 6.8 Hz, 2H), 1.86 – 1.72 (m, 2H), 1.60 – 1.50 (m, 2H), 1.37 (ddd, *J* = 14.4, 9.5, 7.6 Hz, 2H), 1.28 (s, 3H), 1.24 – 1.08 (m, 3H), 0.86 (d, *J* = 6.6 Hz, 6H). **^{13}C NMR** (101 MHz, $CDCl_3$) δ = 153.9, 129.4, 127.2, 121.1, 119.4, 117.2, 76.2, 39.8, 39.3, 30.6, 27.9, 24.2, 22.6, 22.6, 22.07, 21.4 ppm. **HRMS** (**ESI**) calcd. for $C_{16}H_{24}ONa$ [M+Na] *m/z* 255.1719 found *m/z* 255.1713. The enantiomeric ratio of **2u** was determined by HPLC analysis using Daicel Chiralcel OD-H column: *n*-Hexane : isopropanol = 99:1, flow rate 1.0 mL/min, λ = 254 nm (channel 1), 232 nm (channel 2): t_1 (major) = 6.3 min, t_2 (minor) = 6.9 min.

(*R*)-2-methyl-2-(4-methylpentyl)chromane (**2v**)

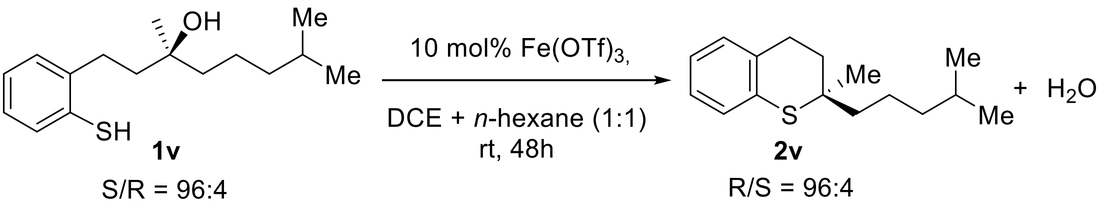

Alcohol **2v** (26.6 mg, 0.1 mmol) and the catalyst $Fe(OTf)_3$ (5.05 mg, 0.01 mmol) in DCE + *n*-Hexane (0.5:0.5) were treated as described for **1a** at room temperature for 48 h and purified as described for **1a** to obtain **2v** (22.8 mg, 0.092 mmol, 92% yield) as a colorless oil. **^{1}H NMR** (400 MHz, Chloroform-*d*) = δ 7.19 – 7.05 (m, 3H), 7.00 (ddd, *J* = 7.3, 6.3, 2.5 Hz, 1H), 2.94 – 2.84 (m, 2H), 2.00 – 1.87 (m, 2H), 1.65 – 1.53 (m, 3H), 1.50 – 1.41 (m, 2H), 1.40 (s, 3H), 1.25 – 1.14 (m, 2H), 0.90 (d, *J* = 6.6 Hz, 3H) ppm. ^{13}C NMR (101 MHz, $CDCl_3$) δ = 133.8, 132.8, 129.6, 126.9, 126.3, 123.8, 46.0, 42.9, 39.4, 36.0, 27.9, 27.3, 26.8, 22.6, 21.8 ppm. **HRMS** (**ESI**) calcd. for $C_{16}H_{24}SNa$ [M+Na] *m/z* 271.4123 found *m/z* 271.4115. The enantiomeric ratio of **2v** was determined by HPLC analysis using Daicel Chiralcel OJ-H column: *n*-Hexane : isopropanol = 99.5:0.5, flow rate 0.5 mL/min, λ = 254 nm (channel 1), 232 nm (channel 2): t_1 (major) = 12.4 min, t_2 (minor) = 13.7 min. $[\alpha]_D^{28}$ +2.0, (*c* 0.1, $CHCl_3$).

Total Synthesis of (+)-Lentiginosine (8):

Step-I: Synthesis of intermediate 4

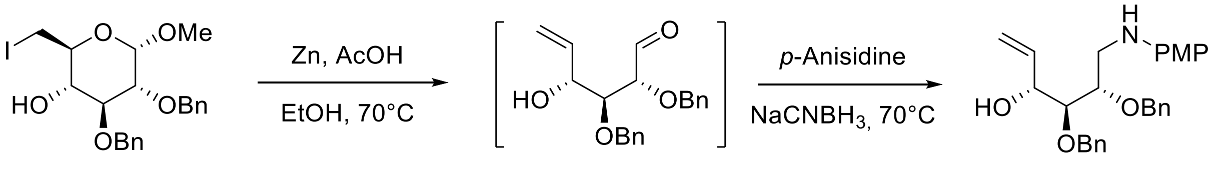

3 **4**

2,3-di-*O*-benzyl-6-deoxy-6-iodo-α-D-methyl-glucopyranose (3)[13]

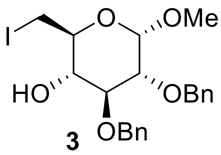

3

6-Iodo glucopyronoside **3** was prepared according to the literature report[13].

$[\alpha]_D^{28}$ +45.0, (*c* 0.4, CH_2Cl_2), lit, $[\alpha]_D^{26}$ +50.6, (*0.02615*, CH_2Cl_2)

IR (neat) 3468, 3030, 2912, 1726, 1602, 1496, 1454, 1367, 1277, 1198, 1096, 1066, 738 cm^{-1}; **^{1}H NMR** (400 MHz, $CDCl_3$) δ = 7.48-7.28 (m, 10H), 5.05 (d, *J*= 11.5 Hz, 1H), 4.80 (d, *J*= 12.1 Hz, 1H), 4.73-4.64 (m, 3H), 3.80 (t, *J*= 9.1 Hz, 1H), 3.59-3.52 (m, 2H), 3.46 (s, 3H), 3.48-3.40 (m, 1H), 3.37-3.24 (m, 2H), 2.19 (s, 1H) ppm; **^{13}C NMR** (101 MHz, $CDCl_3$) δ = 138.5, 137.9, 128.7, 128.6, 128.1 (3), 128.0, 98.1, 80.7, 79.9, 75.4, 73.7, 69.8, 55.6, 7.0 ppm; **HRMS (ESI)** Calcd. for, $C_{21}H_{25}INaO_5$ $[M+Na]^+$: 507.0639 Found 507.0649.

To a mixture of 6-Iodo glucopyranoside[13] (1.53 g, 5.05 mmol) **3** and activated zinc (1.65 g, 25.25 mmol) in ethanol (40 mL), AcOH (0.1 mL) was added. The reaction mixture was stirred at 70°C for 30 min then a solution of *p*-anisidine (1.24 g, 10.1 mmol) in ethanol (10 mL) and a solution of $NaCNBH_3$ (635 mg, 10.1 mmol) in ethanol (10 mL) were added. The reaction mixture was stirred overnight at 70°C. After the reaction was completed, it was cooled to room temperature and quenched with saturated aq.NaCl and extracted with EtOAc (3x100 mL). The combined organic layers were dried over anhydrous Na_2SO_4, filtered and concentrated under

reduced pressure. The crude amino alcohol was purified by column chromatography on silica gel eluting with pentane: EtOAc (4:1) to give the desired product **4** as a brown solid (1.27 g, 2.94 mmol, 58 % yield). Mp = 75-76 °C; $[\alpha]_D^{28}$ +4.4, (*c* 0.09, CH_2Cl_2); **IR** (neat) = 3398, 3004, 2905, 1513, 1454, 1235, 1089, 1073, 1028 cm^{-1}; **^{1}H NMR** (400 MHz, $CDCl_3$) δ = 7.44-7.30 (m, 10H), 6.81-6.76 (m, 2H), 6.62-6.56 (m, 2H), 5.97 (ddd, *J*= 5.13, 10.5, 17.2Hz, 1H), 5.39 (dt, *J*= 18.2, 1.64 Hz, 1H), 5.21 (dt, *J*= 10.5, 1.6 Hz, 1H), 4.72 (d, *J*= 11.3 Hz, 1H), 4.67(d, *J*= 11.3 Hz, 1H), 4.64 (d, *J*= 11.6 Hz, 1H), 4.59 (d, *J*= 11.6 Hz, 1H), 4.41-4.35 (m, 1H), 3.86 (dd, *J*= 5.1, 10.6 Hz, 1H), 3.77 (s, 3H), 3.64 (dd, *J*= 3.0, 5.7 Hz, 1H), 3.39-3.29 (m, 2H) ppm; **^{13}C NMR** (101 MHz, $CDCl_3$) δ = 152.7, 141.9, 138.5, 138.0 (2), 128.6, 128.5, 128.3, 128.0 (2), 115.6, 115.1, 114.9, 81.3, 78.0, 74.6, 72.8, 71.3, 55.8, 44.7 ppm; **HRMS (ESI)** Calcd. for, $C_{27}H_{31}NNaO_4$ $[M+Na]^+$: 456.2145 Found 456.2144.

Step-II: (2S,3S,4S)-3,4-bis(benzyloxy)-1-(4-methoxyphenyl)-2-vinylpyrrolidine (5)

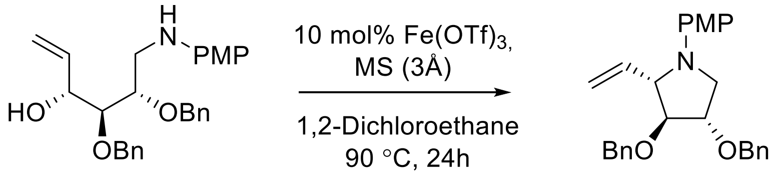

4 **5**

Alcohol **4** (433 mg, 1 mmol), MS (3Å) (500 mg), and the catalyst $Fe(OTf)_3$ (50.5 mg, 0.1 mmol) were treated as described for **1a** at 90 °C for 24 h. After completion of reaction (TLC), the crude was concentrated under vacuum and purified by the column chromatographic using silica gel (mess 100-200) to obtain pure **7** (410 mg, 0.99 mmol, 99% yield) as colorless oil. $[\alpha]_D^{28}$ +15.3, (*c* 0.326, CH_2Cl_2); **IR** (neat) 3030, 2906, 2859, 1512, 1454, 1349, 1241, 1096, 1041 cm^{-1}; **^{1}H NMR** (400 MHz, $CDCl_3$) δ = 7.44-7.26 (m, 10H), 6.86-6.80 (m, 2H), 6.65-6.58 (m, 2H), 5.92 (ddd, *J*= 7.3, 10.2, 17.4 Hz, 1H), 5.35 (d, *J*= 17.4 Hz, 1H), 5.21 (d, *J*= 10.2 Hz, 1H), 4.68 (d, *J*= 12.0 Hz, 1H), 4.62 (d, *J*= 12.0 Hz, 1H), 4.59 (s, 2H), 4.20-4.16 (m, 1H), 4.13 (d, *J*= 7.2 Hz, 1H), 4.03 (t, *J*= 2.2 Hz, 1H), 3.77 (s, 3H), 3.66-3.56 (m, 2H) ppm; **^{13}C NMR** (101 MHz, $CDCl_3$) δ = 151.4, 143.8, 138.5, 137.9 (2), 128.4, 127.8, 127.7 (2), 127.6, 116.3, 114.7, 113.6, 87.2,

80.9, 71.7, 71.4, 67.8, 55.9, 53.3 ppm; **HRMS (ESI)** Calcd. for, $C_{27}H_{30}NO_3$ $[M+H]^+$: 416.2220 Found 416.2215.

Step-III: (*2S,3S,4S*)-3,4-Bis(benzyloxy)-2-vinylpyrrolidine (6)

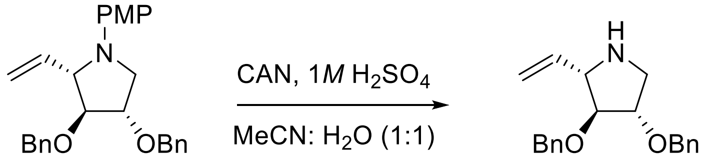

5 **6**

To a solution of PMP protected pyrrolidine **5** (415 mg, 1 mmol) in MeCN: H_2O (1:1) (10 mL), ceric ammoniumnitrate (822 mg, 1.5 mmol) and 1*M* H_2SO_4 (0.8 mL) were added. The reaction mixture was stirred overnight at room temperature. Then the mixture was basify to pH 10 by adding 1*M* NaOH and then extracted in EtOAc (3x10 mL). The combined organic layers were dried over anhydrous Na_2SO_4, filtered and concentrated under reduced pressure. The crude pyrrolidine was purified by column chromatography on silica gel eluting with CH_2Cl_2: MeOH (10:1) to give the desired product as yellow oil **6** (253 mg, 0.82 mmol, 82 % yield). $[\alpha]_D^{28}$ +29.6, (*c* 0.358, CH_2Cl_2), lit $[\alpha]_D^{20}$ +21.6, (*2.0*, $CHCl_3$).[14] **IR** (neat) 3338, 3063, 3030, 3864, 1642, 1496, 1454, 1362, 1206, 1097 cm^{-1}; **^{1}H NMR** (400 MHz, $CDCl_3$) δ = 7.42-7.23 (m, 10H), 6.04-5.89 (m, 1H), 5.32 (d, *J*= 17.1 Hz, 1H), 5.17 (d, *J*= 10.3 Hz, 1H), 4.65-4.45 (m, 4H), 4.04 (br s, 1H), 3.80 (br s, 1H), 3.61 (br s, 1H), 3.23-3.06 (m, 2H), 2.04 (br s, 1H) ppm; **^{13}C NMR** (101 MHz, $CDCl_3$) δ = 138.1 (2), 138.0, 128.4 (2), 116.2, 89.2, 84.5, 72.0, 71.1, 67.2, 51.1 ppm; **HRMS (ESI)** Calcd. for, $C_{20}H_{24}NO_2$ $[M+H]^+$: 310.1802 Found 310.1814.

Step-IV: (*2S,3S,4S*)-3,4-Bis(benzyloxy)-1-(but-3-en-1-yl)-2-vinylpyrrolidine (7)[15]

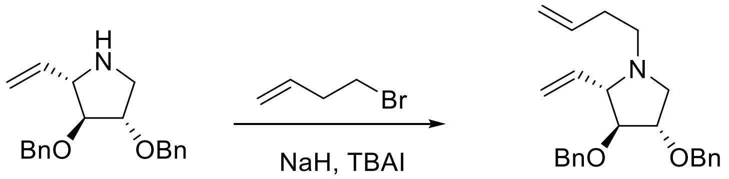

6 **7**

To a mixture of the pyrrolidine **6** (241 mg, 0.78 mmol) and NaH (60% in oil, 93 mg, 2.34 mmol) in DMF (4 mL) at 0°C, 4-bromo-1-butene (0.16 mL, 1.56 mmol) and tetrabutylammonium iodide (57 mg, 0.16 mmol) were added. The reaction mixture was heated and stirred at 70°C for 3h. The reaction mixture was cooled to room temperature, and then it was quenched with aq. $NaHCO_3$ and extracted with CH_2Cl_2 (3x20 mL). The combined organic layer was dried over anhydrous Na_2SO_4, filtered and concentrated under reduced pressure. The crude product was purified by column chromatography on silica gel eluting with pentane: EtOAc (8:1) to give the diene **7** as colorless oil (235 mg, 0.65 mmol, 83% yield). $[\alpha]_D^{28}$ +68.3, (*c* 0.116, CH_2Cl_2); **IR** (neat) 3002, 2904, 2794, 1641, 1496, 1453, 1364, 1098, 1073, 917 cm^{-1}; **^{1}H NMR** (400 MHz, $CDCl_3$) δ = 7.42-7.24 (m, 10H), 5.92-5.74 (m, 2H), 5.34 (dd, *J*= 1.3, 17.2 Hz, 1H), 5.26 (dd, *J*= 1.7, 10.1 Hz, 1H), 5.06 (ddt, *J*= 1.5, 1.9, 15.2 Hz, 1H), 5.00 (ddt, *J*= 1.1, 1.9, 10.2 Hz, 1H), 4.64-4.47 (m, 4H), 4.01-3.95 (m, 1H), 3.87 (dd, *J*= 2.4, 6.7 Hz, 1H), 3.24 (d, *J*= 10.4 Hz, 1H), 2.89-2.71 (m, 2H), 2.49 (dd, *J*= 6.4, 10.4 Hz, 1H), 2.25 (dd, *J*= 7.3, 14.7 Hz, 2H), 2.15-2.05 (m, 1H) ppm; **^{13}C NMR** (101 MHz, $CDCl_3$) δ = 138.7, 138.2, 138.1, 136.6, 128.3(2), 127.8, 127.7, 127.6, 118.8, 115.5, 89.1, 82.0, 74.0, 72.1, 71.2, 57.1, 53.1, 32.1 ppm; **HRMS (ESI)** Calcd. for, $C_{24}H_{30}NO_2$ $[M+H]^+$: 364.2271 Found 364.2268.

Step-V: (*1S,2S,8aS*)-1,2-Bis(benzyloxy)-1,2,3,5,6,8a-hexahydroindolizine[15]

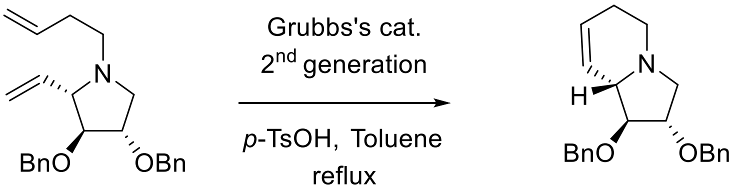

7 **7a**

To a solution of diene **7** (100 mg, 0.26 mmol) in toluene (14 mL, 0.02*M*), *p*-TsOH (105 mg, 0.55 mmol) and Grubbs's catalyst 2nd generation were added. The reaction mixture was heated and stirred at 100°C for 18h. After that it was cooled to room temperature, then neutralized by K_2CO_3, filtered and the filtrate was concentrated under reduced pressure. The residue was

purified by column chromatography on silica gel eluting with pentane: EtOAc: NH_4OH (1:1:0.05) to give the indolizidine **7a** (58 mg, 0.17 mmol, 70% yield). $[\alpha]_D^{28}$ -10.8, (*c* 0.0596, CH_2Cl_2); **IR** (neat): 3030, 2915, 1606, 1496, 1454, 1361, 1095 cm^{-1}; **^{1}H NMR** (400 MHz, $CDCl_3$) δ = 7.40-7.27 (m, 10H), 5.85-5.77 (m, 2H), 4.62 (s, 2H), 4.60 (AB quatet, *J*= 11.7 Hz, 1H), 4.53 (AB quatet, *J*= 11.7 Hz, 1H), 4.15-4.08 (m, 1H), 3.84 (dd, *J*= 3.2, 6.3 Hz, 1H), 3.24 (dt, *J*= 3.1, 6.3 Hz, 1H), 3.07 (dd, *J*= 4.0, 10.2 Hz, 1H), 3.01 (ddd, *J*= 2.7, 6.1, 8.7 Hz, 1H), 2.96 (dd, *J*= 6.4, 10.2 Hz, 1H), 2.72 (ddd, *J*= 4.9, 9.7, 12.0 Hz, 1H), 2.40-2.28 (m, 1H), 2.06-1.93 (m, 1H) ppm; **^{13}C NMR** (101 MHz, $CDCl_3$) δ = 138.2, 128.4, 127.9, 127.8, 127.6, 126.8, 125.9, 88.7, 83.7, 72.0, 71.7, 64.3, 60.0, 46.9, 23.0 ppm; **HRMS (ESI)** Calcd. for, $C_{22}H_{26}NO_2$ $[M+H]^+$: 336.1958 Found 336.1971.

Step-VI: (+)-Lentiginosine 8[15]

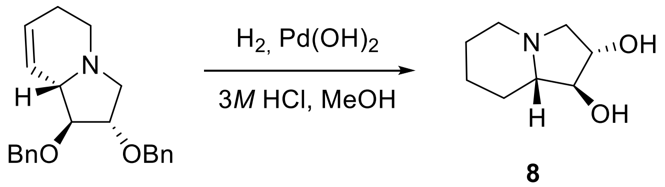

7a

8

(+)-Lentiginosine

To a solution of indolizidine **7a** (26 mg, 0.077 mmol) in methanol (1 mL), Pd/C (20%w/w, 10 mg) and 3*M* HCl (0.5 mL) were added and stirred under atmosphere of hydrogen gas (balloon) for 30 h. The mixture was neutralized by K_2CO_3, filtered and then concentrated under reduced pressure. The residue was purified by column chromatography on silica gel eluting with CH_2Cl_2: MeOH: NH_4OH (10:1:0.1) to give the (+)-Lentiginosine **8** (9 mg, 0.057 mmol, 75% yield) as a white solid. Mp = 138-140. The absolute configuration of (+)-Lentiginosine **8** was confirmed by comparing the optical rotation with the known literature value. $[\alpha]_D^{29}$ +3.07 (*c* 0.065, MeOH).[16] ref= $[\alpha]_D^{24}$ +3.11 (*c* , MeOH) ; **IR** (neat) 3298, 2932, 2853, 2800, 1590, 1443, 1325, 1263, 1141, 1044 cm^{-1}; **^{1}H NMR** (400 MHz, D_2O) δ = 4.03-4.97 (m, 1H), 3.62-3.54 (dd, *J*= 3.9, 8.8 Hz, 1H), 2.88 (d, *J*= 11.1 Hz, 1H), 2.77, d, *J*= 11.2 Hz, 1H), 2.57 (dd, *J*= 7.6, 11.2 Hz, 1H), 1.99 (td, *J*= 2.7, 12.0 Hz, 1H), 1.94-1.80 (m, 2H), 1.80-1.68 (m, 1H), 1.57 (d, *J*= 13.5

Hz, 1H), 1.46-1.30 (m, 1H), 1.26-1.09 (m, 2H) ppm; **^{13}C NMR** (101 MHz, D_2O) δ = 82.8, 75.6, 68.5, 60.1, 52.6, 27.5, 23.9, 23.0 ppm; **HRMS (ESI)** Calcd. for, $C_8H_{16}NO_2$ $[M+H]^+$: 158.1176 Found 158.1168.

Characterization data of synthesized intermediates:

1-(4-methoxyphenyl)piperidin-2-one (U2)

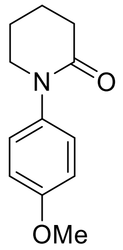

IR (neat) 2953, 2905, 2839, 1683, 1652, 1601, 1510, 1492, 1459, 1360.65, 1331, 1295, 1268, 1247.94, 1223, 1178, 1105, 1031, 830, 755, 730.61, 601, 575, 556.49 cm^{-1}. **^{1}H NMR** (400 MHz, Chloroform-*d*) δ = 7.19 – 7.10 (m, 2H), 6.93 – 6.87 (m, 2H), 3.80 (s, 3H), 3.62 – 3.56 (m, 2H), 2.58 – 2.50 (m, 2H), 1.97 – 1.87 (m, 4H).

(*S*)-1-phenylethan-1-ol (X)

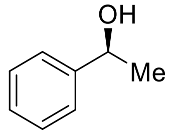

^{1}H NMR (400 MHz, Chloroform-*d*) δ = 7.40 – 7.32 (m, 4H), 7.31 – 7.24 (m, 1H), 4.89 (q, *J* = 6.4 Hz, 1H), 1.50 (d, *J* = 6.5 Hz, 3H) ppm. **^{13}C NMR** (101 MHz, $CDCl_3$) δ = 145.8, 128.5, 127.4, 125.4, 70.4, 25.1 ppm.

(*S*)-1-phenylethyl diisopropylcarbamate (Y)

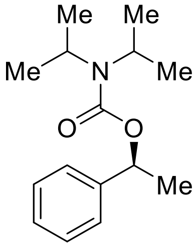

^{1}H NMR (400 MHz, Chloroform-*d*) δ = 7.40 – 7.29 (m, 4H), 7.31 – 7.22 (m, 1H), 5.85 (q, *J* = 6.6 Hz, 1H), 1.55 (d, *J* = 6.6 Hz, 3H), 1.20 (d, *J* = 7.8 Hz, 12H) ppm. **^{13}C NMR** (101 MHz, $CDCl_3$) δ = 155.0, 142.8, 128.4, 127.4, 126.0, 72.7, 46.2 (br), 22.9, 21.3 (br) ppm.

2-phenylbut-3-en-2-ol (Z)

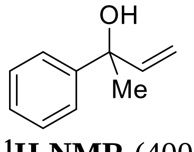

^{1}H NMR (400 MHz, $CDCl_3$) δ 7.51, 7.51, 7.50, 7.49, 7.49, 7.49, 7.39, 7.39, 7.38, 7.37, 7.36, 7.35, 7.35, 7.30, 7.30, 7.29, 7.28, 7.28, 7.26, 6.23, 6.20, 6.19, 6.16, 5.34, 5.34, 5.30, 5.30, 5.18,

5.18, 5.16, 5.15, 2.17, 1.68. **^{13}C NMR** (101 MHz, $CDCl_3$) δ 146.4, 144.8, 128.1, 126.9, 125.1, 112.3, 74.7, 29.2.

2-iodo-N-phenylaniline (AA1)

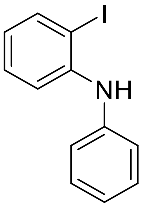

^{1}H NMR (400 MHz, Chloroform-*d*) δ = 7.83 – 7.76 (m, 1H), 7.34 (dd, *J* = 8.5, 7.4 Hz, 2H), 7.24 – 7.20 (m, 2H), 7.15 (dt, *J* = 7.8, 1.1 Hz, 2H), 7.09 – 7.02 (m, 1H), 6.64 (ddd, *J* = 7.9, 5.4, 3.3 Hz, 1H), 5.93 (s, 1H) ppm. **^{13}C NMR** (101 MHz, $CDCl_3$) δ = 143.9, 142.0, 139.5, 129.4, 129.0, 122.5, 121.9, 120.0, 115.9, 88.8 ppm.

1-(4-methoxyphenyl)pyrrolidin-2-one (A2)

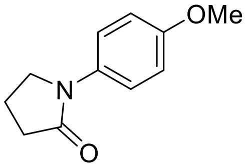

^{1}H NMR (400 MHz, Chloroform-*d*) δ = 7.52 – 7.44 (m, 2H), 6.93 – 6.82 (m, 2H), 3.80 (d, *J* = 6.9 Hz, 2H), 3.78 (s, 3H), 2.56 (t, *J* = 8.1 Hz, 2H), 2.18 – 2.05 (m, 2H) ppm. **^{13}C NMR** (101 MHz, $CDCl_3$) δ = 173.8, 156.5, 132.6, 121.7, 114.0, 55.4, 49.1, 32.4, 17.9 ppm.

(5-phenyltetrahydrofuran-3-yl)methanol (**2h**′)

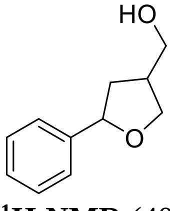

^{1}H NMR (400 MHz, Chloroform-*d*) δ =7.41 – 7.32 (m, 10H), 7.29 (dq, *J* = 6.0, 2.9 Hz, 3H), 5.00 (t, *J* = 7.2 Hz, 1H), 4.88 (dd, *J* = 9.4, 6.4 Hz, 1H), 4.24 (dd, *J* = 8.8, 7.1 Hz, 1H), 4.04 (dd, *J* = 8.8, 7.5 Hz, 1H), 3.95 (dd, *J* = 8.8, 5.6 Hz, 1H), 3.77 (dd, *J* = 8.8, 5.9 Hz, 2H), 3.69 (dd, *J* = 10.9, 7.0 Hz, 2H), 3.65 (dd, *J* = 6.9, 1.9 Hz, 2H), 2.72 – 2.54 (m, 2H), 2.48 (ddd, *J* = 12.5, 8.1, 6.4 Hz, 1H), 2.19 (ddd, *J* = 12.3, 7.2, 5.0 Hz, 1H), 2.00 (ddd, *J* = 12.7, 8.5, 7.3 Hz, 1H), 1.90 – 1.80 (m, 2H), 1.77 (s, 1H), 1.55 (ddd, *J* = 12.4, 9.4, 7.8 Hz, 1H) ppm. **^{13}C NMR** (100 MHz, $CDCl_3$) δ = 143.1, 142.3, 128.3, 128.3, 127.3, 127.2, 125.7, 125.5, 81.2, 80.1, 71.1, 70.9,

65.1, 64.5, 42.4, 41.7, 37.8, 37.3 ppm. **HRMS** (**ESI**) calcd. for $C_{11}H_{14}NaO_2$ [M+Na] *m/z* 201.0886 found *m/z* 201.0881.

Phenyl(2-phenyltetrahydrofuran-3-yl)methyl acetate (**2h′′**)

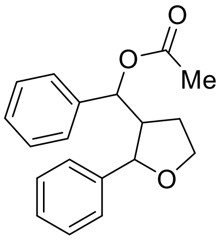

IR (neat) 3063, 3032, 2947, 2875.58, 1738, 1494, 1455, 1371, 1233.84, 1063, 1024, 962, 913, 756.71, 700 cm^{-1}. **^{1}H NMR** (400 MHz, Chloroform-*d*) δ = 7.38 – 7.33 (m, 8H), 7.32 – 7.27 (m, 2H), 7.25 – 7.18 (m, 4H), 7.08 (ddd, *J* = 7.9, 1.6, 0.6 Hz, 3H), 5.79 (d, *J* = 9.7 Hz, 1H), 4.78 (d, *J* = 6.3 Hz, 1H), 4.02 – 3.98 (m, 1H), 3.98 – 3.92 (m, 1H), 2.88 – 2.77 (m, 1H), 2.11 (s, 3H), 2.09 – 2.03 (m, 1H), 1.91 – 1.83 (m, 1H) ppm. **^{13}C NMR** (101 MHz, $CDCl_3$) δ = 173.8, 156.5, 132.6, 121.7, 114.0, 55.4, 49.1, 32.4, 17.9 ppm. **HRMS** (**ESI**) calcd. for $C_{19}H_{20}NaO_3$ [M+Na] *m/z* 319.1305 found *m/z* 319.1310.

(*R*,*E*)-1-(2-mercaptophenyl)-3,7-dimethylocta-1,6-dien-3-ol:

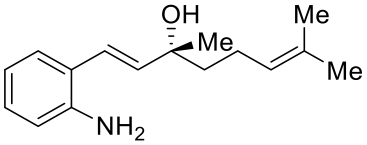

^{1}H NMR (400 MHz, Chloroform-*d*) δ 7.24 (d, *J* = 1.5 Hz, 1H), 7.07 (td, *J* = 7.6, 1.6 Hz, 1H), 6.76 (td, *J* = 7.5, 1.1 Hz, 1H), 6.71 – 6.58 (m, 2H), 6.15 (d, *J* = 15.8 Hz, 1H), 5.15 (tt, *J* = 7.2, 1.5 Hz, 1H), 3.74 (s, 2H), 2.20 – 1.99 (m, 2H), 1.69 (d, *J* = 1.5 Hz, 2H), 1.61 (d, *J* = 1.2 Hz, 3H), 1.39 (s, 3H) ppm. **^{13}C NMR** (101 MHz, $CDCl_3$) δ 143.6, 138.6, 132.2, 128.3, 127.3, 124.3, 123.4, 122.5, 118.9, 116.0, 73.7, 42.6, 28.6, 25.7, 23.0, 17.8 ppm.

(*R*)-1-(2-aminophenyl)-3,7-dimethyloctan-3-ol (DD):

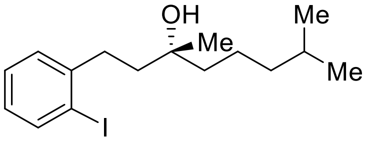

^{1}H NMR (400 MHz, Chloroform-*d*) δ 7.80 (dd, *J* = 7.9, 1.2 Hz, 1H), 7.27 (d, *J* = 1.2 Hz, 1H), 7.24 – 7.18 (m, 1H), 6.88 (ddd, *J* = 7.9, 7.0, 2.1 Hz, 1H), 2.87 – 2.72 (m, 2H), 1.75 – 1.65 (m, 2H), 1.59 – 1.47 (m, 3H), 1.44 – 1.33 (m, 2H), 1.28 (s, 3H), 1.25 – 1.14 (m, 2H), 0.89 (d, *J* = 6.7 Hz, 6H) ppm. **^{13}C NMR** (101 MHz, $CDCl_3$) δ 145.2, 139.5, 129.4, 128.5, 127.7, 100.4, 72.7, 42.5, 42.3, 39.5, 35.6, 28.0, 26.9, 22.7, 21.8 ppm.

(*R*)-S-(2-(3-hydroxy-3,7-dimethyloctyl)phenyl) benzothioate (EE):

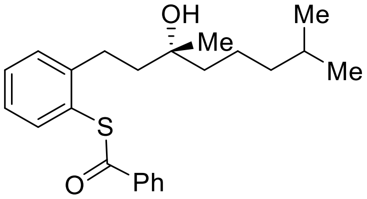

^{1}H NMR (400 MHz, Chloroform-*d*) δ 8.05 (dd, *J* = 8.4, 1.4 Hz, 2H), 7.64 – 7.58 (m, 1H), 7.49 (ddd, *J* = 8.6, 4.5, 3.2 Hz, 3H), 7.44 – 7.35 (m, 2H), 7.32 – 7.27 (m, 1H), 2.86 – 2.74 (m, 2H), 1.76 – 1.67 (m, 2H), 1.48 – 1.40 (m, 2H), 1.36 – 1.23 (m, 2H), 1.20 (m, 1H), 1.19 (s, 3H), 1.11 (dd, *J* = 8.3, 6.8 Hz, 2H), 0.82 (dd, *J* = 6.6, 0.6 Hz, 6H) ppm. **^{13}C NMR** (101 MHz, $CDCl_3$) δ 146.8, 137.1, 136.7, 133.6, 130.4, 130.1, 128.7, 127.5, 126.9, 126.2, 72.7, 43.3, 42.1, 39.5, 29.2, 27.9, 26.9, 22.6, 22.6, 21.7 ppm.

(*R*)-2-methyl-2-(4-methylpentyl)chroman-5-ol (3C′)

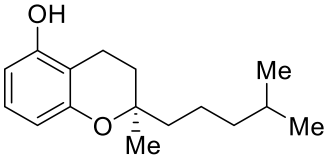

^{1}H NMR (400 MHz, Chloroform-*d*) δ = 7.00 – 6.90 (m, 1H), 6.41 (dd, *J* = 8.3, 1.1 Hz, 1H), 6.31 (dd, *J* = 7.9, 1.1 Hz, 1H), 4.68 (s, 1H), 2.63 (t, *J* = 6.8 Hz, 2H), 1.93 – 1.70 (m, 2H), 1.58 (s, 1H), 1.55 – 1.48 (m, 2H), 1.39 (ddd, *J* = 14.5, 9.4, 7.4 Hz, 2H), 1.27 (s, 3H), 1.22 – 1.10 (m, 2H), 0.87 (dd, *J* = 6.6, 0.8 Hz, 6H) ppm. **^{13}C NMR** (101 MHz, $CDCl_3$) δ =155.1, 153.8, 127.1, 109.9, 108.6, 105.8, 76.0, 39.7, 39.4, 30.2, 27.9, 24.0, 22.6, 22.6, 21.4, 16.6 ppm.

HPLC chromatograms for all starting alcohols and products:

1a:

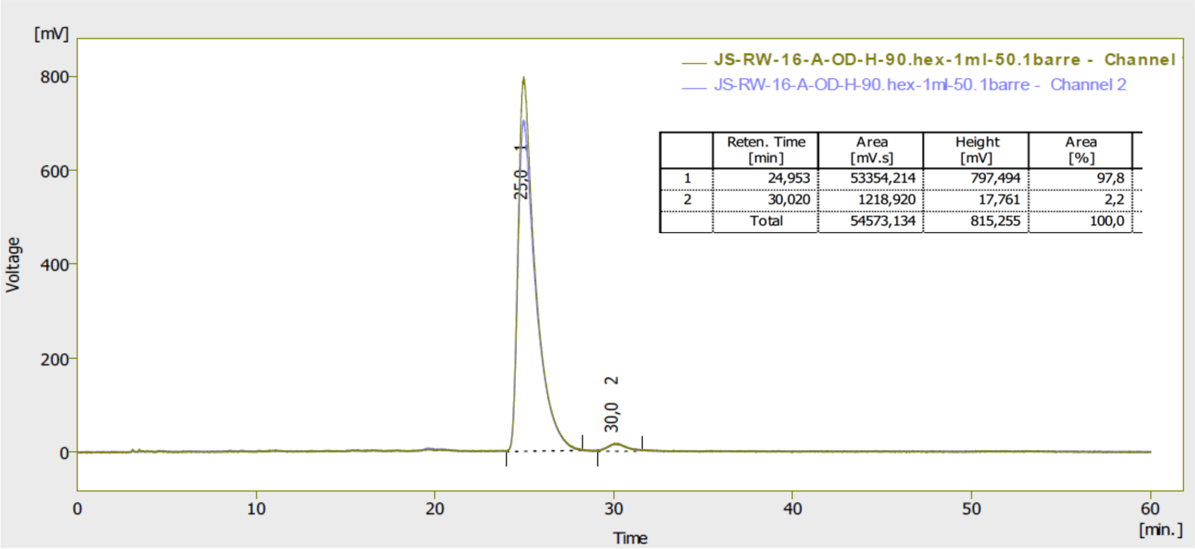

1a: racemates

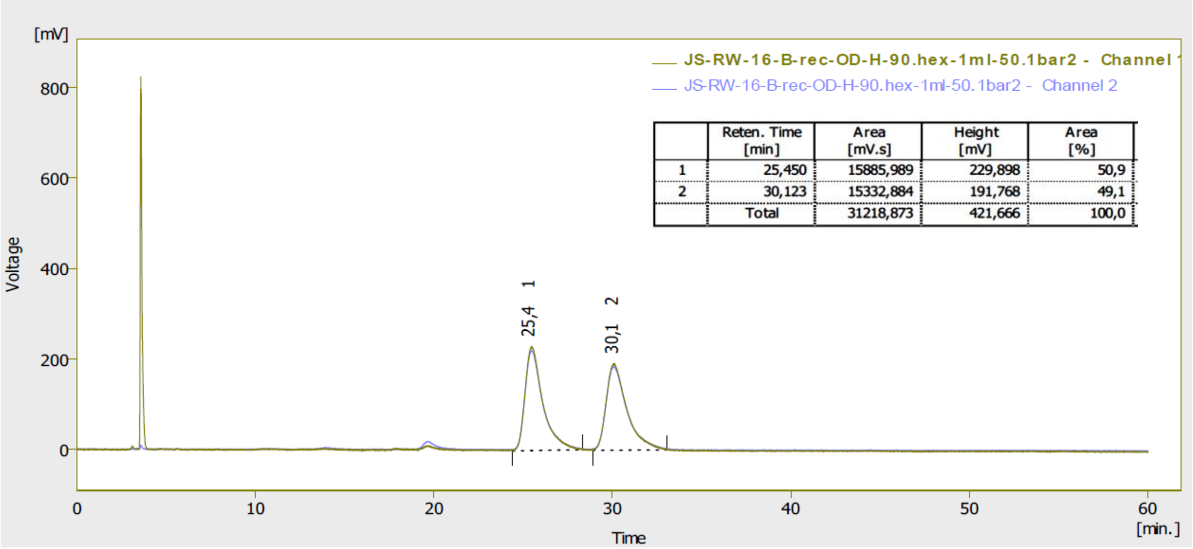

1b:

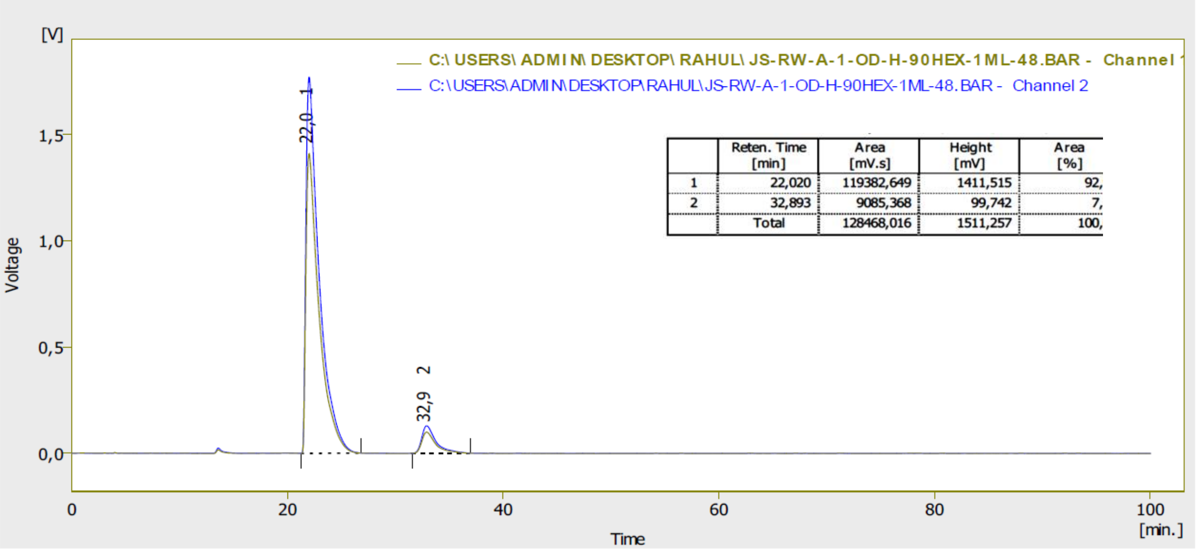

	Reten. Time [min]	Area [mV.s]	Height [mV]	Area [%]
1	22,020	119382,649	1411,515	92,
2	32,893	9085,368	99,742	7,
	Total	128468,016	1511,257	100,

1b: racemates

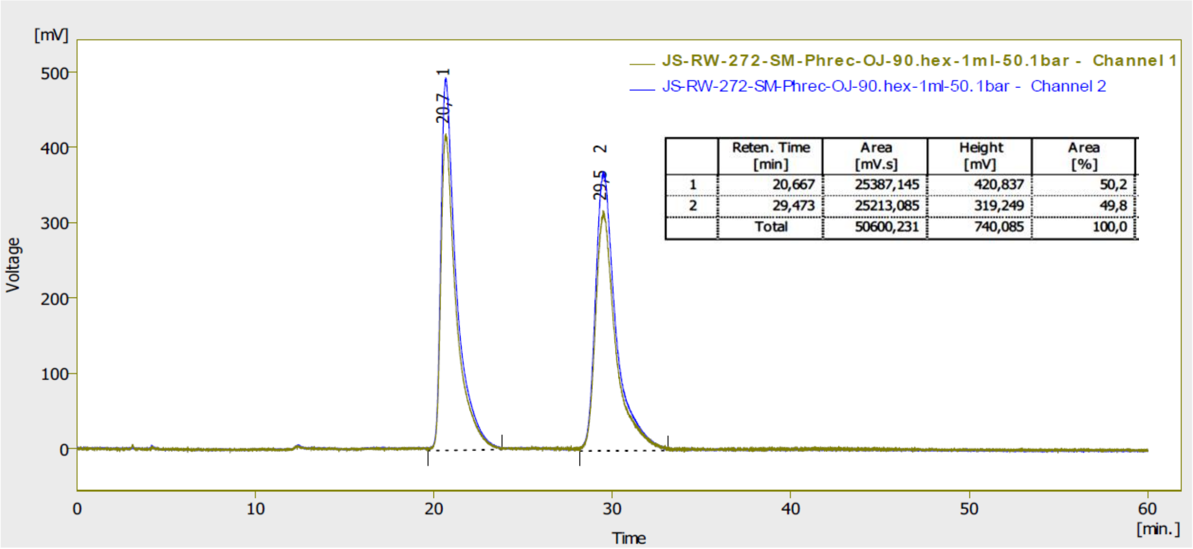

	Reten. Time [min]	Area [mV.s]	Height [mV]	Area [%]
1	20,667	25387,145	420,837	50,2
2	29,473	25213,085	319,249	49,8
	Total	50600,231	740,085	100,0

1c:

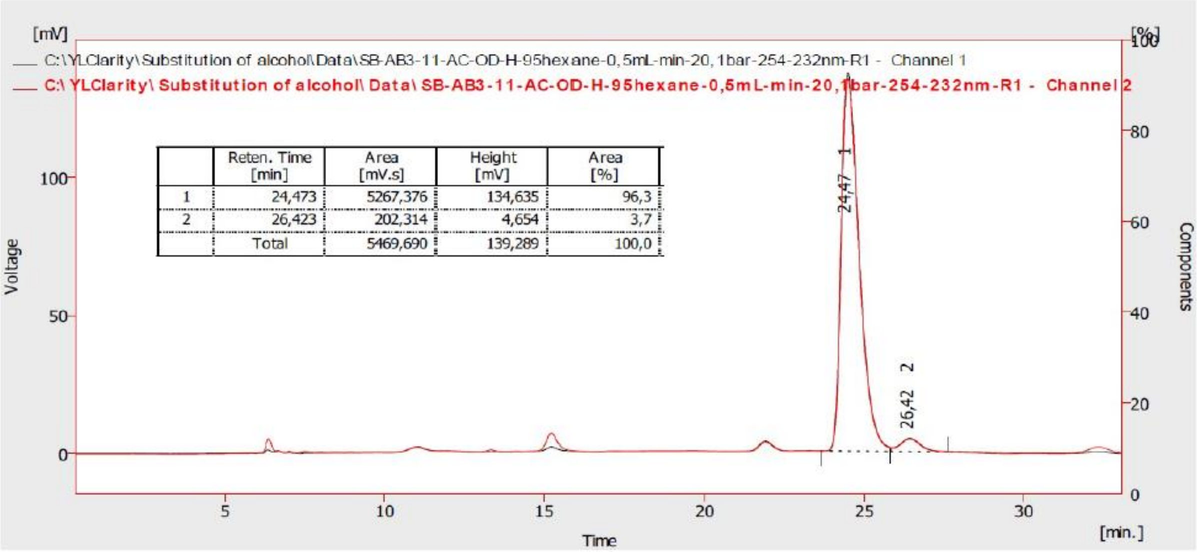

1c: racemates

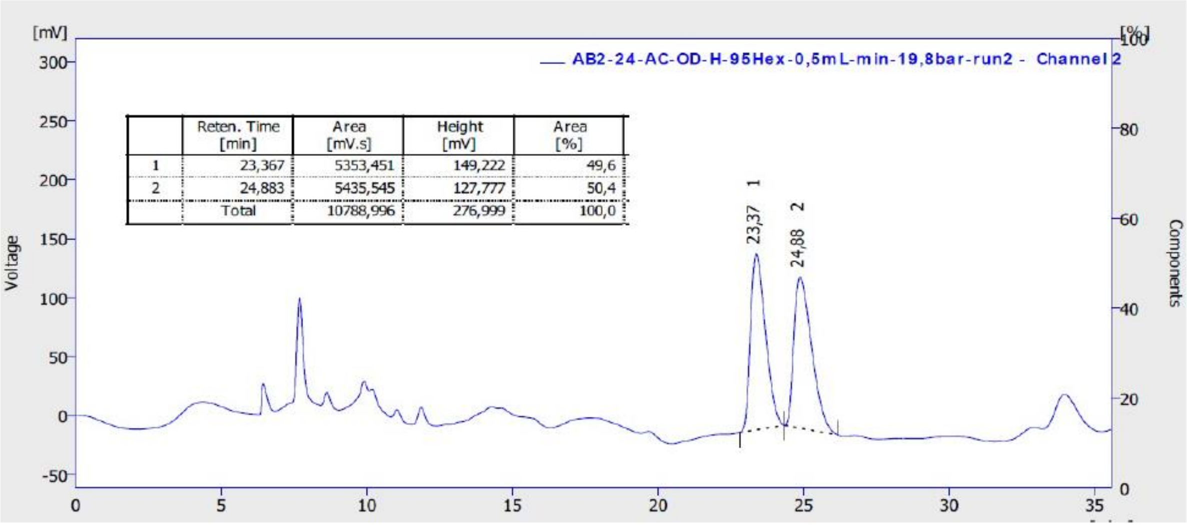

1d:

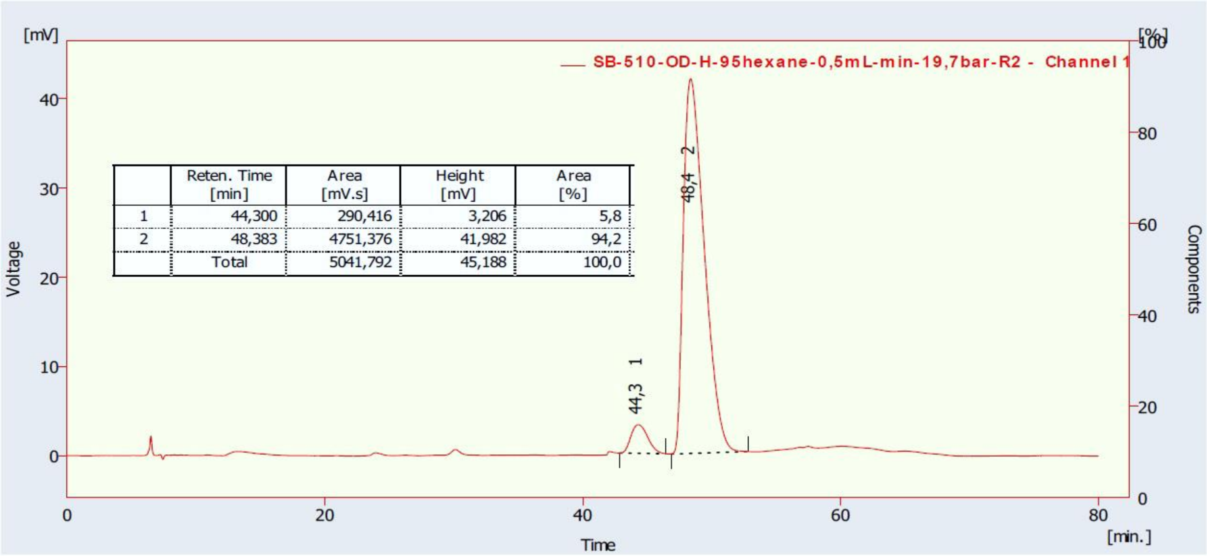

	Reten. Time [min]	Area [mV.s]	Height [mV]	Area [%]
1	44,300	290,416	3,206	5,8
2	48,383	4751,376	41,982	94,2
	Total	5041,792	45,188	100,0

1d: racemates

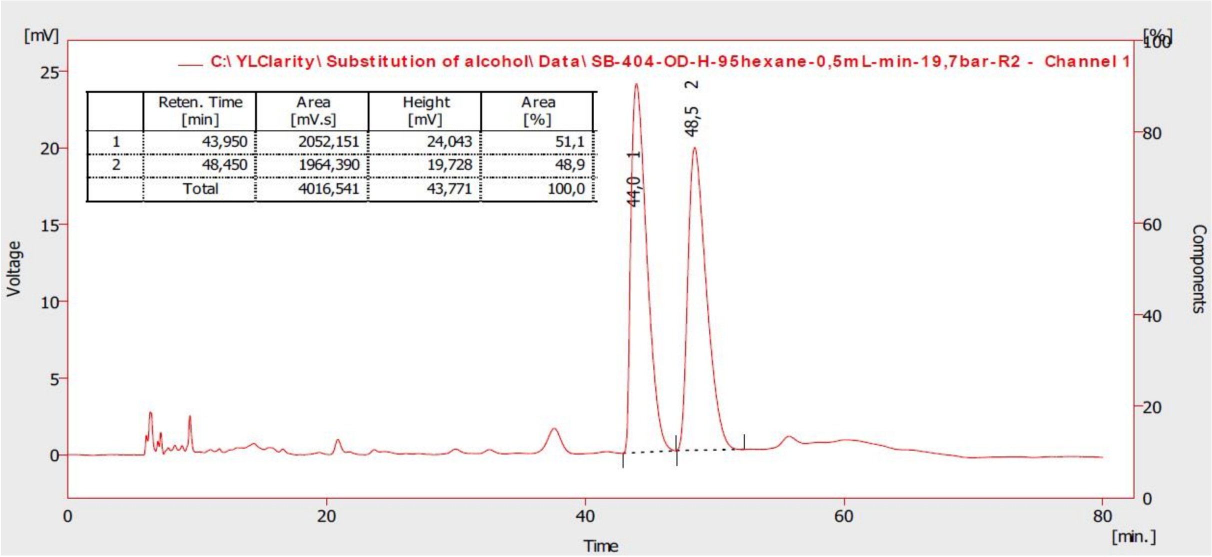

	Reten. Time [min]	Area [mV.s]	Height [mV]	Area [%]
1	43,950	2052,151	24,043	51,1
2	48,450	1964,390	19,728	48,9
	Total	4016,541	43,771	100,0

1e:

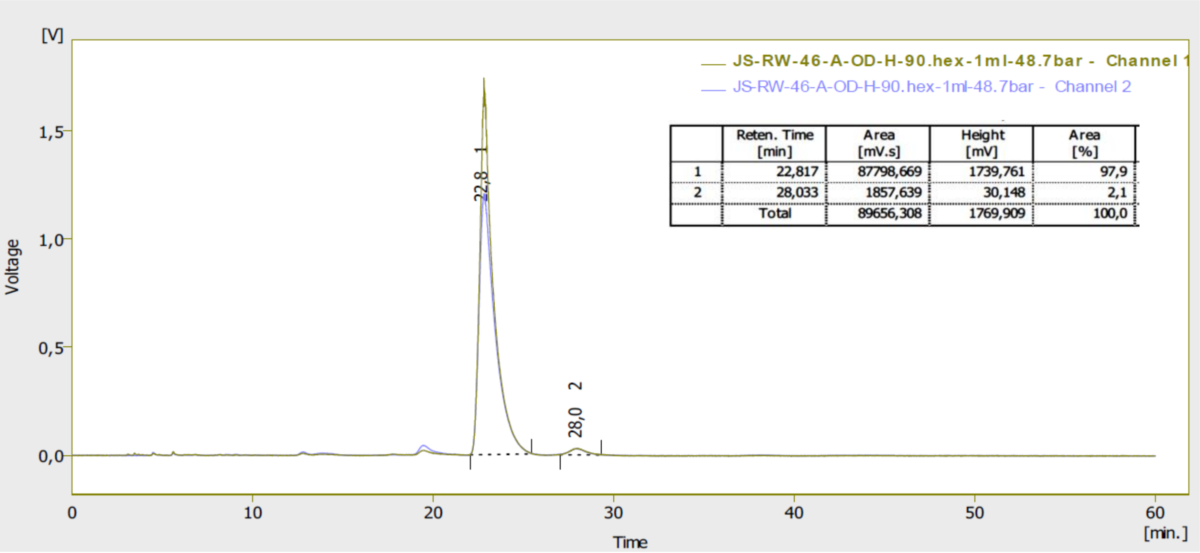

	Reten. Time [min]	Area [mV.s]	Height [mV]	Area [%]
1	22,817	87798,669	1739,761	97,9
2	28,033	1857,639	30,148	2,1
	Total	89656,308	1769,909	100,0

1e: racemates

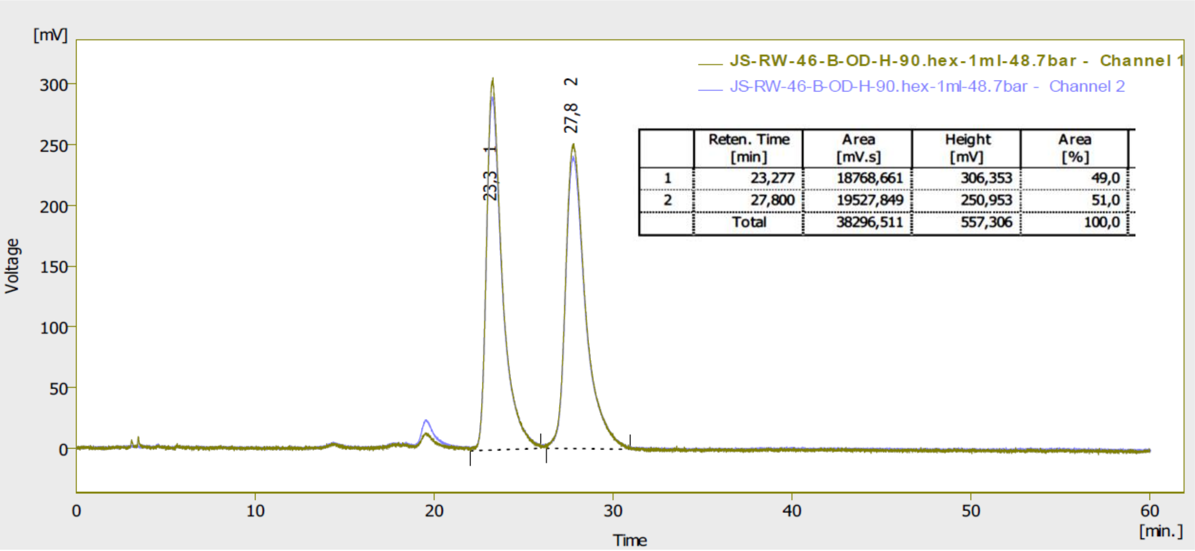

	Reten. Time [min]	Area [mV.s]	Height [mV]	Area [%]
1	23,277	18768,661	306,353	49,0
2	27,800	19527,849	250,953	51,0
	Total	38296,511	557,306	100,0

1f:

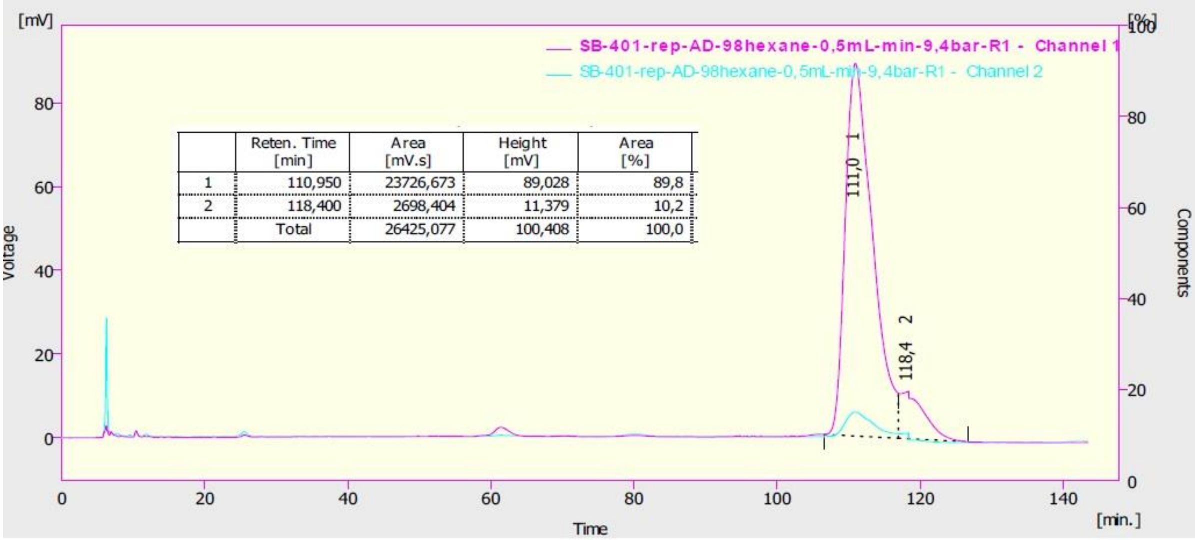

1f: racemates

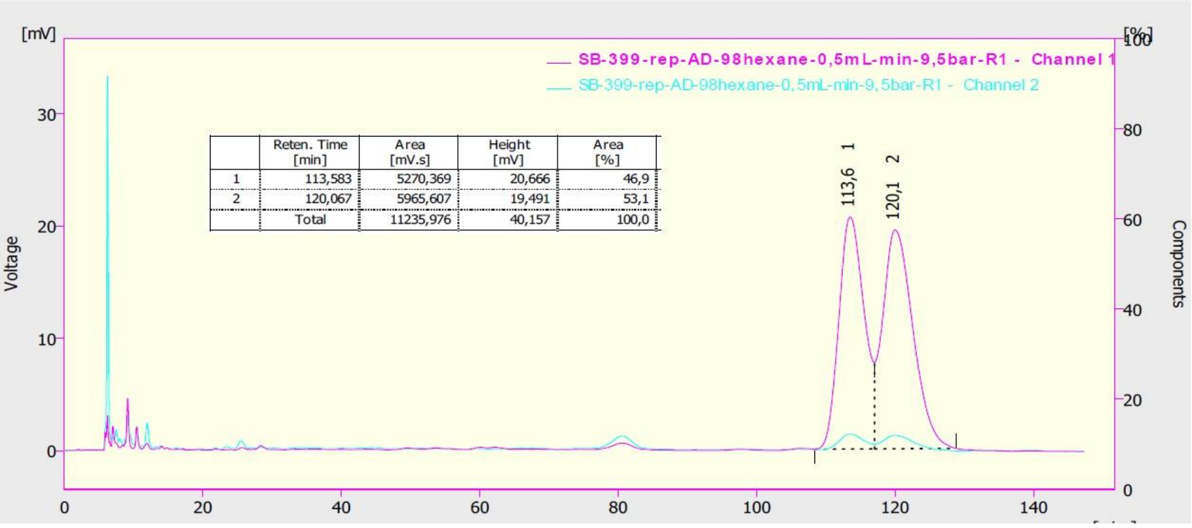

1g:

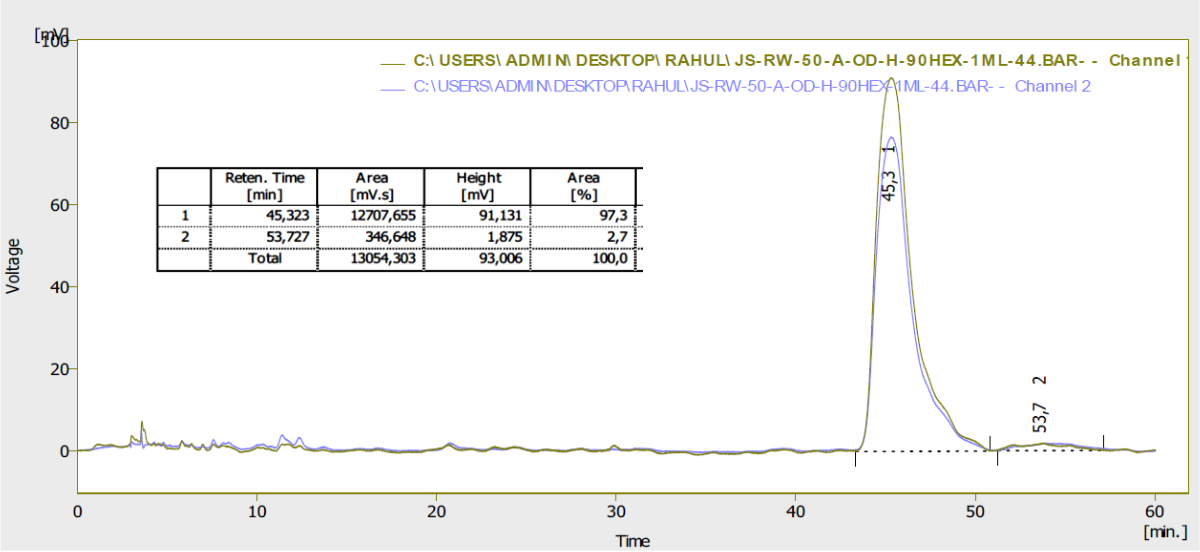

	Reten. Time [min]	Area [mV.s]	Height [mV]	Area [%]
1	45,323	12707,655	91,131	97,3
2	53,727	346,648	1,875	2,7
	Total	13054,303	93,006	100,0

1g: racemates

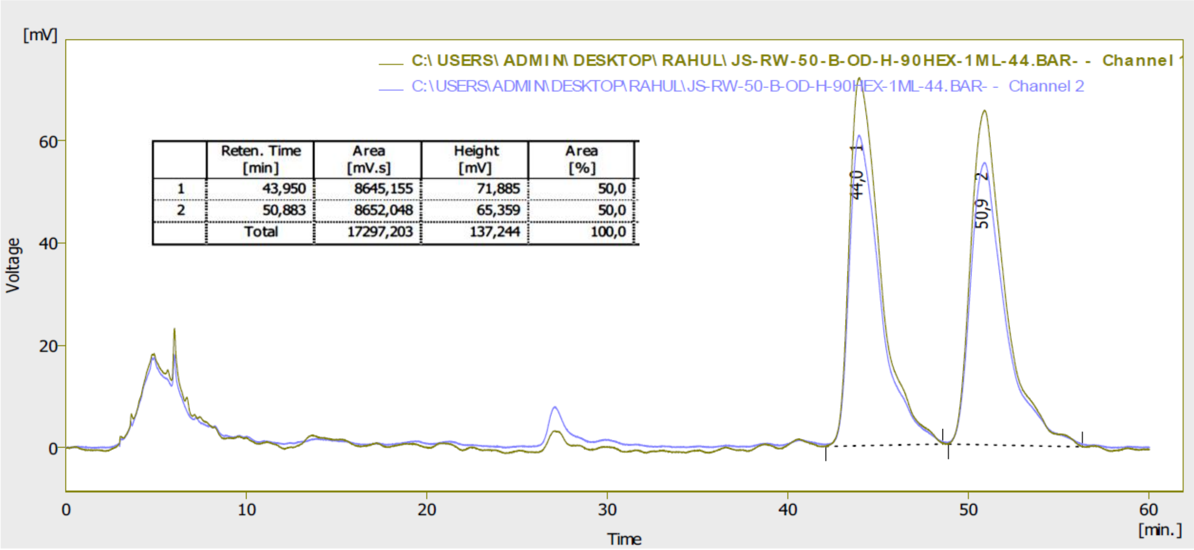

	Reten. Time [min]	Area [mV.s]	Height [mV]	Area [%]
1	43,950	8645,155	71,885	50,0
2	50,883	8652,048	65,359	50,0
	Total	17297,203	137,244	100,0

1h:

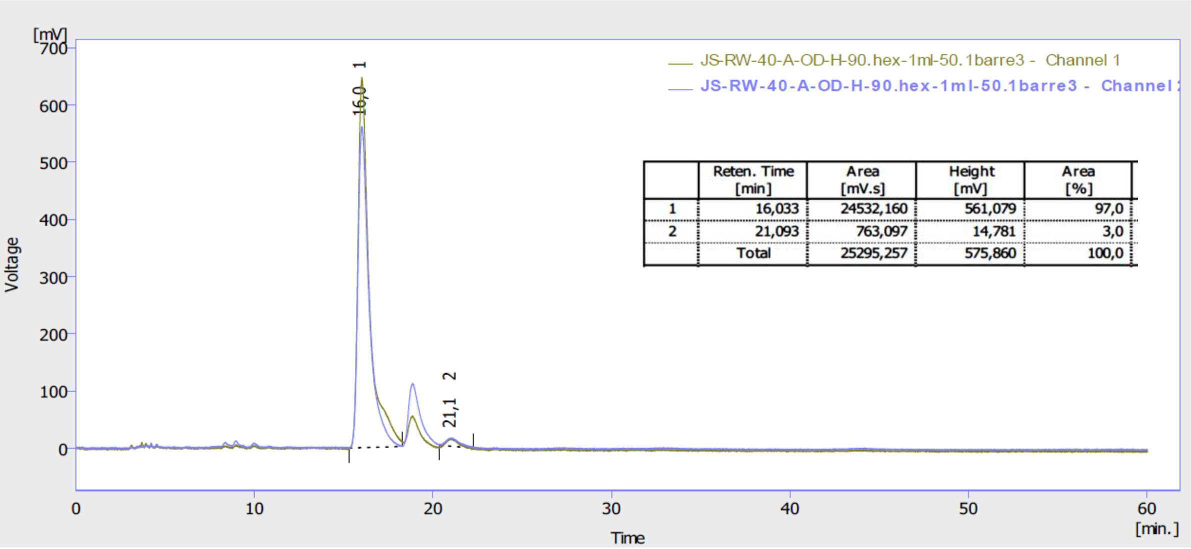

1h: racemates

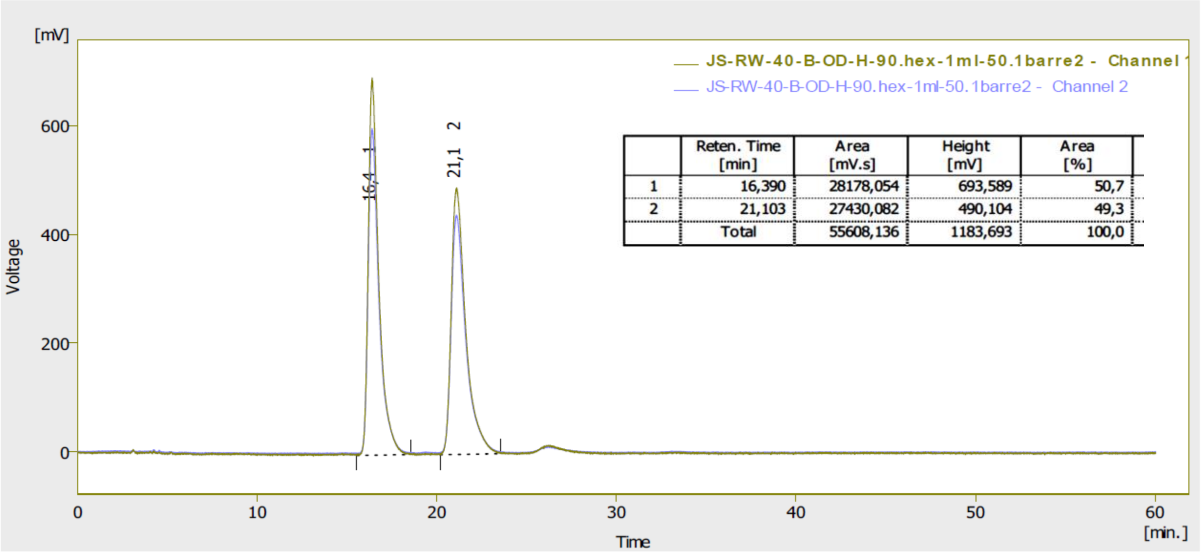

1i:

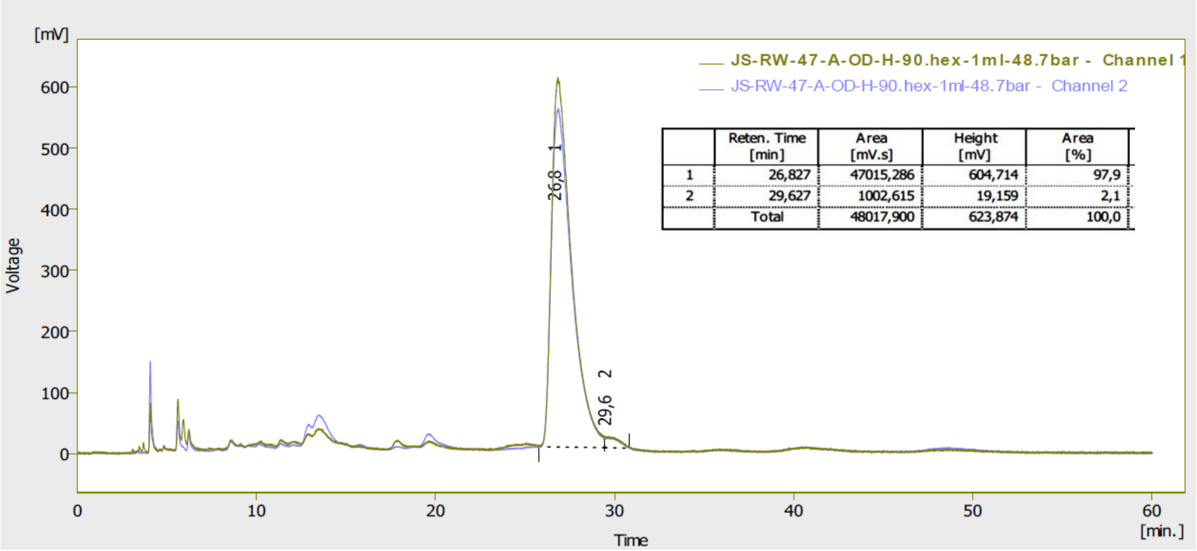

1i: racemates

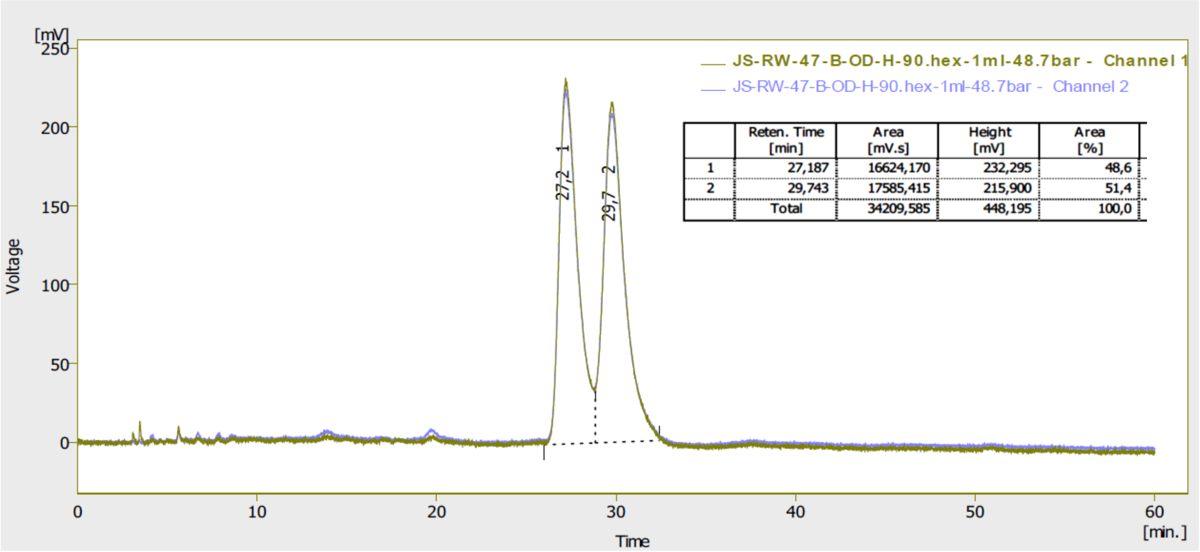

1j:

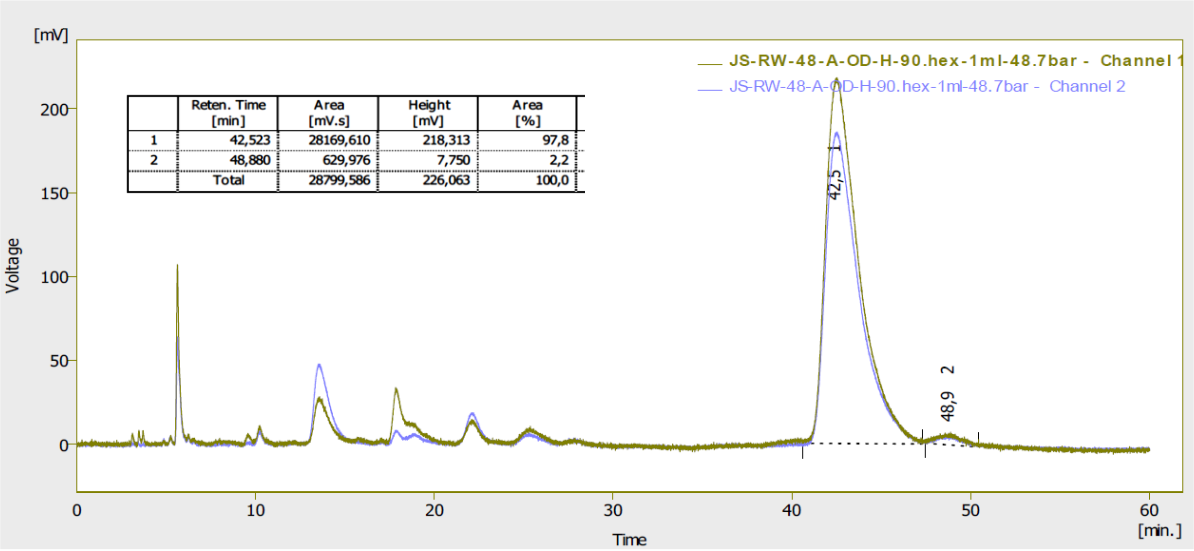

	Reten. Time [min]	Area [mV.s]	Height [mV]	Area [%]
1	42,523	28169,610	218,313	97,8
2	48,880	629,976	7,750	2,2
	Total	28799,586	226,063	100,0

1j: racemates

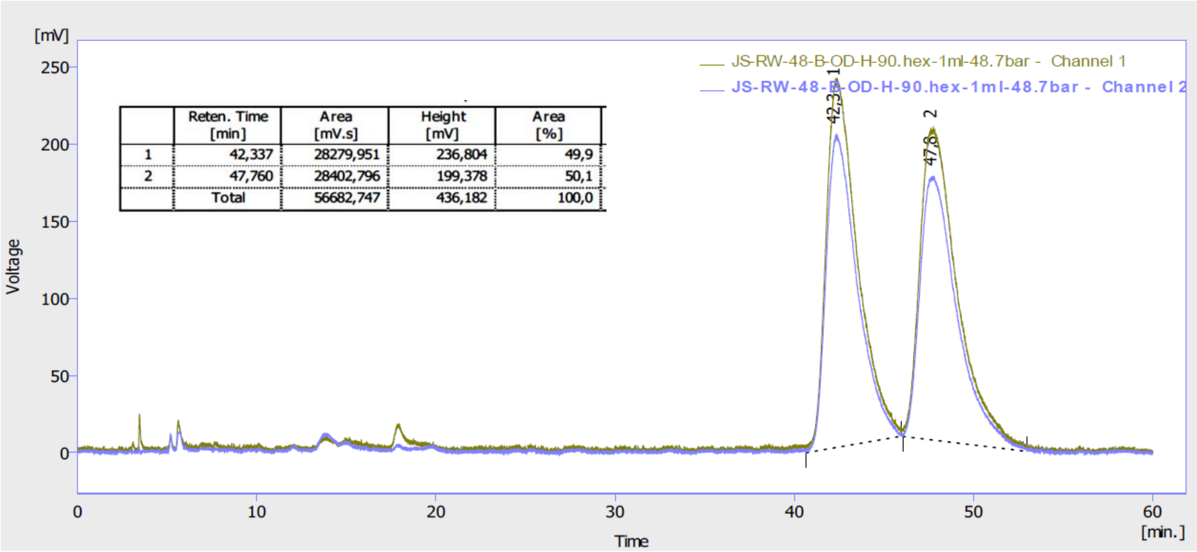

	Reten. Time [min]	Area [mV.s]	Height [mV]	Area [%]
1	42,337	28279,951	236,804	49,9
2	47,760	28402,796	199,378	50,1
	Total	56682,747	436,182	100,0

1k:

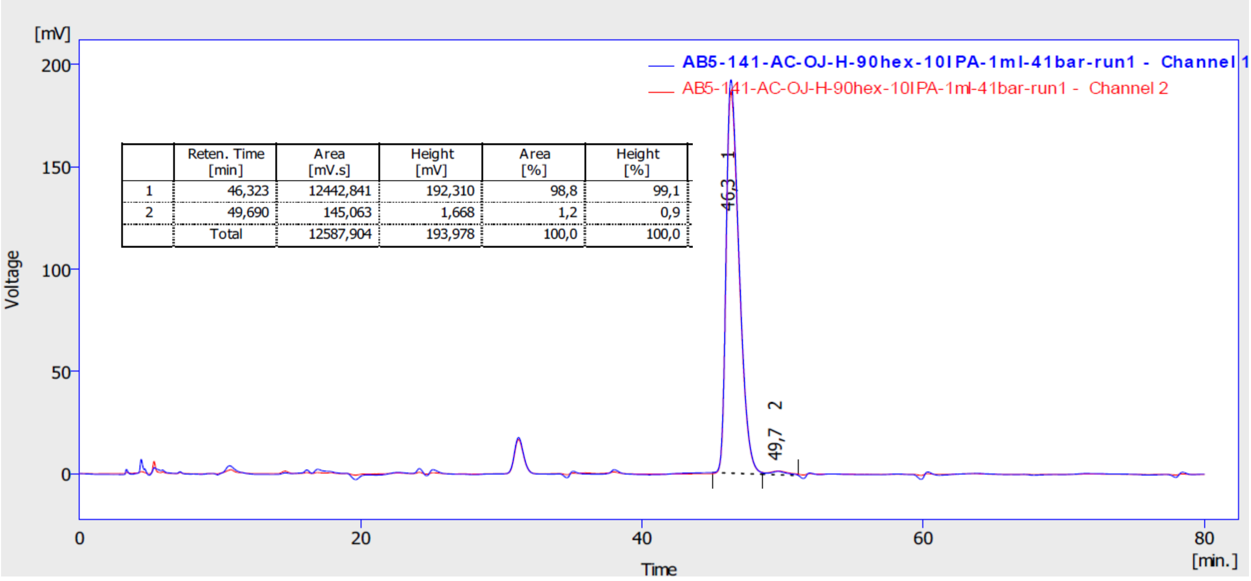

	Reten. Time [min]	Area [mV.s]	Height [mV]	Area [%]	Height [%]
1	46,323	12442,841	192,310	98,8	99,1
2	49,690	145,063	1,668	1,2	0,9
	Total	12587,904	193,978	100,0	100,0

1k: racemates

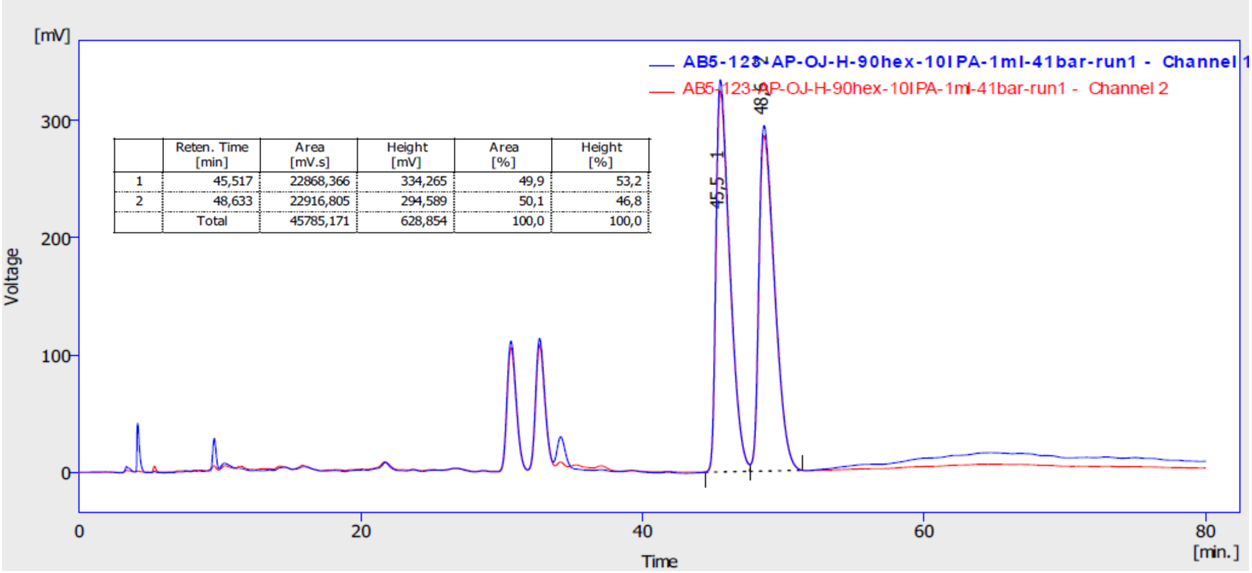

	Reten. Time [min]	Area [mV.s]	Height [mV]	Area [%]	Height [%]
1	45,517	22868,366	334,265	49,9	53,2
2	48,633	22916,805	294,589	50,1	46,8
	Total	45785,171	628,854	100,0	100,0

1l:

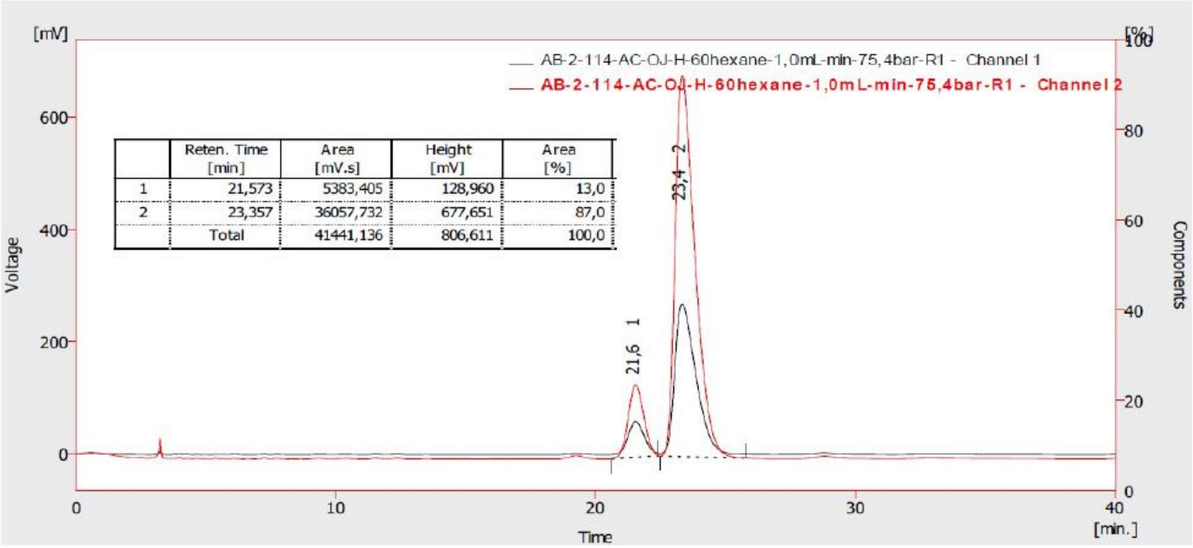

1l : racemates

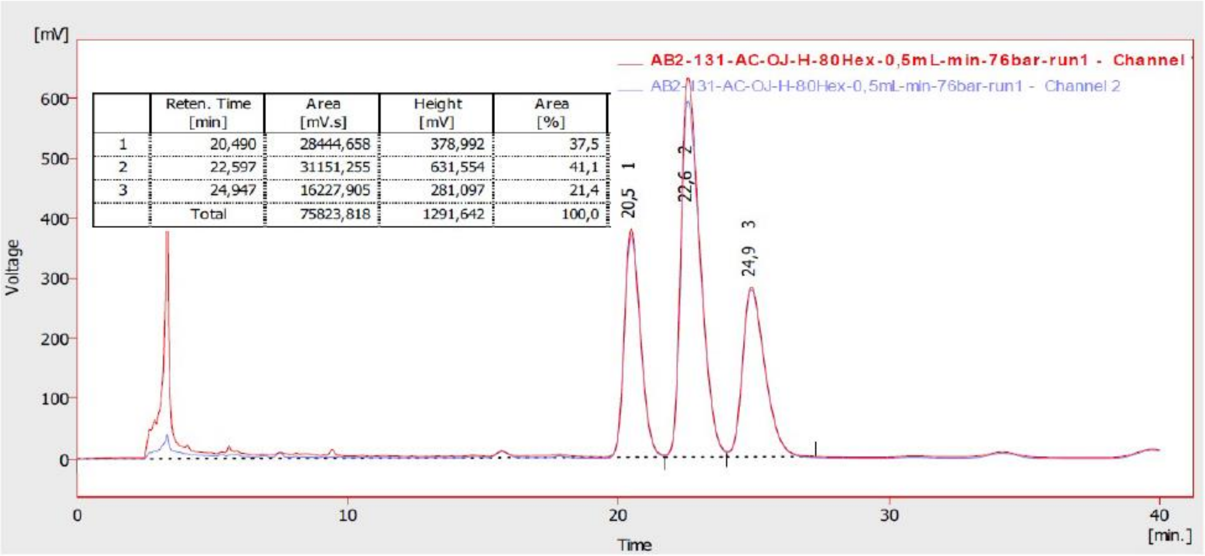

1m:

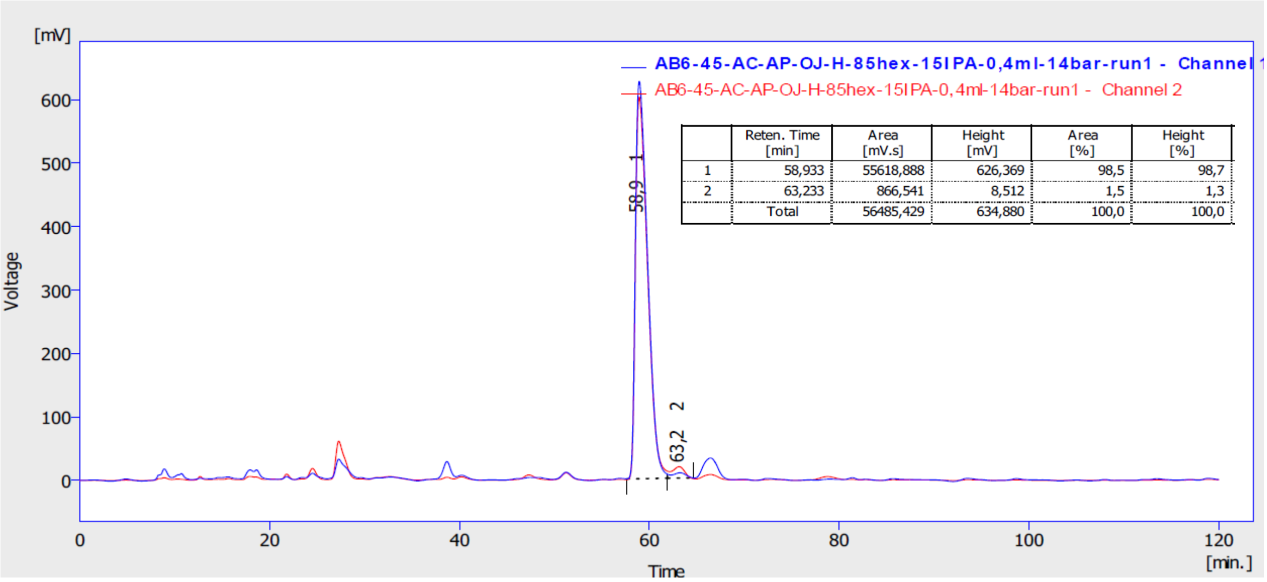

	Reten. Time [min]	Area [mV.s]	Height [mV]	Area [%]	Height [%]
1	58,933	55618,888	626,369	98,5	98,7
2	63,233	866,541	8,512	1,5	1,3
	Total	56485,429	634,880	100,0	100,0

1m: racemates

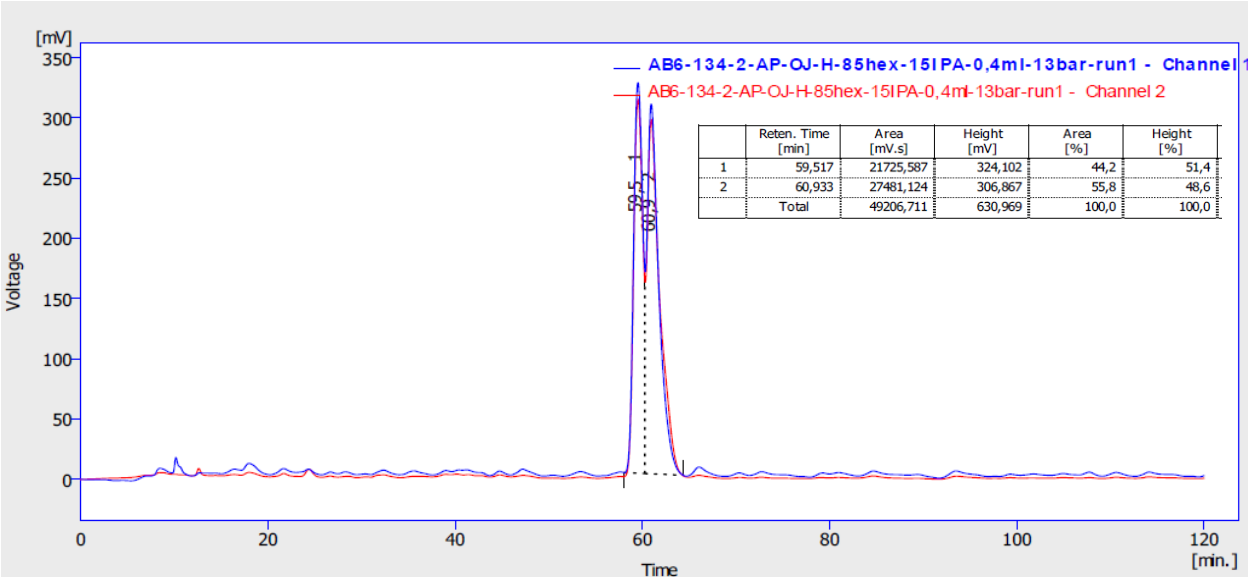

	Reten. Time [min]	Area [mV.s]	Height [mV]	Area [%]	Height [%]
1	59,517	21725,587	324,102	44,2	51,4
2	60,933	27481,124	306,867	55,8	48,6
	Total	49206,711	630,969	100,0	100,0

1n:

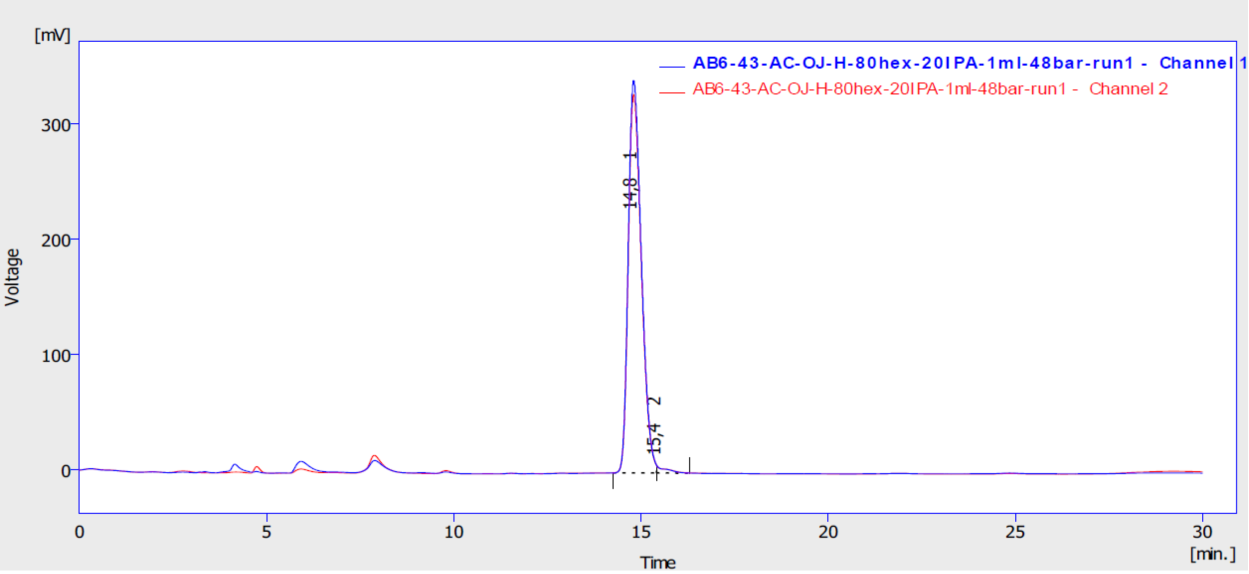

1n: racemates

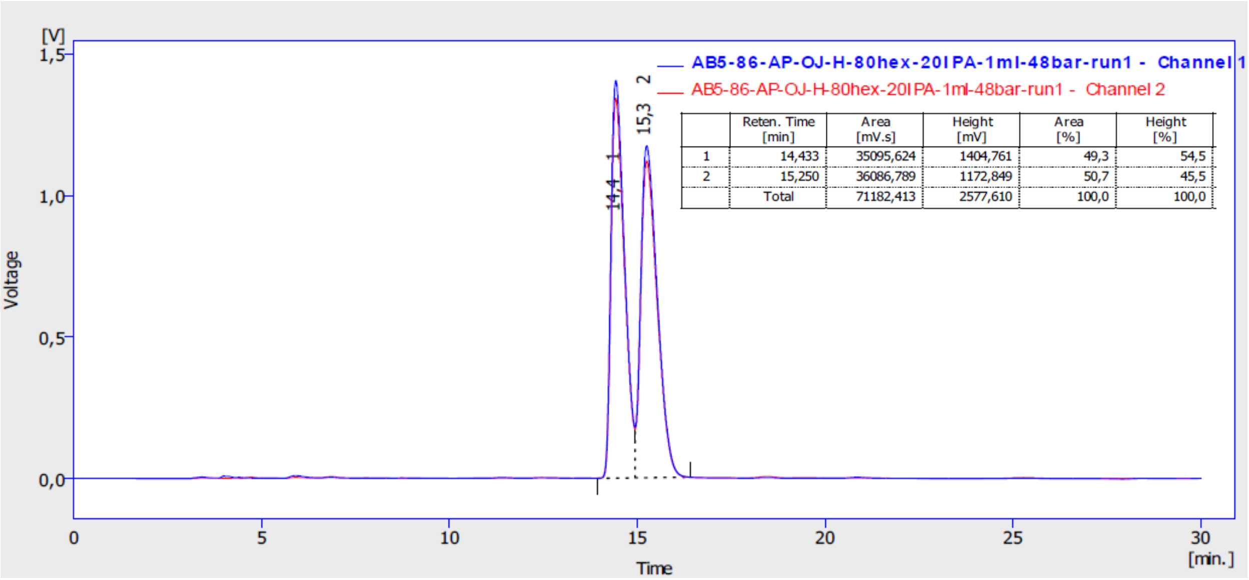

1o:

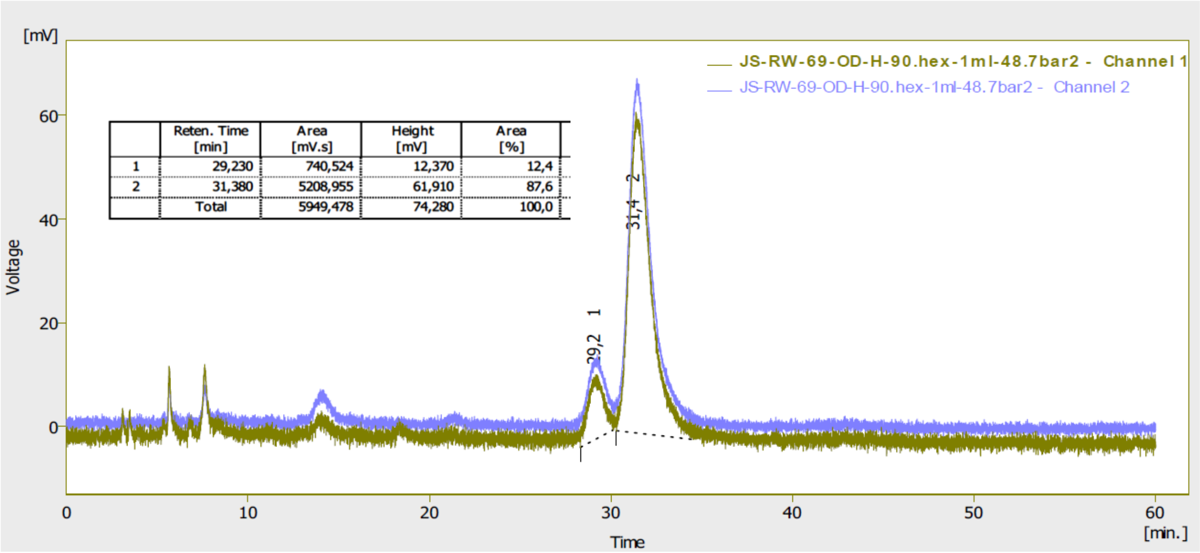

	Reten. Time [min]	Area [mV.s]	Height [mV]	Area [%]
1	29,230	740,524	12,370	12,4
2	31,380	5208,955	61,910	87,6
	Total	5949,478	74,280	100,0

1o: racemates

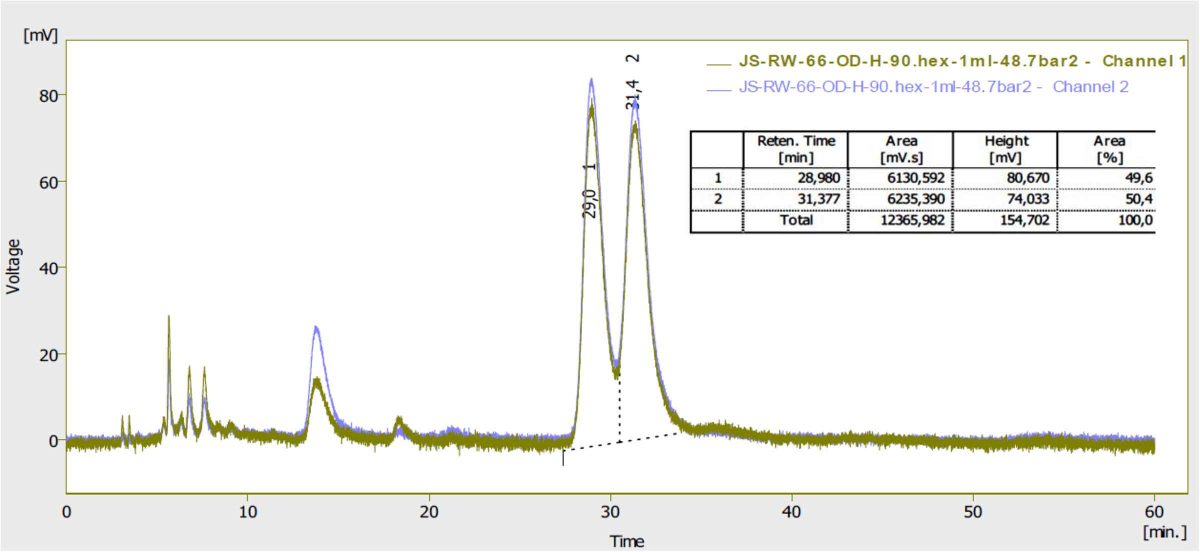

	Reten. Time [min]	Area [mV.s]	Height [mV]	Area [%]
1	28,980	6130,592	80,670	49,6
2	31,377	6235,390	74,033	50,4
	Total	12365,982	154,702	100,0

1p:

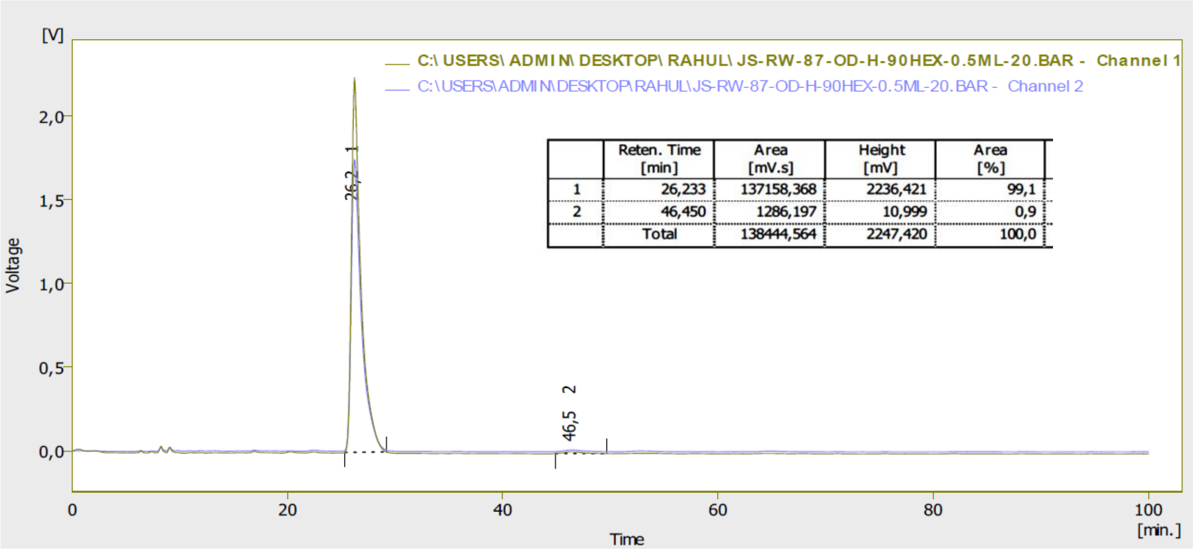

	Reten. Time [min]	Area [mV.s]	Height [mV]	Area [%]
1	26,233	137158,368	2236,421	99,1
2	46,450	1286,197	10,999	0,9
	Total	138444,564	2247,420	100,0

1p: racemates

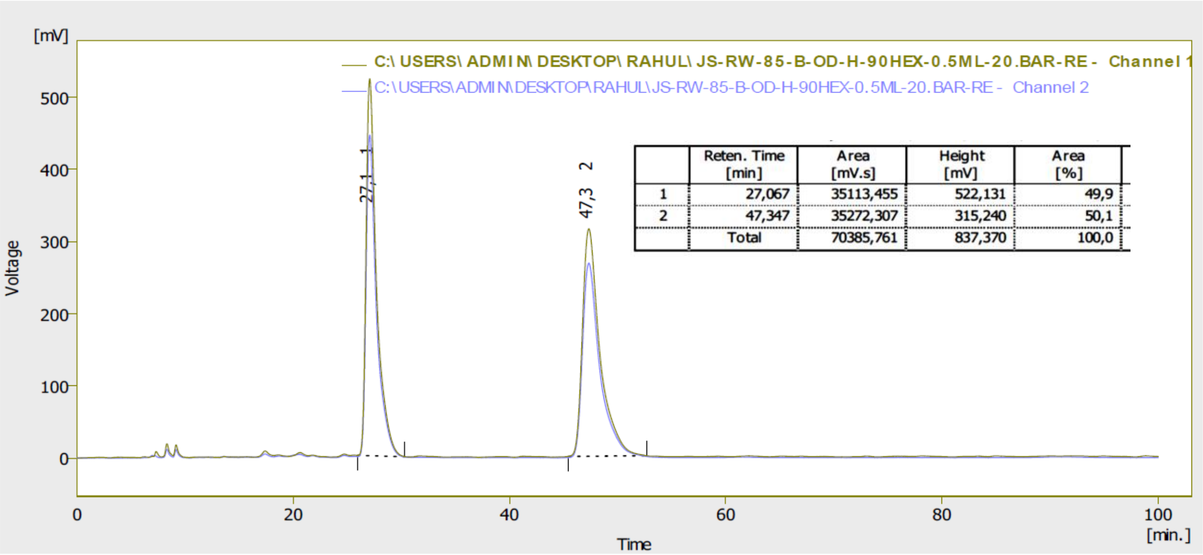

	Reten. Time [min]	Area [mV.s]	Height [mV]	Area [%]
1	27,067	35113,455	522,131	49,9
2	47,347	35272,307	315,240	50,1
	Total	70385,761	837,370	100,0

1q:

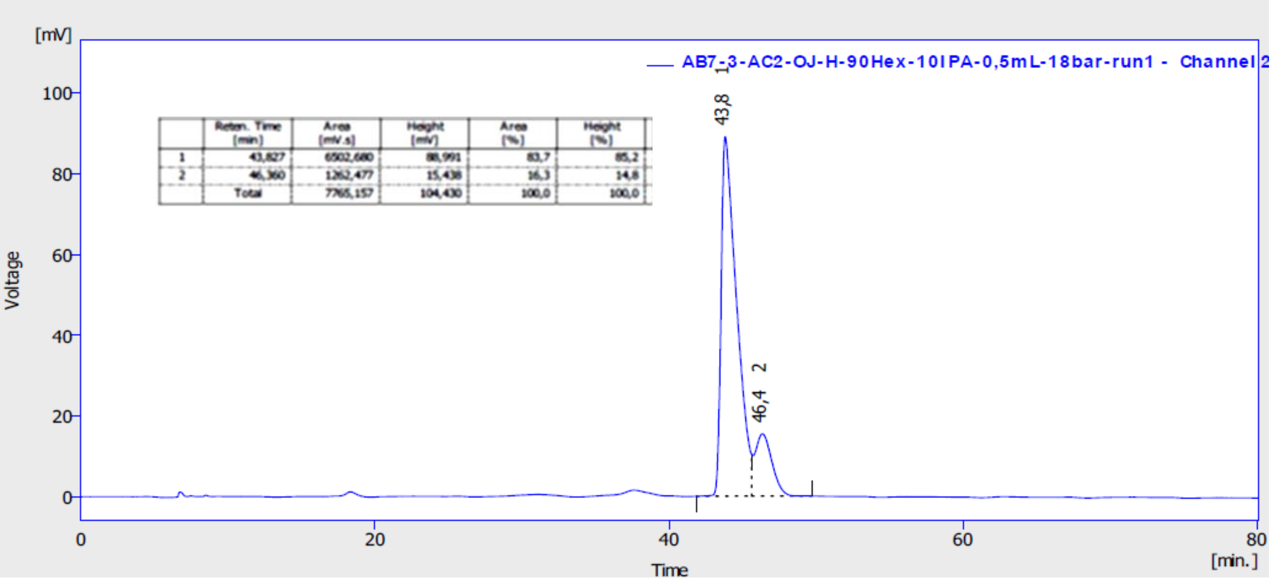

	Reten. Time [min]	Area [mV.s]	Height [mV]	Area [%]	Height [%]
1	43,827	6502,680	88,991	83,7	85,2
2	46,360	1262,477	15,438	16,3	14,8
	Total	7765,157	104,430	100,0	100,0

1q: racemates

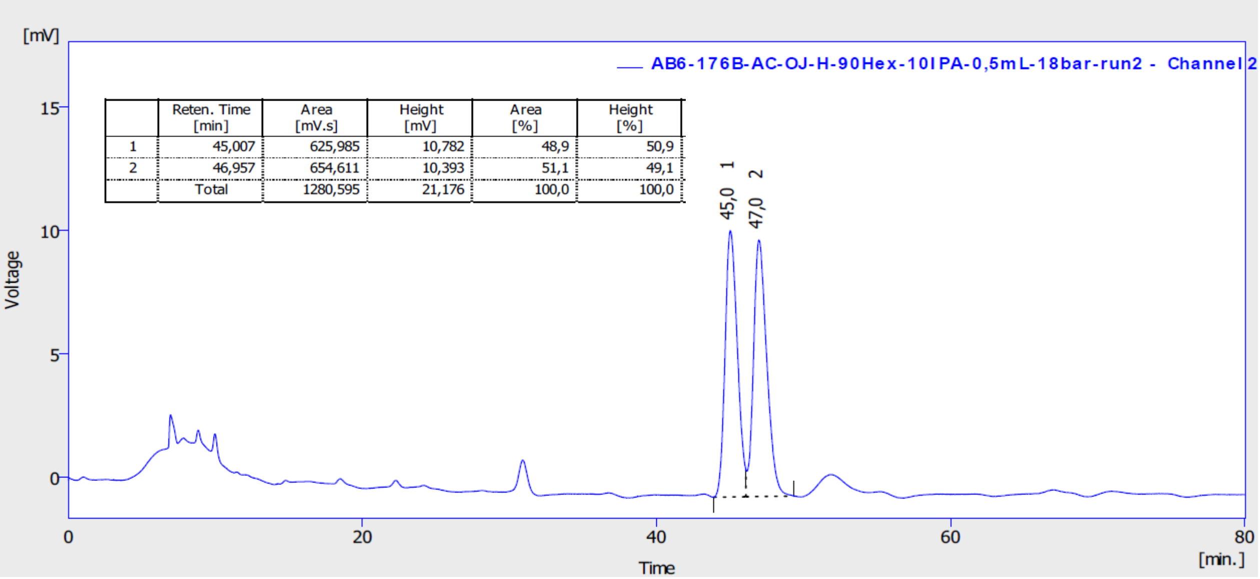

	Reten. Time [min]	Area [mV.s]	Height [mV]	Area [%]	Height [%]
1	45,007	625,985	10,782	48,9	50,9
2	46,957	654,611	10,393	51,1	49,1
	Total	1280,595	21,176	100,0	100,0

1r:

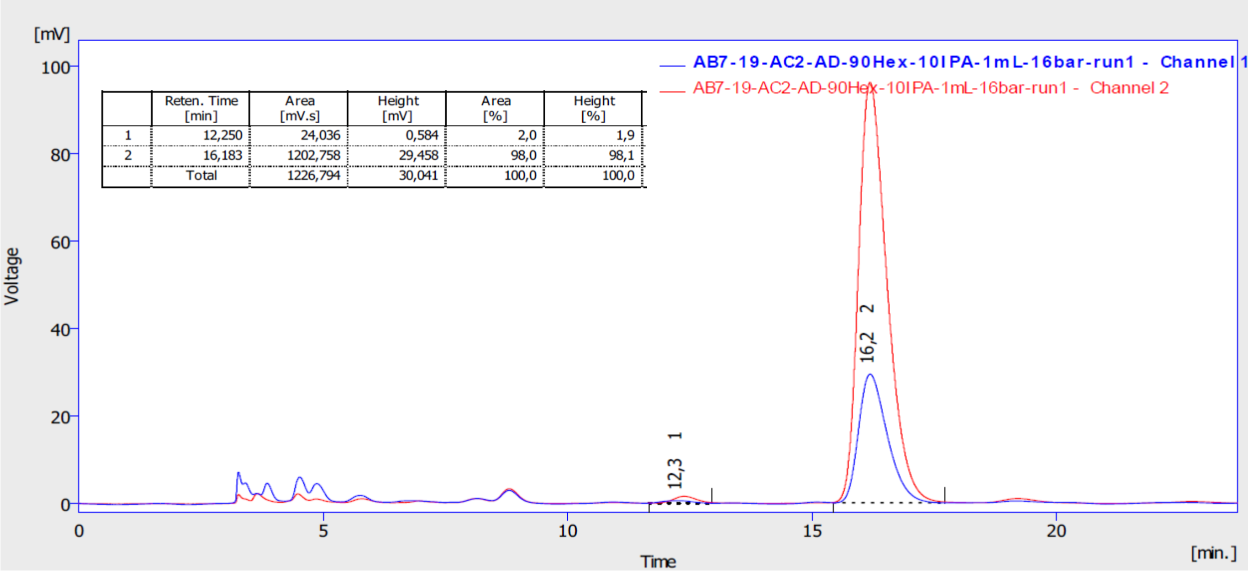

	Reten. Time [min]	Area [mV.s]	Height [mV]	Area [%]	Height [%]
1	12,250	24,036	0,584	2,0	1,9
2	16,183	1202,758	29,458	98,0	98,1
	Total	1226,794	30,041	100,0	100,0

1r: racemates

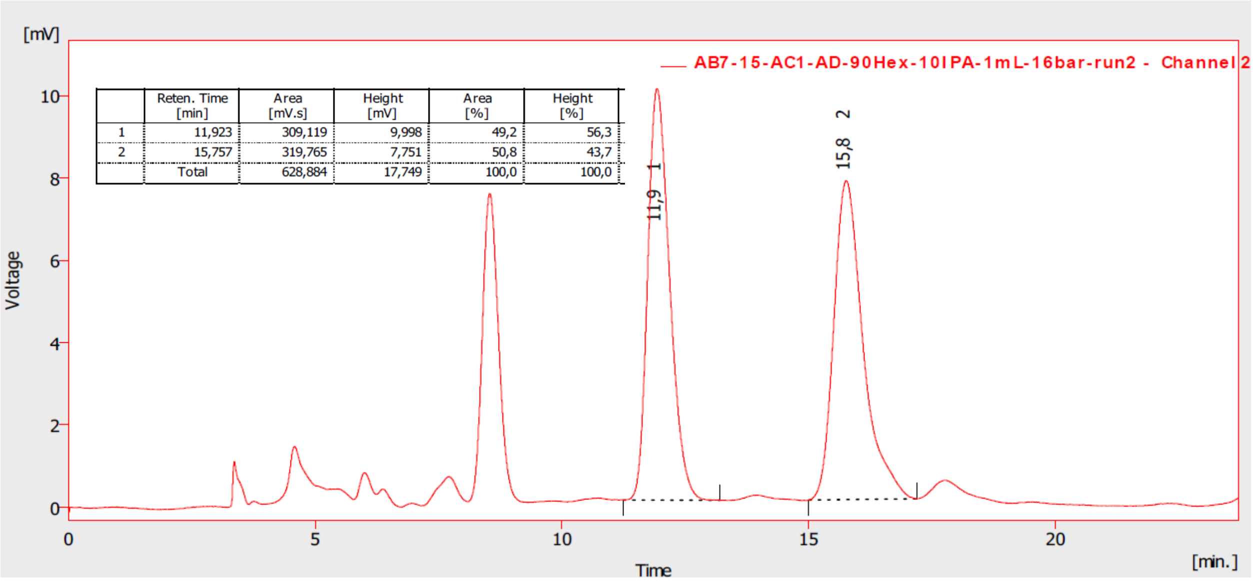

	Reten. Time [min]	Area [mV.s]	Height [mV]	Area [%]	Height [%]
1	11,923	309,119	9,998	49,2	56,3
2	15,757	319,765	7,751	50,8	43,7
	Total	628,884	17,749	100,0	100,0

1s:

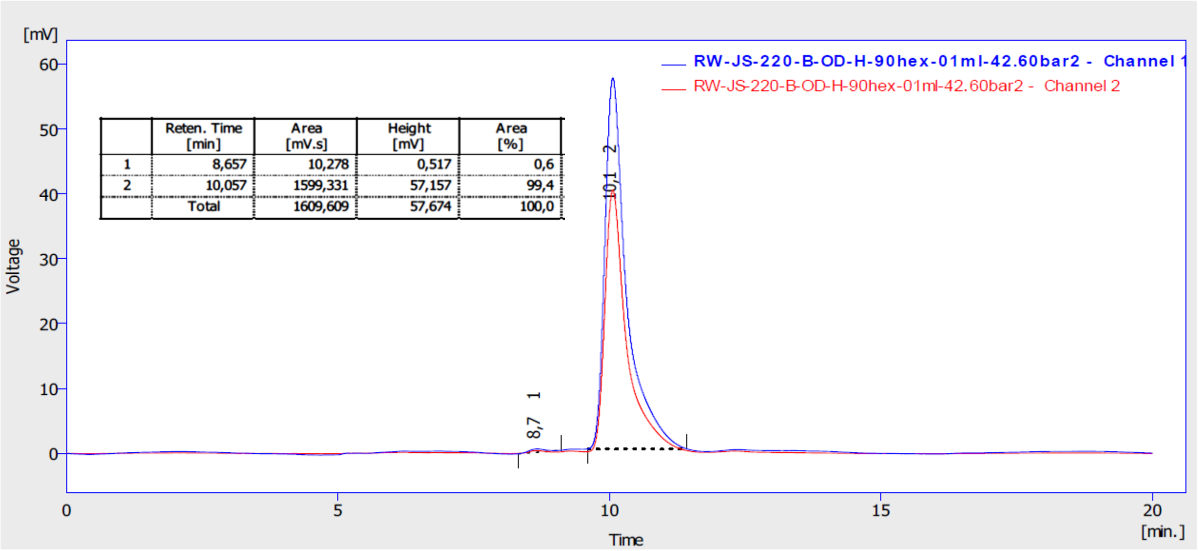

	Reten. Time [min]	Area [mV.s]	Height [mV]	Area [%]
1	8,657	10,278	0,517	0,6
2	10,057	1599,331	57,157	99,4
	Total	1609,609	57,674	100,0

1s: racemates

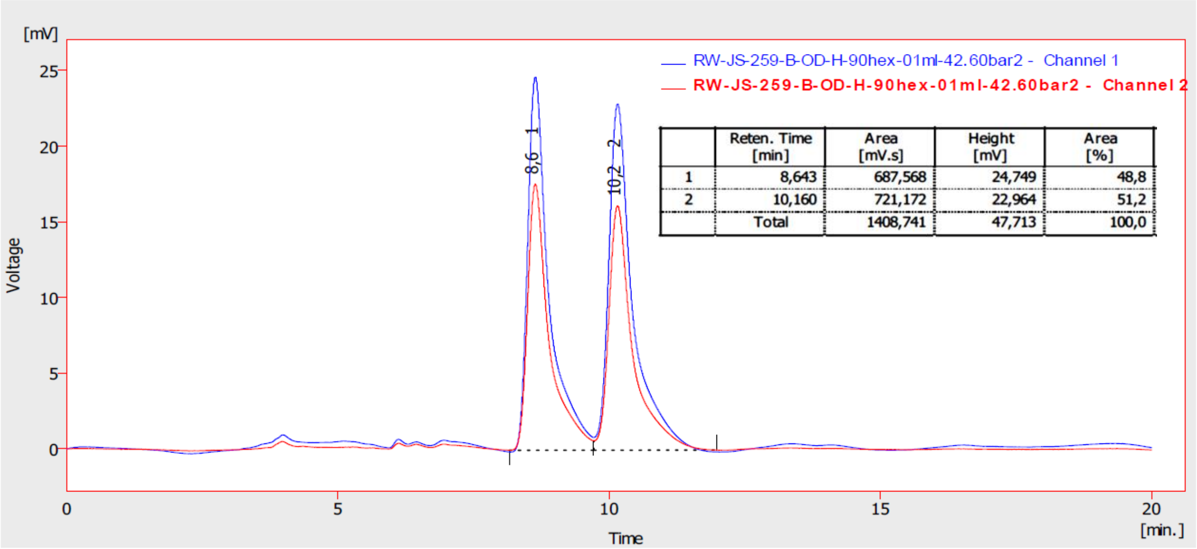

	Reten. Time [min]	Area [mV.s]	Height [mV]	Area [%]
1	8,643	687,568	24,749	48,8
2	10,160	721,172	22,964	51,2
	Total	1408,741	47,713	100,0

1t:

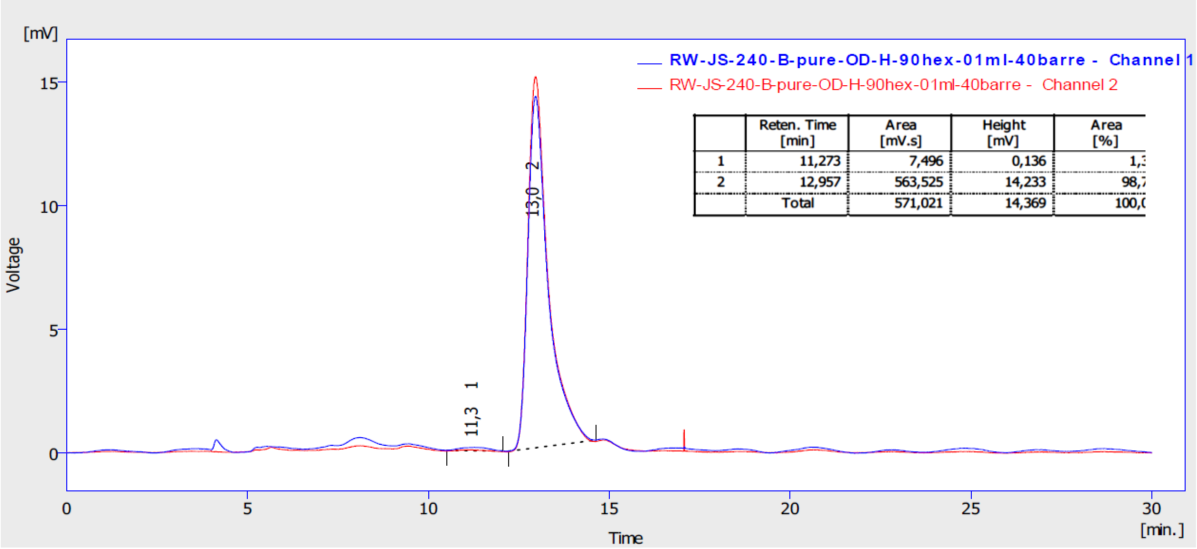

1t: racemates

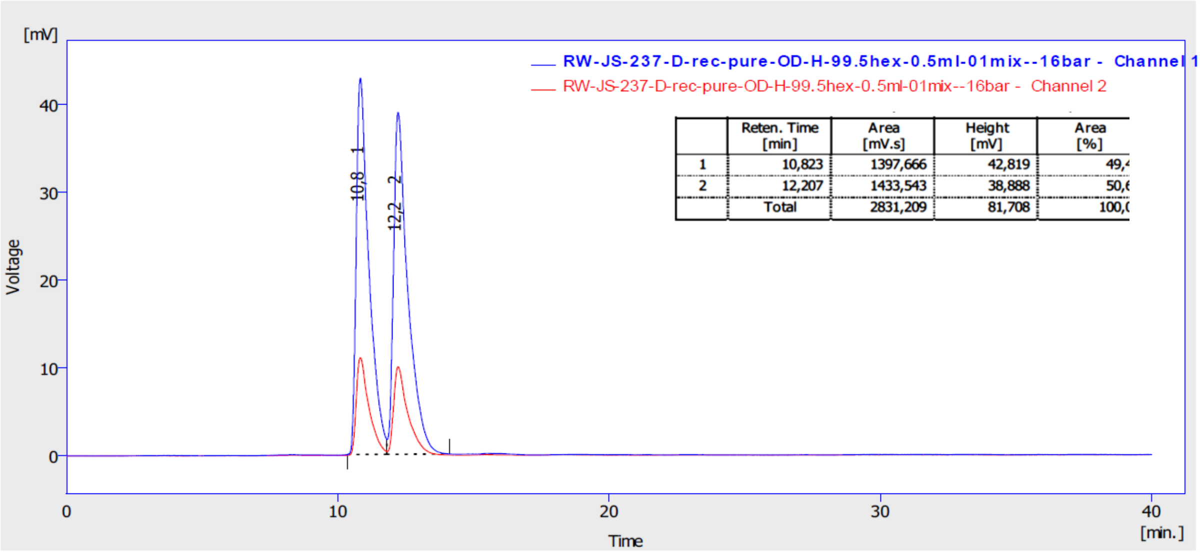

1u:

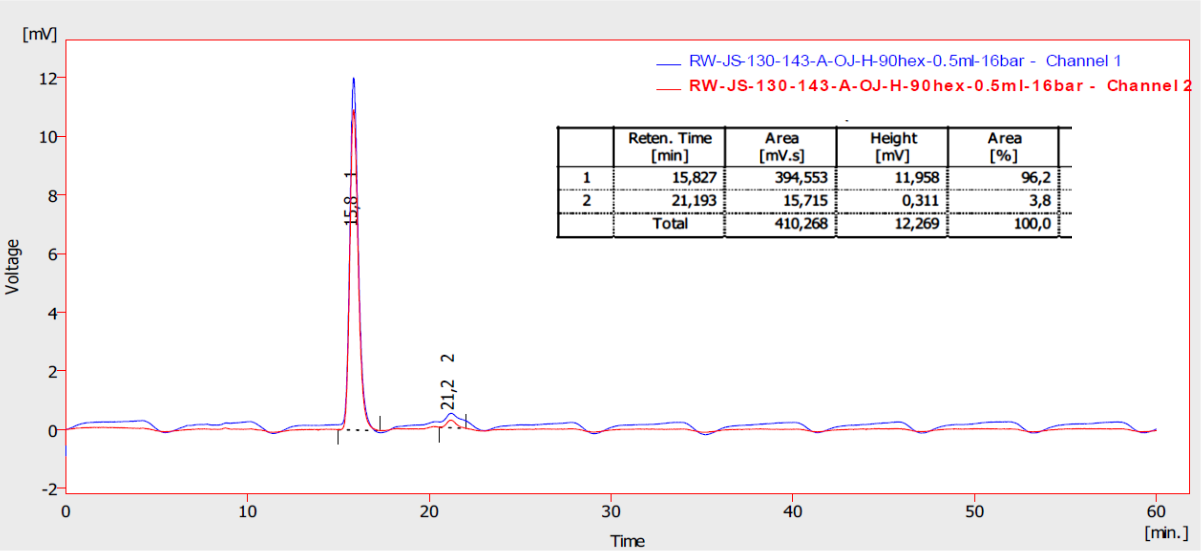

	Reten. Time [min]	Area [mV.s]	Height [mV]	Area [%]
1	15,827	394,553	11,958	96,2
2	21,193	15,715	0,311	3,8
	Total	410,268	12,269	100,0

1u: racemates

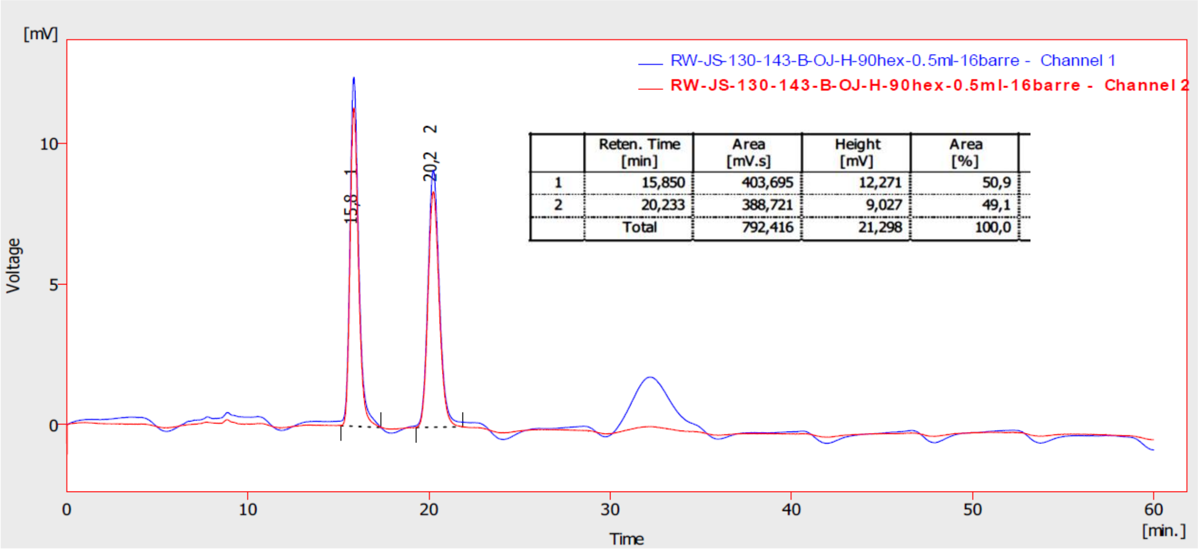

	Reten. Time [min]	Area [mV.s]	Height [mV]	Area [%]
1	15,850	403,695	12,271	50,9
2	20,233	388,721	9,027	49,1
	Total	792,416	21,298	100,0

1v:

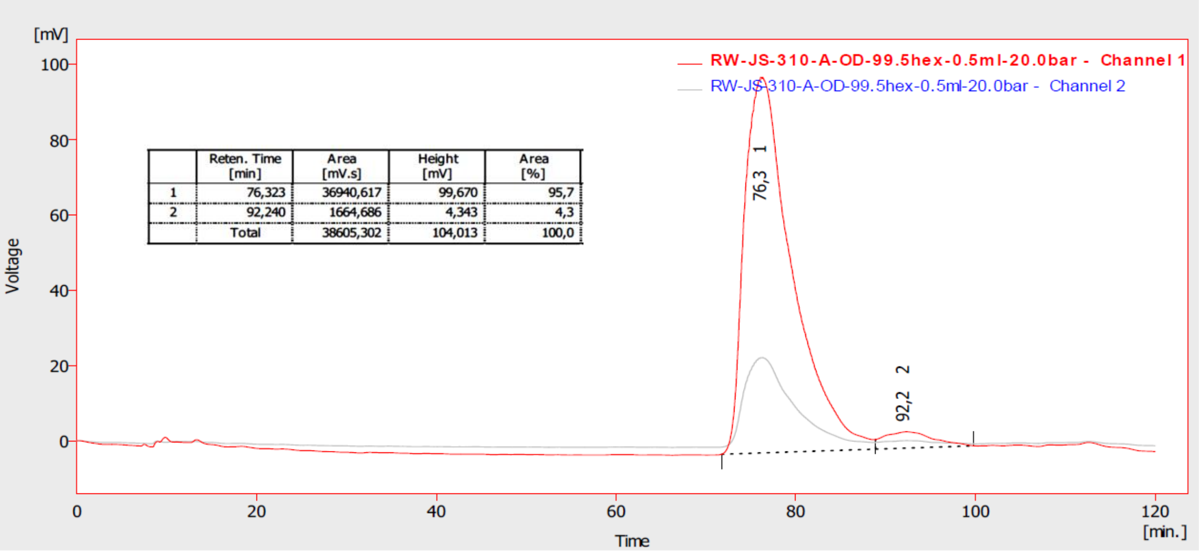

1v: racemates

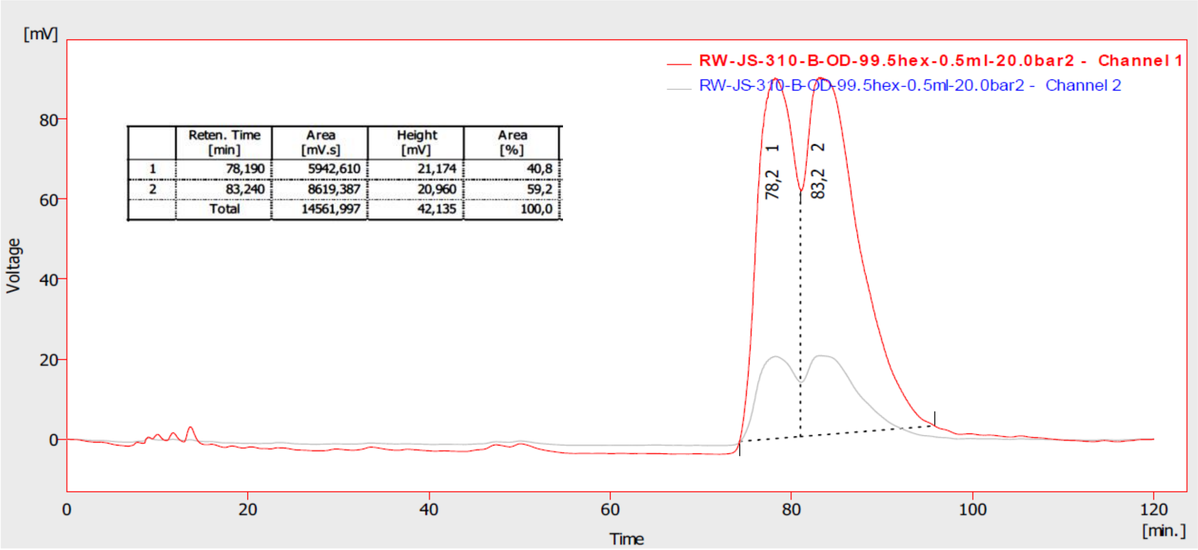

2a:

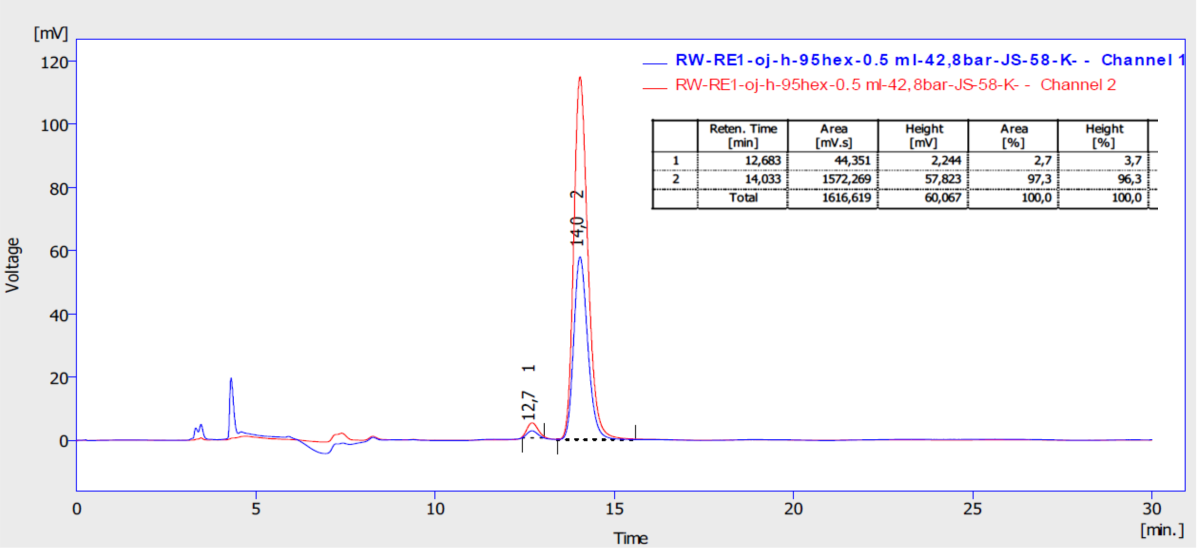

	Reten. Time [min]	Area [mV.s]	Height [mV]	Area [%]	Height [%]
1	12,683	44,351	2,244	2,7	3,7
2	14,033	1572,269	57,823	97,3	96,3
	Total	1616,619	60,067	100,0	100,0

2a: racemates

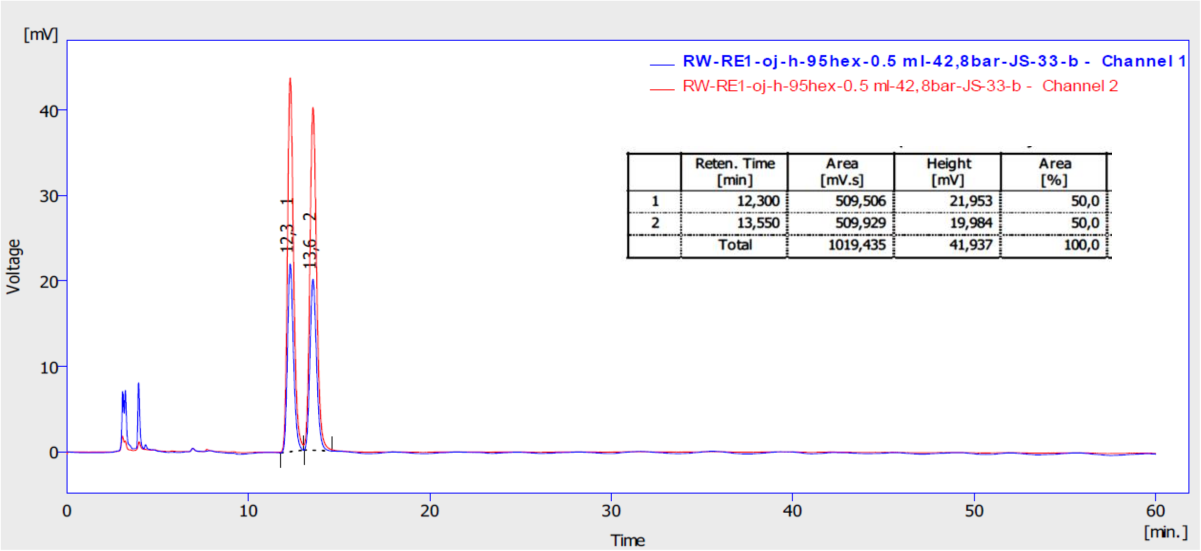

	Reten. Time [min]	Area [mV.s]	Height [mV]	Area [%]
1	12,300	509,506	21,953	50,0
2	13,550	509,929	19,984	50,0
	Total	1019,435	41,937	100,0

2b:

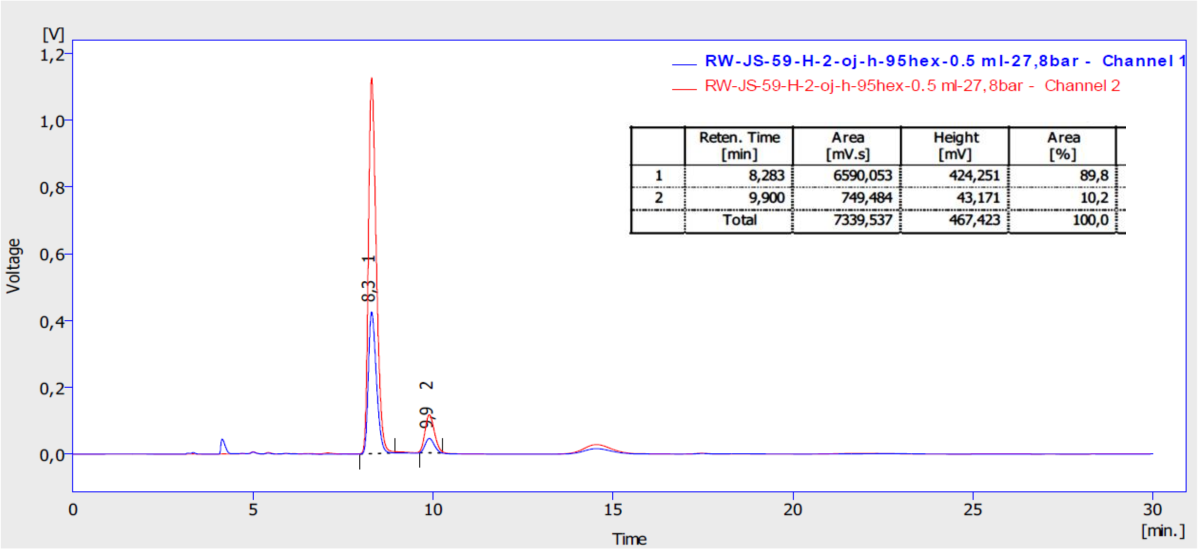

	Reten. Time [min]	Area [mV.s]	Height [mV]	Area [%]
1	8,283	6590,053	424,251	89,8
2	9,900	749,484	43,171	10,2
	Total	7339,537	467,423	100,0

2b: racemates

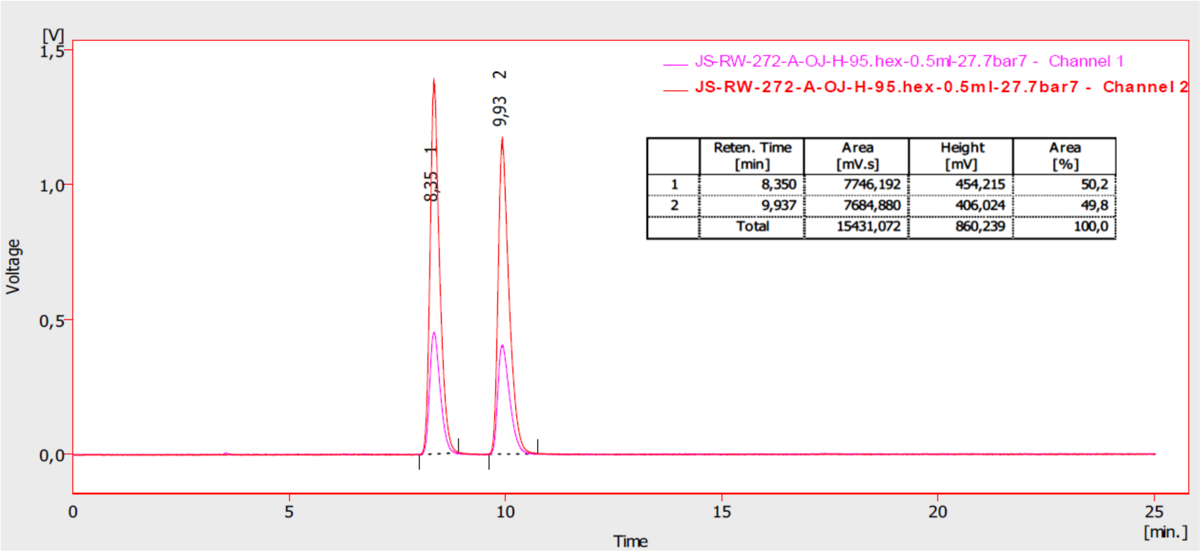

	Reten. Time [min]	Area [mV.s]	Height [mV]	Area [%]
1	8,350	7746,192	454,215	50,2
2	9,937	7684,880	406,024	49,8
	Total	15431,072	860,239	100,0

2c:

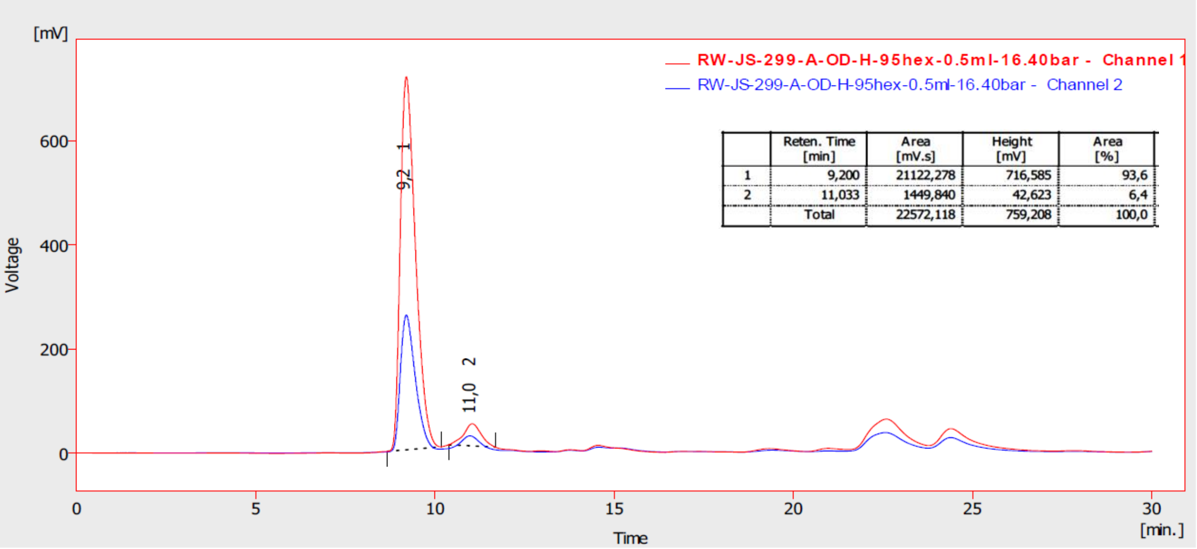

2c: racemates

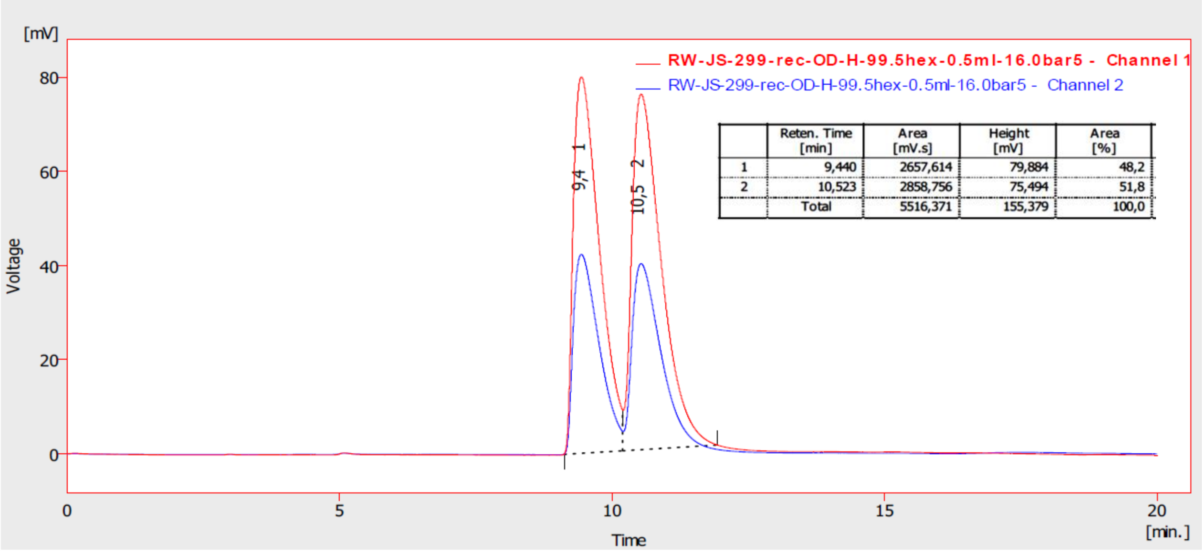

2d:

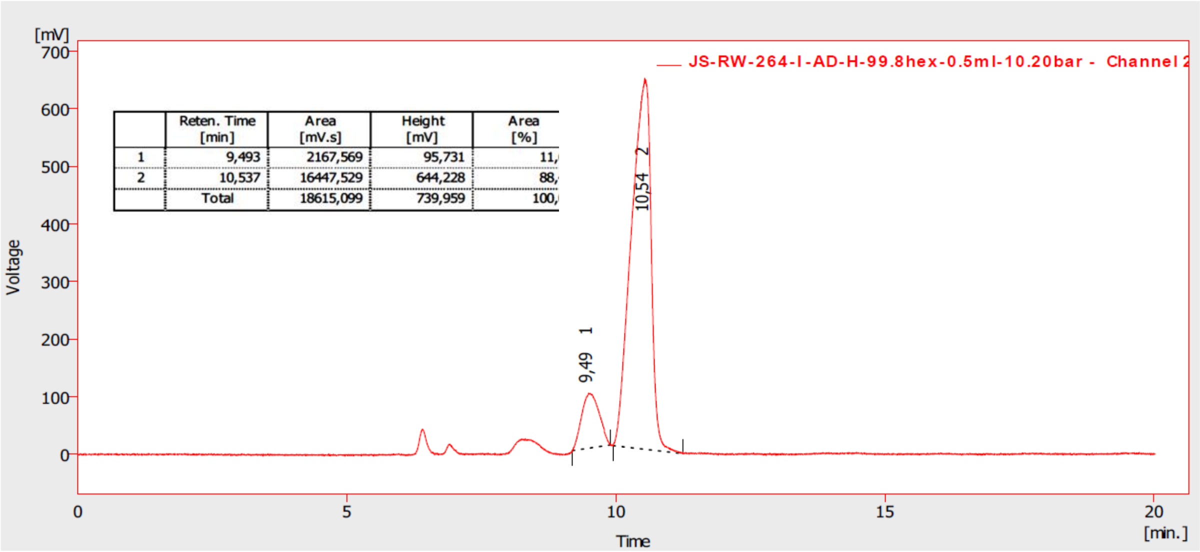

2d: racemates

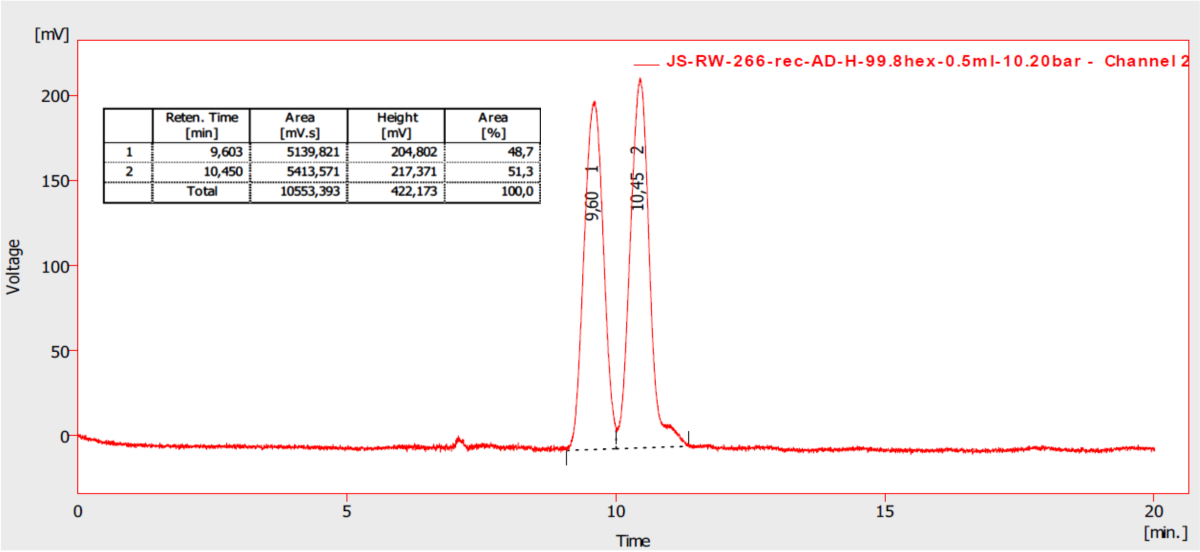

2e:

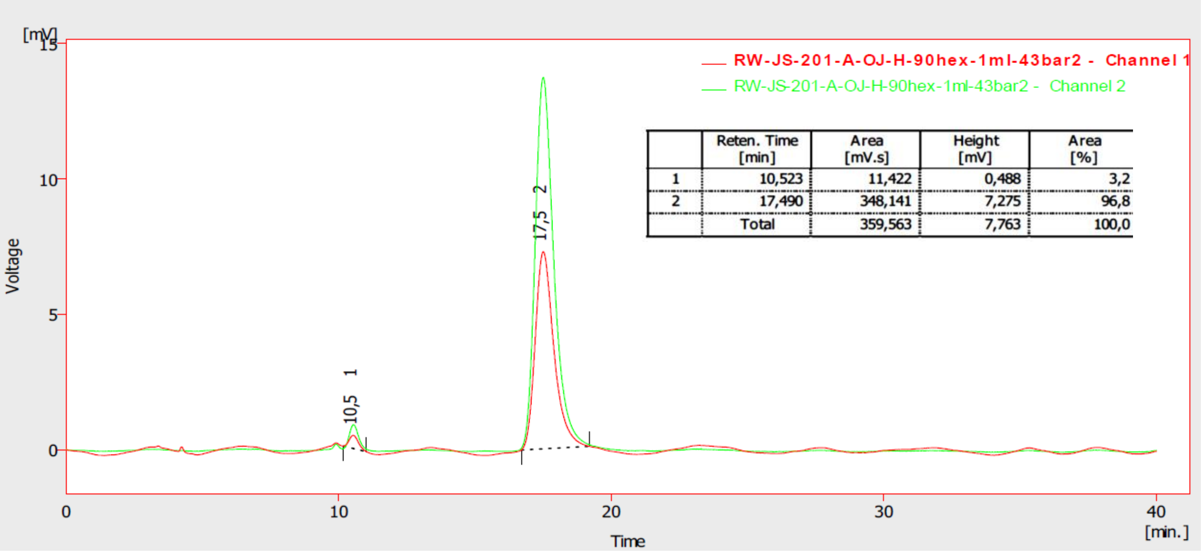

	Reten. Time [min]	Area [mV.s]	Height [mV]	Area [%]
1	10,523	11,422	0,488	3,2
2	17,490	348,141	7,275	96,8
	Total	359,563	7,763	100,0

2e: racemates

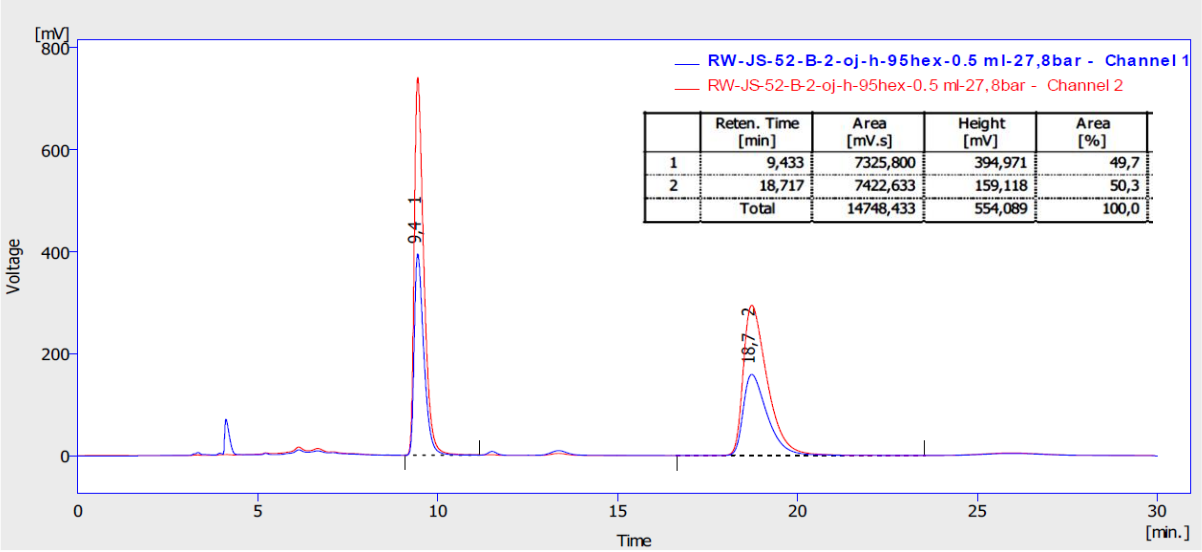

	Reten. Time [min]	Area [mV.s]	Height [mV]	Area [%]
1	9,433	7325,800	394,971	49,7
2	18,717	7422,633	159,118	50,3
	Total	14748,433	554,089	100,0

2f

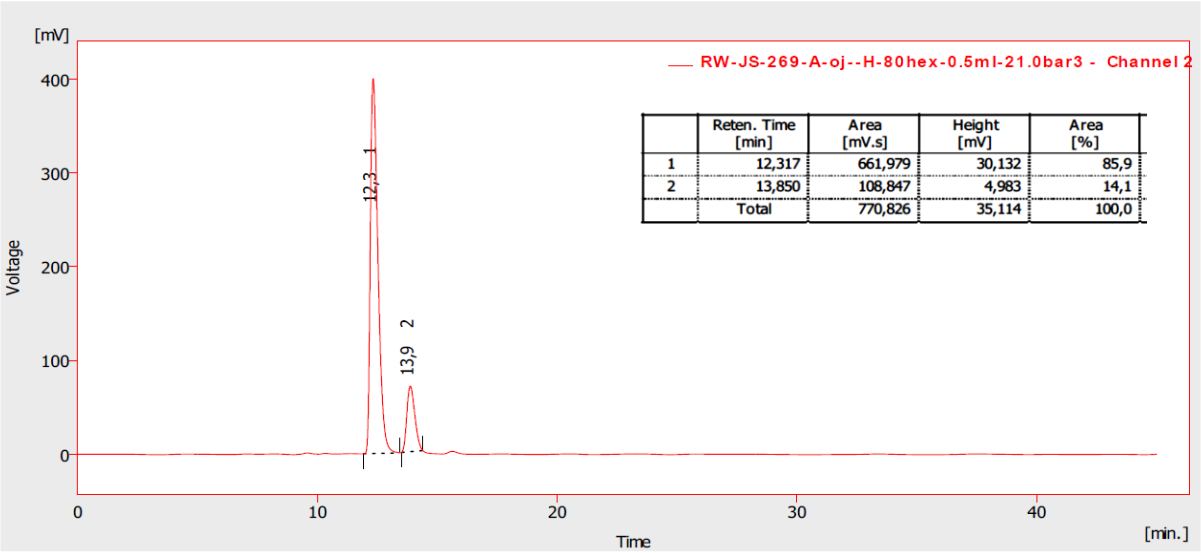

	Reten. Time [min]	Area [mV.s]	Height [mV]	Area [%]
1	12,317	661,979	30,132	85,9
2	13,850	108,847	4,983	14,1
	Total	770,826	35,114	100,0

2f: racemates

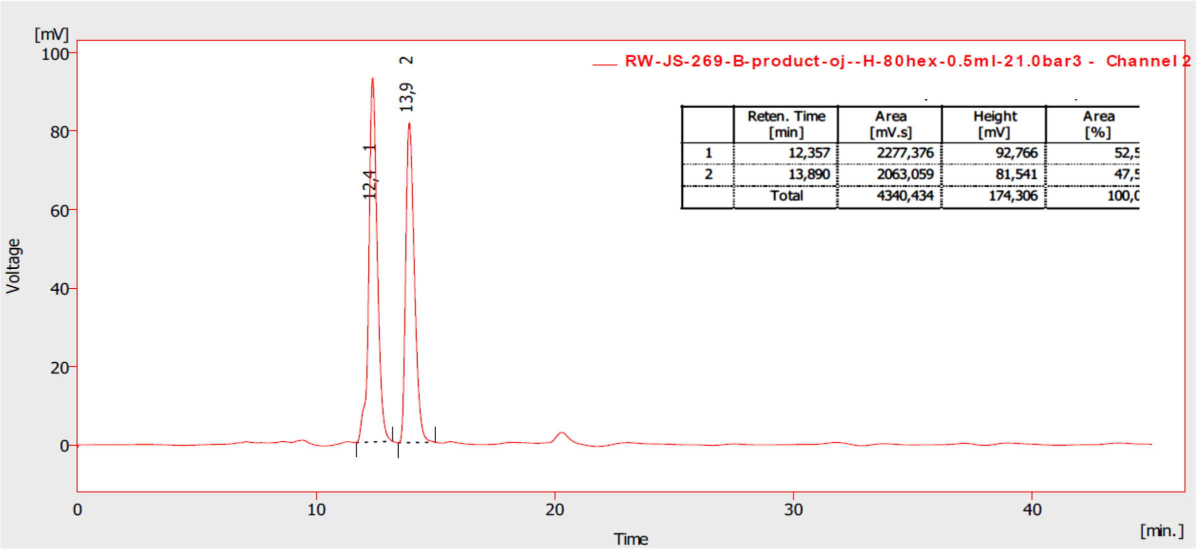

	Reten. Time [min]	Area [mV.s]	Height [mV]	Area [%]
1	12,357	2277,376	92,766	52,[illegible]
2	13,890	2063,059	81,541	47,[illegible]
	Total	4340,434	174,306	100,[illegible]

2g:

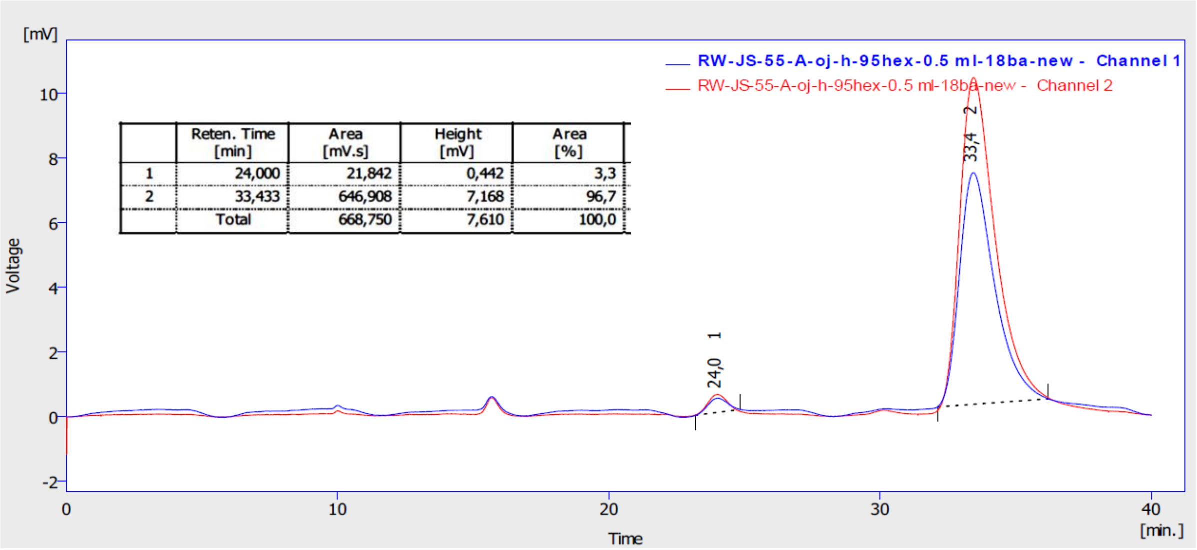

	Reten. Time [min]	Area [mV.s]	Height [mV]	Area [%]
1	24,000	21,842	0,442	3,3
2	33,433	646,908	7,168	96,7
	Total	668,750	7,610	100,0

2g: racemates

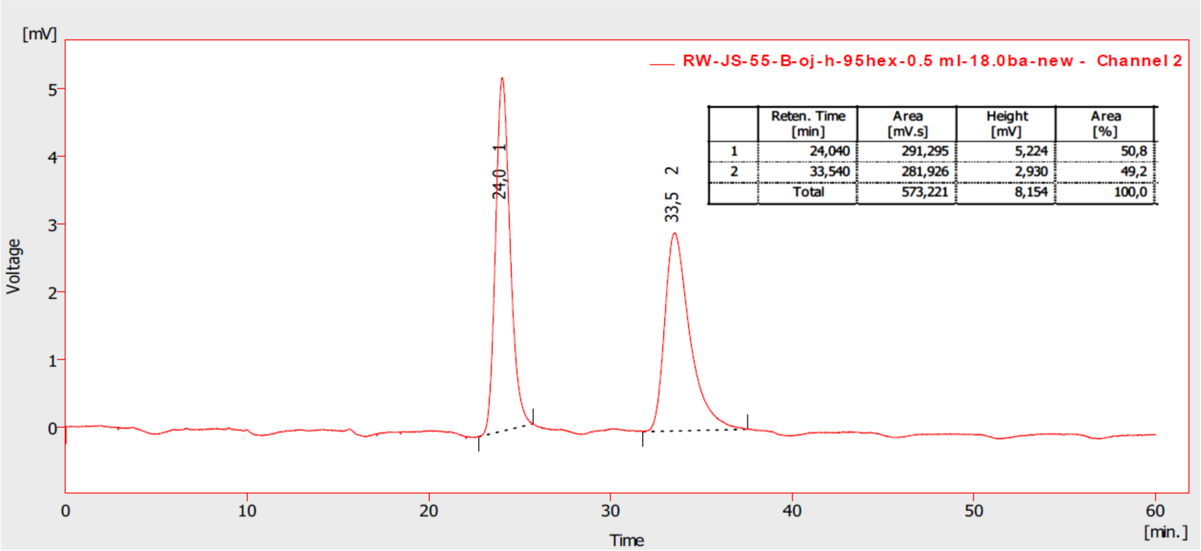

	Reten. Time [min]	Area [mV.s]	Height [mV]	Area [%]
1	24,040	291,295	5,224	50,8
2	33,540	281,926	2,930	49,2
	Total	573,221	8,154	100,0

2h

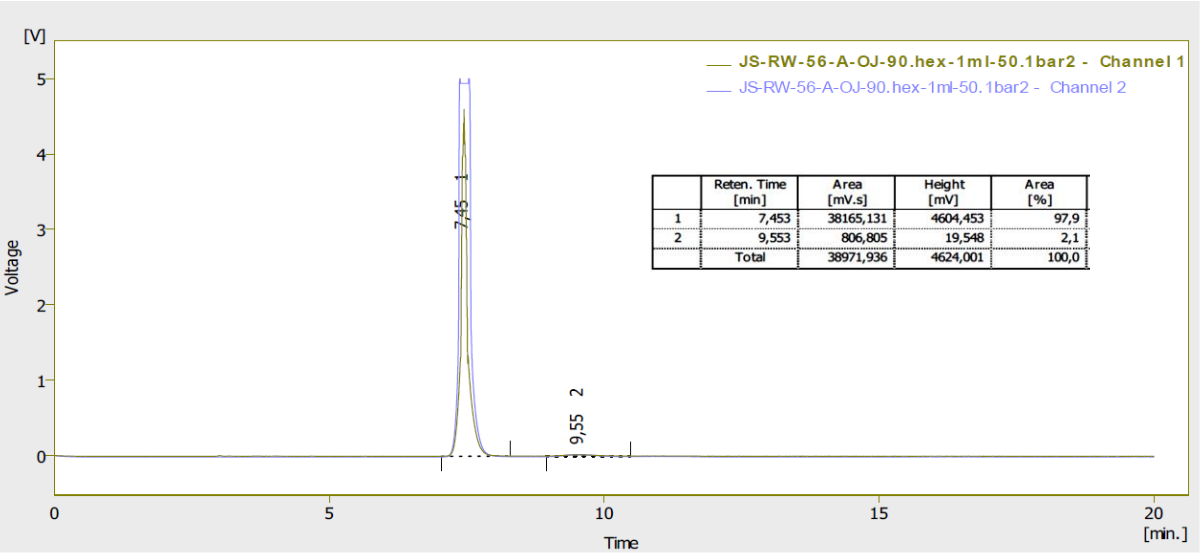

	Reten. Time [min]	Area [mV.s]	Height [mV]	Area [%]
1	7,453	38165,131	4604,453	97,9
2	9,553	806,805	19,548	2,1
	Total	38971,936	4624,001	100,0

2h: racemates

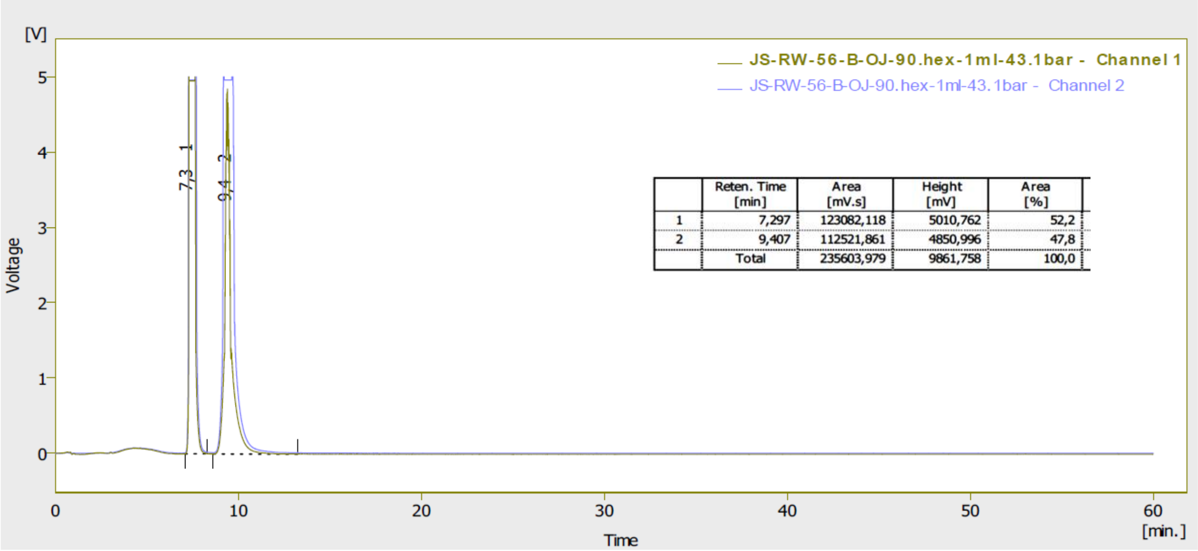

	Reten. Time [min]	Area [mV.s]	Height [mV]	Area [%]
1	7,297	123082,118	5010,762	52,2
2	9,407	112521,861	4850,996	47,8
	Total	235603,979	9861,758	100,0

2i:

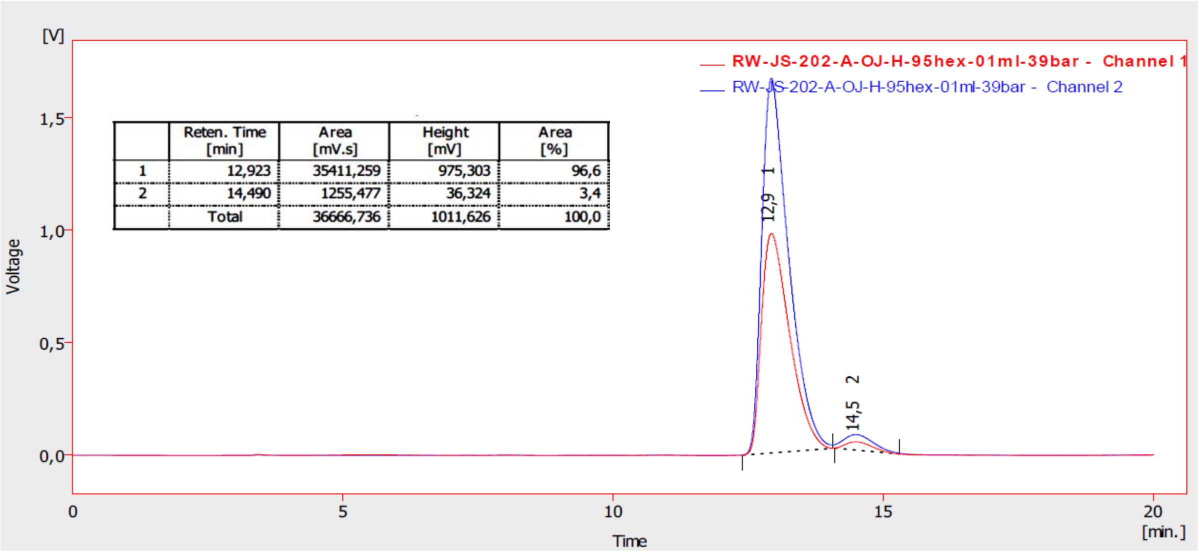

	Reten. Time [min]	Area [mV.s]	Height [mV]	Area [%]
1	12,923	35411,259	975,303	96,6
2	14,490	1255,477	36,324	3,4
	Total	36666,736	1011,626	100,0

2i: racemates

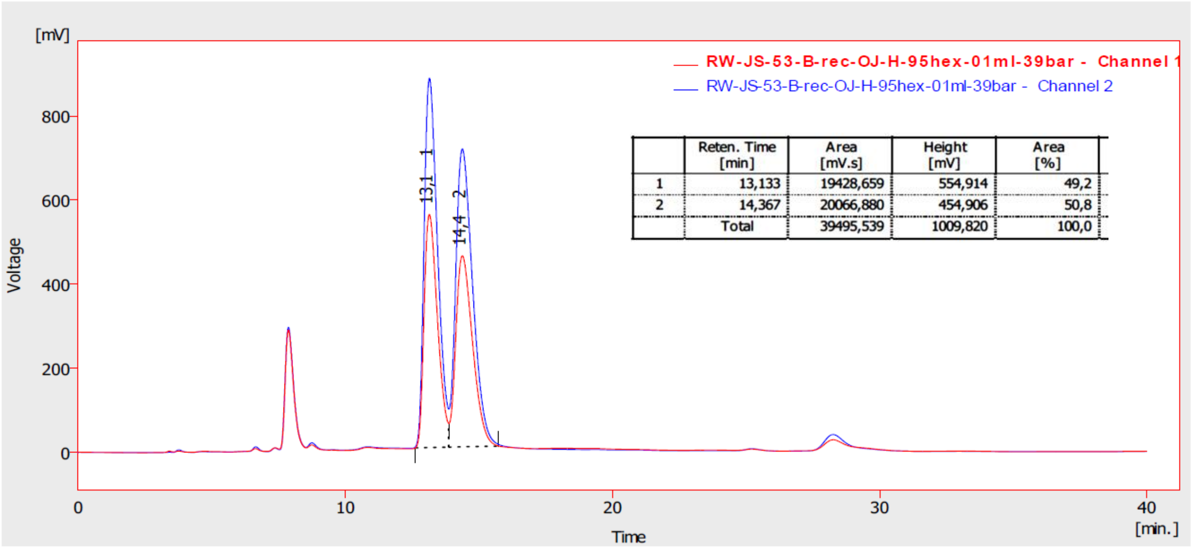

	Reten. Time [min]	Area [mV.s]	Height [mV]	Area [%]
1	13,133	19428,659	554,914	49,2
2	14,367	20066,880	454,906	50,8
	Total	39495,539	1009,820	100,0

2j:

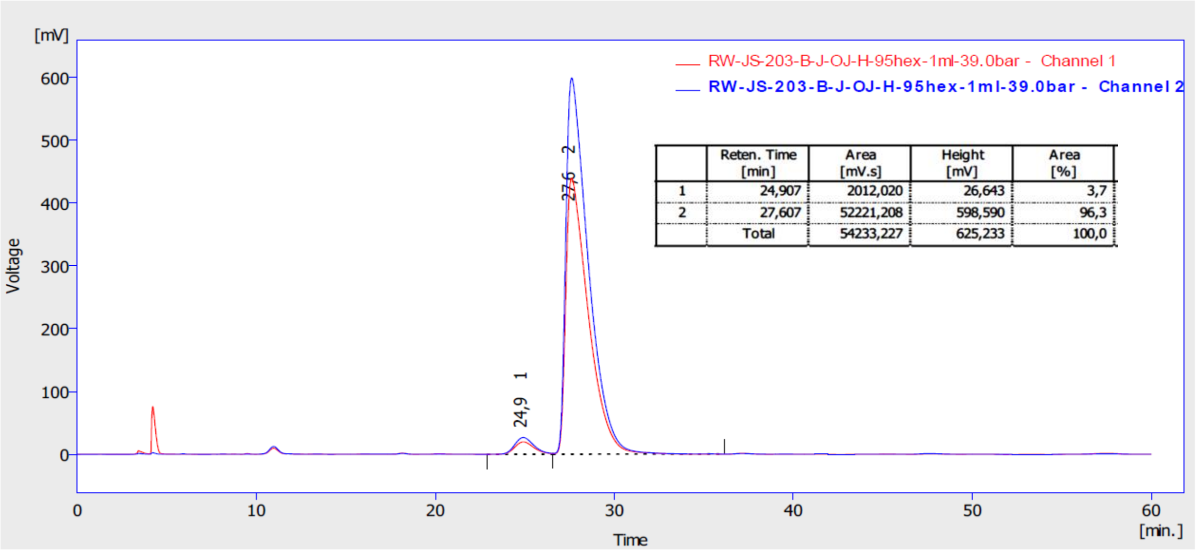

	Reten. Time [min]	Area [mV.s]	Height [mV]	Area [%]
1	24,907	2012,020	26,643	3,7
2	27,607	52221,208	598,590	96,3
	Total	54233,227	625,233	100,0

2j: racemates

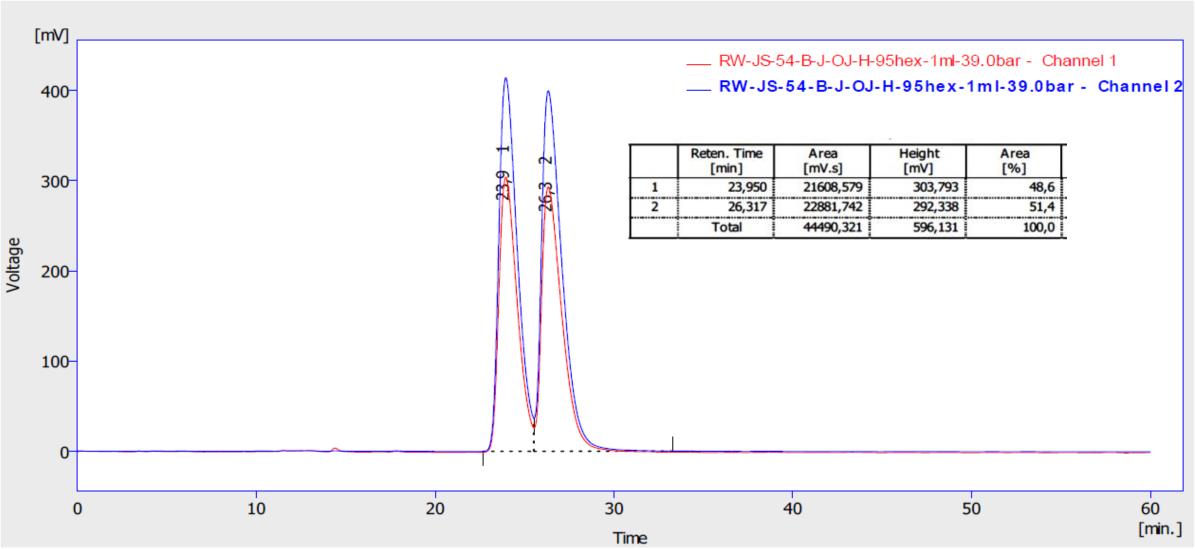

	Reten. Time [min]	Area [mV.s]	Height [mV]	Area [%]
1	23,950	21608,579	303,793	48,6
2	26,317	22881,742	292,338	51,4
	Total	44490,321	596,131	100,0

2k:

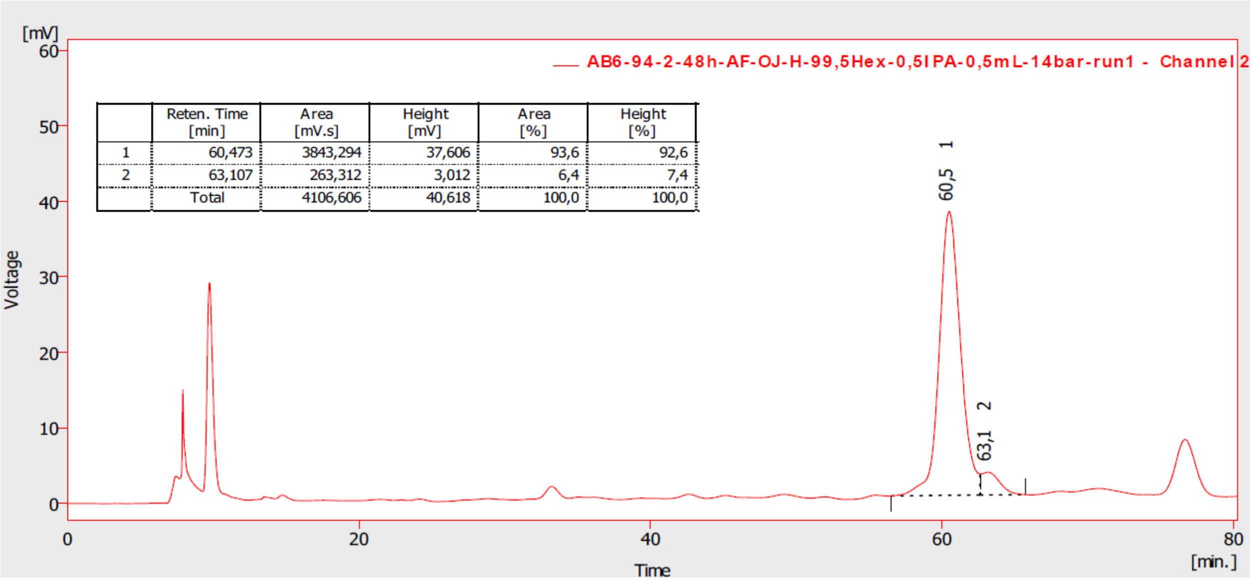

	Reten. Time [min]	Area [mV.s]	Height [mV]	Area [%]	Height [%]
1	60,473	3843,294	37,606	93,6	92,6
2	63,107	263,312	3,012	6,4	7,4
	Total	4106,606	40,618	100,0	100,0

2k: racemates

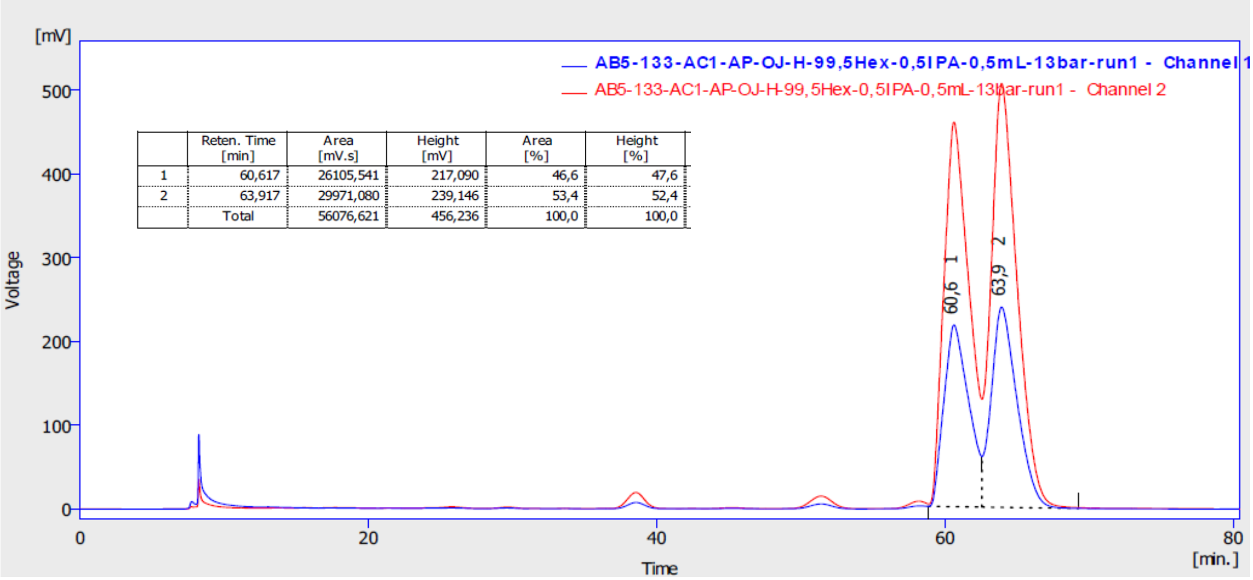

	Reten. Time [min]	Area [mV.s]	Height [mV]	Area [%]	Height [%]
1	60,617	26105,541	217,090	46,6	47,6
2	63,917	29971,080	239,146	53,4	52,4
	Total	56076,621	456,236	100,0	100,0

2l:

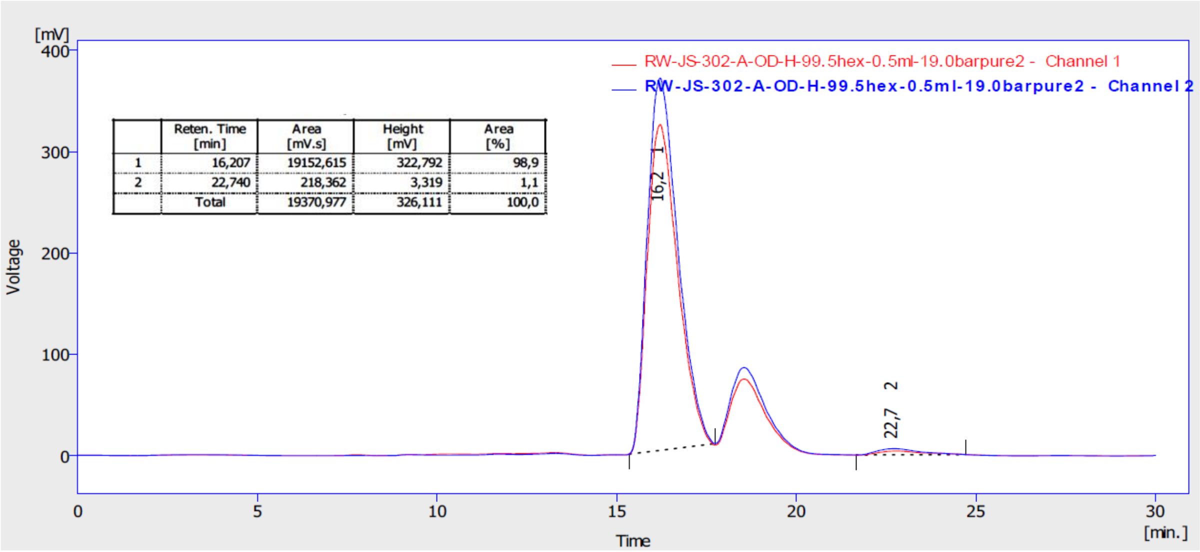

	Reten. Time [min]	Area [mV.s]	Height [mV]	Area [%]
1	16,207	19152,615	322,792	98,9
2	22,740	218,362	3,319	1,1
	Total	19370,977	326,111	100,0

2l: racemates

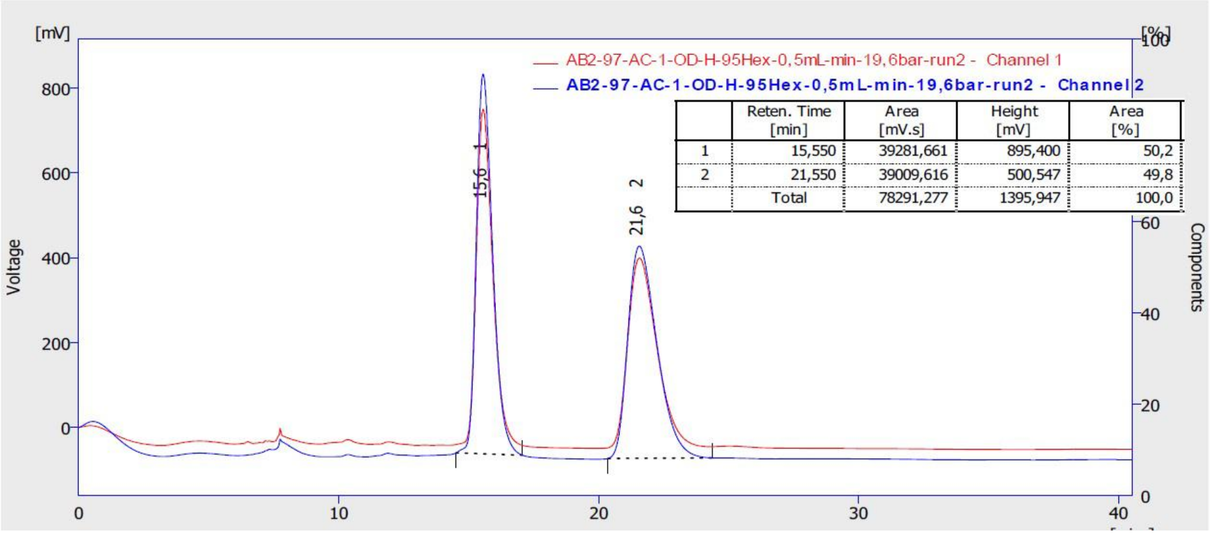

	Reten. Time [min]	Area [mV.s]	Height [mV]	Area [%]
1	15,550	39281,661	895,400	50,2
2	21,550	39009,616	500,547	49,8
	Total	78291,277	1395,947	100,0

2m:

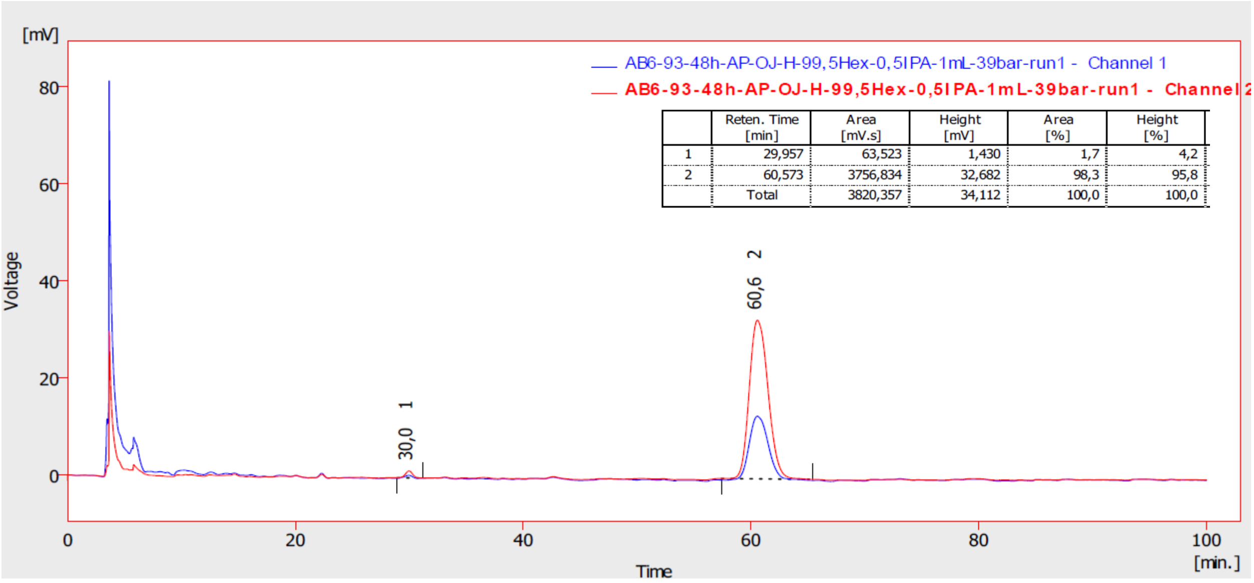

	Reten. Time [min]	Area [mV.s]	Height [mV]	Area [%]	Height [%]
1	29,957	63,523	1,430	1,7	4,2
2	60,573	3756,834	32,682	98,3	95,8
	Total	3820,357	34,112	100,0	100,0

2m: racemates

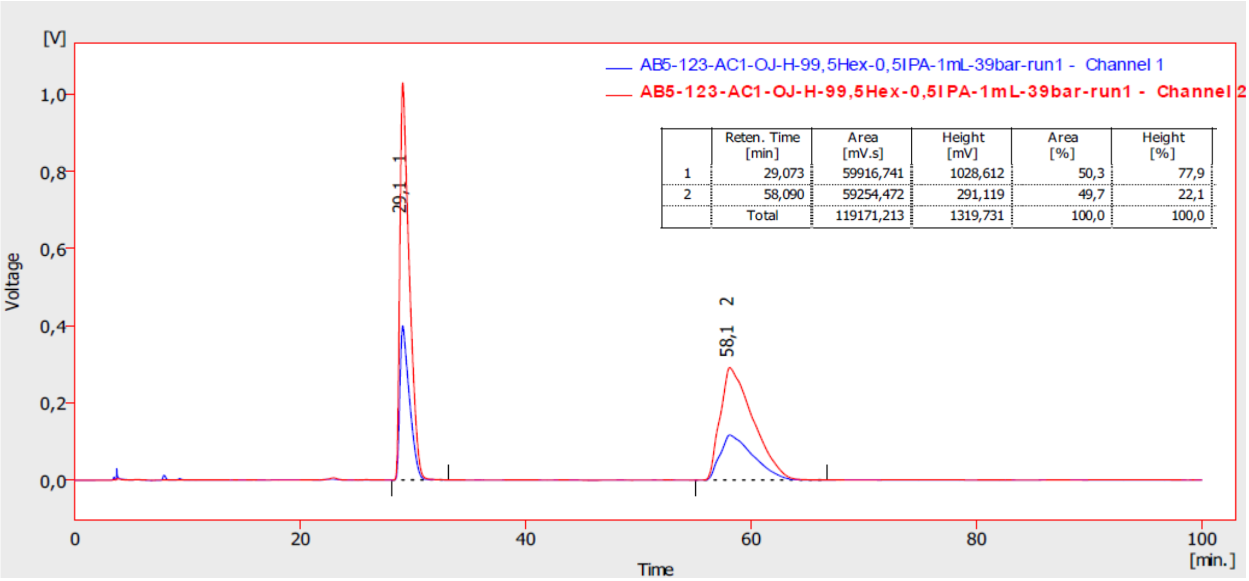

	Reten. Time [min]	Area [mV.s]	Height [mV]	Area [%]	Height [%]
1	29,073	59916,741	1028,612	50,3	77,9
2	58,090	59254,472	291,119	49,7	22,1
	Total	119171,213	1319,731	100,0	100,0

2n:

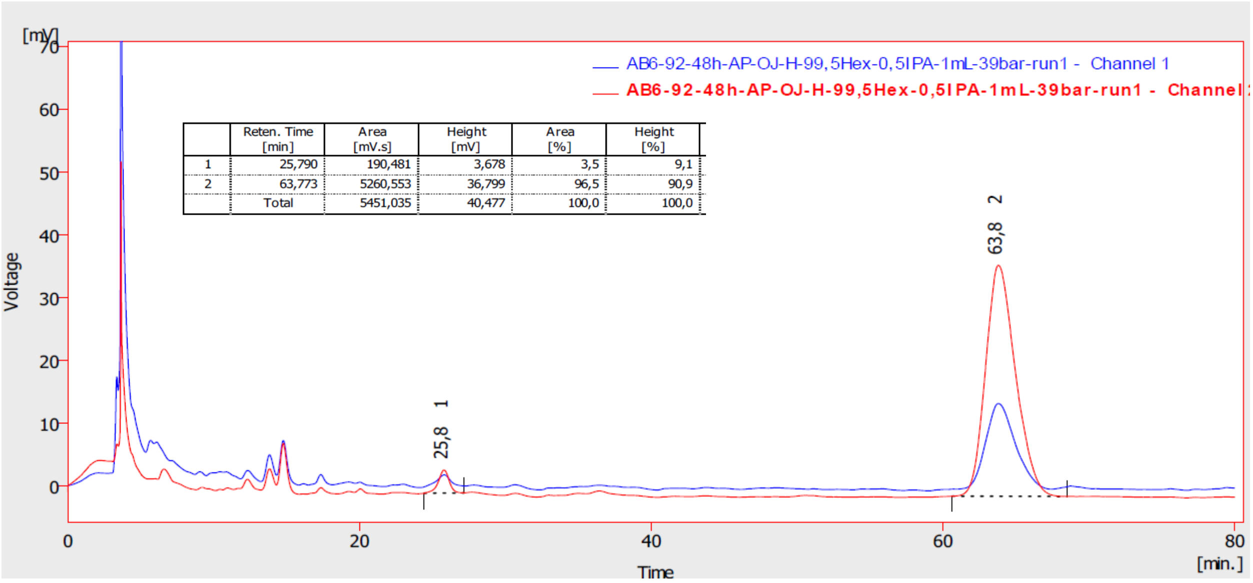

	Reten. Time [min]	Area [mV.s]	Height [mV]	Area [%]	Height [%]
1	25,790	190,481	3,678	3,5	9,1
2	63,773	5260,553	36,799	96,5	90,9
	Total	5451,035	40,477	100,0	100,0

2n: racemates

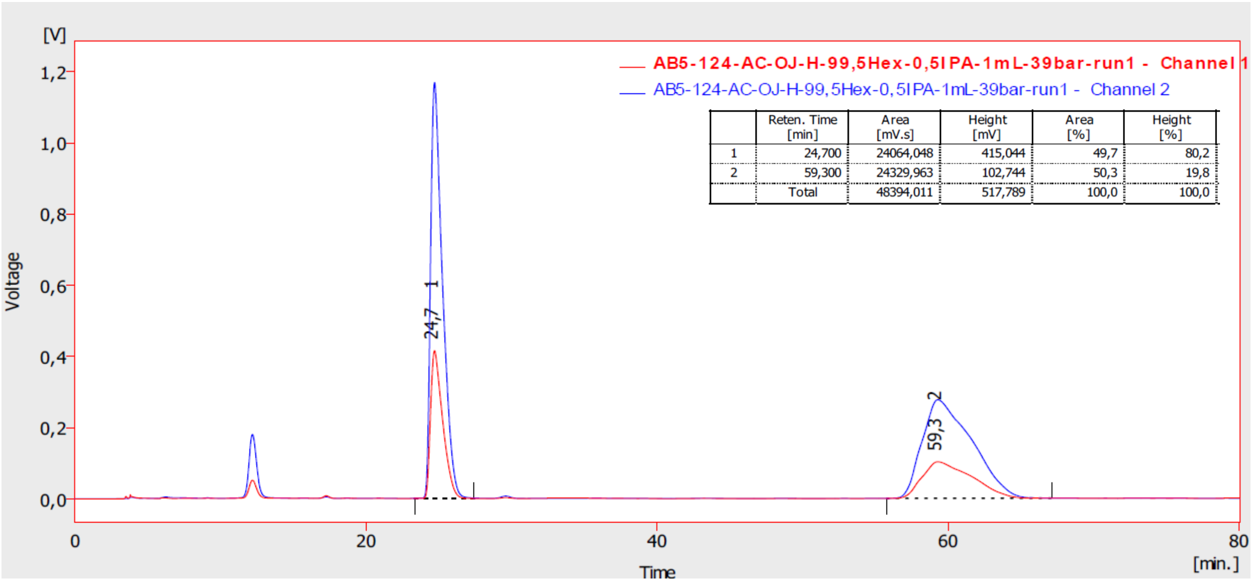

	Reten. Time [min]	Area [mV.s]	Height [mV]	Area [%]	Height [%]
1	24,700	24064,048	415,044	49,7	80,2
2	59,300	24329,963	102,744	50,3	19,8
	Total	48394,011	517,789	100,0	100,0

2o:

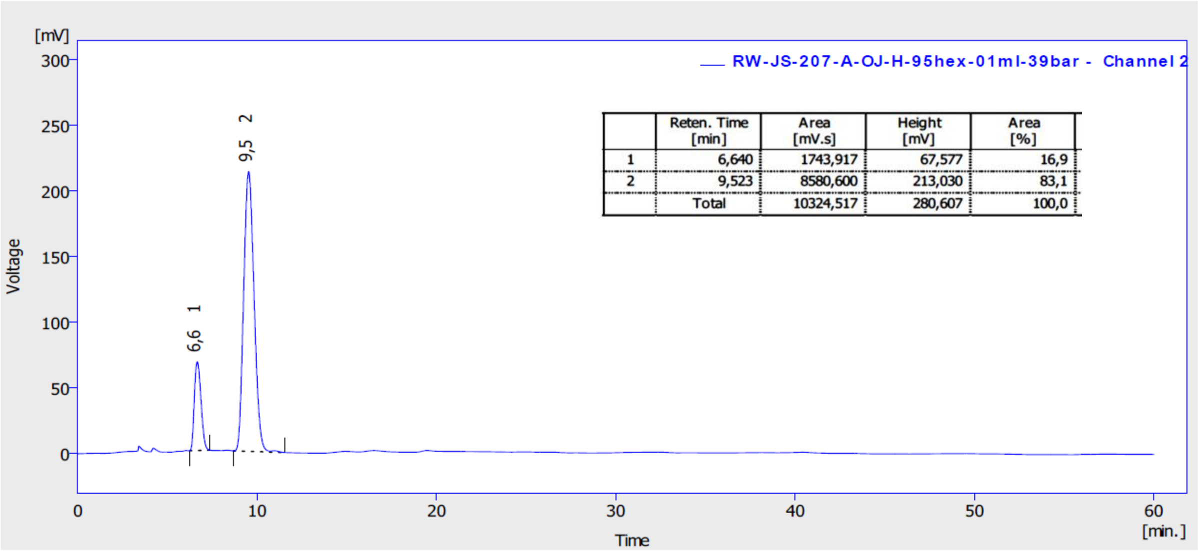

	Reten. Time [min]	Area [mV.s]	Height [mV]	Area [%]
1	6,640	1743,917	67,577	16,9
2	9,523	8580,600	213,030	83,1
	Total	10324,517	280,607	100,0

2o: racemates

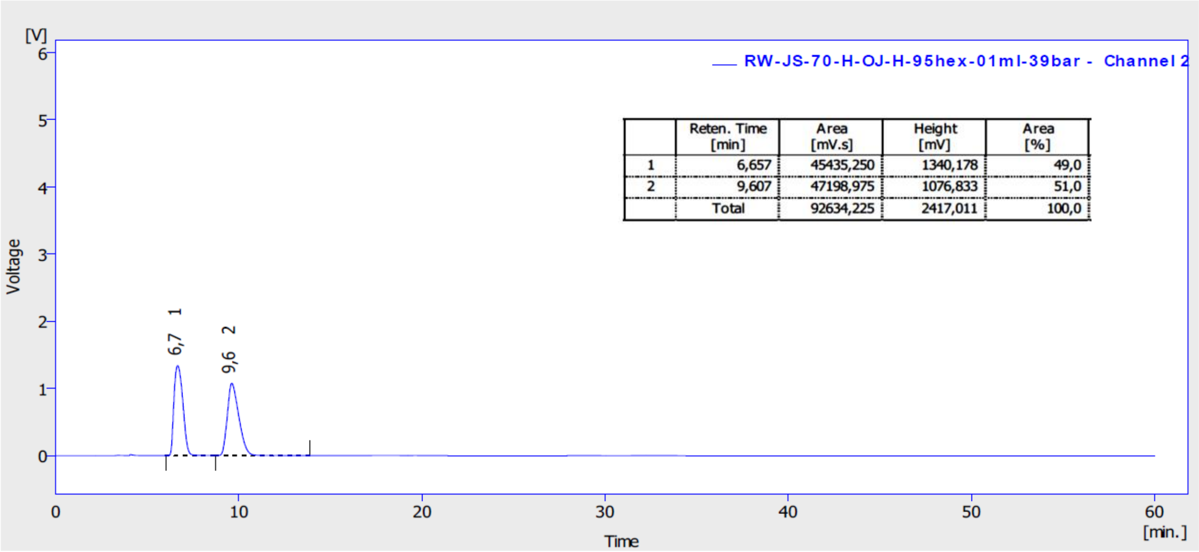

	Reten. Time [min]	Area [mV.s]	Height [mV]	Area [%]
1	6,657	45435,250	1340,178	49,0
2	9,607	47198,975	1076,833	51,0
	Total	92634,225	2417,011	100,0

2p:

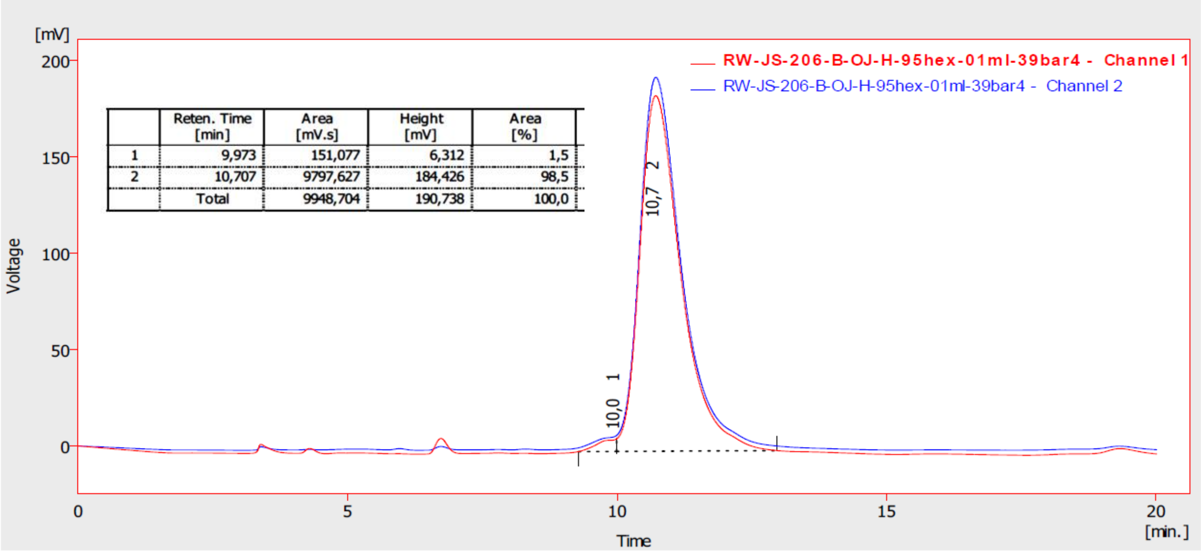

	Reten. Time [min]	Area [mV.s]	Height [mV]	Area [%]
1	9,973	151,077	6,312	1,5
2	10,707	9797,627	184,426	98,5
	Total	9948,704	190,738	100,0

2p: racemates

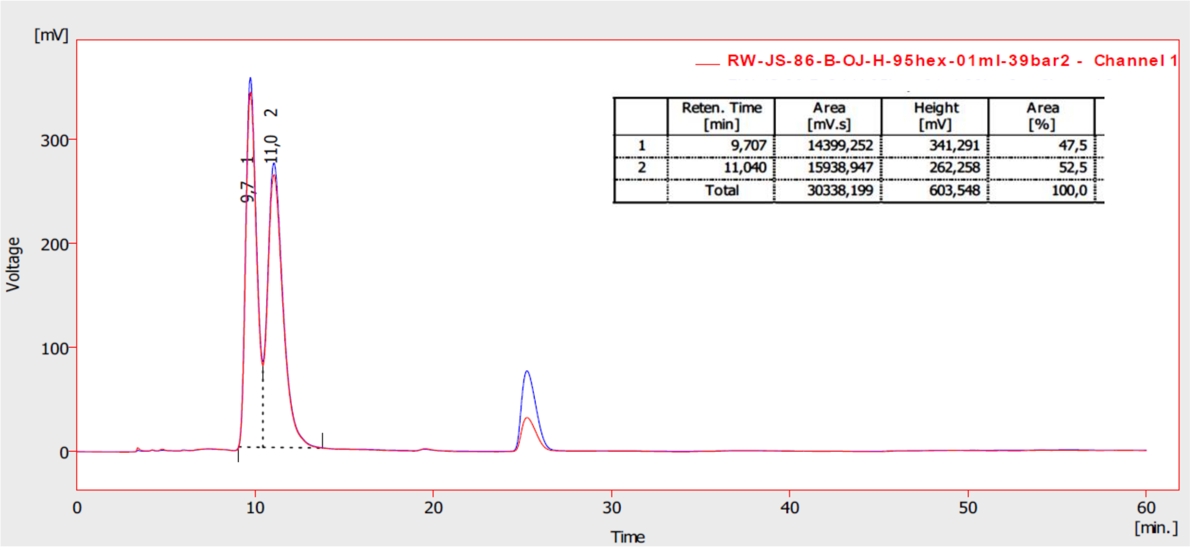

	Reten. Time [min]	Area [mV.s]	Height [mV]	Area [%]
1	9,707	14399,252	341,291	47,5
2	11,040	15938,947	262,258	52,5
	Total	30338,199	603,548	100,0

2q:

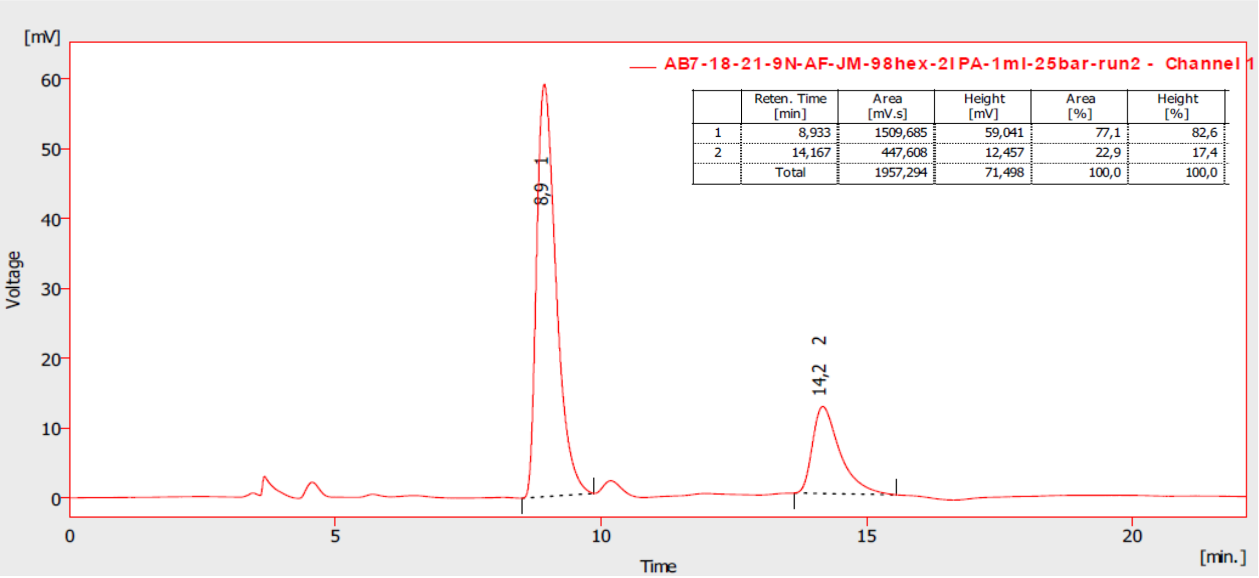

	Reten. Time [min]	Area [mV.s]	Height [mV]	Area [%]	Height [%]
1	8,933	1509,685	59,041	77,1	82,6
2	14,167	447,608	12,457	22,9	17,4
	Total	1957,294	71,498	100,0	100,0

2q: racemates

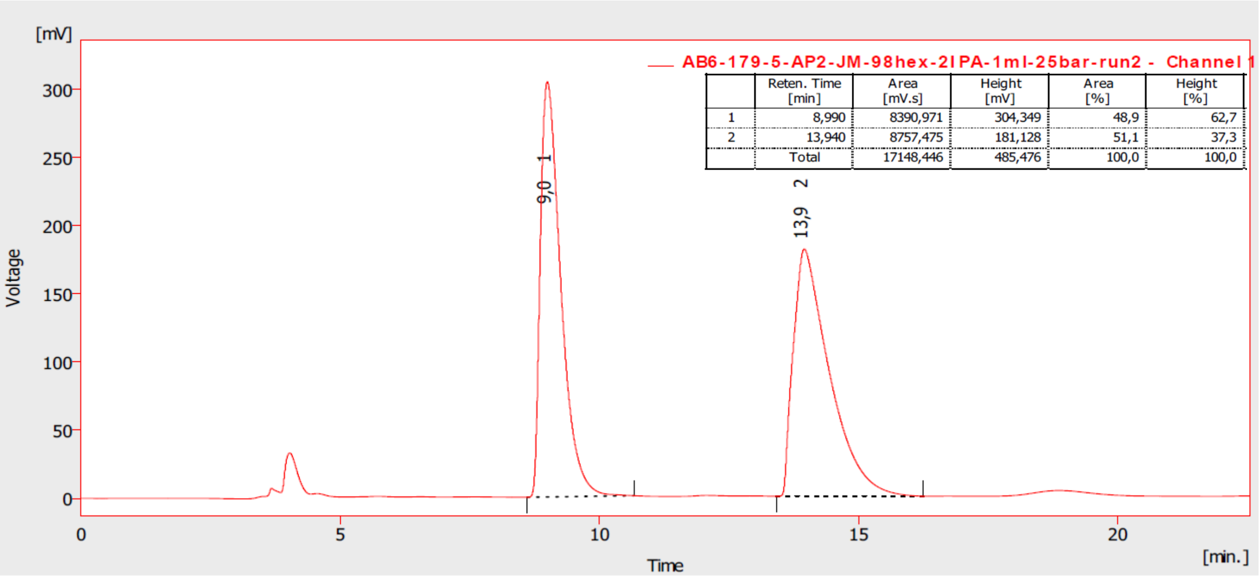

	Reten. Time [min]	Area [mV.s]	Height [mV]	Area [%]	Height [%]
1	8,990	8390,971	304,349	48,9	62,7
2	13,940	8757,475	181,128	51,1	37,3
	Total	17148,446	485,476	100,0	100,0

2r:

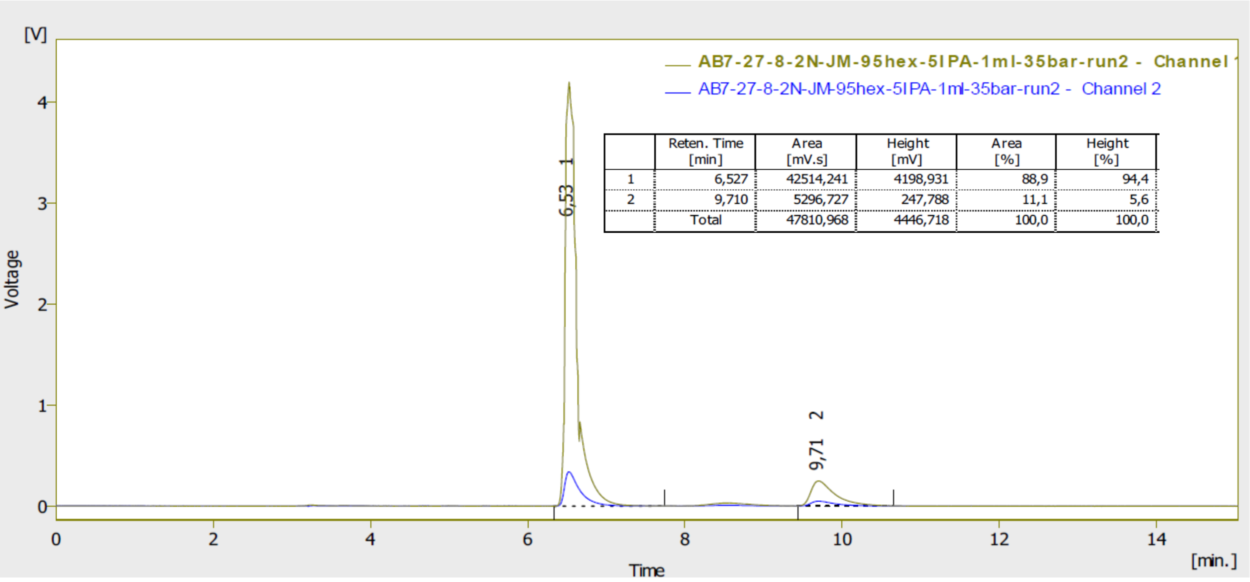

	Reten. Time [min]	Area [mV.s]	Height [mV]	Area [%]	Height [%]
1	6,527	42514,241	4198,931	88,9	94,4
2	9,710	5296,727	247,788	11,1	5,6
	Total	47810,968	4446,718	100,0	100,0

2r: racemates

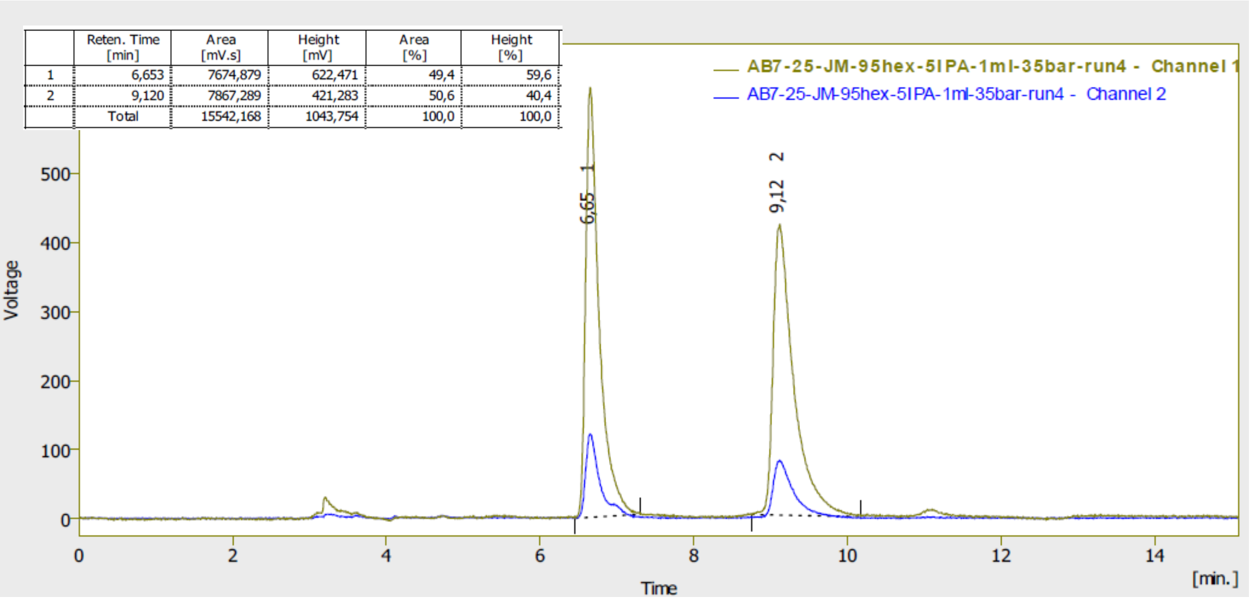

	Reten. Time [min]	Area [mV.s]	Height [mV]	Area [%]	Height [%]
1	6,653	7674,879	622,471	49,4	59,6
2	9,120	7867,289	421,283	50,6	40,4
	Total	15542,168	1043,754	100,0	100,0

2s:

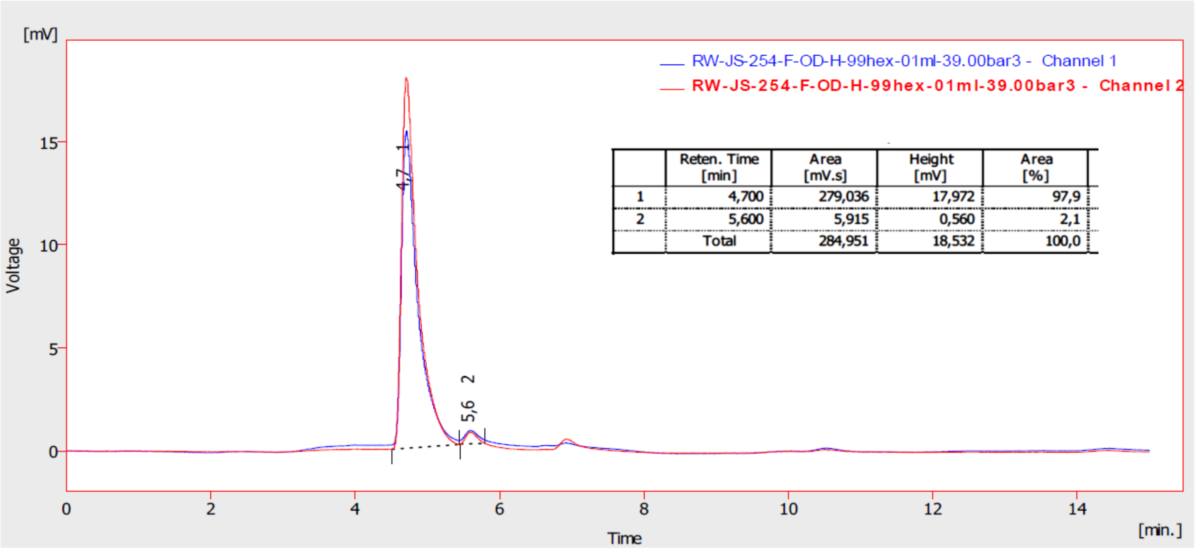

	Reten. Time [min]	Area [mV.s]	Height [mV]	Area [%]
1	4,700	279,036	17,972	97,9
2	5,600	5,915	0,560	2,1
	Total	284,951	18,532	100,0

2s: racemates

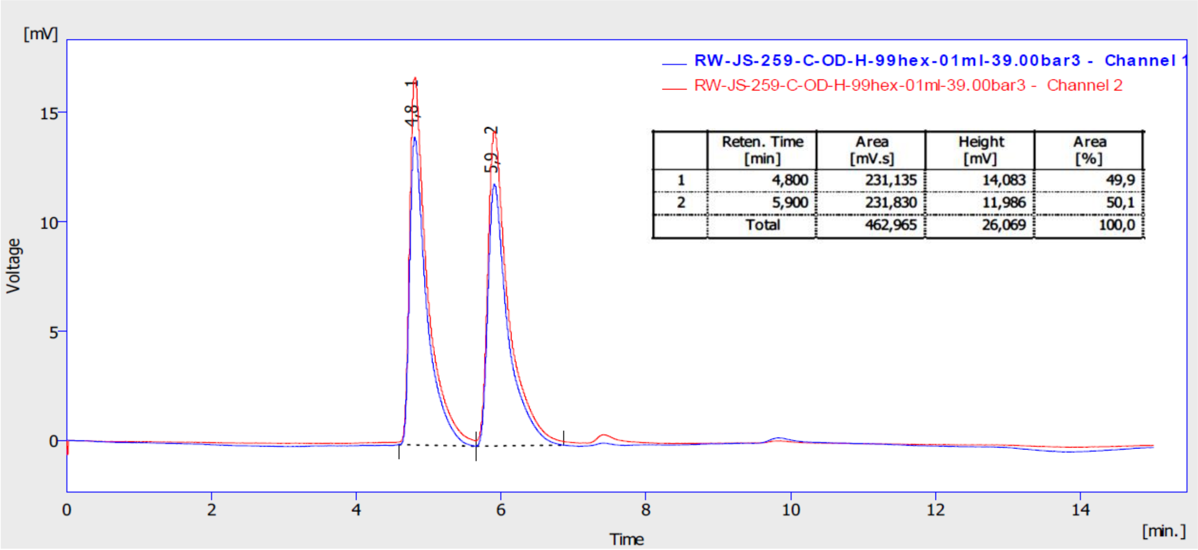

	Reten. Time [min]	Area [mV.s]	Height [mV]	Area [%]
1	4,800	231,135	14,083	49,9
2	5,900	231,830	11,986	50,1
	Total	462,965	26,069	100,0

2t:

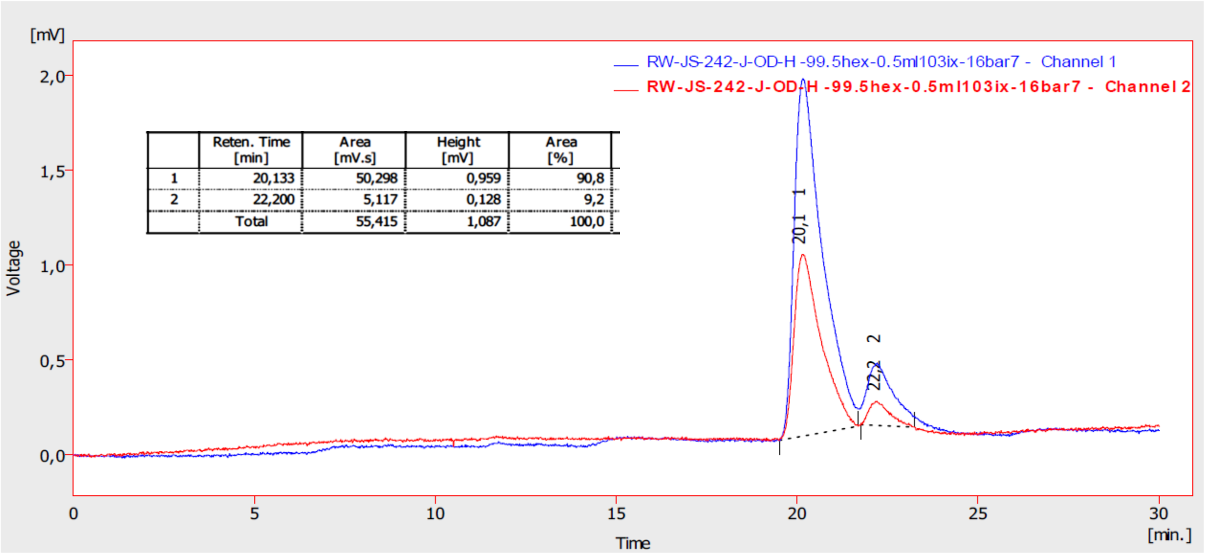

	Reten. Time [min]	Area [mV.s]	Height [mV]	Area [%]
1	20,133	50,298	0,959	90,8
2	22,200	5,117	0,128	9,2
	Total	55,415	1,087	100,0

2t: racemates

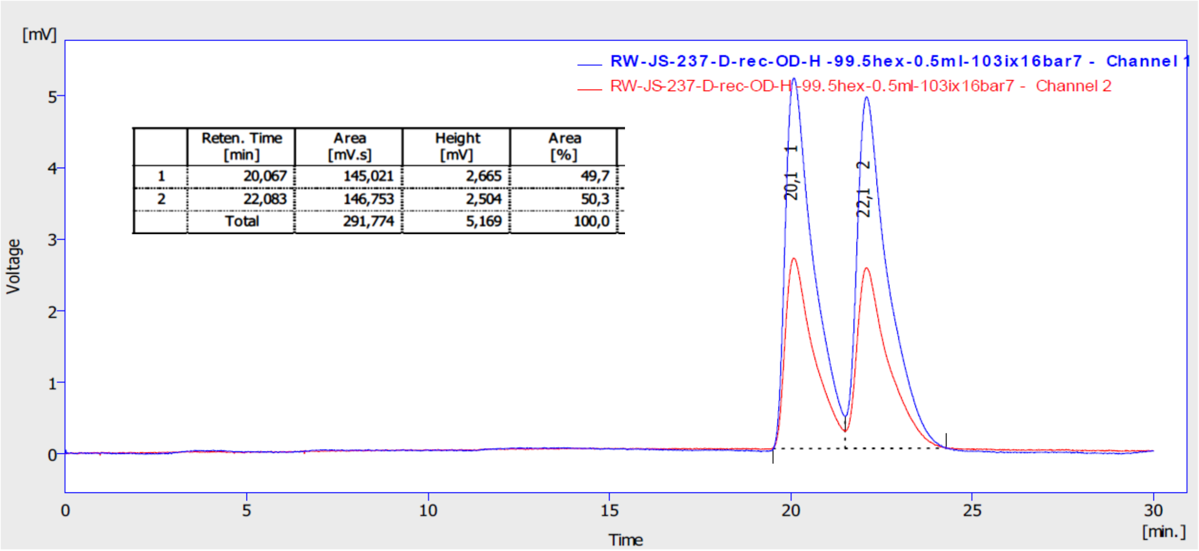

	Reten. Time [min]	Area [mV.s]	Height [mV]	Area [%]
1	20,067	145,021	2,665	49,7
2	22,083	146,753	2,504	50,3
	Total	291,774	5,169	100,0

2u:

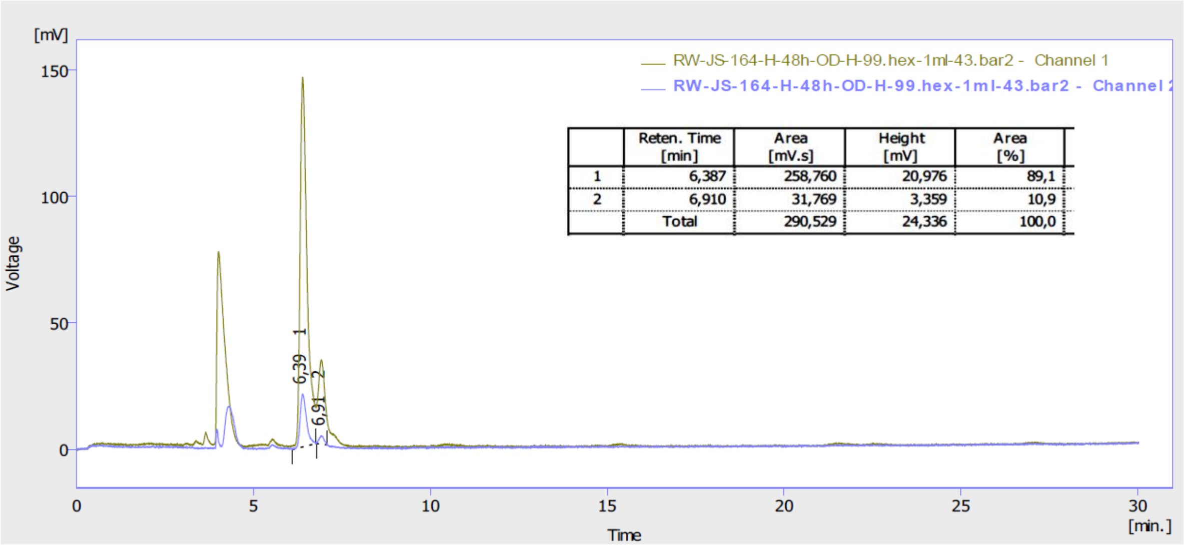

2u: racemates

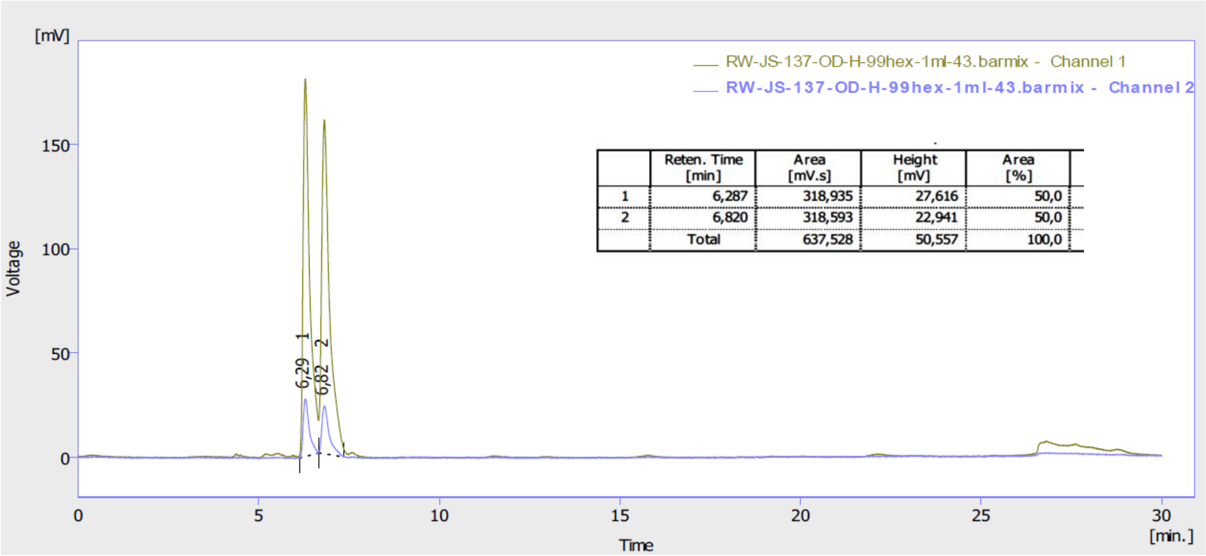

2v:

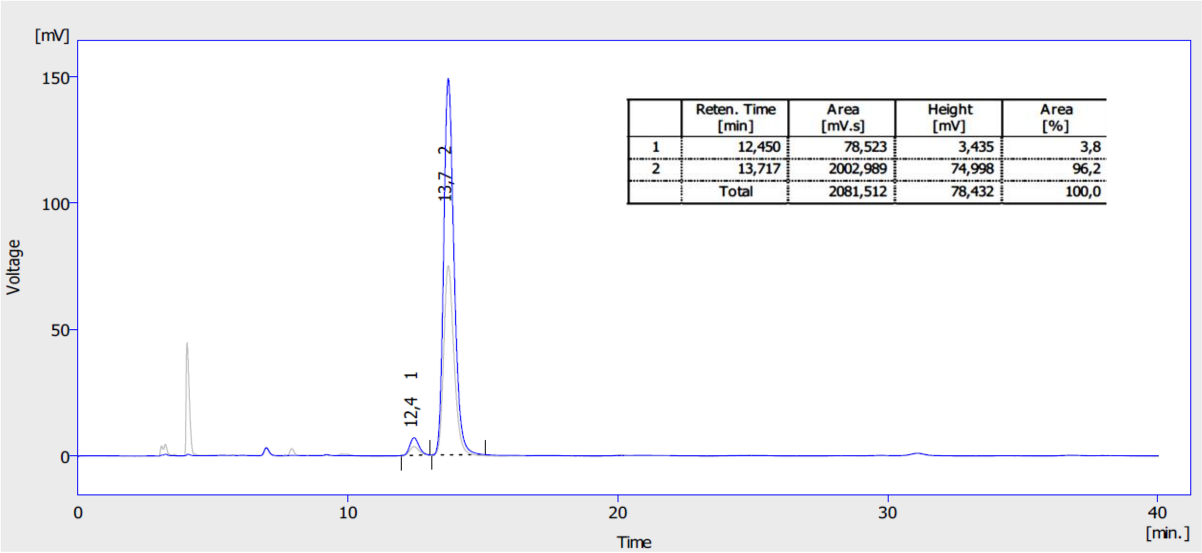

	Reten. Time [min]	Area [mV.s]	Height [mV]	Area [%]
1	12,450	78,523	3,435	3,8
2	13,717	2002,989	74,998	96,2
	Total	2081,512	78,432	100,0

2v: racemates

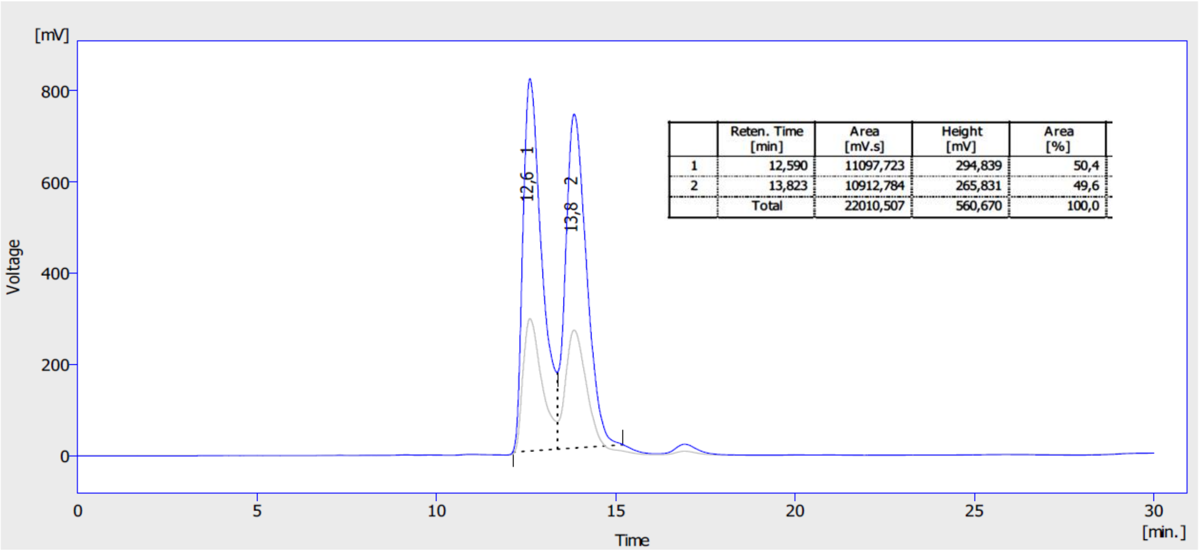

	Reten. Time [min]	Area [mV.s]	Height [mV]	Area [%]
1	12,590	11097,723	294,839	50,4
2	13,823	10912,784	265,831	49,6
	Total	22010,507	560,670	100,0

Copies of NMR for all starting alcohols and products:

1a (^{1}H NMR and ^{13}C NMR, $CDCl_3$)

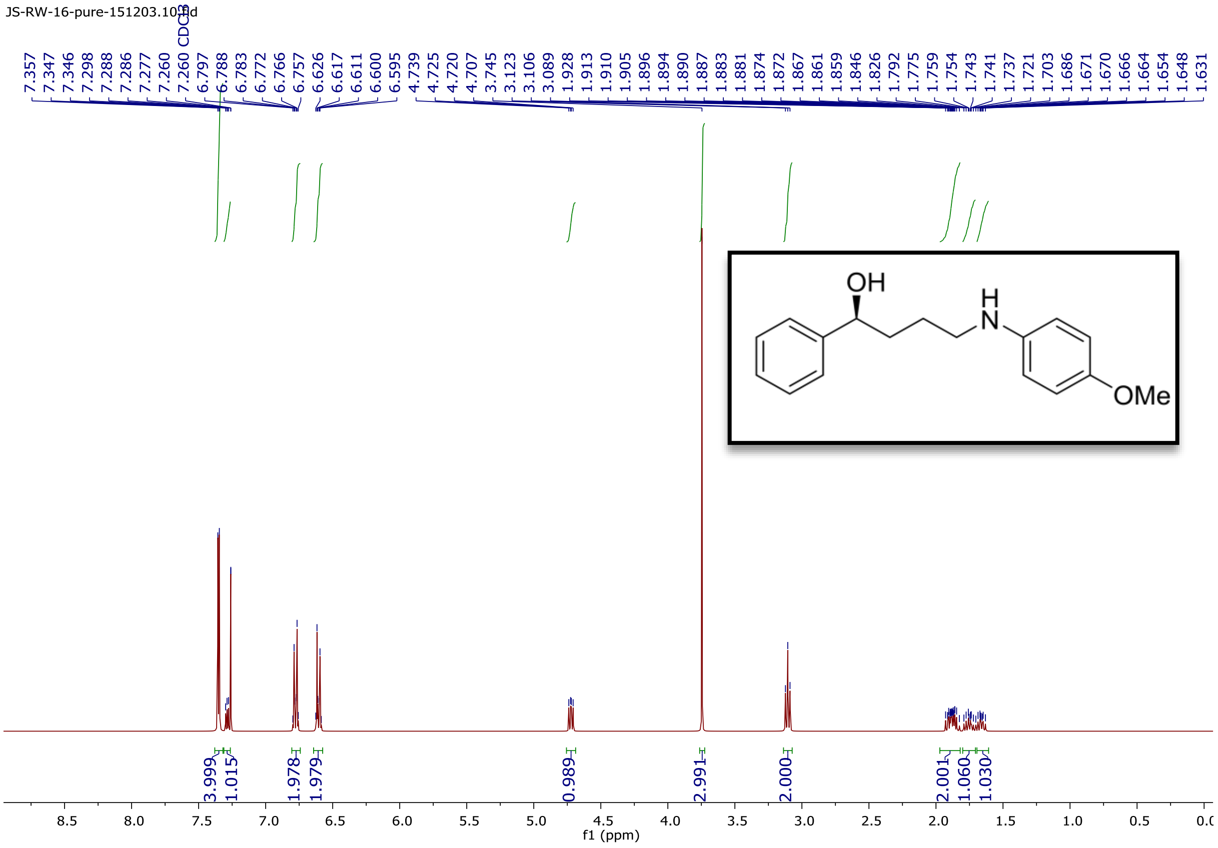

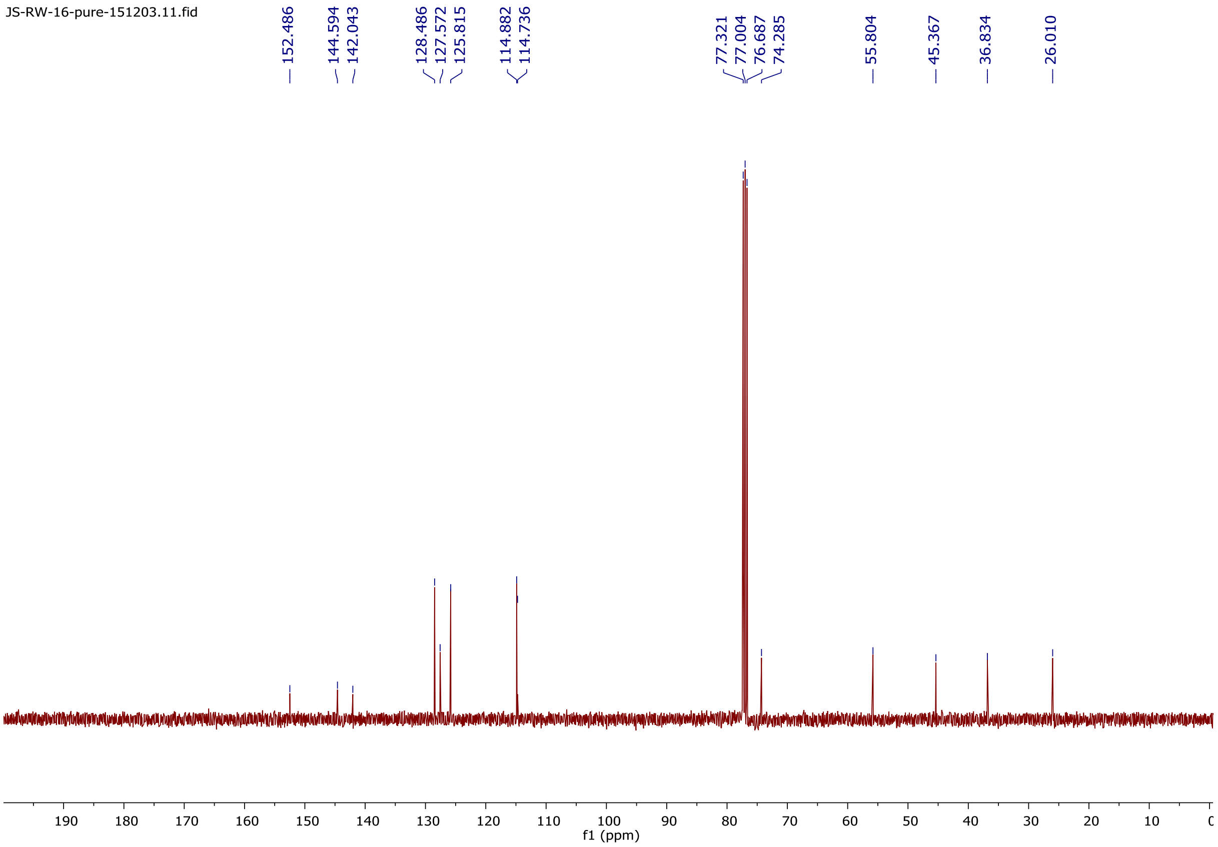

1b (^{1}H NMR and ^{13}C NMR, $CDCl_3$)

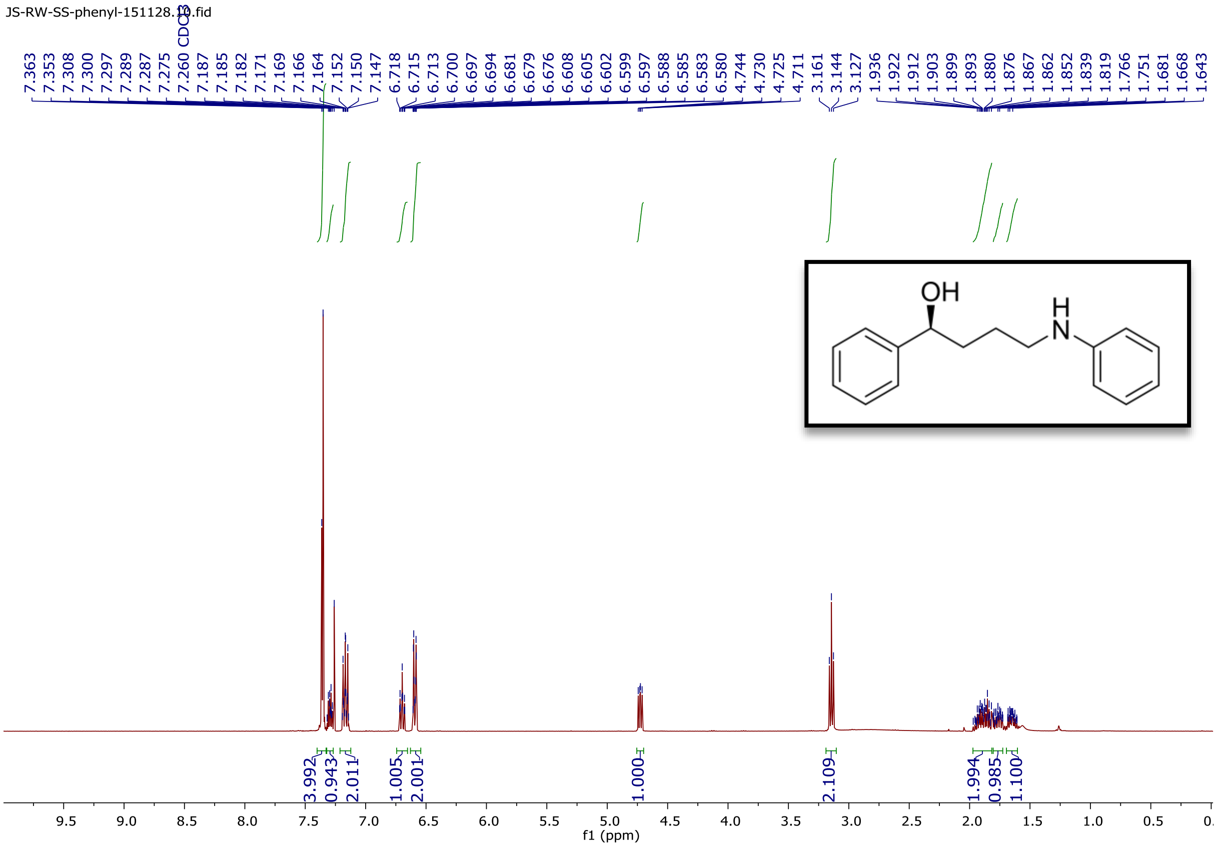

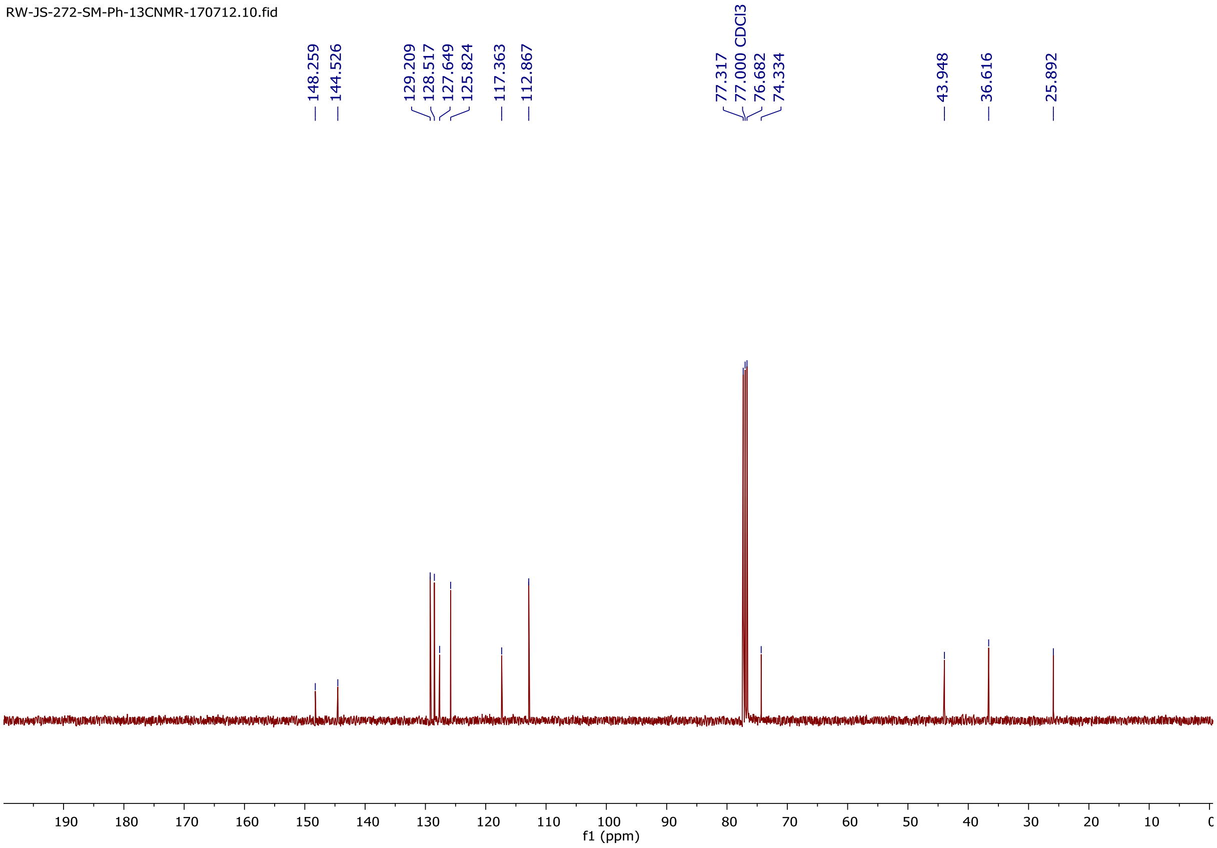

1b' (^{1}H NMR, $CDCl_3$)

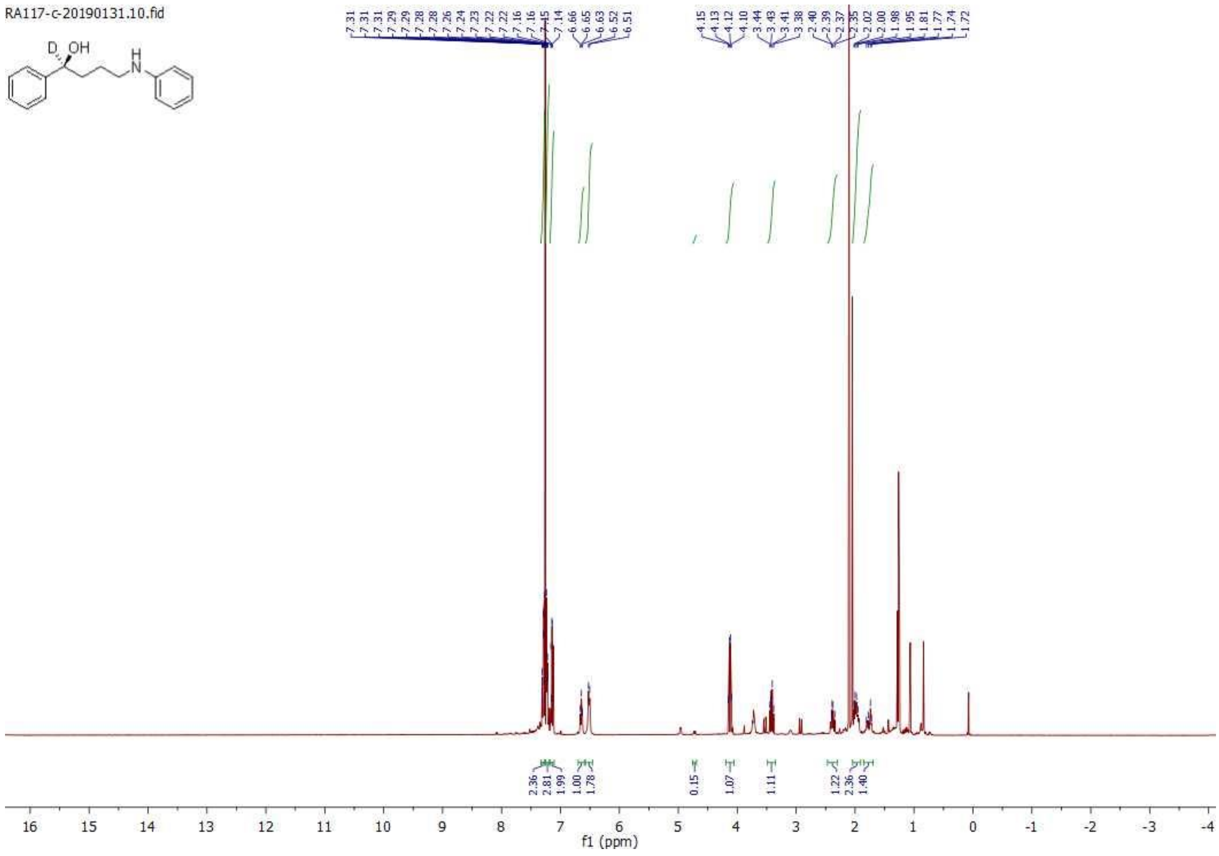

1d (^{1}H NMR and ^{13}C NMR, $CDCl_3$)

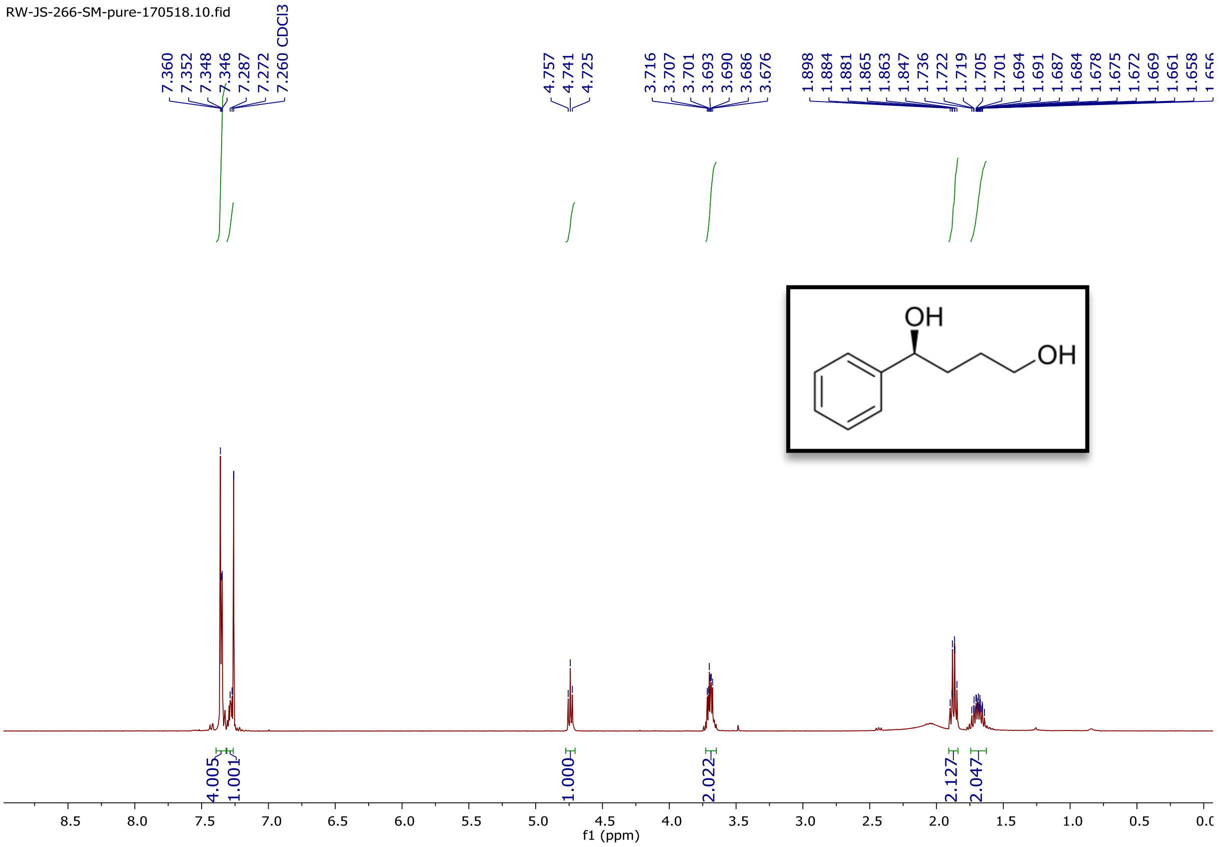

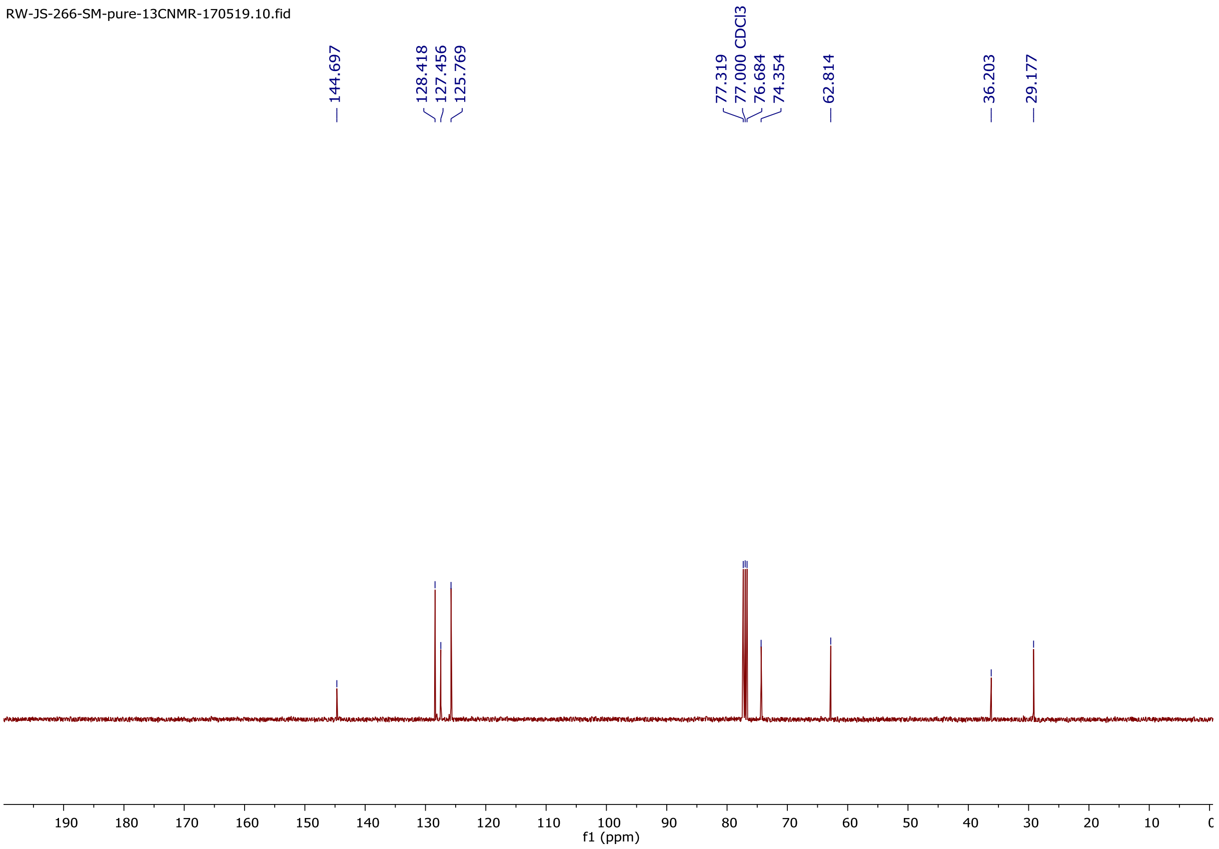

1e (^{1}H NMR and ^{13}C NMR, $CDCl_3$)

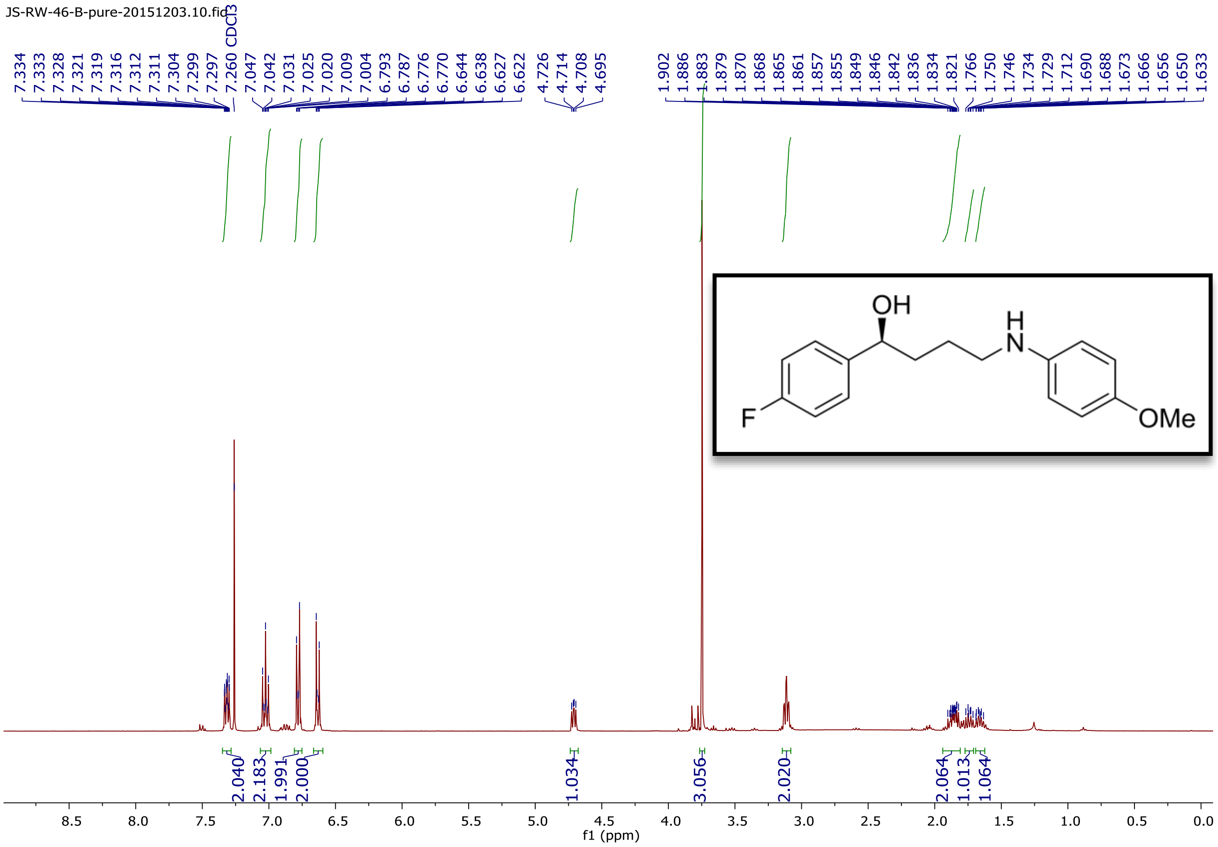

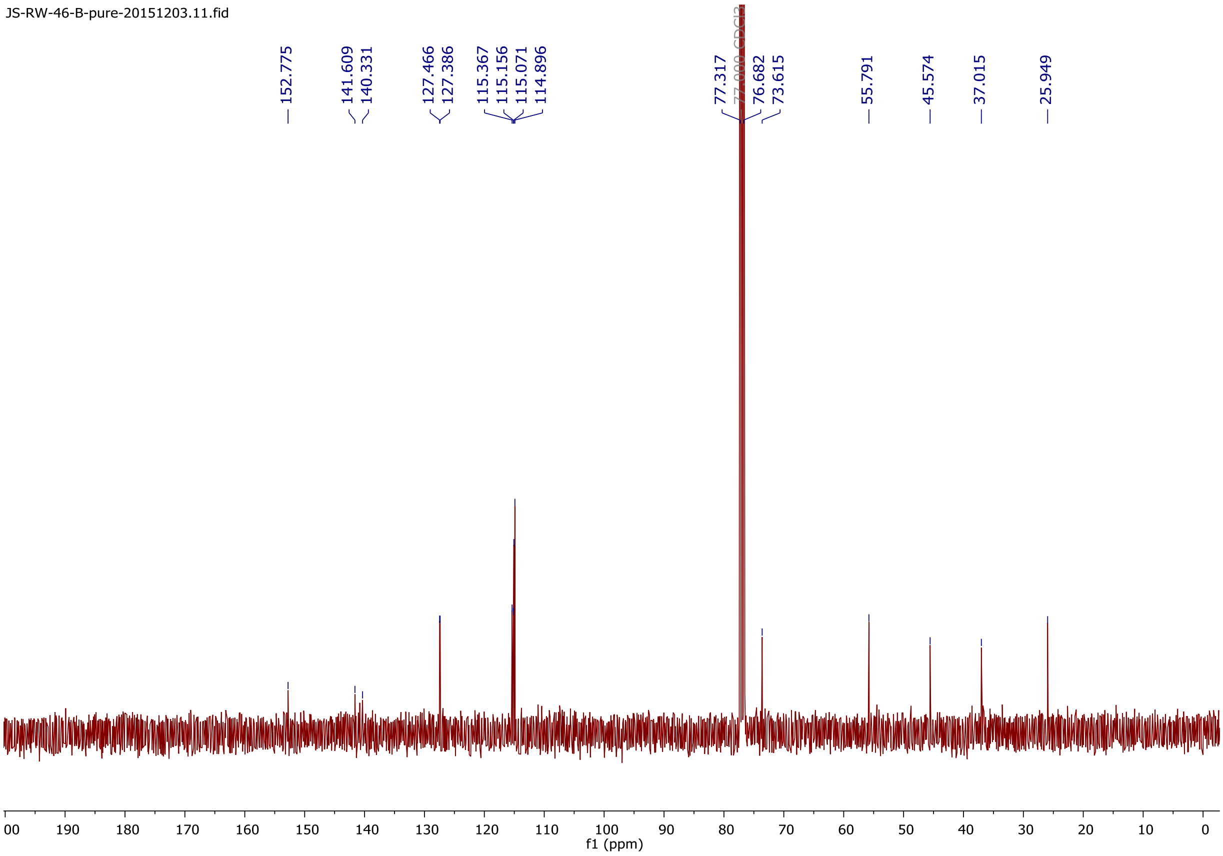

1f (^{1}H NMR and ^{13}C NMR, $CDCl_3$)

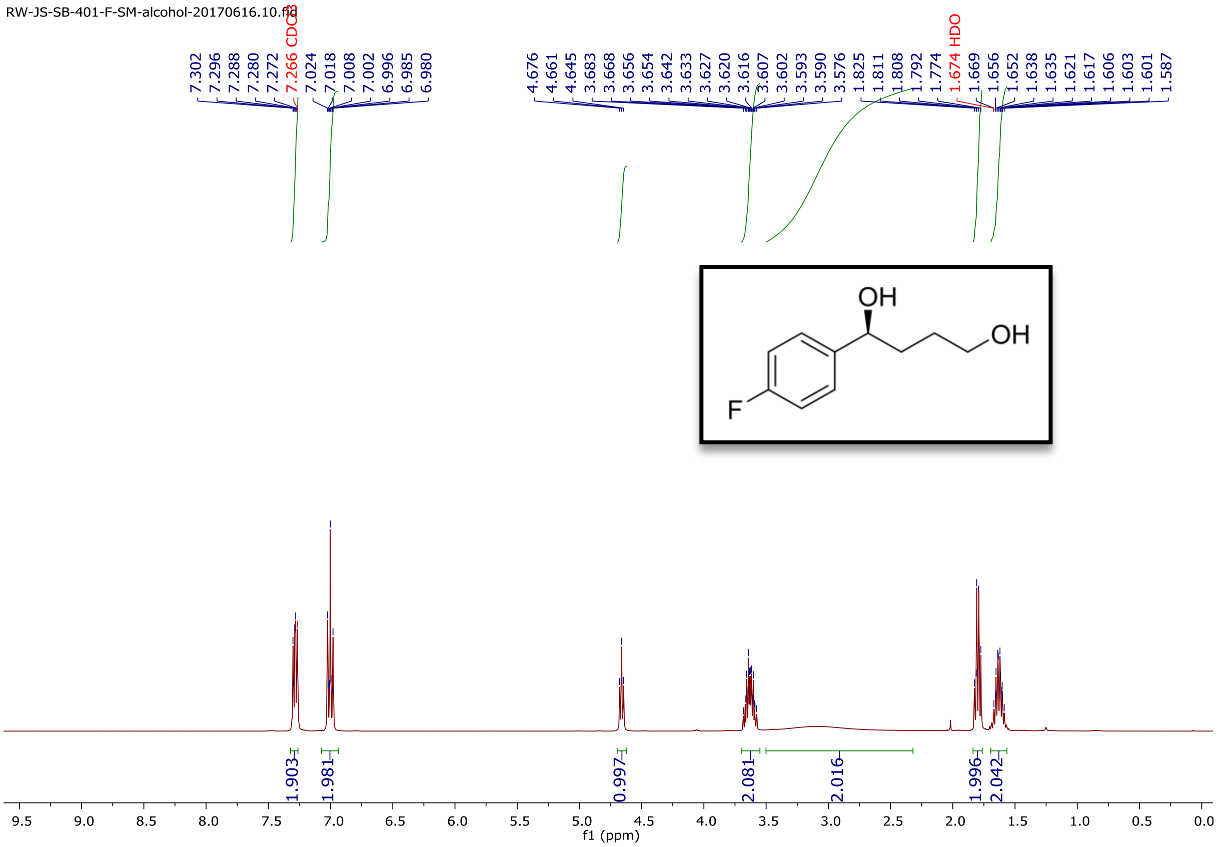

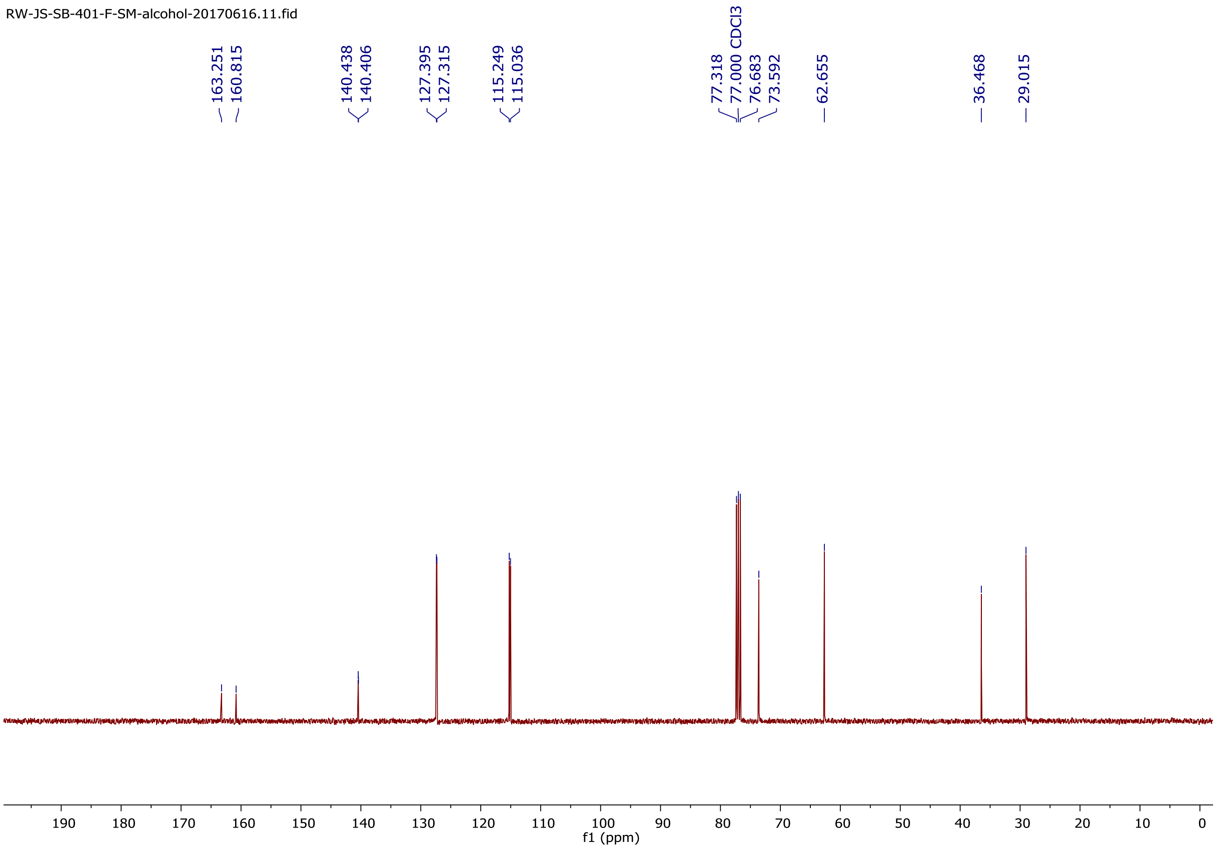

1g (^{1}H NMR and ^{13}C NMR, $CDCl_3$)

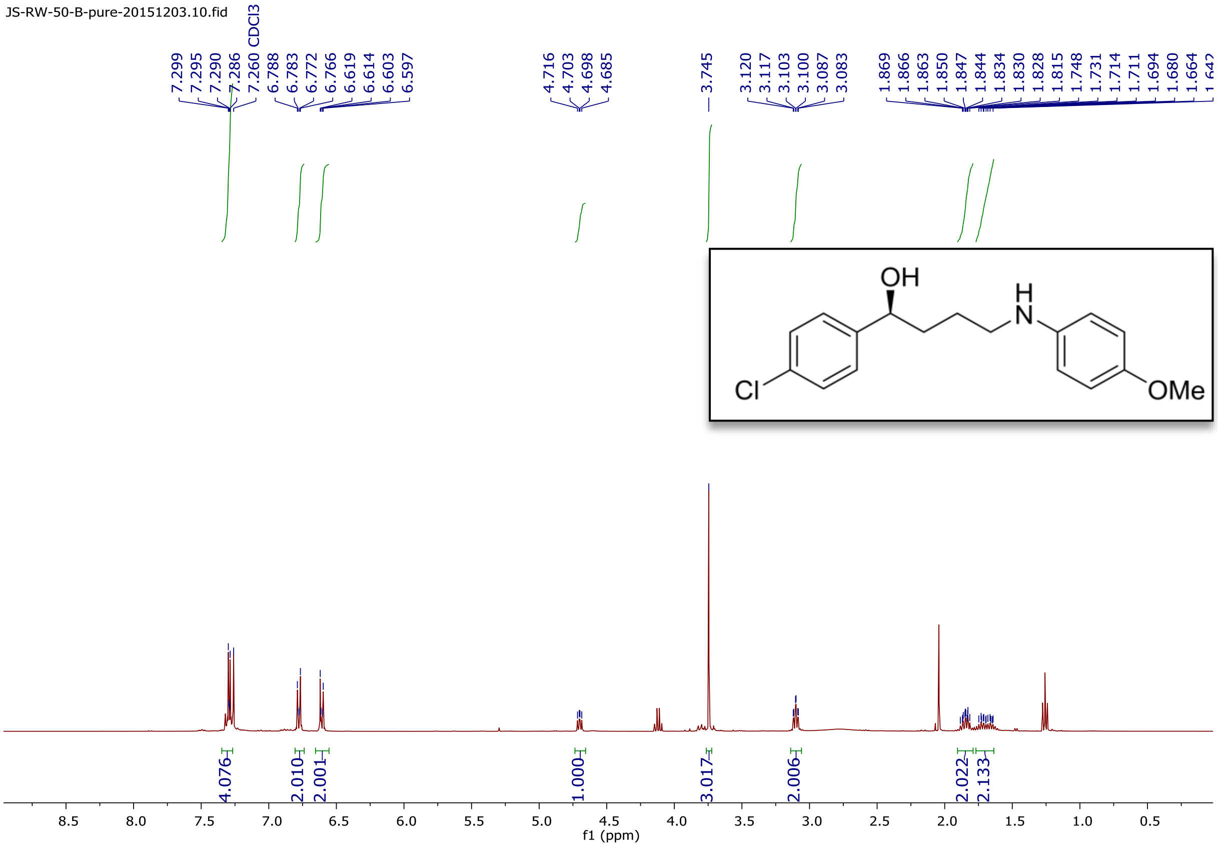

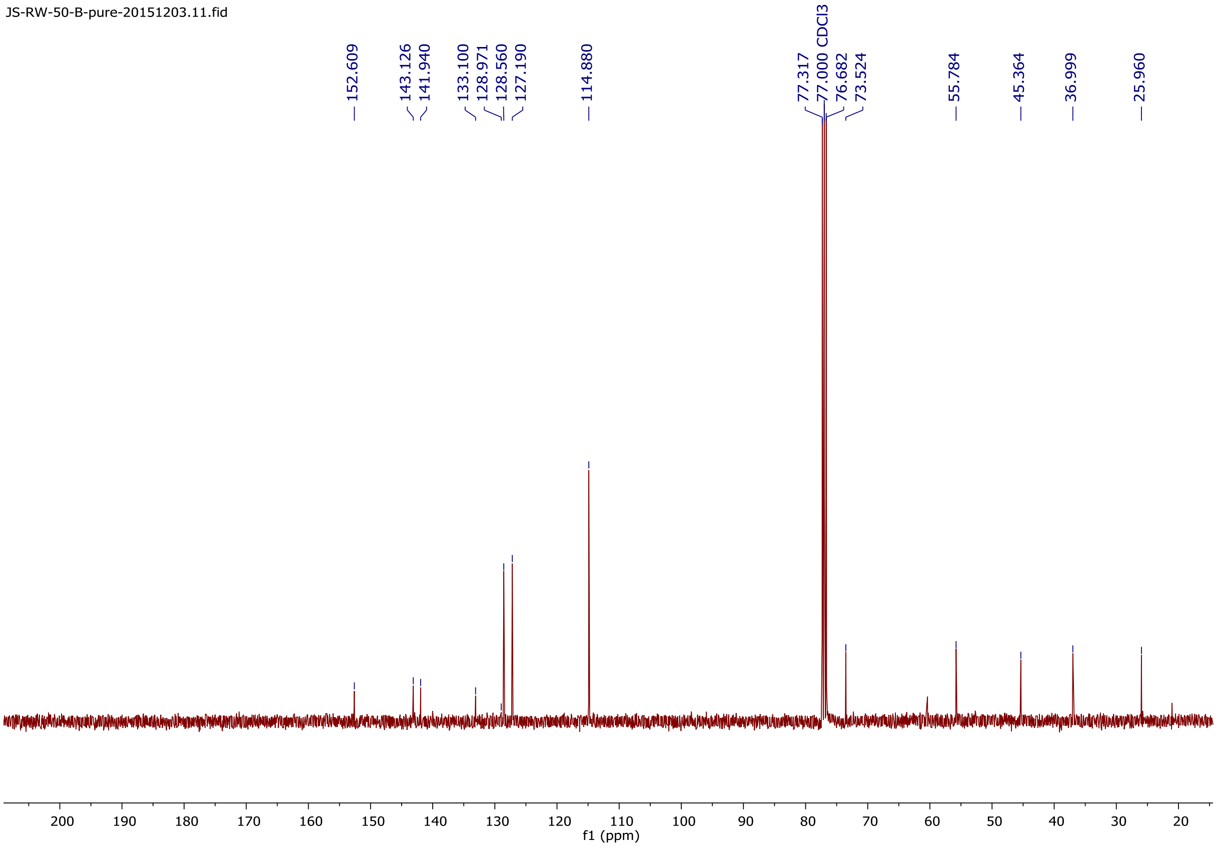

1h (^{1}H NMR and ^{13}C NMR, $CDCl_3$)

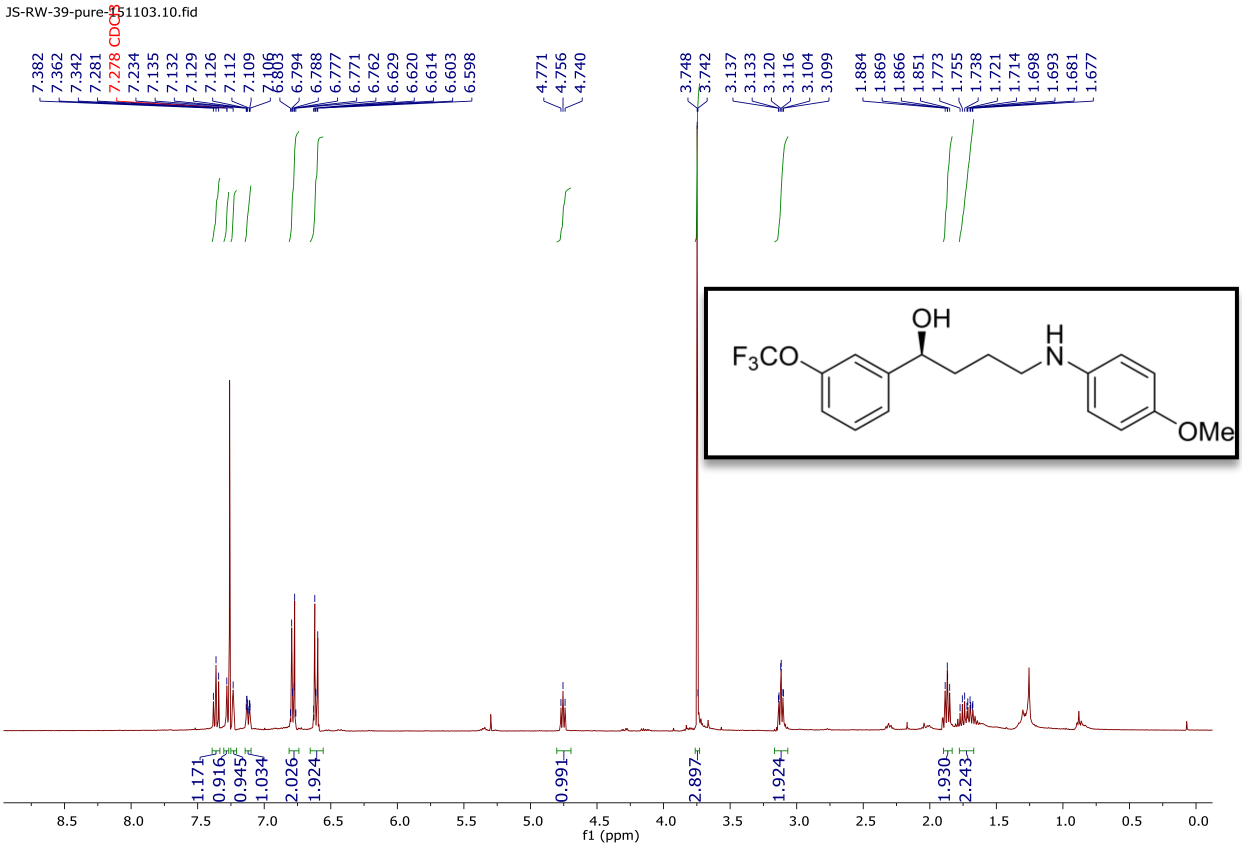

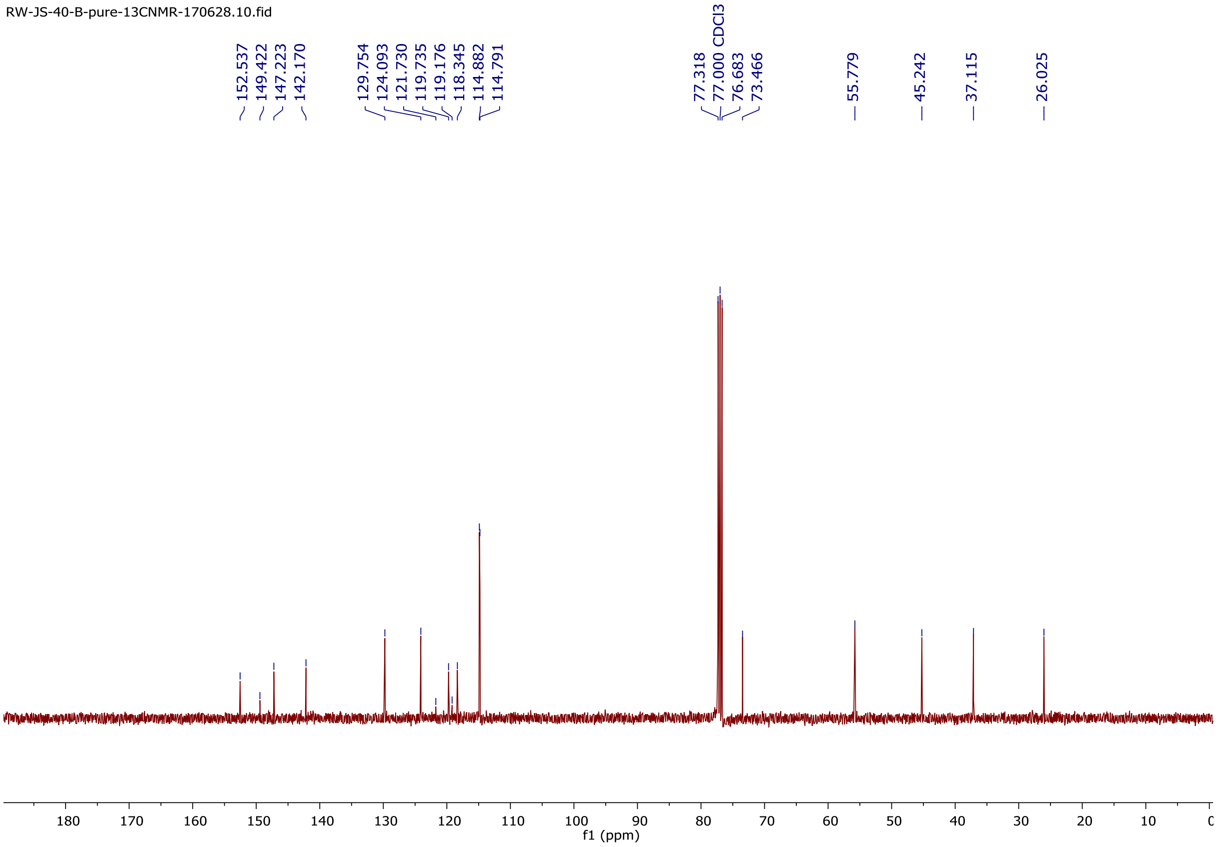

1i (^{1}H NMR and ^{13}C NMR, $CDCl_3$)

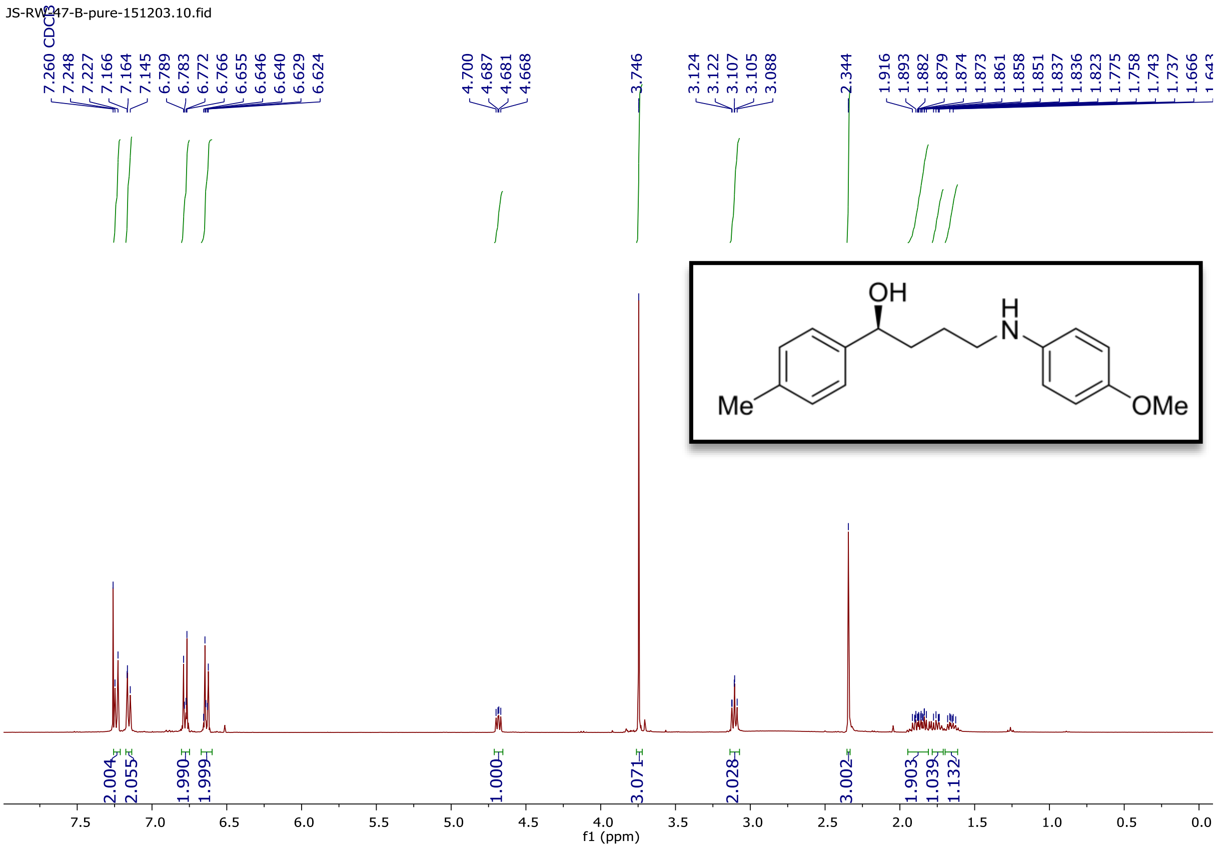

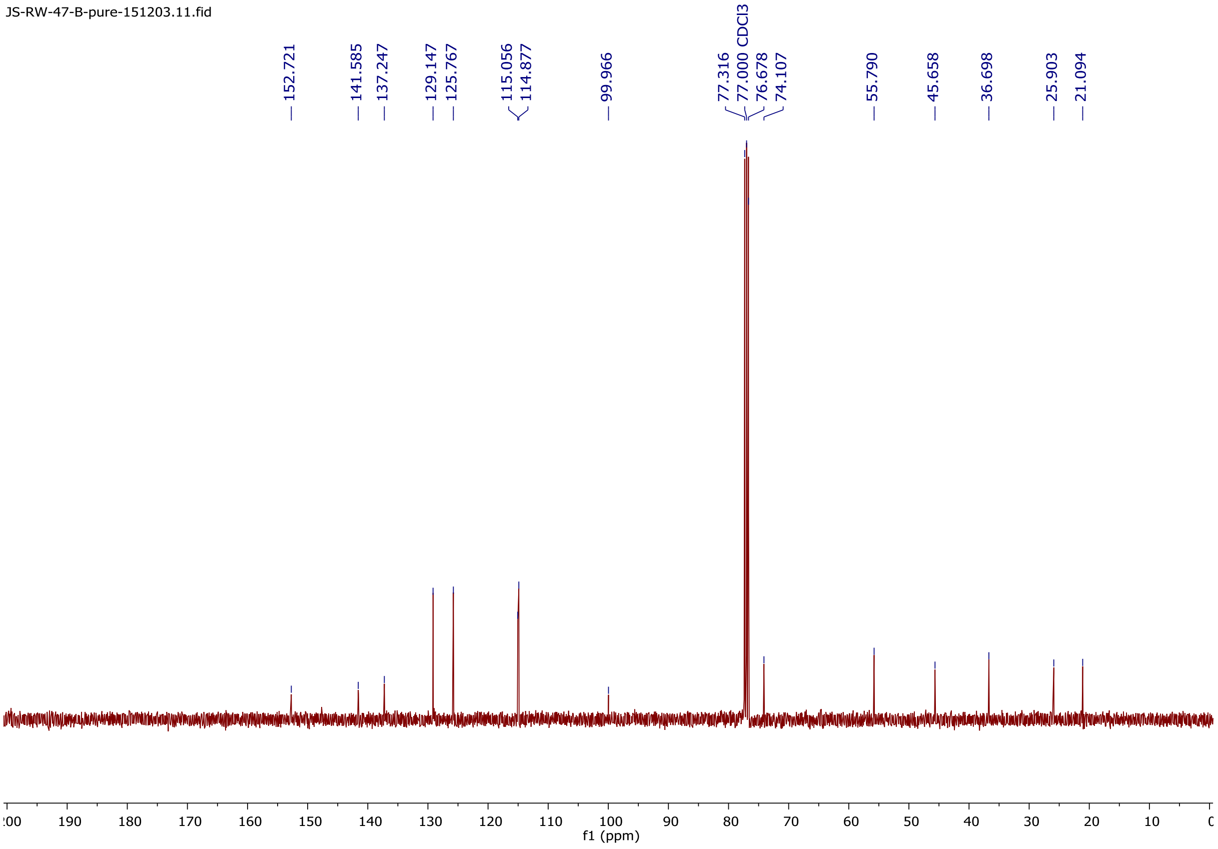

1j (^{1}H NMR and ^{13}C NMR, $CDCl_3$)

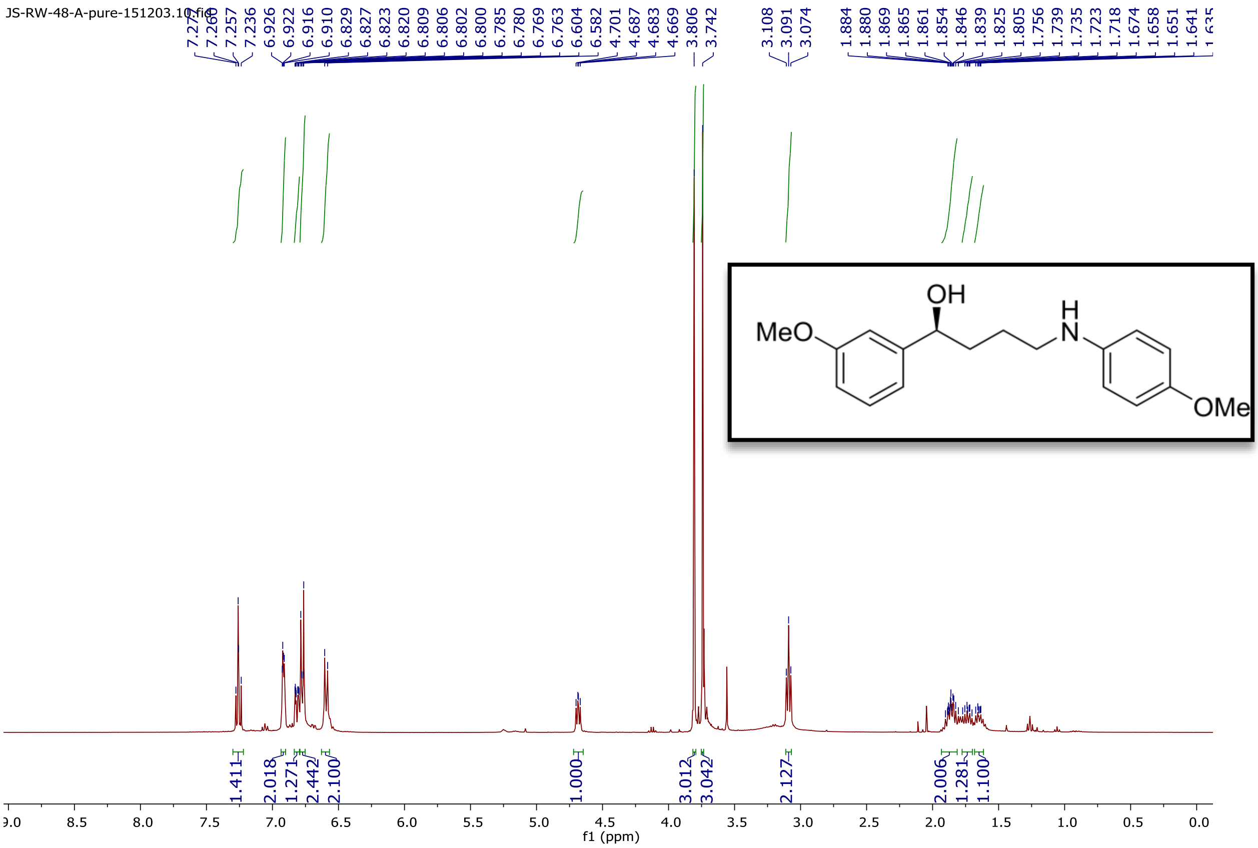

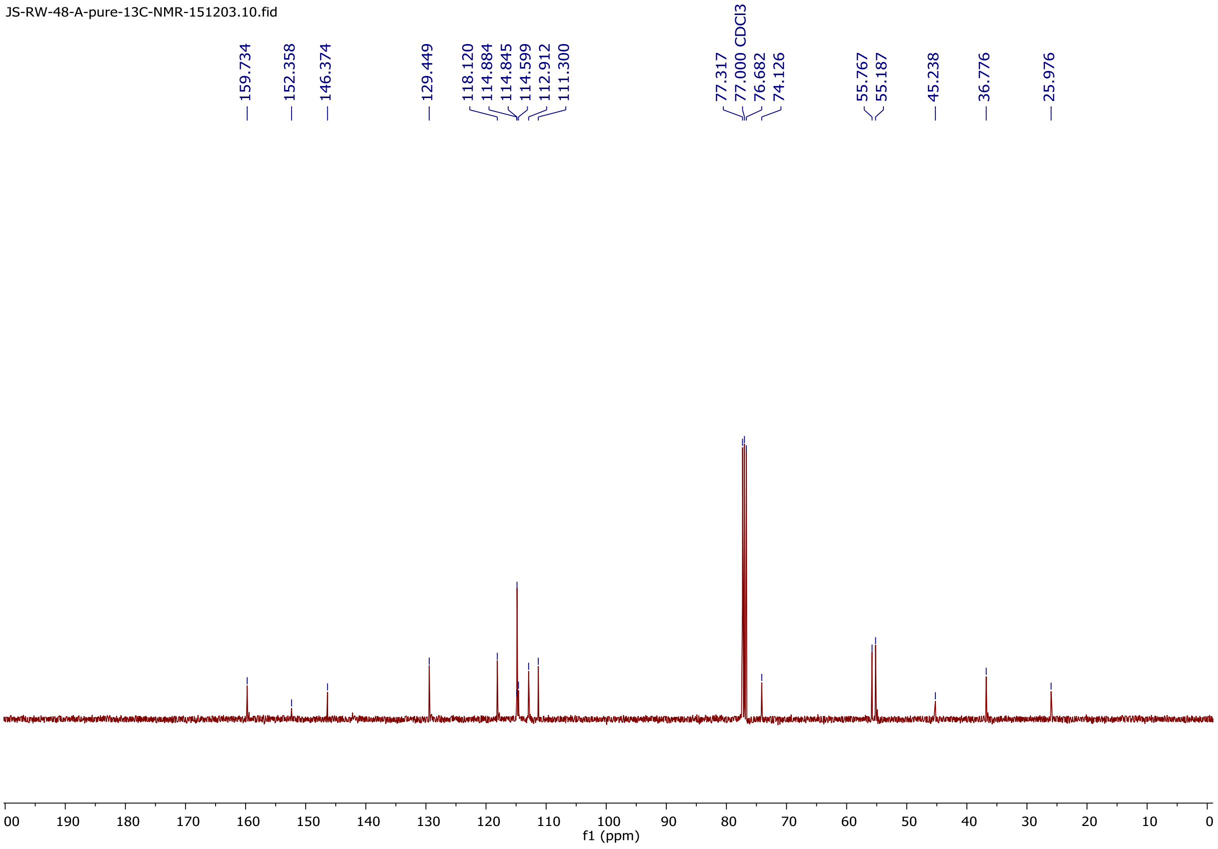

1k (^{1}H NMR and ^{13}C NMR, $CDCl_3$)

AB5-141-AC-170622.10.fid

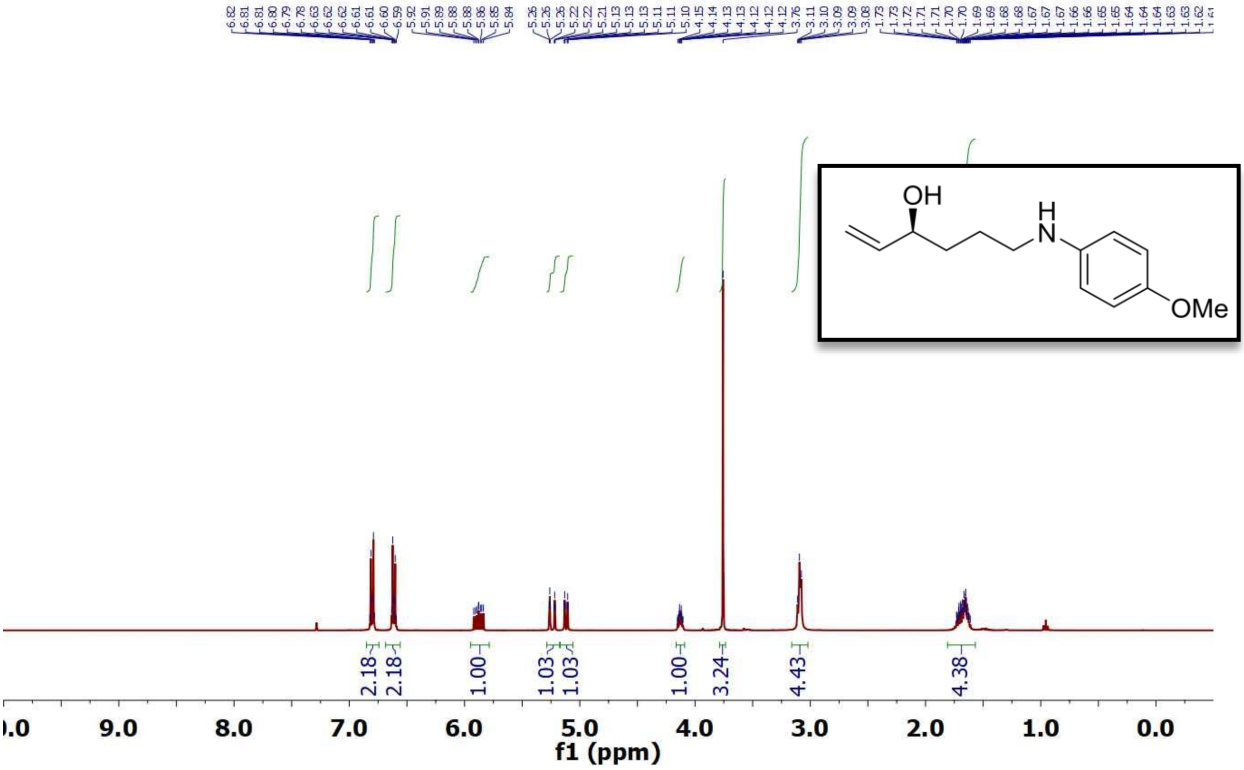

AB5-141-AC-170622.11.fid

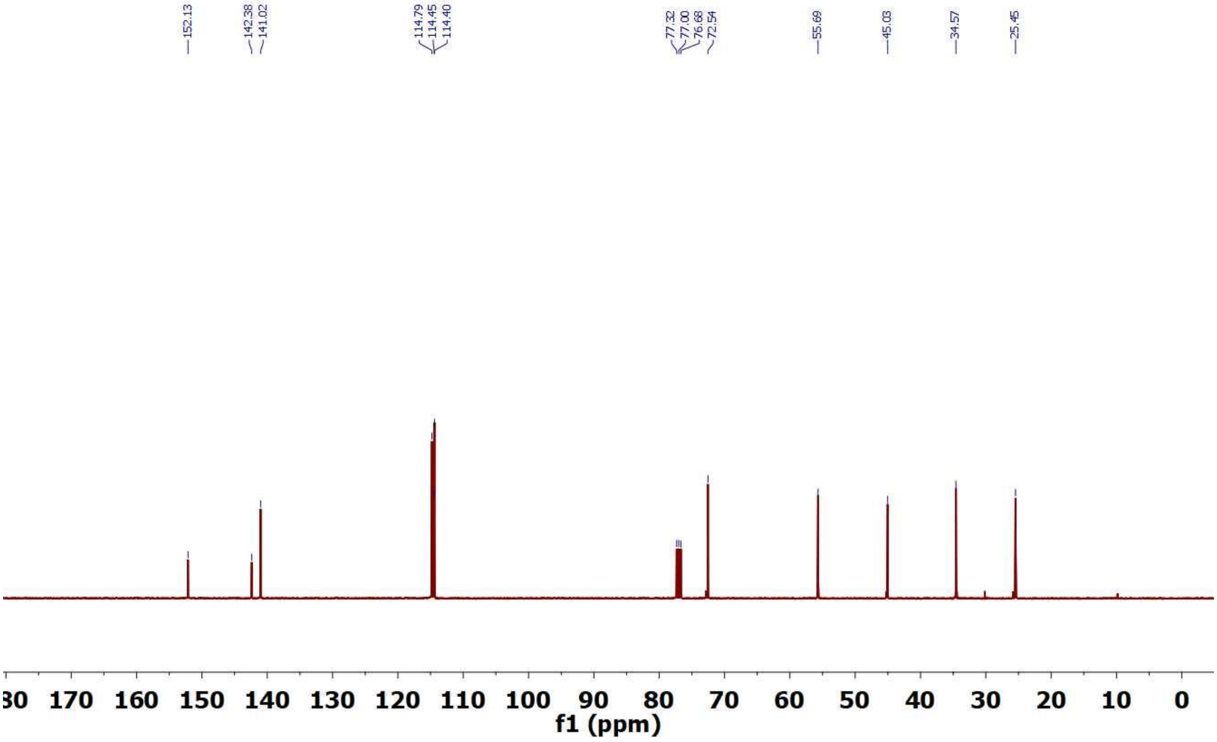

1l (^{1}H NMR and ^{13}C NMR, $CDCl_3$)

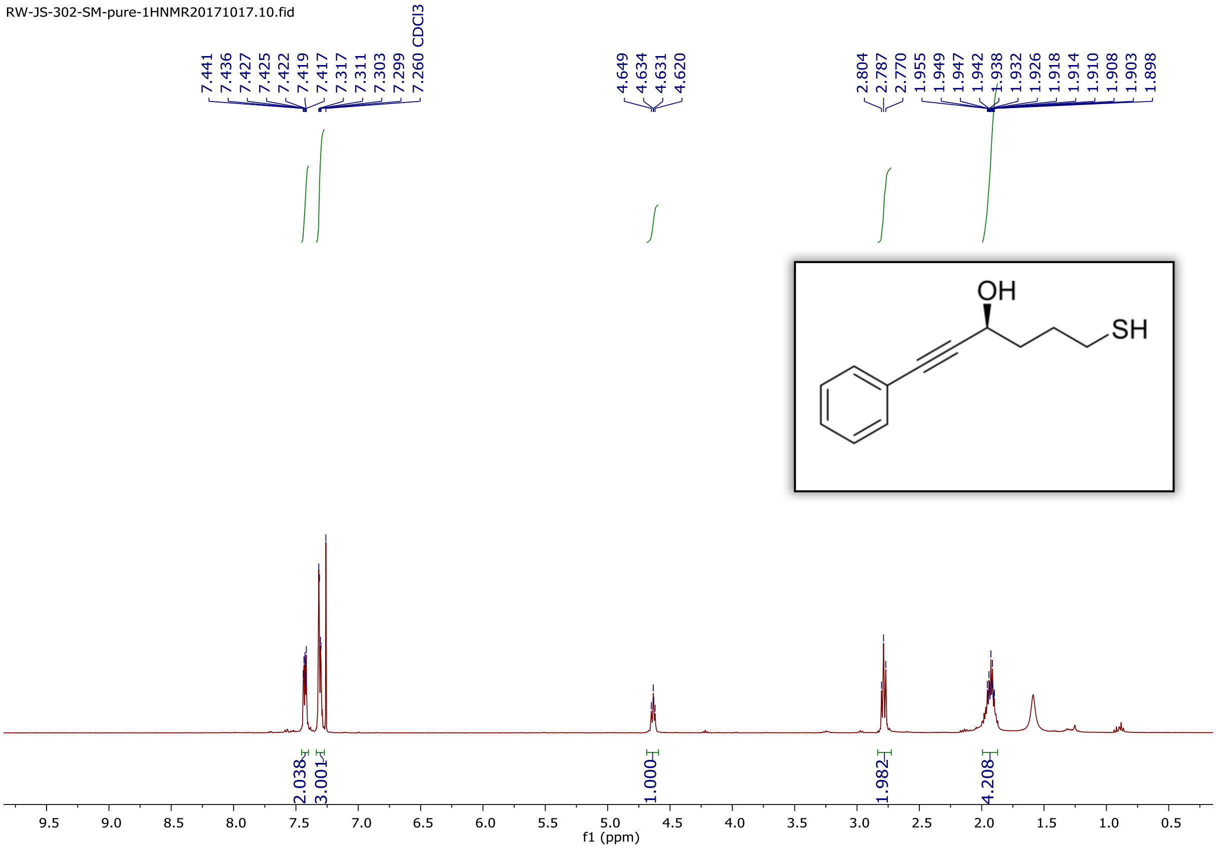

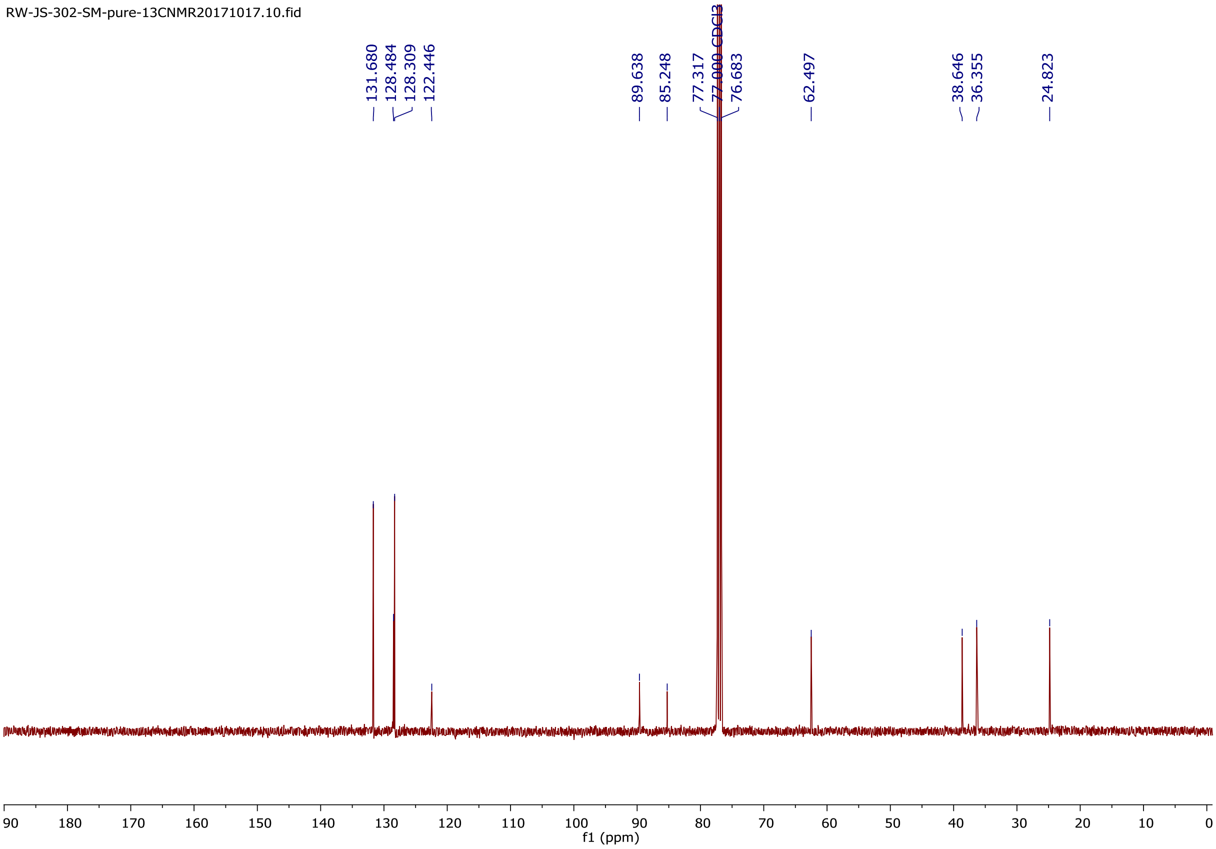

1m (^{1}H NMR and ^{13}C NMR, $CDCl_3$)

AB4-60-AP1-160227-2.10.fid

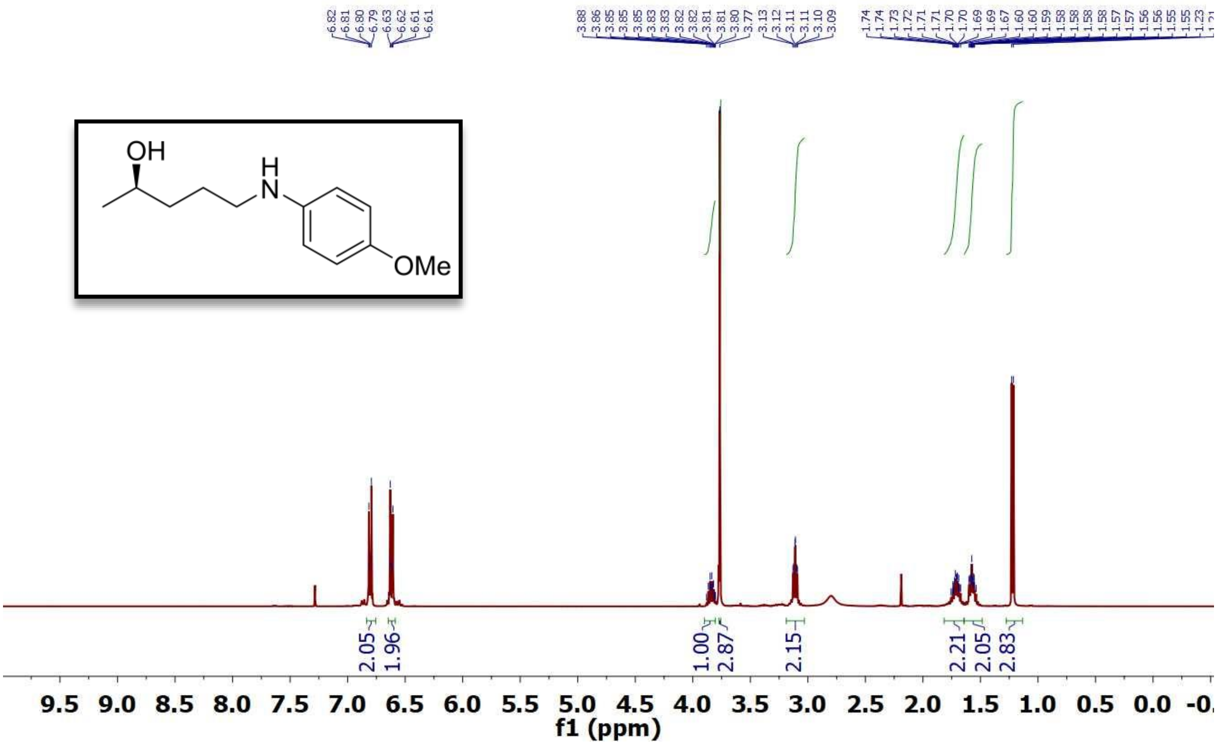

AB4-60-AP1-160227-2.11.fid

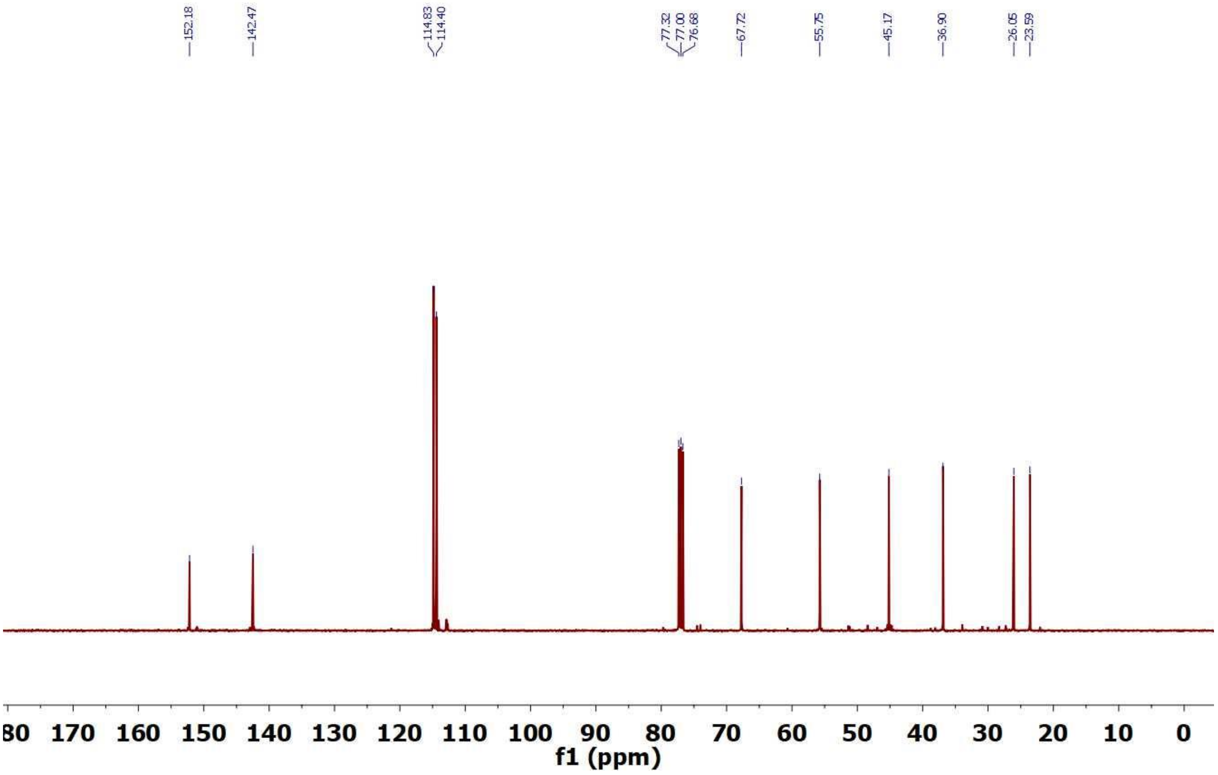

1n (^{1}H NMR and ^{13}C NMR, $CDCl_3$)

AB5-142-AC-160815.10.fid

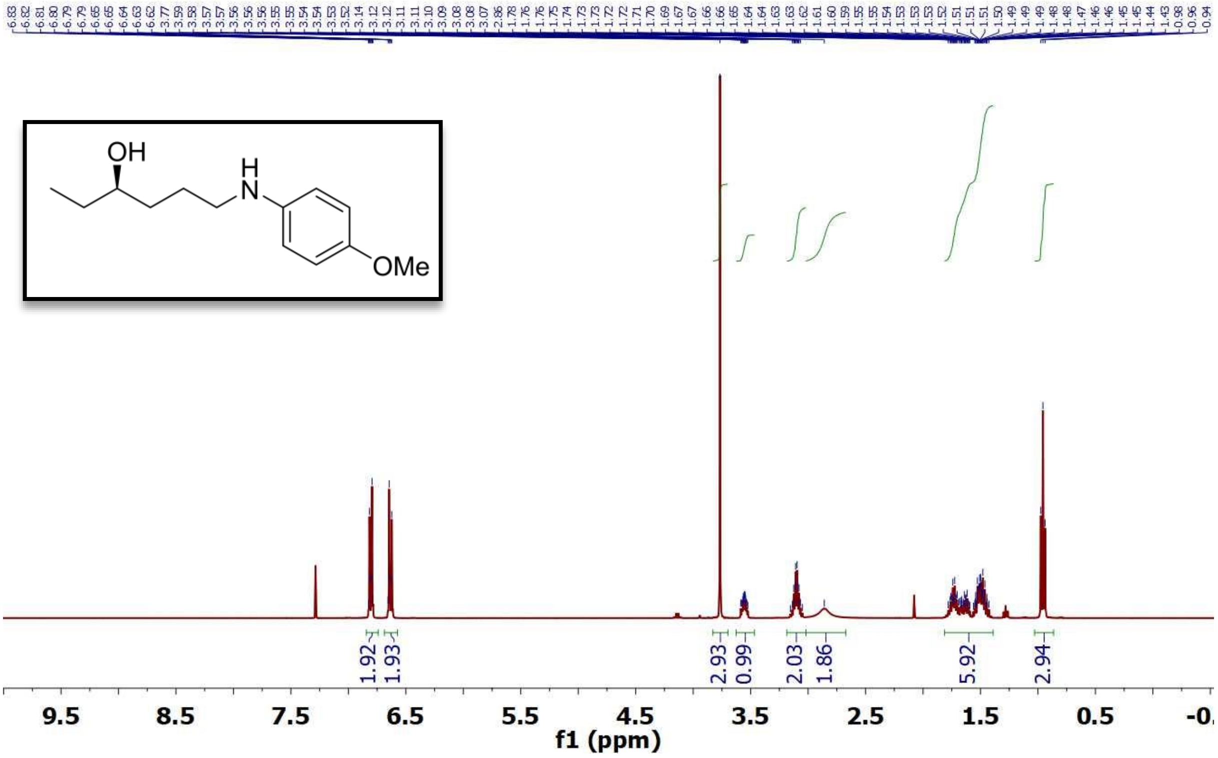

AB5-142-AC-160815.11.fid

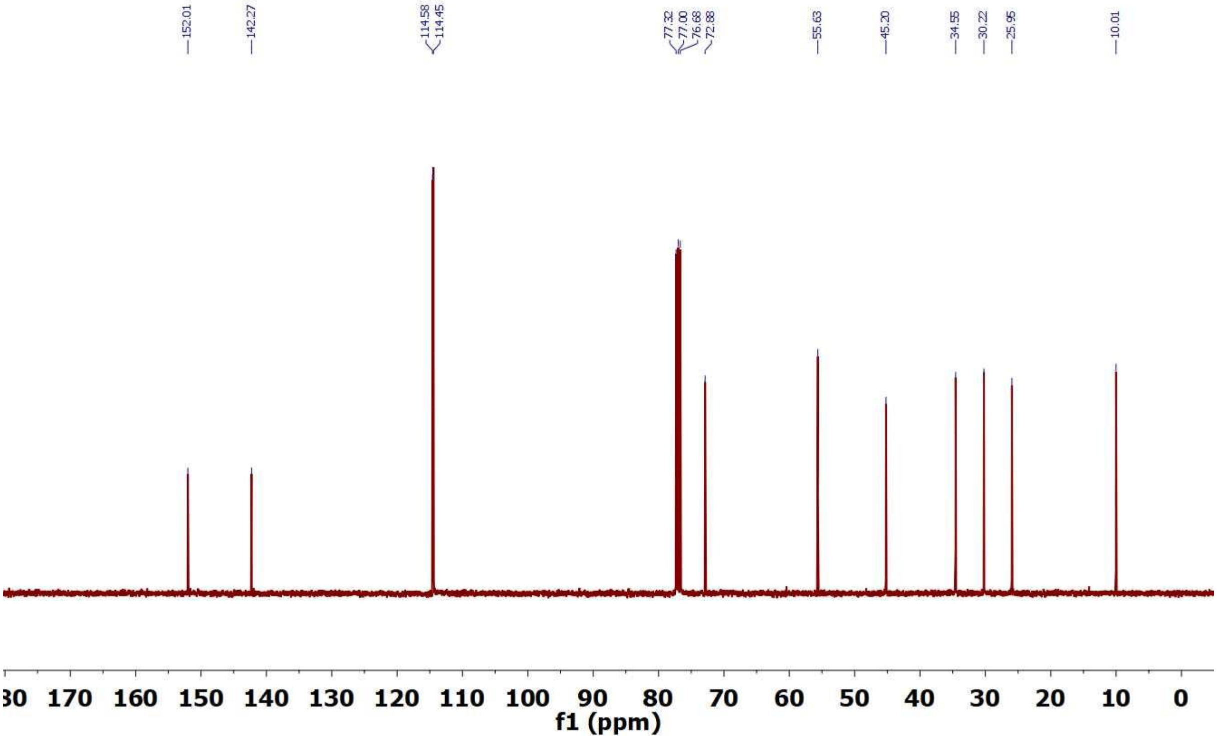

1o (^{1}H NMR and ^{13}C NMR, $CDCl_3$)

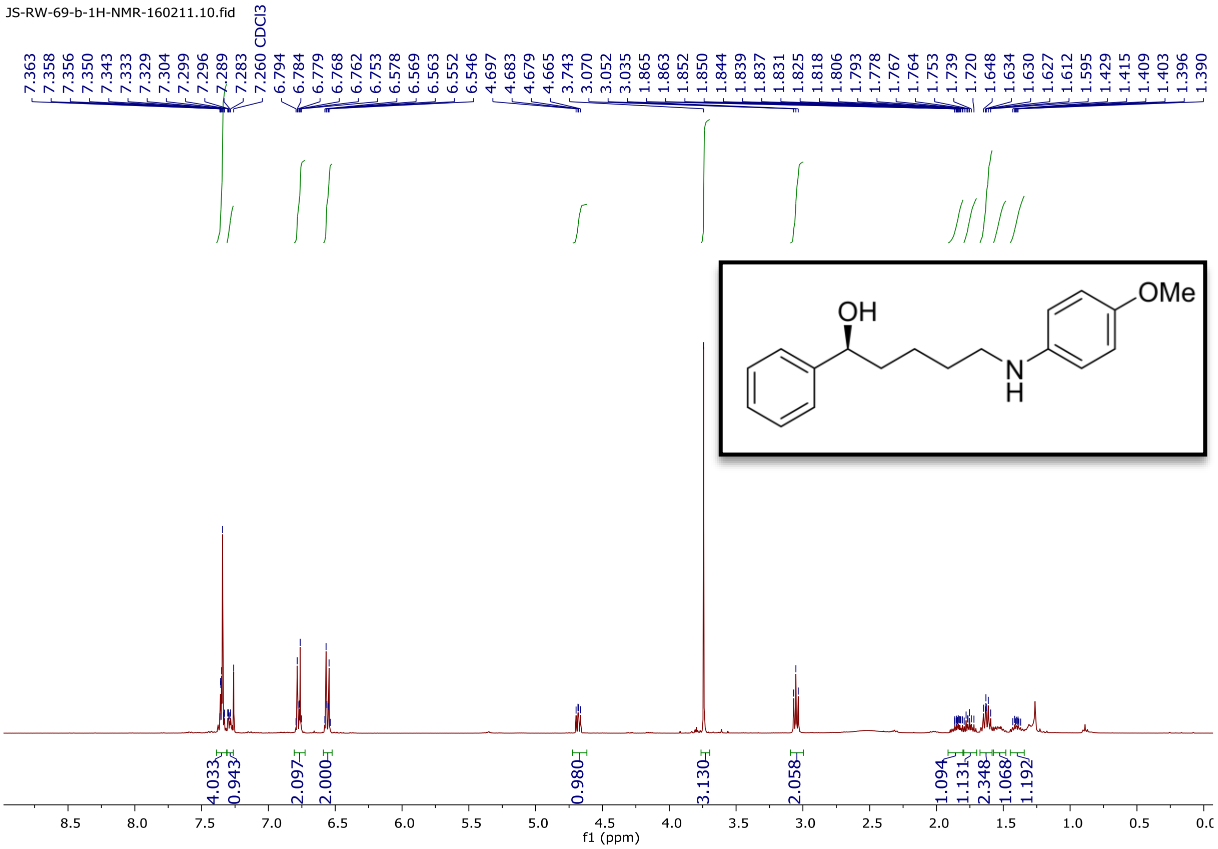

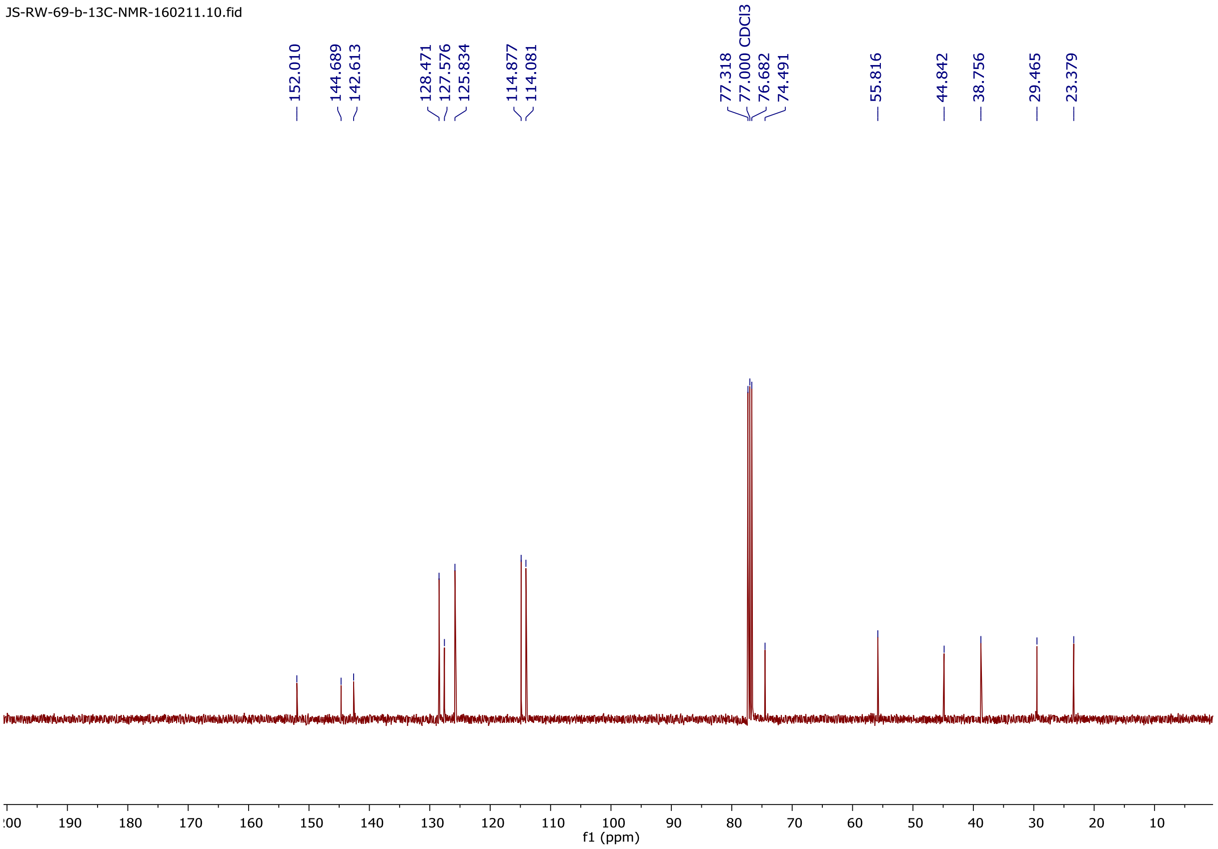

1p (^{1}H NMR and ^{13}C NMR, $CDCl_3$)

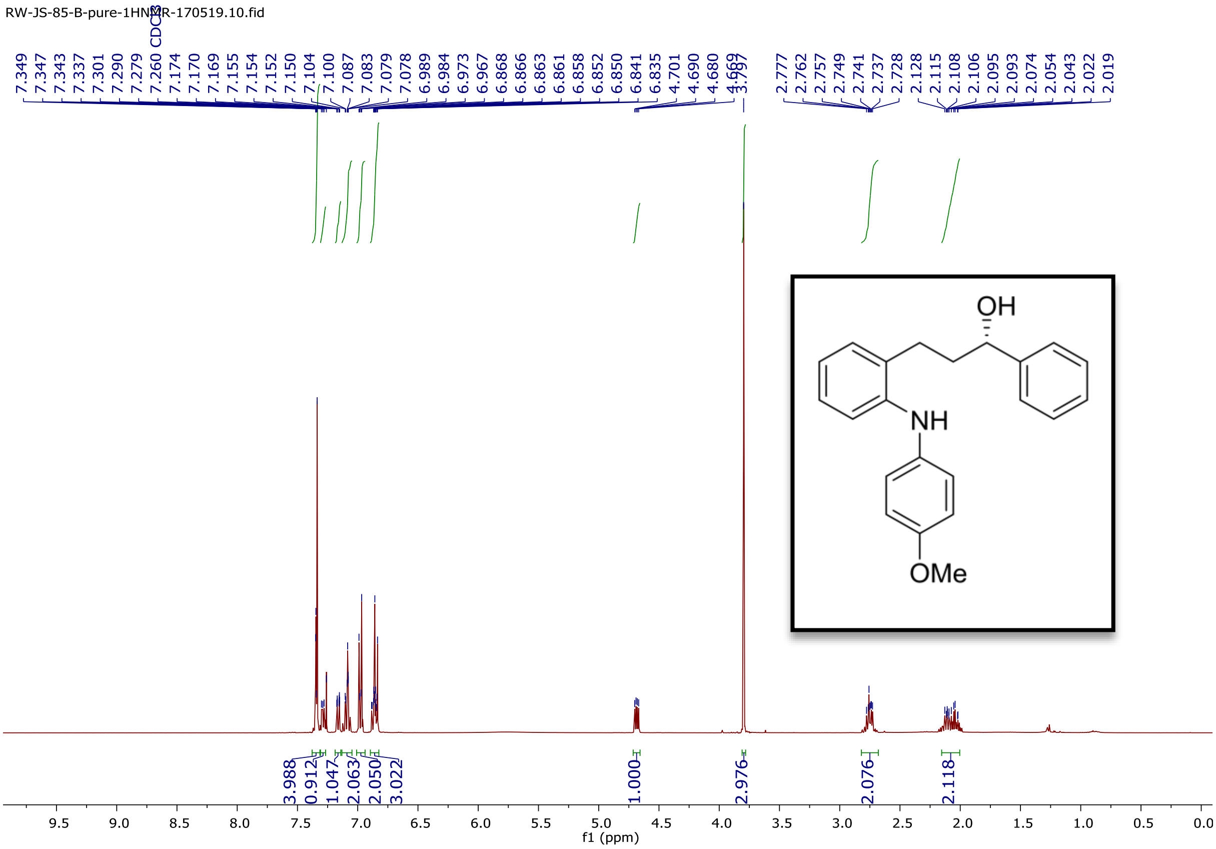

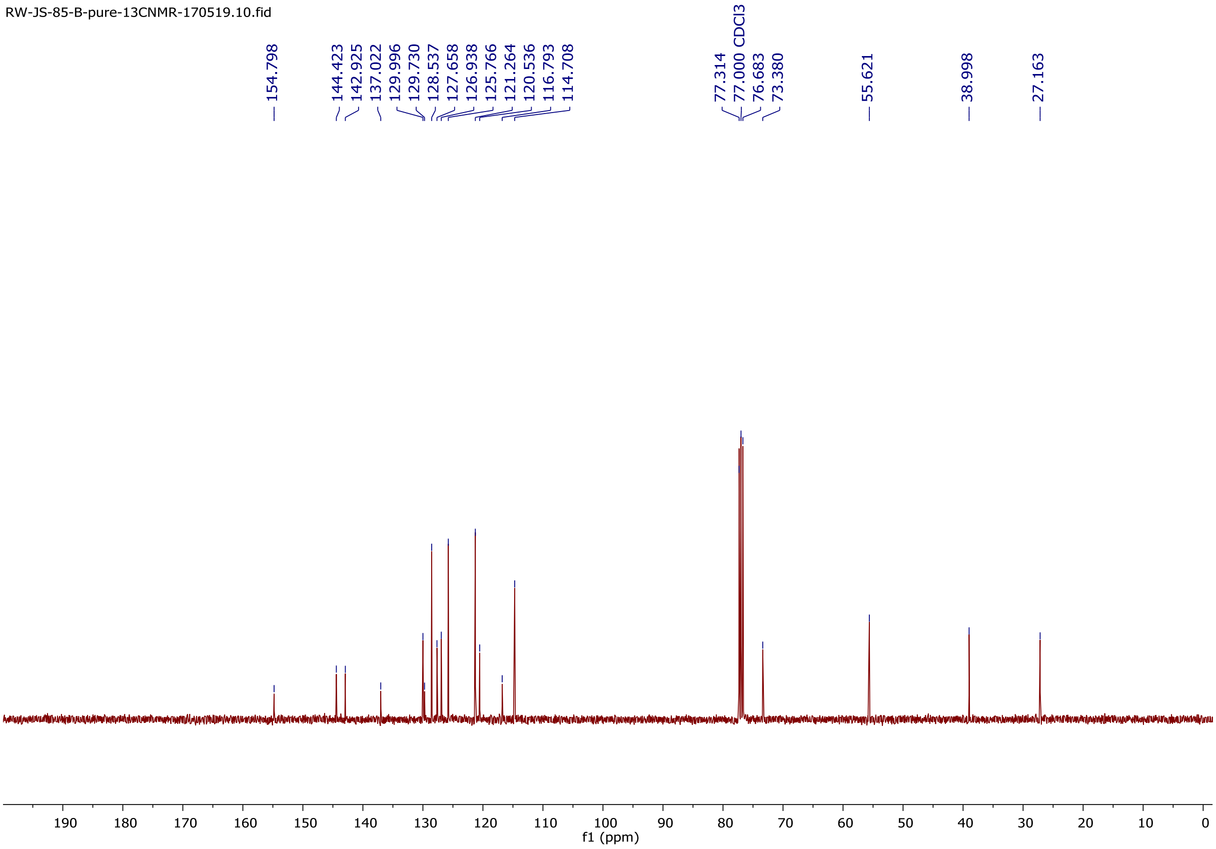

1q (^{1}H NMR and ^{13}C NMR, $CDCl_3$)

AB6-176A-AC1-170622.10.fid

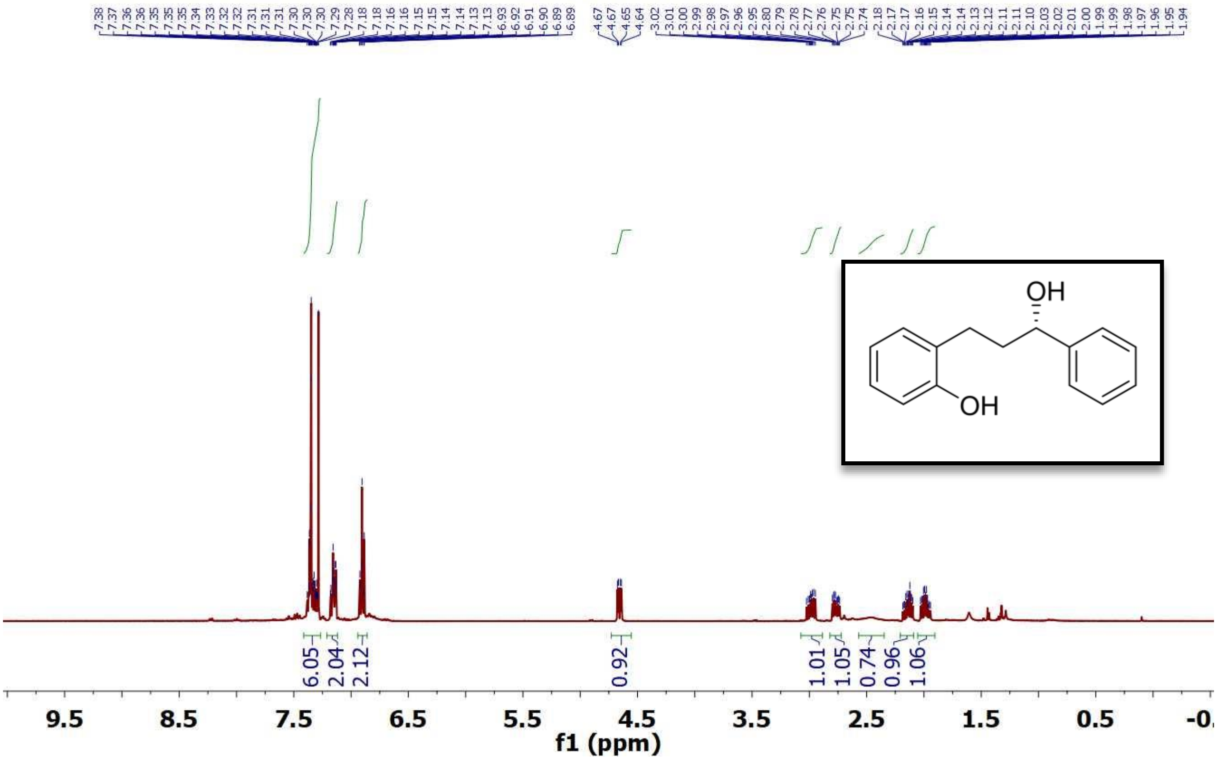

AB6-176A-AC1-170622.12.fid

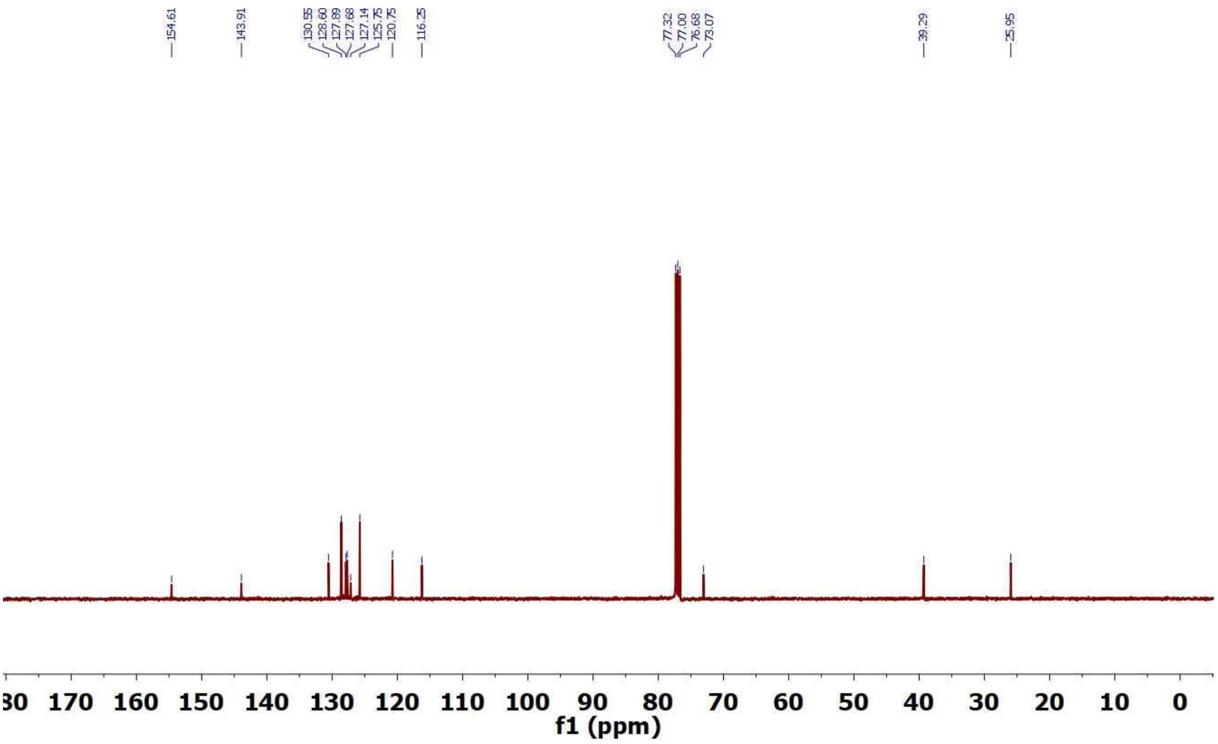

1r (^{1}H NMR and ^{13}C NMR, $CDCl_3$)

AB7-19-AC1-170622.10.fid

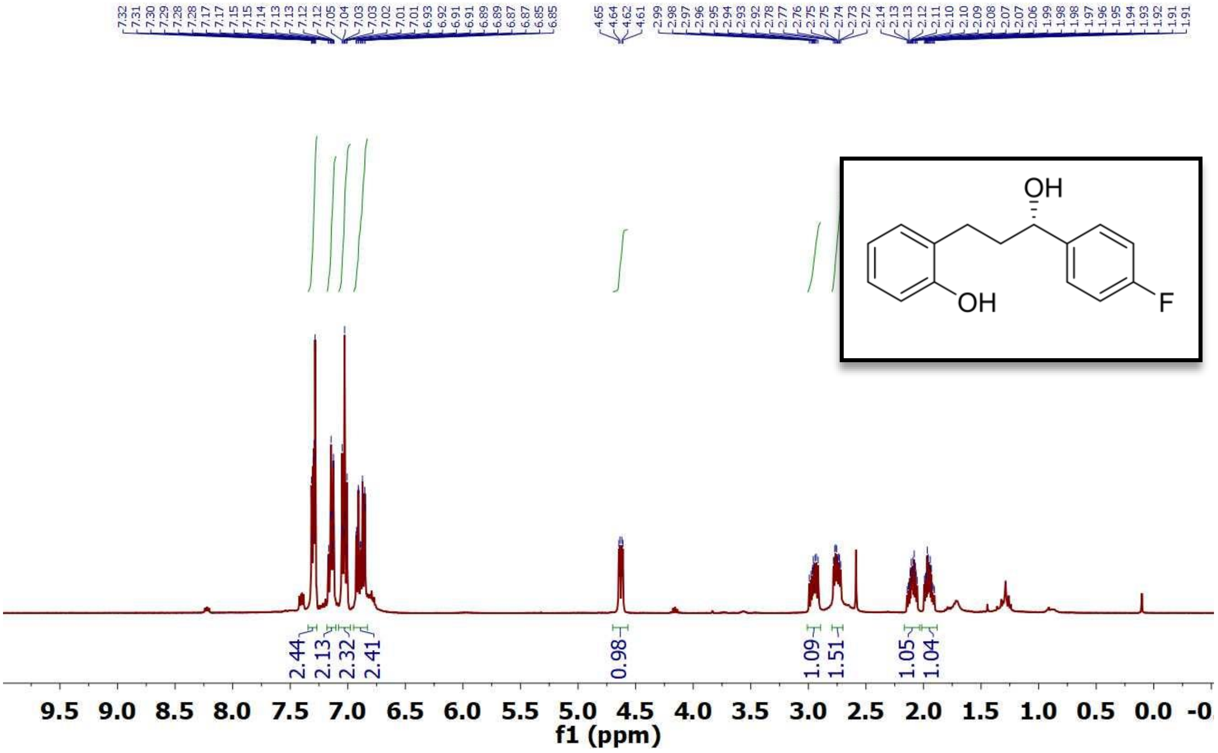

AB7-19-AC1-170622.12.fid

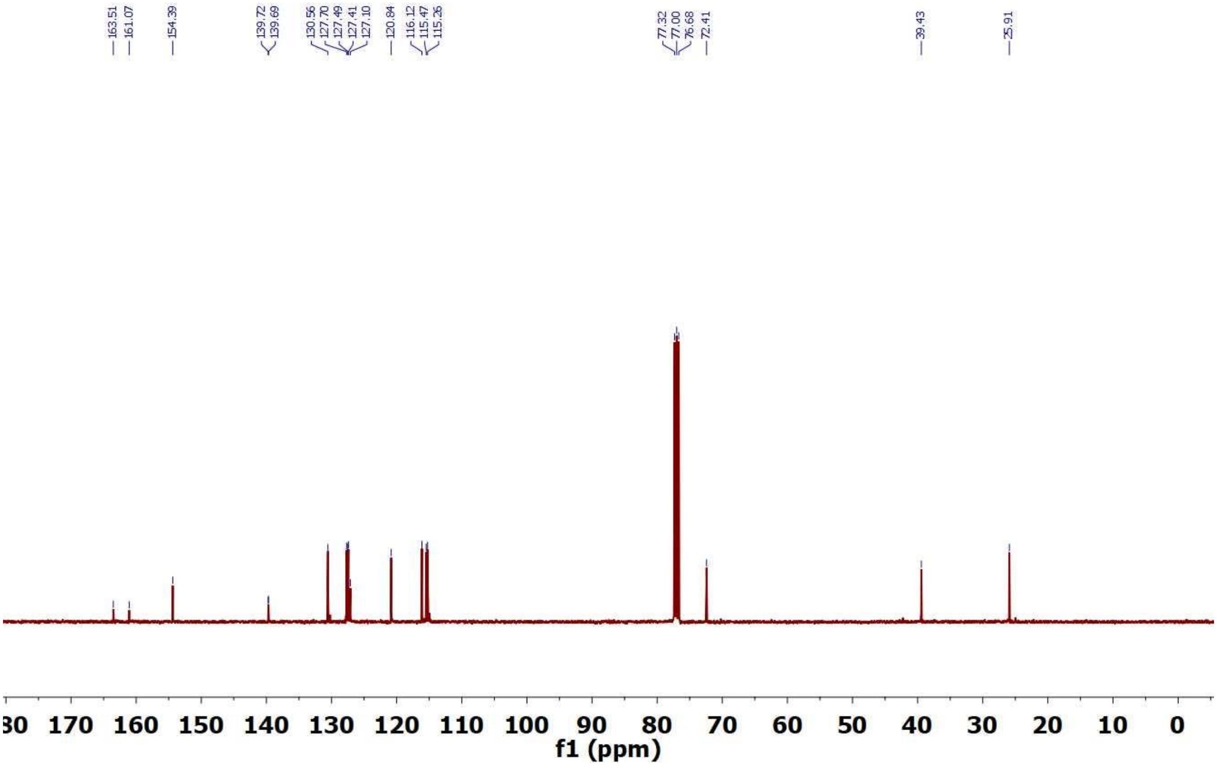

1d´ (^{1}H NMR and ^{13}C NMR, $CDCl_3$)

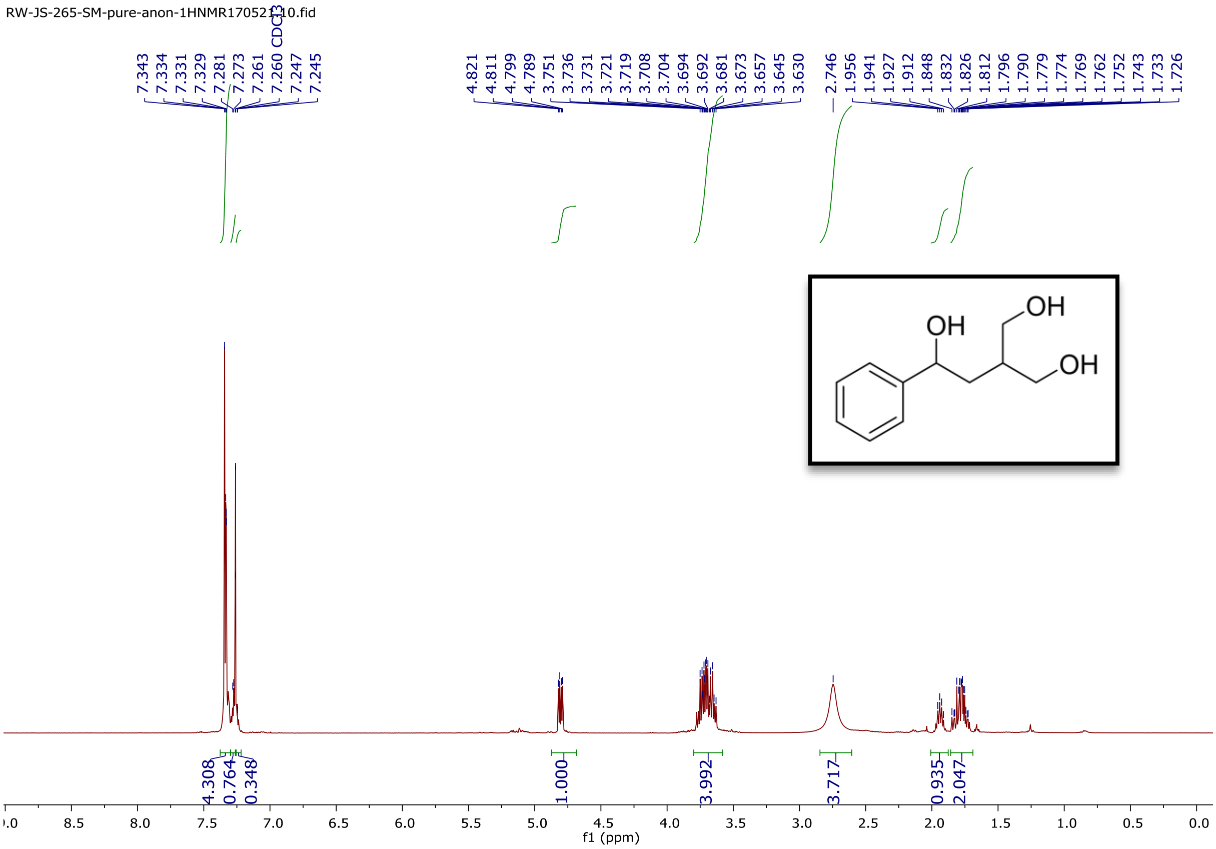

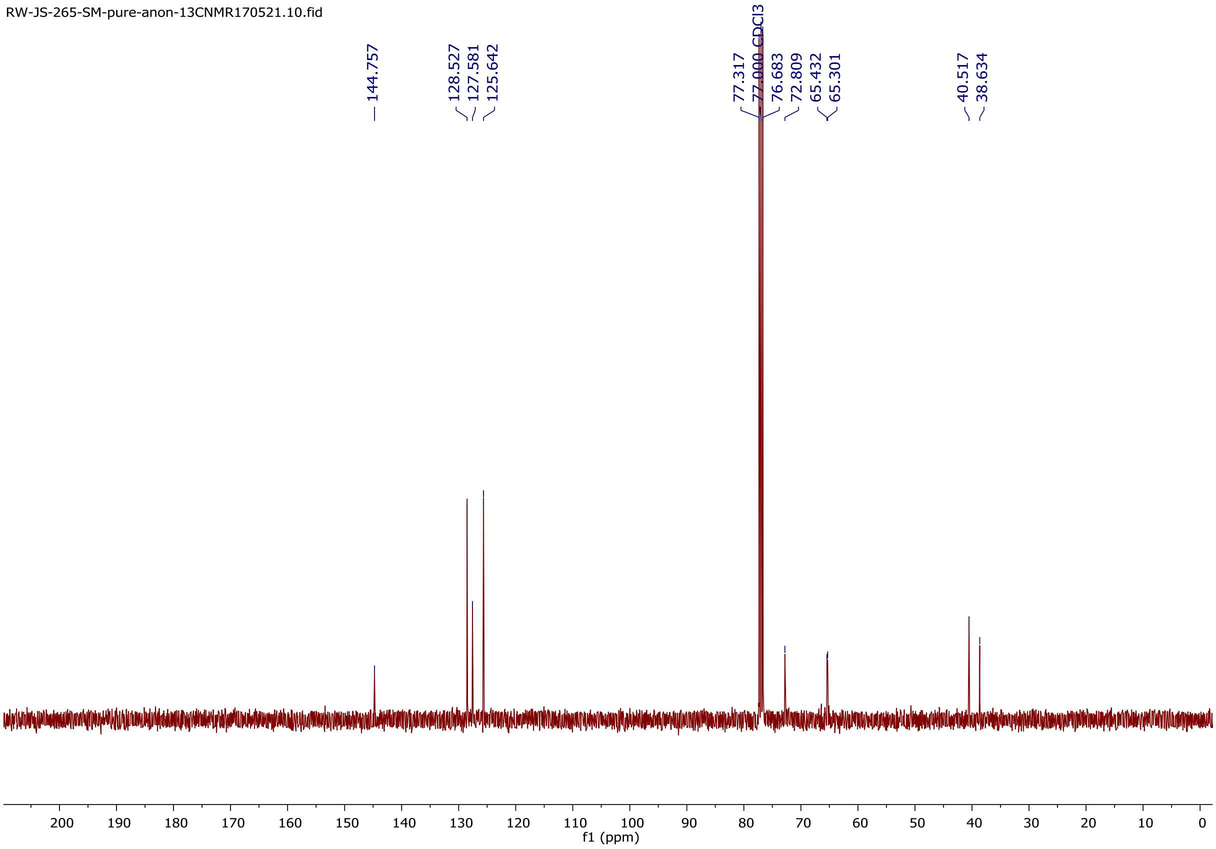

1d´´ (^{1}H NMR and ^{13}C NMR, $CDCl_3$)

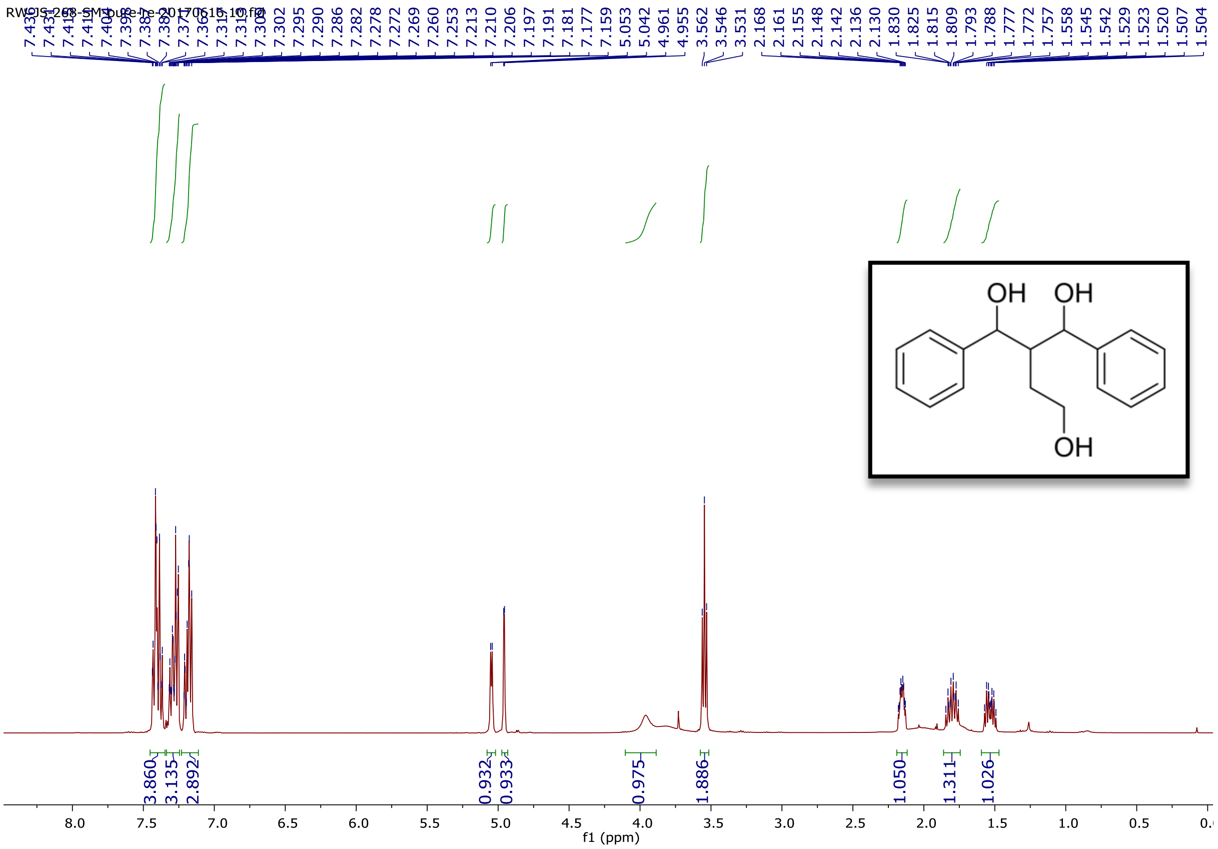

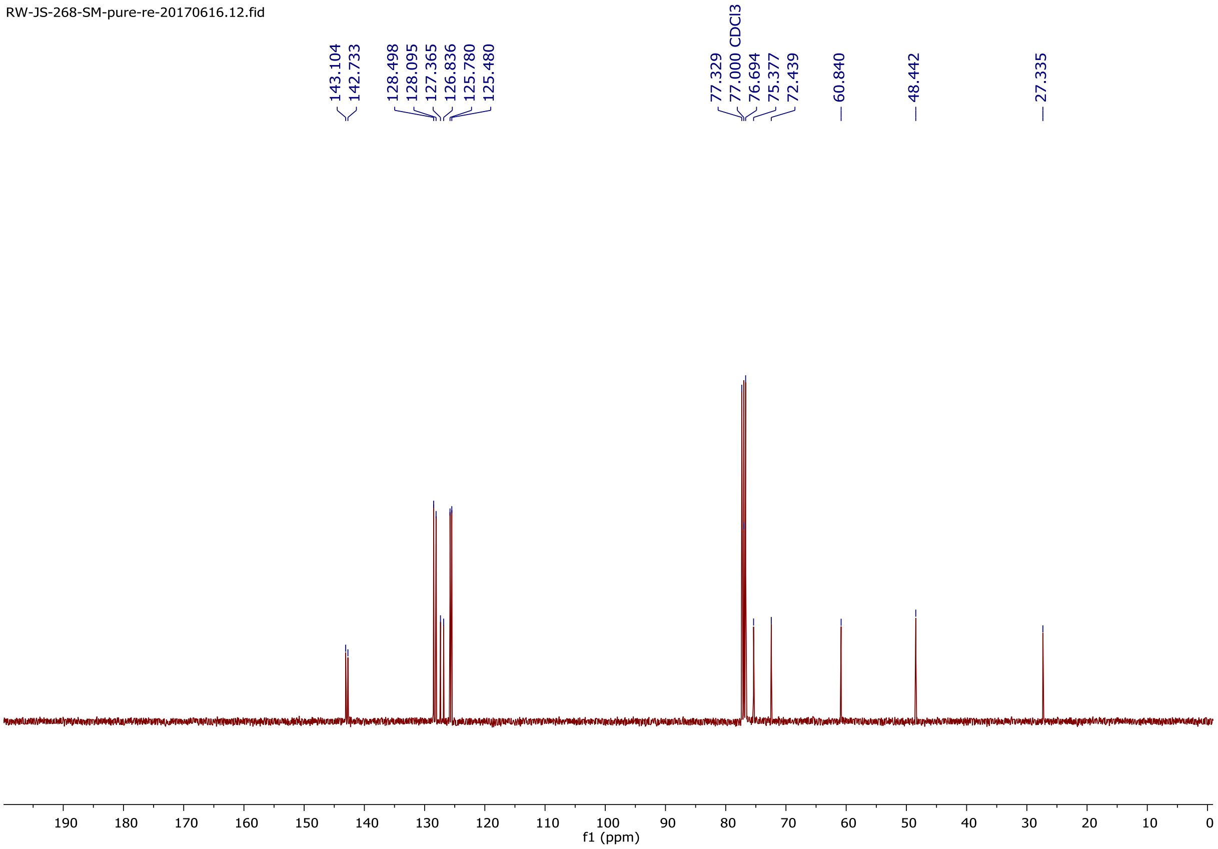

1s (^{1}H NMR and ^{13}C NMR, $CDCl_3$)

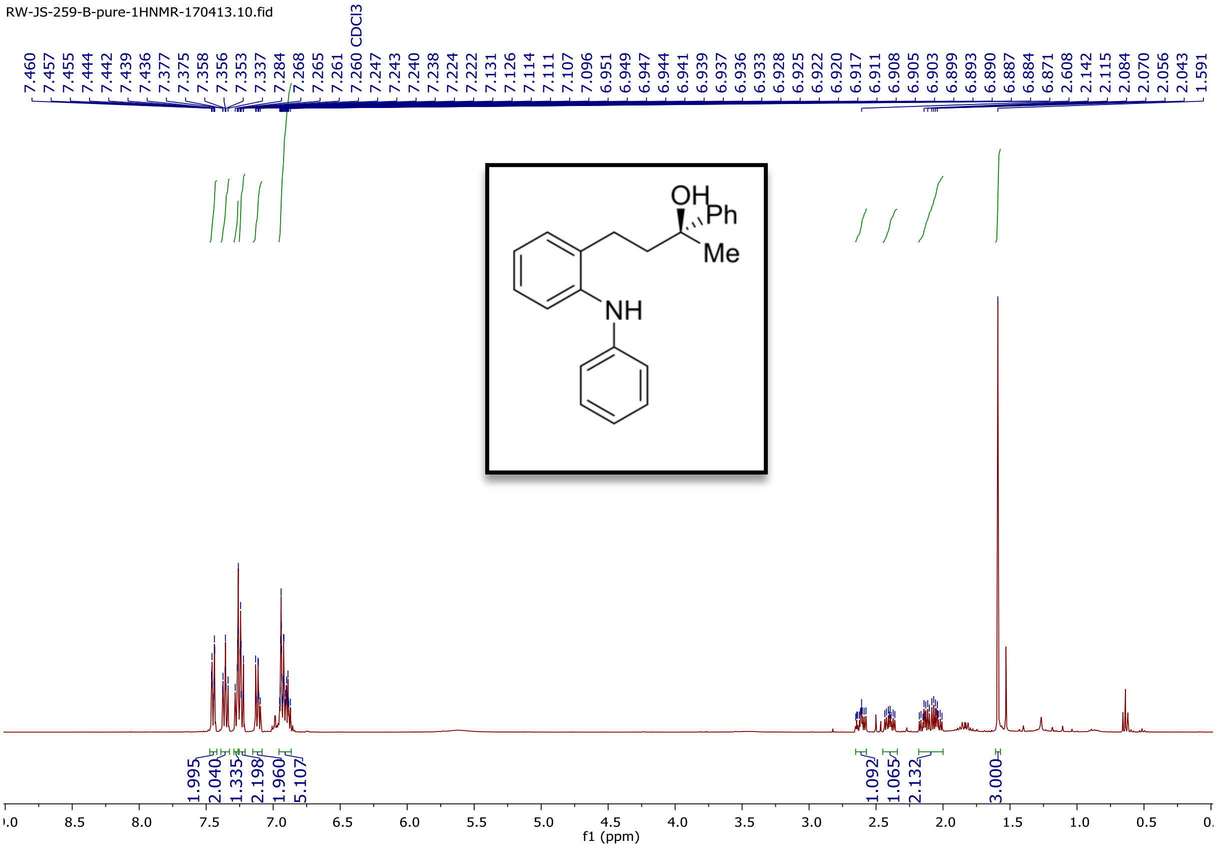

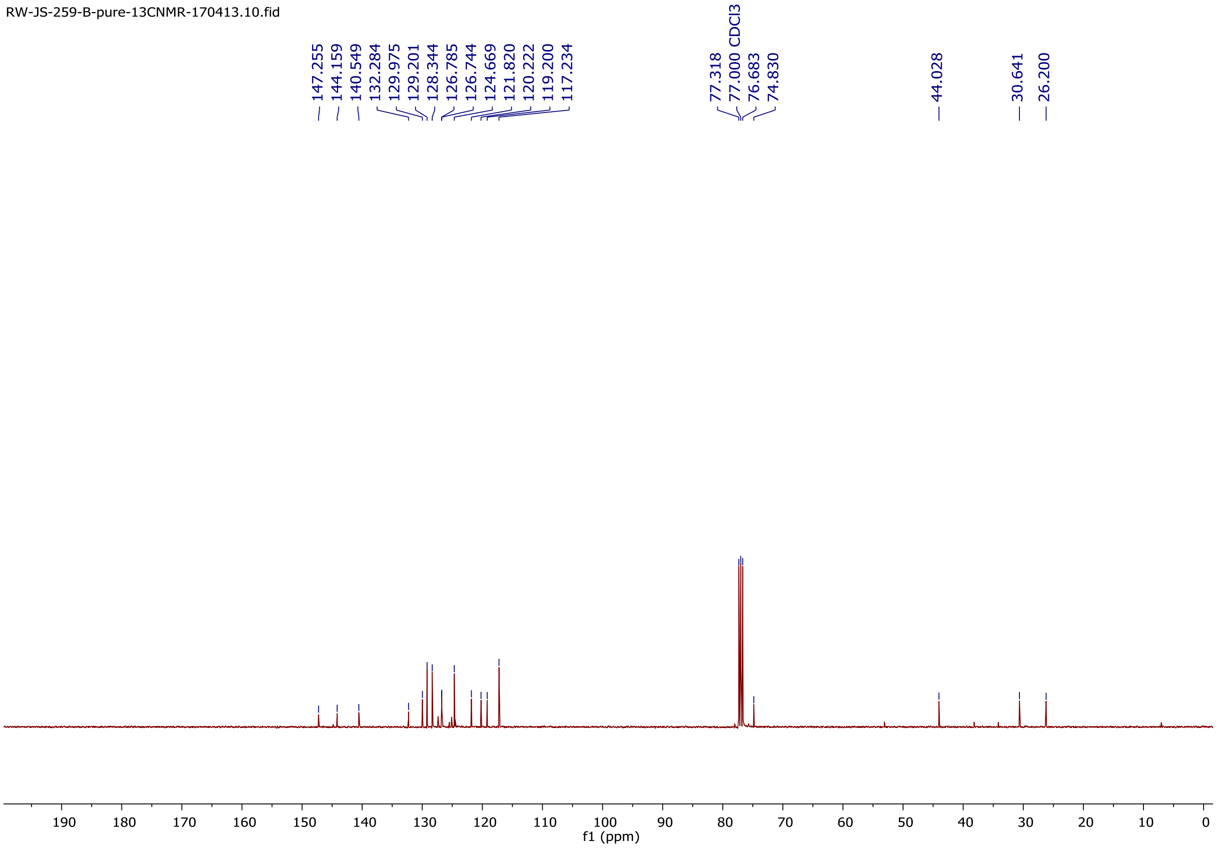

1t (^{1}H NMR and ^{13}C NMR, $CDCl_3$)

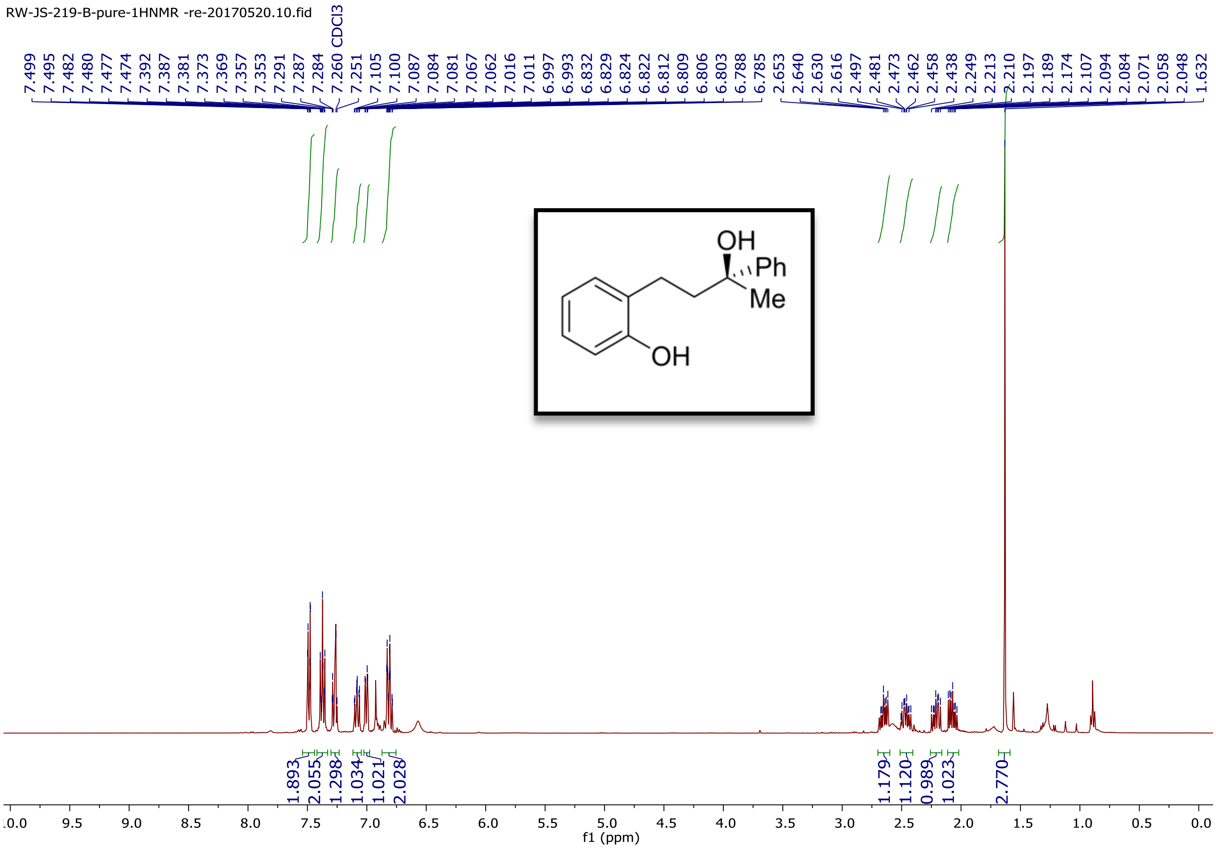

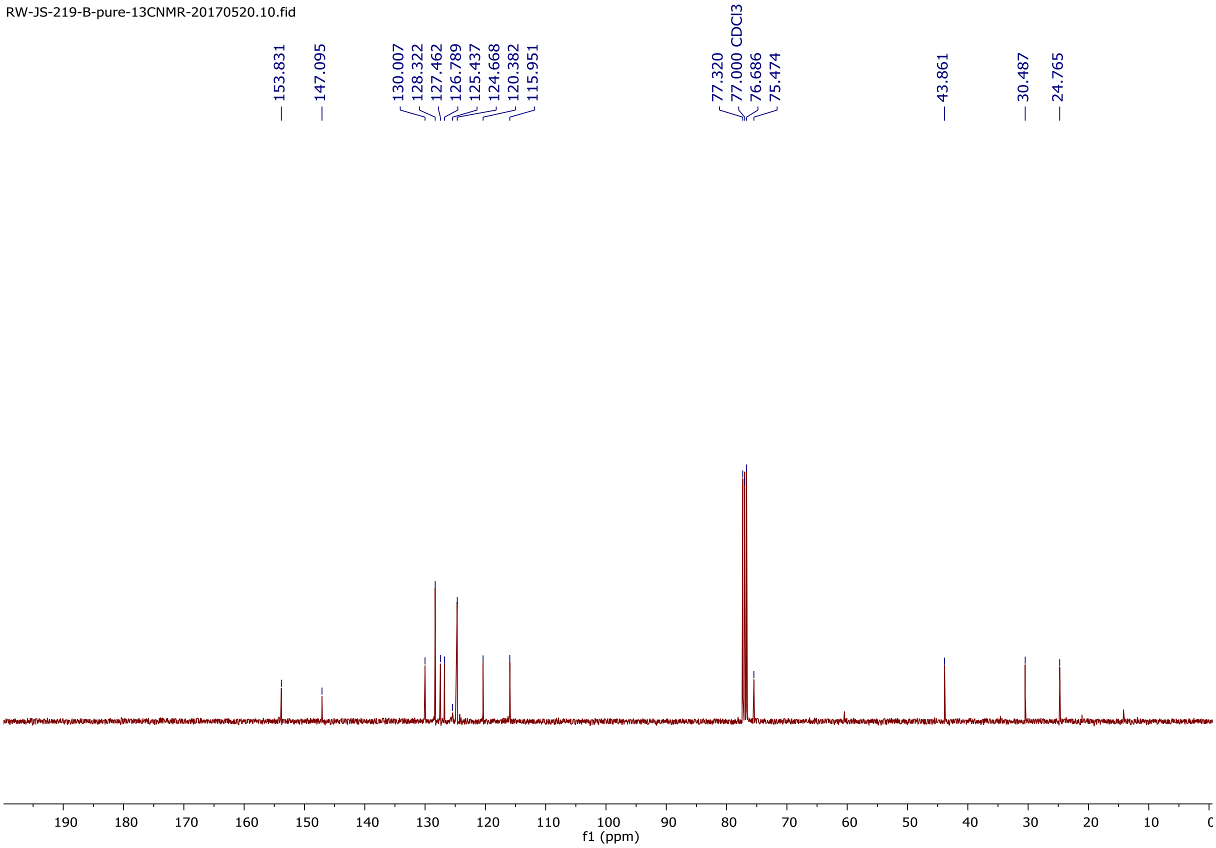

1u (^{1}H NMR and ^{13}C NMR, $CDCl_3$)

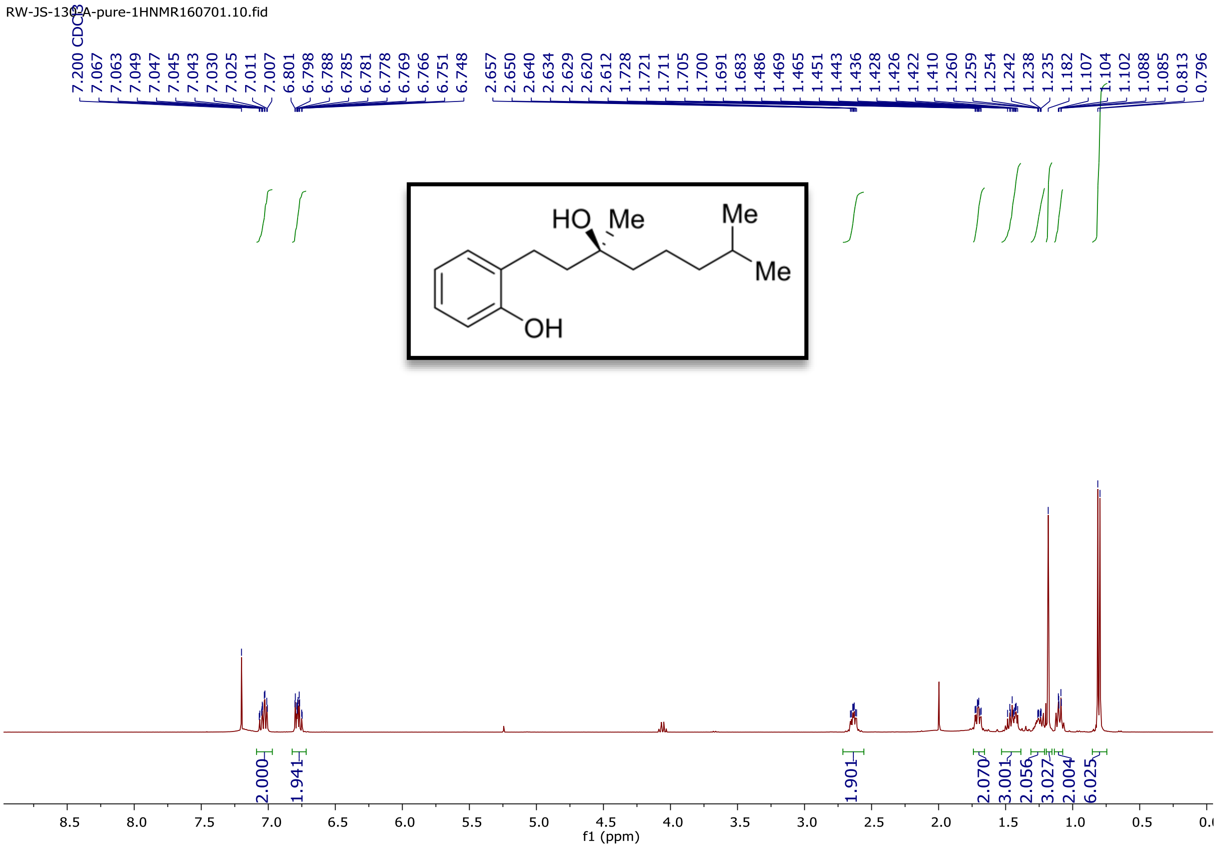

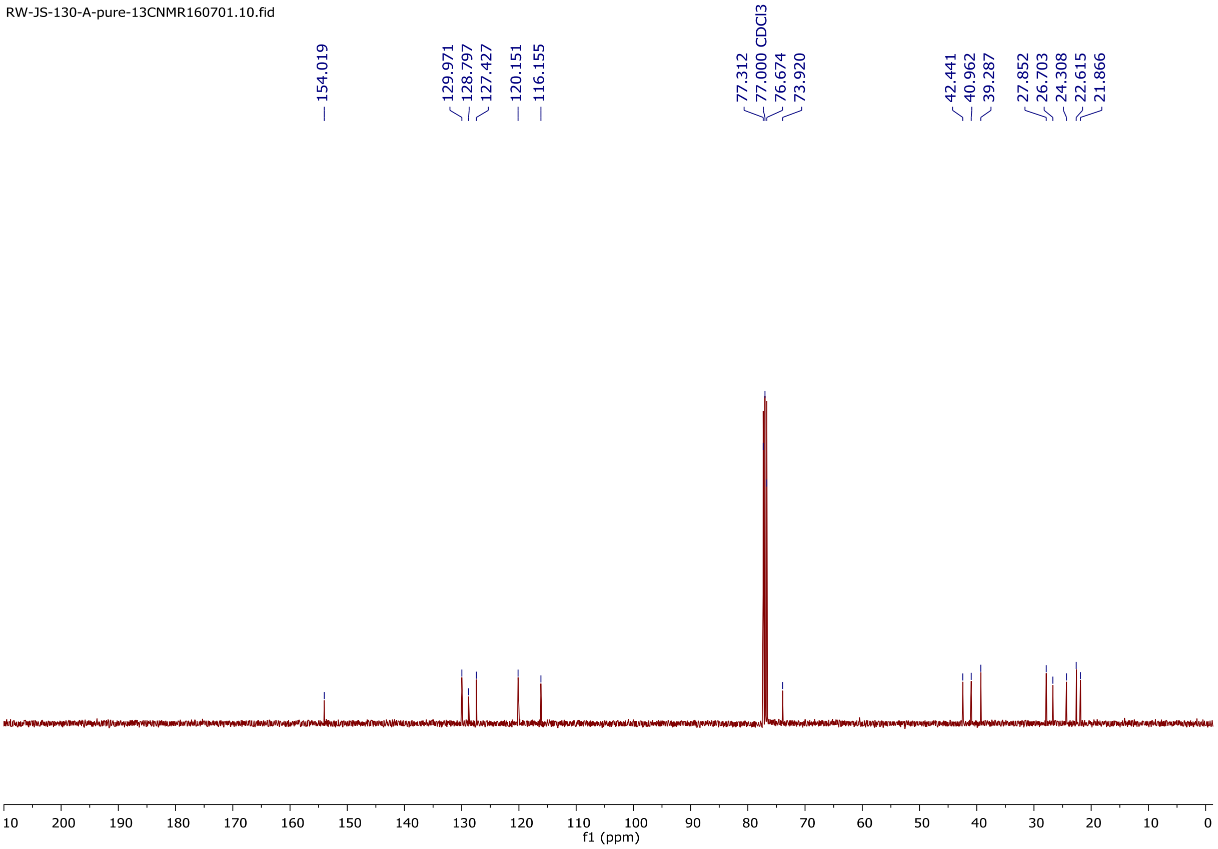

1v (^{1}H NMR and ^{13}C NMR, $CDCl_3$)

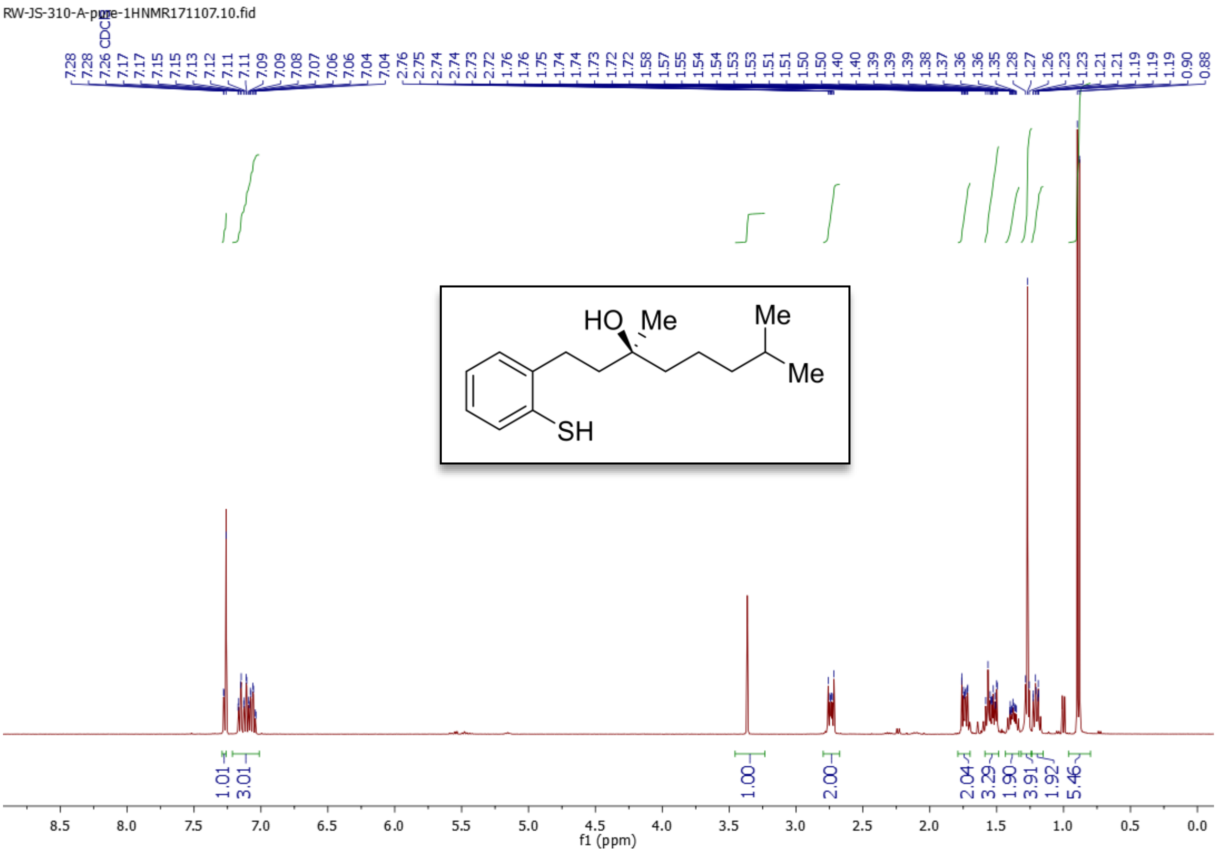

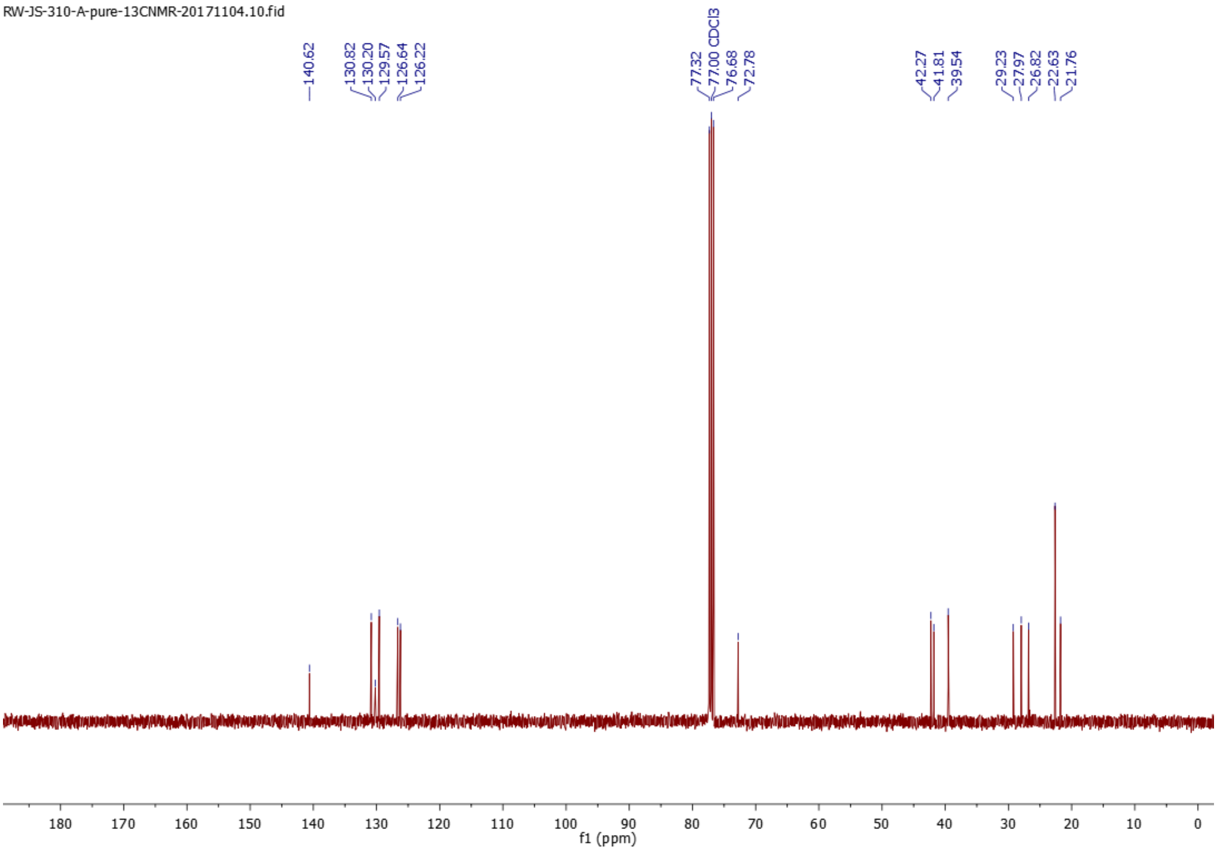

1u′ (^{1}H NMR and ^{13}C NMR, $CDCl_3$)

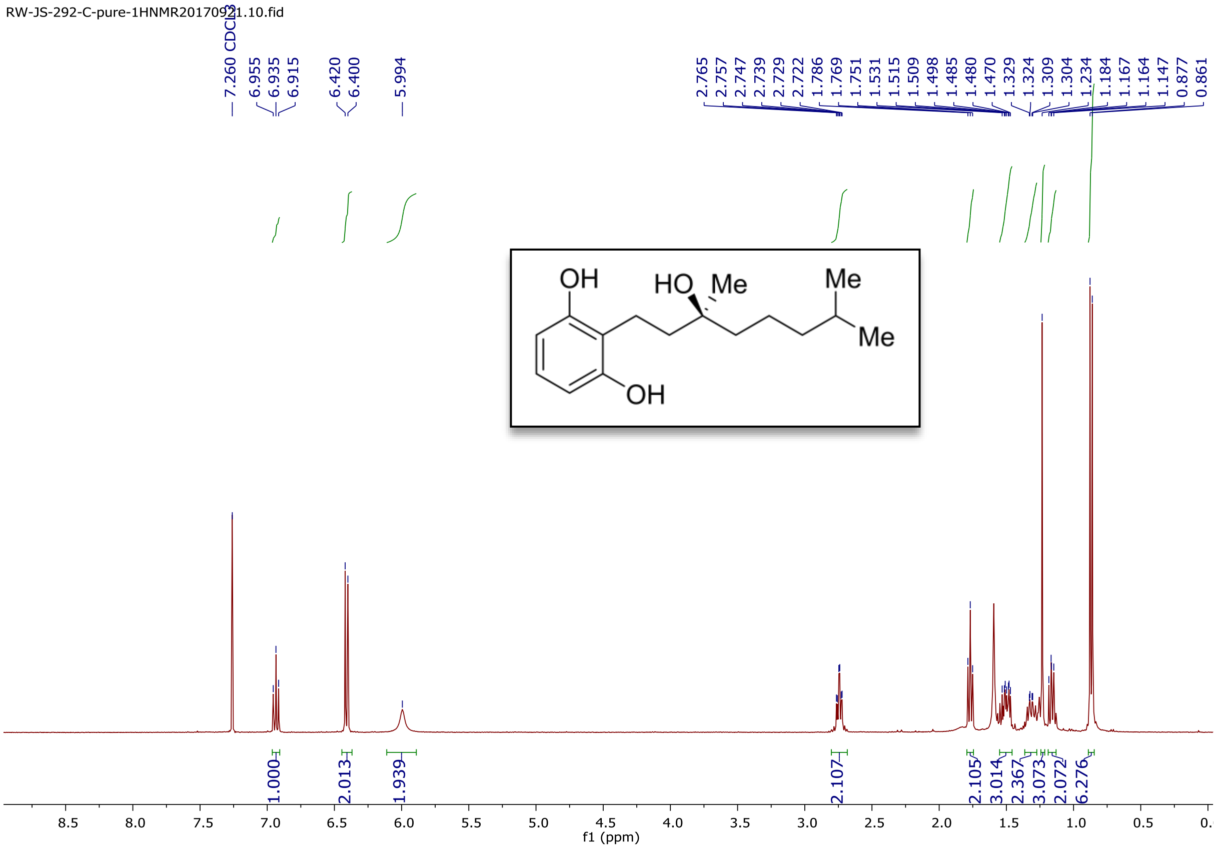

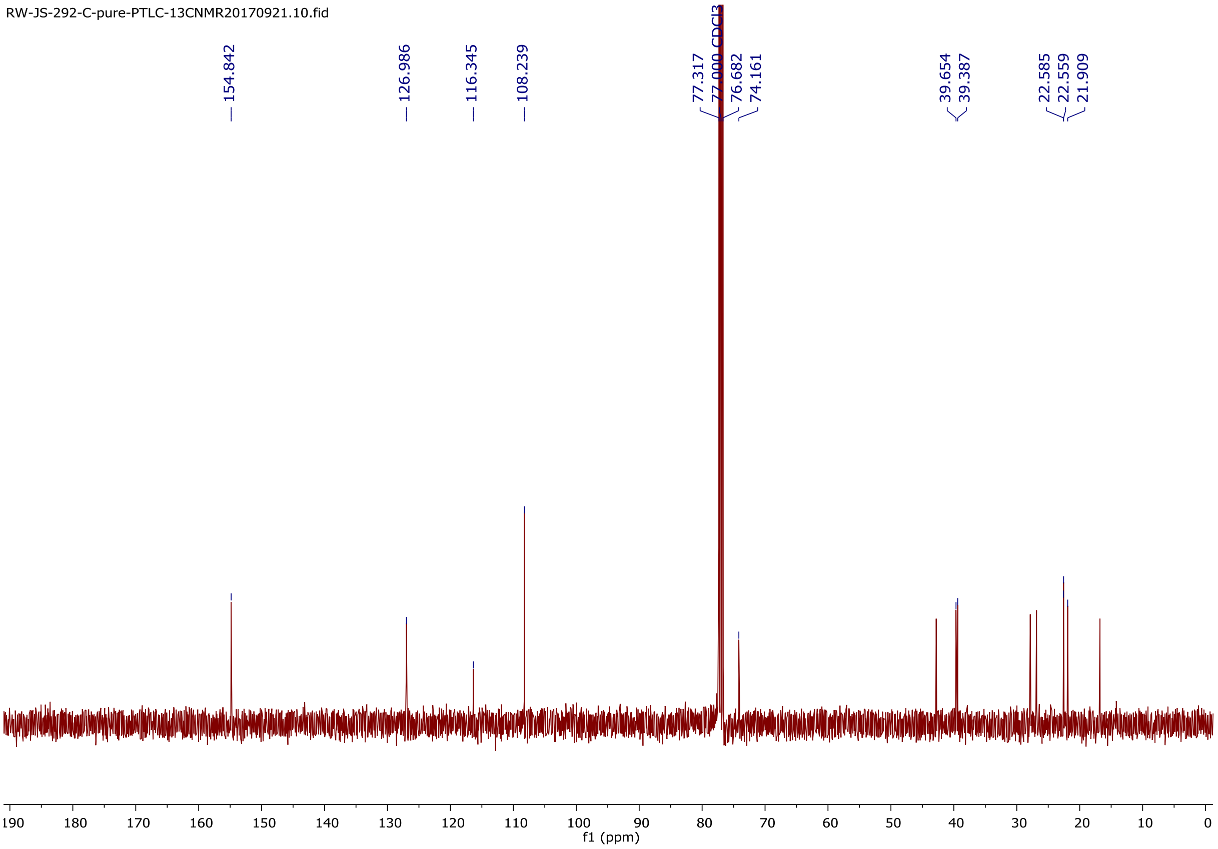

2a (^{1}H NMR and ^{13}C NMR, $CDCl_3$)

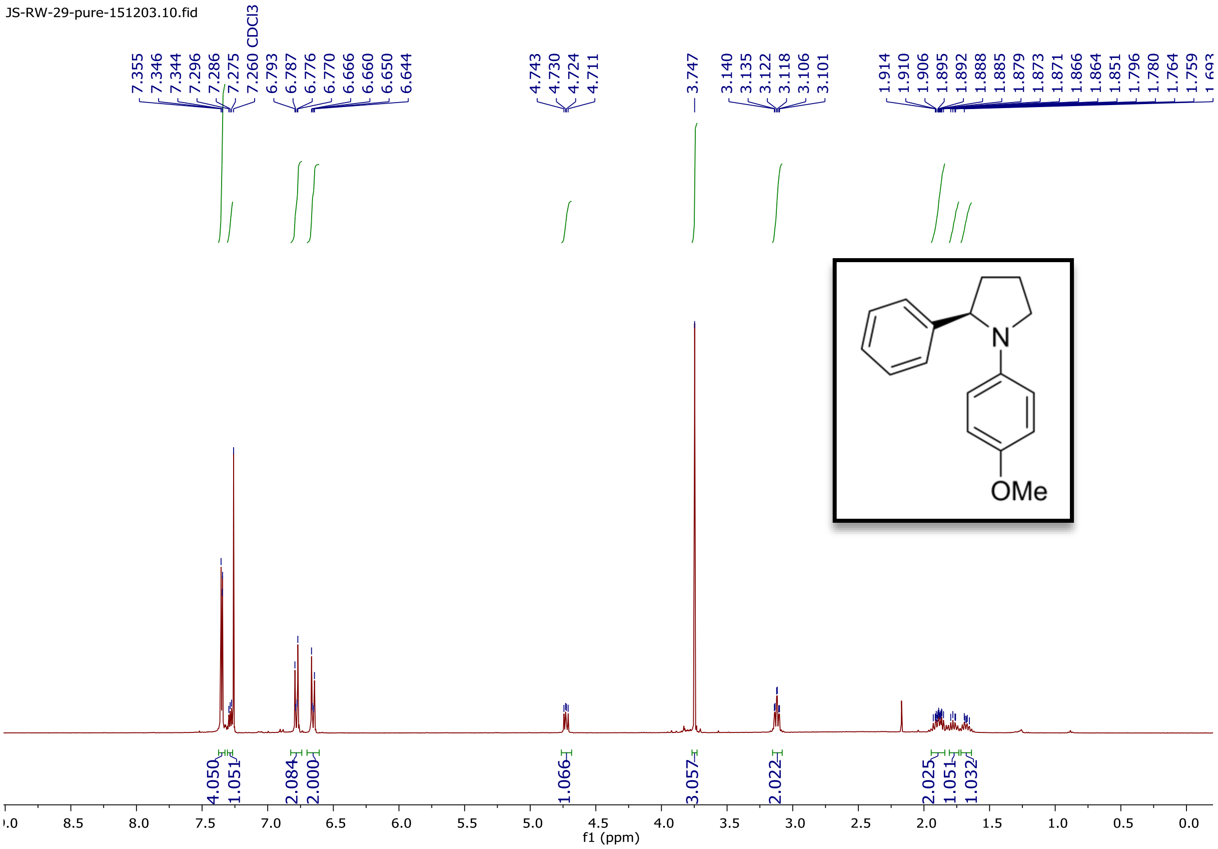

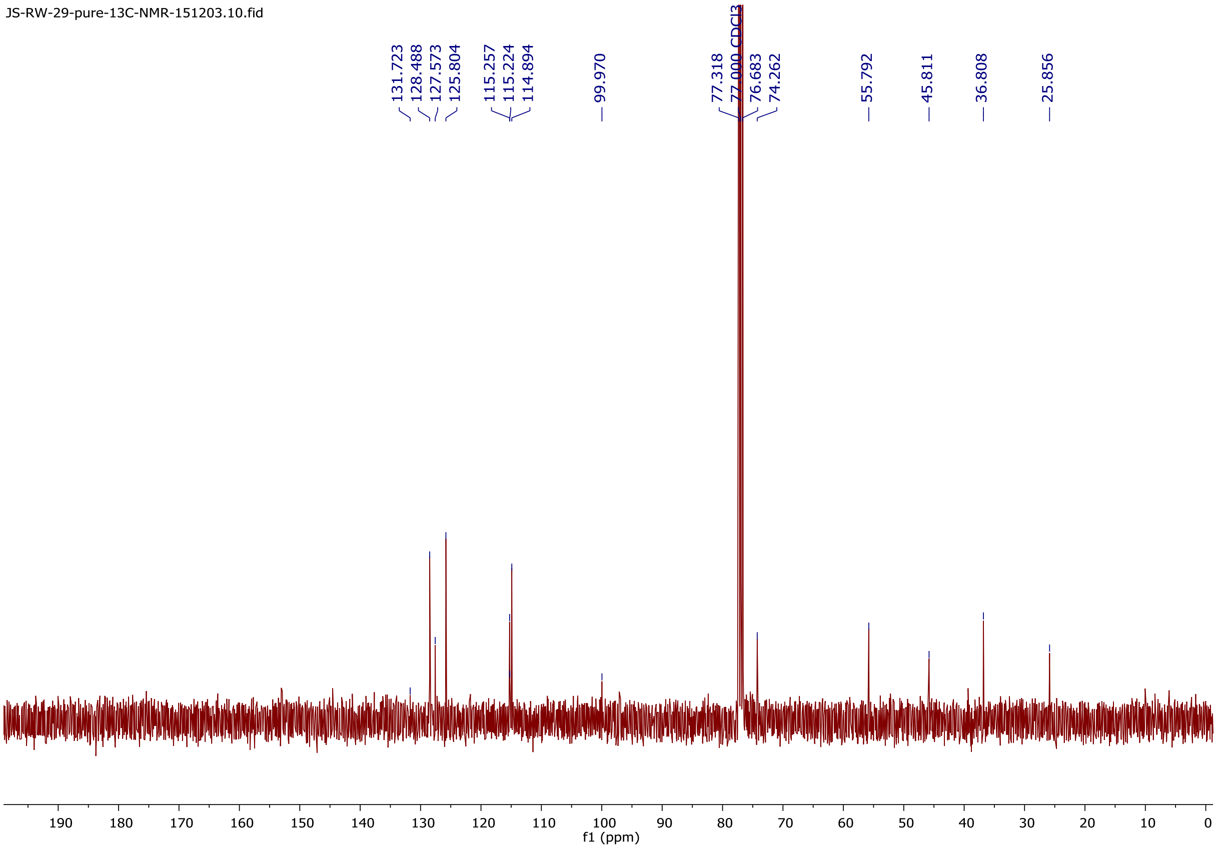

2b (^{1}H NMR and ^{13}C NMR, $CDCl_3$)

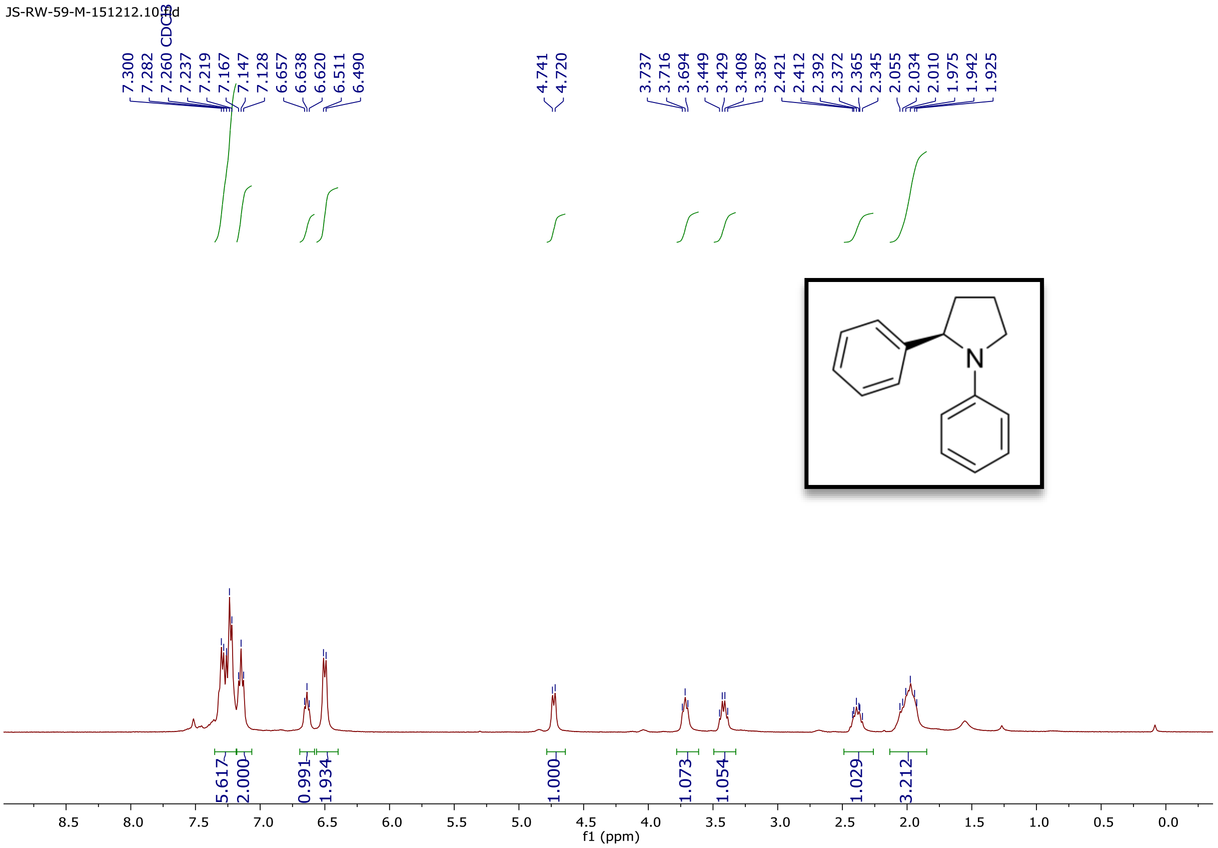

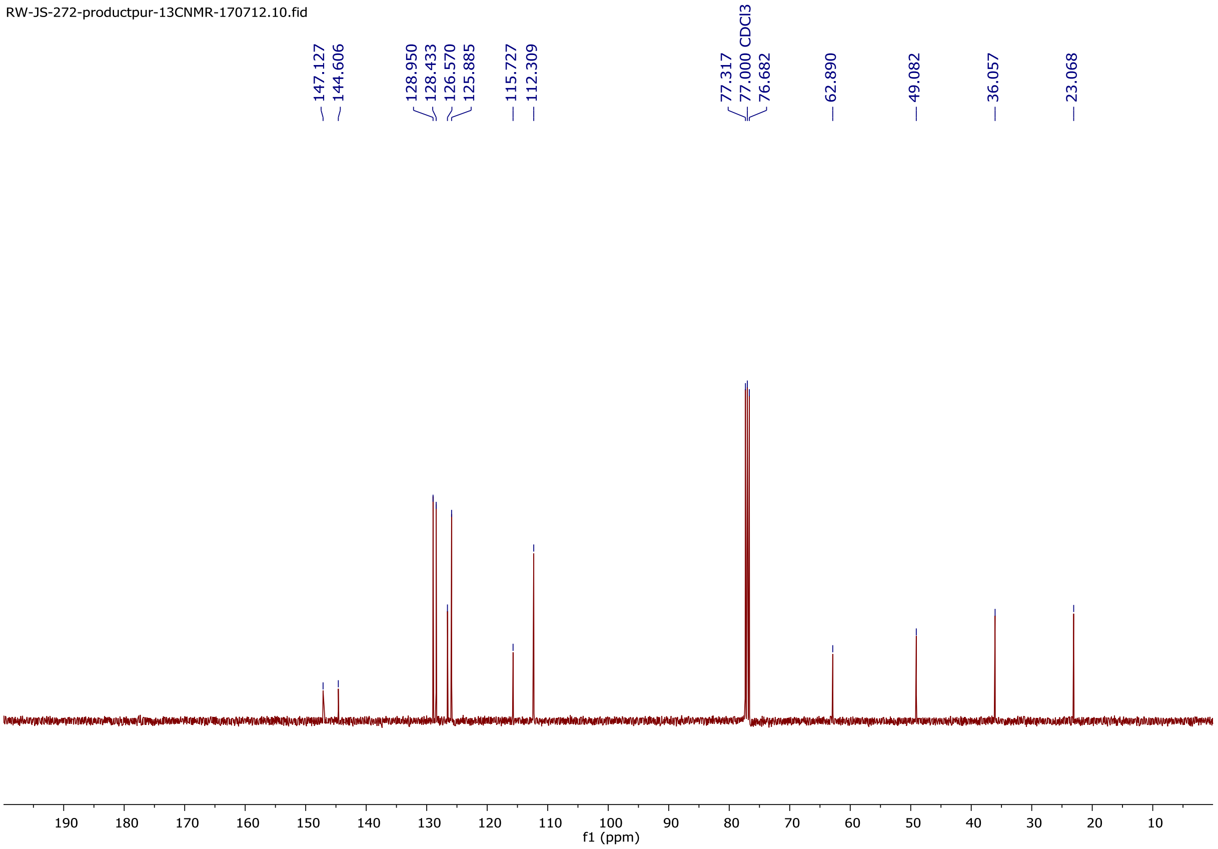

2c (^{1}H NMR and ^{13}C NMR, $CDCl_3$)

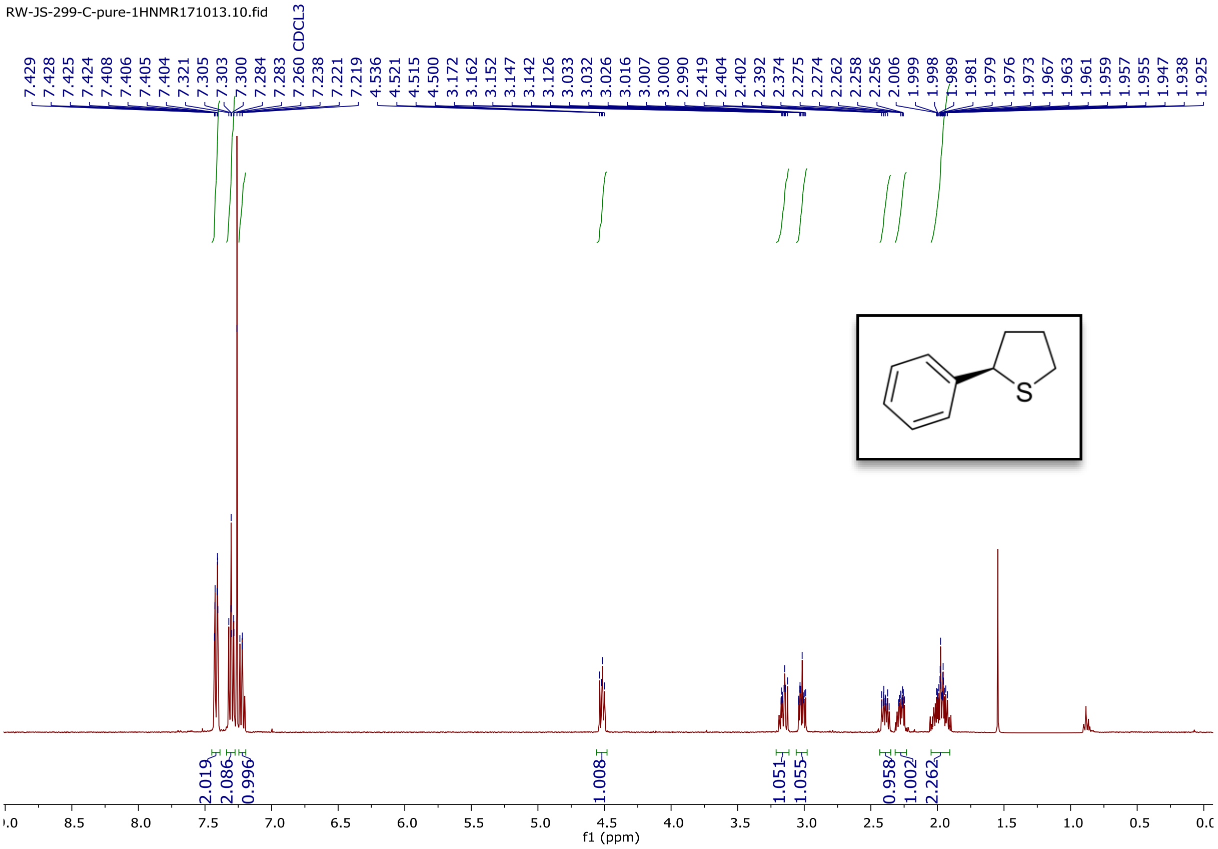

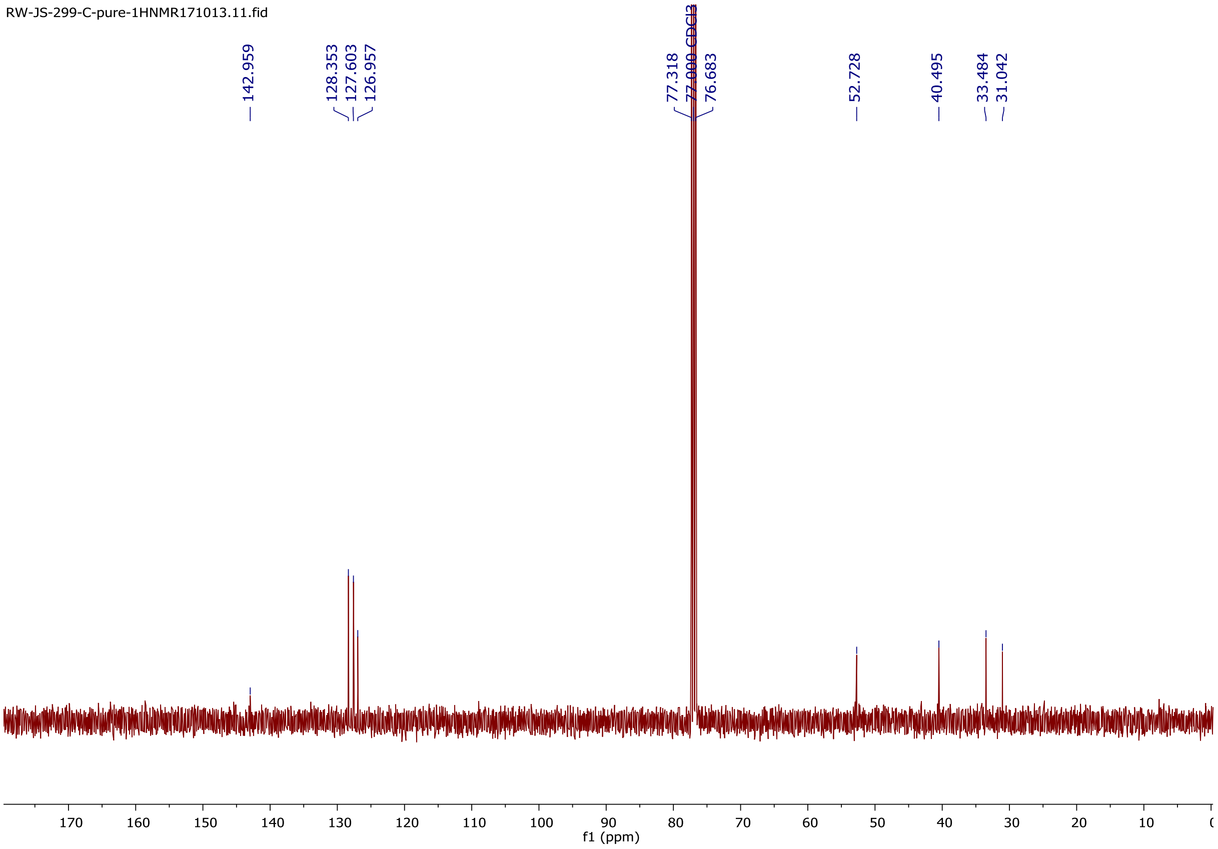

2d (^{1}H NMR and ^{13}C NMR, $CDCl_3$)

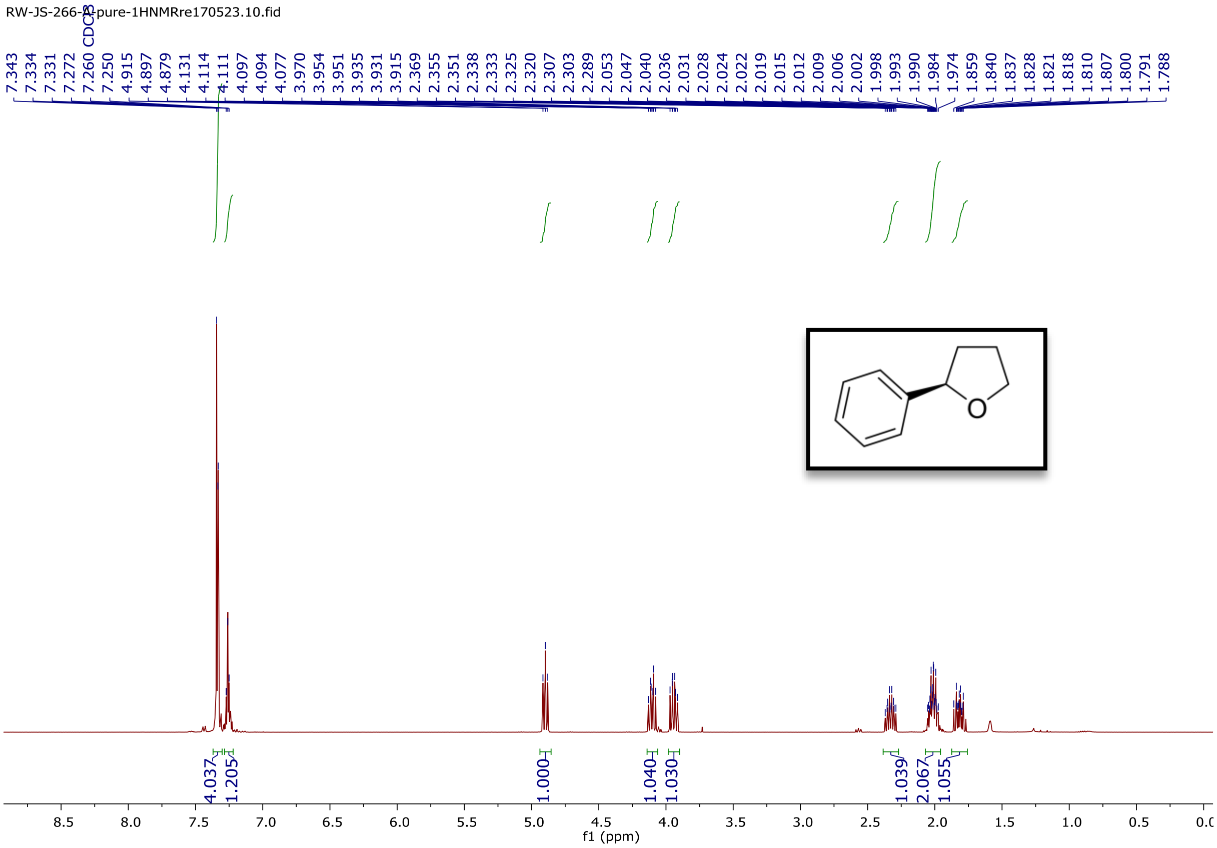

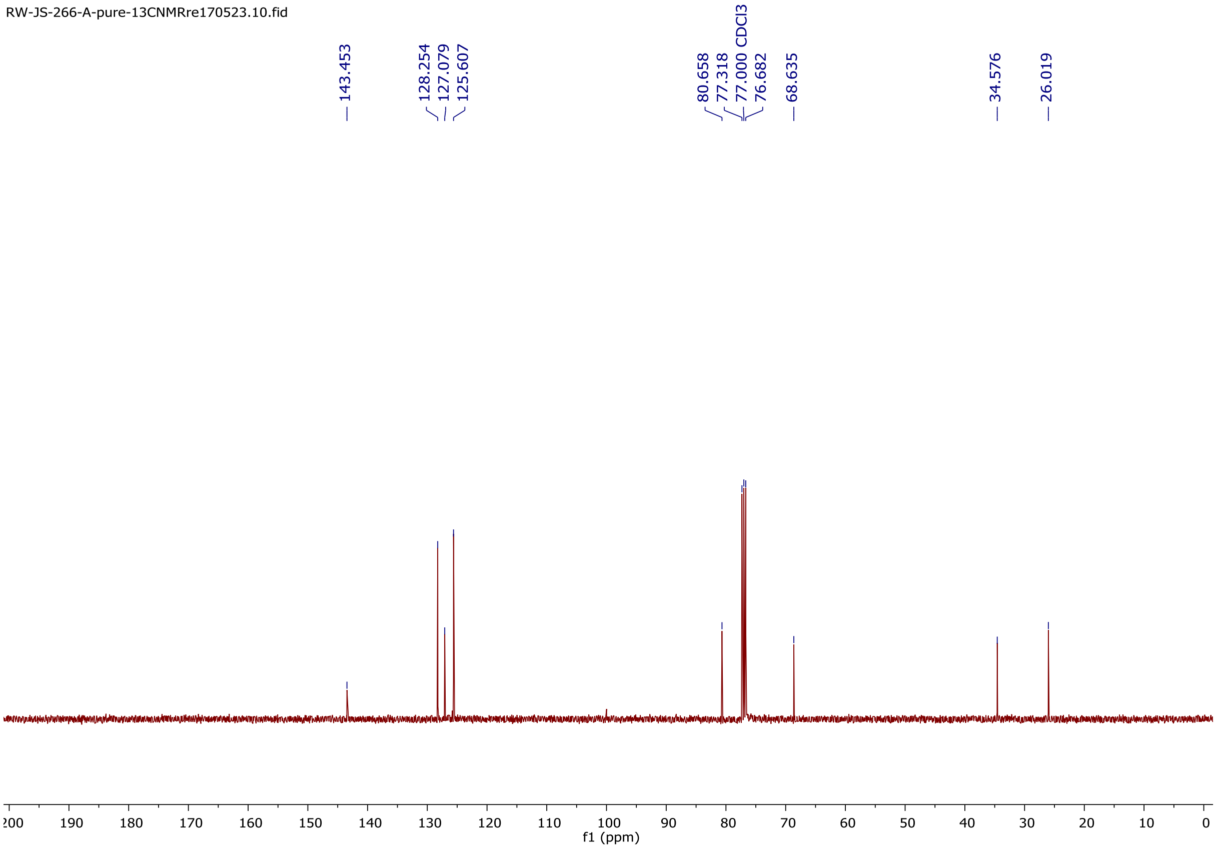

2e (^{1}H NMR and ^{13}C NMR, $CDCl_3$)

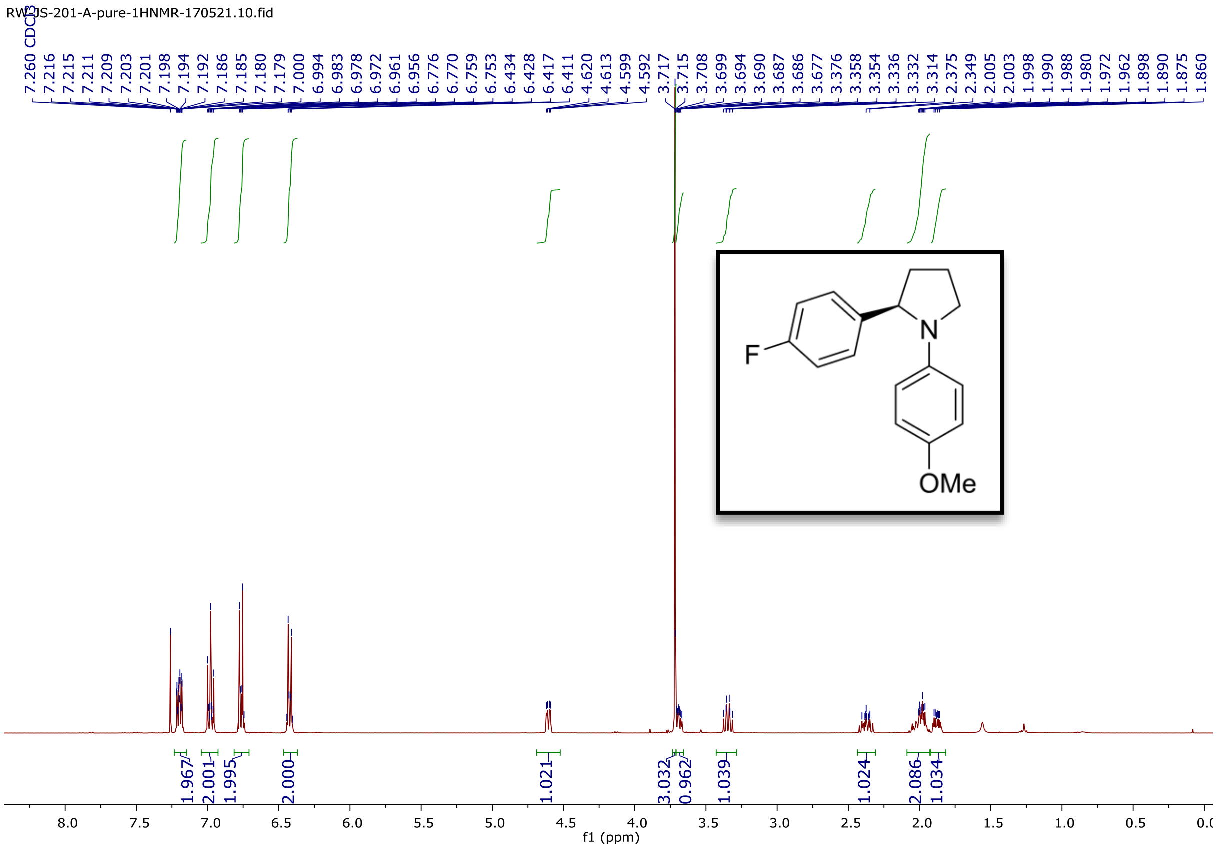

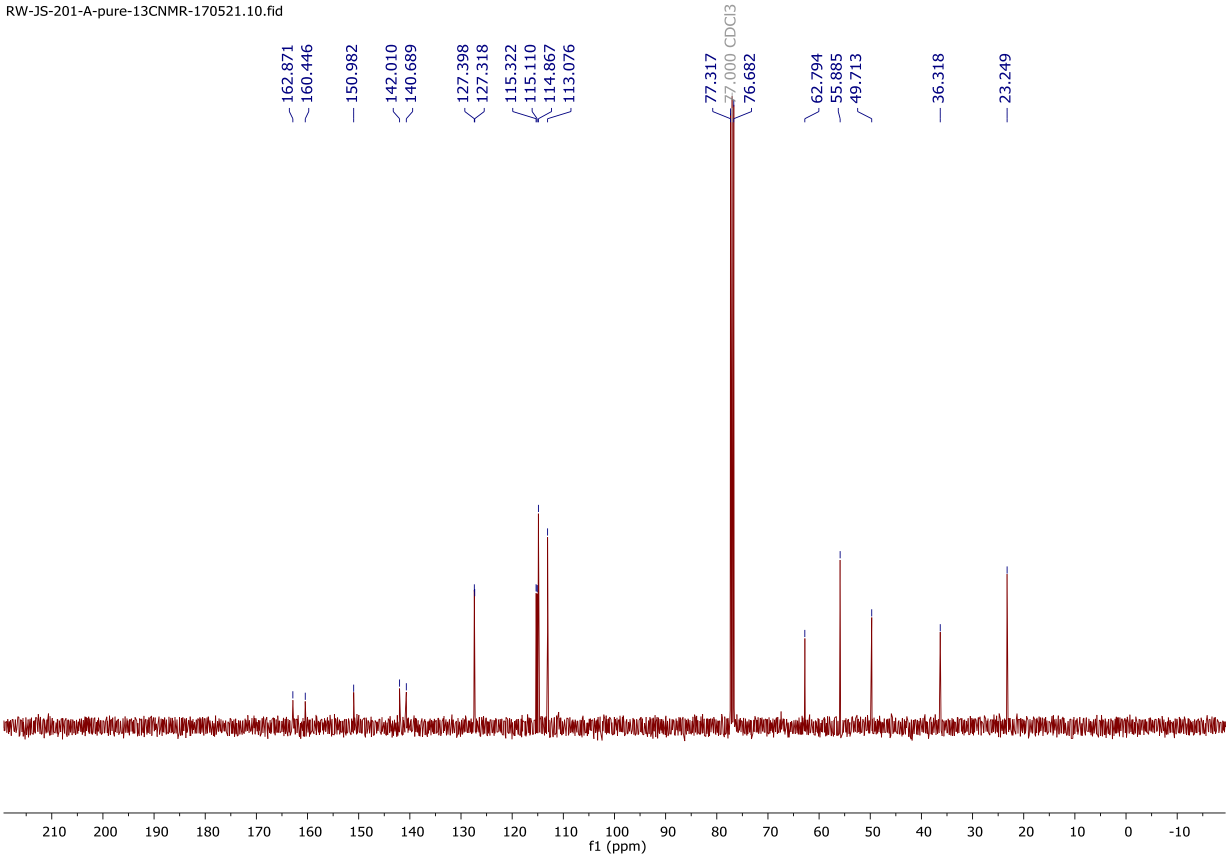

2f (^{1}H NMR and ^{13}C NMR, $CDCl_3$)

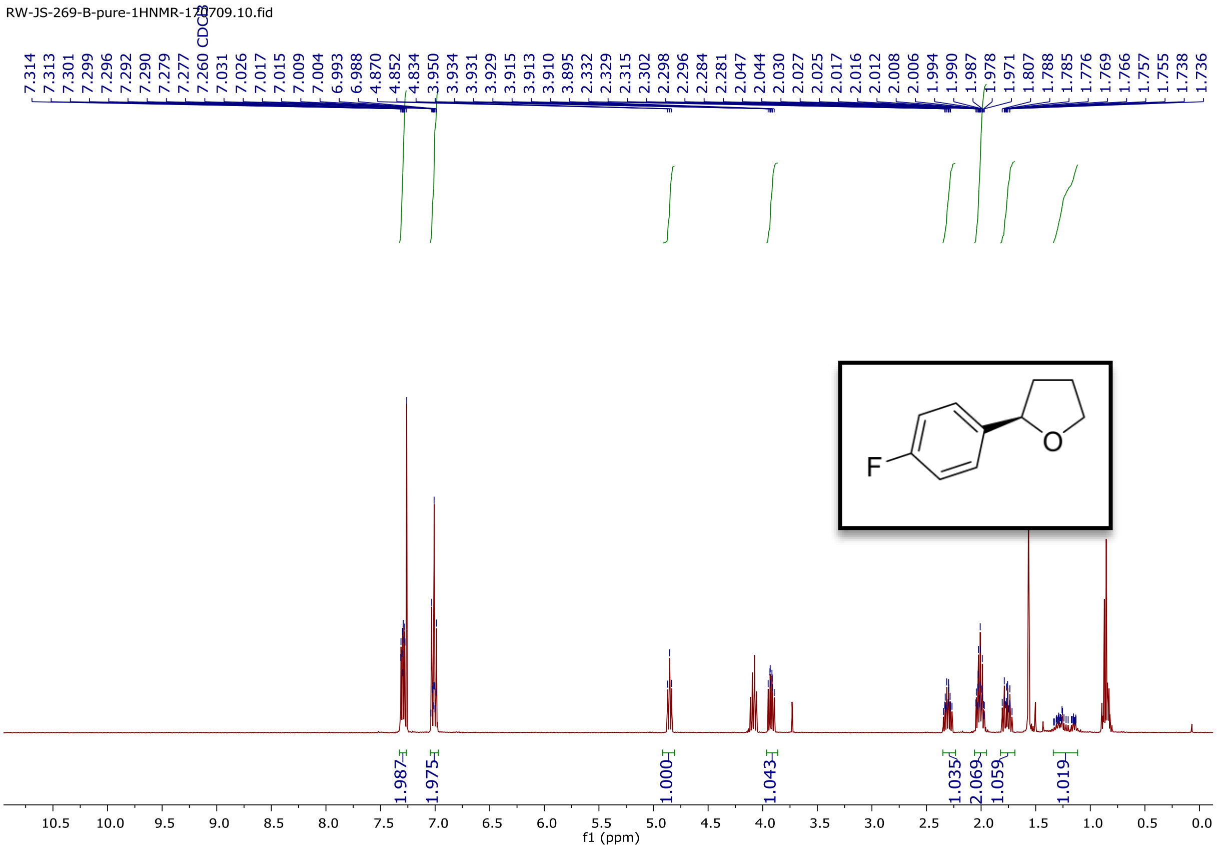

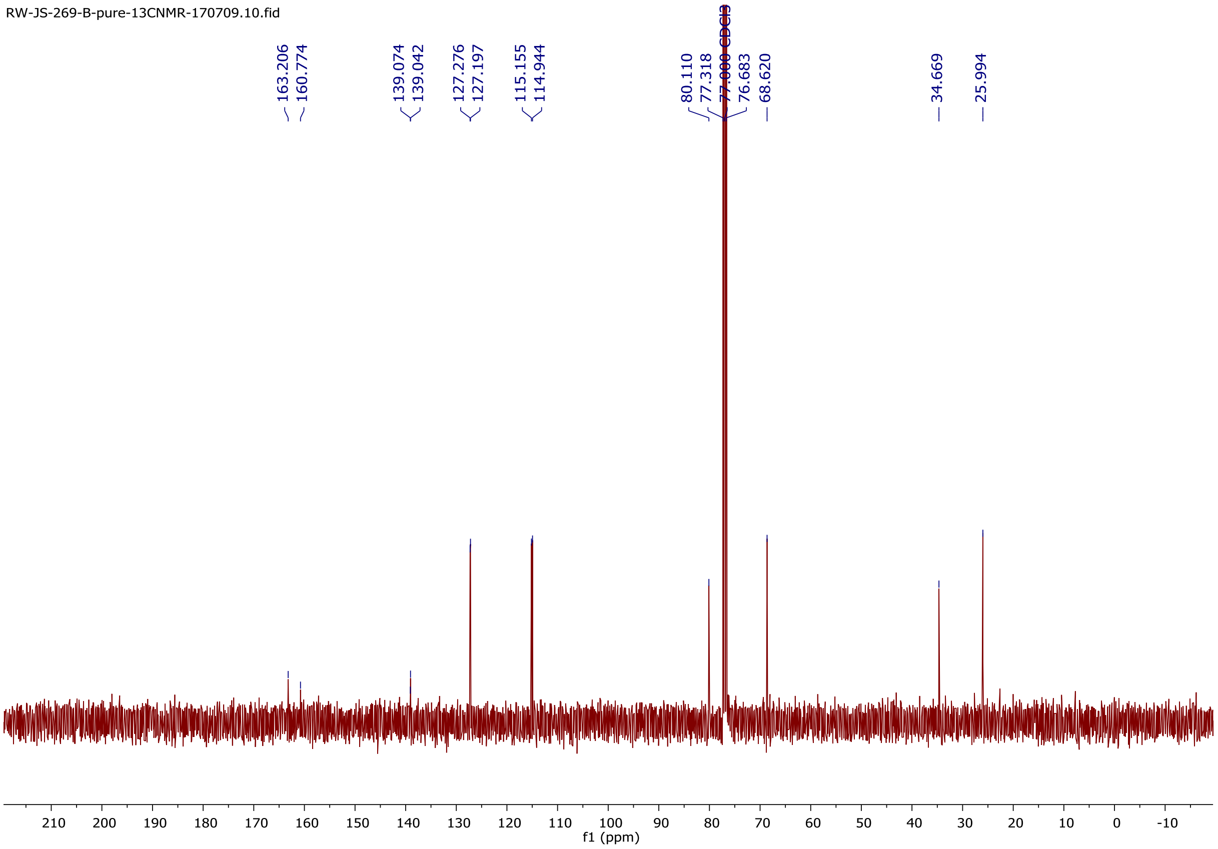

2g (^{1}H NMR and ^{13}C NMR, $CDCl_3$)

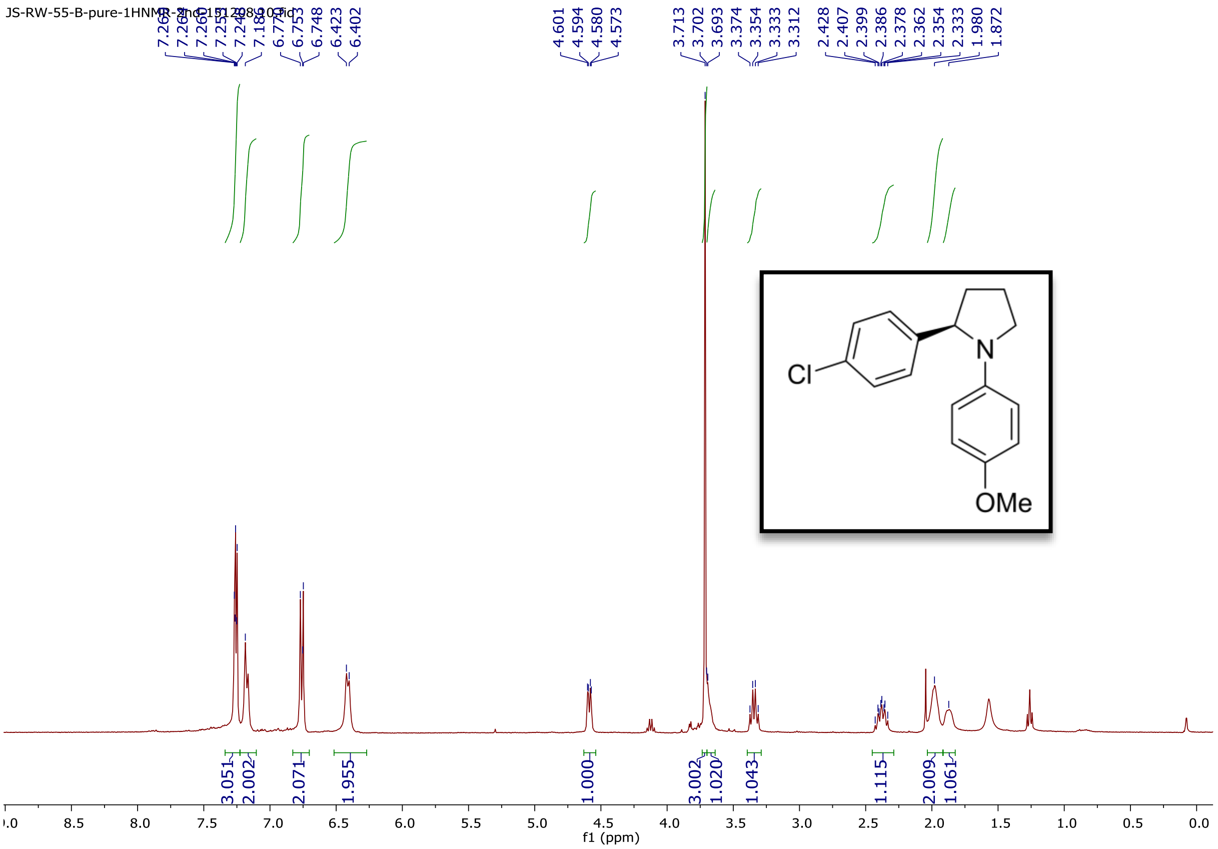

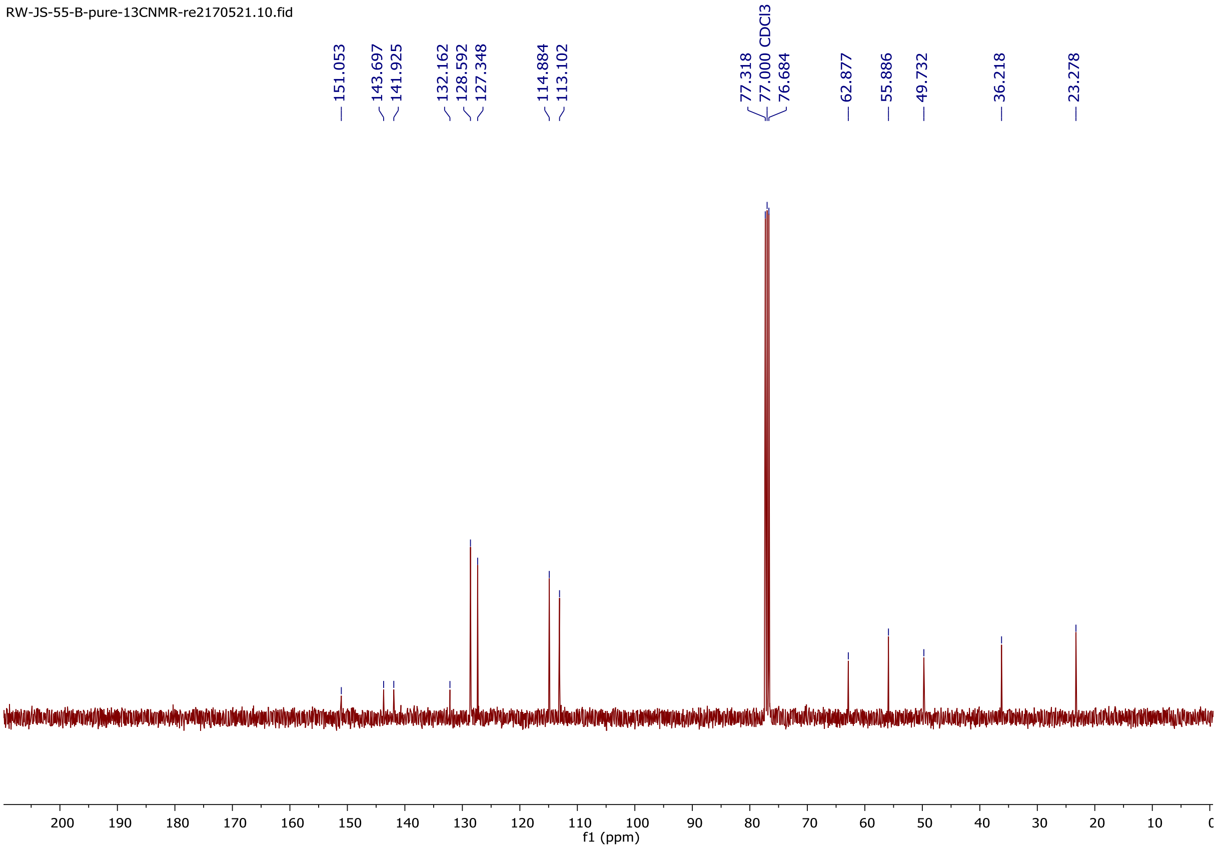

2h (^{1}H NMR and ^{13}C NMR, $CDCl_3$)

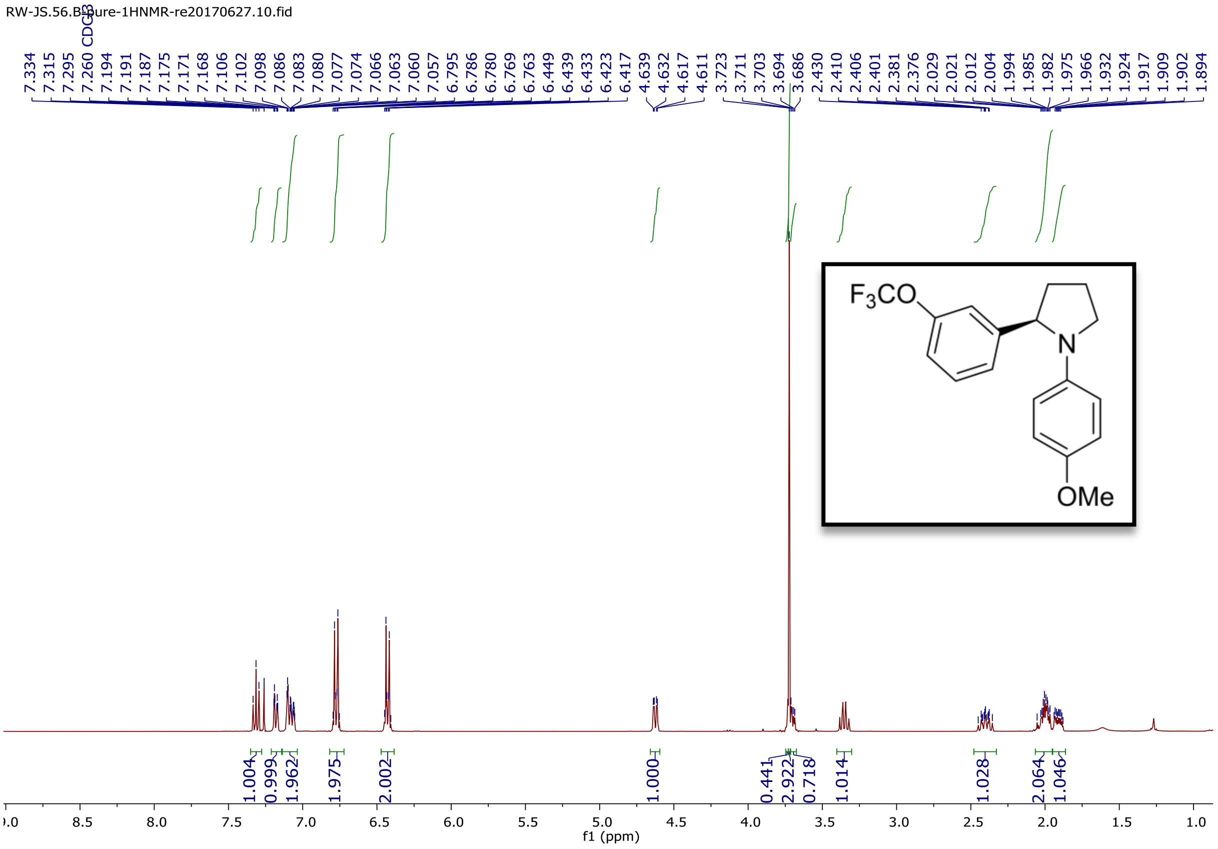

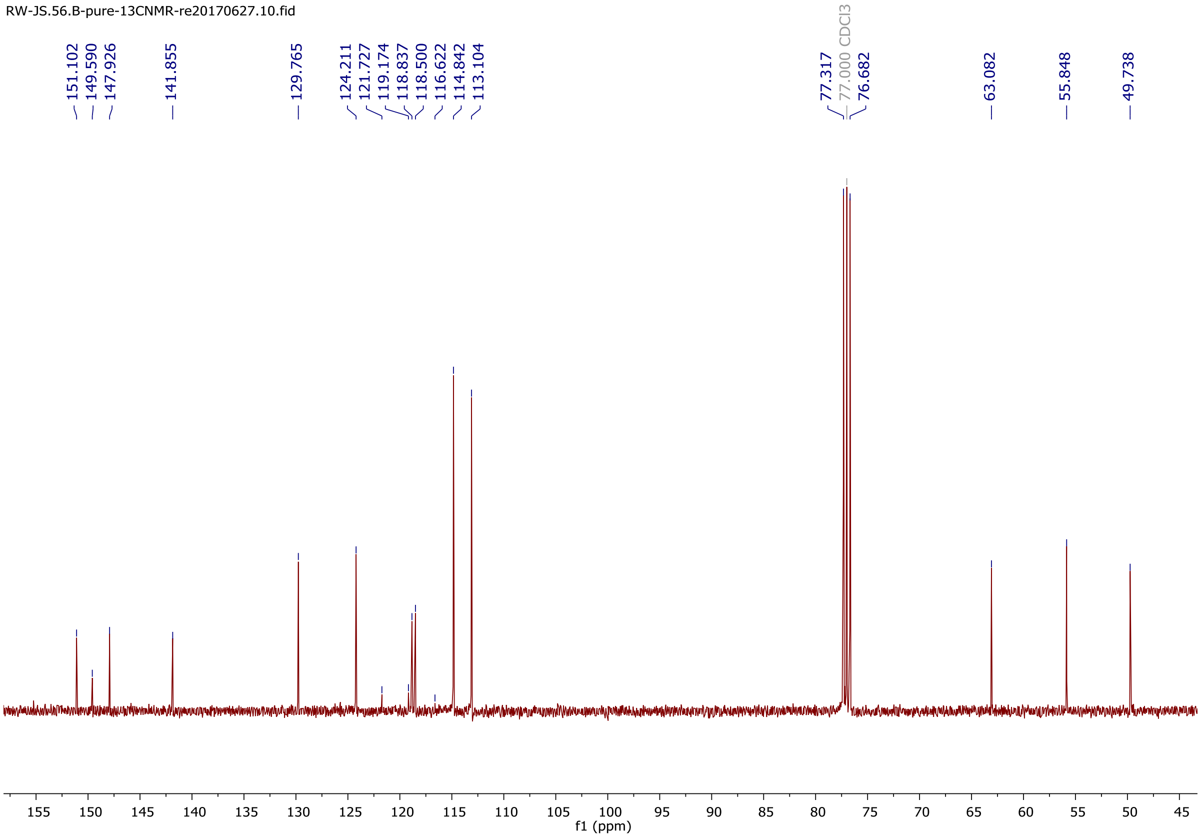

2i (^{1}H NMR and ^{13}C NMR, $CDCl_3$)

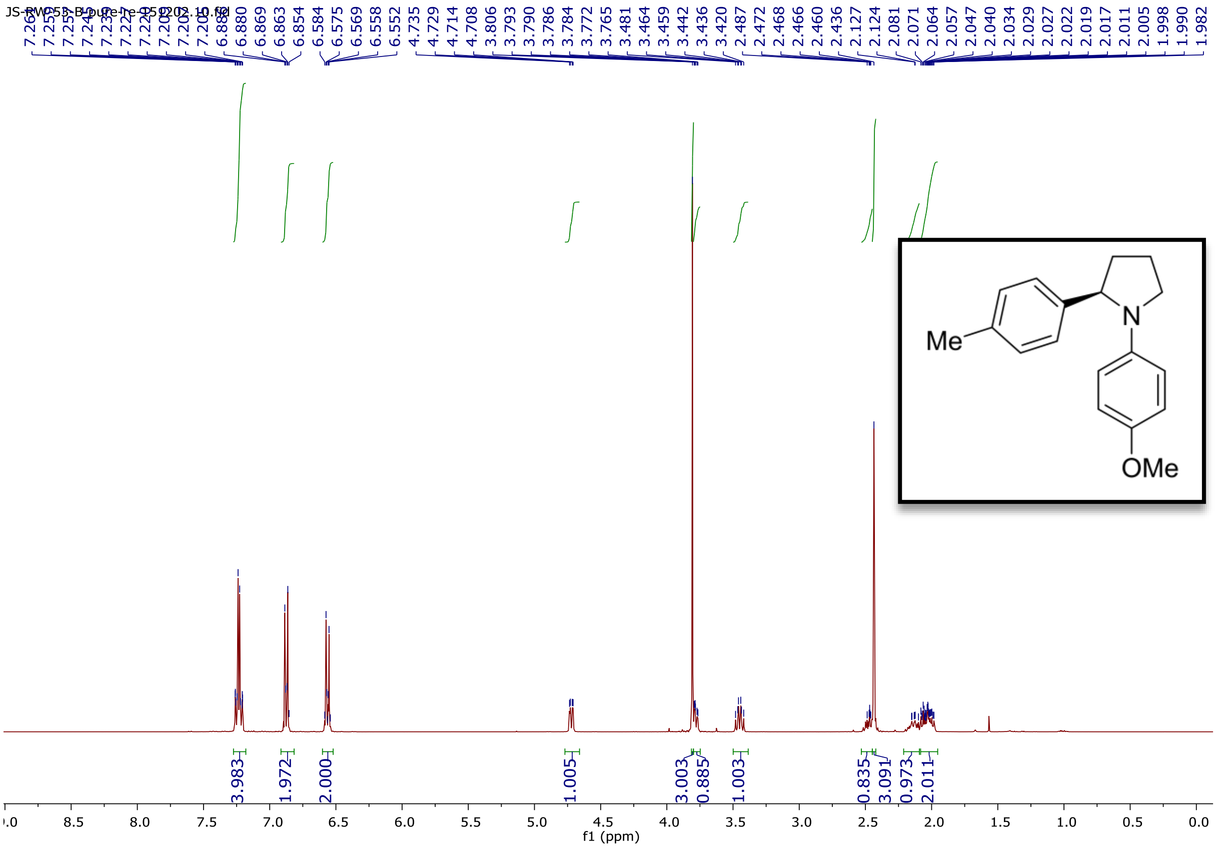
JS-RW053-B-pure-re-150202.10.fid
6.880 6.869 6.863 6.854 6.584 6.575 6.569 6.558 6.552 4.735 4.729 4.714 4.708 3.806 3.793 3.790 3.786 3.784 3.772 3.765 3.481 3.464 3.459 3.442 3.436 3.420 2.487 2.472 2.468 2.466 2.460 2.436 2.127 2.124 2.081 2.071 2.064 2.057 2.047 2.040 2.034 2.029 2.027 2.022 2.019 2.017 2.011 2.005 1.998 1.990 1.982
Me
N
OMe
3.983 1.972 2.000 1.005 3.003 0.885 1.003 0.835 3.091 0.973 2.011
8.5 8.0 7.5 7.0 6.5 6.0 5.5 5.0 4.5 4.0 3.5 3.0 2.5 2.0 1.5 1.0 0.5 0.0
f1 (ppm)

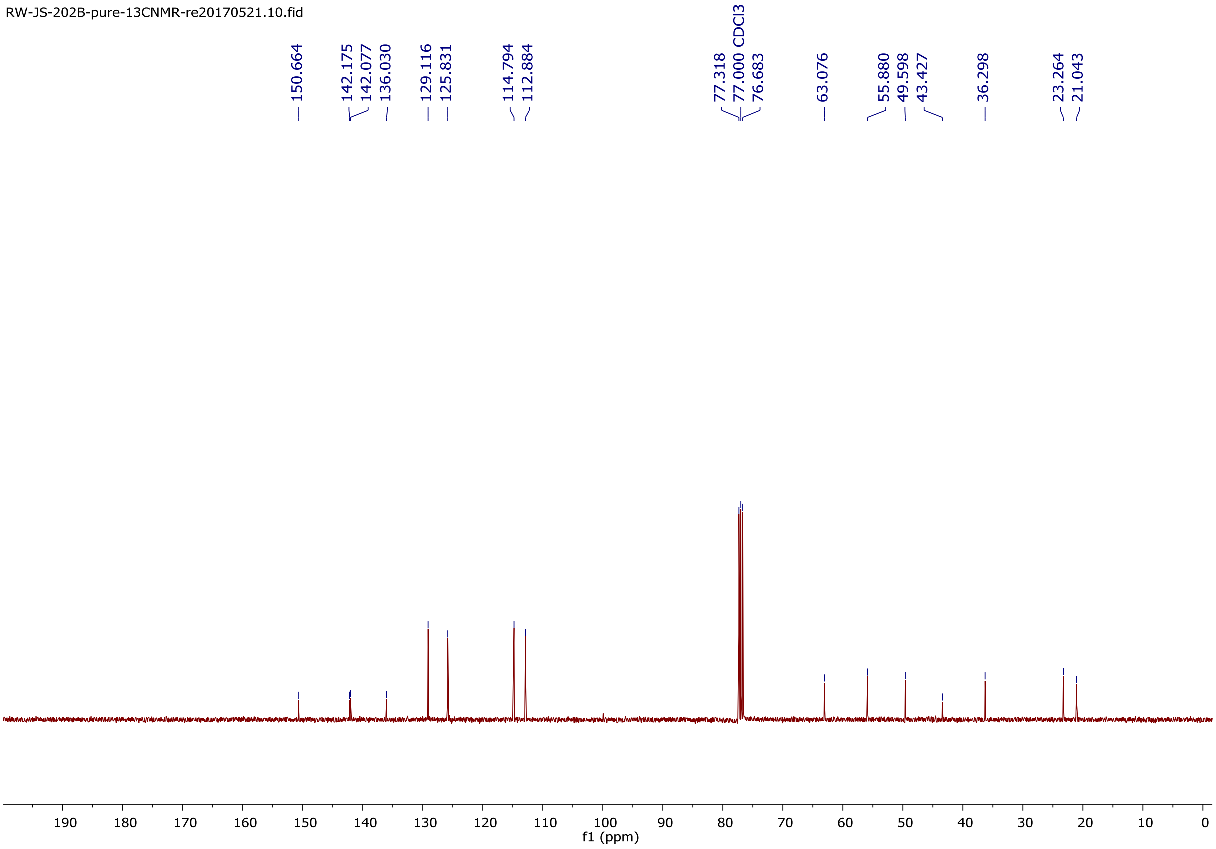
RW-JS-202B-pure-13CNMR-re20170521.10.fid
150.664 142.175 142.077 136.030 129.116 125.831 114.794 112.884 77.318 77.000 CDCl3 76.683 63.076 55.880 49.598 43.427 36.298 23.264 21.043
190 180 170 160 150 140 130 120 110 100 90 80 70 60 50 40 30 20 10 0
f1 (ppm)

2j (^{1}H NMR and ^{13}C NMR, $CDCl_3$)

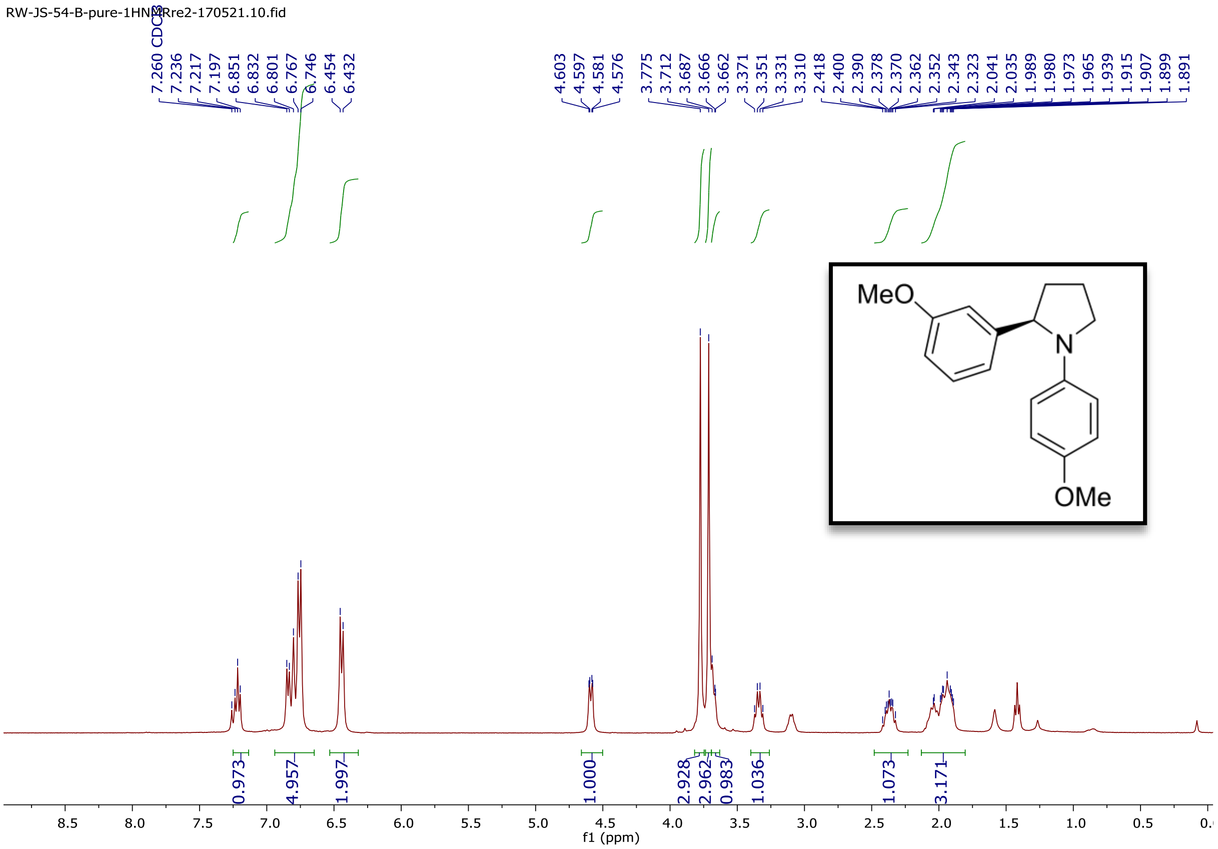

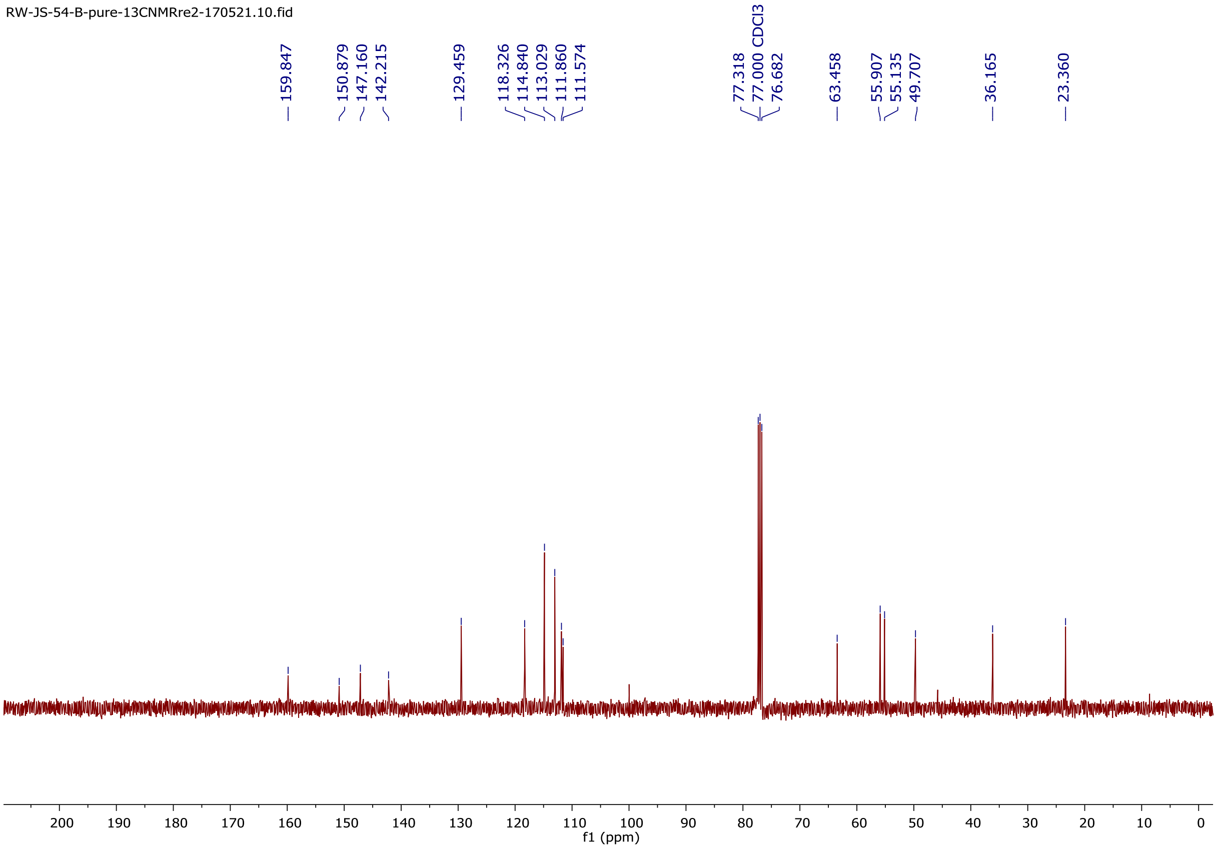

2k (^{1}H NMR and ^{13}C NMR, $CDCl_3$)

AB5-120-P-AC1-170624.10.fid

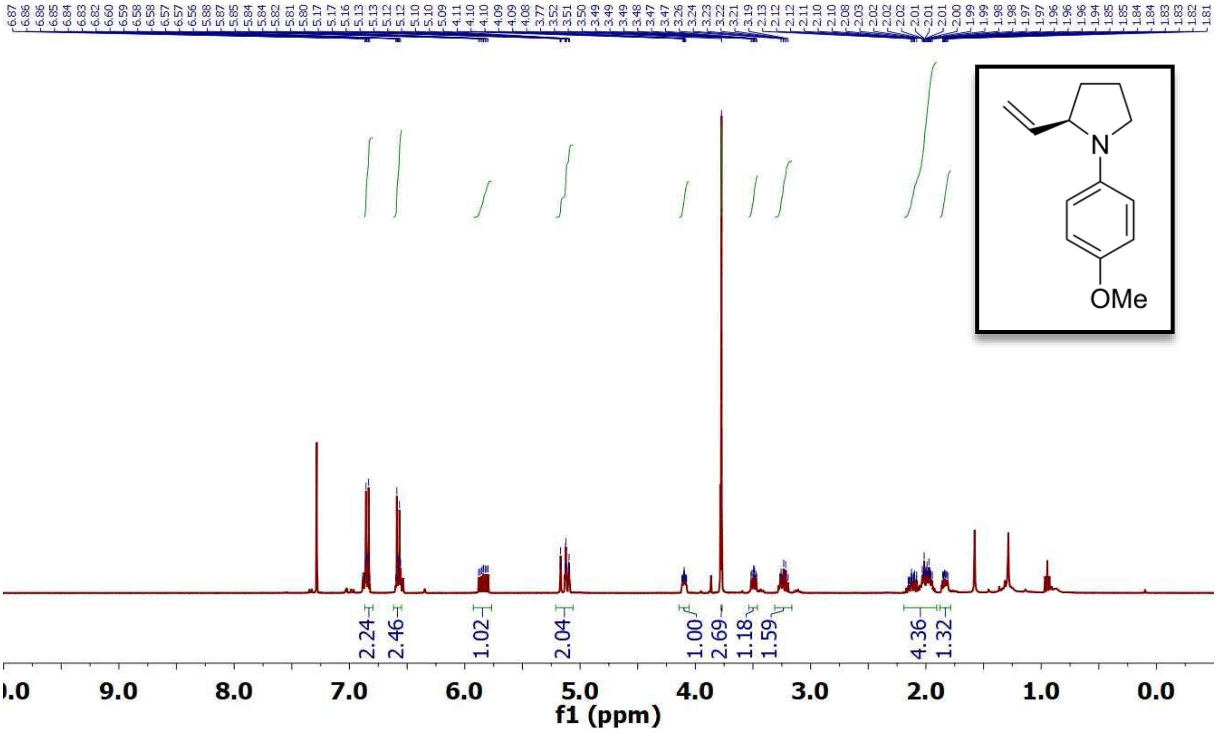

AB5-120-P-AC1-170624.11.fid

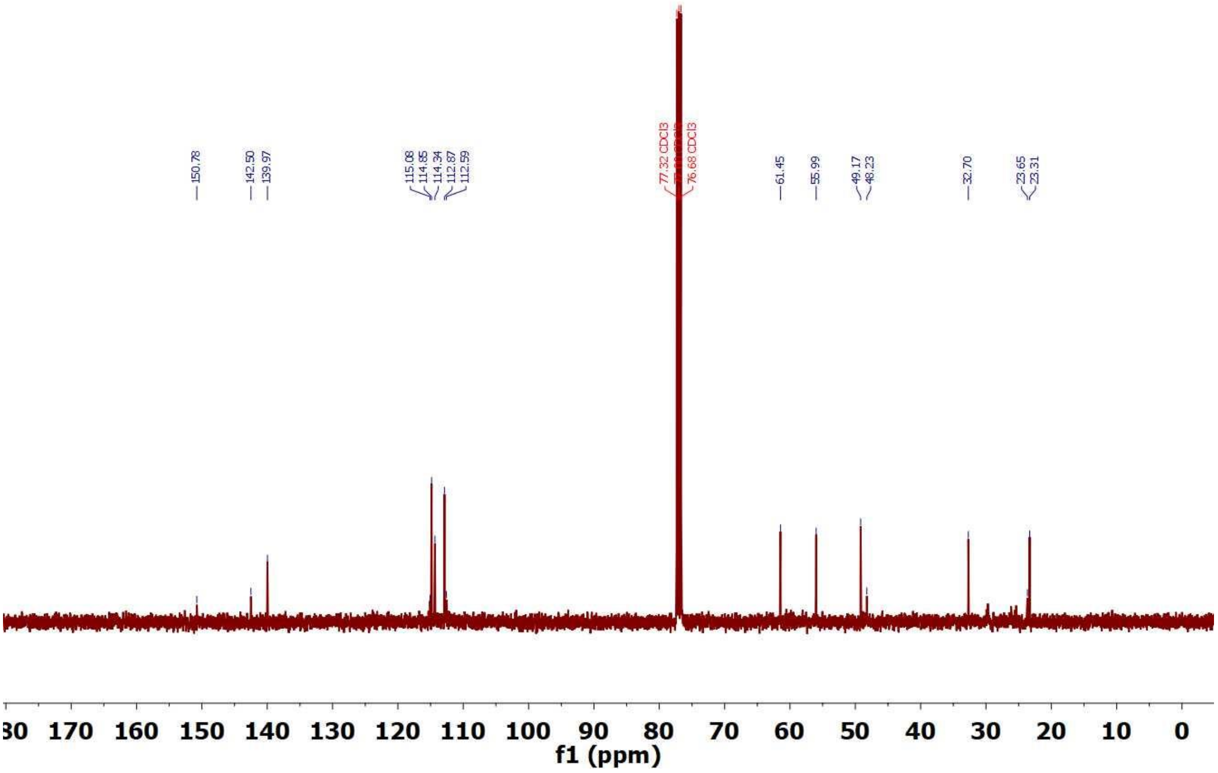

2l (^{1}H NMR and ^{13}C NMR, $CDCl_3$)

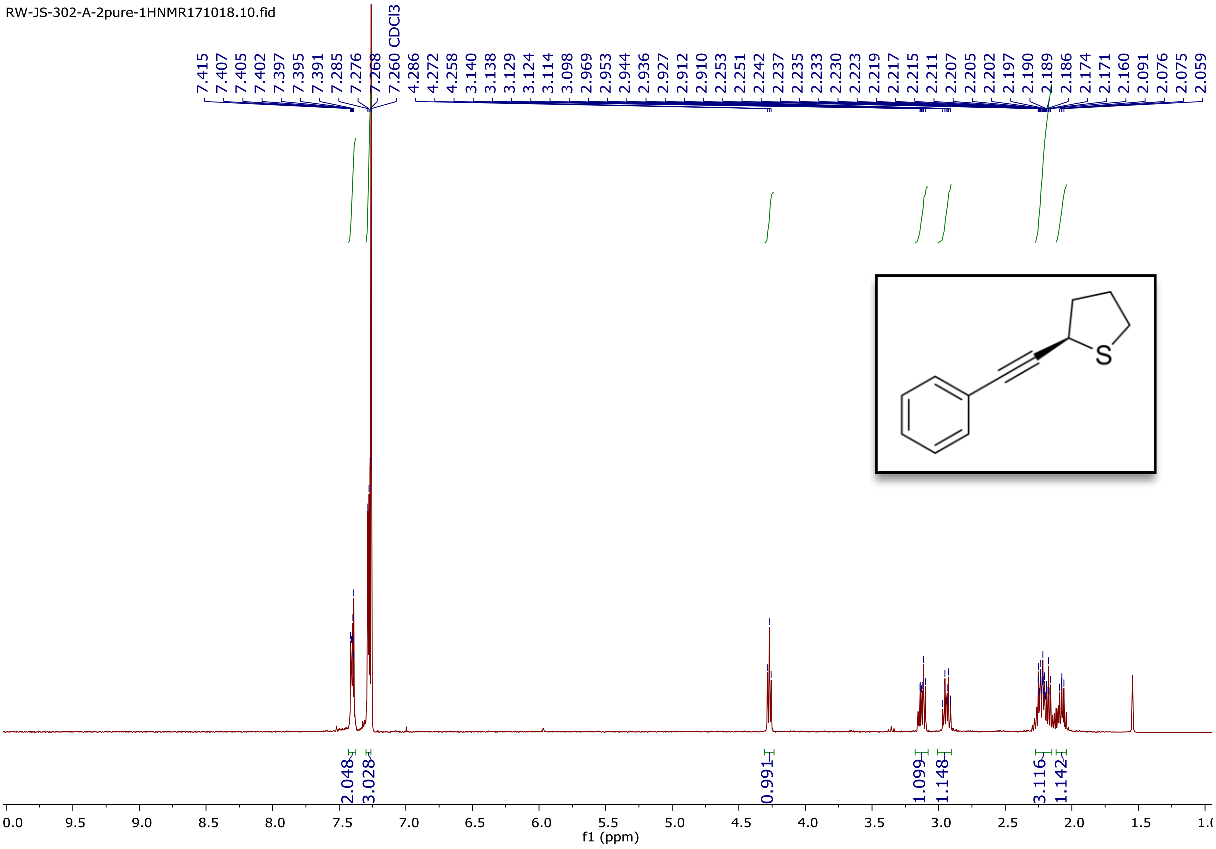

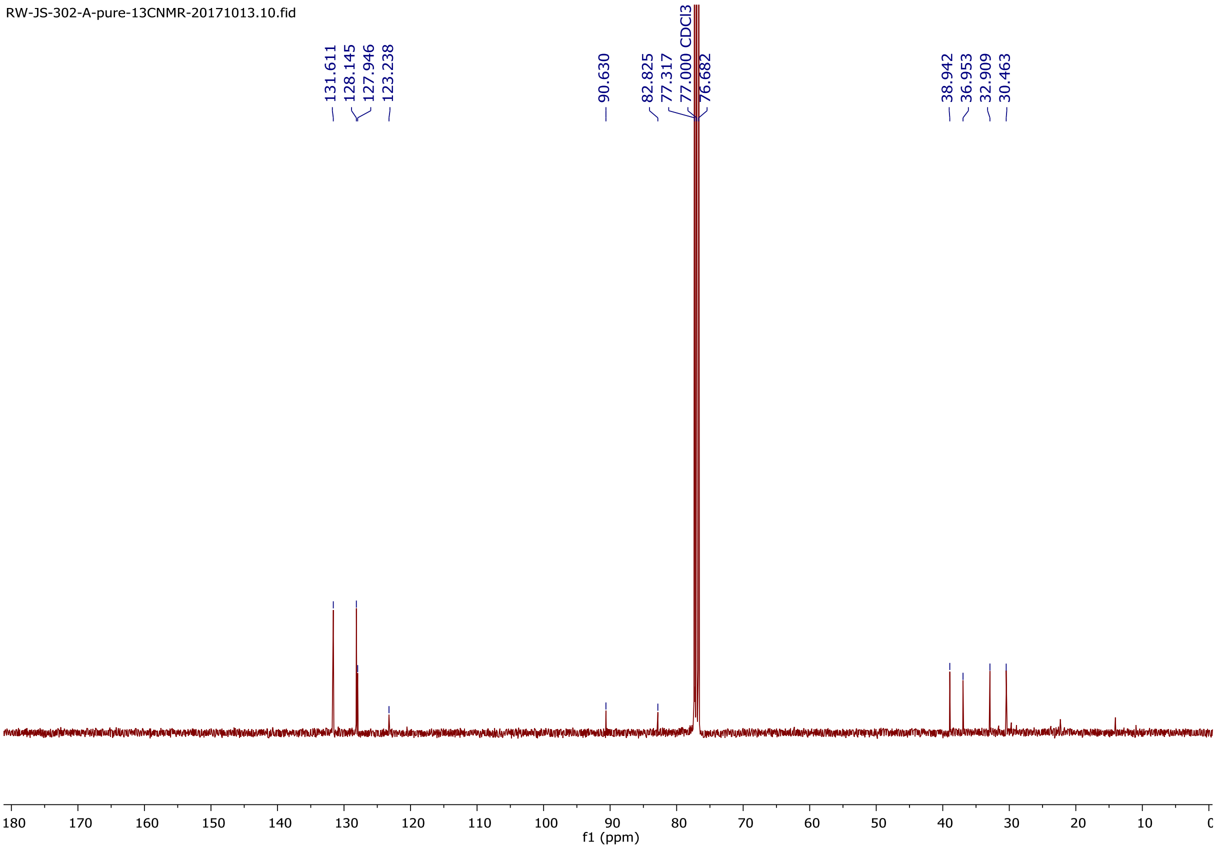

2m (^{1}H NMR and ^{13}C NMR, $CDCl_3$)

AB5-123-AC1-160803.20.fid

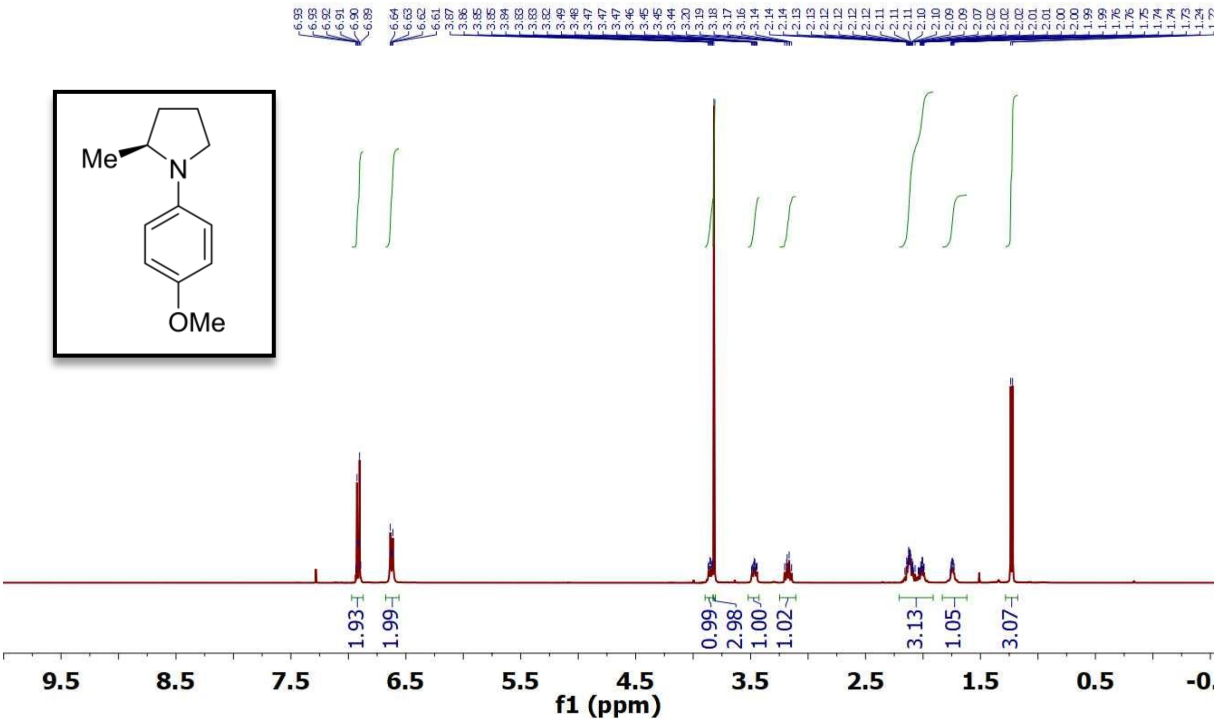

AB5-123-AC1-160803.21.fid

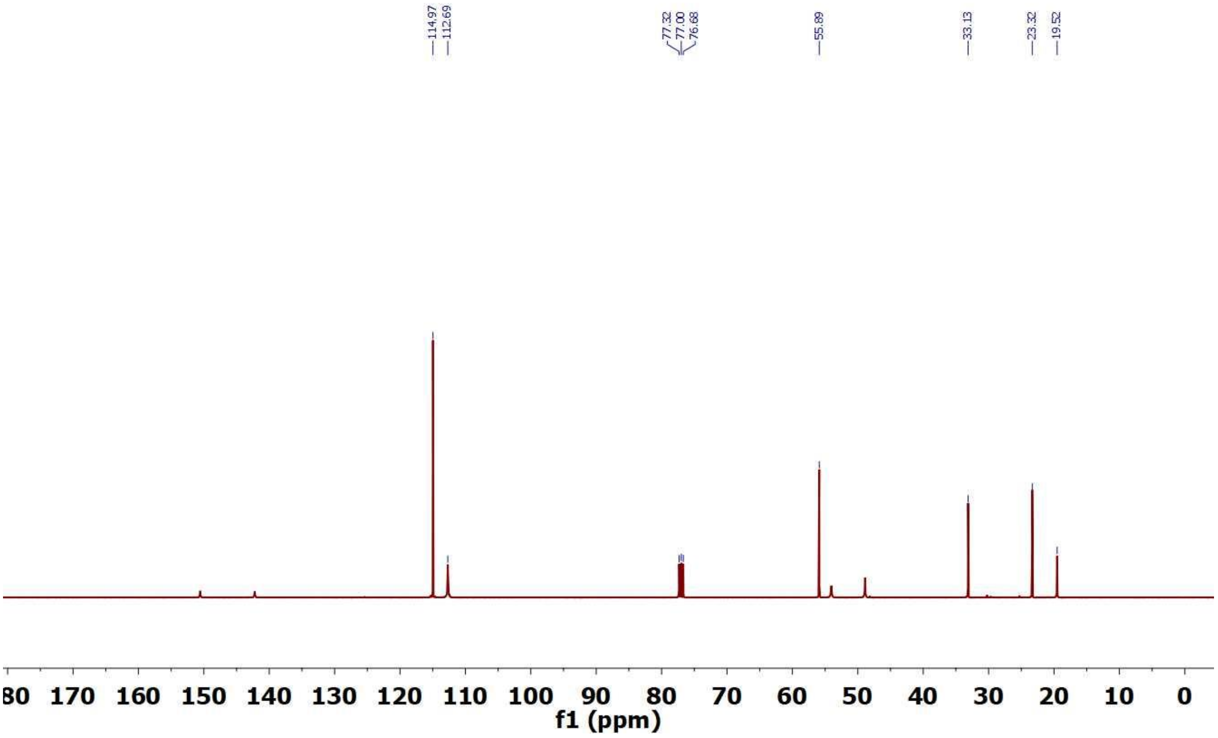

2n (^{1}H NMR and ^{13}C NMR, $CDCl_3$)

AB5-22-AC3-170622.10.fid

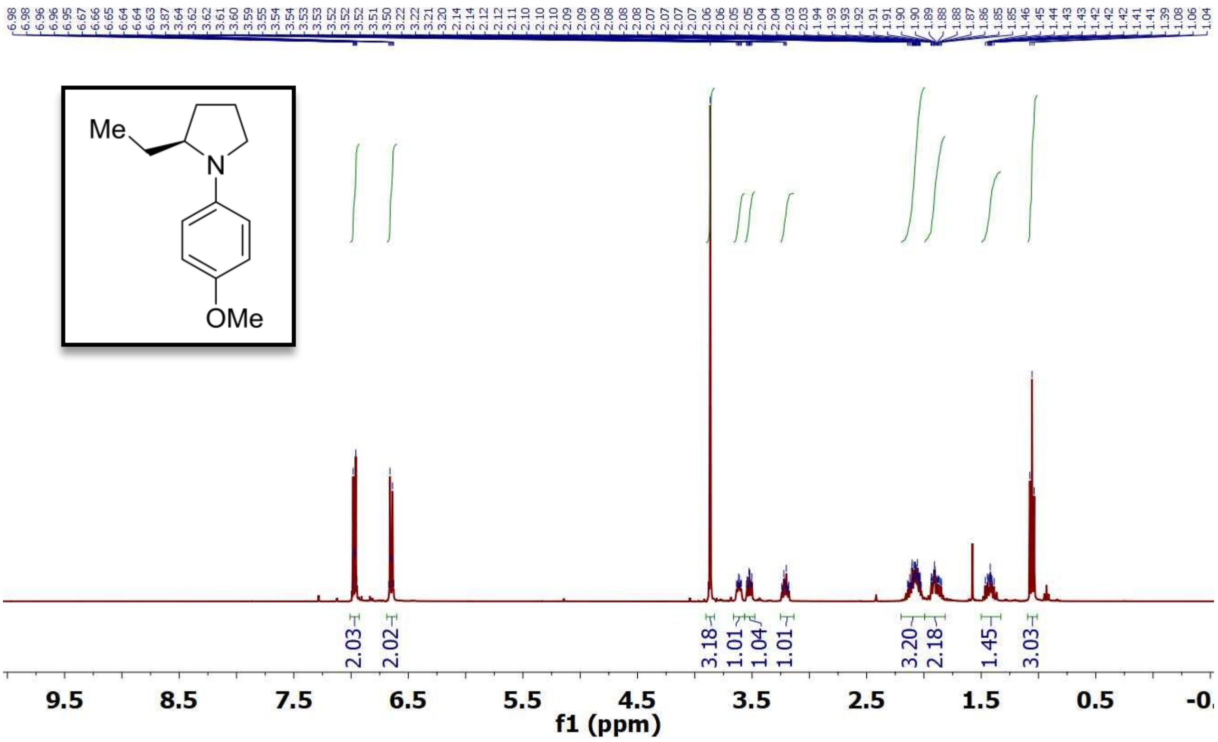

AB5-22-AC3-170622.11.fid

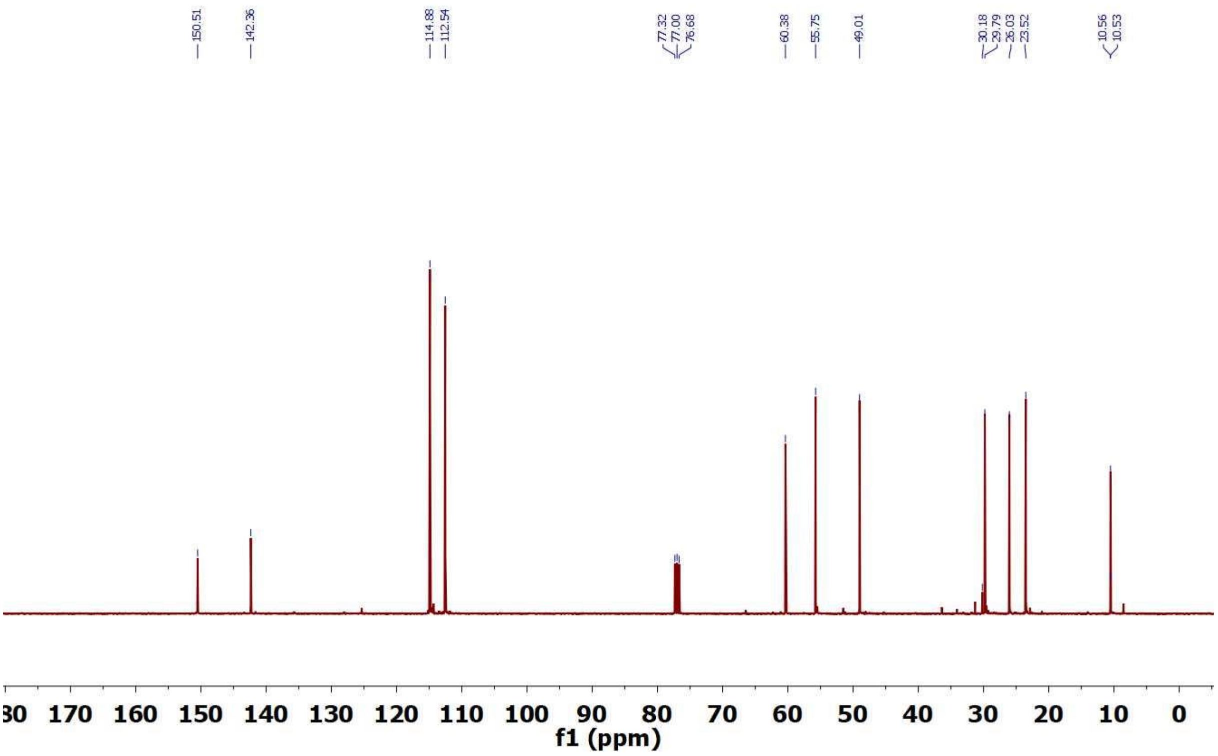

2o (^{1}H NMR and ^{13}C NMR, $CDCl_3$)

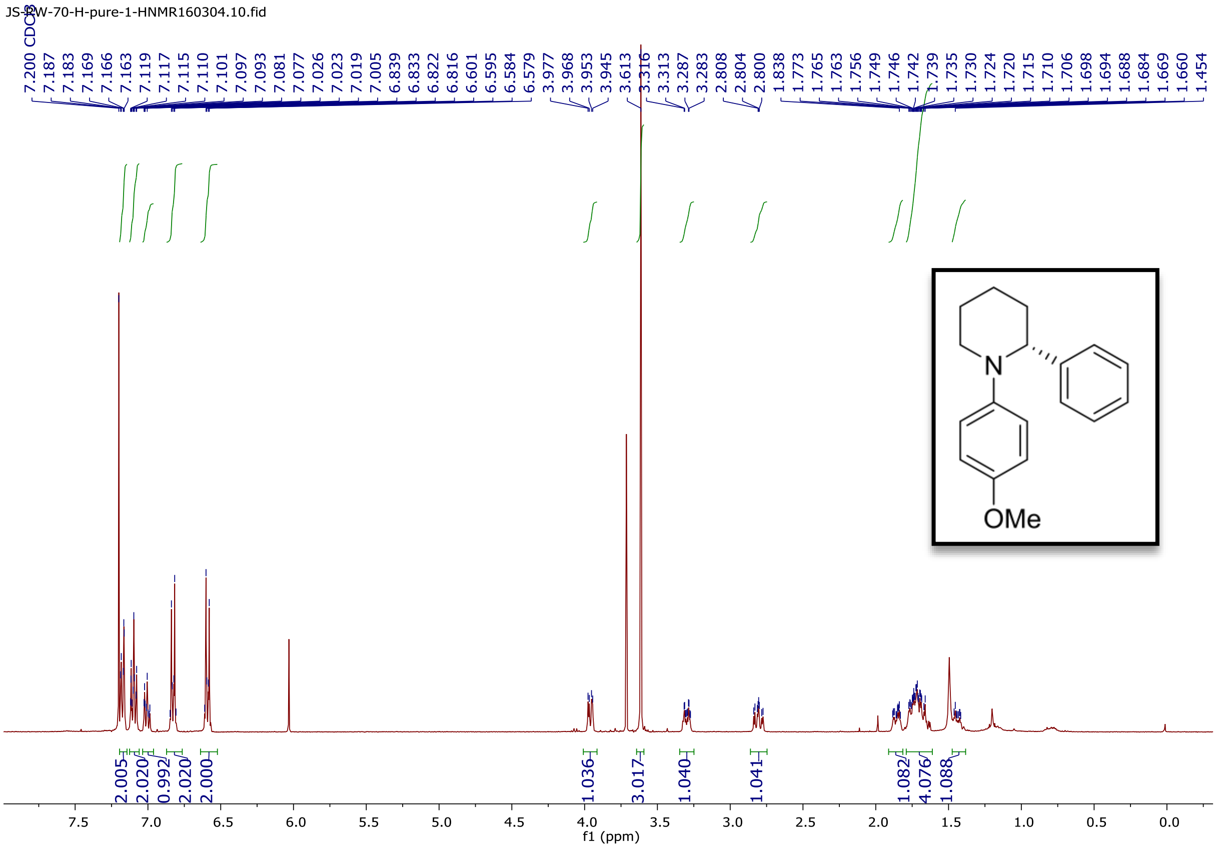

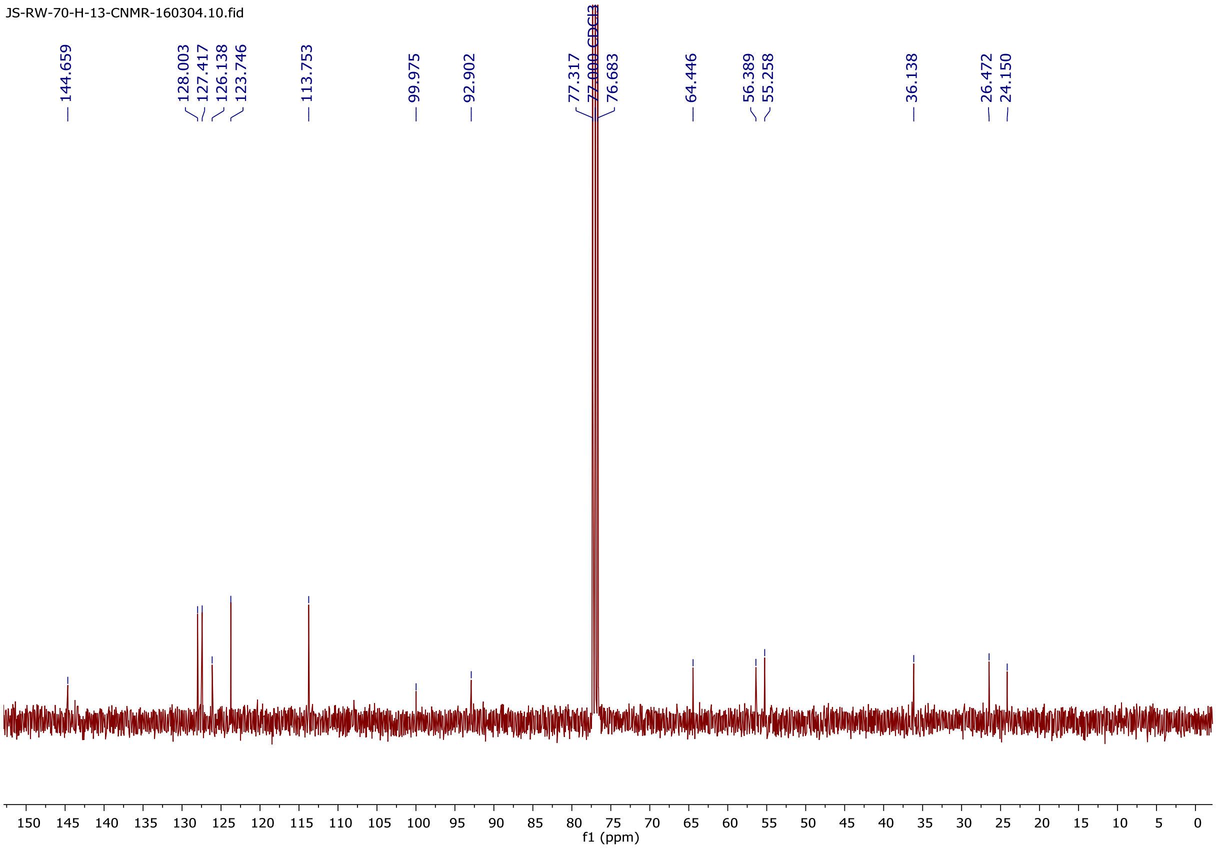

2p (^{1}H NMR and ^{13}C NMR, $CDCl_3$)

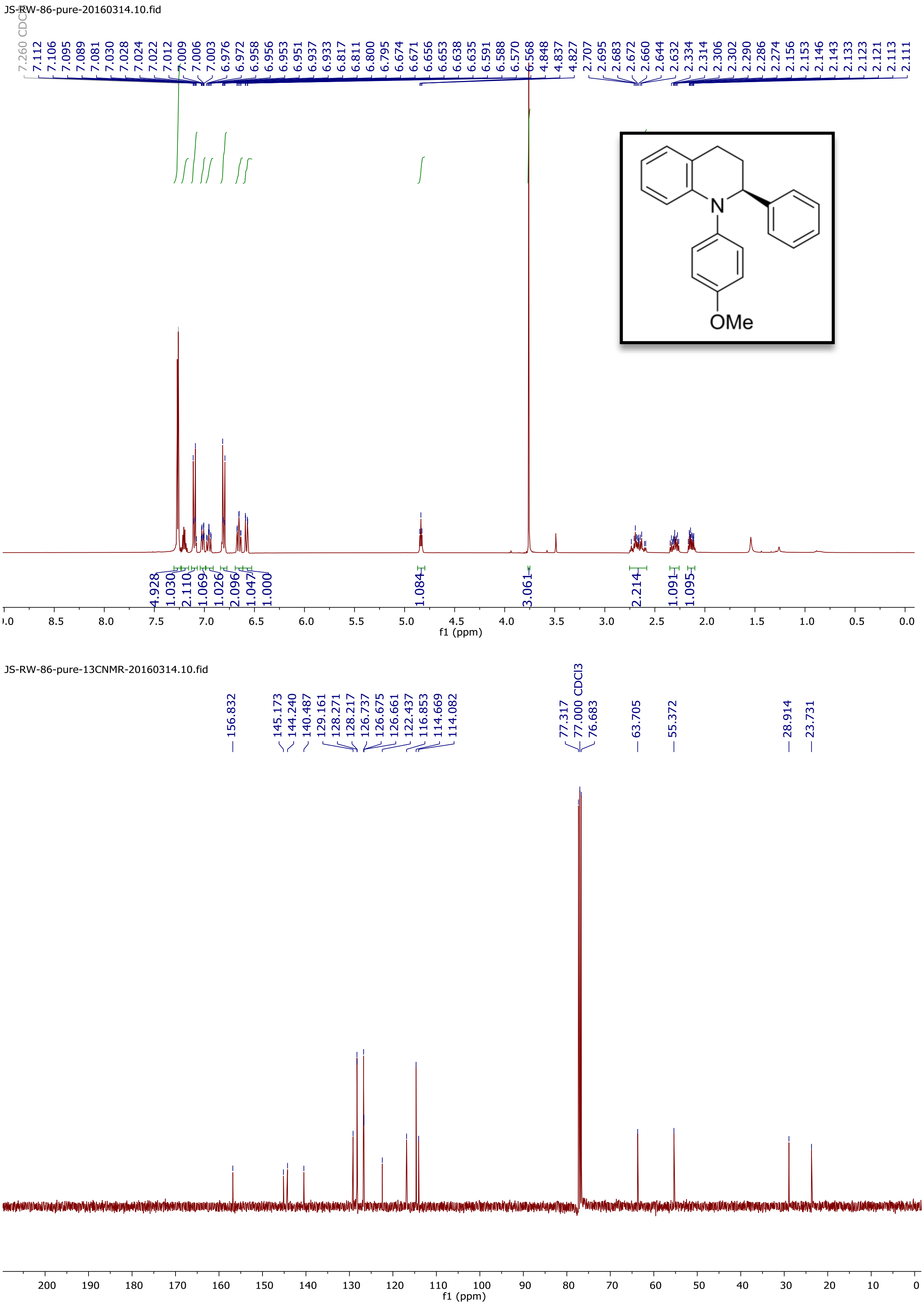

2q (^{1}H NMR and ^{13}C NMR, $CDCl_3$)

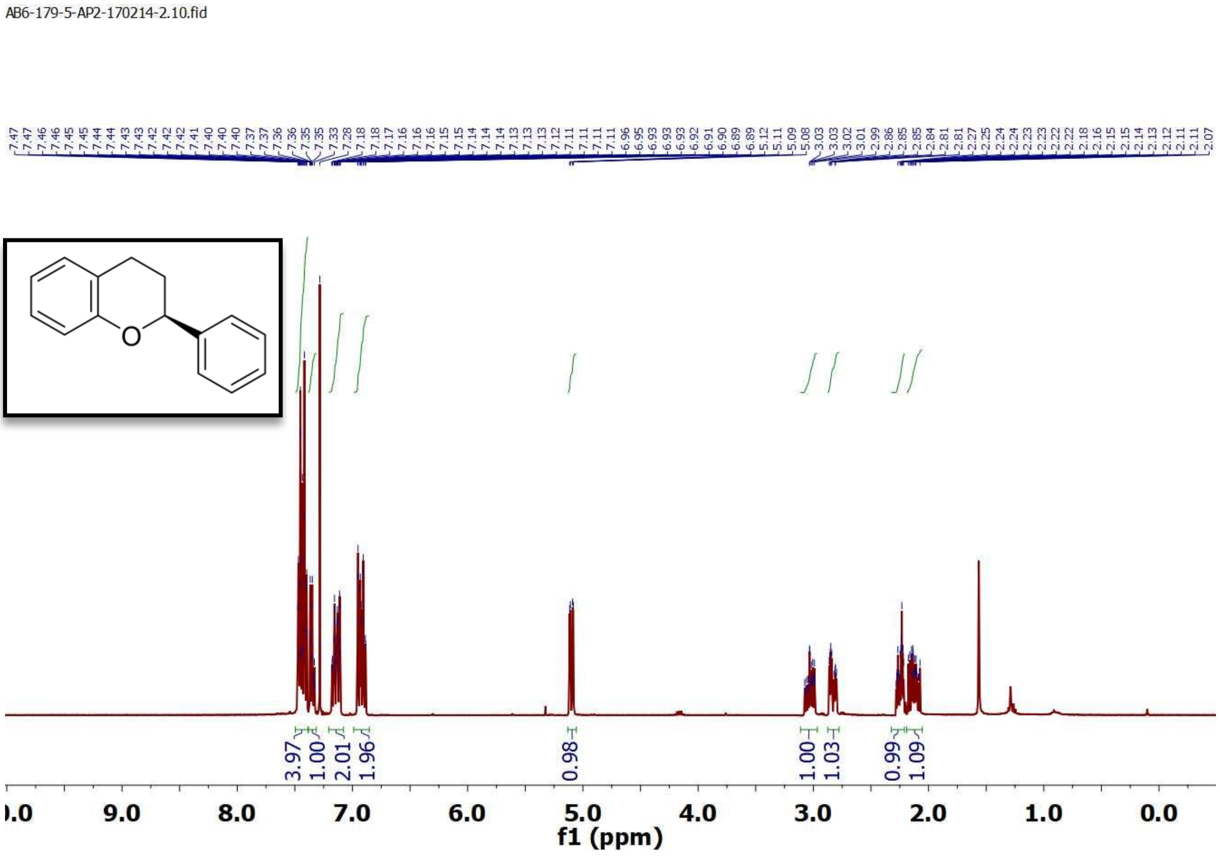

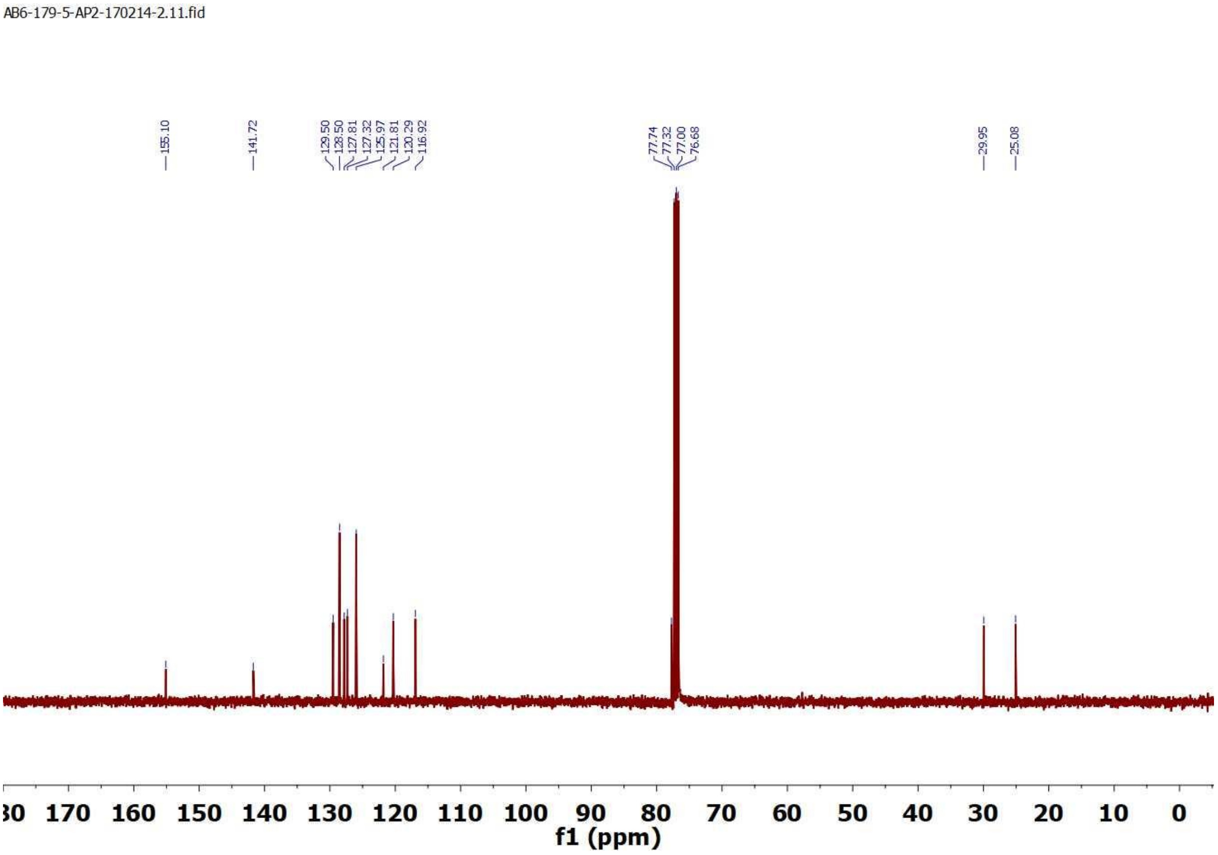

2r (^{1}H NMR and ^{13}C NMR, $CDCl_3$)

AB7-25-AF-170622.10.fid

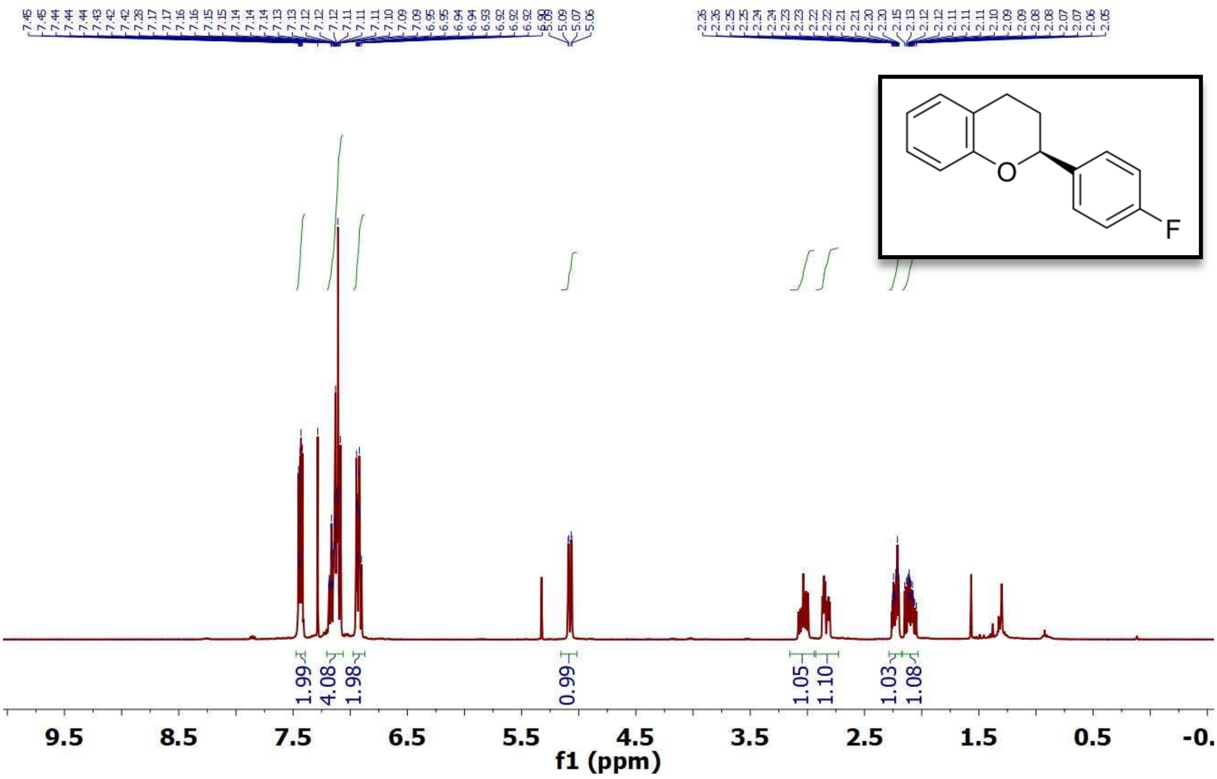

AB7-25-AF-170622.11.fid

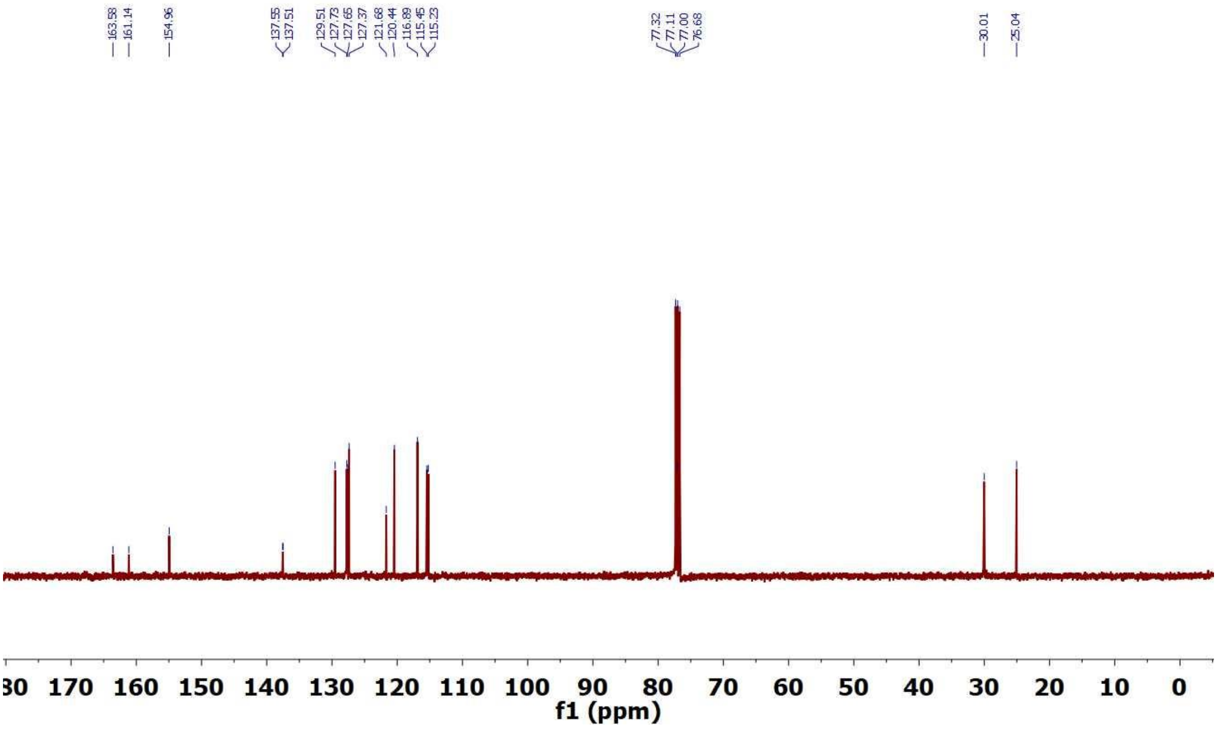

2d´ (^1H NMR and ^{13}C NMR, $CDCl_3$)

AB7-61-AC-170530.10.fid

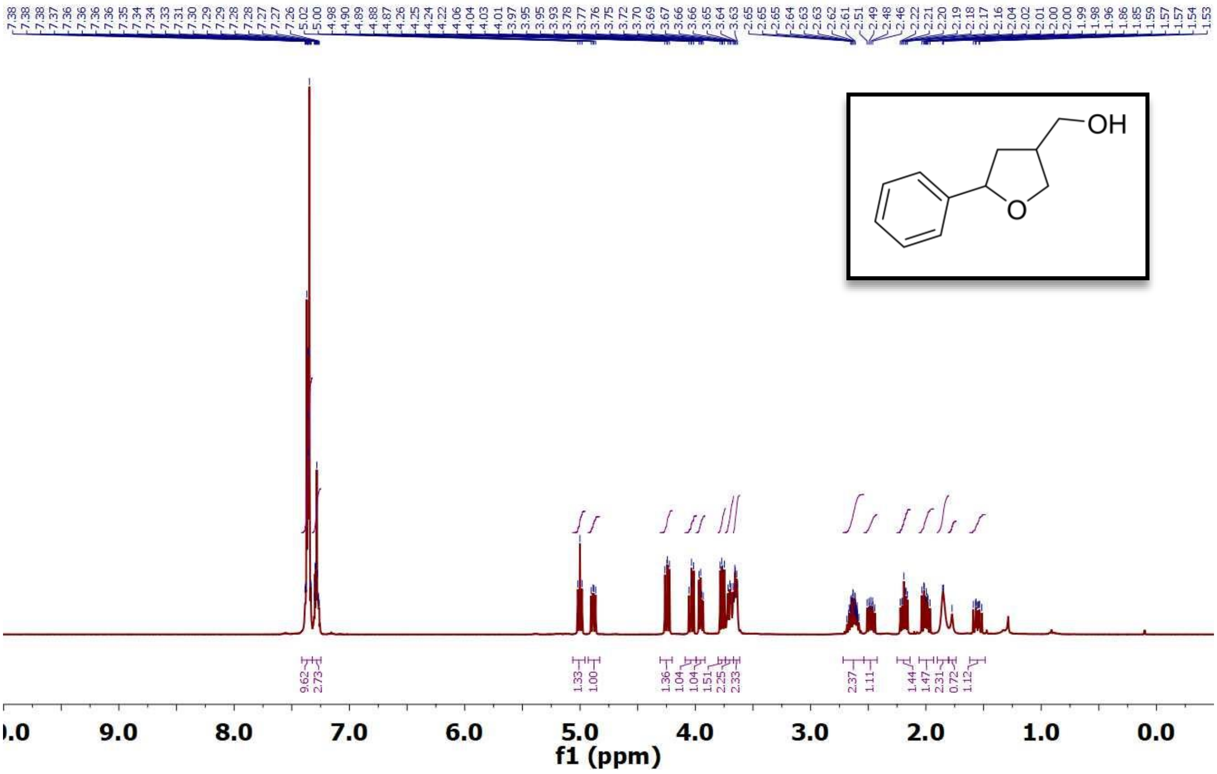

AB7-61-AC-170530.11.fid

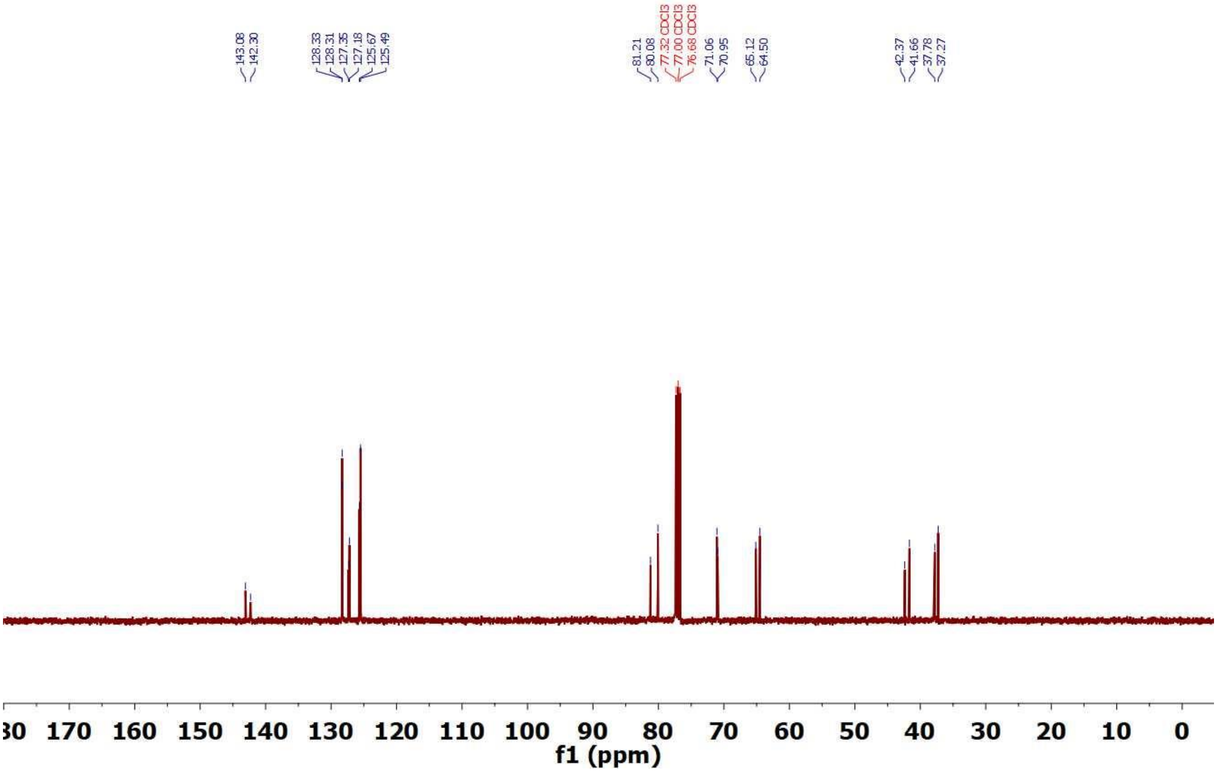

2d´´ (^{1}H NMR and ^{13}C NMR, $CDCl_3$)

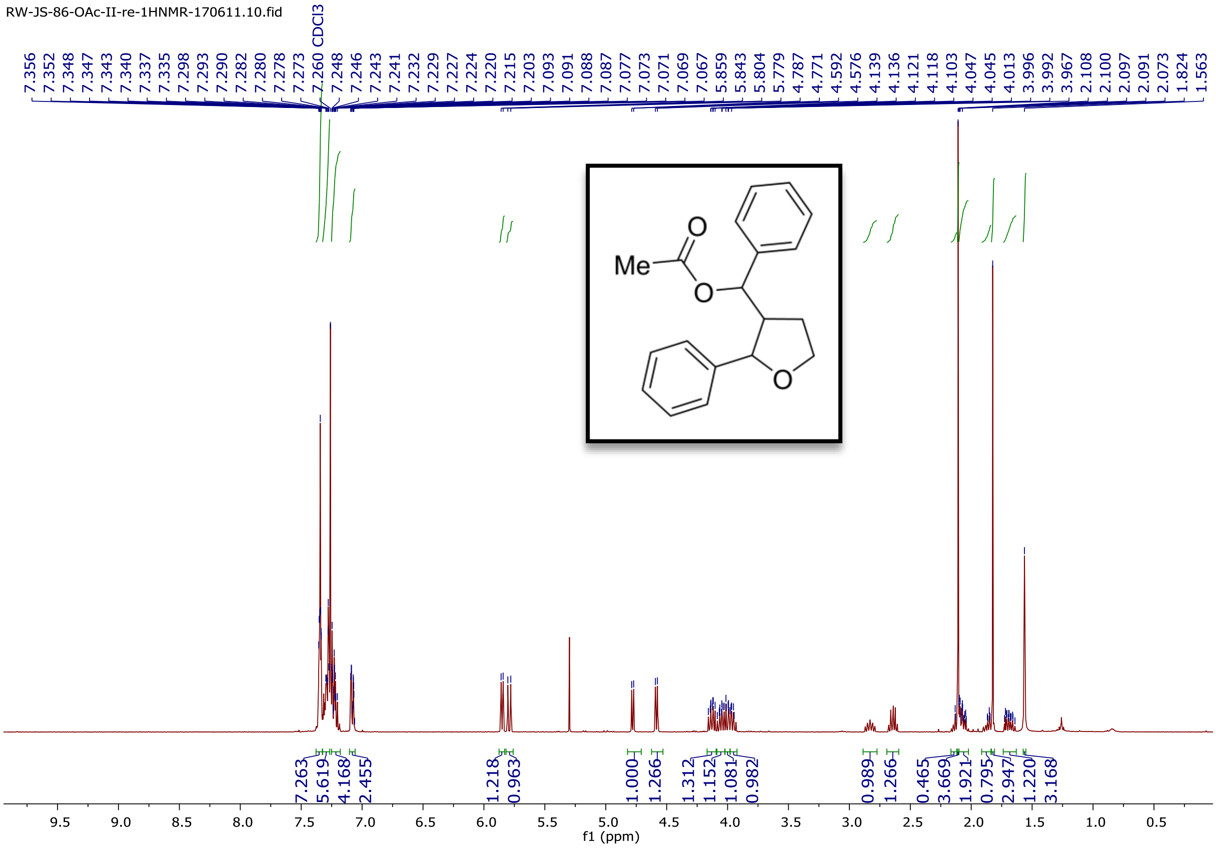

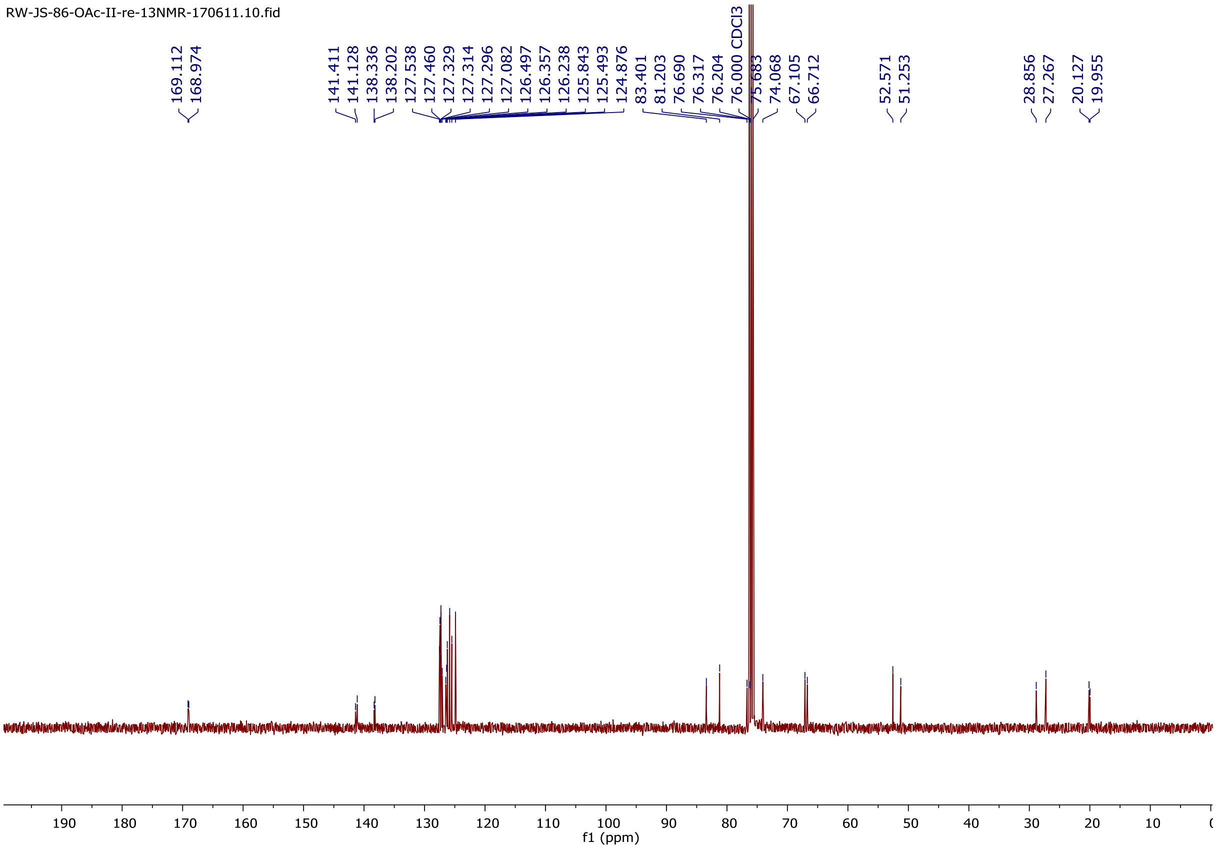

2s (^{1}H NMR and ^{13}C NMR, $CDCl_3$)

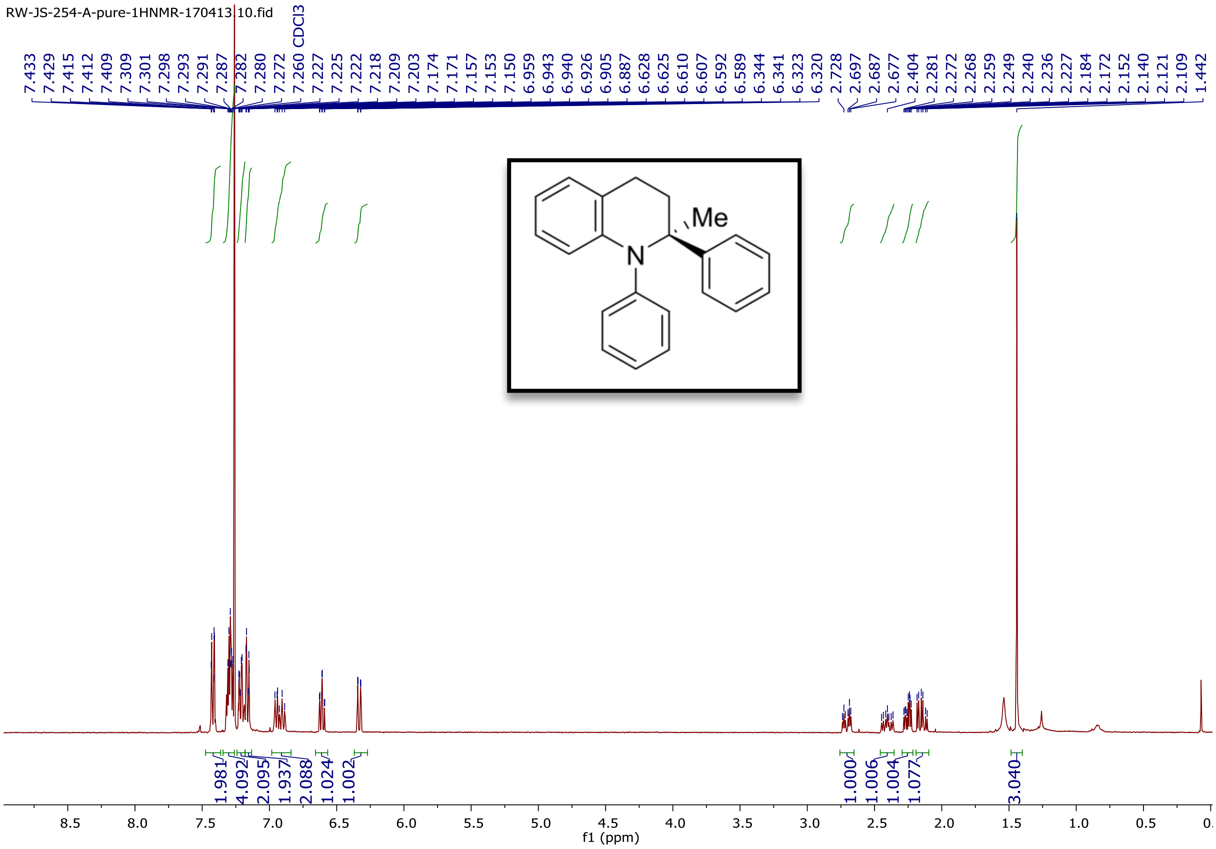

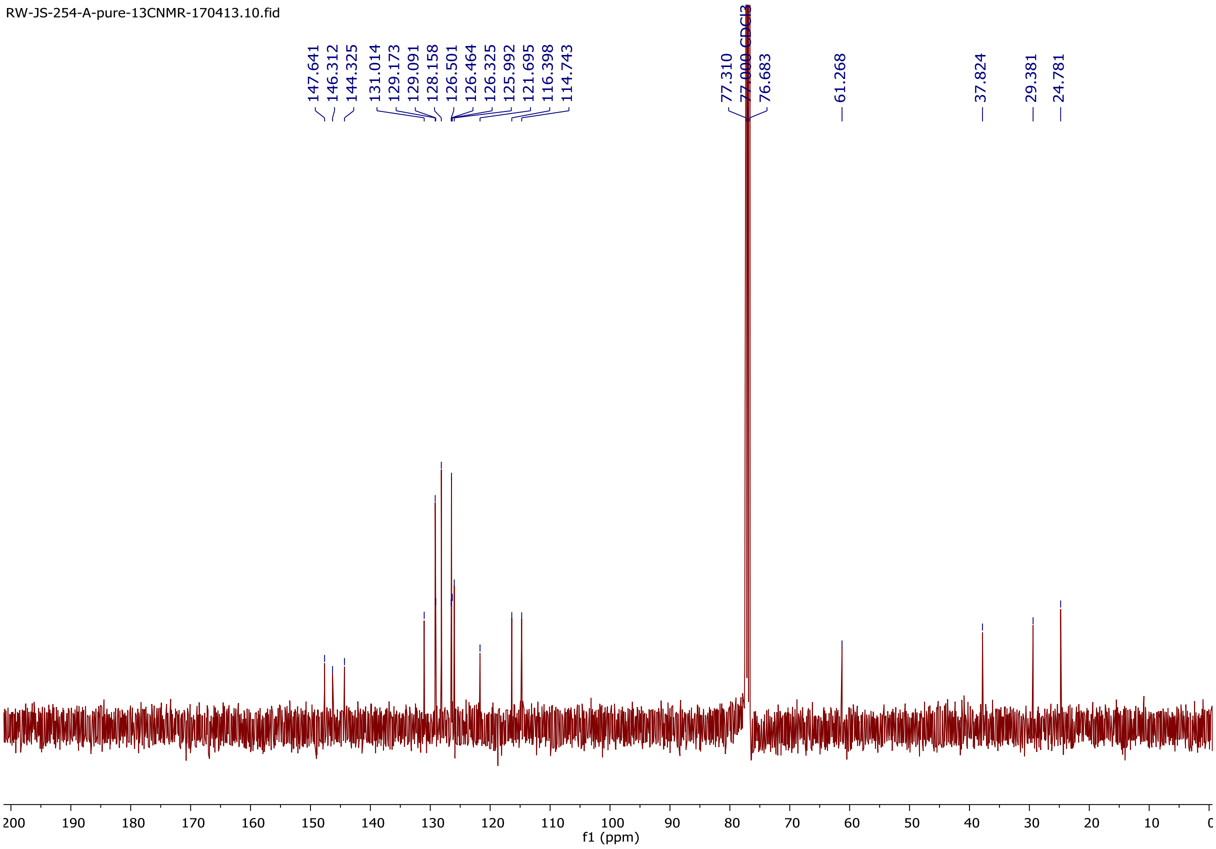

2t (^{1}H NMR and ^{13}C NMR, $CDCl_3$)

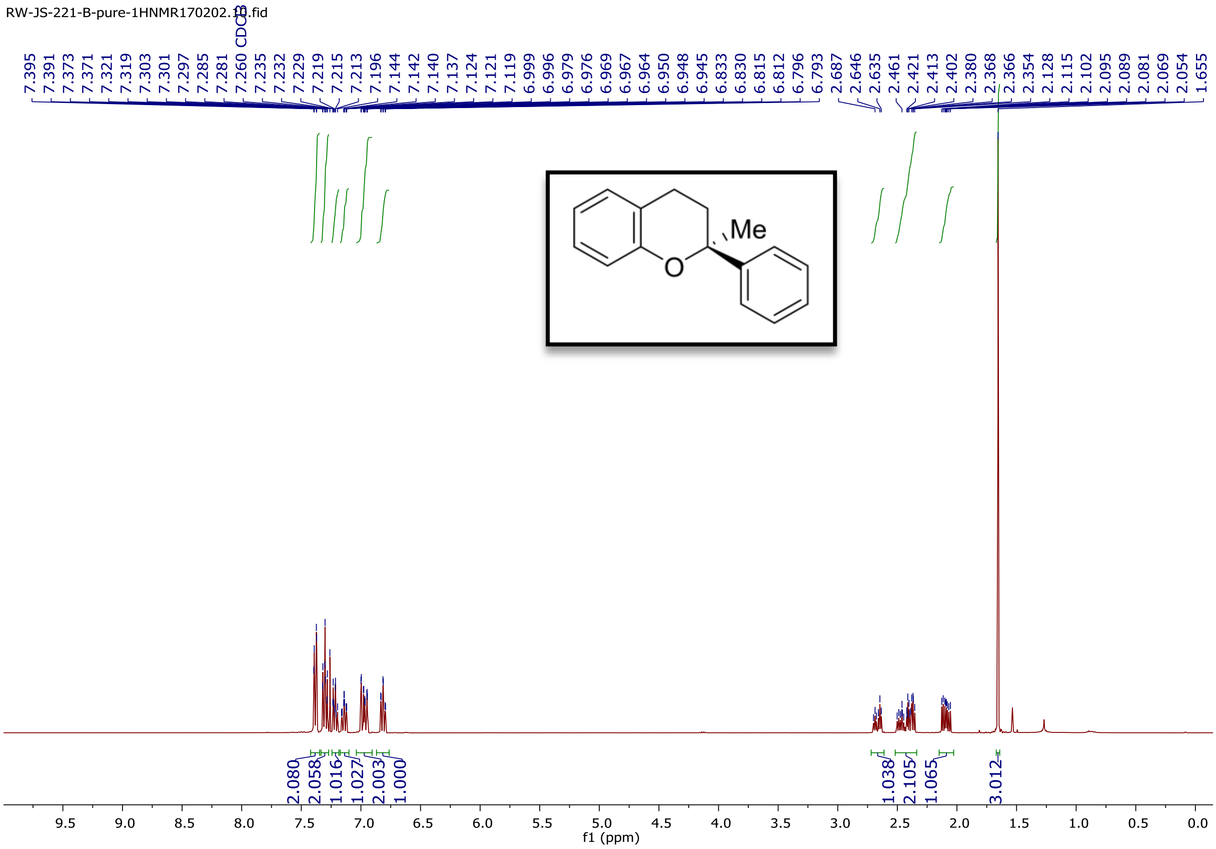

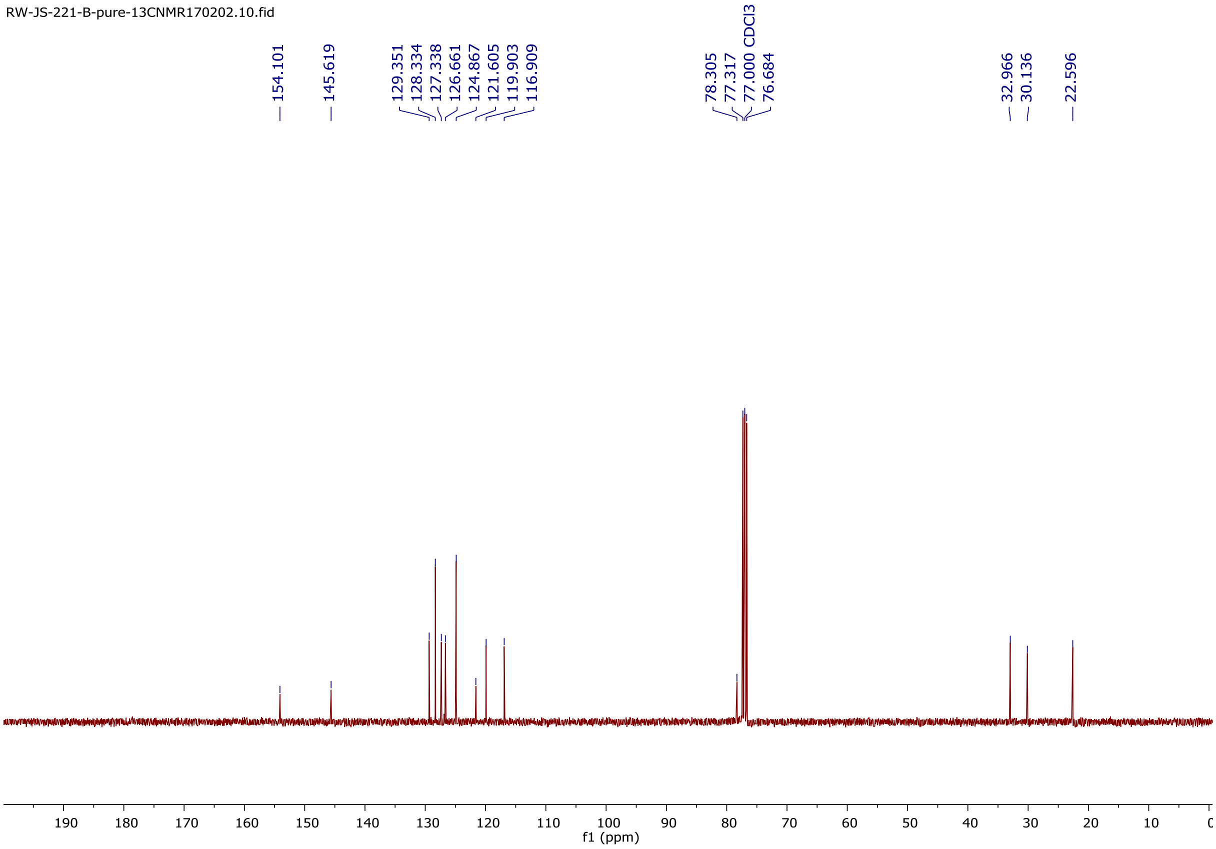

2u (^{1}H NMR and ^{13}C NMR, $CDCl_3$)

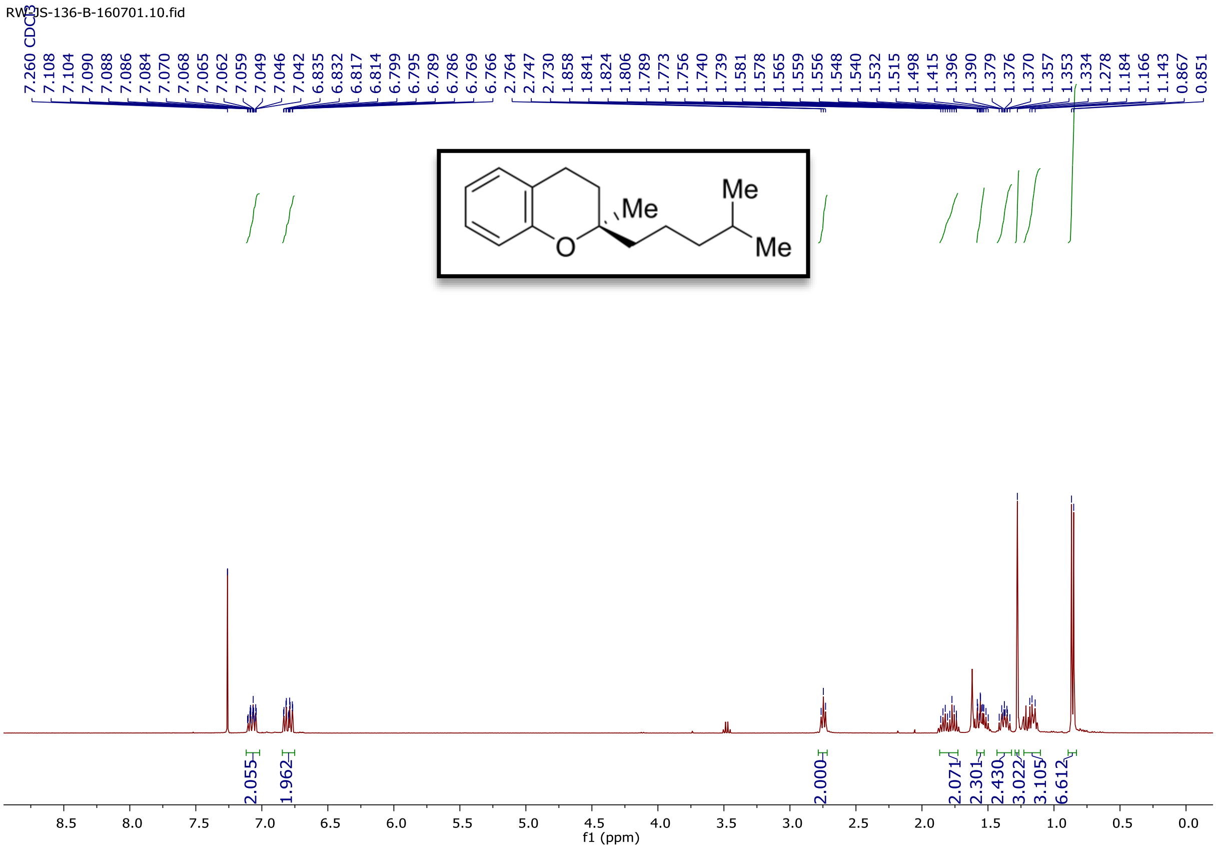

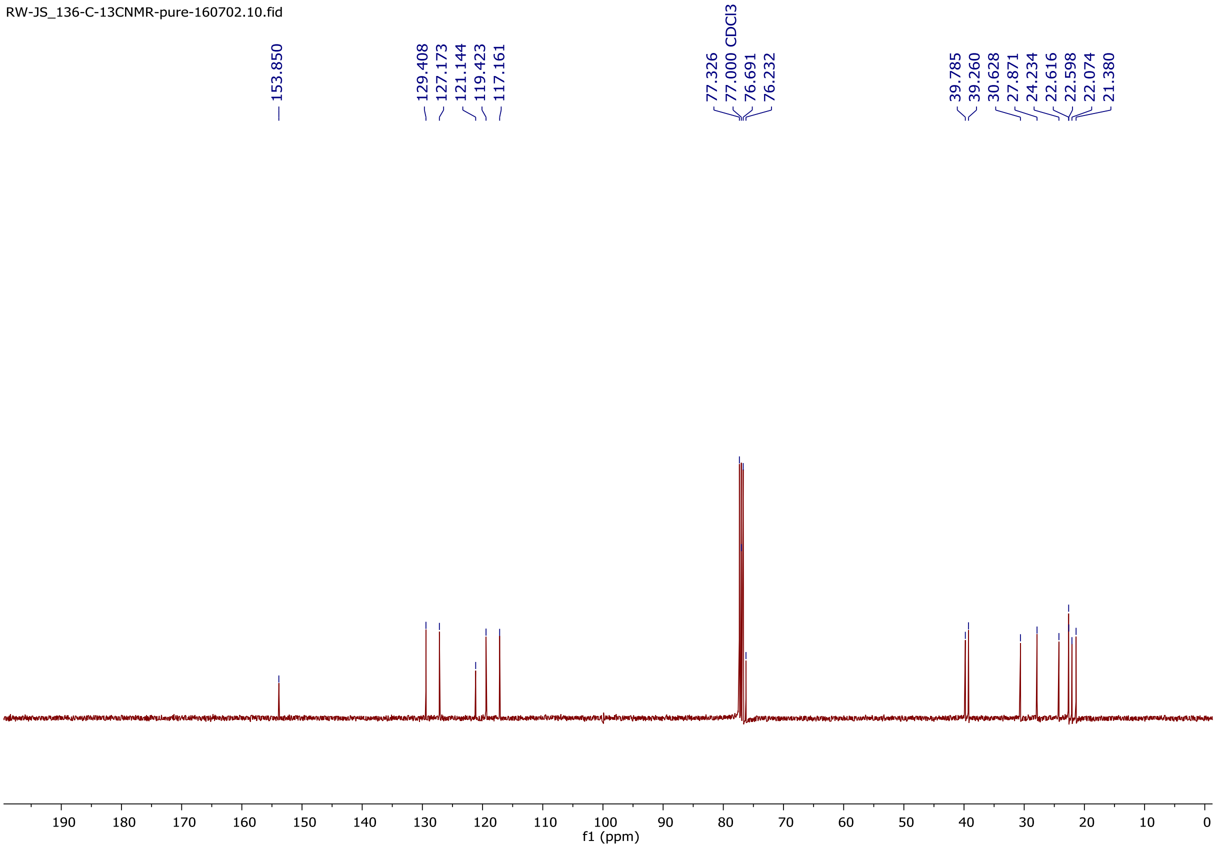

2v (^{1}H NMR and ^{13}C NMR, $CDCl_3$)

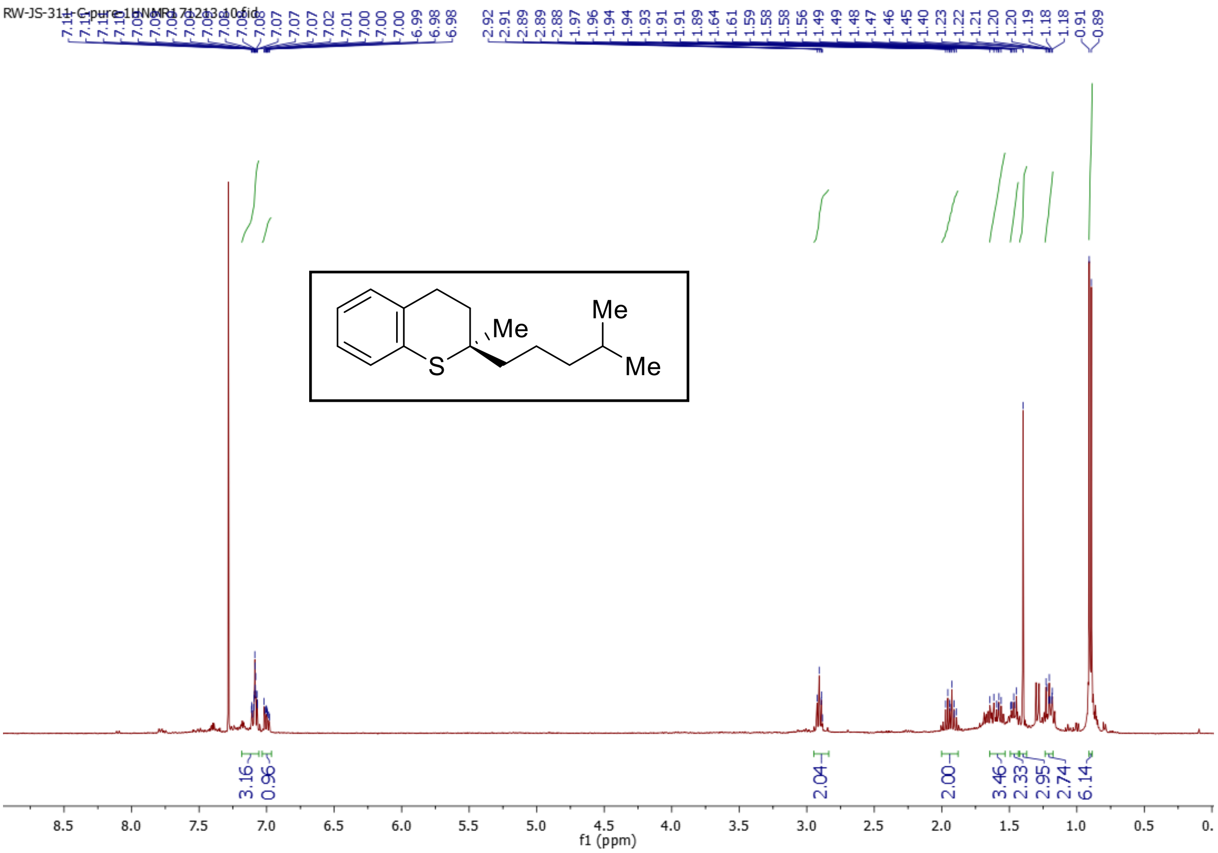

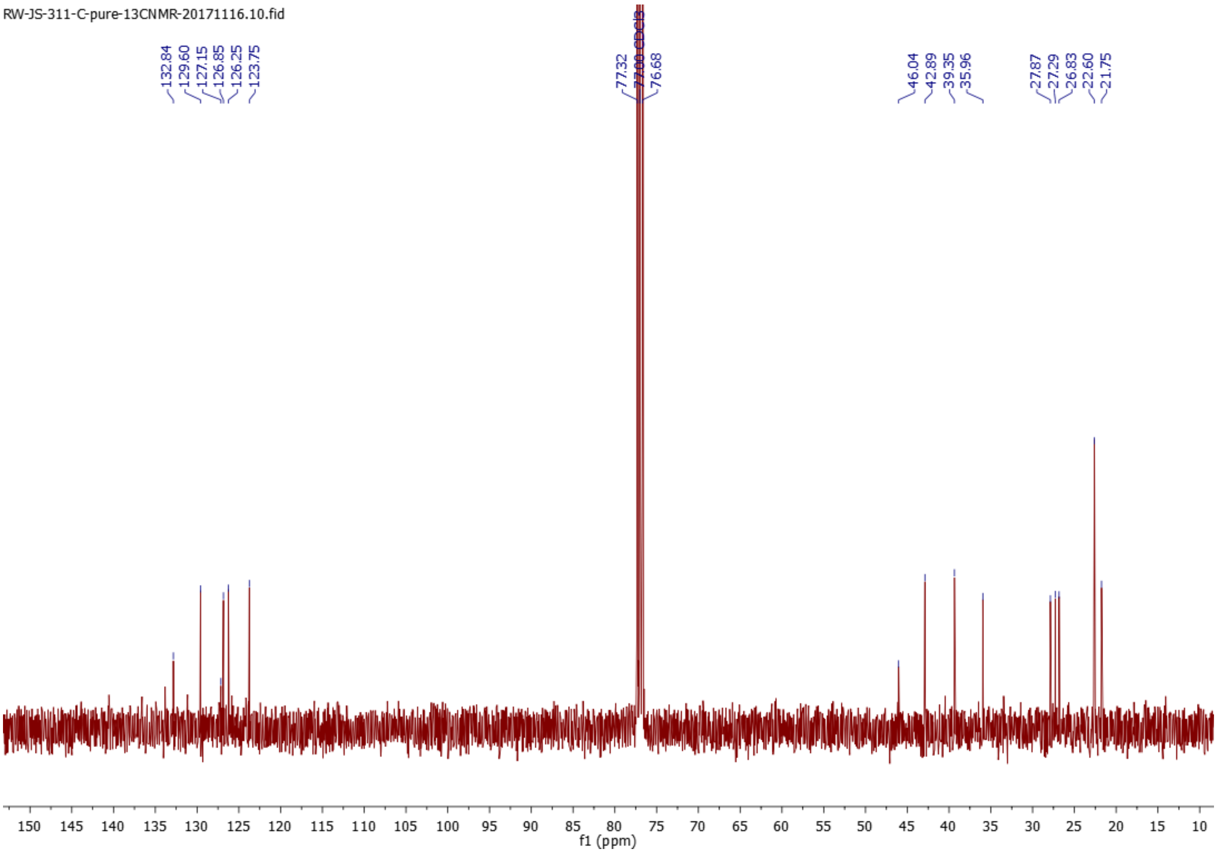

2u′ (^{1}H NMR and ^{13}C NMR, $CDCl_3$)

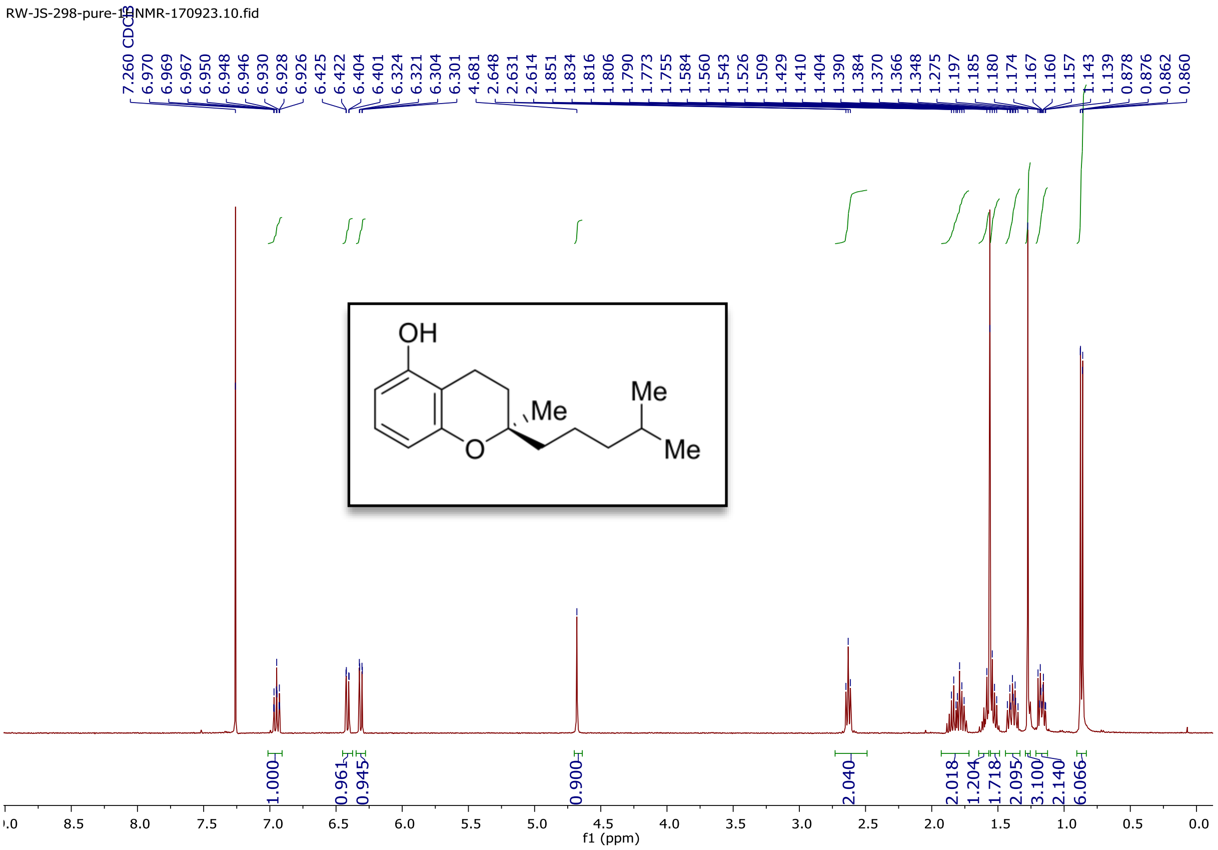

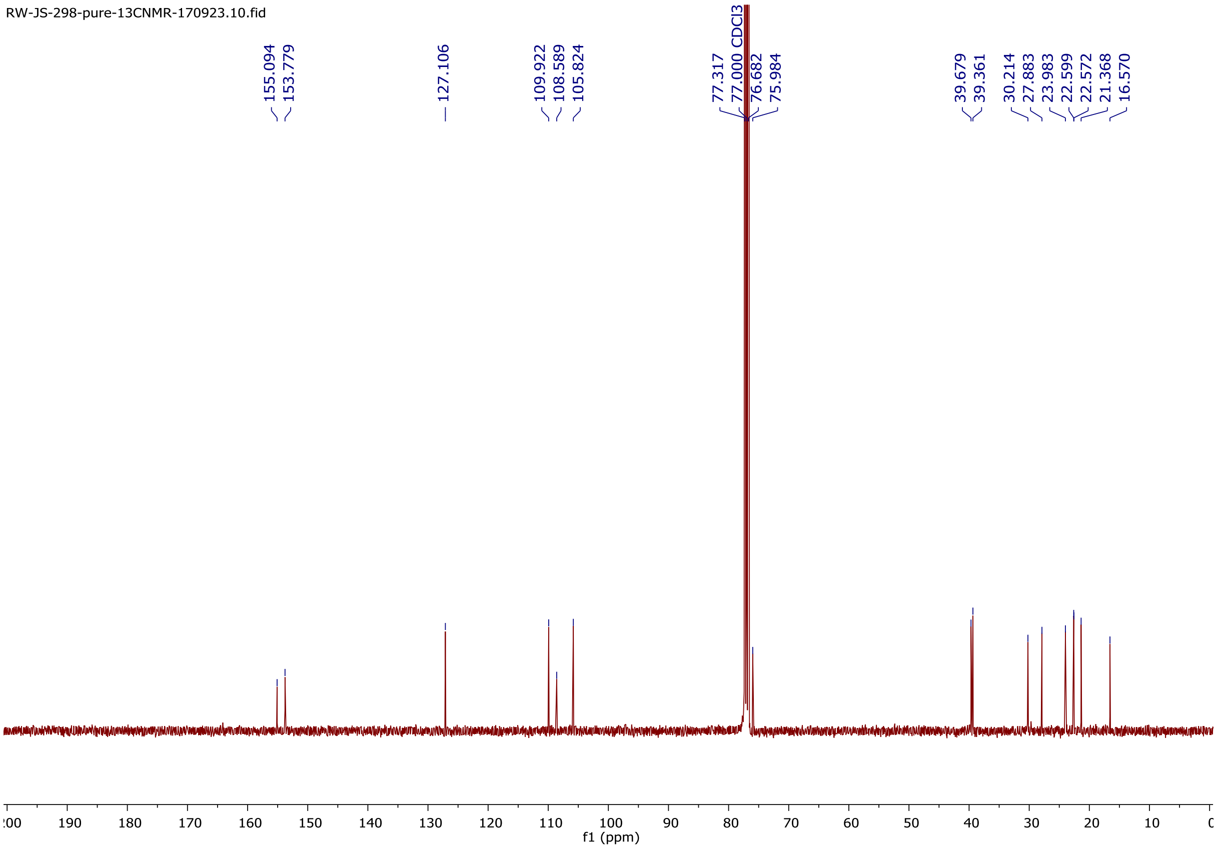

A1 (^{1}H NMR and ^{13}C NMR, $CDCl_3$)

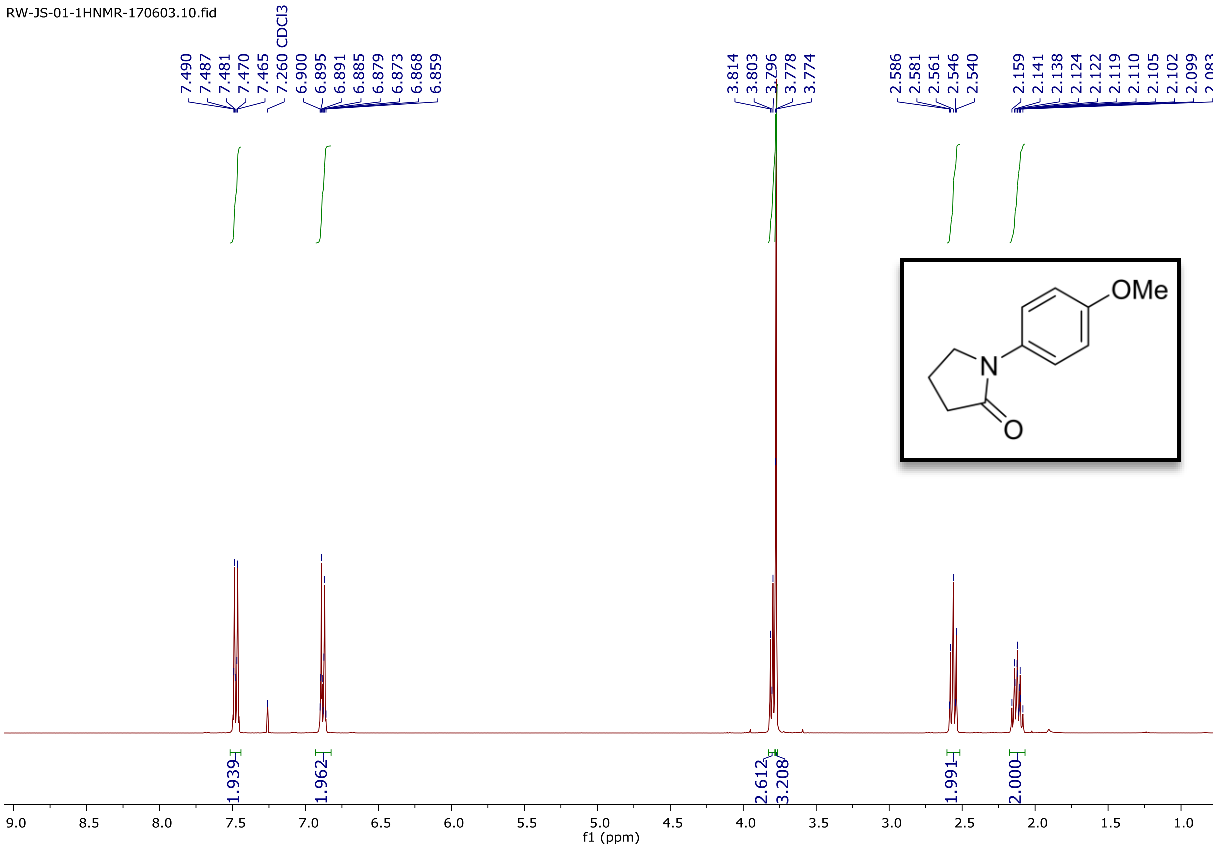

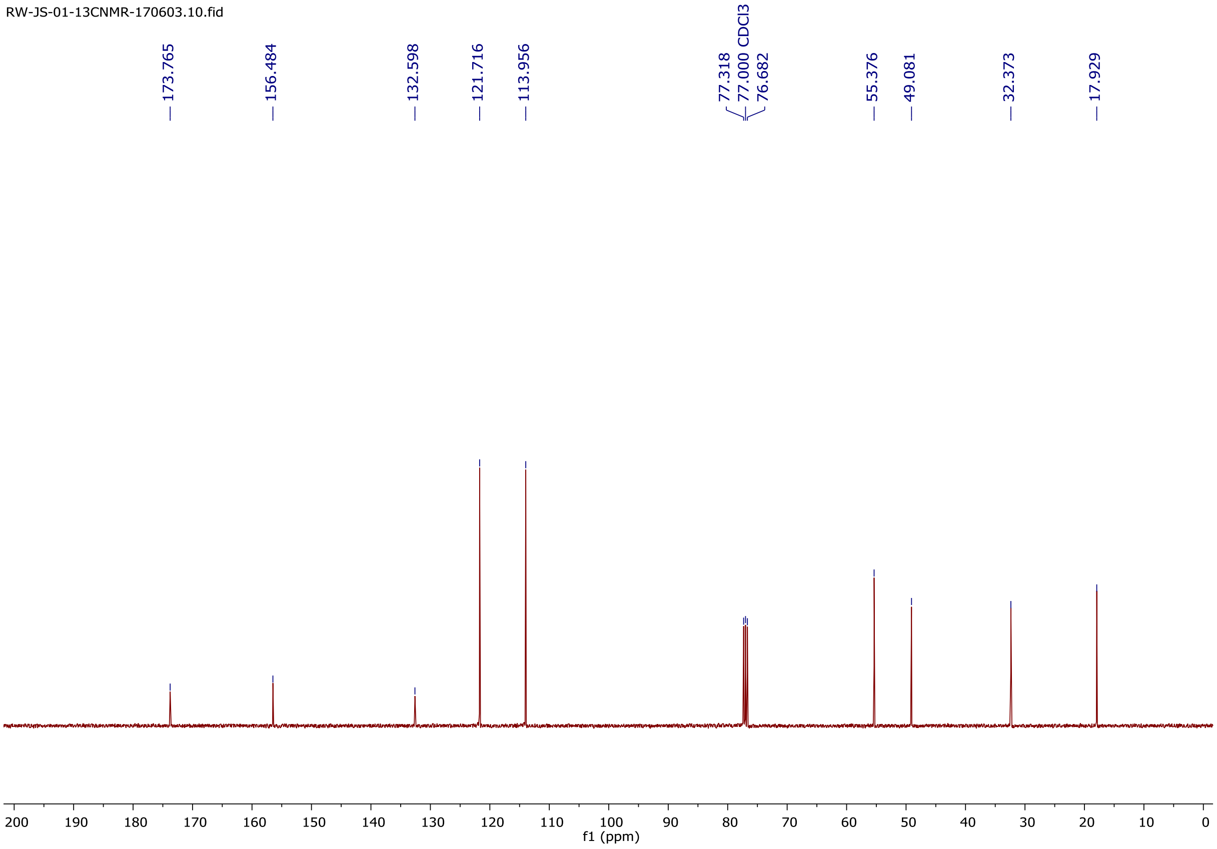

G1 (^{1}H NMR and ^{13}C NMR, $CDCl_3$)

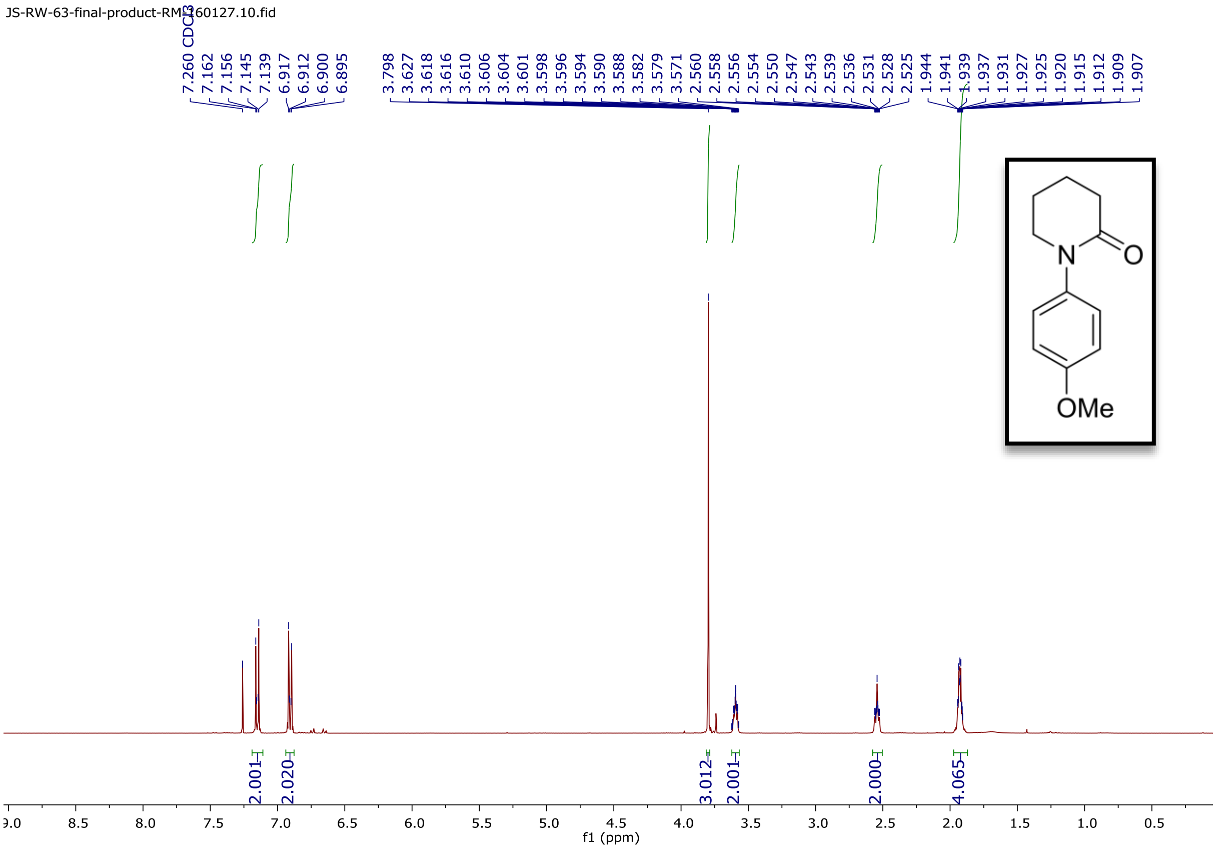

K (^{1}H NMR and ^{13}C NMR, $CDCl_3$)

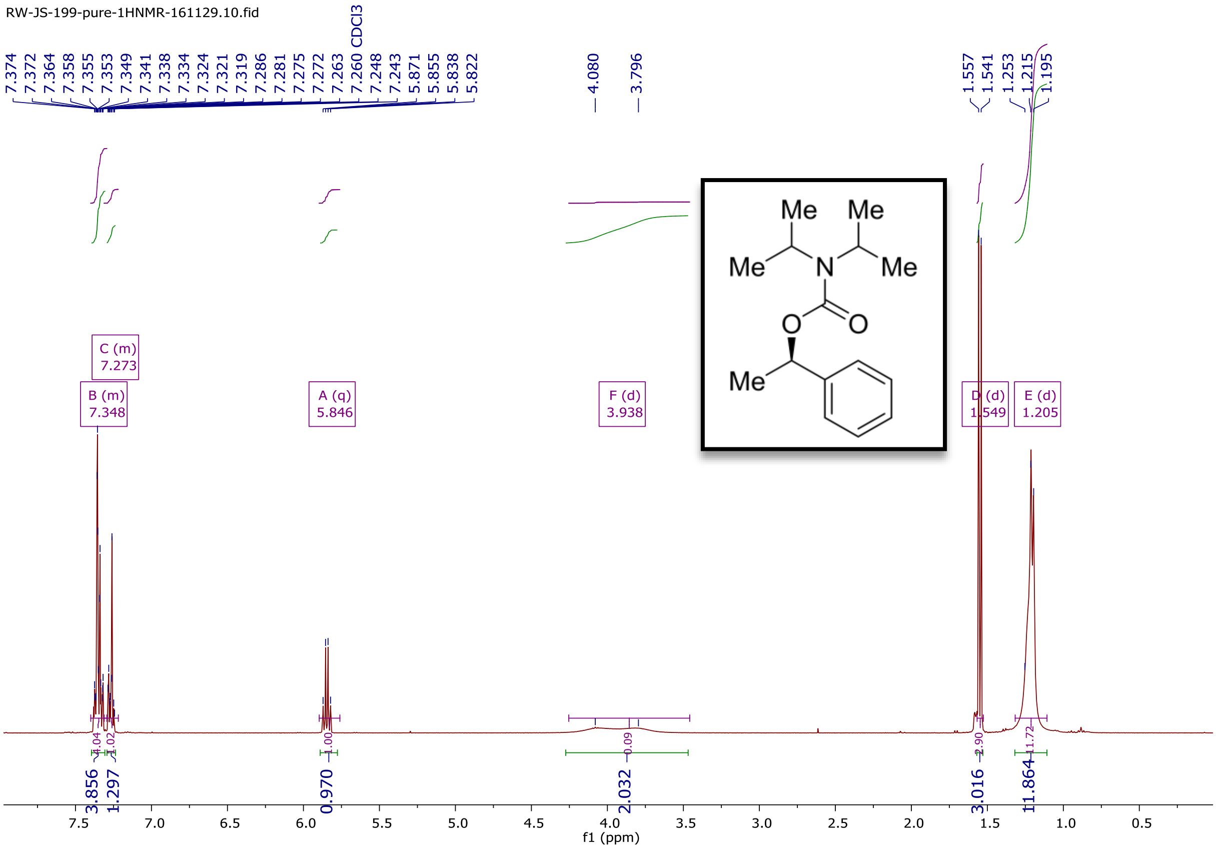

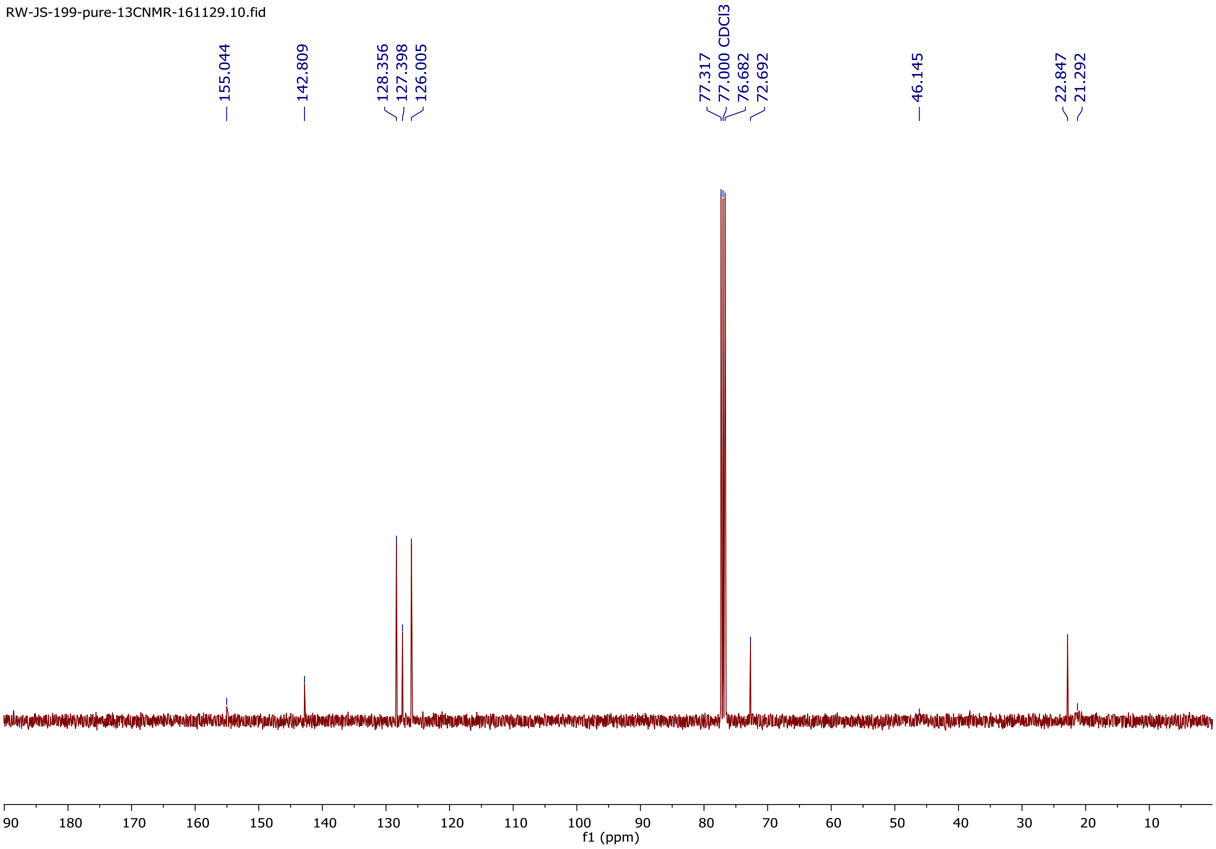

M (^{1}H NMR and ^{13}C NMR, $CDCl_3$)

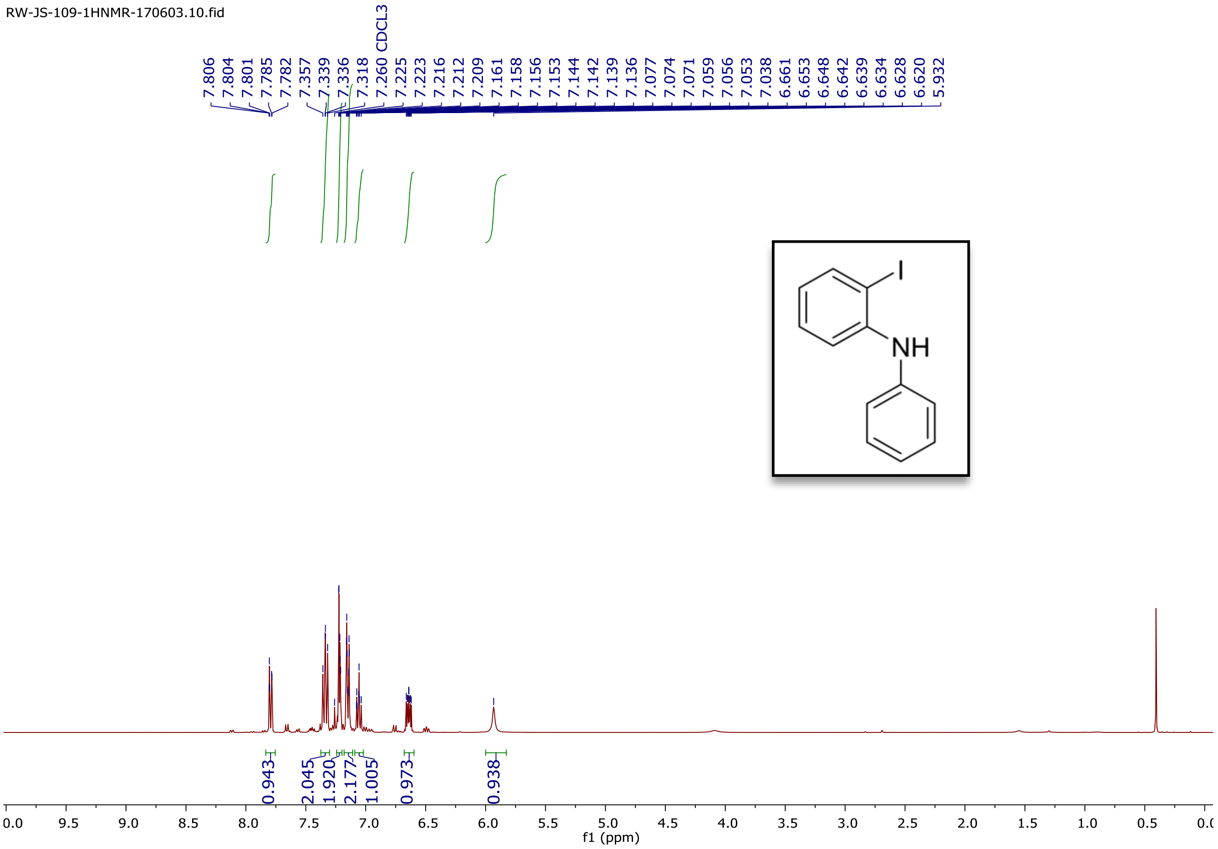

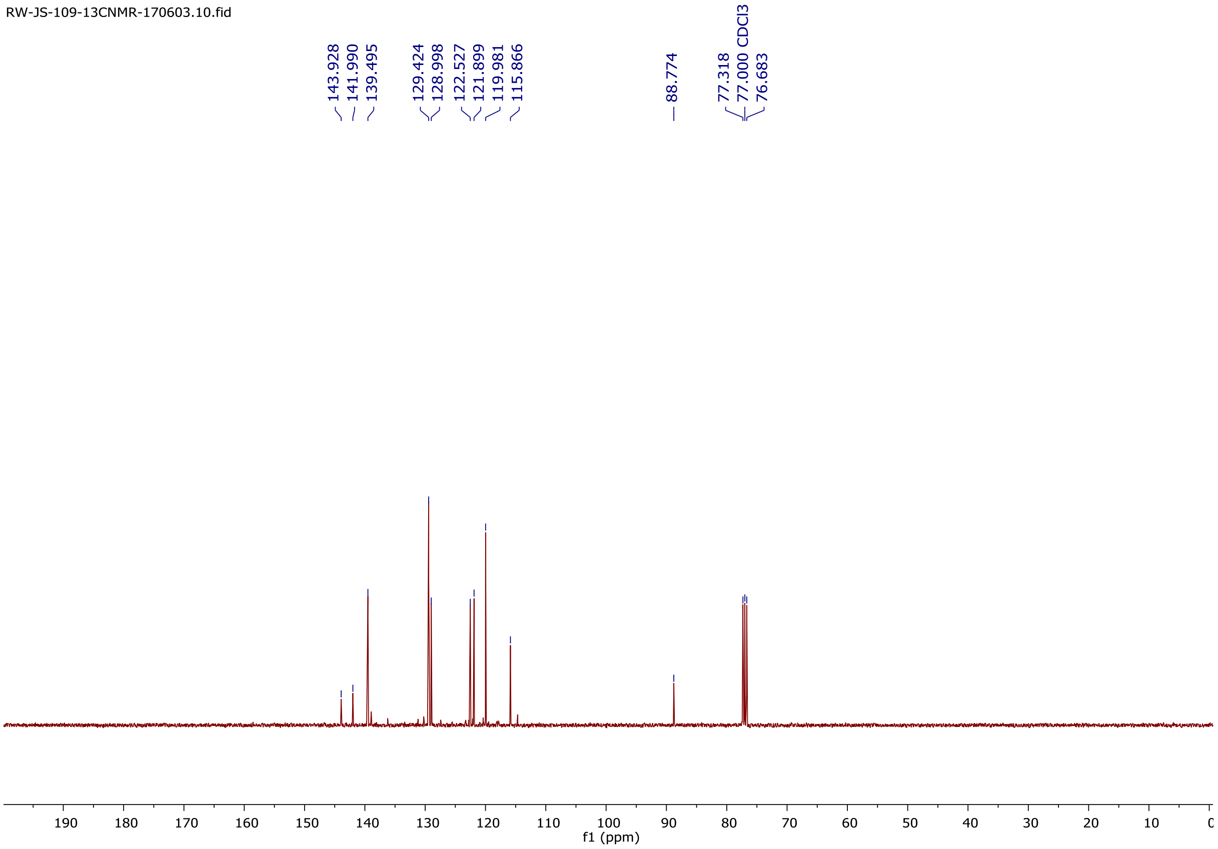

J (^{1}H NMR and ^{13}C NMR, $CDCl_3$)

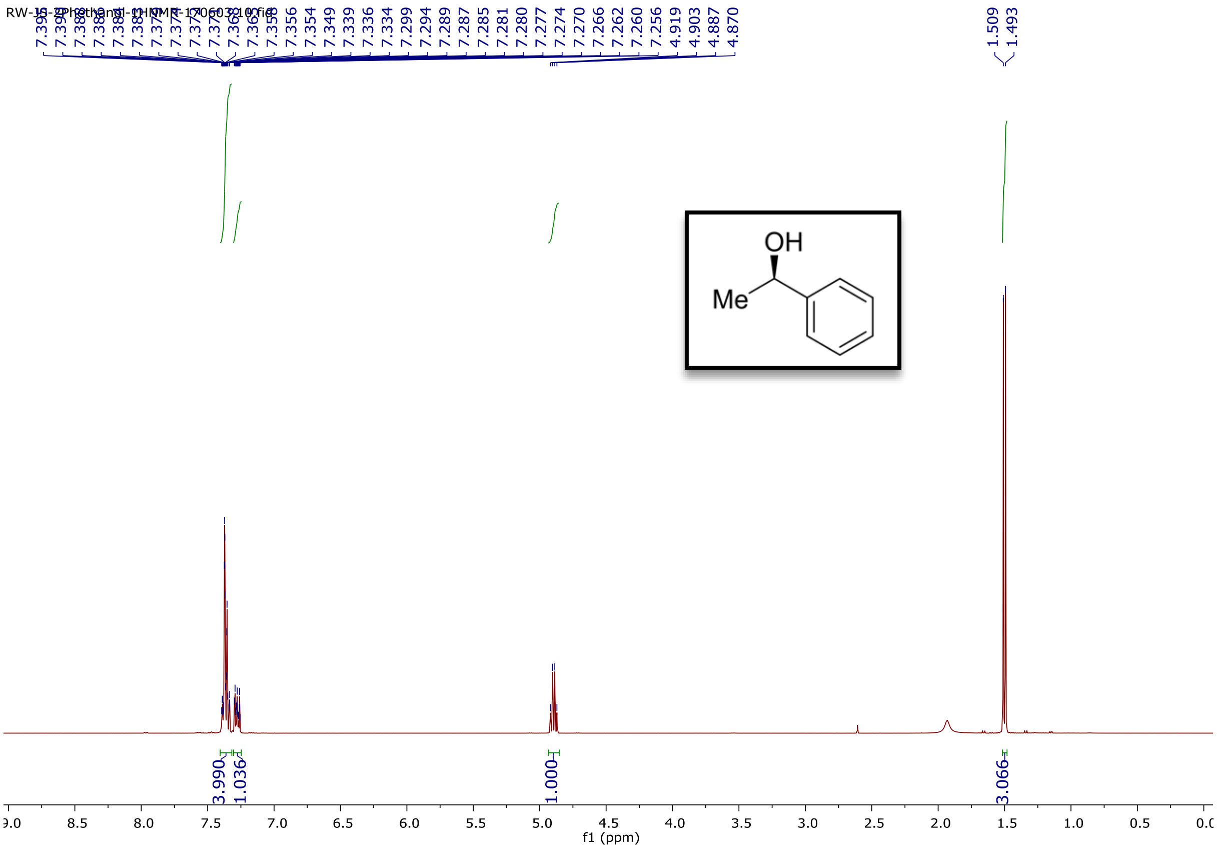

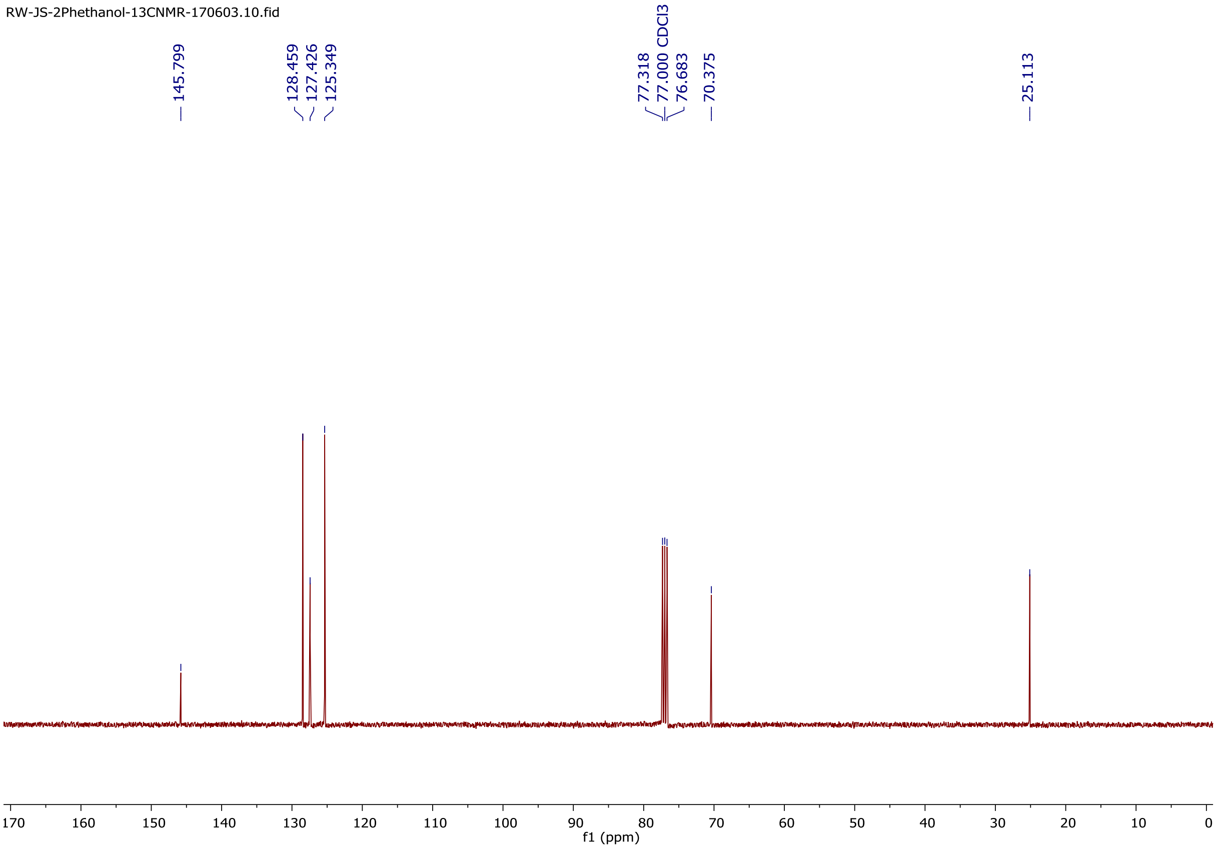

P (^{1}H NMR and ^{13}C NMR, $CDCl_3$)

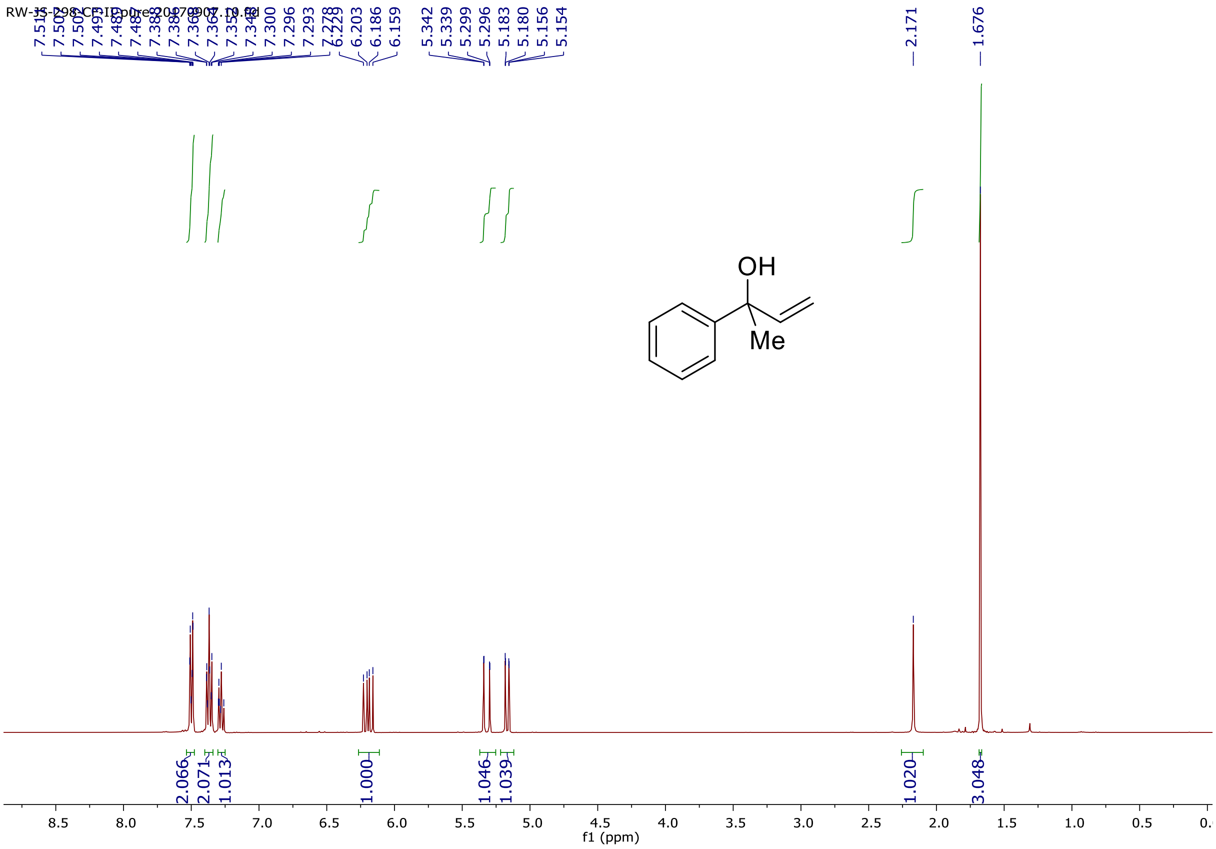

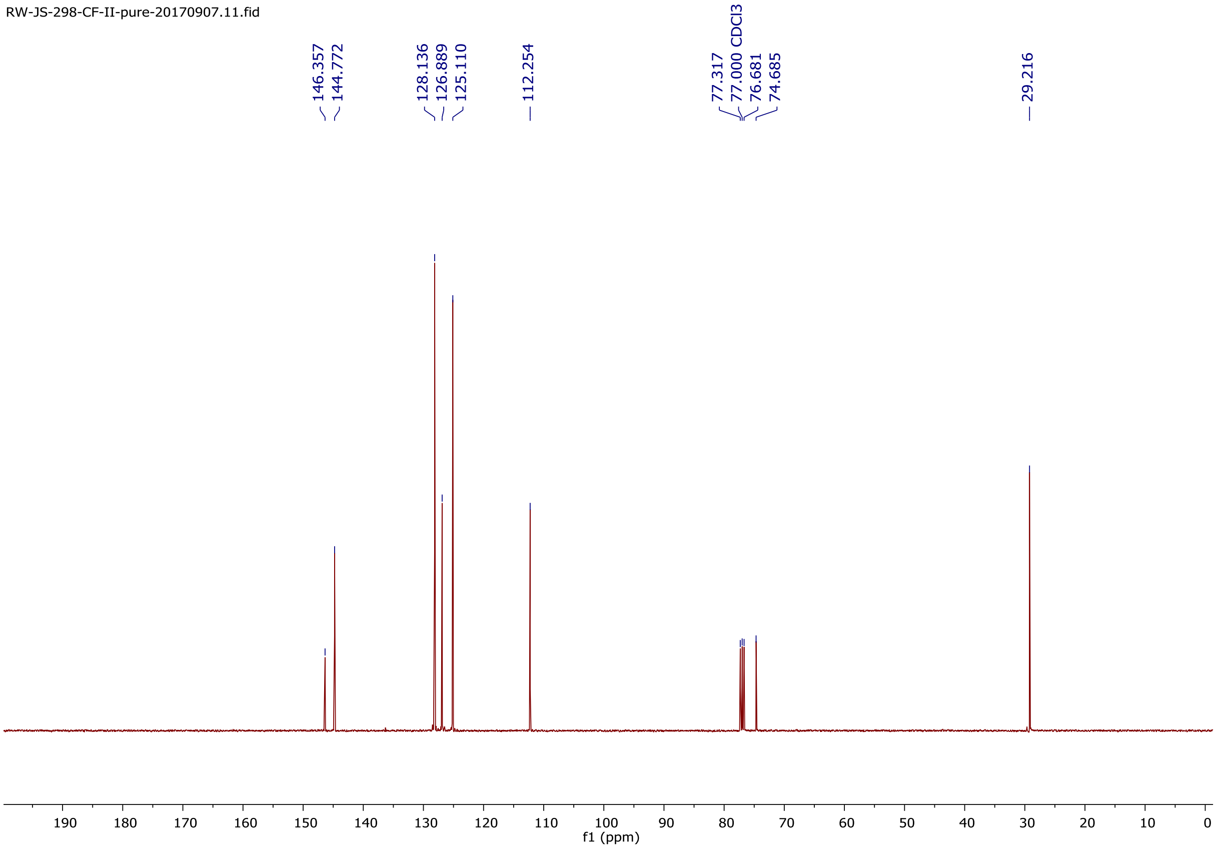

CC (^{1}H NMR and ^{13}C NMR, $CDCl_3$)

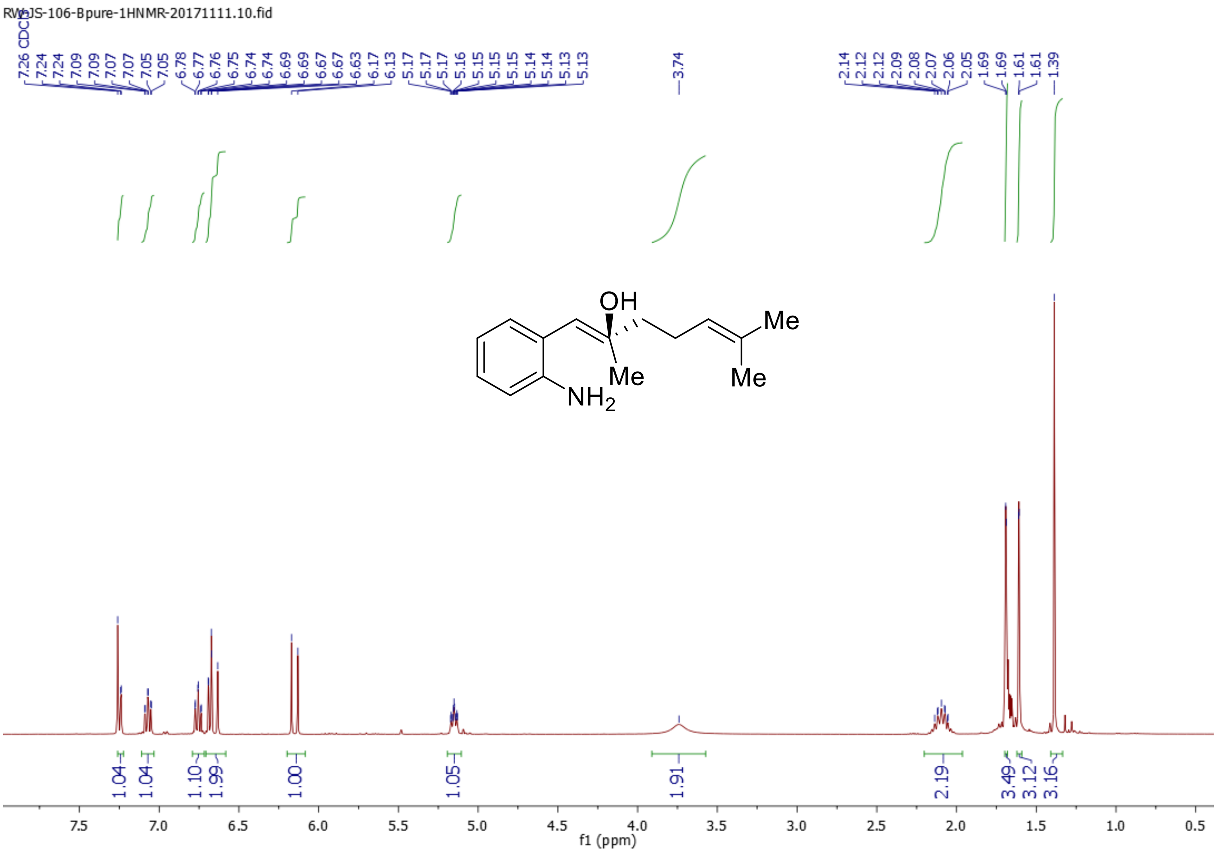

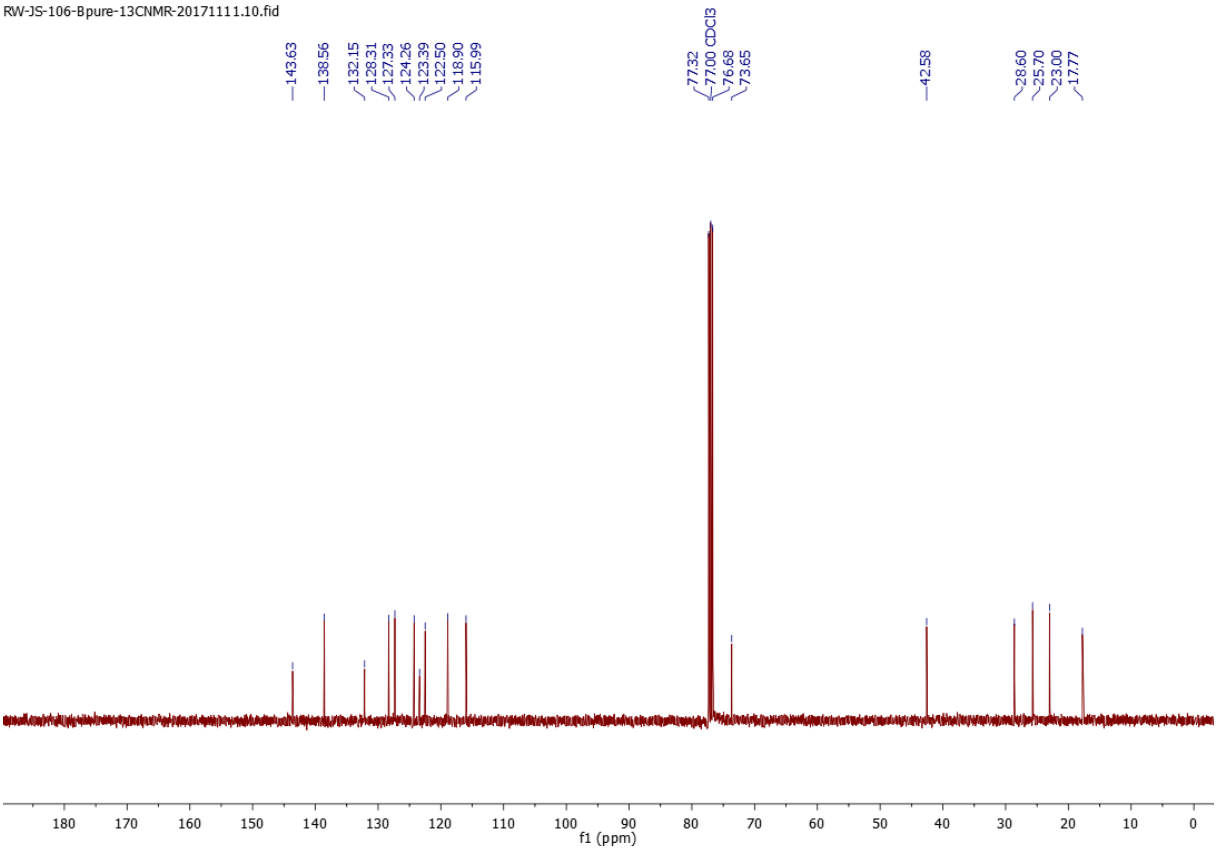

DD (^{1}H NMR and ^{13}C NMR, $CDCl_3$)

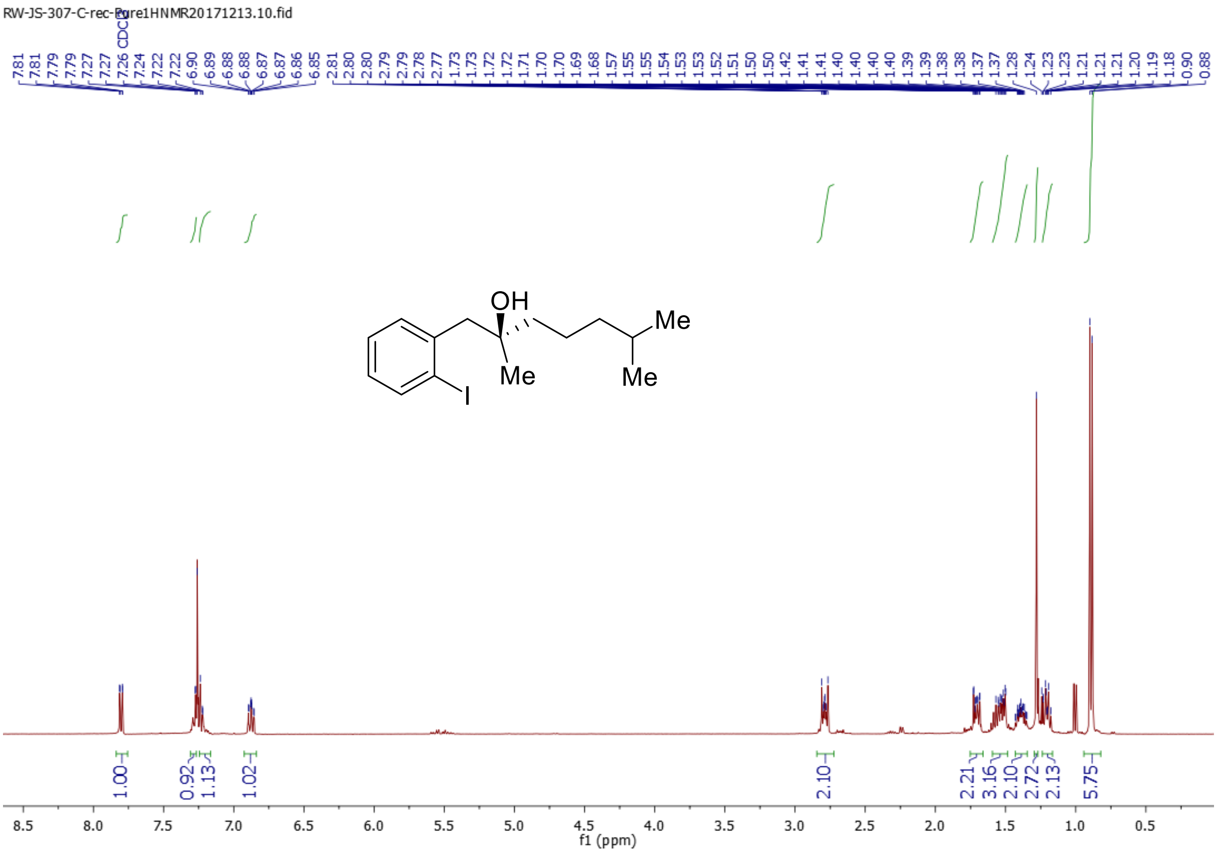

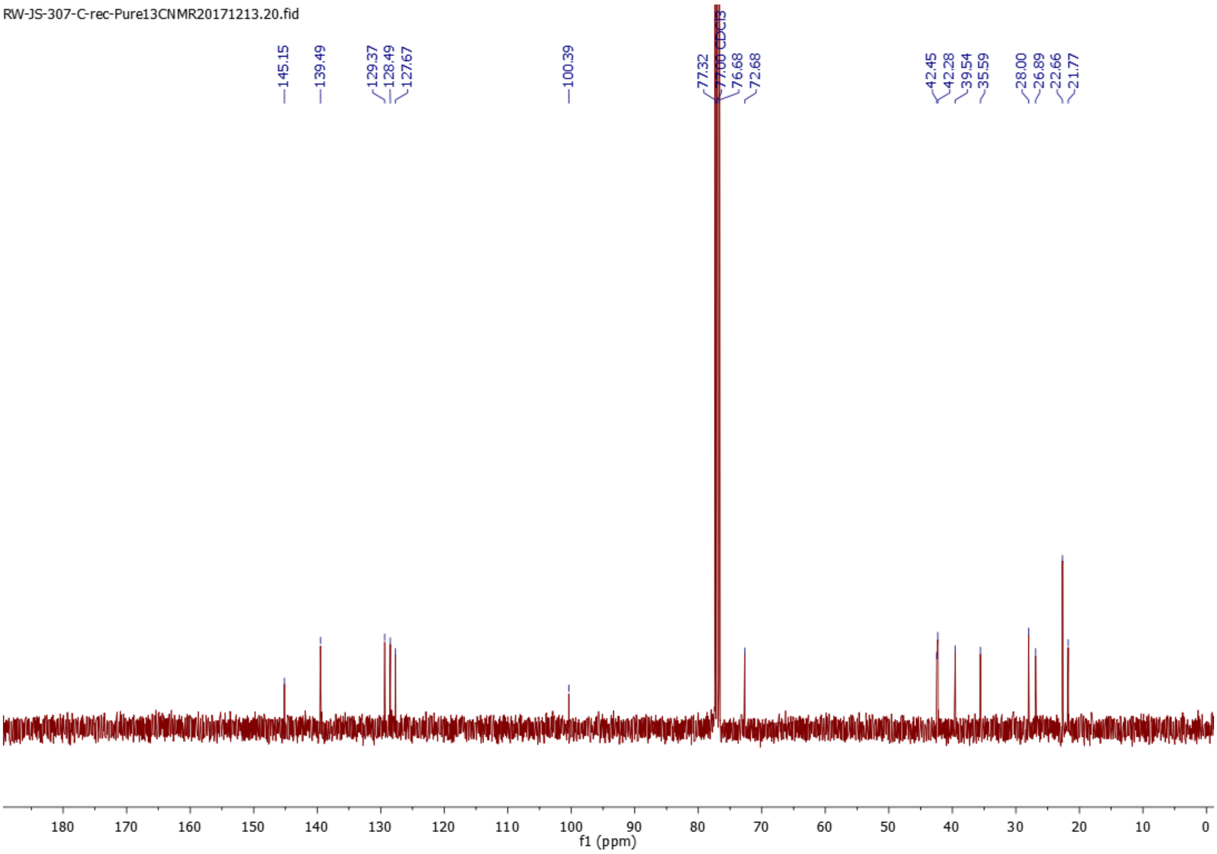

EE (^{1}H NMR and ^{13}C NMR, $CDCl_3$)

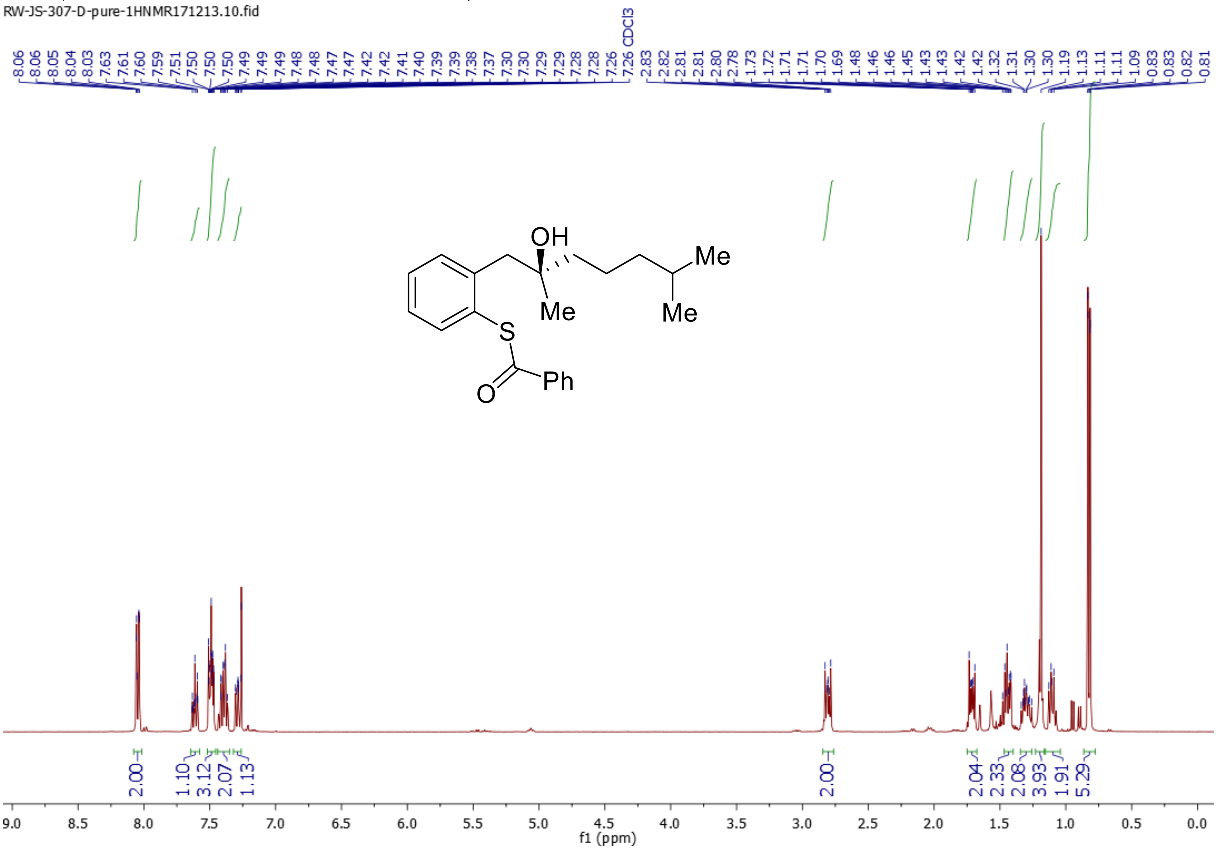

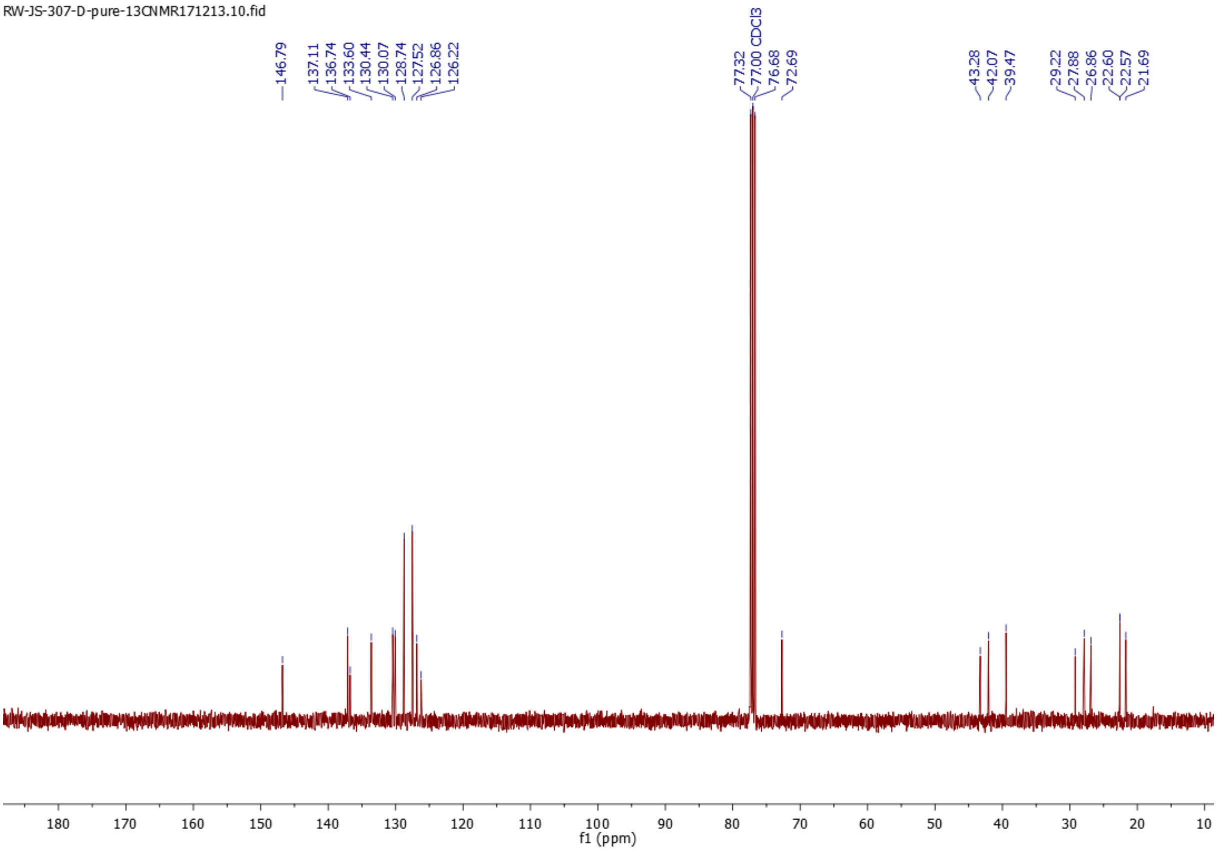

3 (^{1}H NMR and ^{13}C NMR, $CDCl_3$)

SA-I097-170704-2

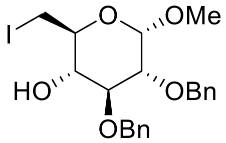

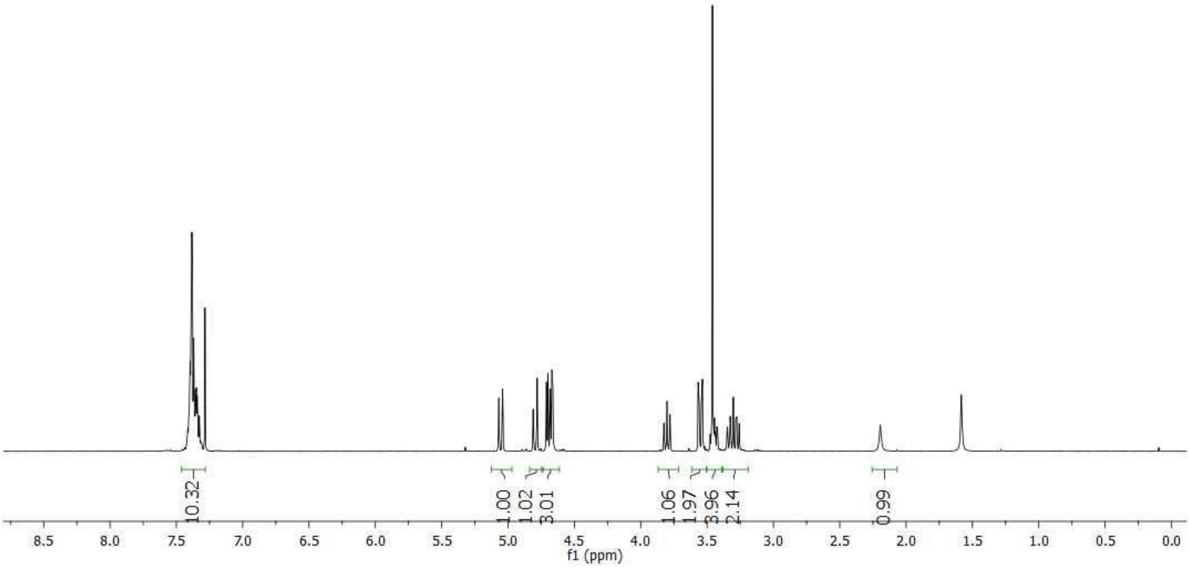

SA-I097-170704-2

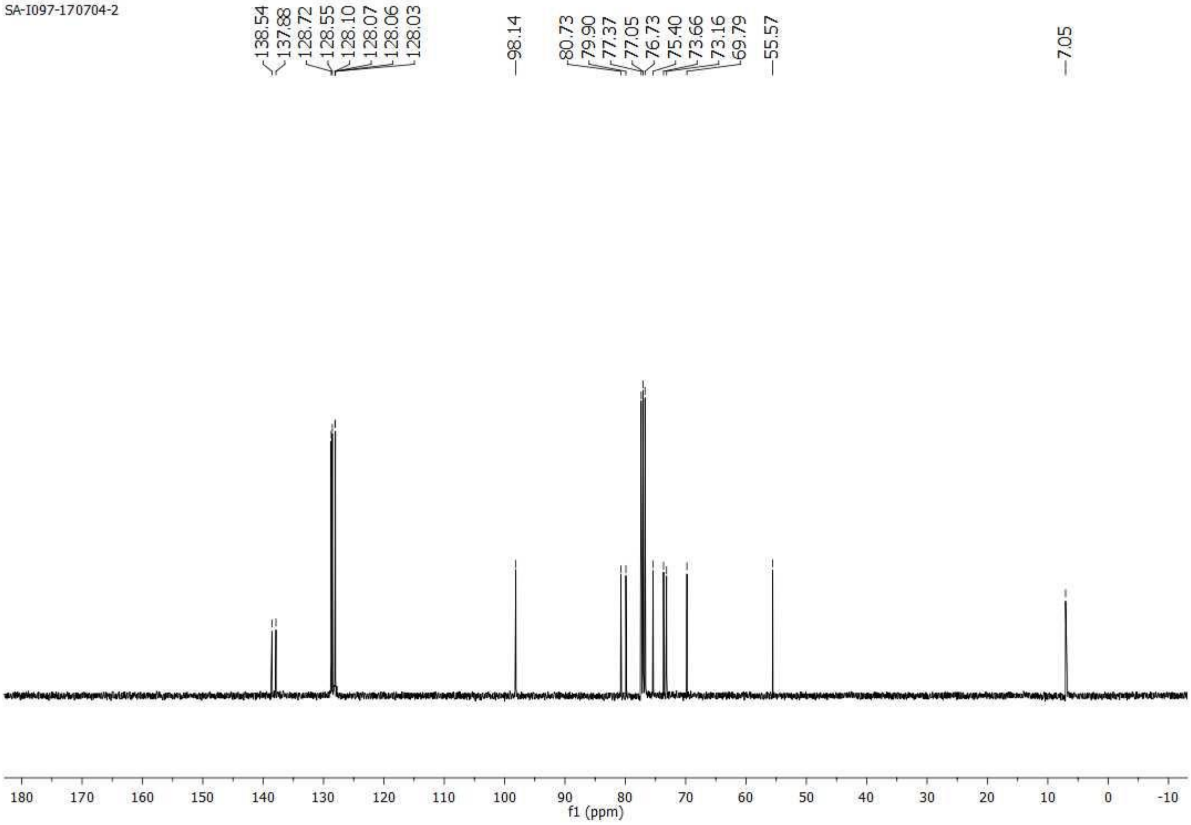

4 (^{1}H NMR and ^{13}C NMR, $CDCl_3$)

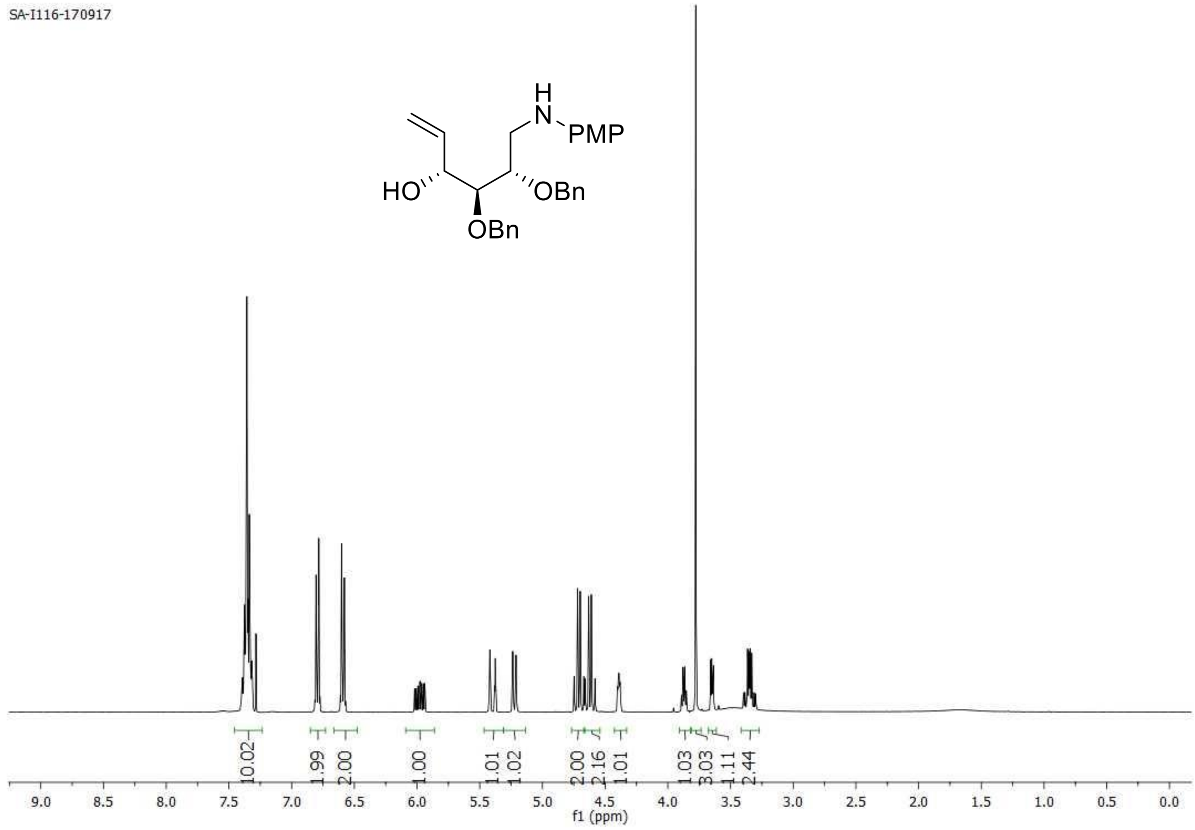

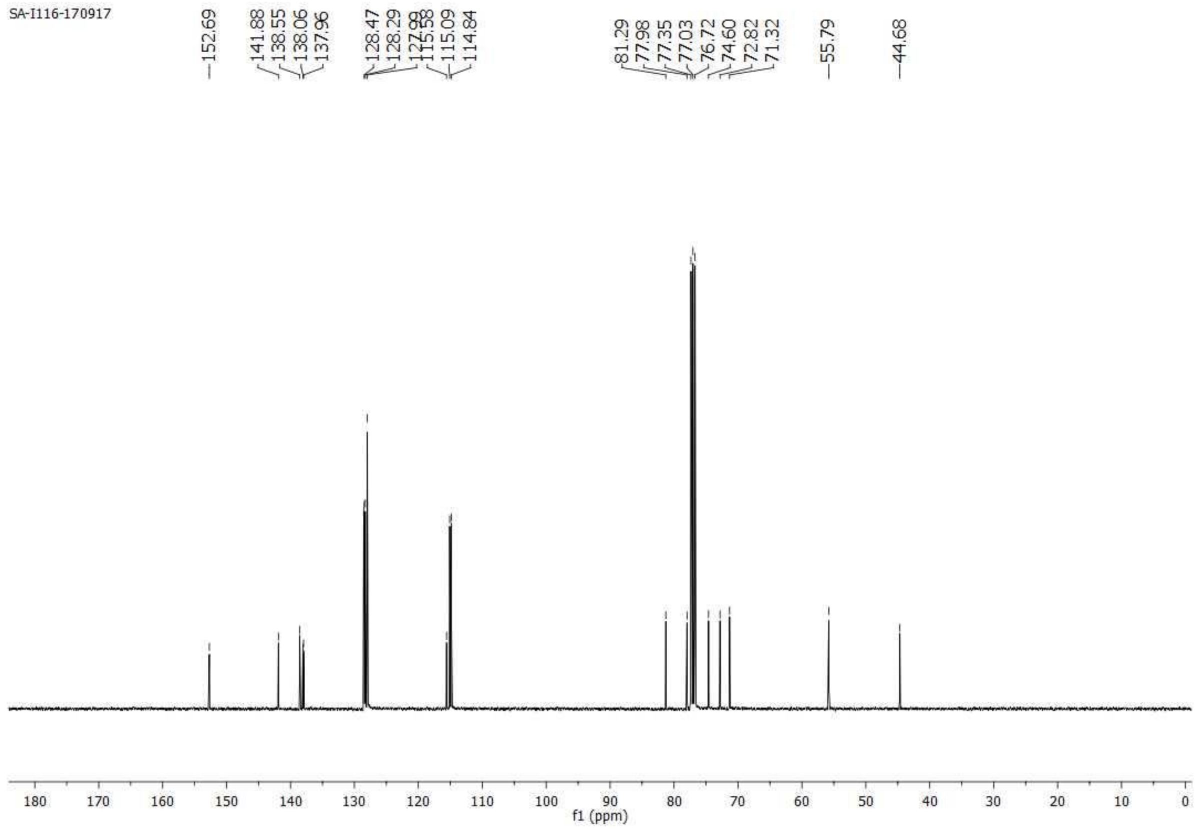

5 (^{1}H NMR and ^{13}C NMR, $CDCl_3$)

Rw-293B-SA-170916-2

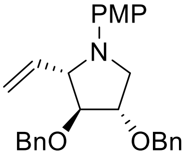

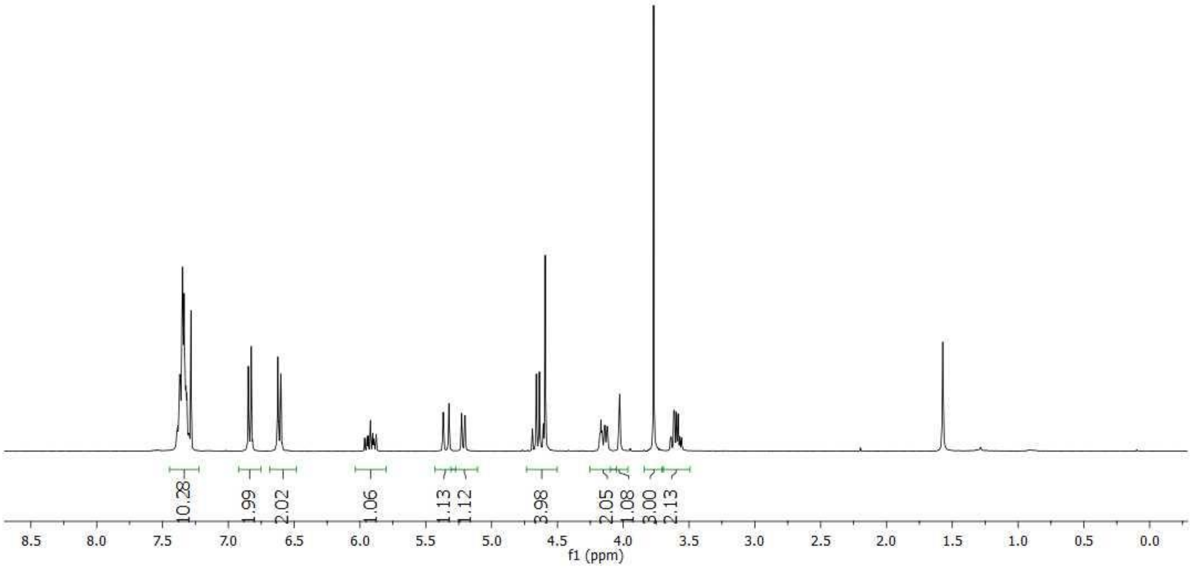

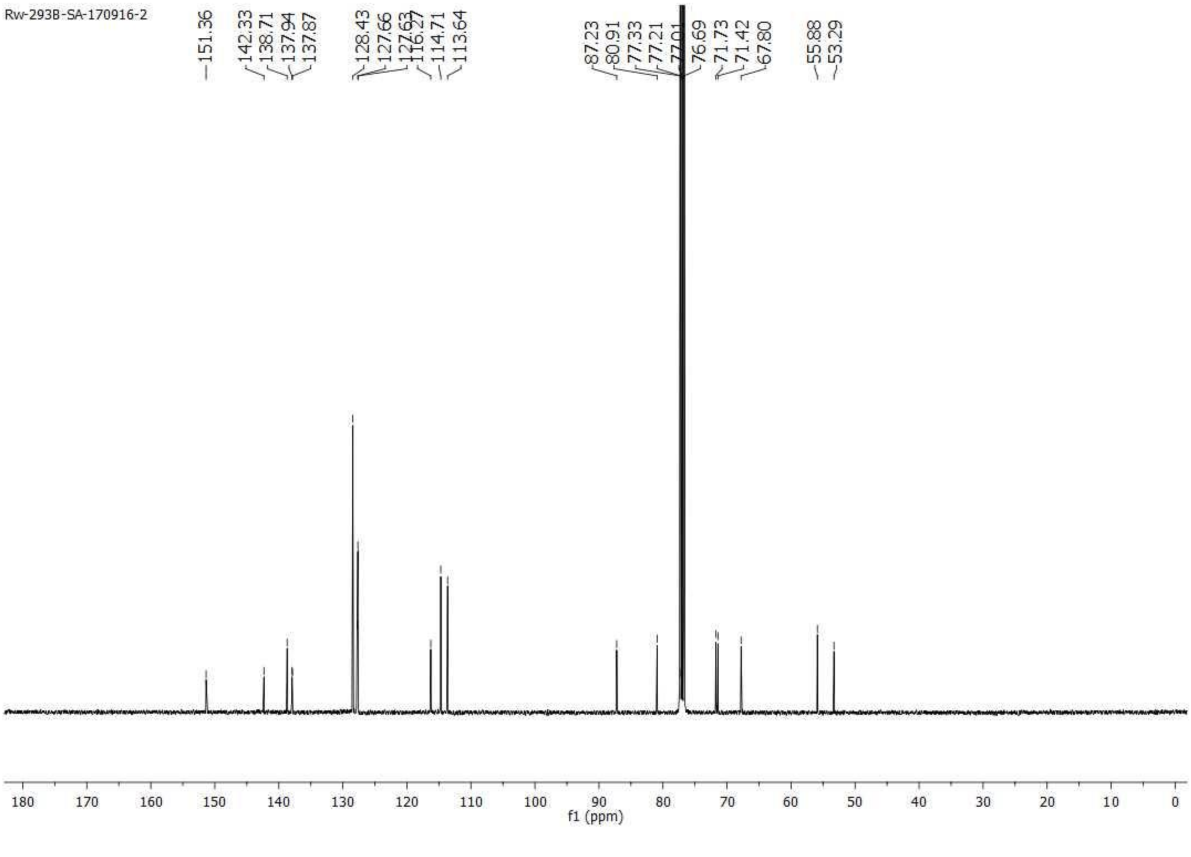

6 (^{1}H NMR and ^{13}C NMR, $CDCl_3$)

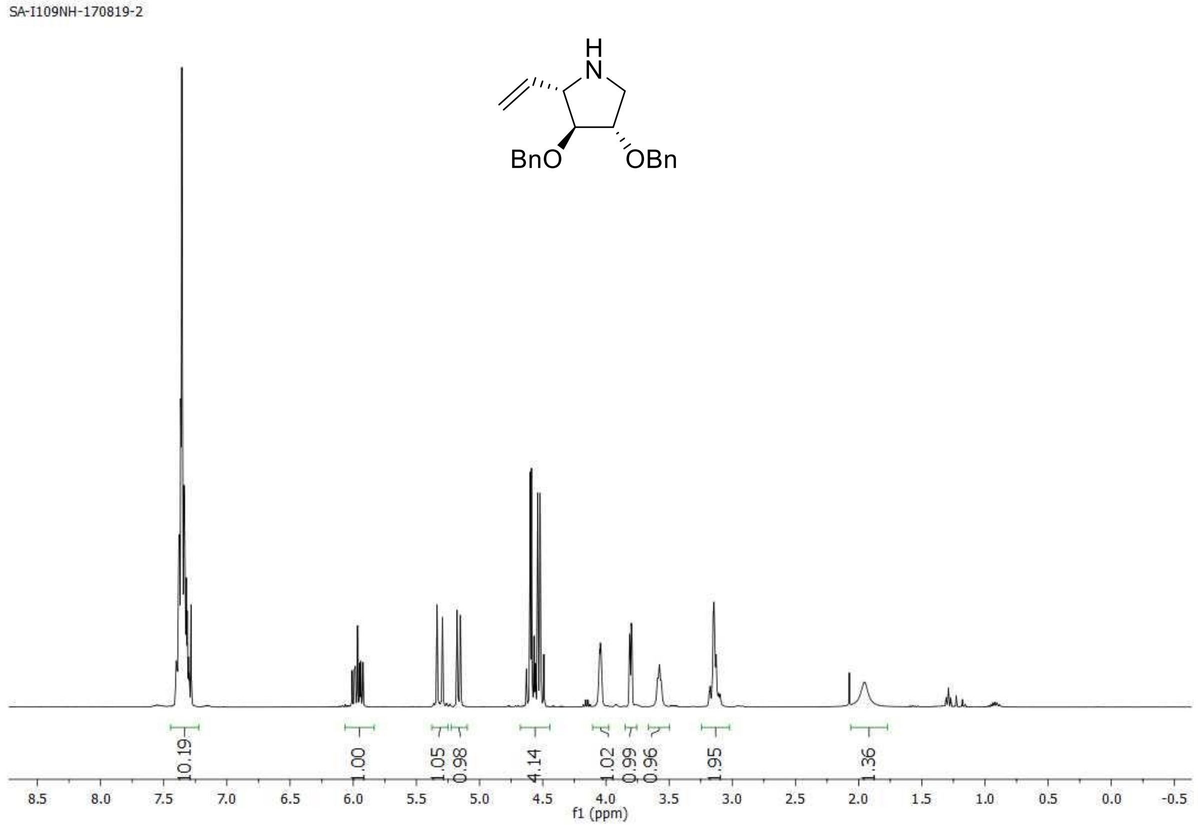

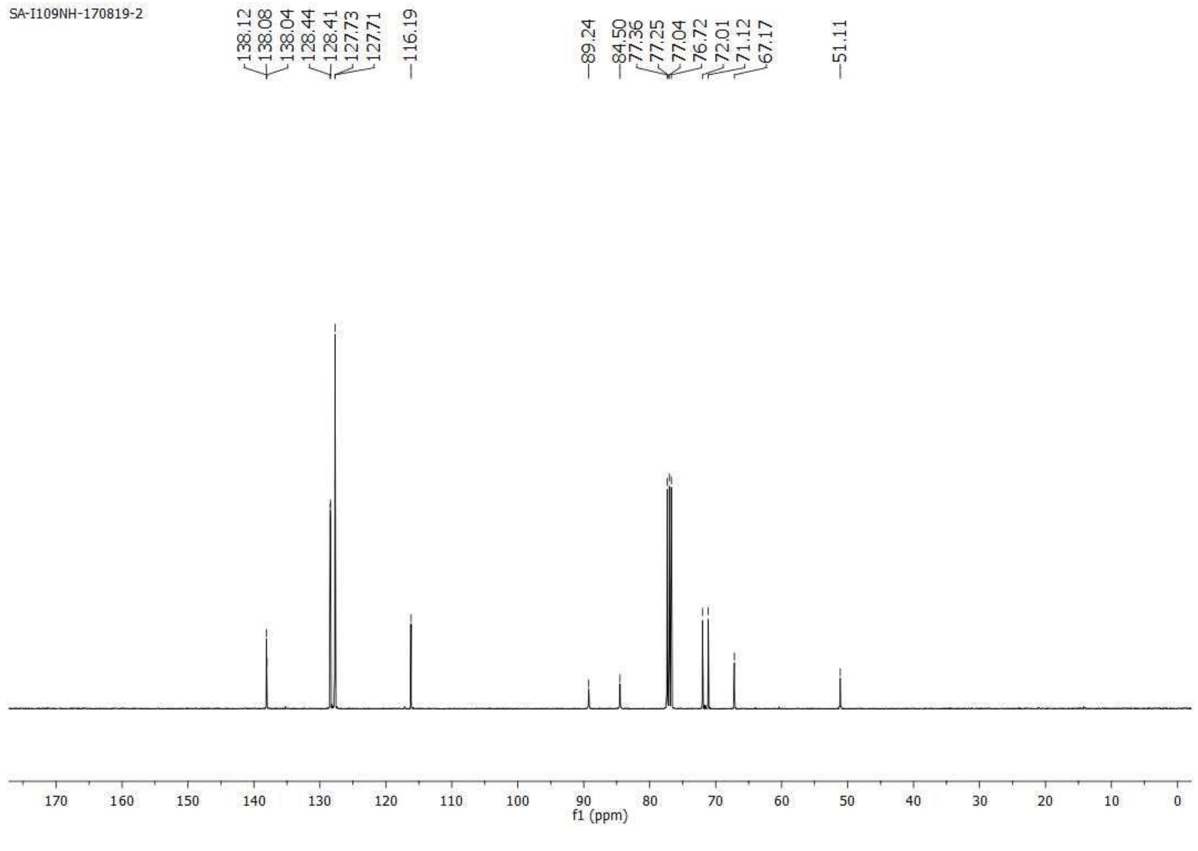

(^{1}H NMR and ^{13}C NMR, $CDCl_3$)

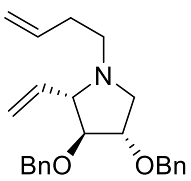

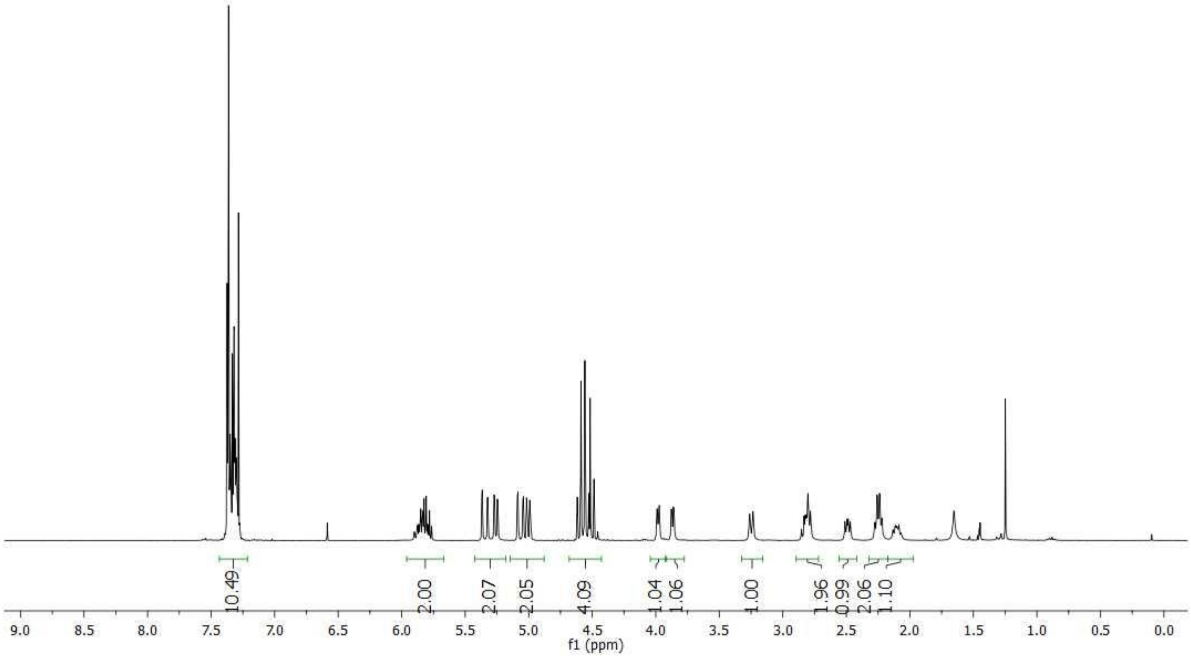

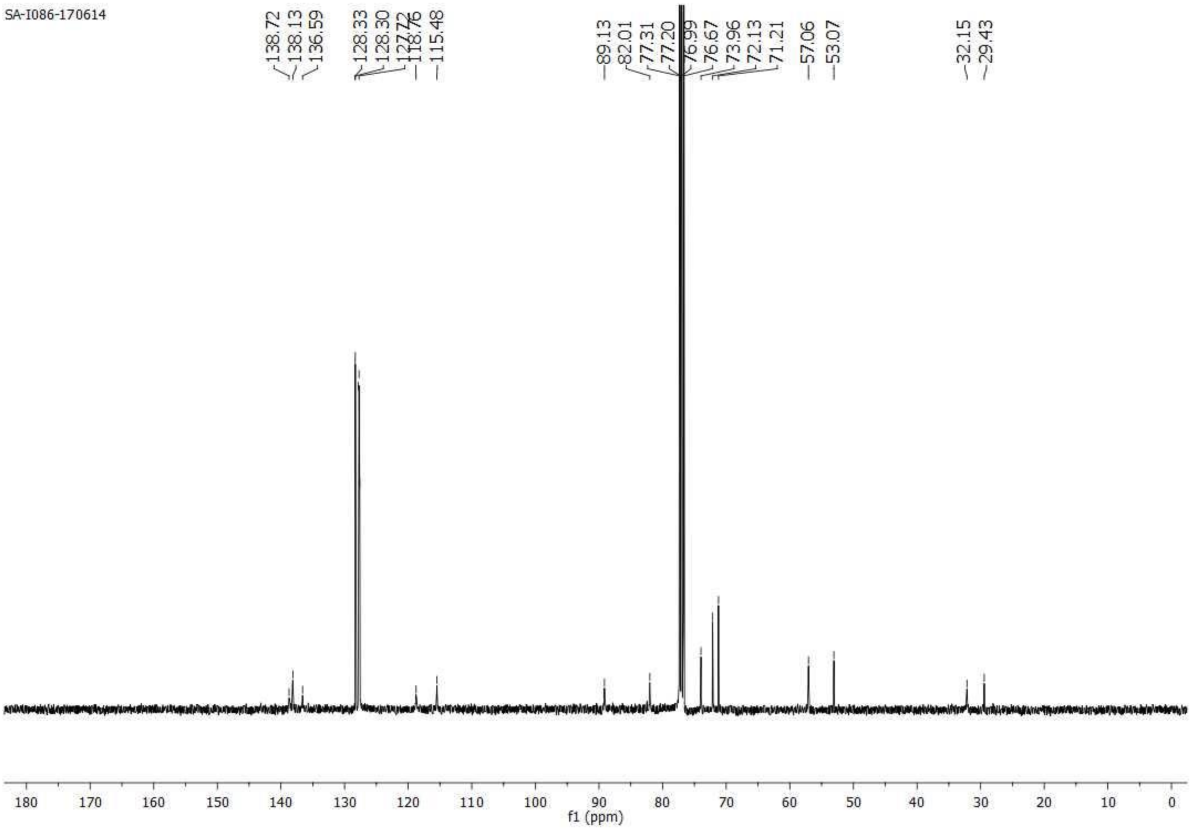

7a (^{1}H NMR and ^{13}C NMR, $CDCl_3$)

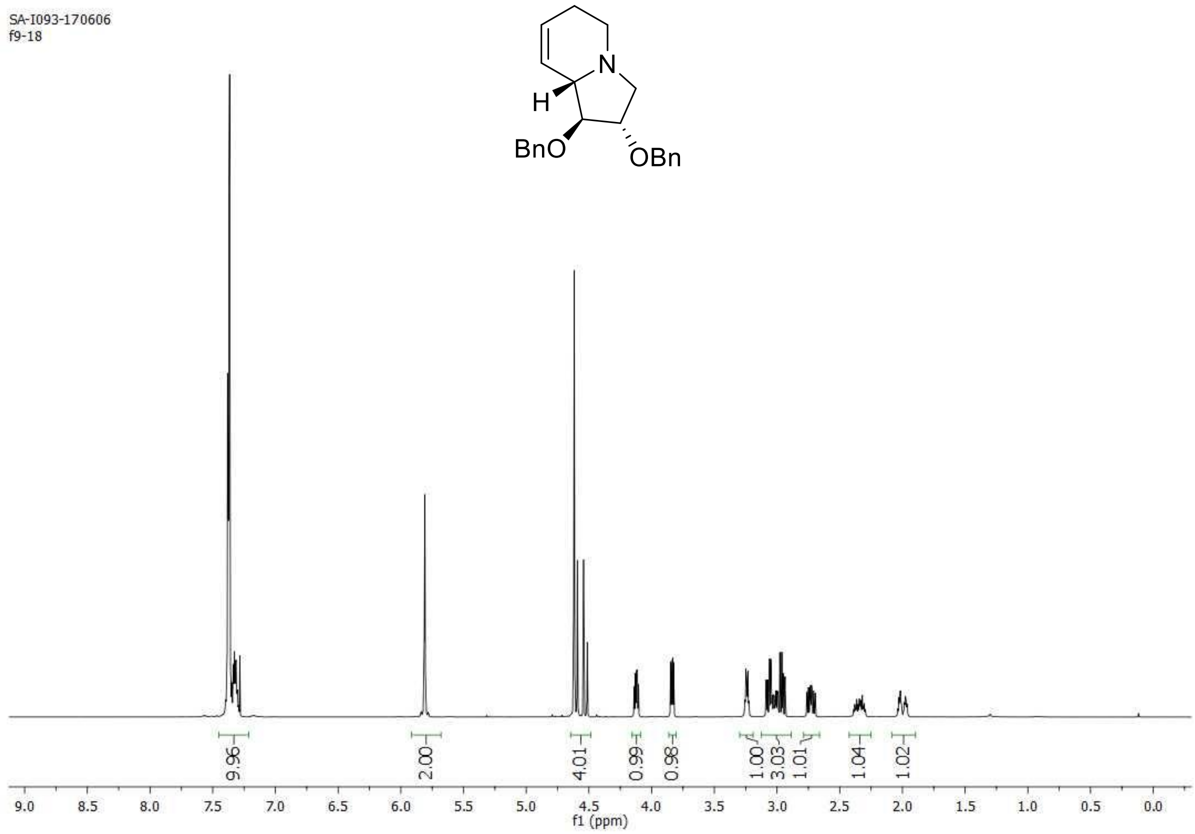

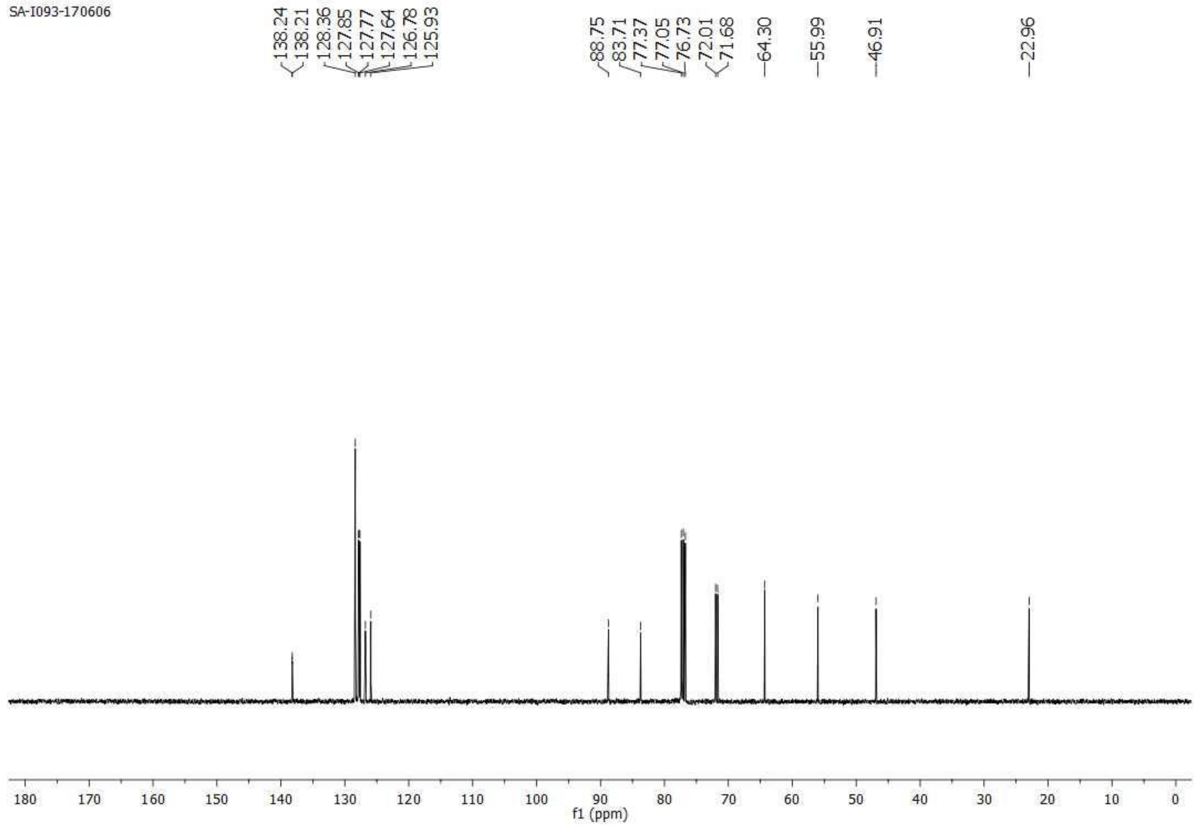

(+)-Lentiginosine (**8**) (^{1}H NMR and ^{13}C NMR, D_2O)

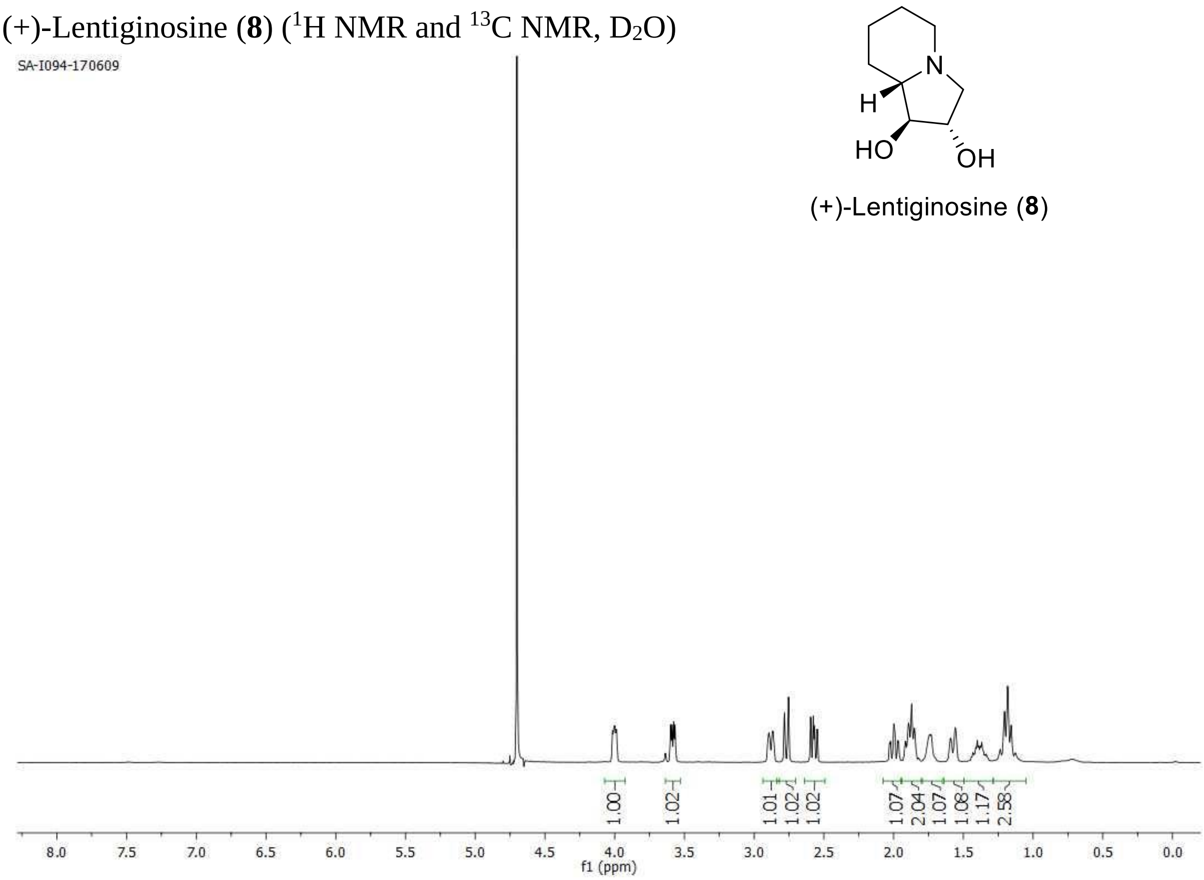

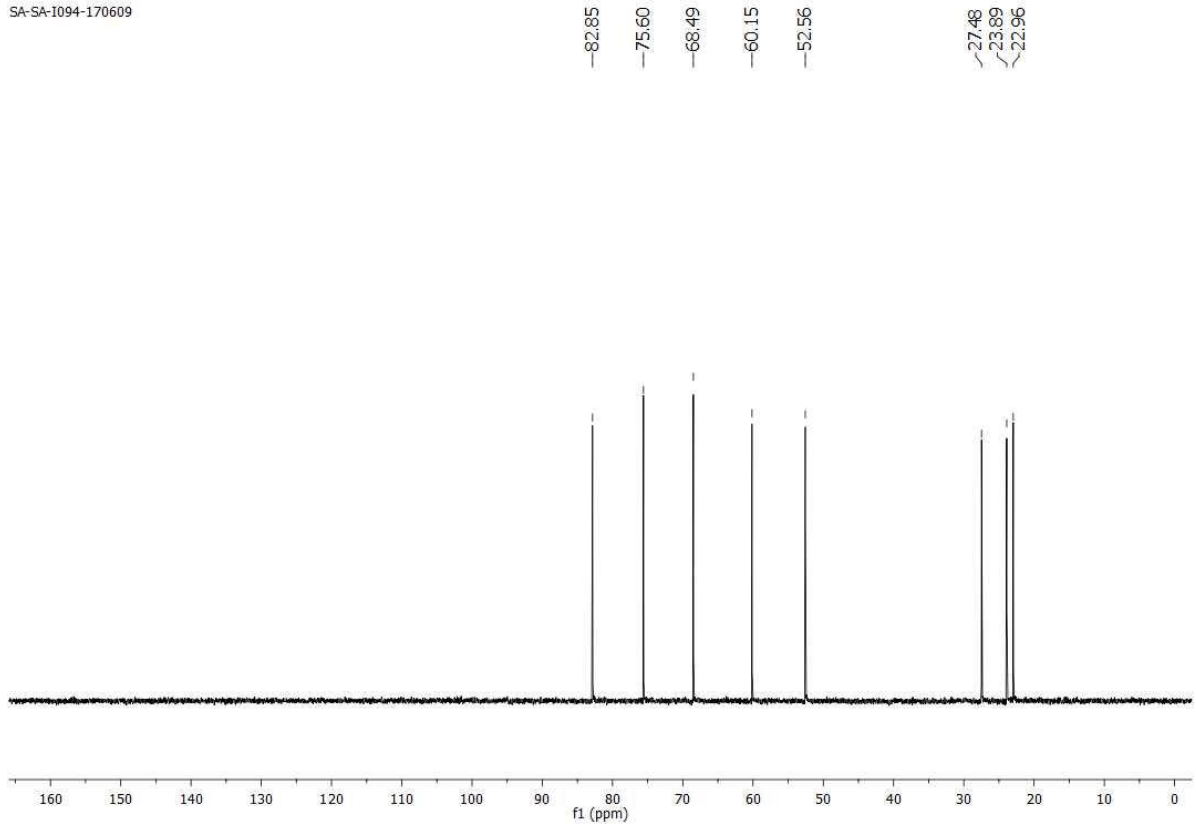

Supplementary Note 2

Computational details: Geometries of all transition states and intermediates were optimized using the Gaussian 09 program,[17] employing the unrestricted-B3LYP[18,19] density functional (UB3LYP), the LANL2DZ[20] basis set for iron and the 6-31+G**[21-23] basis set for all other elements. Solvation correction was applied in the course of the optimizations using the PCM model with the default parameters for dichloroethane.[24-26] The complexes were treated with charge 0 and three possible singlet states were evaluated (doublet, quartet and sextet). Nature of the stationary points, minima or transition states (TS), were confirmed by performing vibrational frequency calculations at 298.15 K. The energies were further refined by performing single-point calculations using the above-mentioned parameters, with the exception that the 6-311+G(2d,2p)[27,28] basis set was used for all elements except for iron, and by applying dispersion correction using the DFT-D3[2930] model. All energies reported are Gibbs free energies at 298.15 K and calculated as $G_{reported} = G_{6\text{-}31+G^{**}} + (E_{6\text{-}311+G(2d,2p)} - E_{6\text{-}31+G^{**}}) + E_{DFT\text{-}D3}$.

Supplementary Figure 5: DFT calculations for intramolecular substitution with substrate **1d**

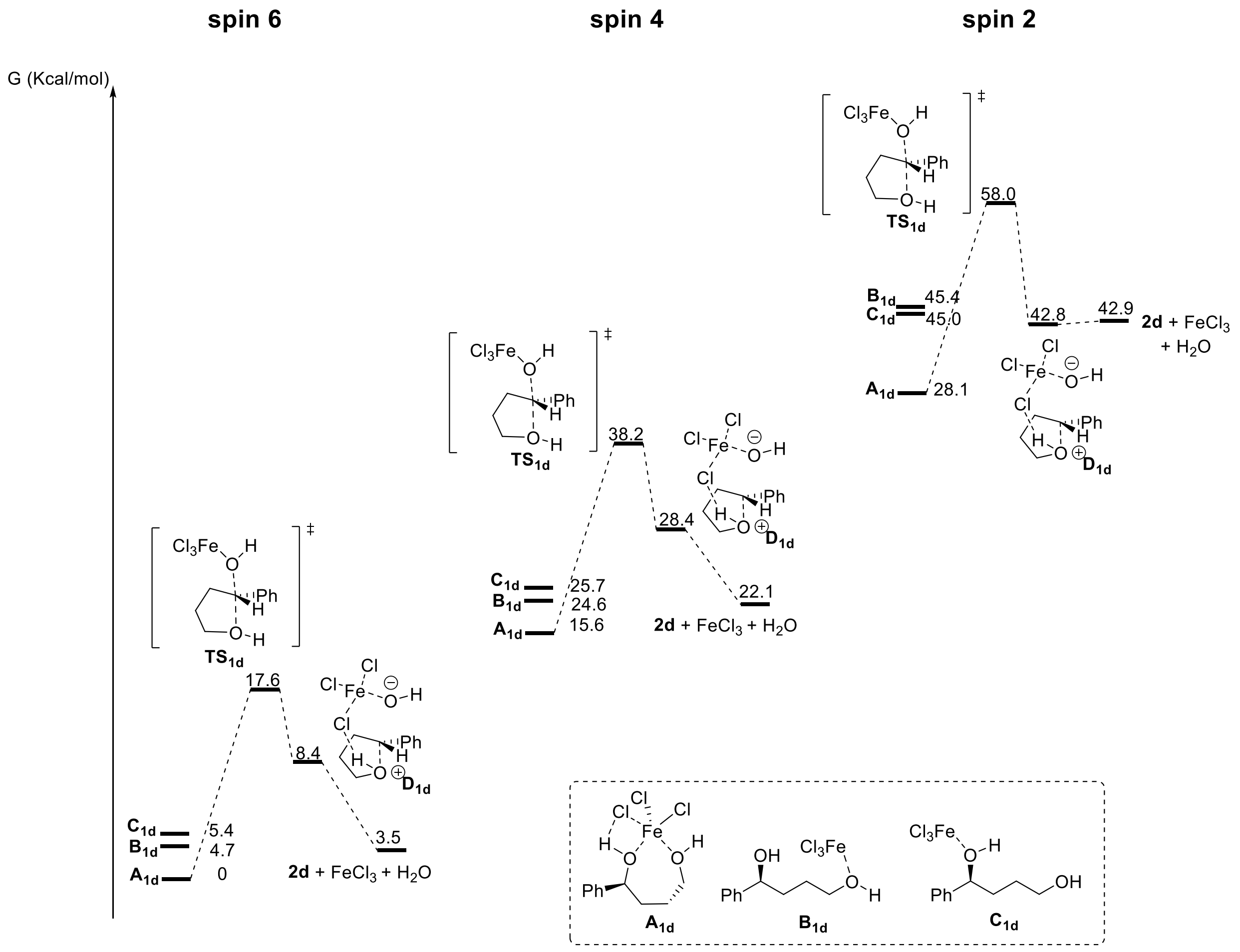

Supplementary Figure 5: Energy profile of the intramolecular substitution for substrate **1d** considering all possible spin states for iron (sextet, quartet and doublet).

Supplementary Figure 6: DFT calculations for intramolecular substitution with substrates **1a** and **1c**

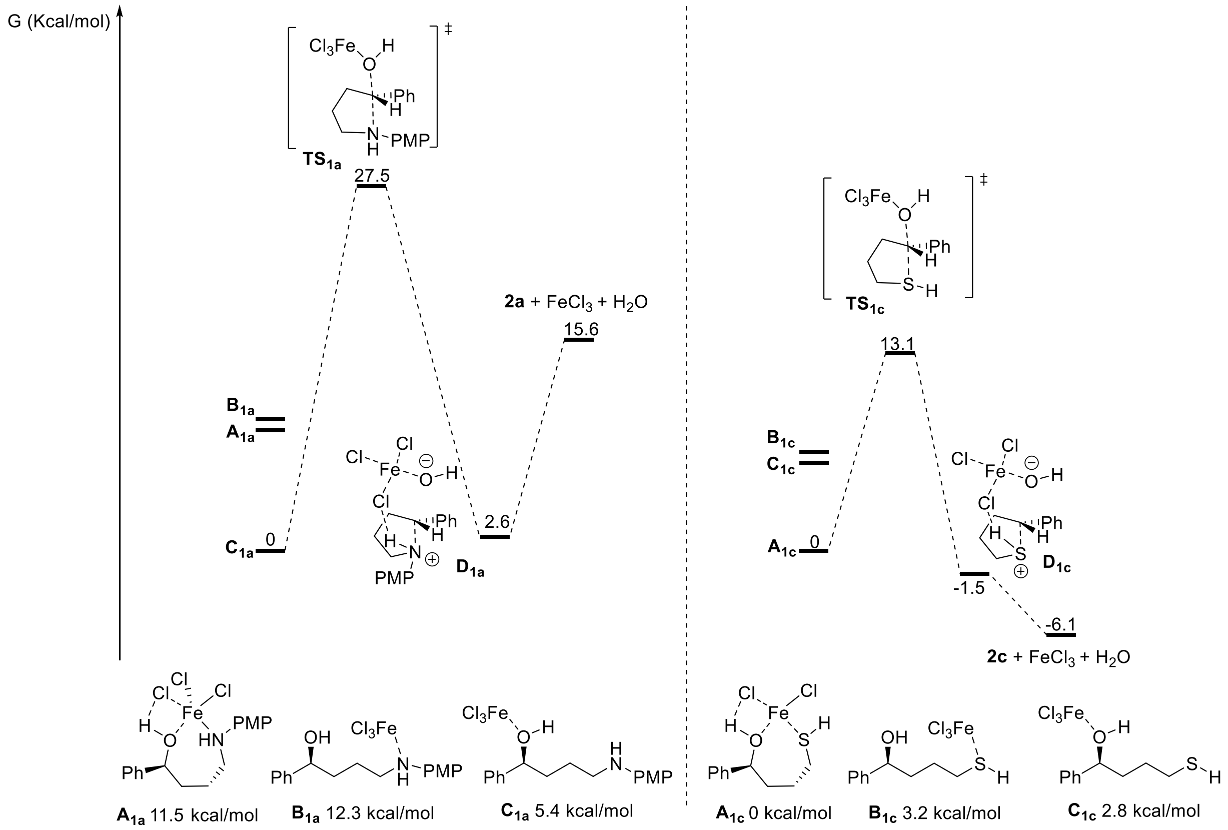

Supplementary Figure 6: Energy profile of the intramolecular substitution for substrates **1a** and **1c** considering the sextet spin state for iron(III).

Supplementary Table 4: Energies and structures of intermediates and transition states involving substrates 1a,c-d

Structure	E(UB3LYP, 6-31+G**)	G(UB3LYP, 6-31+G**)	E(UB3LYP,6-311+G(2d,2p))	D-3	G
**$FeCl_3$_spin2**	-1504,092426	-1504,122850	-1504,197090	-0,009719	-1504,237233
**$FeCl_3$_spin4**	-1504,123932	-1504,155213	-1504,229317	-0,009860	-1504,270458
**$FeCl_3$_spin6**	-1504,155118	-1504,186274	-1504,259689	-0,009234	-1504,300080
**1a**	-865,710570	-865,415128	-865,916333	-0,076648	-865,697539
**1a'**	-865,709674	-865,413161	-865,915273	-0,077649	-865,696408
**1c**	-862,954579	-862,778257	-863,093606	-0,047083	-862,964367
**1d**	-539,987088	-539,804245	-540,120585	-0,043812	-539,987088
**2a**	-789,272566	-788,995019	-789,451655	-0,080317	-789,254426
**2c**	-786,522958	-786,364670	-786,635956	-0,047074	-786,524742
**2d**	-463,543693	-463,382711	-463,650899	-0,041016	-463,530934
**H_2O**	-76,441217	-76,438312	-76,468013	-0,000574	-76,465682
**A_{1a}_spin6**	-2369,884137	-2369,587627	-2370,208158	-0,113579	-2370,025227
**A'_{1a}_spin6**	-2369,884762	-2369,588614	-2370,208981	-0,113892	-2370,026726
**B'_{1a}-spin6**	-2369,890135	-2369,595496	-2370,214994	-0,105091	-2370,025446
**C_{1a}-spin6**	-2369,909157	-2369,620266	-2370,226976	-0,102913	-2370,040998
**C'_{1a}-spin6**	-2369,911714	-2369,621138	-2370,233226	-0,102356	-2370,045006
**$TS_{1a\text{-}A}$-spin6**	-2369,860071	-2369,571150	-2370,183892	-0,106921	-2370,001892
**$TS_{1a\text{-}A'}$-spin6**	-2369,859379	-2369,570518	-2370,183541	-0,106505	-2370,001186
**D_{1a}-spin6**	-2369,899706	-2369,608482	-2370,222731	-0,109396	-2370,040903
**$A_{1c\text{-}S(R)}$-spin6**	-2367,120570	-2366,948454	-2367,373658	-0,077014	-2367,278557
**$A_{1c\text{-}S(S)}$-spin6**	-2367,122175	-2366,950474	-2367,374925	-0,077481	-2367,280705
**B_{1c}-spin6**	-2367,122091	-2366,952398	-2367,377068	-0,068198	-2367,275573
**C_{1c}-spin6**	-2367,122248	-2366,951590	-2367,376344	-0,070558	-2367,276244
**TS_{1c}-spin6**	-2367,099016	-2366,934621	-2367,352812	-0,071486	-2367,259902
**D_{1c}-spin6**	-2367,118808	-2366,953140	-2367,374283	-0,072162	-2367,280778
**E_{1c}-spin6**	-2367,123553	-2366,957718	-2367,378184	-0,070685	-2367,283034
**A_{1d}-spin2**	-2044,121685	-2043,939240	-2044,366079	-0,073788	-2044,257421
**A_{1d}-spin4**	-2044,141206	-2043,960222	-2044,385384	-0,072990	-2044,277389
**A_{1d}-spin6**	-2044,165914	-2043,986467	-2044,410598	-0,071095	-2044,302245
**B_{1d}-spin2**	-2044,103036	-2043,925319	-2044,343671	-0,063995	-2044,229948
**B_{1d}-spin4**	-2044,131461	-2043,953654	-2044,377415	-0,063512	-2044,263120
**B_{1d}-spin6**	-2044,160688	-2043,985674	-2044,407157	-0,062591	-2044,294733
**C_{1d}-spin2**	-2044,097405	-2043,918491	-2044,341335	-0,068156	-2044,230577
**C_{1d}-spin4**	-2044,124872	-2043,947036	-2044,371621	-0,067533	-2044,261318
**C_{1d}-spin6**	-2044,157190	-2043,980128	-2044,403987	-0,066738	-2044,293663
**TS_{1d}-spin2**	-2044,076004	-2043,903358	-2044,315396	-0,067135	-2044,209886
**TS_{1d}-spin4**	-2044,102356	-2043,928879	-2044,348172	-0,066750	-2044,241445
**TS_{1c}-spin6**	-2044,133163	-2043,963453	-2044,379243	-0,064728	-2044,274261
**D_{1c}-spin2**	-2044,099825	-2043,923039	-2044,344817	-0,065998	-2044,099825
**D_{1c}-spin4**	-2044,120428	-2043,944435	-2044,365444	-0,067483	-2044,120428
**D_{1c}-spin6**	-2044,152785	-2043,978687	-2044,398155	-0,064848	-2044,288907
**E_{1c}-spin2**	-2044,084298	-2043,909196	-2044,324000	-0,064318	-2044,213217
**E_{1c}-spin4**	-2044,108444	-2043,934283	-2044,353140	-0,064334	-2044,243313
**E_{1c}-spin6**	-2044,139944	-2043,968705	-2044,384990	-0,064024	-2044,277775

$FeCl_3$-spin2

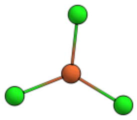

Fe	0,038443000	-0,054623000	0,204750000
Cl	2,182745000	0,061401000	-0,111847000
Cl	-1,035854000	1,796922000	-0,104460000
Cl	-1,211240000	-1,802644000	-0,095425000

FeCl3-spin4

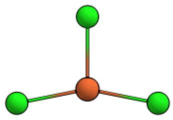

Fe	0.000000000	0.298608000	0.000000000
Cl	-0.000001000	-1.904821000	0.000000000
Cl	2.146048000	0.724061000	0.000000000
Cl	-2.146048000	0.724065000	0.000000000

FeCl3-spin6

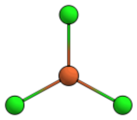

Fe	0.000001000	-0.018802000	0.000000000
Cl	-0.000005000	2.206745000	0.000000000
Cl	0.000001000	-1.088994000	1.963280000
Cl	0.000001000	-1.088994000	-1.963280000

1a

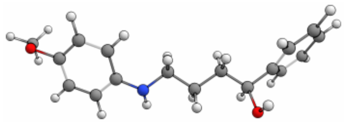

C	6.020440000	-1.642731000	-0.757788000
C	6.894606000	-1.489539000	0.323817000
C	6.607530000	-0.546123000	1.314147000
C	5.455021000	0.240552000	1.218838000
C	4.569965000	0.095409000	0.141353000
C	4.866778000	-0.859625000	-0.845072000
H	6.234330000	-2.374899000	-1.531242000
H	7.279381000	-0.419291000	2.158286000
H	5.240832000	0.976128000	1.990317000
H	4.197556000	-1.002344000	-1.689810000
C	3.333184000	0.977431000	0.046419000
H	3.273940000	1.577736000	0.960372000
C	2.027680000	0.179241000	-0.096997000
H	2.044523000	-0.382643000	-1.041001000

H	1.998394000	-0.567409000	0.707133000
C	0.770251000	1.054756000	-0.039684000
H	0.759108000	1.609315000	0.909663000
H	0.798404000	1.798645000	-0.843179000
C	-0.512988000	0.230362000	-0.158102000
H	-0.547956000	-0.525798000	0.645018000
H	-0.503744000	-0.316467000	-1.109261000
O	3.470722000	1.965028000	-0.998318000
H	3.589598000	1.510747000	-1.845017000
N	-1.688475000	1.095245000	-0.123247000
H	-1.579048000	1.904481000	0.475749000
C	-2.980144000	0.568720000	-0.117815000
C	-4.065975000	1.390893000	0.257993000
C	-3.259487000	-0.751808000	-0.520818000
C	-5.373835000	0.915724000	0.225374000
H	-3.875422000	2.416467000	0.564936000
C	-4.576195000	-1.225291000	-0.548577000
H	-2.456404000	-1.417188000	-0.816582000
C	-5.636647000	-0.399841000	-0.178520000
H	-6.195509000	1.568737000	0.505251000
H	-4.781481000	-2.244108000	-0.864207000
H	7.789833000	-2.100598000	0.393163000
O	-6.939675000	-0.873659000	-0.268663000
C	-7.558073000	-1.176457000	0.989133000
H	-7.615544000	-0.288131000	1.629346000
H	-8.567731000	-1.525377000	0.763990000
H	-7.004781000	-1.965695000	1.513437000

1a'

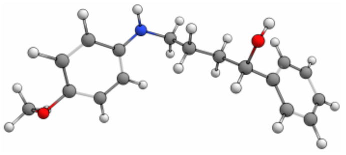

C	-6.137671000	-0.003309000	-0.964248000
C	-6.566011000	1.275105000	-0.591070000
C	-5.745340000	2.069718000	0.214263000
C	-4.506646000	1.584674000	0.645518000
C	-4.064821000	0.305783000	0.277699000
C	-4.896311000	-0.481241000	-0.536340000
H	-6.767761000	-0.627080000	-1.591936000
H	-6.068223000	3.064242000	0.508752000
H	-3.875672000	2.206106000	1.276127000
H	-4.580584000	-1.474477000	-0.845044000
C	-2.727015000	-0.211186000	0.786725000
H	-2.211616000	0.615770000	1.286580000
C	-1.812865000	-0.754248000	-0.322809000
H	-2.273495000	-1.646371000	-0.770081000
H	-1.760669000	-0.001310000	-1.120415000
C	-0.398617000	-1.091576000	0.164522000
H	0.078576000	-0.185775000	0.559748000
H	-0.453741000	-1.808095000	0.992336000
C	0.474514000	-1.686847000	-0.953563000

H	0.510081000	-1.004925000	-1.810705000
H	0.016156000	-2.611563000	-1.321892000
O	-2.912703000	-1.181480000	1.841827000
H	-3.448609000	-1.912054000	1.500468000
N	1.840899000	-2.019394000	-0.560781000
H	-7.529655000	1.647632000	-0.926173000
C	2.809241000	-1.055196000	-0.280950000
C	2.728107000	0.268659000	-0.758134000
C	3.953216000	-1.418556000	0.464118000
C	3.759509000	1.180414000	-0.513386000
H	1.863180000	0.598172000	-1.322689000
C	4.975347000	-0.504854000	0.709639000
H	4.034620000	-2.430640000	0.853501000
C	4.885831000	0.802319000	0.217462000
H	3.682521000	2.198918000	-0.883083000
H	5.841592000	-0.801053000	1.294452000
H	1.907002000	-2.842467000	0.024486000
O	5.875317000	1.736419000	0.501826000
C	7.019125000	1.671712000	-0.360793000
H	6.732641000	1.862998000	-1.402497000
H	7.709662000	2.447196000	-0.023411000
H	7.509228000	0.692794000	-0.294195000

1c

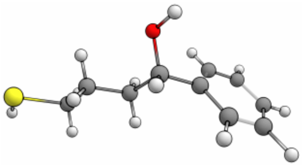

C	-3.654099000	0.437197000	1.194298000
C	-4.356680000	-0.576231000	0.532297000
C	-3.761568000	-1.238382000	-0.545345000
C	-2.471832000	-0.886909000	-0.957198000
C	-1.757514000	0.121623000	-0.295858000
C	-2.363839000	0.782264000	0.783907000
H	-4.111533000	0.959189000	2.030153000
H	-5.360223000	-0.842948000	0.850973000
H	-4.301642000	-2.021280000	-1.070279000
H	-2.017745000	-1.400337000	-1.801618000
H	-1.825268000	1.573701000	1.296952000
C	-0.341232000	0.467357000	-0.730277000
H	-0.179135000	0.054559000	-1.737170000
C	0.713818000	-0.126830000	0.212201000
H	0.576755000	0.314587000	1.208007000
H	0.513024000	-1.200742000	0.309018000
C	2.155541000	0.089993000	-0.270019000
H	2.274865000	-0.349431000	-1.269154000
H	2.352423000	1.162831000	-0.365752000
C	3.168876000	-0.538717000	0.688087000
H	3.000295000	-1.615286000	0.777654000
H	3.090272000	-0.088523000	1.681281000
O	-0.120987000	1.888336000	-0.763162000
H	-0.767524000	2.285108000	-1.363550000

S	4.887052000	-0.264150000	0.052598000
H	5.541666000	-0.899906000	1.044660000

1d

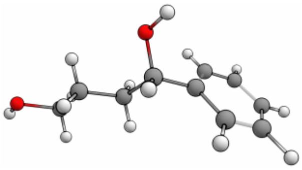

C	-3.171404000	0.504982000	1.180083000
C	-3.895703000	-0.507652000	0.540388000
C	-3.314371000	-1.207513000	-0.520721000
C	-2.016573000	-0.894373000	-0.938543000
C	-1.280678000	0.112971000	-0.299580000
C	-1.873479000	0.811674000	0.763703000
H	-3.618171000	1.056106000	2.002937000
H	-4.905326000	-0.744469000	0.863523000
H	-3.871279000	-1.990027000	-1.028399000
H	-1.573257000	-1.436972000	-1.770350000
H	-1.317529000	1.602824000	1.258210000
C	0.142856000	0.419047000	-0.740246000
H	0.298862000	-0.024246000	-1.735078000
C	1.185991000	-0.167237000	0.219796000
H	1.055036000	0.306772000	1.201613000
H	0.960273000	-1.232960000	0.349680000
C	2.630987000	0.003720000	-0.266311000
H	2.751013000	-0.474555000	-1.247453000
H	2.858563000	1.067044000	-0.395462000
C	3.637562000	-0.604850000	0.700420000
H	3.439576000	-1.678568000	0.830886000
H	3.555991000	-0.123352000	1.685395000
O	4.955438000	-0.408744000	0.163034000
H	5.599517000	-0.786900000	0.776393000
O	0.387816000	1.834940000	-0.812969000
H	-0.250426000	2.225131000	-1.426356000

2a

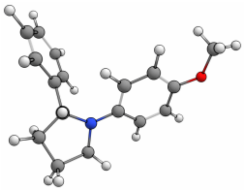

C	-2.570225000	-2.083348000	1.594515000
C	-3.079907000	-3.003157000	0.671130000
C	-3.095133000	-2.676727000	-0.688162000
C	-2.603083000	-1.439636000	-1.117159000
C	-2.096189000	-0.510042000	-0.198843000
C	-2.083820000	-0.846913000	1.161905000
H	-2.548216000	-2.330573000	2.652373000
H	-3.455506000	-3.965317000	1.007366000

H	-3.482895000	-3.384886000	-1.415219000
H	-2.609023000	-1.196799000	-2.177661000
H	-1.679320000	-0.138560000	1.879281000
C	-1.635830000	0.859311000	-0.683391000
H	-1.353646000	0.766637000	-1.746500000
C	-2.749791000	1.925372000	-0.553606000
H	-3.445131000	1.883616000	-1.396215000
H	-3.320360000	1.744875000	0.364501000
C	-1.978459000	3.244956000	-0.452772000
H	-1.661303000	3.575905000	-1.448310000
H	-2.563428000	4.050301000	0.000460000
C	-0.750720000	2.867009000	0.387753000
H	-0.935922000	3.023007000	1.460753000
H	0.129275000	3.460597000	0.115255000
N	-0.530411000	1.441322000	0.089014000
C	0.754025000	0.902215000	0.076883000
C	1.082225000	-0.254848000	-0.657761000
C	1.792458000	1.498740000	0.833856000
C	2.377806000	-0.788318000	-0.647815000
H	0.327963000	-0.762454000	-1.245931000
C	3.079966000	0.972010000	0.838438000
H	1.589188000	2.375646000	1.438425000
C	3.389175000	-0.176769000	0.097669000
H	2.573048000	-1.679843000	-1.232498000
H	3.860619000	1.445630000	1.426548000
O	4.691986000	-0.618787000	0.172543000
C	5.046996000	-1.783554000	-0.568004000
H	6.105455000	-1.954753000	-0.368604000
H	4.469513000	-2.656885000	-0.240738000
H	4.899702000	-1.632905000	-1.644555000

2c

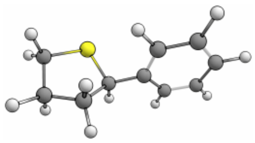

C	-2.595475000	-1.200579000	0.698161000
C	-3.553708000	-0.320885000	0.179256000
C	-3.138157000	0.845983000	-0.465880000
C	-1.773435000	1.131302000	-0.587860000
C	-0.804530000	0.261126000	-0.067349000
C	-1.235574000	-0.913076000	0.572799000
H	-2.909067000	-2.113226000	1.197177000
H	-4.611752000	-0.547101000	0.274825000
H	-3.871407000	1.534352000	-0.876502000
H	-1.457766000	2.041506000	-1.091836000
H	-0.501525000	-1.611264000	0.964729000
C	0.662131000	0.617459000	-0.181400000
H	0.744958000	1.554119000	-0.739814000
C	1.409947000	0.767344000	1.157760000
H	1.136200000	1.716033000	1.633098000
H	1.116786000	-0.040745000	1.838098000
C	2.909100000	0.680906000	0.858100000

H	3.231509000	1.572628000	0.307535000
H	3.505691000	0.622815000	1.775300000
C	3.118973000	-0.568695000	-0.007200000
H	3.151039000	-1.475742000	0.602484000
H	4.029358000	-0.518474000	-0.608394000
S	1.652521000	-0.658763000	-1.138439000

2d

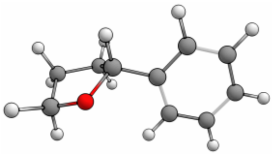

C	2.363486000	1.329961000	0.361160000
C	3.309885000	0.315417000	0.173298000
C	2.884770000	-0.965952000	-0.187544000
C	1.521220000	-1.228973000	-0.359380000
C	0.566270000	-0.222633000	-0.163097000
C	1.002868000	1.061922000	0.195832000
H	2.686687000	2.330627000	0.634540000
H	4.368037000	0.525033000	0.300860000
H	3.611325000	-1.758264000	-0.344242000
H	1.197446000	-2.225191000	-0.651551000
H	0.272979000	1.854155000	0.333226000
C	-0.907684000	-0.538795000	-0.299815000
H	-1.013061000	-1.476211000	-0.866133000
C	-1.678617000	-0.662331000	1.027070000
H	-1.565470000	-1.652647000	1.476642000
H	-1.312651000	0.083174000	1.742381000
C	-3.115359000	-0.342384000	0.593806000
H	-3.583635000	-1.223903000	0.141946000
H	-3.750230000	-0.004816000	1.417844000
C	-2.894140000	0.750966000	-0.458570000
H	-2.897914000	1.752047000	-0.007491000
H	-3.634181000	0.730329000	-1.265286000
O	-1.586941000	0.510309000	-1.026162000

H_2O

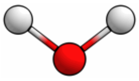

O	0.000000000	0.117541000	0.000000000
H	0.767250000	-0.470164000	0.000000000
H	-0.767250000	-0.470167000	0.000000000

A_{1a}-spin6

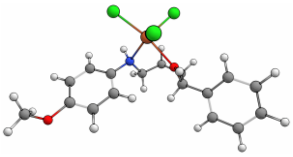

C	-4.879042000	-3.299508000	-0.350747000
C	-5.822751000	-2.412974000	-0.878131000
C	-5.548171000	-1.040788000	-0.904910000
C	-4.338929000	-0.555627000	-0.400798000
C	-3.386308000	-1.441093000	0.127701000
C	-3.663896000	-2.814947000	0.142957000
H	-5.082386000	-4.366290000	-0.331155000
H	-6.763863000	-2.788169000	-1.269612000
H	-6.275710000	-0.348112000	-1.317890000
H	-4.131640000	0.510508000	-0.424072000
H	-2.928438000	-3.508882000	0.542717000
C	-2.080167000	-0.933261000	0.705633000
H	-1.398577000	-1.785462000	0.803282000
C	-2.257678000	-0.256891000	2.067237000
H	-2.620188000	-1.042153000	2.740283000
H	-3.058192000	0.486670000	1.996317000
C	-1.004413000	0.411310000	2.683785000
H	-1.130564000	0.412677000	3.770812000
H	-0.969788000	1.466352000	2.396413000
C	0.344559000	-0.240131000	2.351859000
H	0.996358000	-0.250279000	3.231547000
H	0.232722000	-1.277113000	2.035839000
O	-1.455822000	0.015321000	-0.212229000
H	-1.584434000	-0.268976000	-1.131847000
N	1.102285000	0.484941000	1.264350000
H	1.532736000	1.300340000	1.703076000
C	2.176677000	-0.307061000	0.695886000
C	1.888692000	-1.428907000	-0.097857000
C	3.507486000	0.018159000	0.966401000
C	2.919372000	-2.220007000	-0.587668000
H	0.861044000	-1.685008000	-0.333452000
C	4.551209000	-0.770388000	0.476406000
H	3.741764000	0.887952000	1.573531000
C	4.258903000	-1.902653000	-0.297736000
H	2.706750000	-3.090419000	-1.199598000
H	5.572268000	-0.495299000	0.709580000
O	5.194265000	-2.748679000	-0.808609000
C	6.575015000	-2.496740000	-0.527606000
H	6.767107000	-2.521317000	0.550880000
H	7.126328000	-3.300618000	-1.014913000
H	6.891238000	-1.532257000	-0.939712000
Fe	0.023388000	1.657572000	-0.305988000
Cl	1.871133000	3.052213000	-0.354211000
Cl	-1.542514000	3.189846000	0.351409000
Cl	-0.220354000	1.329723000	-2.576878000

A'$_{1a}$-spin6

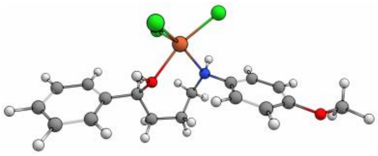

C	-6.062410000	-0.606616000	-0.264001000
C	-6.312781000	-1.403522000	0.856837000
C	-5.250653000	-2.041961000	1.507132000
C	-3.945541000	-1.891116000	1.033042000
C	-3.684847000	-1.088025000	-0.090495000
C	-4.753939000	-0.443666000	-0.728911000
H	-6.881306000	-0.105522000	-0.771801000
H	-7.327908000	-1.526115000	1.223027000
H	-5.438786000	-2.661290000	2.379248000
H	-3.131151000	-2.401702000	1.540699000
H	-4.562440000	0.186340000	-1.593289000
C	-2.278297000	-0.917746000	-0.622026000
H	-2.303202000	-0.191743000	-1.437331000
C	-1.637539000	-2.221290000	-1.103969000
H	-2.143804000	-2.485296000	-2.040678000
H	-1.855675000	-3.027193000	-0.395107000
C	-0.113876000	-2.172077000	-1.321970000
H	0.164865000	-3.067956000	-1.885962000
H	0.390554000	-2.266439000	-0.359105000
C	0.433648000	-0.945390000	-2.069944000
H	1.190903000	-1.259875000	-2.796077000
H	-0.349945000	-0.424904000	-2.627315000
O	-1.408682000	-0.320623000	0.412452000
H	-1.944581000	-0.048170000	1.176828000
N	1.093955000	0.094763000	-1.190202000
H	1.349454000	0.851768000	-1.827567000
C	2.340565000	-0.362852000	-0.584185000
C	3.548156000	-0.169275000	-1.258227000
C	2.352853000	-0.993255000	0.667871000
C	4.759518000	-0.590260000	-0.701834000
H	3.557955000	0.322536000	-2.227467000
C	3.550319000	-1.425400000	1.226869000
H	1.430401000	-1.134770000	1.221776000
C	4.763882000	-1.222649000	0.549857000
H	5.678522000	-0.416077000	-1.247839000
H	3.563932000	-1.905797000	2.199762000
O	5.883542000	-1.666380000	1.187192000
C	7.156156000	-1.444550000	0.572793000
H	7.892564000	-1.856702000	1.262595000
H	7.225834000	-1.963048000	-0.389981000
H	7.343682000	-0.374081000	0.432498000
Fe	-0.136173000	1.441451000	0.117761000
Cl	-1.014420000	1.994520000	2.207759000
Cl	-1.404407000	2.373058000	-1.519228000
Cl	1.656638000	2.876474000	0.171617000

B’$_{1a}$-spin6

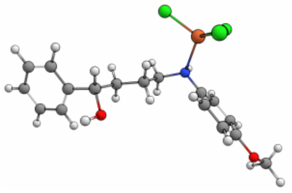

C	6.824263000	1.864208000	-0.826597000
C	7.649235000	0.752277000	-0.620329000
C	7.140685000	-0.373477000	0.034981000
C	5.814744000	-0.385072000	0.481201000
C	4.979225000	0.721087000	0.272094000
C	5.498292000	1.847382000	-0.385244000
H	7.213476000	2.744049000	-1.331675000
H	8.679898000	0.765784000	-0.963373000
H	7.775478000	-1.238557000	0.205522000
H	5.427565000	-1.260777000	0.997335000
H	4.862473000	2.713911000	-0.542404000
C	3.525913000	0.679561000	0.721166000
H	3.416212000	-0.121081000	1.467560000
C	2.580519000	0.384265000	-0.450252000
H	2.688154000	1.185831000	-1.192660000
H	2.919232000	-0.542212000	-0.929088000
C	1.108358000	0.248926000	-0.030216000
H	1.015655000	-0.540737000	0.726626000
H	0.784494000	1.185110000	0.428963000
C	0.253635000	-0.074685000	-1.253607000
H	0.640489000	-0.966143000	-1.753422000
H	0.286682000	0.757991000	-1.966515000
O	3.102240000	1.925412000	1.298800000
H	3.675728000	2.125868000	2.051620000
N	-1.207915000	-0.370234000	-0.993913000
H	-1.576061000	-0.691860000	-1.891897000
C	-2.045840000	0.741545000	-0.580951000
C	-1.915172000	1.330782000	0.687344000
C	-3.024142000	1.218557000	-1.459615000
C	-2.733243000	2.390913000	1.050001000
H	-1.199927000	0.947315000	1.403463000
C	-3.855584000	2.281103000	-1.100892000
H	-3.143250000	0.765255000	-2.440323000
C	-3.710396000	2.875999000	0.161631000
H	-2.641637000	2.847448000	2.029915000
H	-4.600971000	2.627882000	-1.805637000
Fe	-1.644012000	-2.164887000	0.114665000
Cl	-3.452800000	-2.868290000	-1.043518000
Cl	0.079744000	-3.553538000	-0.287390000
Cl	-2.056783000	-1.836795000	2.299715000
O	-4.463776000	3.909926000	0.617726000
C	-5.507224000	4.426101000	-0.216005000
H	-5.973739000	5.225601000	0.359006000
H	-5.100956000	4.833206000	-1.148256000
H	-6.249311000	3.651881000	-0.438814000

C_{1a}-spin6

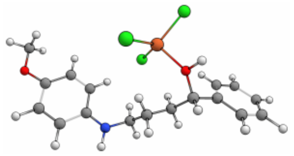

C	-5.664095000	-0.459742000	1.365539000
C	-6.621809000	-1.220946000	0.684386000
C	-6.261888000	-1.909930000	-0.477162000
C	-4.948619000	-1.837521000	-0.954503000
C	-3.982747000	-1.084237000	-0.273673000
C	-4.352228000	-0.393015000	0.891482000
H	-5.939553000	0.082779000	2.265371000
H	-7.641854000	-1.270729000	1.054405000
H	-7.000729000	-2.496437000	-1.015749000
H	-4.674559000	-2.368686000	-1.862934000
H	-3.617745000	0.205539000	1.422157000
C	-2.555543000	-1.045618000	-0.786480000
H	-2.519346000	-1.518883000	-1.775100000
C	-1.567064000	-1.744445000	0.149865000
H	-1.472973000	-1.146010000	1.063508000
H	-2.015808000	-2.701112000	0.441086000
C	-0.181717000	-2.000533000	-0.463341000
H	-0.264691000	-2.720287000	-1.286955000
H	0.245302000	-1.083627000	-0.881296000
C	0.773406000	-2.554882000	0.609109000
H	0.387861000	-3.494683000	1.015334000
H	0.864659000	-1.849349000	1.436947000
O	-2.117229000	0.338770000	-0.958820000
H	-2.761588000	0.806100000	-1.511654000
N	2.106341000	-2.844541000	0.080143000
H	2.217612000	-3.747373000	-0.370813000
C	3.182056000	-2.035312000	0.075995000
C	4.406120000	-2.518036000	-0.497653000
C	3.149641000	-0.716387000	0.628581000
C	5.525822000	-1.731968000	-0.504469000
H	4.435711000	-3.516630000	-0.922999000
C	4.279720000	0.070076000	0.616279000
H	2.235305000	-0.311862000	1.049137000
C	5.484849000	-0.422827000	0.055289000
H	6.458379000	-2.083979000	-0.931478000
H	4.229739000	1.068476000	1.031480000
O	6.627792000	0.259823000	0.001487000
C	6.703314000	1.600874000	0.532196000
H	7.733745000	1.911724000	0.371422000
H	6.022409000	2.262086000	-0.009709000
H	6.473640000	1.600296000	1.600793000
Fe	-0.702850000	1.785550000	-0.151564000
Cl	0.139164000	1.180609000	1.984855000
Cl	0.849884000	1.711182000	-1.927598000
Cl	-2.175261000	3.643390000	-0.119214000

C’$_{1a}$-spin6

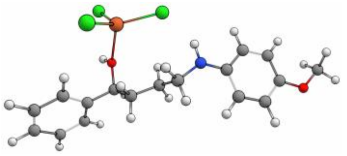

C	5.481846000	2.104074000	0.922983000
C	5.929687000	3.195271000	0.168733000
C	5.169447000	3.648178000	-0.913303000
C	3.967158000	3.010883000	-1.238166000
C	3.509114000	1.922872000	-0.482480000
C	4.278232000	1.471551000	0.602167000
H	6.071404000	1.744015000	1.761432000
H	6.866598000	3.684179000	0.420142000
H	5.513047000	4.489359000	-1.508642000
H	3.383801000	3.361387000	-2.086328000
H	3.939684000	0.621879000	1.188096000
C	2.183225000	1.273539000	-0.828285000
H	1.856663000	1.632203000	-1.812333000
C	1.083637000	1.548787000	0.202189000
H	1.366630000	1.048440000	1.136527000
H	1.066344000	2.623792000	0.409274000
C	-0.295670000	1.063707000	-0.274623000
H	-0.740300000	1.786406000	-0.968326000
H	-0.178804000	0.124260000	-0.821901000
C	-1.262062000	0.810319000	0.897174000
H	-1.621007000	1.737419000	1.342922000
H	-0.749599000	0.241577000	1.680618000
Fe	1.773229000	-2.026301000	0.103906000
Cl	2.434518000	-1.637423000	2.312284000
Cl	-0.510191000	-2.670847000	-0.232546000
Cl	3.163205000	-3.129385000	-1.518170000
O	2.318789000	-0.176784000	-0.924559000
H	2.987975000	-0.417471000	-1.585250000
N	-2.388361000	-0.011213000	0.462458000
C	-3.678072000	0.331742000	0.312642000
C	-4.177225000	1.642401000	0.604881000
C	-4.596455000	-0.662566000	-0.159398000
C	-5.508193000	1.923328000	0.434286000
H	-3.510949000	2.418925000	0.957997000
C	-5.930955000	-0.375082000	-0.329385000
H	-4.224283000	-1.657403000	-0.382924000
C	-6.408630000	0.926270000	-0.033365000
H	-5.901344000	2.910765000	0.650242000
H	-6.600956000	-1.146418000	-0.687152000
H	-2.112245000	-0.964777000	0.203785000
O	-7.678861000	1.314398000	-0.160865000
C	-8.678273000	0.386264000	-0.631191000
H	-9.610423000	0.948019000	-0.632718000
H	-8.440104000	0.052517000	-1.644675000
H	-8.755900000	-0.466750000	0.048027000

$TS_{1a\text{-}A'}$-spin6

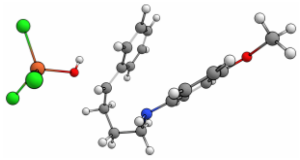

C	1.473079000	-1.597753000	2.828744000
C	1.808901000	-2.598152000	1.909318000
C	1.393672000	-2.494028000	0.575443000
C	0.641313000	-1.397689000	0.167291000
C	0.292719000	-0.379629000	1.083724000
C	0.723283000	-0.495136000	2.422839000
H	1.796423000	-1.677828000	3.861943000
H	2.393538000	-3.455177000	2.230162000
H	1.656388000	-3.267202000	-0.139738000
H	0.312290000	-1.317879000	-0.864530000
H	0.467419000	0.266548000	3.150763000
C	-0.525966000	0.726881000	0.611715000
H	-0.727481000	0.742122000	-0.452987000
C	-0.789519000	2.004797000	1.353474000
H	-1.870812000	2.162286000	1.365354000
H	-0.464915000	1.933833000	2.394428000
C	-0.143532000	3.241012000	0.684134000
H	-0.601597000	3.409325000	-0.298958000
H	-0.371931000	4.121859000	1.292874000
C	1.367206000	3.095224000	0.530805000
H	1.816718000	2.905980000	1.508204000
H	1.808408000	4.024680000	0.141370000
O	-2.346259000	-0.130852000	0.856304000
H	-2.217816000	-0.867860000	1.474797000
Fe	-3.764410000	-0.324214000	-0.380425000
Cl	-4.494161000	-2.463397000	-0.245103000
Cl	-2.865763000	0.154900000	-2.414556000
Cl	-5.385675000	1.191940000	0.134633000
N	1.641586000	1.961140000	-0.350747000
H	1.269842000	2.114140000	-1.282754000
C	2.886814000	1.309588000	-0.394270000
C	3.278836000	0.663534000	-1.579426000
C	3.740255000	1.215051000	0.723314000
C	4.476070000	-0.051351000	-1.662282000
H	2.639180000	0.721948000	-2.456924000
C	4.934835000	0.505420000	0.645147000
H	3.480977000	1.690508000	1.662188000
C	5.315226000	-0.135900000	-0.543189000
H	4.737352000	-0.528968000	-2.599040000
H	5.588326000	0.438132000	1.509552000
O	6.509385000	-0.806427000	-0.512367000
C	6.935850000	-1.485276000	-1.693843000
H	7.892903000	-1.944248000	-1.444440000
H	6.221329000	-2.265034000	-1.983009000
H	7.072938000	-0.785464000	-2.526647000

$TS_{1a\text{-}B'}$-spin6

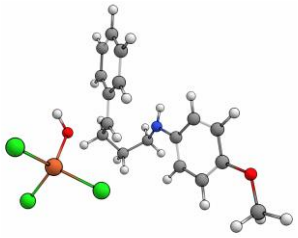

C	0.929307000	4.727036000	-0.731255000
C	0.471419000	4.474120000	-2.030242000
C	0.048267000	3.187819000	-2.388983000
C	0.083931000	2.159796000	-1.451655000
C	0.533962000	2.402320000	-0.133785000
C	0.962657000	3.701775000	0.212015000
H	1.261297000	5.723256000	-0.456038000
H	0.446929000	5.276520000	-2.761540000
H	-0.303444000	2.990124000	-3.396689000
H	-0.233832000	1.159137000	-1.728963000
H	1.322670000	3.914376000	1.212648000
C	0.556240000	1.288001000	0.804861000
H	0.094933000	0.372382000	0.456355000
C	0.805581000	1.387885000	2.281811000
H	1.747431000	0.873840000	2.493022000
H	0.935865000	2.427882000	2.592560000
C	-0.306112000	0.717891000	3.111244000
H	-0.377489000	-0.340164000	2.835348000
H	-0.032611000	0.751988000	4.171386000
C	-1.652878000	1.421931000	2.904800000
H	-1.597352000	2.433880000	3.317776000
H	-2.451989000	0.895530000	3.441495000
O	2.332313000	0.459132000	0.240531000
H	2.801947000	1.122503000	-0.289819000
Fe	2.687448000	-1.349144000	-0.184743000
Cl	3.786846000	-1.361828000	-2.167581000
Cl	0.682475000	-2.388011000	-0.383993000
Cl	3.913328000	-2.313963000	1.468028000
N	-1.974453000	1.547427000	1.479972000
H	-2.160192000	2.499724000	1.191383000
C	-2.789334000	0.618800000	0.806957000
C	-2.958343000	-0.702455000	1.246128000
C	-3.434539000	1.011271000	-0.385441000
C	-3.748463000	-1.607325000	0.525363000
H	-2.476554000	-1.050346000	2.152921000
C	-4.215630000	0.116219000	-1.104410000
H	-3.325008000	2.031644000	-0.743939000
C	-4.380288000	-1.204918000	-0.656086000
H	-3.851773000	-2.618628000	0.899945000
H	-4.711821000	0.428195000	-2.018279000
O	-5.169341000	-2.010477000	-1.434328000
C	-5.356098000	-3.367211000	-1.031128000
H	-5.844403000	-3.426385000	-0.051122000
H	-6.002117000	-3.816079000	-1.786132000
H	-4.401847000	-3.905959000	-0.999310000

D$_{1a}$-spin6

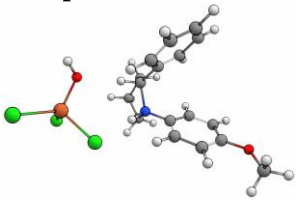

C	-3.148452000	3.415252000	-0.447218000
C	-2.823471000	3.435459000	-1.806901000
C	-1.676393000	2.772623000	-2.255021000
C	-0.863618000	2.092830000	-1.345396000
C	-1.187653000	2.054263000	0.021274000
C	-2.336903000	2.729072000	0.460450000
H	-4.032657000	3.935152000	-0.090307000
H	-3.455218000	3.969853000	-2.510427000
H	-1.410130000	2.790927000	-3.307717000
H	0.036304000	1.594148000	-1.695195000
H	-2.606454000	2.732464000	1.511384000
C	-0.259332000	1.325643000	0.960258000
H	0.747664000	1.347392000	0.533299000
C	-0.168033000	1.753914000	2.422802000
H	0.368655000	2.702329000	2.505229000
H	-1.156818000	1.888739000	2.873401000
C	0.576695000	0.587532000	3.083305000
H	1.640532000	0.621738000	2.829568000
H	0.486978000	0.583146000	4.172088000
C	-0.084842000	-0.652831000	2.478696000
H	-0.971840000	-0.953227000	3.033312000
H	0.574577000	-1.514801000	2.373230000
O	2.824937000	1.407065000	-0.345844000
H	3.076953000	2.070638000	-1.004832000
Fe	3.559526000	-0.259586000	-0.414119000
Cl	4.988224000	-0.481990000	-2.167321000
Cl	1.778797000	-1.755531000	-0.719774000
Cl	4.505945000	-0.813705000	1.590239000
N	-0.542680000	-0.241222000	1.070540000
H	0.119334000	-0.681222000	0.410779000
C	-1.884139000	-0.720712000	0.697877000
C	-2.032975000	-1.436969000	-0.485783000
C	-2.994589000	-0.456080000	1.507728000
C	-3.292701000	-1.903324000	-0.874698000
H	-1.172913000	-1.643182000	-1.115214000
C	-4.248462000	-0.913682000	1.126001000
H	-2.895673000	0.104064000	2.431134000
C	-4.408054000	-1.640369000	-0.068096000
H	-3.382748000	-2.463709000	-1.796628000
H	-5.119561000	-0.716040000	1.741605000
O	-5.675138000	-2.043498000	-0.349730000
C	-5.910564000	-2.784695000	-1.552258000
H	-6.981305000	-2.986516000	-1.568124000
H	-5.633908000	-2.198478000	-2.435301000
H	-5.358584000	-3.730863000	-1.544938000

$A_{1c\text{-}S(\boldsymbol{R})}$-spin6

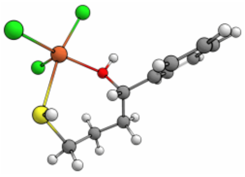

C	-4.379244000	-0.775437000	1.422547000
C	-5.134808000	-0.958045000	0.260524000
C	-4.645715000	-0.497981000	-0.966793000
C	-3.407698000	0.145972000	-1.032110000
C	-2.639083000	0.327828000	0.130444000
C	-3.134538000	-0.143472000	1.354937000
H	-4.751830000	-1.132752000	2.377940000
H	-6.098482000	-1.456359000	0.309729000
H	-5.227892000	-0.637719000	-1.872743000
H	-3.045624000	0.501803000	-1.993051000
H	-2.545195000	-0.014009000	2.258915000
C	-1.280162000	0.990230000	0.077659000
H	-0.882446000	1.035840000	1.091854000
C	-1.261288000	2.378353000	-0.566769000
H	-1.808708000	3.050956000	0.105952000
H	-1.827325000	2.348123000	-1.503837000
C	0.128043000	2.956952000	-0.874745000
H	-0.027747000	3.929485000	-1.358395000
H	0.640103000	2.331352000	-1.612767000
C	1.055526000	3.223301000	0.314400000
H	1.879200000	3.866351000	-0.001989000
H	0.536945000	3.724614000	1.135458000
O	-0.338921000	0.116953000	-0.667118000
H	-0.831628000	-0.604214000	-1.095073000
Fe	1.495094000	-0.723480000	-0.070694000
Cl	0.544077000	-2.726070000	-0.782313000
Cl	2.552249000	-1.406629000	1.824961000
Cl	3.025052000	-0.251722000	-1.656216000
S	1.924239000	1.749230000	1.055089000
H	1.172087000	1.592650000	2.161668000

$A_{1c\text{-}S(\boldsymbol{S})}$-spin6

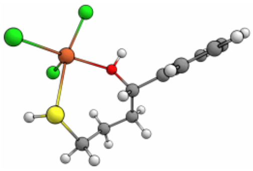

C	-4.519837000	-0.688281000	1.462873000
C	-5.234490000	-0.981501000	0.297611000
C	-4.692943000	-0.656609000	-0.950716000
C	-3.444029000	-0.036331000	-1.034161000
C	-2.717306000	0.258138000	0.132270000
C	-3.264641000	-0.079344000	1.378845000
H	-4.933263000	-0.940147000	2.434934000
H	-6.207034000	-1.460670000	0.360701000

H	-5.242723000	-0.883109000	-1.859385000
H	-3.040971000	0.214165000	-2.011548000
H	-2.708529000	0.137745000	2.287091000
C	-1.351475000	0.905783000	0.063178000
H	-0.999249000	1.070432000	1.084325000
C	-1.301124000	2.207307000	-0.738933000
H	-1.941487000	2.926792000	-0.212612000
H	-1.755966000	2.043745000	-1.721396000
C	0.095475000	2.814898000	-0.948561000
H	-0.038341000	3.752524000	-1.501990000
H	0.704704000	2.166857000	-1.585583000
C	0.864321000	3.165509000	0.328350000
H	1.674639000	3.864417000	0.116283000
H	0.210971000	3.629103000	1.072702000
O	-0.390215000	-0.052329000	-0.532796000
H	-0.860235000	-0.843304000	-0.847194000
Fe	1.555952000	-0.684489000	-0.030587000
Cl	0.779336000	-2.786440000	-0.642248000
Cl	2.973154000	-1.243288000	1.662822000
Cl	2.815468000	-0.008734000	-1.775122000
S	1.664400000	1.739820000	1.223294000
H	2.915065000	1.896112000	0.751370000

B_{1c}-spin6

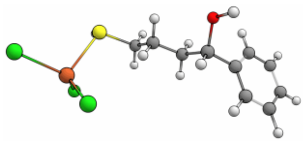

C	-6.500400000	0.190864000	0.895776000
C	-6.819284000	-1.130934000	0.562435000
C	-5.925777000	-1.885500000	-0.203845000
C	-4.721233000	-1.318948000	-0.634477000
C	-4.390957000	0.001859000	-0.300248000
C	-5.293973000	0.752437000	0.468902000
H	-7.192088000	0.784195000	1.487513000
H	-7.758313000	-1.565993000	0.892649000
H	-6.168056000	-2.909549000	-0.474039000
H	-4.034197000	-1.907182000	-1.238674000
H	-5.054584000	1.781095000	0.722890000
C	-3.065784000	0.595153000	-0.752861000
H	-2.659188000	-0.041804000	-1.552159000
C	-2.037441000	0.659226000	0.385190000
H	-2.417099000	1.338593000	1.159086000
H	-1.964635000	-0.336760000	0.837249000
C	-0.651960000	1.116230000	-0.097137000
H	-0.279571000	0.406880000	-0.845545000
H	-0.736343000	2.093291000	-0.582252000
C	0.332876000	1.202522000	1.067837000
H	0.447320000	0.250173000	1.588835000
H	0.036731000	1.968552000	1.789135000
O	-3.217270000	1.936260000	-1.248779000
H	-3.851297000	1.924672000	-1.979219000
Fe	3.310943000	-0.414502000	-0.051930000

Cl	1.880325000	-1.646753000	-1.251631000
Cl	5.002231000	0.479607000	-1.204478000
Cl	3.770457000	-1.144531000	2.012330000
S	2.001744000	1.734129000	0.443550000
H	2.599424000	1.955811000	1.632961000

C_{1c}-spin6

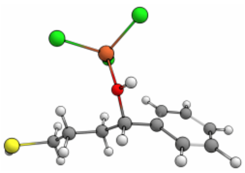

C	-3.797578000	-1.143979000	1.282785000
C	-4.701771000	-1.816817000	0.451816000
C	-4.269330000	-2.331114000	-0.773210000
C	-2.936344000	-2.171572000	-1.165424000
C	-2.023707000	-1.502496000	-0.337316000
C	-2.466476000	-0.987641000	0.892183000
H	-4.129309000	-0.741991000	2.235540000
H	-5.736345000	-1.938105000	0.759273000
H	-4.964673000	-2.853450000	-1.423605000
H	-2.603743000	-2.573442000	-2.119322000
H	-1.777637000	-0.463721000	1.548401000
C	-0.586476000	-1.364358000	-0.784318000
H	-0.451454000	-1.884504000	-1.737434000
C	0.458266000	-1.861550000	0.209427000
H	0.401326000	-1.283457000	1.140152000
H	0.165603000	-2.885760000	0.469688000
C	1.891122000	-1.859996000	-0.346050000
H	1.920794000	-2.455769000	-1.267260000
H	2.191277000	-0.841952000	-0.614973000
C	2.881923000	-2.436144000	0.667757000
H	2.605127000	-3.458563000	0.938797000
H	2.903115000	-1.826435000	1.574864000
O	-0.285147000	0.065730000	-1.107764000
H	-0.800089000	0.324090000	-1.890304000
Fe	0.231278000	1.745223000	-0.037106000
Cl	0.233146000	1.806046000	2.201349000
Cl	2.278120000	2.269119000	-0.807876000
Cl	-1.254724000	3.228427000	-0.821628000
S	4.579213000	-2.459164000	-0.068275000
H	5.217561000	-2.979014000	0.998184000

TS_{1c}-spin6

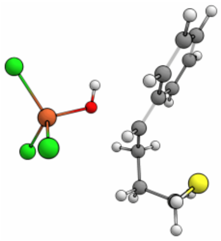

C	3.490717000	2.584599000	-1.261130000
C	3.469884000	3.324508000	-0.070929000
C	2.724153000	2.880528000	1.030322000
C	1.999148000	1.699368000	0.939612000
C	2.017330000	0.933468000	-0.252300000
C	2.774004000	1.396516000	-1.354798000
H	4.067221000	2.937198000	-2.110138000
H	4.035449000	4.248900000	-0.001797000
H	2.712113000	3.457748000	1.949027000
H	1.415777000	1.349863000	1.786222000
H	2.794525000	0.833982000	-2.281328000
C	1.244641000	-0.280841000	-0.296144000
H	0.737280000	-0.556247000	0.621078000
C	1.295412000	-1.313183000	-1.375090000
H	0.380939000	-1.182949000	-1.964748000
H	2.132985000	-1.135502000	-2.054815000
C	1.319720000	-2.768631000	-0.865456000
H	0.515777000	-2.921943000	-0.136517000
H	1.101946000	-3.427140000	-1.714580000
C	2.662232000	-3.195527000	-0.270132000
H	3.468984000	-3.070537000	-0.998580000
H	2.641076000	-4.247242000	0.024768000
O	-0.683433000	0.576488000	-0.786108000
H	-0.532406000	1.506321000	-1.018512000
Fe	-2.339512000	0.164448000	-0.007259000
Cl	-3.497520000	2.086836000	0.309880000
Cl	-1.893267000	-0.834326000	1.993113000
Cl	-3.456761000	-1.293888000	-1.350956000
S	3.188237000	-2.183726000	1.175900000
H	2.236182000	-2.601793000	2.034771000

D_{1c}-spin6

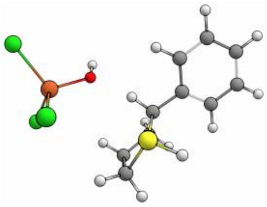

C	-5.455826000	-0.640638000	-0.392669000
C	-5.313963000	-2.006027000	-0.121319000
C	-4.041909000	-2.541739000	0.100410000
C	-2.917233000	-1.714670000	0.051224000
C	-3.049714000	-0.343426000	-0.222082000
C	-4.331890000	0.186994000	-0.440961000
H	-6.441076000	-0.219615000	-0.569091000
H	-6.189633000	-2.647273000	-0.085118000
H	-3.923002000	-3.600549000	0.309723000
H	-1.929026000	-2.133207000	0.220411000
H	-4.464095000	1.243310000	-0.656061000
C	-1.807019000	0.504587000	-0.281714000
H	-0.915730000	-0.131197000	-0.305873000
C	-1.682090000	1.593040000	-1.352931000
H	-1.509579000	1.105365000	-2.317894000

H	-2.603187000	2.178932000	-1.436354000
C	-0.501694000	2.486743000	-0.959651000
H	0.437130000	1.939680000	-1.082262000
H	-0.449581000	3.387578000	-1.577388000
C	-0.667419000	2.894251000	0.516133000
H	-1.302711000	3.768898000	0.657868000
H	0.276631000	3.028283000	1.043150000
O	0.942970000	-1.028141000	-0.833794000
H	0.975219000	-1.681568000	-1.548266000
Fe	2.521955000	-0.491201000	-0.080729000
Cl	3.859103000	-2.311159000	0.268615000
Cl	1.984894000	0.552641000	1.891802000
Cl	3.616217000	0.979435000	-1.471000000
S	-1.532382000	1.467529000	1.348915000
H	-2.768910000	2.006953000	1.435125000

E_{1c}-spin6

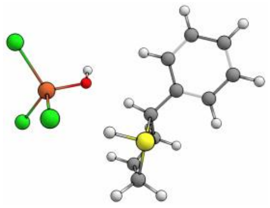

C	-5.294166000	-1.049119000	0.018861000
C	-4.942618000	-2.403320000	-0.028009000
C	-3.603104000	-2.767097000	-0.189805000
C	-2.618520000	-1.780908000	-0.301261000
C	-2.962755000	-0.420763000	-0.260157000
C	-4.311619000	-0.064292000	-0.096721000
H	-6.333016000	-0.759883000	0.147164000
H	-5.708788000	-3.167695000	0.062275000
H	-3.322026000	-3.815390000	-0.226645000
H	-1.576885000	-2.064688000	-0.423858000
H	-4.604040000	0.981188000	-0.053342000
C	-1.870122000	0.605943000	-0.401967000
H	-0.915216000	0.113627000	-0.606394000
C	-2.086522000	1.765589000	-1.388210000
H	-1.910446000	1.387826000	-2.400247000
H	-3.119932000	2.124589000	-1.345330000
C	-1.116719000	2.894259000	-1.024555000
H	-0.084901000	2.597818000	-1.237548000
H	-1.327313000	3.807647000	-1.588900000
C	-1.285405000	3.167241000	0.474533000
H	-2.188176000	3.738206000	0.699489000
H	-0.428177000	3.639440000	0.956392000
O	1.045909000	-0.811378000	-1.092478000
H	1.084061000	-1.461596000	-1.809741000
Fe	2.555659000	-0.448986000	-0.134041000
Cl	3.630681000	-2.385726000	0.415150000
Cl	1.834752000	0.600529000	1.817473000
Cl	4.014592000	0.962677000	-1.200328000
S	-1.547514000	1.512137000	1.265384000
H	-0.230467000	1.147059000	1.440147000

A$_{1d}$-spin2

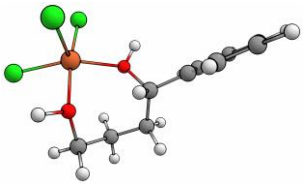

C	-4.632000000	-0.579331000	1.235418000
C	-5.242542000	-0.848009000	0.006801000
C	-4.556656000	-0.584493000	-1.184072000
C	-3.269172000	-0.044702000	-1.146438000
C	-2.646332000	0.221996000	0.085187000
C	-3.336579000	-0.055588000	1.273660000
H	-5.158737000	-0.783150000	2.162911000
H	-6.246658000	-1.260608000	-0.023925000
H	-5.025372000	-0.793115000	-2.141168000
H	-2.753569000	0.168250000	-2.079406000
H	-2.860756000	0.141271000	2.230530000
C	-1.257270000	0.811873000	0.147485000
H	-0.921022000	0.848118000	1.183673000
C	-1.134863000	2.199142000	-0.505417000
H	-1.604718000	2.913155000	0.181790000
H	-1.725272000	2.215235000	-1.427127000
C	0.291905000	2.655790000	-0.856623000
H	0.232613000	3.660801000	-1.289844000
H	0.699933000	2.016525000	-1.646398000
C	1.282084000	2.738598000	0.291965000
H	2.268375000	3.047275000	-0.065277000
H	0.941876000	3.425493000	1.072841000
O	-0.302288000	-0.094558000	-0.540266000
H	-0.718737000	-0.929121000	-0.813430000
O	1.403038000	1.423601000	0.928433000
H	2.086911000	1.448733000	1.616841000
Fe	1.603248000	-0.322998000	-0.087775000
Cl	1.627278000	-2.133684000	-1.395523000
Cl	1.481373000	-1.346230000	1.913330000
Cl	3.791539000	0.100802000	-0.275043000

A$_{1d}$-spin4

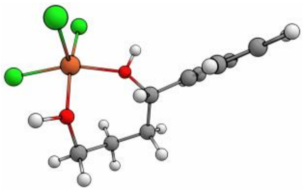

C	-4.619158000	-0.647118000	1.196074000
C	-5.238616000	-0.854617000	-0.040006000
C	-4.563262000	-0.527645000	-1.221253000
C	-3.277401000	0.013786000	-1.165951000
C	-2.646215000	0.221276000	0.073180000
C	-3.325640000	-0.120072000	1.251307000
H	-5.137382000	-0.901883000	2.115705000

H	-6.241148000	-1.269860000	-0.084182000
H	-5.038659000	-0.689151000	-2.184176000
H	-2.767475000	0.272425000	-2.090201000
H	-2.842463000	0.028349000	2.213167000
C	-1.256356000	0.808079000	0.151246000
H	-0.917769000	0.808927000	1.188546000
C	-1.137950000	2.215134000	-0.454544000
H	-1.620247000	2.903624000	0.249858000
H	-1.719341000	2.255864000	-1.381013000
C	0.286604000	2.696521000	-0.777405000
H	0.216819000	3.707034000	-1.196163000
H	0.716659000	2.076030000	-1.570787000
C	1.261428000	2.781660000	0.382291000
H	2.231824000	3.157175000	0.047107000
H	0.881577000	3.414976000	1.189942000
O	-0.310203000	-0.076230000	-0.576379000
H	-0.718192000	-0.932042000	-0.790929000
O	1.456958000	1.448513000	0.964946000
H	2.160935000	1.480832000	1.632839000
Fe	1.617100000	-0.309598000	-0.060132000
Cl	1.649644000	-2.079364000	-1.462567000
Cl	1.386039000	-1.432507000	1.965301000
Cl	3.827589000	0.055222000	-0.341230000

A_{1d}-spin6

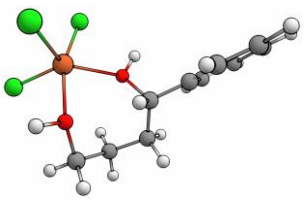

C	-4.580826000	-0.771379000	1.248163000
C	-5.252536000	-0.917422000	0.030836000
C	-4.644821000	-0.489000000	-1.154468000
C	-3.373631000	0.089321000	-1.122319000
C	-2.690033000	0.238019000	0.096907000
C	-3.304005000	-0.203630000	1.278341000
H	-5.045504000	-1.103779000	2.171727000
H	-6.243289000	-1.361526000	0.004578000
H	-5.161892000	-0.599865000	-2.103004000
H	-2.919897000	0.424680000	-2.051127000
H	-2.781851000	-0.099313000	2.225703000
C	-1.312098000	0.857200000	0.161515000
H	-0.985469000	0.890810000	1.200437000
C	-1.211987000	2.257977000	-0.459226000
H	-1.736908000	2.940949000	0.220511000
H	-1.757995000	2.280351000	-1.407845000
C	0.215552000	2.767941000	-0.723185000
H	0.146763000	3.797423000	-1.093939000
H	0.672571000	2.189609000	-1.534419000
C	1.150095000	2.805314000	0.475927000
H	2.130499000	3.195619000	0.186328000
H	0.738795000	3.423110000	1.281439000

O	-0.336745000	-0.031249000	-0.515376000
H	-0.775829000	-0.798817000	-0.914582000
O	1.325548000	1.459782000	1.015724000
H	1.909620000	1.486799000	1.788807000
Fe	1.681549000	-0.431590000	-0.053128000
Cl	1.409314000	-2.275531000	-1.410230000
Cl	1.926489000	-1.369379000	2.004451000
Cl	3.735104000	0.310850000	-0.687811000

B_{1d}-spin2

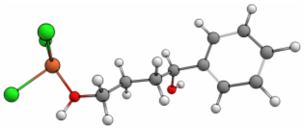

C	-6.342182000	-0.693446000	-0.631338000
C	-6.820910000	0.621064000	-0.690705000
C	-6.020844000	1.669979000	-0.228301000
C	-4.748355000	1.404170000	0.289980000
C	-4.258234000	0.092437000	0.347315000
C	-5.069793000	-0.955198000	-0.117143000
H	-6.960150000	-1.513826000	-0.985832000
H	-7.810755000	0.823785000	-1.089707000
H	-6.386963000	2.692215000	-0.263980000
H	-4.132896000	2.223166000	0.655054000
H	-4.706532000	-1.977678000	-0.065743000
C	-2.863023000	-0.182193000	0.886867000
H	-2.494828000	0.731530000	1.376211000
C	-1.881419000	-0.570837000	-0.226265000
H	-2.235176000	-1.503657000	-0.684100000
H	-1.921048000	0.201973000	-1.002636000
C	-0.437928000	-0.741248000	0.271735000
H	-0.063451000	0.218090000	0.654729000
H	-0.408338000	-1.460167000	1.095959000
C	0.459308000	-1.233058000	-0.850880000
H	0.484074000	-0.547196000	-1.701292000
H	0.167223000	-2.228783000	-1.192365000
O	1.839770000	-1.359354000	-0.352038000
H	2.299788000	-2.127291000	-0.729662000
O	-2.858574000	-1.261670000	1.837738000
H	-3.474432000	-1.045166000	2.551590000
Fe	3.058717000	0.082275000	0.095861000
Cl	2.605399000	1.422655000	-1.591681000
Cl	4.042797000	1.474392000	1.454886000
Cl	4.773425000	-1.257516000	-0.292839000

B_{1d}-spin4

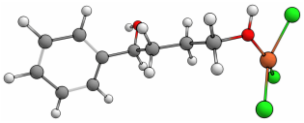

C	-6.399541000	-0.683263000	-0.477832000
C	-6.890348000	0.612805000	-0.280981000

C	-6.054989000	1.592930000	0.263813000
C	-4.736872000	1.276172000	0.609735000
C	-4.234752000	-0.017230000	0.409251000
C	-5.080455000	-0.995230000	-0.137145000
H	-7.044210000	-1.451075000	-0.896779000
H	-7.915950000	0.854204000	-0.545489000
H	-6.429308000	2.599713000	0.426979000
H	-4.094558000	2.040485000	1.041393000
H	-4.705249000	-2.003669000	-0.285942000
C	-2.787988000	-0.338169000	0.753696000
H	-2.413594000	0.440042000	1.435305000
C	-1.894616000	-0.360052000	-0.494109000
H	-2.268151000	-1.135076000	-1.175648000
H	-2.009959000	0.601469000	-1.008414000
C	-0.413632000	-0.610760000	-0.171432000
H	-0.050540000	0.152430000	0.528679000
H	-0.303061000	-1.585554000	0.314177000
C	0.434239000	-0.566798000	-1.432307000
H	0.443971000	0.422741000	-1.889585000
H	0.113192000	-1.301938000	-2.175199000
O	1.842413000	-0.868078000	-1.148536000
H	2.018883000	-1.823735000	-1.124622000
O	-2.654548000	-1.622409000	1.384104000
H	-3.203651000	-1.633209000	2.180456000
Fe	3.180994000	0.128524000	-0.103006000
Cl	2.575040000	2.080354000	-1.028357000
Cl	4.324619000	1.022929000	1.576200000
Cl	4.277231000	-1.871342000	0.023461000

B_{1d}-spin6

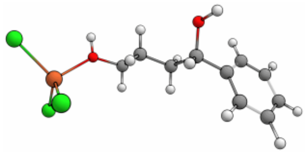

C	6.403611000	0.109221000	-0.873893000
C	6.714393000	-1.120390000	-0.282264000
C	5.792350000	-1.726387000	0.577301000
C	4.568393000	-1.103739000	0.842480000
C	4.245839000	0.124910000	0.248329000
C	5.176682000	0.726398000	-0.612036000
H	7.116878000	0.588277000	-1.538991000
H	7.668330000	-1.598837000	-0.485182000
H	6.027571000	-2.677406000	1.047062000
H	3.859536000	-1.576026000	1.519018000
H	4.941449000	1.683805000	-1.067619000
C	2.892821000	0.767477000	0.517432000
H	2.497483000	0.356102000	1.458068000
C	1.882399000	0.467603000	-0.598881000
H	2.248211000	0.916728000	-1.531017000
H	1.862506000	-0.617995000	-0.751272000
C	0.467560000	0.971395000	-0.272096000
H	0.144912000	0.557121000	0.689935000

H	0.474603000	2.062779000	-0.177057000
C	-0.526026000	0.552564000	-1.344737000
H	-0.564161000	-0.531309000	-1.468705000
H	-0.316981000	1.010970000	-2.315264000
O	-1.902736000	0.930675000	-0.988946000
H	-2.002118000	1.894732000	-0.935243000
O	2.979980000	2.196500000	0.627191000
H	3.584007000	2.414224000	1.350834000
Fe	-3.269087000	-0.187676000	0.026275000
Cl	-2.159073000	-1.131565000	1.728309000
Cl	-4.666211000	1.438556000	0.661384000
Cl	-4.167189000	-1.673773000	-1.377545000

C_{1d}-spin2

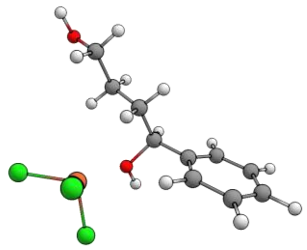

C	3.84367000	-0.87021100	1.31334700
C	4.98785200	-0.66263500	0.53335600
C	4.88704800	0.02489500	-0.67904900
C	3.64484000	0.50231200	-1.10963100
C	2.49703700	0.30680200	-0.32844100
C	2.60514100	-0.38569200	0.88877100
H	3.91678900	-1.40866600	2.25370600
H	5.95028900	-1.03829600	0.86844700
H	5.76922500	0.18625100	-1.29155000
H	3.56972800	1.03376000	-2.05505200
H	1.72277600	-0.55457200	1.49814100
C	1.17884200	0.87761300	-0.79977700
H	1.30470200	1.29944200	-1.80207900
C	0.56933900	1.92873400	0.12317400
H	0.25148900	1.44841200	1.05603400
H	1.38562500	2.60939000	0.39134100
C	-0.57642300	2.73726600	-0.50323700
H	-0.21018600	3.26441800	-1.39307700
H	-1.39096400	2.08406000	-0.83655400
C	-1.14712000	3.75715400	0.47470100
H	-0.35722200	4.44698800	0.80521000
H	-1.54582200	3.24763500	1.36313300
O	-2.18925000	4.47864300	-0.19497100
H	-2.58289100	5.10509100	0.42676300
O	0.19243200	-0.22733500	-0.98939100
H	0.57313300	-0.90234700	-1.57667300
Fe	-1.47739700	-0.92830500	-0.18616100
Cl	-1.07345700	-0.95375200	1.96477100
Cl	-3.65018700	-0.78638200	-0.12032700
Cl	-1.09465900	-2.96536700	-0.95456200

C_{1d}-spin4

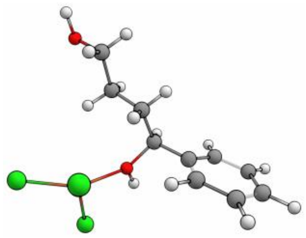

C	3.850620000	-0.916757000	1.386439000
C	4.981712000	-0.838079000	0.564616000
C	4.876416000	-0.273511000	-0.709307000
C	3.643198000	0.209352000	-1.159900000
C	2.509014000	0.142255000	-0.338560000
C	2.621878000	-0.426538000	0.940770000
H	3.926759000	-1.357896000	2.376071000
H	5.936930000	-1.217615000	0.915663000
H	5.748315000	-0.211948000	-1.353884000
H	3.565049000	0.643656000	-2.153526000
H	1.749204000	-0.492732000	1.583264000
C	1.200056000	0.716975000	-0.832473000
H	1.309754000	1.027057000	-1.876800000
C	0.682108000	1.895178000	-0.011743000
H	0.456575000	1.562193000	1.006718000
H	1.531060000	2.585123000	0.070830000
C	-0.512414000	2.641409000	-0.623473000
H	-0.287568000	2.919058000	-1.661198000
H	-1.409299000	2.012770000	-0.646504000
C	-0.845710000	3.904074000	0.162874000
H	0.017463000	4.585115000	0.169899000
H	-1.085688000	3.648506000	1.204563000
O	-1.969949000	4.533421000	-0.466112000
H	-2.211935000	5.318493000	0.042810000
O	0.169820000	-0.361599000	-0.882451000
H	0.446879000	-1.048365000	-1.513599000
Fe	-1.486662000	-0.986234000	0.029762000
Cl	-1.020255000	-0.523222000	2.163394000
Cl	-3.690711000	-0.829666000	0.136275000
Cl	-1.446711000	-2.766645000	-1.383497000

C_{1d}-spin6

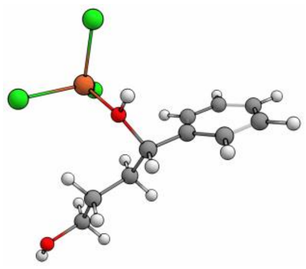

C	3.778087000	-0.378754000	1.304953000
C	4.885626000	-0.173301000	0.473301000
C	4.718550000	0.455391000	-0.763263000
C	3.446402000	0.873771000	-1.167326000

C	2.332505000	0.673862000	-0.339352000
C	2.509542000	0.043158000	0.903208000
H	3.902835000	-0.867041000	2.266966000
H	5.871405000	-0.501926000	0.789322000
H	5.572705000	0.618883000	-1.413692000
H	3.320481000	1.361445000	-2.130869000
H	1.662026000	-0.122371000	1.561848000
C	0.973457000	1.150764000	-0.800194000
H	1.075081000	1.659520000	-1.763479000
C	0.243510000	2.070469000	0.174917000
H	0.026171000	1.539328000	1.109876000
H	0.966197000	2.852103000	0.439872000
C	-1.024219000	2.723773000	-0.392890000
H	-0.774202000	3.280098000	-1.305220000
H	-1.766287000	1.967896000	-0.669847000
C	-1.656154000	3.683348000	0.608791000
H	-0.934840000	4.462383000	0.895376000
H	-1.949099000	3.141257000	1.519059000
O	-2.806518000	4.276045000	-0.008166000
H	-3.236000000	4.859354000	0.631454000
O	0.101628000	-0.027392000	-1.102205000
H	0.479747000	-0.514987000	-1.852627000
Fe	-1.151346000	-1.252490000	-0.019217000
Cl	-1.157517000	-1.394367000	2.218327000
Cl	-3.223398000	-0.789510000	-0.758048000
Cl	-0.553553000	-3.221730000	-0.913708000

TS_{1d}-spin2

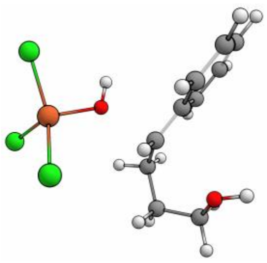

C	-3.793768000	-2.047619000	-1.193374000
C	-3.978649000	-2.749341000	0.006411000
C	-3.289167000	-2.372789000	1.167855000
C	-2.413602000	-1.295742000	1.127743000
C	-2.220949000	-0.568795000	-0.074268000
C	-2.922901000	-0.965184000	-1.238761000
H	-4.329996000	-2.349460000	-2.087069000
H	-4.661501000	-3.593218000	0.035394000
H	-3.436907000	-2.921743000	2.091971000
H	-1.871018000	-0.997090000	2.019500000
H	-2.785447000	-0.430879000	-2.171735000
C	-1.315298000	0.544211000	-0.052750000
H	-0.888247000	0.797476000	0.911197000
C	-1.119416000	1.552511000	-1.127884000
H	-0.060748000	1.519680000	-1.404443000
H	-1.698716000	1.318432000	-2.023563000
C	-1.416734000	2.996464000	-0.646368000

H	-0.740858000	3.253009000	0.176904000
H	-1.196724000	3.678804000	-1.473641000
C	-2.854789000	3.189887000	-0.197679000
H	-3.552706000	2.932282000	-1.005052000
H	-3.024968000	4.236558000	0.086209000
O	-3.059982000	2.329856000	0.932697000
H	-4.006839000	2.199005000	1.076844000
O	0.533977000	-0.583261000	-0.269999000
H	0.387561000	-1.485536000	-0.602310000
Fe	2.244120000	-0.265222000	0.173954000
Cl	2.886717000	-2.414854000	0.094626000
Cl	1.891572000	1.501250000	1.517809000
Cl	3.791105000	0.623256000	-1.146561000

TS_{1d}-spin4

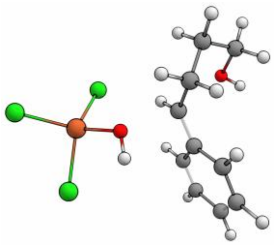

C	3.385165000	2.558786000	-0.790645000
C	3.152749000	3.198372000	0.435743000
C	2.377234000	2.582549000	1.428885000
C	1.828946000	1.329477000	1.192562000
C	2.059562000	0.663462000	-0.038850000
C	2.847368000	1.300168000	-1.030045000
H	3.984364000	3.046653000	-1.552318000
H	3.576586000	4.181599000	0.617061000
H	2.201800000	3.085909000	2.373851000
H	1.218571000	0.845165000	1.948537000
H	3.030556000	0.813777000	-1.981182000
C	1.477099000	-0.630492000	-0.222157000
H	0.911869000	-1.029043000	0.613684000
C	1.714592000	-1.563539000	-1.352080000
H	0.741666000	-1.718881000	-1.831649000
H	2.388466000	-1.144629000	-2.102541000
C	2.224954000	-2.948638000	-0.877639000
H	1.482315000	-3.409994000	-0.217185000
H	2.327986000	-3.594217000	-1.755551000
C	3.555938000	-2.853499000	-0.151159000
H	4.320438000	-2.401818000	-0.796437000
H	3.899868000	-3.852001000	0.147880000
O	3.336638000	-2.037638000	1.009234000
H	4.176500000	-1.671988000	1.318339000
O	-0.599091000	0.070213000	-0.867120000
H	-0.462975000	0.975617000	-1.196056000
Fe	-2.245906000	-0.027823000	-0.086480000
Cl	-2.512326000	2.230872000	-0.357363000
Cl	-1.600292000	-1.575696000	1.456118000
Cl	-4.419528000	-0.624779000	-0.277729000

TS$_{1d}$-spin6

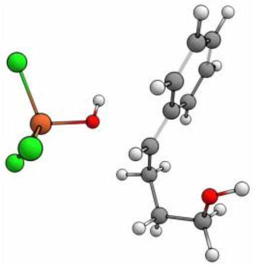

C	-3.684008000	-2.205456000	-1.218630000
C	-3.577542000	-3.046176000	-0.100833000
C	-2.790768000	-2.677560000	0.999974000
C	-2.105430000	-1.470643000	0.979889000
C	-2.204936000	-0.604782000	-0.139835000
C	-3.007498000	-0.992246000	-1.242121000
H	-4.294212000	-2.502642000	-2.065117000
H	-4.109526000	-3.992763000	-0.088734000
H	-2.714316000	-3.334396000	1.860056000
H	-1.485387000	-1.177742000	1.821582000
H	-3.094680000	-0.349364000	-2.110540000
C	-1.477232000	0.626166000	-0.105311000
H	-0.924132000	0.828841000	0.804888000
C	-1.543743000	1.734247000	-1.090766000
H	-0.545464000	1.806016000	-1.536930000
H	-2.249230000	1.526737000	-1.898458000
C	-1.860073000	3.097990000	-0.428311000
H	-1.084167000	3.341638000	0.306203000
H	-1.832298000	3.872139000	-1.201537000
C	-3.219478000	3.097850000	0.250416000
H	-4.015137000	2.885198000	-0.474827000
H	-3.419412000	4.074328000	0.709552000
O	-3.176945000	2.076419000	1.258710000
H	-4.074805000	1.797731000	1.484321000
O	0.551361000	-0.216201000	-0.738330000
H	0.352715000	-1.015614000	-1.250466000
Fe	2.270239000	-0.120997000	-0.027998000
Cl	3.120181000	-2.229904000	-0.122120000
Cl	2.097595000	0.588448000	2.137244000
Cl	3.505328000	1.356029000	-1.263472000

D$_{1d}$-spin2

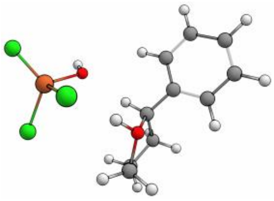

C	-5.059767000	-0.823000000	0.388090000
C	-4.793529000	-2.173631000	0.129998000
C	-3.533510000	-2.559435000	-0.334562000
C	-2.540040000	-1.597278000	-0.539274000
C	-2.804471000	-0.241348000	-0.295929000

C	-4.072923000	0.139435000	0.172265000
H	-6.036363000	-0.521106000	0.754635000
H	-5.565460000	-2.919873000	0.293853000
H	-3.320078000	-3.605740000	-0.531551000
H	-1.554567000	-1.895593000	-0.886019000
H	-4.291666000	1.183237000	0.377958000
C	-1.722744000	0.762442000	-0.561496000
H	-0.817647000	0.271936000	-0.924286000
C	-2.043970000	2.014641000	-1.368287000
H	-1.956364000	1.798821000	-2.435835000
H	-3.065433000	2.354696000	-1.173528000
C	-1.016949000	3.053930000	-0.891885000
H	-0.035289000	2.861243000	-1.333943000
H	-1.312331000	4.076499000	-1.137849000
C	-0.972300000	2.846419000	0.615516000
H	-1.762535000	3.369022000	1.153890000
H	-0.004543000	3.017772000	1.085064000
O	-1.285512000	1.389507000	0.790322000
H	-0.504046000	0.882852000	1.198393000
O	1.071913000	-1.001750000	-0.913273000
H	1.422496000	-1.479897000	-1.684590000
Fe	2.379006000	-0.483602000	0.135706000
Cl	3.899343000	-2.012974000	-0.442484000
Cl	1.051394000	0.044321000	1.989725000
Cl	3.285715000	1.471282000	-0.440626000

D_{1d}-spin4

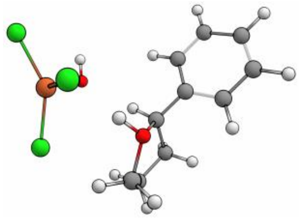

C	-4.831555000	-0.880473000	0.530086000
C	-4.629594000	-2.161901000	0.002068000
C	-3.436149000	-2.463164000	-0.658915000
C	-2.444368000	-1.486160000	-0.789690000
C	-2.646378000	-0.197613000	-0.275900000
C	-3.848045000	0.098765000	0.388298000
H	-5.755624000	-0.644873000	1.049508000
H	-5.399018000	-2.920798000	0.109635000
H	-3.271710000	-3.456573000	-1.065208000
H	-1.511637000	-1.723291000	-1.293894000
H	-4.014557000	1.088254000	0.804326000
C	-1.580895000	0.839259000	-0.475792000
H	-0.697954000	0.408893000	-0.953110000
C	-1.957348000	2.173376000	-1.108705000
H	-1.946149000	2.082724000	-2.197469000
H	-2.963114000	2.477018000	-0.803211000
C	-0.895024000	3.155917000	-0.590428000
H	0.045064000	3.030714000	-1.133777000
H	-1.213500000	4.197045000	-0.679474000

C	-0.712556000	2.764758000	0.871193000
H	-1.415765000	3.246959000	1.549486000
H	0.308284000	2.839836000	1.243061000
O	-1.083084000	1.309938000	0.905812000
H	-0.341132000	0.716059000	1.272288000
O	1.414003000	-0.723391000	-1.519507000
H	1.529035000	-1.656506000	-1.771515000
Fe	2.390970000	-0.365452000	-0.077200000
Cl	3.455018000	-2.354547000	-0.086987000
Cl	1.085649000	-0.425146000	1.926899000
Cl	2.737372000	1.842478000	-0.461511000

D_{1d}-spin6

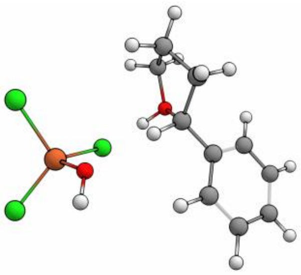

C	-4.953721000	-0.948650000	0.620474000
C	-4.747166000	-2.224895000	0.081657000
C	-3.576043000	-2.500084000	-0.628692000
C	-2.610098000	-1.502971000	-0.796310000
C	-2.817093000	-0.219927000	-0.270851000
C	-3.997685000	0.051309000	0.440137000
H	-5.859807000	-0.733646000	1.179030000
H	-5.495095000	-3.000146000	0.219817000
H	-3.408095000	-3.489037000	-1.044400000
H	-1.692689000	-1.719999000	-1.335903000
H	-4.167279000	1.036726000	0.864609000
C	-1.778626000	0.836515000	-0.506784000
H	-0.890452000	0.415885000	-0.984326000
C	-2.197870000	2.150143000	-1.157899000
H	-2.182210000	2.045903000	-2.245342000
H	-3.213693000	2.424149000	-0.857347000
C	-1.173591000	3.177646000	-0.650055000
H	-0.224315000	3.076176000	-1.183638000
H	-1.524364000	4.206048000	-0.762661000
C	-1.003924000	2.818398000	0.819787000
H	-1.760713000	3.258297000	1.468603000
H	-0.006416000	2.969762000	1.230114000
O	-1.272146000	1.342470000	0.864004000
H	-0.469532000	0.813595000	1.191140000
O	1.114291000	-0.691658000	-1.460599000
H	1.129715000	-1.511743000	-1.976320000
Fe	2.348434000	-0.433630000	-0.148041000
Cl	3.764536000	-2.177567000	0.106415000
Cl	1.108058000	-0.100467000	1.856306000
Cl	3.545253000	1.481035000	-0.483330000

E_{1d}-spin2

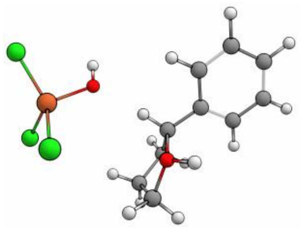

C	-5.473734000	-0.599296000	-0.380448000
C	-5.328776000	-1.921627000	0.054516000
C	-4.060944000	-2.420816000	0.368646000
C	-2.939881000	-1.597329000	0.250880000
C	-3.074818000	-0.268917000	-0.188985000
C	-4.353772000	0.224043000	-0.505328000
H	-6.457281000	-0.211163000	-0.626502000
H	-6.202080000	-2.560530000	0.146278000
H	-3.945165000	-3.446598000	0.704545000
H	-1.953755000	-1.981951000	0.494928000
H	-4.483399000	1.245767000	-0.848586000
C	-1.843303000	0.569317000	-0.291420000
H	-0.937448000	-0.025919000	-0.171820000
C	-1.678815000	1.617846000	-1.375999000
H	-1.241186000	1.151823000	-2.262795000
H	-2.636565000	2.061809000	-1.661286000
C	-0.744515000	2.673697000	-0.761220000
H	0.286278000	2.311774000	-0.732678000
H	-0.766843000	3.617429000	-1.310795000
C	-1.270349000	2.865271000	0.653427000
H	-2.133537000	3.528596000	0.711495000
H	-0.517000000	3.115254000	1.397671000
O	-1.752675000	1.486061000	1.018614000
H	-2.595487000	1.492914000	1.509373000
O	0.951312000	-1.092245000	-0.344410000
H	1.053592000	-1.934686000	-0.819099000
Fe	2.524588000	-0.448451000	0.136952000
Cl	3.722782000	-2.335788000	-0.103429000
Cl	1.869171000	1.021148000	1.733982000
Cl	3.697162000	0.920025000	-1.185232000

E_{1d}-spin4

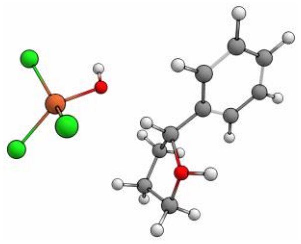

C	-5.157932000	-0.885934000	-0.694230000
C	-5.045846000	-2.040476000	0.089121000
C	-3.862897000	-2.300756000	0.787750000
C	-2.794546000	-1.405865000	0.705862000
C	-2.896024000	-0.246498000	-0.082460000
C	-4.088538000	0.005888000	-0.784630000

H	-6.075029000	-0.683845000	-1.238931000
H	-5.878148000	-2.735155000	0.152292000
H	-3.771565000	-3.196560000	1.394078000
H	-1.873332000	-1.607216000	1.244647000
H	-4.190441000	0.893445000	-1.401504000
C	-1.726409000	0.678798000	-0.137600000
H	-0.849581000	0.252881000	0.345809000
C	-1.361357000	1.416634000	-1.412819000
H	-0.645795000	0.808423000	-1.971177000
H	-2.236613000	1.589899000	-2.044288000
C	-0.747210000	2.748555000	-0.949881000
H	0.267957000	2.600051000	-0.572922000
H	-0.712503000	3.489842000	-1.751394000
C	-1.663480000	3.211982000	0.170409000
H	-2.593242000	3.656140000	-0.187133000
H	-1.195942000	3.828596000	0.935990000
O	-2.018947000	1.919167000	0.847757000
H	-2.931632000	1.902361000	1.191922000
O	0.873743000	-1.085649000	-0.618113000
H	1.048561000	-1.869140000	-1.165518000
Fe	2.408400000	-0.437243000	0.041156000
Cl	3.619979000	-2.197689000	-0.771344000
Cl	1.496197000	0.309384000	1.994771000
Cl	3.957117000	1.222216000	-0.281558000

E_{1d}-spin6

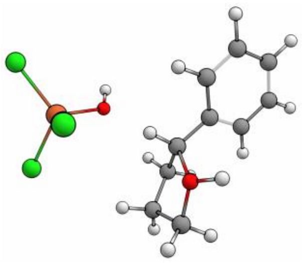

C	-5.077464000	-0.928009000	-0.764380000
C	-4.883203000	-2.201444000	-0.216921000
C	-3.665792000	-2.524659000	0.390138000
C	-2.644935000	-1.574472000	0.452421000
C	-2.827985000	-0.295443000	-0.101089000
C	-4.055368000	0.020491000	-0.711541000
H	-6.021750000	-0.676132000	-1.237103000
H	-5.678579000	-2.939210000	-0.265665000
H	-3.510965000	-3.512199000	0.813441000
H	-1.698675000	-1.823791000	0.923709000
H	-4.220247000	1.001596000	-1.146275000
C	-1.708199000	0.686398000	-0.006793000
H	-0.797431000	0.225559000	0.368729000
C	-1.418893000	1.671543000	-1.124207000
H	-0.682813000	1.219218000	-1.793166000
H	-2.318693000	1.908263000	-1.697862000
C	-0.868800000	2.928391000	-0.427729000
H	0.165015000	2.776005000	-0.106215000
H	-0.901931000	3.807600000	-1.074933000

C	-1.777011000	3.114441000	0.776165000
H	-2.744519000	3.550737000	0.525023000
H	-1.326706000	3.609691000	1.634669000
O	-2.026714000	1.697395000	1.206938000
H	-2.920384000	1.557107000	1.572169000
O	0.967517000	-0.682246000	-1.233364000
H	0.987020000	-1.441393000	-1.834478000
Fe	2.402699000	-0.394926000	-0.141352000
Cl	3.796921000	-2.206613000	-0.138939000
Cl	1.586731000	-0.028308000	1.982701000
Cl	3.512815000	1.499650000	-0.807988000

Supplementary references

1. Bunce, R. A., Herron, D. M., Lewis, J. R. & Kotturi, S. V. *J. N*-phenyl-substituted pyrrolidines, piperidines and azabicyclics by a tandem reduction-double reductive amination reaction, *Heterocyclic Chem.* **40**, 113-120 (2003).

2. Kang, J. Y. & Connel, B. T. Chromium-catalyzed homoaldol equivalent reaction employing a nucleophilic propenyl acetate, *J. Am. Chem. Soc.* **132**, 7826-7827 (2010).

3. Coric, I., Mueller, S. & List, B. Kinetic resolution of homoaldols via catalytic assymetric transacetalization. *J. Am. Chem. Soc.* **132**, 17370-17373 (2010).

4. Filippi, J., Fernandez, X., Loiseau, A., Lizzani-Cuvelier, L. & Meierhenrich, U. J. Enantiomer separation of 1,4-sulfanylalcohols by conventional and low-temperature gas chromatography, *Chirality*, **18**, 558-61 (2006).

5. Gong, J. & Fuchs, P. L. Alkynylation of C-H bonds via reaction with acetylenic triflones. *J. Am. Chem. Soc.* **118**, 4486-4487 (1996).

6. Mortelmans, C. & Van Binst, G. Synthesis of 9,10-dihydro-8bH-quino[1,2-f]phenanthridine- and 6-phenyl-4,5,6,7-tetrahydropyrido[3,2,1-j,k]-carbazole-derivative, *Tetrahedron*, **34,** 363-369 (1978).

7. Bell, M.R., Zalay, A. W., Oesterlin, R., Schane, P. & Potts, G. O. Basic ethers of 1-(p-hydroxyphenyl)-2-phenyl-1,2,3,4-tetrahydroquinoline and 1-(p-hydroxyphenyl)-2-phenylindole. Antifertility agents. *J. Med. Chem.* **13**, 4, 664–668 (1970).

8. Evans, P. A. & Oliver, S. Regio- and enantiospecific rhodium-catalyzed allylic substitution with an acyl anion equivalent. *Org. Lett.* **15**, 5626-5629 (*2013*).

9. Stymiest, J. L., Bagutski, V., French, R. M. & Aggarwal, V. K. Enantiodivergent conversion of chiral secondary alcohols into tertiary alcohols, *Nature*, **456(72223)**, 778-82 (2008).

10. Yokoyama Y., Takagi N., Hikawa H., Kaneko S., Tsubaki N., & Okuno H. Chemoselective palladium-catalyzed reaction in aqueous media: selectivity in the reaction of haloanilines with 1,1-dimethylallyl alcohols. *Adv. Synth. Catal.* **349**, 662–668 (2007).

11. Zhiqi H. & Yong H., Diverting C-H annulation pathways: nickel-catalyzed dehydrogenative homologation of aromatic amides. *ACS Catal*. **6**, 7814-7823 (2016).

12. Sawada N., *et al*. Efficient copper-catalyzed coupling of aryl iodides and thiobenzoic acid, *Tet. Lett.* **47**, 6595-6597 (2006).

13. Sletten E. M. & Liotta, L. J. A flexible stereospecific synthesis of polyhydroxylated pyrrolizidines from commercially available pyranoside. *J. Org. Chem.* **71**, 1335-1343 (2006).

14. Bandaru, A. & Kaliappan, K. P. Synthetic utility of sugar-derived cyclic nitrones: A diastereoselective synthesis of linear 4-azatriquinanes. Synlett, **23**, 1473-1476 (2012).

15. Ansaria, A.A. & Vankar, Y. D. Synthesis of pyrrolidine iminosugars, (-)-lentiginosine, (-)-swainsonine and their 8a-epimers from D-glycals. *RSC Adv.* **4**, 12555-12557 (2014).

16. Zeng, J., Zhang, Q., Zhang, H-K., & Chen, A. Practical synthesis of trans-dihyroxybutyrolactols as chiral C4 building blocks and their application to the synthesis of polyhydroxylated alkoids. *RSC Adv.* **3**, 20298-20303 (2013).

17. Frisch, M. J., *et. al*., *Gaussian 09*, revision A.02; Gaussian, Inc.: Wallingford, CT, (2009).

18. Lee, C., Yang, W. & Parr, R. G. Development of the colle-salvetti correlation-energy formula into a functional of the electron density. *Phys. Rev. B.* **37**, 785-789 (1988).

19. Becke, A. D. Densityfunctional thermochemistry. III. The role of exact exchange. *J. Chem. Phys.* **98**, 5648-5652 (1993).

20. Hay, P. J. & Wadt, W. R. Ab initio effective core potentials for molecular calculations. Potentials for K to Au including the outermost core orbitals. *J. Chem. Phys.* **82**, 299-310 (1985).

21. Hehre, W. J., Ditchfield, R. & Pople, J. A. Self-consistent molecular orbital methods. XII. Further extensions of Gaussian-type basis sets for use in molecular orbital studies of organic molecules. *J. Chem. Phys.* **56**, 2257-2261 (1972).

22. Hariharan, P. C. & Pople, J. A. The influence of polarization functions on molecular orbitals hydrogenation energies. *Theor. Chim. Acta*, **28**, 213-222 (1973).

23. Francl, M. M., *et. al.*, Self-consistent molecular orbital methods. XIII. A polarization-type basis set for second-row elements *J. Chem. Phys.*, **77**, 3654-3665 (1982).

24. Miertus, S. & Tomasi, Approximate evaluations of the electrostatic free energy and internal energy changes in solution processes. J. *Chem. Phys.* **65**, 239-245 (1982).

25. Mennucci, B.; Tomasi, J. Continuum solvation models: A new approach to the problem of solute's charge distribution and cavity boundries. *J. Chem. Phys.* **106**, 5151-5158 (1997).

26. Cossi, M., Barone, V., Mennucci, B. & Tomasi, J. Ab initio study of ionic solutions by a polarizable continuum dielectric model. *Chem. Phys. Lett.* **286**, 253-260 (1998).

27. Krishnan, R., Binkley, J. S., Seeger, R. & Pople, J. A. Self-consistent molecular orbital methods. XX. A basis set for correlated wave functions. *J. Chem. Phys.* **72**, 650-654 (1980).

28. McLean, A. D. & Chandler, G. S. Contracted Gaussian basis sets for molecular calculations. I. Second row atoms, Z = 11- 18. *J. Chem. Phys.* **72**, 5639-5648 (1980).

29. Grimme, S., Antony, J., Ehrlich, S. & Krieg, H. A consistent and accurate ab initio parametrization of density functional dispersion correction (DFT-D) for the 94 elements H-Pu. *J. Chem. Phys*. **132**, 154104-19 (2010).

30. Grimme, S., Ehrlich, S. & Goerigk, L. Effect of the damping function in dispersion corrected density functional theory. *J. Comput. Chem.* **32**, 1456-65 (2011).
