# Supplementary material for: Intramolecular substitutions of secondary and tertiary alcohols with chirality transfer by an iron(III) catalyst
Source: Nat Commun. 2019 Aug 23;10:3826. doi: 10.1038/s41467-019-11838-x (PMC6707304; doi:10.1038/s41467-019-11838-x)
Supplement: Supplementary file 1 — Peer Review [file 41467_2019_11838_MOESM1_ESM.pdf]

Reviewers' comments:

Reviewer #1 (Remarks to the Author):

In the manuscript, the authors report a synthetically useful method for the preparation of optically active five and six-membered heterocyclic rings via direct stereoselective substitution of optically active alcohols. There is indeed a gap in methodology that is addressed well in this work The study seems to be carefully designed and executed. The SI seems detailed enough to allow reproduction of the experiments.

However, there are several issues that require major revisions:
1) Scope and Limitations: For a new methodology it is important to elucidate the scope and limitations. Otherwise, chemists interested in applying such conditions have to find out by themselves. Surprisingly, limitations are not mentioned. In the abstract and introduction it is stated that N,O and S-nucleophiles are used for this study. In the actual substrate table, this is not true for all cases. For instance 2k is only reported with the N-nucleophile. What happened in case of S or O? 2l is only reported with the S-nucleophile. see also 2h, 2i, 2m, 2n, 2s, 2t, 2u, 2v etc. Did the other nucleophiles fail in these cases?
The limitations have to be mentioned, to make this study synthetically useful for the community. If the other nucleophiles were not tested yet in these cases, for several examples all nucleophiles have to be explored and the results reported.

2) Overselling: a) in the abstract it is stated "Optically pure alcohols are abundant in nature, but the gate to this potential supply of chiral building blocks is yet to be unlocked for chemical synthesis". This statement is extremely exaggerating and simply wrong. Naturally optically pure alcohols are used all over in organic synthesis, catalysis, total synthesis etc.
Also the sentence in line 339ff should be toned down: "These advancements have unlocked important gates to use nature's supply of readily available alcohols as raw material in the synthesis of important motifs found in biologically active compounds important in pharmaceutical and agrochemical industries."
b) line 190: "Notably, this is the first time a non-activated phenol has been used as a nucleophile in the substitution of an OH group". This statement is so general, that it simply cannot be true after 150+ years of chemistry. Scifinder finds thousands of examples – even intermolecular.

3) Purity of cyclization products. Although the NMR spectra in the SI are for the most part are of very high purity, there are some problematic cases, especially for cyclization products:
a) page S147, 2k: substantial impurities/overlap in the aromatic region. What are these side products/impurities? How are these impurities reflected in the reported yield of 79% and 89%es?
b) S160, 2v: substantial impurities

Minor details:
a) SI: 20. General Scheme I for synthesis of 1v: one carbon is missing in CC and onward. This is generally a problem in the whole SI

b) I would avoid the term "stereospecific" (unfortunately, it seems that this term is used in that field, see Ref 10 etc) since this usually refers to really specific reactions: Stereospecific Reaction: A reaction in which the stereochemistry of the reactant completely determines the stereochemistry of the product without any other option. In contrast to: Stereoselective Reaction: A reaction in which there is a choice of pathway, but the product stereoisomer is formed due to its reaction pathway being more favorable than the others available.

c) Concerning the rate-order determination. Secondary kinetic isotope effects would provide further support (absence for SN2, presence for SN1)

Reviewer #2 (Remarks to the Author):

The manuscript by Repo, Samec, and co-workers describes a direct iron-catalyzed substitution of alcohols with heteroatom nucleophiles. More specifically, acyclic precursors are used in the preparation of aliphatic nitrogen, oxygen, and sulfur heterocycles. The products should be useful and the authors should be commended for expanding the scope to include all of these heterocycles. While I will agree that this is interesting and timely subject matter, I have some issues with the paper. One issue is with novelty. It should be noted that very similar work has been reported by Cossy and is not cited a single time here. See Acc. Chem. Res. 2015, 48, 761−773. This is FeCl3-catalyzed substitution of allylic alcohols and acetates. In fact your calculations use FeCl3 as a model… While the present work is not identical, it is certainly very very closely related and it is hard to imagine how this now somewhat mature body of work could have been missed when determining what to cite. In fact you cited this exact paper here: 10.1002/chem.201705164. In addition to this, there are some other issues as follows:

Terminology- The term stereospecific is used extensively here, but perhaps incorrectly. The term stereospecific should be reserved for reactions whose product stereochemistry is dictated by the stereochemistry of the starting material. Highly stereoselective reactions can appear to be stereospecific, but in reactions where optical purity is lost, these should be termed selective, not specific in my opinion.

What does % e.s. mean? If your starting material is 100 % ee and the product is 90% ee is that 90% es because you lost 10% ee or 95% es (100:0) -> (95:5)? Please define in the footnote of the tables.

The first sentences of the abstract seem to make the problem appear much more herculean than it is in actuality. The direct alcohol substitution problem was solved long ago by Mitsunobu, but there are certainly drawbacks that could be improved upon.

Abstract line 46- "kinetically less favored" is a comparison, but it is not clear what it is being compared to.

Pg 9, Line 182 and line 184- Again kinetically less favored. This is very confusing to me. There is no kinetic problem for 6-membered ring formation.

Pg 9, Line 190 and line 191- Unclear what a non-activated phenol is. Do you mean that a substituent is typically needed to increase the electron density or modulate the acidity?

Kinetics:

-Every occurrence of the SN2 terminology for the mechanism of an intramolecular reaction must be replaced with "SN2-like" as, by definition, it cannot be bimolecular.

- I am not sure that the proper comparisons in are being made when describing the reactions outlined in Figure 3. Different rates for reactions of different substrates can't tell you anything about the order of the reaction. These are different compounds. By definition they react at different rates. It would only be by coincidence if the rates were the same. As an example, the change made from 1d to 1d' is the addition of a hydroxymethyl group. If you had added 1 methyl group instead of a hydroxymethyl group or better yet 2 methyl groups, the reaction should be much faster, but not due to order of the reaction. They are just different compounds that react at different rates for a variety of different

reasons. Period. Inferring more is not scientifically sound and I don't think that these experiments are predicated on first principles.

SI, pg S3 entry 39, “product” is misspelled

In the SI, there is virtually no optical rotation data for most of the compounds. This data absolutely needs to be included in the experimental write-ups and also listed as an item to check off in the characterization check list. It is essential and this raises some questions:

1- A stereochemical proof is needed for both starting materials and products. This may be comparison to known optical rotation data or something else, but data definitely must be included.

2- How do you know that there is inversion if you don't know the absolute configuration?

3- When R/S is specified in the schemes in the SI, where does this come from?

Calculations

I am not a computational chemist, but I have some concerns here nevertheless. Mainly, my question is whether or not using FeCl3 instead of Fe(OTf)3 is valid. Table 1, entries 9 and 14 (chloride and triflate) show very different results. Are the experimental differences observed inconsequential?

Pg 21- lines 455-561. The references numbering seems to start over…

Due to these issues, it is my opinion that, in its current construction, the manuscript does not rise to the scholarly expectation of Nature Communications. I feel that this is a work more suited for a specialized organic chemistry journal like J. Org. Chem.

Our respond to Reviewers:

Reviewer #1 (Remarks to the Author):

In the manuscript, the authors report a synthetically useful method for the preparation of optically active five and six-membered heterocyclic rings via direct stereoselective substitution of optically active alcohols. There is indeed a gap in methodology that is addressed well in this work The study seems to be carefully designed and executed. The SI seems detailed enough to allow reproduction of the experiments.

However, there are several issues that require major revisions:
1) Scope and Limitations: For a new methodology it is important to elucidate the scope and limitations. Otherwise, chemists interested in applying such conditions have to find out by themselves. Surprisingly, limitations are not mentioned. In the abstract and introduction it is stated that N,O and S-nucleophiles are used for this study. In the actual substrate table, this is not true for all cases. For instance 2k is only reported with the N-nucleophile. What happened in case of S or O? 2l is only reported with the S-nucleophile. see also 2h, 2i, 2m, 2n, 2s, 2t, 2u, 2v etc. Did the other nucleophiles fail in these cases?
The limitations have to be mentioned, to make this study synthetically useful for the community. If the other nucleophiles were not tested yet in these cases, for several examples all nucleophiles have to be explored and the results reported.

Answer: This is a very relevant question. All of the compounds that we have synthesized have worked (that is gave conversion and chirality transfers). Each substrate in this table has at least two months of work behind it where the compound needs to be synthesized in enantioenriched and racemic forms and fully characterized. The difference in conservation of chirality from substrate to product varies in the table from 80-99%. This number is showing the scope and limitations of this procedure.

When it comes to the specific question on the allylic substrate 2k, this substrate is a highly activated one and also one of the easier ones to transform. Here, also complementary methodologies such as redox chemistry proceeding through pi-allyls are highly developed. In addition, similar substrates have been transformed by gold and palladium previously. In this submission, this allylic substrate is more to show that we can perform the reaction also with this substrate.

In addition to this, we want to refer to the Angewandte paper (DOI:10.1002/anie.201812894) recently published asap where "only" one nucleophile and one ring-size is reported. With the substrate scope in this submission ranging from 6 types (depending on how you count) of nucleophiles and 5-6 membered rings, benzyl, alkyl, allyl, propargyl alcohols, we have been relatively ambitious.

2) Overselling: a) in the abstract it is stated "Optically pure alcohols are abundant in nature, but the gate to this potential supply of chiral building blocks is yet to be unlocked for chemical synthesis". This statement is extremely exaggerating and simply wrong. Naturally optically pure alcohols are used all over in organic synthesis, catalysis, total synthesis etc.
Also the sentence in line 339ff should be toned down: "These advancements have unlocked important gates to use nature's supply of readily available alcohols as raw material in the synthesis of important motifs found in biologically active compounds important in pharmaceutical and

agrochemical industries."

Answer: We totally agree with the reviewer that is a too bombastic and exaggerated statement. We have rephrased this to what it is all about namely: to use the alcohol as an electrophile (nucloefuge) without derivatization, and taking advantage of that alcohols are easily accessible.

We rephrased these two sentences as follows:

Abstract: Optically pure alcohols are abundant in nature and attractive but truly challenging starting materials for further transformation.

Conclusions: These advancements have enabled a green transformation readily available alcohols as raw material in the synthesis of important motifs found in biologically active compounds important in pharmaceutical and agrochemical industries.

b) line 190: "Notably, this is the first time a non-activated phenol has been used as a nucleophile in the substitution of an OH group". This statement is so general, that it simply cannot be true after 150+ years of chemistry. Scifinder finds thousands of examples – even intermolecular.

Answer: Here again, we agree with the reviewer; the statement is to general. We rephrased the sentence to: Notably, this is the first time a phenol has been used as a nucleophile in the substitution of an OH group with transfer of chirality.

3) Purity of cyclization products. Although the NMR spectra in the SI are for the most part are of very high purity, there are some problematic cases, especially for cyclization products:
a) page S147, 2k: substantial impurities/overlap in the aromatic region. What are these side products/impurities? How are these impurities reflected in the reported yield of 79% and 89%es?
b) S160, 2v: substantial impurities

Answer: This is not side-product. We put a considerable effort in purification of these compounds. These two compounds in particular were very unstable and decomposed while purifying them. The conversions in most cases is quantitative and the losses are made during column chromatography. For the allylic substrate, decomposition also occurred in the NMR tube.

Minor details:
a) SI: 20. General Scheme I for synthesis of 1v: one carbon is missing in CC and onward. This is generally a problem in the whole SI

Answer: thanks, we fixed these typos.

b) I would avoid the term "stereospecific" (unfortunately, it seems that this term is used in that field, see Ref 10 etc) since this usually refers to really specific reactions: Stereospecific Reaction: A reaction in which the stereochemistry of the reactant completely determines the stereochemistry of

the product without any other option. In contrast to: Stereoselective Reaction: A reaction in which there is a choice of pathway, but the product stereoisomer is formed due to its reaction pathway being more favorable than the others available.

Answer: Half the community wants us to use chirality transfer or conservation of chirality and the other half wants us to use stereospecific. I would prefer not to term this stereoselective as this alludes to an induction step. We have included a short description of the terminology a footnote:

"Terminology of such transformation is not settled by the community and both stereospecific and chirality transfer are used. In this article, chirality transfer will be used."

c) Concerning the rate-order determination. Secondary kinetic isotope effects would provide further support (absence for SN2, presence for SN1)

Answer; that was a great idea and we performed such experiment that has been added to the revised manuscript. We find an inverse kinetic isotope effect, a bit lower than we expected. However, there is some precedence that an inverse effect can be observed. Maybe, this is due to a pre-equilibrium. Anyhow, this also support our hypothesis.

We wish to thank the reviewer for this suggestion!

Reviewer #2 (Remarks to the Author):

The manuscript by Repo, Samec, and co-workers describes a direct iron-catalyzed substitution of alcohols with heteroatom nucleophiles. More specifically, acyclic precursors are used in the preparation of aliphatic nitrogen, oxygen, and sulfur heterocycles. The products should be useful and the authors should be commended for expanding the scope to include all of these heterocycles. While I will agree that this is interesting and timely subject matter, I have some issues with the paper. One issue is with novelty. It should be noted that very similar work has been reported by Cossy and is not cited a single time here. See Acc. Chem. Res. 2015, 48, 761−773. This is FeCl3-catalyzed substitution of allylic alcohols and acetates. In fact your calculations use FeCl3 as a model… While the present work is not identical, it is certainly very very closely related and it is hard to imagine how this now somewhat mature body of work could have been missed when determining what to
cite. In fact you cited this exact paper here: 10.1002/chem.201705164. In addition to this, there are some other issues as follows:

Answer: We included the Accounts by Cossy as a reference in the resubmission. In the previous submission, only references to the original articles were included (see both references 13-20; as well as Figure 1a). As Cossy's Accounts also include diastereoselective cyclizations and gives a good overview of S_N1-type substitutions Iron(III), we think that it is a welcomed reference to include and thank the reviewer for this suggestion.

Terminology- The term stereospecific is used extensively here, but perhaps incorrectly. The term stereospecific should be reserved for reactions whose product stereochemistry is dictated by the

stereochemistry of the starting material. Highly stereoselective reactions can appear to be stereospecific, but in reactions where optical purity is lost, these should be termed selective, not specific in my opinion.

Answer: As we answer reviewer one, the community is split. In our JACS paper we termed it chirality transfer which we personally think describes the process the very best way and got the same remarks but opposite, namely that we should term it stereospecific. We don't have a strong preference ourselves. However, we don't want to use stereoselective as this may allude to a reaction with an induction. We have included a footnote:

"Terminology of such transformation is not settled by the community and both stereospecific and chirality transfer are used. In this article, chirality transfer will be used."

What does % e.s. mean? If your starting material is 100 % ee and the product is 90% ee is that 90% es because you lost 10% ee or 95% es (100:0) -> (95:5)? Please define in the footnote of the tables.

Answer: We thank the reviewer for this as it is not trivial. Mainly Carreira uses this terminology. What we mean in the above case is that we lose 10% ee; we included a definition a footnote: Chirality transfer is determined as percentage of conserved ee.

The first sentences of the abstract seem to make the problem appear much more herculean than it is in actuality. The direct alcohol substitution problem was solved long ago by Mitsunobu, but there are certainly drawbacks that could be improved upon.

Answer: We agree to the overselling and this was also a comment by reviewer 1. We have changed this and also a similar phrasing in the conclusions!

Abstract line 46- "kinetically less favored" is a comparison, but it is not clear what it is being compared to.

Answer: we rephrased to "as compared to five-membered rings."

Pg 9, Line 182 and line 184- Again kinetically less favored. This is very confusing to me. There is no kinetic problem for 6-membered ring formation.

Answer: The five membered rings have a more kinetic drive than the six membered rings however thermodynamically, it is the opposite. Please see for example page 241 in Stuart Warren's "The Disconnection Approach" For these reactions with a kinetic challenge, this is very important.

The Angewandte Chemie (DOI:10.1002/anie.201812894) is only disclosing 5-membered rings. We believe they were not able to perform substrates to generate six-membered rings. The same was true in our JACS paper, where we worked hard on this. This is one of the great achievements in this work.

Pg 9, Line 190 and line 191- Unclear what a non-activated phenol is. Do you mean that a substituent is typically needed to increase the electron density or modulate the acidity?

Answer: This is an unclear sentence and we rephrased to: "…with transfer of chirality.

Kinetics:

-Every occurrence of the SN2 terminology for the mechanism of an intramolecular reaction must be replaced with "SN2-like" as, by definition, it cannot be bimolecular.

Answer: We agree and changed accordingly!

- I am not sure that the proper comparisons in are being made when describing the reactions outlined in Figure 3. Different rates for reactions of different substrates can't tell you anything about the order of the reaction. These are different compounds. By definition they react at different rates. It would only be by coincidence if the rates were the same. As an example, the change made from 1d to 1d' is the addition of a hydroxymethyl group. If you had added 1 methyl group instead of a hydroxymethyl group or better yet 2 methyl groups, the reaction should be much faster, but not due to order of the reaction. They are just different compounds that react at different rates for a variety of different reasons. Period. Inferring more is not scientifically sound and I don't think that these experiments are predicated on first principles.

Answer: We agree with the reviewer and have made significant changes in the text. It is a big challenge for this type of reactions to try to distinguish the reaction mechanism using first principles. We did the experiment with one methyl group to rule out the Thorpe-Ingold effect and we got a rate somewhere in between the two. We changed the paragraph to more describe the shortcomings with the experiments:

To elucidate the reaction mechanism of the present transformations, we studied the rate order of the $Fe(OTf)_3$ catalyst. The rate-order was determined by varying the concentration of catalyst in the transformation of ***1a*** *to* ***2a****, and a first-order dependence on catalyst concentration was found (Supplementary Figure S1).*

*The overall rate in an S_N1 reaction is only dependent on the concentration of the electrophile due to the rate-limiting generation of the carbenium ion, whereas an S_N2 reaction is dependent on both the electrophile and nucleophile. As we couldn't vary the concentration of the electrophile and nucleophile, we prepared substrates with two nucleophiles present instead of only one (****1d´*** *and* ***1u´****, Figure 3). Even though this does not show the rate-order, it might give useful information whether the kinetics are governed by the nucleophile. It should be noted that this experimental set-up does not follow first principles and the results should be interpreted with care. As a result, we found that the reaction rate was doubled when two nucleophiles were present on the molecule (****1d´****) instead of only one (****1d****) (Figure 3a-3b). Similarly, the reaction rate with a substrate with two electrophilic moieties was twice that of the standard substrate (Supplementary Figure S2). Moreover, rate constant and deuterium kinetic isotope effect was also measured for substrates* ***1b*** *and* ***1b´*** *(Figure 3e-3f). A deuterium kinetic isotope effect (KIE = k_H/k_D = 0.91) was observed when the hydrogen atom on* ***1b*** *is replaced by deuterium and the reactions were performed under similar conditions. This inverse kinetic isotope effect also support an S_N2-like pathway. Even though the full rate-order is not elucidated, these observations support an S_N2-like reaction mechanism for secondary alcohols where one equivalent of iron is responsible for the catalysis[38].*

To continue our mechanistic investigations, we introduced tertiary alcohols ***1u*** *and its derivative* ***1u´*** *with two nucleophiles for comparison (Figure 3c-3d). Now the reaction rate did not show a rate increase for* ***1u´*** *and indicates that the reaction mechanism for the tertiary alcohol is different than for secondary alcohols, likely following an S_N1 pathway.*

."

SI, pg S3 entry 39, “product” is misspelled

Answer: Corrected.

In the SI, there is virtually no optical rotation data for most of the compounds. This data absolutely needs to be included in the experimental write-ups and also listed as an item to check off in the characterization check list. It is essential and this raises some questions:

1- A stereochemical proof is needed for both starting materials and products. This may be comparison to known optical rotation data or something else, but data definitely must be included.

2- How do you know that there is inversion if you don't know the absolute configuration?

3- When R/S is specified in the schemes in the SI, where does this come from?

Answer: We agree to the need of optical rotation data and we have verified several of our compounds. We have in our JACS paper X-rays of re-crystalized compounds as we were not certain. Also this data has been used in this publication. In all, we are confident that we have a stereoinversion during the substitution. We looked into reports by other groups and only found optical rotation in one of Aponicks first paper. We believe that the community has settled that this occur. Nevertheless, we agree with the reviewer and included this data (SI).

Calculations

I am not a computational chemist, but I have some concerns here nevertheless. Mainly, my question is whether or not using FeCl3 instead of Fe(OTf)3 is valid. Table 1, entries 9 and 14 (chloride and triflate) show very different results. Are the experimental differences observed inconsequential?

Answer: We started out using Fe(OTf)3, however the computations didn’t work. Then we consulted Prof. Fahmi Himo, who is a computational chemist and he explained that Iron(III) is very difficult to compute especially without ligands that are coordinating to the metal. He suggested us to try with chlorides and this gave us much more reliable calculations. Apparently, it is still difficult to calculate naked Iron(III) species, especially without coordinating ligands. The conversion is lower for the chloride species, however the transfer of chirality is similar. The conversion is due to rust formation that is a problem with FeCl3. We think therefore that the calculations are important. However, we agree to the reviewer that this should be explained and added the following in the resubmission:

$FeCl_3$ was chosen as a model to mimic the $Fe(OTf)_3$ used in the experiments as it also was found to promote the substitution (Table 1, entry 9) and $FeCl_3$ gave more coherent results as compared to $Fe(OTf)_3$
.

Pg 21- lines 455-561. The references numbering seems to start over…

Answer: thanks for making us aware of this typo!

Due to these issues, it is my opinion that, in its current construction, the manuscript does not rise to the scholarly expectation of Nature Communications. I feel that this is a work more suited for a specialized organic chemistry journal like J. Org. Chem.

Answer: We disagree with the reviewer on this point. Our previous report on the first direct substitution of the hydroxyl group of enantioenriched allyl, alkyl, propargyl and benzyl secondary alcohols to generate only 5-membered heterocycles with transfer of chirality was published 3 years ago in JACS and has already received 23 citations (Google Scholar) which we think is noteworthy. In this work, we expand the study to 6-membered rings and also to tertiary alcohols where the chirality is transferred from substrate to product. This has never been reported before and is a major achievement. In addition the scope is very broad with respect to the nucleophile where we can use even phenols and still conserve the chirality. Overall, this is the first submitted manuscript of Iron(III) to promote chirality transfer of enantioenriched alcohols. We also want to bring attention to the asap article in Angewandte showing "only" five membered ring formation and one nucleophile demonstrating the impact of the results presented.

Reviewers' comments:

Reviewer #1 (Remarks to the Author):

In my opinion, the authors did a very good job in addressing my concerns from the first round of review. The manuscript is now suited for publication.

Reviewer #2 (Remarks to the Author):

The revised manuscript by Repo and Samec describes a catalytic method for intramolecular substitution of non-racemic alcohol with chirality transfer. My enthusiasm for this paper is tempered by the manner in which it is written with a high level of salesmanship that somehow neglects common chemistry taught to undergraduates. For instance, the first sentence of the abstract states that alcohols are "truly challenging starting materials for further transformation". They are not. Alcohols are just about the least challenging starting materials for further transformations and a plethora of functional group interconversions are known. The second sentence of the abstract states that conversion of alcohols with "conservation of chirality" usually requires multistep procedures; however, the textbook way to transform alcohols with chirality transfer is the Mitsunobu reaction, a one-step reaction. The manuscript received two reviews and both comment on this overselling issue. While the authors stated that they agree, they have not toned it down to an acceptable level and it is off-putting. How does a paper about alcohol substitution make these claims and not cite or even mention the Mitsunobu reaction? Moving past this, the authors do address the issues raised in the reviews, mostly in a satisfactory way.

However, I still have concerns about some of the important issues here, such as making the comparisons between reaction rates of 1d and 1d' because the likelihood is that rate differences are due to entropy. I would be much more receptive to this interpretation with 1u vs 1u', as the conformation is not likely changed in a significant way when moving from one nucleophilic OH to two in this case. Also, I couldn't find supplementary Figure S1 or S2 that describe kinetic experiments. When I search the SI for the word "figure" I only find S4 and S5 on pages 184 and 185. Am I missing these (and 3) or are they not in the document? The KIE study is a nice addition here and I think that, with this, it is acceptable to conclude that experimental evidence points towards an SN2-like mechanism for secondary alcohols. The problem here is really the convention to use SN2 to imply something about mechanism. All this really means is that two molecules are involved in the rate determining step of the reaction. Any reaction, not only nucleophilic substitution. It would be perfectly feasible for these reactions to show SN1 kinetics (one molecule in the rate determining step) and still be a completely stereospecific reaction in the way that the traditional undergraduate SN2 reaction is. I bet that is what would be observed if you made a pair of secondary alcohols with the phenol/bisphenol nucleophiles as in 1u/1u'.

Also, I still object to using the term stereospecific here. Why use this word at all if it is so polarizing that half the papers use it and half do not? The reader really just wants to know what the ee of the starting material and the product is. I would suggest that ee's also be listed along with % chirality transfer.

Minor issue: Pg 3 line 72- replace charged or rewrite to clearly indicate transfer of synthetic intermediates instead of charged in the sense of positive or negative formal charge.

In light of these comments, I can support publication but the SI Figures should be made available for review, and the issues mentioned above should be addressed.

Answer to reviewers' comments:

Reviewer #1 (Remarks to the Author):

In my opinion, the authors did a very good job in addressing my concerns from the first round of review. The manuscript is now suited for publication.

Answer: We thank the reviewer for the positive feedback and also again for the suggestion regarding the KIE experiment that greatly improved the manuscript.

Reviewer #2 (Remarks to the Author):

The revised manuscript by Repo and Samec describes a catalytic method for intramolecular substitution of non-racemic alcohol with chirality transfer. My enthusiasm for this paper is tempered by the manner in which it is written with a high level of salesmanship that somehow neglects common chemistry taught to undergraduates. For instance, the first sentence of the abstract states that alcohols are "truly challenging starting materials for further transformation". They are not. Alcohols are just about the least challenging starting materials for further transformations and a plethora of functional group interconversions are known. The second sentence of the abstract states that conversion of alcohols with "conservation of chirality" usually requires multistep procedures; however, the textbook way to transform alcohols with chirality transfer is the Mitsunobu reaction, a one-step reaction. The manuscript received two reviews and both comment on this overselling issue. While the authors stated that they agree, they have not toned it down to an acceptable level and it is off-putting. How does a paper about alcohol substitution make these claims and not cite or even mention the Mitsunobu reaction? Moving past this, the authors do address the issues raised in the reviews, mostly in a satisfactory way.

Answer: We first of all would like to thank the reviewer for all the efforts given to both give suggestions on phrasings to avoid the undesired salesmanship level and also to critically scrutinize our experimental set-ups and interpretation of these. We also thank the reviewer for acknowledging our response in the revised manuscript in mostly a satisfactory way.

We agree with the reviewer(s) current and previous comments on overselling the work and we are thankful for pointing out the critical parts of the body text. The first sentence in the abstract is revised as follows "…challenging for further transformation using atom efficient and sustainable methodologies, particularly when there is a desire to conserve the chirality."

To avoid discussion about one-step reaction vs. multistep procedures, we revised the second sentence of the abstract is as follows"… it requires derivatization as part of a more complex, stoichiometric procedures (instead of the original "multistep procedures").

Following and agreeing the criticism above, we included Mitsunobu reaction to Introduction and gave the references list. We agree that FGI of alcohols should have references to the most well-known named reaction and in fact, this state-of-the-art transformation nicely demonstrates the benefits of the catalytic system developed herein. The following sentence was included to the revised

manuscript with references "An example of the state-of-the-art transformations is the Mitsunobu reaction[i], which is based on the stoichiometric use of an azodicarboxylate and triphenylphosphine."

However, I still have concerns about some of the important issues here, such as making the comparisons between reaction rates of 1d and 1d' because the likelihood is that rate differences are due to entropy. I would be much more receptive to this interpretation with 1u vs 1u', as the conformation is not likely changed in a significant way when moving from one nucleophilic OH to two in this case. Also, I couldn't find supplementary Figure S1 or S2 that describe kinetic experiments. When I search the SI for the word "figure" I only find S4 and S5 on pages 184 and 185. Am I missing these (and 3) or are they not in the document? The KIE study is a nice addition here and I think that, with this, it is acceptable to conclude that experimental evidence points towards an SN2-like mechanism for secondary alcohols. The problem here is really the convention to use SN2 to imply something about mechanism. All this really means is that two molecules are involved in the rate determining step of the reaction. Any reaction, not only nucleophilic substitution. It would be perfectly feasible for these reactions to show SN1 kinetics (one molecule in the rate determining step) and still be a completely stereospecific reaction in the way that the traditional undergraduate SN2 reaction is. I bet that is what would be observed if you made a pair of secondary alcohols with the phenol/bisphenol nucleophiles as in 1u/1u'.

Answer: We thank the reviewer for insightful comments and acknowledging the experiment that was suggested by reviewer 1 to support an SN2-like mechanism. Also the suggestion using two phenols is very good. Unfortunately, the phenols are too poor nucleophiles here and these reactions were run in less polar solvent mixtures (even secondary) to promote a tight ion pair or a less polar transition state, especially in the case of reactions proceeding to generate six membered heterocycles this was necessary. Therefore, we agree with the reviewer that in this case, this type of substrate will probably give negligible differences in kinetics. We have investigated the possibility to change the phenol to more nucleophilic thiol, however, these substrates are extremely difficult to prepare and we are not certainly sure that they would be strong enough nucleophiles to promote the reactivity. For example, in the case of tertiary substrates having a sulphur centred nucleophile required a less polar solvent mixture.

We agree with the reviewer that we might have been to "black or white" with respect to SN1 or SN2 like reactivity in the previous versions. We included a paragraph about this and added references to early investigations on the reactivity that are borderline SN1 or SN2. We think that this is important as we probably have the whole spectra of reactivity in our substrates (especially oxygen nucleophiles). In addition, we know that SN1 is a competing reaction also for secondary alcohols. If enantioenriched 1-phenylethanol is reacted under the reaction conditions employed (Fe(OTf)3 and DCE) etherification will occur to produce racemic diastereomers (this is reported in the JACS paper in 2015).

We think that we have been pretty modest in the mechanistic part of our revised manuscript. We are only proposing mechanisms based on the experiments that have been performed and also given the inherent weaknesses with the experiments. In this revision, we also include a more scholarly section with respect to substitution reactions. We think that this will be appreciated by the readers of Nature Communication.

The SI is very comprehensive and we understand that all information is difficult to find. We double checked the document and all the figures were present in the previous submission.

Also, I still object to using the term stereospecific here. Why use this word at all if it is so polarizing that half the papers use it and half do not? The reader really just wants to know what the ee of the starting material and the product is. I would suggest that ee's also be listed along with % chirality transfer.

Answer: We fully agree that using one term is better and we have removed the term stereospecific, from the revised manuscript except in the specific footnote. We prefer to only give the chirality transfer as this is the value that we are aiming to obtain in high degree together with the yield. Adding ee (or er) of starting material and product was attempted, however, the table gets very messy and we prefer to keep this information in the SI. If the manuscript contained less substrates, this would have been more feasible. The template for Nature Communication is rather restricted and the table with substrates is already at the limit. In principle we do agree with the reviewer at this point.

Minor issue: Pg 3 line 72- replace charged or rewrite to clearly indicate transfer of synthetic intermediates instead of charged in the sense of positive or negative formal charge.

Answer: Thanks for pointing this out, we have replaced charged with derivatized.

In light of these comments, I can support publication but the SI Figures should be made available for review, and the issues mentioned above should be addressed.

Answer: We have made sure that the pdf shows these figures!

REVIEWERS' COMMENTS:

Reviewer #2 (Remarks to the Author):

The revised manuscript by Repo, Samec, and co-workers describes Fe-catalyzed substitution of alcohols with chirality transfer. The authors did a satisfactory job addressing my concerns and I support publication of this revised draft. I specifically thank the authors for the detailed explanations provided in the rebuttal letter. I feel that this manuscript has improved tremendously throughout the process and that it will be an impactful publication.
